# Molybdenum‐Catalyzed Asymmetric Amination of α‐Hydroxy Esters: Synthesis of α‐Amino Acids

**DOI:** 10.1002/advs.202403437

**Published:** 2025-03-10

**Authors:** Shahida Perveen, Tahir Rahman, Tariq Ali, Lingyun Wang, Junjie Zhang, Ajmal Khan

**Affiliations:** ^1^ Department of Chemistry School of Chemistry Xi'an Key Laboratory of Sustainable Energy Materials Chemistry and MOE Key Laboratory for Nonequilibrium Synthesis and Modulation of Condensed Matter Xi'an Jiao Tong University Xi'an 710049 P. R. China

**Keywords:** amination, hydrogen borrowing, molybdenum, unnatural α‐amino acid, α‐hydroxy ester

## Abstract

Unnatural α‐amino acids are found in a wide variety of bioactive compounds ranging from proteins to pharmaceutical agents to materials science. As a result, the investigation of efficient and simple methods for their synthesis is a major purpose in reaction development. In this study, it is found that a catalyst based on molybdenum, an earth‐abundant transition metal, can facilitate the amination of readily accessible α‐hydroxy esters to afford *N*‐protected unnatural α‐amino acid esters in high yield. This simple process also enables enantioselective amination, which proceeds through cooperative catalysis of chiral molybdenum complex with chiral phosphoric acid (CPA), and complements earlier procedures to the catalytic synthesis of this important class of compounds. The obtained protected α‐amino acid ester products are directly useful or further utilized for the synthesis of commercially available drugs and analogs.

## Graphical abstract

*N*‐protected unnatural α‐amino acids are synthesized from α‐hydroxy esters and amines in a Mo‐catalyzed reaction. The formation of C─N bond takes place in a highly efficient way, which can be rendered asymmetric when a chiral molybdenum‐complex and chiral phosphoric acid (CPA) are used.

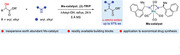

## 1 Introduction

**Scheme 1:**
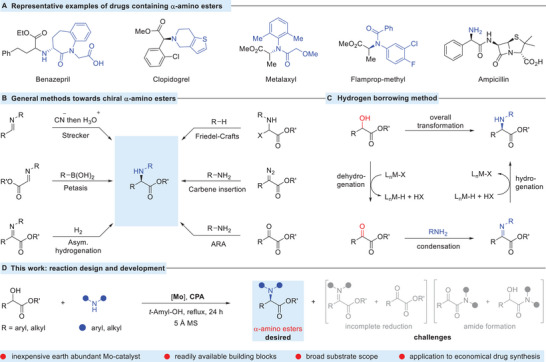
A) Selected examples of drugs containing α‐amino esters B), general methods C), a possible pathway D), and our current approach.

The development of efficient and straightforward methods to access chiral unnatural α‐amino acids is a highly important task, as these amino acids have found widespread use in the fields of biochemistry, pharmaceutical, and materials science (**Scheme** **1**A).^[^ ^1^ ^]^ In addition, they can readily be converted into other useful chiral molecules, such as α‐amino alcohols and α‐amino acid amides.^[^ ^2^ ^]^ Beside the conventional Strecker synthesis,^[^ ^3^ ^]^ catalytic asymmetric procedures to unnatural α‐amino acids are especially important,^[^ ^4^ ^]^ and numerous approaches have been continuously studied. Among the extensively developed methods are nucleophilic addition to α‐imino esters (Friedel Craft reaction),^[^ ^5^ ^]^ electrophilic alkylation of glycine derivatives (Petasis–Mannich reaction),^[^ ^6^ ^]^ carbene insertion,^[^ ^7^ ^]^ hydrogenations of olefins or imines,^[^ ^8^ ^]^ and reductive amination (Scheme 1B).^[^ ^9^ ^]^ Very recently, asymmetric biomimetic amination using α‐keto esters or acids have emerged as promising alternatives to provide enantioenriched α‐amino acids.^[^ ^10^ ^]^

On the other hand, “borrowing hydrogen method” also called hydrogen auto transfer reaction using cheap and readily available alcohols is the most straightforward and highly desirable method since it directly provides chiral *N*‐protected amines.^[^ ^11^ ^]^ This typical process proceeds through dehydrogenation of an alcohol substrate to form a ketone by the involvement of a metal catalyst system, in situ condensation to form a ketimine, and then the resultant imine hydrogenation (Scheme 1C).^[^ ^12^ ^]^ No prior activation is required for alcohol substrates and water molecule is the only sole by‐product. Despite important advances, such reactions have not yet been realized for the amination of α‐hydroxy esters, which are substrates of high importance.^[^ ^13^ ^]^ Undoubtedly, the successful implementation of this chemical reaction would provide an attractive and smooth way to access *N*‐protected unnatural α‐amino esters that would complement the enzyme catalytic reactions.^[^ ^14^ ^]^ However, there are a few challenges to be addressed: for example; a) the in situ formed α‐keto esters are comparatively less stable and might undergo reduction of esters group during the reductive amination step^[^ ^15^ ^]^; b) the amine nucleophile may also react with ester group, leading to amide formation^[^ ^16^ ^]^; c) the strong coordination effect of the amide products might lead to reducing catalyst activity.^[^ ^17^ ^]^

In previous reports on amination of alcohol substrates, achiral catalyst precursors based on expensive noble (rare) metals were mostly studied,^[^ ^18^ ^]^ particularly iridium,^[^ ^19^ ^]^ and or ruthenium.^[^ ^20^ ^]^ Recently, much attention has been given to catalytic transformations of cheap, non‐noble (earth‐abundant) metals and several successful examples have been reported. For example, homogeneous catalysts of manganese,^[^ ^21^ ^]^ molybdenum,^[^ ^22^ ^]^ chromium,^[^ ^22e,f^ ^]^ cobalt,^[^ ^23^ ^]^ copper,^[^ ^24^ ^]^ and iron.^[^ ^25^ ^]^ To date, there are several reports for the preparation of chiral α‐amines through borrowing hydrogen pathway with the use of expensive and noble metals catalyst,^[^ ^26^ ^]^ but to our knowledge, no such reaction has been realized for the catalytic asymmetric preparation of *N*‐protected unnatural α‐amino acid esters.^[^ ^27^ ^]^

Inspired by previous work and current opportunities in the field,^[^ ^27a^ ^]^ and our recent developments in group VI metal‐catalyzed reaction to form carbon‐heteroatom bonds,^[^ ^28^ ^]^ we became interested in the catalytic reaction of α‐hydroxy esters with amines. Herein, we describe the realization of this idea and report an unprecedented, enantioconvergent amination of α‐hydroxy esters that affords a wide variety of chiral *N*‐protected α‐amino acids bearing different aryl or alkyl α‐substituents. The reaction requires special conditions like catalyst loading, additives, solvent, and temperature to provide imine formation and then reduction without competing side products (Scheme 1D). Under the cooperative catalysis of inexpensive molybdenum complex^[^ ^29^ ^]^ and a chiral phosphoric acid (CPA),^[^ ^30^ ^]^ this system successfully delivers a variety of chiral α‐amino acid esters from simple prochiral α‐hydroxy esters (alcohol) and amines. The synthetic importance of our reaction has also been demonstrated in the synthesis of chiral aliphatic amine‐derived drug benazepril (ACS inhibitors),^[^ ^31^ ^]^ flamprop‐methyl,^[^ ^7i^ ^]^ and ampicillin) (Scheme 1A).^[^ ^32^ ^]^

## 2 Results and Discussion

Regardless of much exertion in catalyst development for the amination of (prochiral) alcohols, a practical and general coupling of α‐hydroxy‐α‐substituted esters and amines was yet to be discovered. Toward this purpose, we first investigated the amination of α‐hydroxy‐α‐phenyl ethyl acetate (**1a**) with *p*‐anisidine (**2a**). Several inexpensive and commercially available (group VI) metal carbonyls as a catalysts were examined in tertiary amyl alcohol, which is the preferred solvent in such reactions.^[^ ^33^ ^]^ Our preliminary experiments with the standard substrates revealed that most of the hexacarbonyl complexes did not yield the desired α‐amino ester product, except for a low‐yielding result with Mo(CO)_6_ and Ru_3_(CO)_12_ (Table S1, Supporting Information). Following this, different additives (acid or base) were studied using these carbonyl complexes (Table S2, Supporting Information).^[^ ^34^ ^]^ The use of bases such as *t*‐BuOK,^[^ ^35^ ^]^ resulted from amide formation. On the other hand, the use of a simple phosphoric acid led us to isolate the desired α‐amino ester product in an acceptable yield (Phosphoric acid presumably operates by providing activation of ketone and ketoimine intermediates towards imine condensation and reduction.^[^ ^36^ ^]^). At this stage, the use of phosphoric acid was also evaluated with Ru‐based catalysts, yet it led to a low reaction outcome. To further enhance the reaction efficiency, various commercially available ligands were examined (Table S3, Supporting Information). After several experimentation, we found that ligand with *N*,*N*‐bipyridine backbone resulted in a lower yield, while phosphine‐based ligands resulted in high yield. Mo(CO)_6_, dppb, and phosphoric acid were then selected as catalyst combinations for our subsequent studies.^[^ ^34^ ^]^

**Scheme 2:**
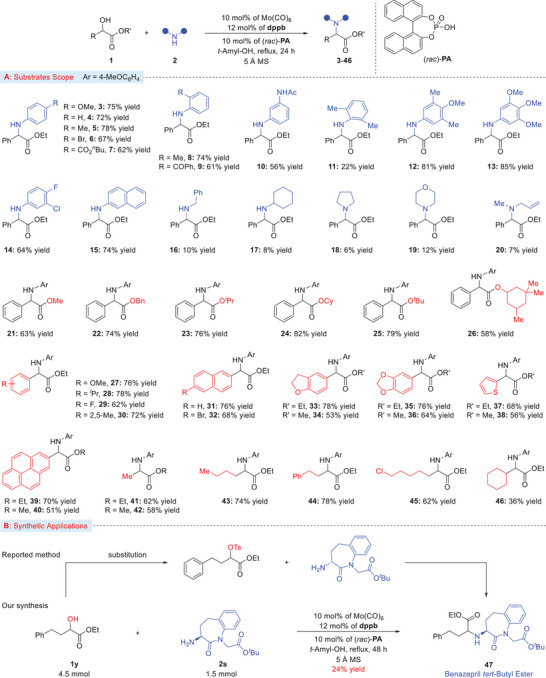
A) Substrate scope for amination of α‐hydroxy esters with different amines. B) Application to one‐step synthesis of Benazepril *tert*‐butyl ester. Reaction conditions: Mo(CO)_6_ (10 mol%), dppb (12 mol%), (*rac*)‐PA (10 mol%), α‐hydroxy esters **1** (0.6 mmol), amine **2** (0.2 mmol), 5 Å MS (80–100 mg), *tert*‐Amyl‐alcohol (2.0 mL), refluxing temperature (140–150 °C), 24 h. All yields are of isolated products.

The substrate scope of this molybdenum‐catalyzed amination of α‐hydroxy esters turned out to be very broad as summarized in **Scheme** **2**. A variety of amines (both aromatic as well as aliphatic) and α‐hydroxyl esters were efficiently converted into the expected amino esters (products, **3**–**20**). Both electron‐donating and electron‐withdrawing groups on *para*, *meta* as well as on *ortho* positions on the phenyl ring of anilines were investigated (products, **3**–**14**). More specifically, amines with electron‐donating groups in the aryl ring on the *para* position provided the desired α‐amino ester products in high yields, as compared to the electron‐withdrawing group substituted aniline. Sterically hindered *ortho*‐substituted methyl aniline provided a low yield of the α‐amino ester (product, **11**). Polycyclic naphthyl amine could also be coupled to isolate the desired product in high yield (product, **15**).

Furthermore, we were quite excited about the reactivity while using aliphatic amine. For this purpose, several aliphatic (both acyclic and cyclic) amines were examined, however; the current optimized conditions were not able to provide the desired α‐amination products with high yields (products, **16**–**20**). Next, we investigated the α‐hydroxy‐α‐substituted (both aryl and alkyl) esters substrate scope of this catalytic amination approach. For this purpose, we selected *p*‐methoxyphenyl (PMP) amine with different α‐substituted α‐hydroxy esters. By changing the ester groups cleared no specific influence on the reactivity (products, **21**–**25**), except for methyl ester which provided the desired α‐amino ester product with a comparatively low yield (product, **21**).^[^ ^37^ ^]^ Notably, cyclohexyl‐based cyclandelate ester also provided a product with a high yield (product, **26**). Esters with different electronic nature on the aromatic ring were tolerated, affording the desired aryl α‐amino ester products in good to high yields (products, **27**–**30**). Disubstituted and Naphthyl‐based esters were also exposed to the catalytic amination protocol, providing the corresponding α‐amino ester products in good to high yields (products, **31**–**36**). The reaction was also applicable to heteroaryl substituted α‐hydroxy esters affording the desired products in high yields (products, **37**, **38**). Pyrene‐based fused aromatic ring‐derived α‐hydroxy esters were also applicable, afforded products **39** and **40** in good yields.

Unnatural aliphatic substituted α‐amino esters are important intermediates for many pharmaceuticals, and several aliphatic α‐hydroxy esters were thus investigated. As shown in Scheme 2, α‐hydroxy esters bearing different alkyl substituents (methyl, *n‐*butyl, phenyl‐substituted ethyl, and chloro‐substituted *n*‐pentyl) reacted smoothly and provided the desired α‐amino esters in good to high isolated yields (products, **41**–**45**). Sterically hindered α‐hydroxy ester was also compatible and only 36% of α‐amino ester was isolated (product, **46**). The synthetic importance of this catalytic α‐hydroxy ester amination procedure appeared in the one‐step synthesis of commercial drug benazepril (Scheme 2B). The reported (patented) synthetic rout requires stepwise protection of α‐hydroxy ester (**1y**) with sulfonyl chloride (Ts, Ms, Ns, etc.) followed by substitution with the corresponding amine.^[^ ^31^ ^]^ On the other hand, our synthesis of **Benazepril** was realized utilizing the same starting materials in a single catalytic step in 24% yield. Although, the product was isolated in comparatively low yield, however, these results are encouraging toward the first catalytic one‐step synthesis of these challenging drug candidates.

**Scheme 3:**
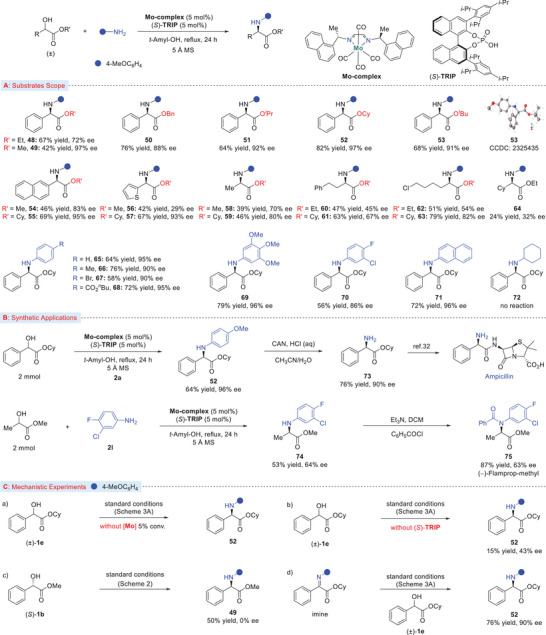
A) Substrate scope for asymmetric amination of α‐hydroxy esters. B) Scaled‐up reaction and synthesis of ampicillin and (−)‐flamprop‐methyl drugs. C) Mechanistic experiments. General reaction conditions for substrate scope evaluation were: α‐hydroxy esters **1** (0.6 mmol), amine **2** (0.2 mmol), Mo‐complex (5 mol%), (*S*)‐TRIP (10 mol%), 5Å MS (100–150 mg), *tert*‐Amyl‐alcohol (0.1 m), refluxing temperature (140–150 °C), 24 h. All yields are of isolated products. Enantiomeric excess (ee values) was determined by using the chiral stationary phases.

To our knowledge, α‐hydroxy‐α‐substituted esters or their derivatives have not been utilized as electrophiles in catalytic asymmetric amination reactions,^[^ ^27a^ ^]^ although they have been employed as precursors to the synthesis of α‐amino esters through protection and then substitution with corresponding amines.^[^ ^15^ ^]^ Toward this goal, we chose a commercially available α‐hydroxy ester **1a** and tried to developed the first asymmetric catalytic amination procedure for the synthesis of α‐amino esters. After several experiments (Table S4, Supporting Information), when this prochiral α‐hydroxy ester was treated with *p*‐anisidine in the presence of chiral (*N*,*N*)‐Mo(CO)_4_ complex and (*S*)‐TRIP as co‐catalyst, amination proceeds to generate the expected PMP protected α‐amino ester in good yield and 72% enantioselectivity (**Scheme** **3**, product, **48**). To our delight, altering the ester groups demonstrated a clear increase in enantioselectivity (products, **49**–**53**). By changing the ethyl ester to the methyl group provided high enantioselectivity with a slightly lower isolated yield (product, **49**). Other ester groups like benzyl, isopropyl, cyclohexyl, and *tert*‐butyl were also evaluated and the desired α‐amino ester products were isolated with high yield and excellent enantioselectivity, among which cyclohexyl‐based ester provided the best results (products, **50**–**53**); while amide and or aryl (phenyl) substituted esters were incompatible (Table S5, Supporting Information). The reaction was applicable to naphthyl‐ and heteroaryl‐substituted α‐hydroxy esters afforded the expected amino esters products in high yields and enantioselectivity (products, **54**–**57**). Furthermore, several α‐alkyl‐α‐hydroxy esters were examined, and smooth conversion to their corresponding α‐amino esters was noticed. However, comparatively low enantioselectivities were obtained (products, **58**–**64**). Excellent results were also achieved while replacing the *p*‐anisidine (PMP) with other aryl amines (products, **65**–**71**). Due to the steric hindrance of cyclohexyl amine, it was not compatible to provide the desired amination product **72**. The absolute configuration (*R*) of α‐amino ester product **53** was confirmed by X‐ray diffraction technique.

To demonstrate the practicality of the current approach, our catalytic amination can be scaled up (2 mmol scale) with the isolation of α‐hydroxy ester **52** in 64% yield with no appreciable loss in enantioselectivity (Scheme 3B). Deprotection of the PMP group in the presence of CAN (cerium ammonium nitrate) followed by amidation of the ester group with aminopenicillanic acid could provide a synthesis of Amplicillin.^[^ ^32^ ^]^ In order to further demonstrate the synthetic utility of the current protocol, we next employed this simple amination procedures to the total synthesis of flamprop‐methyl (Scheme 3B).^[^ ^38a^ ^]^ The amination product **74** was formed on 2‐mmol scale with 64% of isolated yield, which was then simply reacted with benzoyl chloride to provide flamprop‐methyl (product **75**) in 87% of isolated yield with no appreciable loss in enantiopurity. This process can also be applied to the total synthesis of similar flamprop‐isopropyl from isopropyl‐based α‐hydroxy ester as the starting material under otherwise identical conditions.^[^ ^38b^ ^]^ This catalytic method will open new opportunities and will provide direct access to chiral *N*‐protected α‐amino ester‐based drugs and related compounds from simple α‐hydroxyl esters and amines.

Considering the mechanism of this catalytic amination reaction, several control experiments were performed and the results are summarized in Scheme 3C. The reaction of α‐hydroxyl ester **1e** was first investigated. Standard reaction conditions without adding molybdenum complex resulted in no product formation, while the reaction without the phosphoric acid co‐catalyst provided α‐amino ester product **52** in only a trace amount with 43% of enantiopurity (Scheme 3C). To further shed light on the reaction mechanism, we carried out two similar reactions for the formation of α‐amino ester **49** (Scheme 3C). When racemic α‐hydroxy ester **1b** was exposed to the standard reaction condition (Scheme 3A, for asymmetric amination), α‐amino ester **49** was isolated with the same yield and enantioselectivity. On the other hand, amination of enantiopure α‐hydroxy ester (*S*)−**1b** under the standard condition (Scheme 2, for symmetric amination) provided product **49** in 50% yield with no chirality transferred, presumably reduction of imine intermediate. Next, we examined the reaction of free‐formed ketoimine ester in the presence of α‐hydroxy ester **1e** under standard conditions (Scheme 3A) and the desired α‐amino ester product **52** was isolated in good yield and enantioselectivity, suggesting that the amination proceeded through hydrogen borrowing or hydrogen auto transfer mechanism with imine as the key reaction intermediate under the cooperative catalysis of molybdenum and chiral phosphoric acid (CPA).

**Figure 1:**
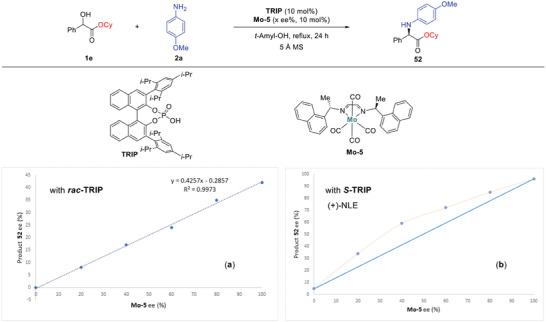
Results of nonlinear effect studies.

To gain further insight into the relationship between enantiopurity of the catalyst and product and the environment between the two chiral catalysts (Mo‐5 and TRIP), nonlinear effects (NLEs) were investigated.^[^ ^39^ ^]^ Initially, we varied the enantiomeric purity of the molybdenum catalyst (**Mo‐5**) from 0 % to 100 % ee with an achiral phosphoric acid (*rac*)‐TRIP. As shown in **Figure** **1**a, a plot of ee_product_ as a function of ee_Mo‐5_ was found to be linear, suggesting that one chiral Mo‐catalyst is functional in the catalytic cycle. In addition, the combination of the optically pure **Mo‐5** (preferentially *S*‐**Mo‐5**) and achiral phosphoric acid (*rac*)‐TRIP gave enantioselectivity (42 % ee). These results suggest that the chiral environment of the molybdenum catalyst is important to control the stereoselectivity of the reaction. Next, we kept the chiral phosphoric acid (*S*)‐TRIP enantiopure while changing the optical purity of the molybdenum catalyst (**Mo‐5**). As shown in Figure 1b, a positive nonlinear effect was observed. We interpreted these results as evidence for the possible collaboration between chiral phosphoric acid (*S*)‐TRIP and molybdenum catalyst.^[^ ^36^ ^]^ These results further suggest the involvement of more than one chiral component in the enantio‐determining step.

## 3 Conclusion

In conclusion, we have developed a general and efficient coupling of α‐hydroxy esters and simple amines through borrowing hydrogen (BH) pathway by adopting a cooperative catalysis method using an inexpensive molybdenum hexacarbonyl and phosphoric acid (PA) as cocatalyst. More significantly, this unprecedented and simple protocol also enables enantioselective amination of α‐substituted‐ α‐hydroxy esters. Using a chiral molybdenum complex with chiral phosphoric acid (CPA), a wide range of valuable chiral α‐amino esters (an important family of target molecules) were isolated in good yield and enantioselectivity from easily accessible α‐hydroxy esters and amines. The usefulness of this newly developed carbon–nitrogen bond formation methodology has been illustrated by its application to the straightforward synthesis of several commercial drugs and analogs. Further investigation to develop additional catalytic enantioselective procedures based on molybdenum (an earth‐abundant transition metal) is underway.

## 4 Experimental Section

Essential Experimental Procedures/Data. (All other characterization data, original spectra, etc., should be provided in the Supporting Information).

## Conflict of Interest

The authors declare no conflict of interest.

## Supporting information

Supporting Information

## Data Availability

The data that support the findings of this study are available in the supplementary material of this article.

## References

[1] a) V. A. Soloshonok , K. Izawa , Asymmetric Synthesis and Application of α‐Amino Acids, American Chemical Society, Washington 2009;

[2] a) D. J. Ager , I. Prakash , D. R. Schaad , Chem. Rev. 1996, 96, 835; 11848773 10.1021/cr9500038

[3] a) H. Groger , Chem. Rev. 2003, 103, 2795; 12914481 10.1021/cr020038p

[4] a) C. Najera , J. M. Sansano , Chem. Rev. 2007, 107, 4584; 17915933 10.1021/cr050580o

[5] a) S. Saaby , X. Fang , N. Gathergood , K. A. Jorgensen , Angew. Chem., Int. Ed. 2000, 39, 4114; 10.1002/1521-3773(20001117)39:22<4114::aid-anie4114>3.0.co;2-v 11093224

[6] a) N. R. Candeias , R. Montalbano , P. M. S. D. Cal , P. M. P. Gois , Chem. Rev. 2010, 110, 6169; 20677749 10.1021/cr100108k

[7] a) S. F. Zhu , Q. L. Zhou , Acc. Chem. Res. 2012, 45, 1365; 22651217 10.1021/ar300051u

[8] a) W. Tang , X. Zhang , Chem. Rev. 2003, 103, 3029; 12914491 10.1021/cr020049i

[9] a) O. I. Afanasyev , E. Kuchuk , D. L. Usanov , D. Chusov , Chem. Rev. 2019, 119, 11857; 31633341 10.1021/acs.chemrev.9b00383

[10] a) Y. Xie , H. Pan , M. Liu , X. Xiao , Y. Shi , Chem. Soc. Rev. 2015, 44, 1740. 25645264 10.1039/c4cs00507d

[11] a) Y. Gao , G. Hong , B.‐M. Yang , Y. Zhao , Chem. Soc. Rev. 2023, 52, 5541; 37519093 10.1039/d3cs00424d

[12] a) X. Ma , C. Su , Q. Xu , Top. Curr. Chem. 2016, 374, 27; 10.1007/s41061-016-0027-1 27573267

[13] a) H. Yang , H. Yu , I. A. Stolarzewicz , W. Tang , Chem. Rev. 2023, 123, 9397; 37417731 10.1021/acs.chemrev.3c00010

[14] a) J. B. Hedges , K. S. Ryan , Chem. Rev. 2020, 120, 3161; 31869221 10.1021/acs.chemrev.9b00408

[15] H. Ishikawa , T. Yurino , R. Komastsu , M.‐Y. Gao , N. Arai , T. Touge , K. Matsumura , T. Ohkuma , Org. Lett. 2023, 25, 2355. 36961208 10.1021/acs.orglett.3c00740

[16] T. Yan , B. L. Feringa , K. Barta , ChemSusChem 2021, 14, 2303. 33961350 10.1002/cssc.202100373 PMC8252633

[17] a) W. Yang , H. Fu , Q. J. Song , M. Zhang , Y. Q. Ding , Organometallics 2011, 30, 77;

[18] a) S. Hameury , H. Bensalem , K. D. O. Vigier , Catalysts 2022, 12, 1306;

[19] a) B. Blank , M. Madalska , R. Kempe , Adv. Synth. Catal. 2008, 350, 749;

[20] a) T. A. Tillack , D. Hollmann , K. Mevius , D. Michalik , S. Bahn , M. Beller , Eur. J. Org. Chem. 2008, 2008, 4745;

[21] a) S. Elangovan , J. Neumann , J.‐B. Sortais , K. Junge , C. Darcel , M. Beller , Nat. Commun. 2016, 7, 12641; 27708259 10.1038/ncomms12641 PMC5059641

[22] a) W. Li , M. Huang , J. Liu , Y.‐L. Huang , X.‐B. Lan , Z. Ye , C. Zhao , Y. Liu , Z. Ke , ACS Catal. 2021, 11, 10377;

[23] a) S. Rösler , M. Ertl , T. Irrgang , R. Kempe , Angew. Chem., Int. Ed. 2015, 54, 15046; 10.1002/anie.201507955 26474443

[24] a) G.‐m. Zhao , H.‐l. Liu , D.‐d. Zhang , X.‐r. Huang , X. Yang , ACS Catal. 2014, 4, 2231;

[25] a) M. Bala , P. K. Verma , U. Sharma , N. Kumar , B. Singh , Green Chem. 2013, 15, 1687; 10.1002/chem.201003621 21500293

[26] a) A. Eka Putra , Y. Oe , T. Ohta , Eur. J. Org. Chem. 2013, 2013, 6146;

[27] a) M. Zhang , S. Imm , S. Gahn , H. Neumann , M. Beller , Angew. Chem., Int. Ed. 2011, 50, 11197; 10.1002/anie.201104309 21987500

[28] a) S. Khan , J. Zhang , A. Khan , Org. Lett. 2024, 26, 2728; 10.1021/acs.orglett.3c01641 37515783

[29] T. Singh , V. Atreya , S. Jalwal , A. Anand , S. Chakraborty , Chem. Asian J. 2023, 18, e202300758. 37815164 10.1002/asia.202300758

[30] a) R. Mamidala , C. Kommuri , J. Paulose , H. Aswath , L. Pawar , A. Arunachalampillai , A. H. Chermey , J. S. Tedrow , A. R. Rotheli , A. Ortiz , Org. Process Res. Dev. 2022, 26, 165;

[31] a) C.‐Y. Chang , T.‐K. Yang , Tetrahedron: Asymmetry 2003, 14, 2239;

[32] A. L. Deaguero , A. S. Bommarius , Biotechnol. Bioeng. 2014, 111, 1054. 24258338 10.1002/bit.25143

[33] N. Garg , I. Agrawal , D. Satav , D. V. Kumar , B. Sundararaju , Tetrahedron Chem. 2023, 8, 100054.

[34] Detailed optimization study, and experimental conditions are given in supporting information data.

[35] A. S. Kozlov , O. I. Afanasyev , D. Chusov , J. Catal. 2022, 413, 1070.

[36] a) Y. Zhang , C.‐S. Lim , D. S. B. Sim , H.‐J. Pan , Y. Zhao , Angew. Chem., Int. Ed. 2014, 53, 1399; 10.1002/anie.201307789 24459057

[37] a) T. Ben Halima , J. Masson‐Makdissi , S. G. Newman , Angew. Chem. Int. Ed. 2018, 57, 12925; 10.1002/anie.201808560 30113123

[38] a) S. Tresch , R. Niggeweg , K. Grossmann , Pest Manage. Sci. 2008, 64, 1195; 10.1002/ps.1618 18551723

[39] a) D. Guillaneux , S.‐H. Zhao , O. Samuel , D. Rainford , H. Kagan , J. Am. Chem. Soc. 1994, 116, 9430;

