# Supplementary material for: Molybdenum‐Catalyzed Asymmetric Amination of α‐Hydroxy Esters: Synthesis of α‐Amino Acids
Source: Adv Sci (Weinh). 2025 Mar 10;12(18):2403437. doi: 10.1002/advs.202403437 (PMC12079543; doi:10.1002/advs.202403437)
Supplement: Supplementary file 1 — Supporting Information [file ADVS-12-2403437-s001.pdf]

ADVANCED SCIENCE
Open Access

Supporting Information

for *Adv. Sci.*, DOI 10.1002/advs.202403437

Molybdenum-Catalyzed Asymmetric Amination of α-Hydroxy Esters: Synthesis of α-Amino Acids

*Shahida Perveen, Tahir Rahman, Tariq Ali, Lingyun Wang, Junjie Zhang and Ajmal Khan**

Molybdenum-Catalyzed Asymmetric Amination of α-Hydroxy Esters: Synthesis of α-Amino Acids

Shahida Perveen, Tahir Rahman, Tariq Ali, Lingyun Wang, Junjie Zhang, and Ajmal Khan*

Department of Chemistry, School of Chemistry, Xi'an Key Laboratory of Sustainable Energy Materials Chemistry and MOE Key Laboratory for Nonequilibrium Synthesis and Modulation of Condensed Matter, Xi'an Jiaotong University, Xi'an 710049, P. R. China

E-mail: ajmalkhan@xjtu.edu.cn

Table of Contents

General experimental details ……………………………………………………………S2
Preparation of α-hydroxy esters……………………………………………………S4-S9
Preparation of Chiral Molybdenum Complexes ……………………………………S10-S15
Optimization study for the amination of α-hydroxy esters……………………… S16-S18
Synthesis and characterization of products (**3-46**) ……………………………… S19-S39
General procedure for the synthesis of Benazepril *tert*-Butyl Ester (**47**) ……………… S39-S40
Optimization study for the asymmetric amination of α-hydroxy esters ……………… S41-S43
Synthesis and characterization of products (**48-71**) ……………………………… S44-S55
Synthetic applications ……………………………………………………………S55-S57
Procedures for Mechanistic Experiments……………………………………………S58-S59
Procedures for nonlinear effect (NLE) studies………………………………………S59-S62
X-ray data of compound **53** and **Mo-6** complex………………………………………S62-S66
References …………………………………………………………………… S66-S67
NMR charts ……………………………………………………………………S68-S166
HPLC charts ……………………………………………………………………S167-S193

General experimental details

Analytical thin-layer chromatography (TLC) was carried out using 0.2 mm commercial silica gel plates (Yantai Jiangyou Silica Gel Development Co., Ltd., silica gel HSGF 254). Preparative column chromatography employing silica gel (Qingdao Shenghai Fine Silica Gel Chemical Co., Ltd., 200-300 mesh) was performed according to the method of Still. Solvents for the chromatography are listed as volume/volume ratios. High-resolution mass spectra (HRMS) were performed at Instrumental Analysis Center of Xi'an Jiao Tong University using ESI method. Proton nuclear magnetic resonance (^{1}H NMR) spectra were recorded with a Varian Mercuryplus 400 (400 MHz) spectrometer. Chemical shifts are reported in delta (δ) units, parts per million (ppm) downfield from tetramethylsilane or ppm relative to the center of the singlet at 7.26 ppm for deuteriochloroform. Coupling constants are reported in Hertz (Hz). Carbon-13 nuclear magnetic resonance (^{13}C NMR) spectra were recorded with a Varian Gemini 400 (100 MHz) spectrometer. Chemical shifts are reported in delta (δ) units, ppm relative to the center of the triplet at 77.0 ppm for deuteriochloroform. ^{13}C NMR spectra were routinely run with broadband decoupling. High performance liquid chromatography (HPLC) was performed with FuLi (instruments) spectrometers using chiral column as noted for each compound with hexane/2-propanol as eluent. Optical rotations were measured on SGW®-1 polarimeter.

All commercially available reagents were purchased from Energy Chemicals and Aladin/Sigma-Aldrich companies and used as received for the reactions without any purification.

α-hydroxy esters and different amine used in the current study:

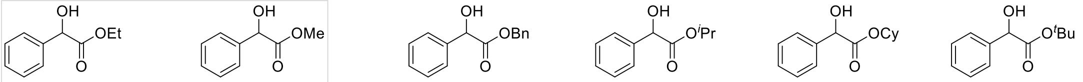

1a | 1b — commercially available

1c known compound ref. 2a | 1d known compound ref. 2b | 1e known compound ref. 2b | 1f known compound ref. 2b

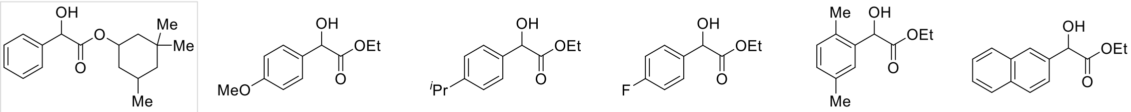

1g commercially available | 1h known compound ref. 2a | 1i known compound ref. 2d | 1j known compound ref. 2a | 1k known compound ref. 2d | 1l known compound ref. 2a

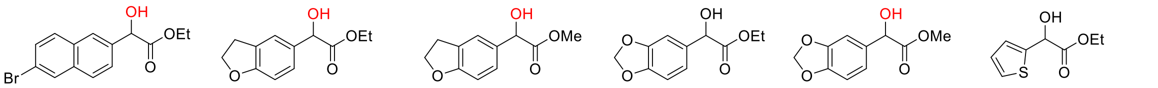

1m unknown compound | 1n unknown compound | 1o unknown compound | 1p known compound ref. 2e | 1q unknown compound | 1r known compound ref. 2a

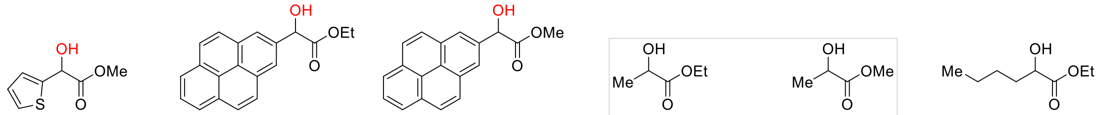

1s unknown compound | 1t unknown compound | 1u unknown compound | 1v | 1w — commercially available | 1x known compound ref. 2c

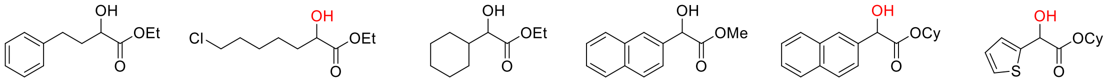

1y known compound ref. 2f | 1z unknown compound | 1za known compound ref. 2c | 1zb known compound ref. 2a | 1zc unknown compound | 1zd unknown compound

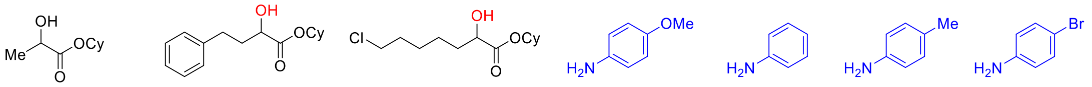

1ze known compound ref. 2g | 1zf unknown compound | 1zg unknown compound | 2a | 2b | 2c | 2d

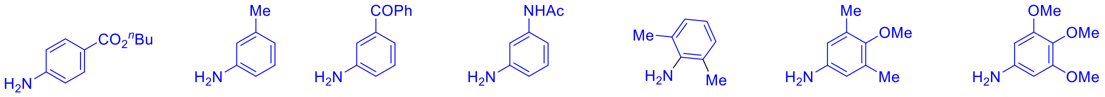

2e | 2f | 2g | 2h | 2i | 2j | 2k

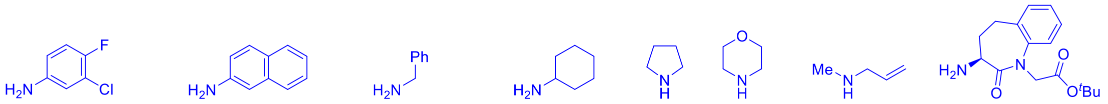

2l | 2m | 2n | 2o | 2p | 2q | 2r | 2s

Preparation of α-substituted α-hydroxy esters

Most of the α-hydroxy esters used in this study are known compounds, some are commercially available and other were synthesized according to the previous reported procedures.[1] All characterization data are in accordance with the literature.[2] Previously unknown compounds have been fully characterized.

Procedure A:

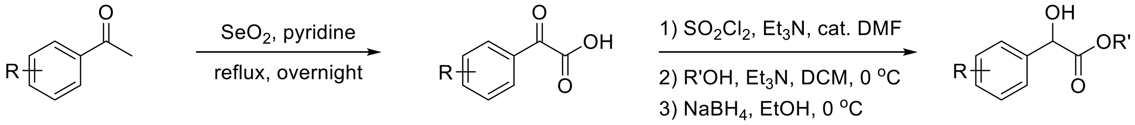

To a 100 mL flame-dried round bottom flask equipped with a stirrer bar in 20 mL of pyridine, were added the corresponding substituted ketones (2 mmol) and selenium dioxide (3 mmol). The mixture was stirred at reflux and monitored by thin layer chromatography (TLC). After completion of reaction, the dark solution was filtered and the residue was washed with EtOAc three times. The collected filtrate was then treated with concentrated HCl and extracted with EtOAc (3×20 mL). The combined organic layer was dried over anhydrous Na_2SO_4 and concentrated in vacuum. The desired α-aryl ketone acids were isolated by silica gel column chromatography.[1a]

The obtained α-aryl ketone acids (14 mmol) and triethylamine (1.95 mL, 14 mmol) were dissolved in CH_2Cl_2 (15 mL), and then thionyl chloride (2.03 mL, 28 mmol) was added dropwise at 0 °C. The mixture was stirred overnight at room temperature in the presence of a catalytic amount of DMF (two drops). Afterwards, the above prepared mixture was added dropwise over 1 h to a solution of corresponding alcohol (3.4 mL, 84 mmol) and triethylamine (5.87 mL, 42 mmol) in CH_2Cl_2 (25 mL) at 0 °C. After 1 h, saturated aqueous $NaHCO_3$ solution (20 mL) was added to quench the reaction and the mixture was extracted with CH_2Cl_2 (3×30 mL). After being dried and concentrated, the crude products were purified by column chromatography using PE/EA as eluent to give the α-keto-ester products.[1a]

The obtained α-keto-esters (11.2 mmol) were suspended in EtOH (20 mL) at –30 °C. To this mixture $NaBH_4$ (0.21 g, 5.6 mmol) was added in three small portions. After 1 hours, the reaction completion was monitored from TLC. When the reaction was complete the mixture was quenched with 10% HCl until evolution of gas stopped. For work-up the mixture was diluted with H_2O and extraction was performed with EtOAc. The organic layer was washed with sat. NaCl solution (2.50 mL) and dried over $MgSO_4$. The curd α-hydroxy ester products were purified by column chromatography.

Procedure B:

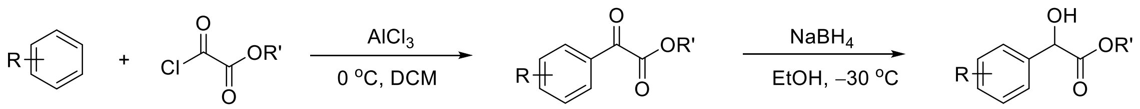

In a two-necked round-bottomed flask, $AlCl_3$ (1.129 g, 8.49 mmol) was suspended in CH_2Cl_2 (60 mL) at 0 °C under inert conditions. To this mixture ethyl oxalate chloride (0.95 mL, 8.49 mmol) was added dropwise in about 15 minutes. After few minutes, the stirred suspension became a pale-yellow solution. At 0 °C arene (4.7 mmol) was added dropwise in about 10 min. Then the solution was stirred at room temperature for 2 h. To this reaction mixture H_2O was carefully added and extraction was performed with EtOAc. The organic layer was collected and washed with sat. NaCl solution, dried over Na_2SO_4 and concentrated in vacuo. The crude product was purified by column chromatography using PE/EA as eluent.[1b]

The obtained α-keto-esters (11.2 mmol) were suspended in EtOH (20 mL) at –30 °C. To this mixture $NaBH_4$ (0.21 g, 5.6 mmol) was added in three small portions. After 1 hours, the reaction completion was monitored from TLC. When the reaction was complete the mixture was quenched with 10% HCl until evolution of gas stopped. For work-up the mixture was diluted with H_2O and extraction was performed with EtOAc. The organic layer was washed with sat. NaCl solution and dried over $MgSO_4$. The curd α-hydroxy ester products were purified by column chromatography.

Procedure C:

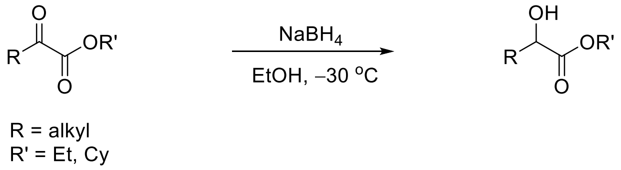

Aliphatic α-keto-esters (11.2 mmol) were suspended in EtOH (20 mL) at –30 °C. To this mixture $NaBH_4$ (0.21 g, 5.6 mmol) was added in three small portions. After 1 hours, the reaction completion was monitored from TLC. When the reaction was complete the mixture was quenched with 10% HCl until evolution of gas stopped. For work-up the mixture was diluted with H_2O and extraction was performed with EtOAc. The organic layer was washed with sat. NaCl solution and dried over $MgSO_4$. The curd α-hydroxy ester products were purified by column chromatography.

Characterization of unknown α-hydroxy esters

ethyl 2-(6-bromonaphthalen-2-yl)-2-hydroxyacetate (1m)

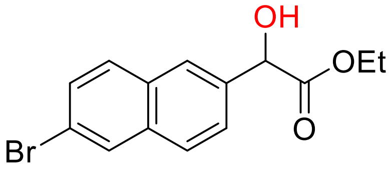

1m

Prepared through **procedure B** and was obtained as a white solid; **^{1}H NMR** (400 MHz, $CDCl_3$) δ 8.34 (s, 1H), 7.79 (d, *J* = 8.3 Hz, 1H), 7.72 (d, *J* = 8.8 Hz, 1H), 7.58–7.53 (m, 2H), 7.47–7.43 (m, 1H), 5.73 (s, 1H), 4.31–4.12 (m, 2H), 3.66 (bs, 1H), 1.16 (t, *J* = 7.3 Hz, 3H); **^{13}C NMR** (100 MHz, $CDCl_3$) δ 173.7, 133.4, 132.3, 132.1, 130.3, 129.2, 129.1, 126.4, 126.3, 125.6, 120.8, 70.9, 62.4, 13.9; **HRMS (ESI)** m/z: calcd. for $C_{14}H_{13}BrNaO_3$ $[M+Na]^+$ 330.9940; Found 330.9936.

ethyl 2-(2,3-dihydrobenzofuran-5-yl)-2-hydroxyacetate (1n)

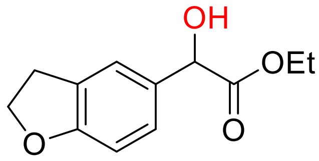

1n

Prepared through **procedure B** and was obtained as a white solid; **^{1}H NMR** (400 MHz, $CDCl_3$) δ 7.23 (s, 1H), 7.12 (d, *J* = 8.2 Hz, 1H), 6.73 (d, *J* = 8.1 Hz, 1H), 5.07 (s, 1H), 4.55 (t, *J* = 8.6 Hz, 2H), 4.30–4.10 (m, 2H), 3.56 (s, 1H), 3.18 (t, *J* = 8.6 Hz, 2H), 1.22 (t, *J* = 7.2 Hz, 3H); **^{13}C NMR** (100 MHz, $CDCl_3$) δ 173.8, 160.2, 130.6, 127.4, 126.7, 123.1, 109.1, 72.7, 71.3, 61.9, 29.5, 13.9; **HRMS (ESI)** m/z: calcd. for $C_{12}H_{14}NaO_4$ $[M+Na]^+$ 245.0784; Found 245.0784.

methyl 2-(2,3-dihydrobenzofuran-5-yl)-2-hydroxyacetate (1o)

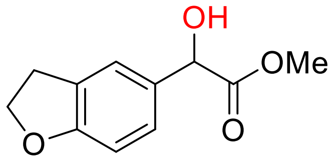

1o

Prepared through **procedure B** and was obtained as a white solid; **^{1}H NMR** (400 MHz, $CDCl_3$) δ 7.25 (s, 1H), 7.15 (d, *J* = 8.3 Hz, 1H), 6.77 (d, *J* = 8.2 Hz, 1H), 5.11 (s, 1H), 4.59 (t, *J* = 8.6 Hz, 2H), 3.78 (s, 1H), 3.43 (bs, 1H), 3.22 (t, *J* = 8.6 Hz, 2H); **^{13}C NMR** (100 MHz, $CDCl_3$)

δ 174.4, 160.4, 130.4, 127.6, 126.9, 123.2, 109.2, 72.7, 71.4, 52.9, 29.5; **HRMS (ESI)** m/z: calcd. for $C_{11}H_{12}NaO_4$ $[M+Na]^+$ 231.0628; Found 231.0626.

methyl 2-(benzo[d][1,3]dioxol-5-yl)-2-hydroxyacetate (1q)

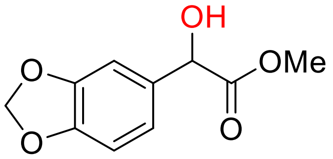

1q

Prepared through **procedure B** and was obtained as a white solid; **^{1}H NMR** (400 MHz, $CDCl_3$) δ 6.90–6.87 (m, 2H), 6.81–6.78 (m, 1H), 5.96 (s, 2H), 5.07 (s, 1H), 3.77 (s, 3H), 3.38 (bs, 1H); **^{13}C NMR** (100 MHz, $CDCl_3$) δ 174.1, 147.9, 147.8, 132.1, 120.4, 108.3, 107.0, 101.2, 72.7, 53.0; **HRMS (ESI)** m/z: calcd. for $C_{10}H_{10}NaO_5$ $[M+Na]^+$ 233.0420; Found 233.0423.

methyl 2-hydroxy-2-(thiophen-2-yl)acetate (1s)

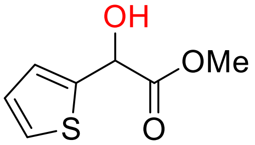

1s

Prepared through **procedure A** and was obtained as a white solid; **^{1}H NMR** (400 MHz, $CDCl_3$) δ 7.26–7.24 (m, 1H), 7.07 (s, 1H), 6.97–6.94 (m, 1H), 5.41 (s, 1H), 3.94 (bs, 1H), 3.76 (s, 3H); **^{13}C NMR** (100 MHz, $CDCl_3$) δ 172.9, 141.3, 127.0, 125.8, 125.5, 69.1, 53.1; **HRMS (ESI)** m/z: calcd. for $C_7H_8NaO_3S$ $[M+Na]^+$ 195.0086; Found 195.0084.

ethyl 2-hydroxy-2-(pyren-2-yl)acetate (1t)

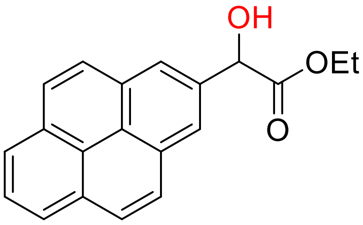

1t

Prepared through **procedure B** and was obtained as a white solid; **^{1}H NMR** (400 MHz, $CDCl_3$) δ 8.42 (d, *J* = 9.2 Hz, 1H), 8.21–8.00 (m, 8H), 6.15 (s, 1H), 4.32–4.12 (m, 2H), 4.01 (bs, 1H), 1.13 (t, *J* = 7.2 Hz, 3H); **^{13}C NMR** (100 MHz, $CDCl_3$) δ 174.3, 131.5, 131.2, 130.6, 128.9, 128.1, 127.8, 127.3, 126.1, 125.5, 125.3, 125.0, 124.9, 124.6, 122.9, 71.0, 62.4, 14.0; **HRMS (ESI)** m/z: calcd. for $C_{20}H_{16}NaO_3$ $[M+Na]^+$ 327.0992; Found 327.0986.

methyl 2-hydroxy-2-(pyren-2-yl)acetate (1u)

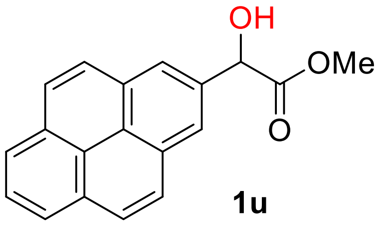

1u

Prepared through **procedure B** and was obtained as a white solid; **^{1}H NMR** (400 MHz, $CDCl_3$) δ 8.39 (d, *J* = 9.2 Hz, 1H), 8.20–8.13 (m, 4H), 8.09–7.99 (m, 4H), 6.14 (s, 1H), 3.75 (bs, 1H), 3.71 (s, 3H); **^{13}C NMR** (100 MHz, $CDCl_3$) δ 174.6, 131.6, 131.3, 131.3, 130.6, 128.9, 128.3, 127.9, 127.3, 126.1, 125.6, 125.4, 125.1, 125.0, 124.9, 124.6, 122.8, 71.0, 53.2; **HRMS (ESI)** m/z: calcd. for $C_{19}H_{14}NaO_3$ $[M+Na]^+$ 313.0835; Found 313.0835.

ethyl 7-chloro-2-hydroxyheptanoate (1z)

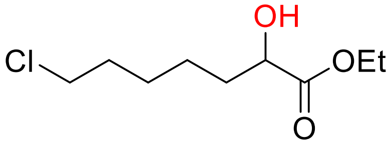

1z

Prepared through **procedure C** and was obtained as a white solid; **^{1}H NMR** (400 MHz, $CDCl_3$) δ 4.27–4.22 (m, 2H), 4.20–4.15 (m, 1H), 3.54 (t, *J* = 5.8 Hz, 2H), 2.91 (s, 1H), 1.82–1.75 (m, 3H), 1.68–1.60 (m, 1H), 1.49–1.40 (m, 4H), 1.30 (t, *J* = 7.2 Hz, 3H); **^{13}C NMR** (100 MHz, $CDCl_3$) δ 175.1, 70.1, 61.5, 44.8, 34.0, 32.3, 26.4, 24.0, 14.1; **HRMS (ESI)** m/z: calcd. for $C_9H_{17}ClNaO_3$ $[M+Na]^+$ 231.0758; Found 231.0756.

cyclohexyl 2-hydroxy-2-(naphthalen-2-yl)acetate (1zc)

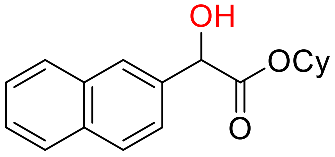

1zc

Prepared through **procedure A** and was obtained as a white solid; **^{1}H NMR** (400 MHz, $CDCl_3$) δ 8.19 (d, *J* = 8.3 Hz, 1H), 7.90–7.78 (m, 2H), 7.52–7.40 (m, 4H), 5.79 (s, 1H), 4.88–4.80 (m, 1H), 3.78 (s, 1H), 1.55–1.09 (m, 10H); **^{13}C NMR** (100 MHz, $CDCl_3$) δ 173.5, 134.4, 133.8, 130.9, 129.0, 128.5, 126.1, 125.6, 125.3, 125.0, 123.8, 74.5, 71.1, 31.0, 30.7, 24.9, 23.0, 22.7; **HRMS (ESI)** m/z: calcd. for $C_{18}H_{20}NaO_3$ $[M+Na]^+$ 307.1305; Found 307.1301.

cyclohexyl 2-hydroxy-2-(thiophen-2-yl)acetate (1zd)

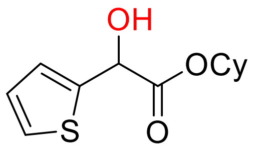

1zd

Prepared through **procedure A** and was obtained as a white solid; **^{1}H NMR** (400 MHz, $CDCl_3$) δ 7.26 (s, 1H), 7.10 (s, 1H), 6.99–6.96 (m, 1H), 5.38 (d, *J* = 6.5 Hz, 1H), 4.92–4.86 (m, 1H), 3.65 (d, *J* = 6.2 Hz, 1H), 1.90–1.85 (m, 1H), 1.78–1.70 (m, 2H), 1.63–1.48 (m, 3H), 1.42–1.25 (m, 5H); **^{13}C NMR** (100 MHz, $CDCl_3$) δ 171.9, 141.7, 126.8, 125.4, 125.0, 75.1, 69.1, 31.3, 31.0, 25.1, 23.3, 23.2; **HRMS (ESI)** m/z: calcd. for $C_{12}H_{16}NaO_3S$ $[M+Na]^+$ 263.0712; Found 263.0714.

cyclohexyl 2-hydroxy-4-phenylbutanoate (1zf)

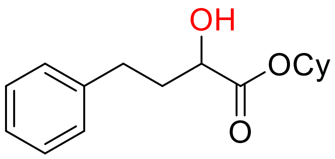

1zf

Prepared through **procedure C** and was obtained as a white solid; **^{1}H NMR** (400 MHz, $CDCl_3$) δ 7.30–7.26 (m, 2H), 7.21–7.17 (m, 3H), 4.89–4.15 (m, 1H), 4.20–4.15 (m, 1H), 2.98 (s, 1H), 2.83–2.67 (m, 2H), 2.15–2.06 (m, 1H), 1.98–1.91 (m, 1H), 1.87–1.83 (m, 2H), 1.74–1.72 (m, 2H), 1.50–1.26 (m, 6H); **^{13}C NMR** (100 MHz, $CDCl_3$) δ 174.6, 141.3, 128.5, 128.4, 125.9, 74.3, 69.7, 36.1, 31.5, 31.4, 31.0, 25.2, 23.5; **HRMS (ESI)** m/z: calcd. for $C_{16}H_{22}NaO_3$ $[M+Na]^+$ 285.1461; Found 285.1465.

ethyl 7-chloro-2-hydroxyheptanoate (1zg)

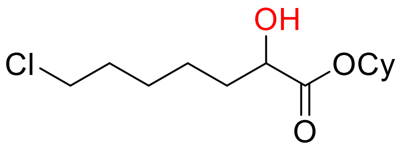

1zg

Prepared through **procedure C** and was obtained as a white solid; **^{1}H NMR** (400 MHz, $CDCl_3$) δ 4.86 (s, 1H), 4.17–4.13 (m, 1H), 3.53 (t, *J* = 5.8 Hz, 2H), 2.91 (d, *J* = 5.4 Hz, 1H), 1.87–1.64 (m, 8H), 1.57–1.33 (m, 10H); **^{13}C NMR** (100 MHz, $CDCl_3$) δ 174.6, 74.1, 70.1, 44.8, 34.1, 32.3, 31.4, 31.3, 26.5, 25.1, 23.9, 23.5; **HRMS (ESI)** m/z: calcd. for $C_{13}H_{13}ClNaO_3$ $[M+Na]^+$ 285.1228; Found 285.1232.

Preparation of chiral Mo-complexes

Synthesis of Mo-1 complex:

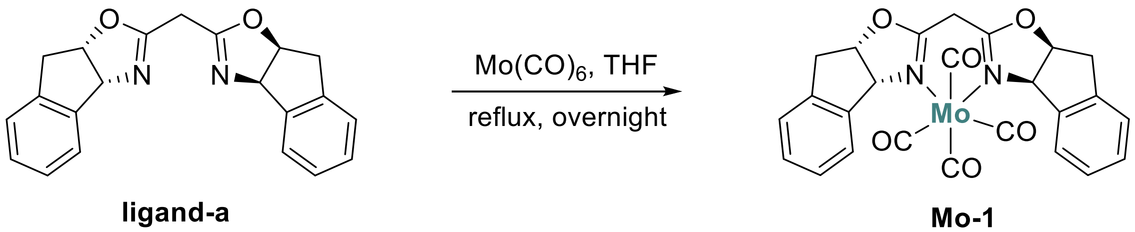

A 100 mL Schleck round bottom flask was charged with $Mo(CO)_6$ (1.0 mmol, 264.0 mg), 20 mL THF and a solution of **ligand-a** (1.0 mmol, 330.4 mg) in 5 mL THF under nitrogen atmosphere. The mixture was stirred at 60 ˚C for overnight. The solvent was removed in vacuo. The resulting mixture was washed with hexane and filtered through Gooch filter. The solid obtained was washed with 20 mL of hexane and dried in vacuo to afford 301.5 mg (56% yields) of the title compound as a light-red solid. **^{1}H NMR** (400 MHz, $CDCl_3$) δ 8.17 (d, *J* = 7.4 Hz, 2H), 7.35–7.27 (m, 4H), 7.22–7.17 (m, 2H), 5.55 (d, *J* = 7.5 Hz, 2H), 5.37–5.33 (m, 2H), 3.36–3.23 (m, 6H); **^{13}C NMR** (100 MHz, $CDCl_3$) δ 205.7, 163.5, 139.2, 138.8, 129.6, 127.8, 127.4, 124.9, 85.1, 80.4, 38.2, 29.6; **HRMS (ESI)** m/z: calcd. for $C_{25}H_{19}MoN_2O_6$ $[M + H]^+$ 541.0292; Found 541.0286.

Synthesis of Mo-2 complex:

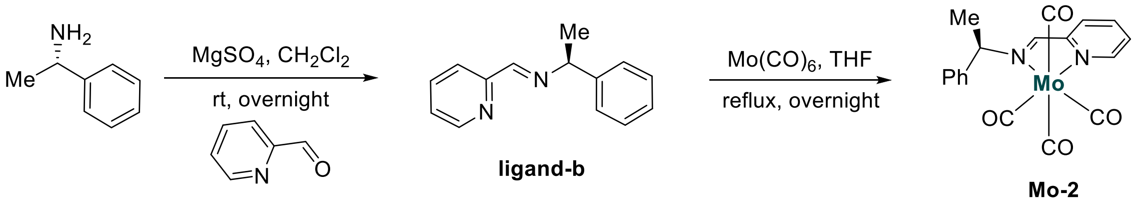

First **ligand-b** was synthesized according to the following modified procedures.[3a] To a two necked round bottom flask equipped with a magnetic stir bar, $MgSO_4$ (674.2 mg, 5.6 mmol) in CH_2Cl_2 (40 mL) was added (*S*)-2-methyl benzylamine (3.39 g, 28.0 mmol). After stirring for 10 minutes, 2-pyridine carboxyaldehyde (3.0 g, 28.0 mmol) was added and the reaction mixture was stirred at room temperature and monitored by TLC. After the reaction is completed, the crude mixture was filtered and the solvent was removed under reduced pressure to give the desired product (**ligang-b**) as yellow-brown oil in 92% yield (5.4 g). **^{1}H NMR** (400 MHz, $CDCl_3$) δ 8.60 (d, *J* = 4.6 Hz, 1H), 8.47 (s, 1H), 8.08 (d, *J* = 7.8 Hz, 1H), 7.67 (t, *J* = 7.6 Hz, 1H), 7.43 (d, *J* = 7.6 Hz, 2H), 7.33 (t, *J* = 7.6 Hz, 2H), 7.25–7.21 (m, 2H), 4.63 (q, *J* = 6.6 Hz, 1H), 1.60

(d, J = 6.6 Hz, 3H); ^{13}**C NMR** (100 MHz, $CDCl_3$) δ 160.2, 154.5, 149.1, 144.3, 136.3, 128.3, 126.8, 126.5, 124.5, 121.2, 69.4, 24.4; All other data are in accordance with literature.[3a]

A 100 mL Schleck round bottom flask was charged with $Mo(CO)_6$ (4.8 mmol, 1.27 g), 50 mL THF and a solution of **Ligand-b** (4.0 mmol, 0.842 g) in 5 mL THF under nitrogen atmosphere. The mixture was stirred at 60 ˚C for overnight. The solvent was removed in vacuo. The resulting mixture was washed with hexane and filtered through Gooch filter. The cake was washed with more hexane and dried in vacuo to afford 1.46 g (87% yield) of the title compound as a brown crystalline solid. ^{1}**H NMR** (400 MHz, $CDCl_3$) δ 9.11 (d, J = 5.4 Hz, 1H), 8.53 (s, 1H), 7.91–7.86 (m, 1H), 7.72 (d, J = 7.6 Hz, 1H), 7.42–7.34 (m, 6H), 4.32 (q, J = 6.7 Hz, 1H), 1.97 (d, J = 6.8 Hz, 3H); ^{13}**C NMR** (100 MHz, $CDCl_3$) δ 203.2, 159.7, 153.9, 153.1, 139.6, 136.7, 128.8, 128.5, 127.8, 127.4, 125.9, 71.7, 21.2; **HRMS (ESI)** m/z: calcd. for $C_{18}H_{15}MoN_2O_4$ $[M + H]^+$ 421.0080; Found 421.0073.

Synthesis of Mo-3 complex:

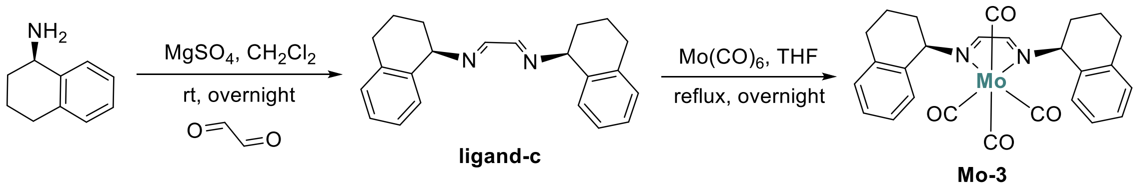

A mixture of glyoxal (0.79 mL, 6.89 mmol, 40% aqueous solution), α-chiral amine (2.03 g, 13.78 mmol), formic acid (52.0 µmL, 1.38 mmol) and $MgSO_4$ (1.66 g, 13.78 mmol) was stirred in CH_2Cl_2 (50 mL) at 20 ˚C. After completion of reaction, the mixture was filtered over Celite and concentrated via rotary evaporator. The residue was dissolved in cyclohexane, dried over Na_2SO_4 and the solvent was evaporated to give the product (**ligand-c**)[3b] as a pale-yellow oil; yield: 1.65 g (76%); ^{1}**H NMR** (400 MHz, $CDCl_3$) δ 8.08 (s, 1H), 7.19–7.11 (m, 3H), 6.97 (d, J = 7.2 Hz, 1H), 4.53 (q, J = 5.2 Hz, 1H), 2.95–2.77 (m, 2H), 2.08–1.80 (m, 4H); ^{13}**C NMR** (100 MHz, $CDCl_3$) δ 161.4, 137.2, 135.8, 129.4, 129.0, 127.3, 126.0, 68.0, 31.1, 29.3, 19.5; All other data are in accordance with literature.[3b]

A 100 mL Schleck round bottom flask was charged with $Mo(CO)_6$ (2.0 mmol, 528.04 mg), 20 mL THF and a solution of **ligand-c** (2.0 mmol, 632.9 mg) in 5 mL THF under nitrogen atmosphere. The mixture was stirred at 60 ˚C for overnight. The solvent was removed in vacuo. The resulting mixture was washed with hexane and filtered through Gooch filter. The solid obtained was washed with 20 mL of hexane and dried in vacuo to afford 0.86 g (82% yields) of the title compound as a dark-brown solid. ^{1}**H NMR** (400 MHz, $CDCl_3$) δ 8.10 (s, 1H), 7.29–7.19 (m, 3H), 6.95 (d, J = 7.6 Hz, 1H), 5.38 (q, J = 6.8 Hz, 1H), 3.07–2.99 (m, 1H), 2.90–2.83 (m,

1H), 2.67–2.59 (m, 1H), 2.35–2.27 (m, 1H), 1.92–1.85 (m, 2H); **^{13}C NMR** (100 MHz, $CDCl_3$) δ 203.0, 158.2, 138.1, 134.7, 129.3, 128.1, 126.3, 72.3, 32.5, 29.3, 19.6; **HRMS (ESI)** m/z: calcd. for $C_{26}H_{25}MoN_2O_4$ [M + H]$^+$ 527.0863; Found 527.0857.

Synthesis of Mo-4 complex:

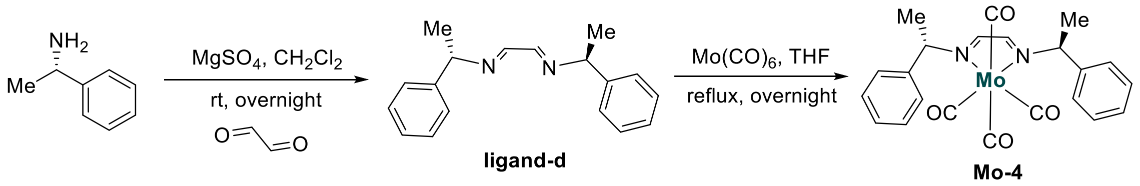

A mixture of glyoxal (2.4 mL, 16.12 mmol, 40% aqueous solution), α-(*S*)-methylbenzylamine (4.0 g, 33.02 mmol), formic acid (0.11 mL, 2.8 mmol) and $MgSO_4$ (8.2 g) was stirred in CH_2Cl_2 (40 mL) for 30 min at 20 °C. After completion of reaction, the mixture was filtered over Celite and concentrated via rotary evaporator. The residue was dissolved in cyclohexane, dried over Na_2SO_4 and the solvent was evaporated to give the product (**ligand-d**)[3c] as a pale-yellow oil; yield: 3.8 g (89%); **^{1}H NMR** (400 MHz, $CDCl_3$) δ 8.05 (s, 2H), 7.37–7.30 (m, 8H), 7.28–7.21 (m, 2H), 4.51 (q, J = 6.6 Hz, 2H), 1.58 (d, J = 6.6 Hz, 6H); **^{13}C NMR** (100 MHz, $CDCl_3$) δ 160.7, 143.6, 128.6, 127.2, 126.6, 69.7, 23.9; All other data are in accordance with literature.[3c]

A 100 mL Schleck round bottom flask was charged with $Mo(CO)_6$ (2.4 mmol, 634 mg), 30 mL THF and a solution of **ligand-d** (2.0 mmol, 528 mg) in 5 mL THF under nitrogen atmosphere. The mixture was stirred at 60 °C for overnight. The solvent was removed in vacuo. The resulting mixture was washed with hexane and filtered through Gooch filter. The solid obtained was washed with 20 mL of hexane and dried in vacuo to afford 0.858 g (91% yields) of the title compound as a dark-brown solid. **^{1}H NMR** (400 MHz, $CDCl_3$) δ 8.27 (s, 2H), 7.40-7.30 (m, 10H), 5.36 (q, J = 6.6 Hz, 2H), 1.90 (d, J = 6.7 Hz, 6H); **^{13}C NMR** (100 MHz, $CDCl_3$) δ 201.0, 156.4, 140.0, 128.7, 128.3, 127.7, 72.0, 21.5; **HRMS (ESI)** m/z: calcd. for $C_{22}H_{21}MoN_2O_4$ [M + H]$^+$ 475.0550; Found 475.0543.

Synthesis of Mo-5 complex:

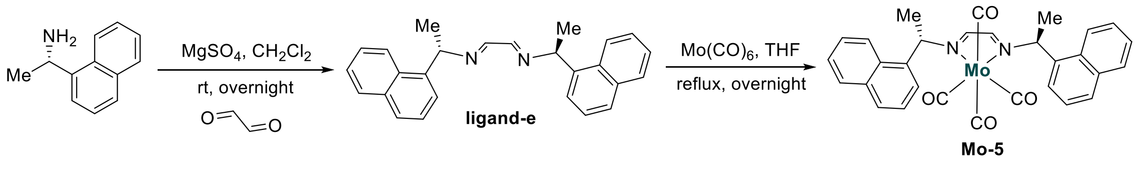

A mixture of glyoxal (0.57 mL, 5.0 mmol, 40% aqueous solution), α-chiral amine (1.71 g, 10.0 mmol), formic acid (46 μmL, 1.0 mmol) and $MgSO_4$ (1.2 g, 10.0 mmol) was stirred in CH_2Cl_2 (30 mL) at 20 ˚C. After completion of reaction, the mixture was filtered over Celite and concentrated via rotary evaporator. The residue was dissolved in cyclohexane, dried over Na_2SO_4 and the solvent was evaporated to give the product (**ligand-e**)[3b] as a pale-yellow oil; yield: 1.4 g (72%); **^{1}H NMR** (400 MHz, $CDCl_3$) δ 8.13 (s, 1H), 8.11 (d, *J* = 8.4 Hz, 1H), 7.85 (d, *J* = 8.2 Hz, 1H), 7.75 (d, *J* = 8.2 Hz, 1H), 7.64 (d, *J* = 7.2 Hz, 1H), 7.52–7.42 (m, 3H), 5.38 (q, *J* = 7.2 Hz, 1H), 1.71 (d, *J* = 6.0 Hz, 3H); **^{13}C NMR** (100 MHz, $CDCl_3$) δ 161.2, 139.4, 134.0, 130.6, 129.0, 127.8, 126.0, 125.6, 125.4, 124.0, 123.2, 64.8, 23.6; All other data are in accordance with literature.[3b]

A 100 mL Schleck round bottom flask was charged with $Mo(CO)_6$ (2.0 mmol, 528.04 mg), 30 mL THF and a solution of **ligand-e** (2.0 mmol, 729.0 mg) in 10 mL THF under nitrogen atmosphere. The mixture was stirred at 60 ˚C for overnight. The solvent was removed in vacuo. The resulting mixture was washed with hexane and filtered through Gooch filter. The solid obtained was washed with 20 mL of hexane and dried in vacuo to afford 0.93 g (82% yields) of the title compound as a dark-brown solid. **^{1}H NMR** (400 MHz, $CDCl_3$) δ 7.88–7.84 (m, 1H), 7.74–7.69 (m, 2H), 7.41–7.32 (m, 5H), 6.07 (q, *J* = 7.2 Hz, 1H), 1.89 (d, *J* = 6.2 Hz, 3H); **^{13}C NMR** (100 MHz, $CDCl_3$) δ 202.8, 157.3, 135.2, 134.1, 131.2, 129.5, 129.0, 127.0, 126.2, 125.2, 125.0, 123.9, 66.8, 22.2; **HRMS (ESI)** m/z: calcd. for $C_{30}H_{25}MoN_2O_4$ $[M + H]^+$ 575.0863; Found 575.0867.

Synthesis of Mo-6 complex:

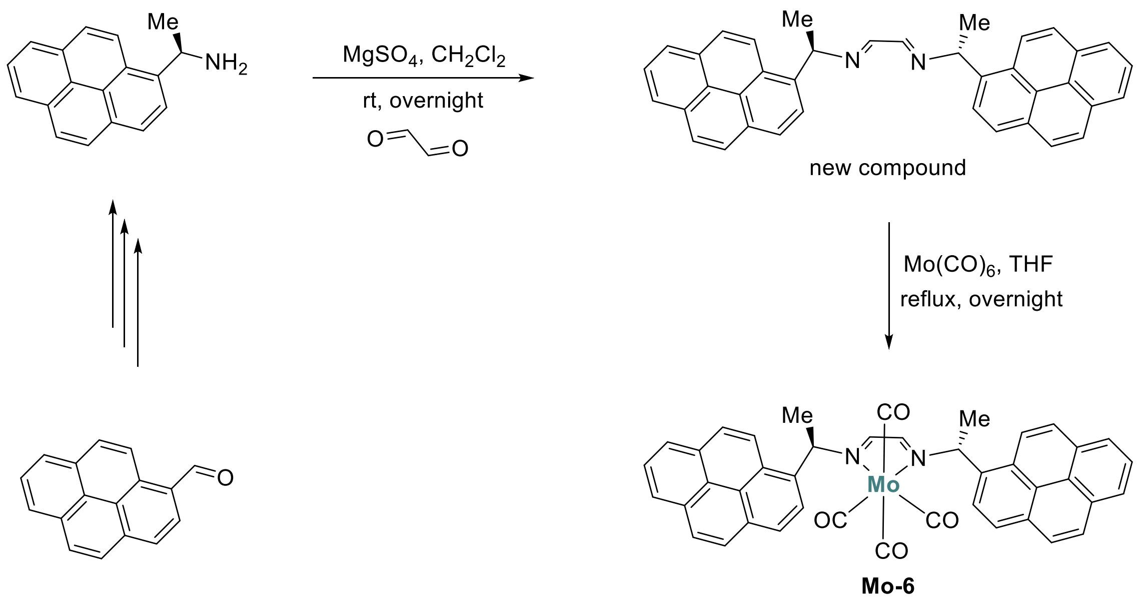

Step 1: To a flask containing a solution of the (*R*)-sulfonamide (10.42 mmol, 1.2 equiv.) and the 1-Pyrenecarboxaldehyde (8.69 mmol, 1.0 equiv.) was added Ti(OiPr)$_4$ (13.03 mmol, 1.5 equiv.) in THF and the reaction mixture was heated at 50 °C for overnight. Upon reaction completion,

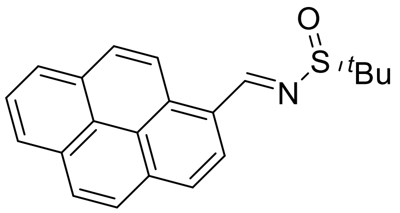

the reaction mixture was allowed to cool to room temperature, diluted with EtOAc, silica gel was added, and poured into brine with rapid stirring. The resulting suspension was filtered through a plug of Celite and the filter cake washed with EtOAc. The filtrate was transferred to a separatory funnel, and the organic layer was washed with an equal volume of brine. The organic phase was dried (MgSO$_4$), filtered, concentrated, and purified by flash chromatography to obtained 2.49 g (86%) of the title compound. **^{1}H NMR** (400 MHz, CDCl$_3$) δ 9.55 (s, 1H), 9.02–8.99 (m, 1H), 8.60–8.57 (m, 1H), 8.24–8.13 (m, 5H), 8.06–8.03 (m, 2H), 1.37 (s, 9H); **^{13}C NMR** (100 MHz, CDCl$_3$) δ 161.1, 134.2, 131.0, 130.8, 130.4, 129.7, 129.6, 127.6, 127.2, 126.5, 126.4, 126.3, 126.0, 124.9, 124.8, 124.3, 122.4, 57.9, 22.7; **HRMS (ESI)** m/z: calcd. for $C_{21}H_{20}NOS$ [M+H]$^+$ 334.1260; Found 334.1258.

Step 2: To a stirred solution of imine from **step 1** (2.0 g, 6.00 mmol) in dry toluene was added

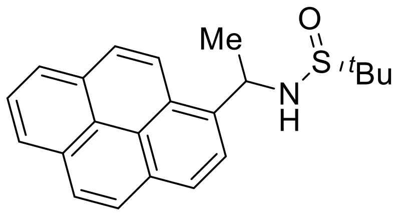

MeMgBr (15.0 mmol, 1.0 mol/L in THF) dropwise at -78 ºC under nitrogen environment. The mixture was allowed to stir at -78 ºC for 1 h and then warmed to room temperature. The reaction was quenched with aqueous solution of saturated NH$_4$Cl and extracted with EtOAc (20 mL×3). The combined organic phases were dried over Na$_2$SO$_4$, filtered, and concentrated in vacuo. The crude mixture was purified by column chromatography (silica gel) to give the title compound (1.51 g, 72 %). **^{1}H NMR** (400 MHz, CDCl$_3$) δ 8.48–8.40 (m, 1H), 8.19–8.11 (m, 5H), 8.02–7.98 (m, 3H), 5.74–5.68 (m, 1H), 3.58 (bs, 1H), 1.83 (d, *J* = 6.4 Hz, 3H), 1.21 (s, 9H); **^{13}C NMR** (100 MHz, CDCl$_3$) δ 136.5, 131.4, 130.7, 128.3, 127.8, 127.4, 126.0, 125.3, 125.1, 125.0, 124.9, 124.2, 122.6, 55.8, 51.2, 25.0, 22.6; **HRMS (ESI)** m/z: calcd. for $C_{22}H_{24}NOS$ [M+H]$^+$ 350.1573; Found 350.1569.

Step 3: To a stirred solution of the addition product (**step 2**) in dry CH$_2$Cl$_2$ was added HCl (2 M

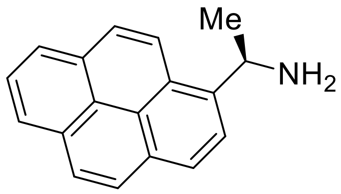

in CH$_2$Cl$_2$, 4 equiv.) dropwise at 24 ºC (a white precipitate was formed upon addition.). The mixture was allowed to stir at 24 ºC for 1 h. The white precipitate was then collected by filtration, suspended in CH$_2$Cl$_2$, and basified with NaOH (aq, 10% w/w).

The organic layer was dried over Na_2SO_4, filtered and concentrated to give desired chiral amine which was used for preparation of molybdenum complex without further purification. **^{1}H NMR** (400 MHz, $CDCl_3$) δ 8.37 (d, *J* = 9.2 Hz, 1H), 8.23–8.10 (m, 5H), 8.03–7.97 (m, 3H), 5.31–5.27 (m, 1H), 1.89 (bs, 2H), 1.66 (d, *J* = 6.4 Hz, 3H); **^{13}C NMR** (100 MHz, $CDCl_3$) δ 141.0, 131.4, 130.7, 130.1, 127.5, 127.4, 126.9, 125.8, 125.2, 125.1, 125.0, 124.9, 124.8, 122.3, 122.1, 46.7, 25.4; **HRMS (ESI)** m/z: calcd. for $C_{18}H_{16}N$ $[M+H]^+$ 246.1277; Found 246.1279.

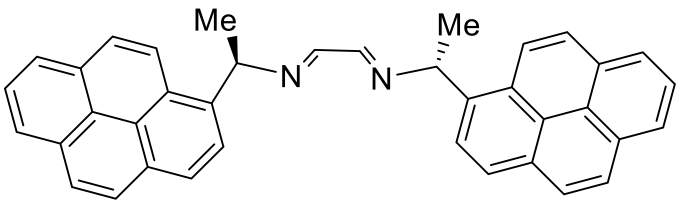

A mixture of glyoxal (0.457 mL, 4.0 mmol, 40% aqueous solution), α-chiral amine (1.96 g, 8 mmol), formic acid (30 μmL, 0.8 mmol) and $MgSO_4$ (0.963 g, 8 mmol) was stirred in CH_2Cl_2 (60 mL) at 20 ˚C. After completion of reaction, the mixture was filtered over Celite and concentrated via rotary evaporator. The residue was dissolved in cyclohexane, dried over Na_2SO_4 and the solvent was evaporated to give the product (**ligand-f**) as a brown solid; yield: 1.27 g (62%); **^{1}H NMR** (400 MHz, $CDCl_3$) δ 8.35 (d, *J* = 8.2 Hz, 1H), 8.24–8.08 (m, 6H), 8.01–7.95 (m, 3H), 5.74–5.65 (m, 1H), 1.83 (d, *J* = 6.4 Hz, 3H); **^{13}C NMR** (100 MHz, $CDCl_3$) δ 161.3, 137.2, 131.3, 130.6, 130.4, 127.7, 127.6, 127.4, 127.1, 125.9, 125.2, 125.1, 125.0, 124.9, 124.3, 122.5, 65.1, 24.3; **HRMS (ESI)** m/z: calcd. for $C_{38}H_{29}N_2$ $[M+H]^+$ 513.2325; Found 513.2321.

Complex Mo-6: A 100 mL Schleck round bottom flask was charged with $Mo(CO)_6$ (2.4 mmol, 634 mg), 30 mL THF and a solution of **ligand-f** (2.0 mmol, 1.03 g) in 10 mL THF under nitrogen atmosphere. The mixture was stirred at 60 ˚C for overnight. The solvent was removed in vacuo. The resulting mixture was washed with hexane and filtered through Gooch filter. The solid obtained was washed with 20 mL of hexane and dried in vacuo to afford 0.81 g (56% yields) of the title compound as a dark-brown solid. **^{1}H NMR** (400 MHz, $CDCl_3$) δ 8.26–8.19 (m, 3H), 8.15–8.01 (m, 6H), 7.45 (s, 1H), 6.57–6.52 (m, 1H), 2.19 (d, *J* = 5.8 Hz, 3H); **^{13}C NMR** (100 MHz, $CDCl_3$) δ 202.8, 157.4, 132.6, 131.6, 130.6, 129.1, 128.6, 128.1, 127.2, 126.3, 125.8, 125.7, 125.2, 124.5, 124.4, 124.3, 122.9, 67.2, 26.9; **HRMS (ESI)** m/z: calcd. for $C_{42}H_{29}MoN_2O_4$ $[M+H]^+$ 723.1176; Found 723.1161.

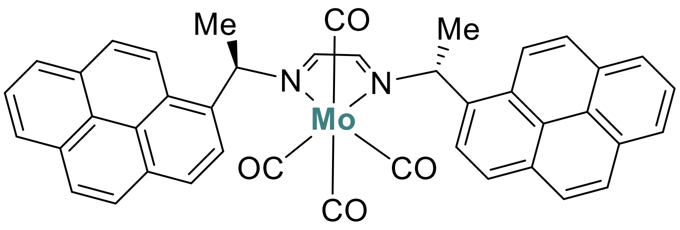

Mo-6

Optimization study for the amination of α-hydroxy esters

Table S1. Initial experiments and evaluation of different metal hexacarbonyl complexes[a]

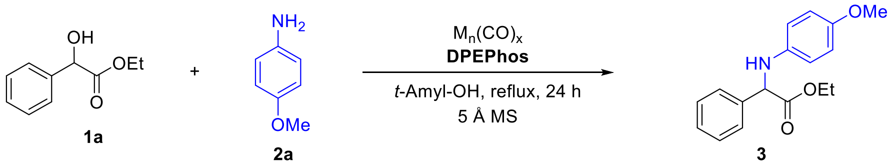

entry	$M_n(CO)_x$	yield (%)[b]
1	$Mo(CO)_6$	17
2	$W(CO)_6$	<5
3	$Cr(CO)_6$	<5
4	$Ru_3(CO)_{12}$	14

[a] Reaction conditions: $M_n(CO)_x$ (5 mol%), DPEPhos (6 mol%), **1a** (0.6 mmol, 3 equiv.), *p*-anisidine **2a** (0.2 mmol, 1 equiv.), *t*-Amyl-OH (2.0 mL), 5Å MS (80-100 mg), reflux (150 °C), 24 hours. [b] Isolated yields.

Table S2. Evaluation of additives and solvents[a]

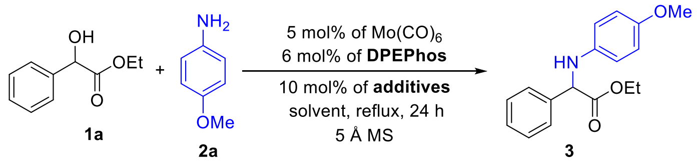

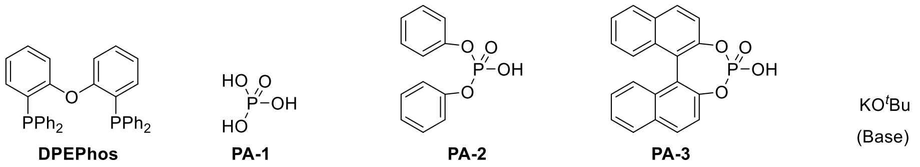

entry	additives	solvent	yield of **3** (%)[b]
1	no	*t*-Amyl-OH	17
2[c]	**PA-1**	*t*-Amyl-OH	24
3[c]	**PA-2**	*t*-Amyl-OH	37
4	**PA-3**	*t*-Amyl-OH	44
5	KO^{t}Bu	*t*-Amyl-OH	12
6	**PA-3**	toluene	<10
7	**PA-3**	dioxane	18
8[d]	**PA-3**	*t*-Amyl-OH	52
9[e]	**PA-3**	*t*-Amyl-OH	21
10[f]	**PA-3**	*t*-Amyl-OH	36

[a] Reaction conditions: $Mo(CO)_6$ (5 mol%), DPEPhos (6 mol%), **1a** (0.6 mmol, 3 equiv.), *p*-anisidine **2a** (0.2 mmol, 1 equiv.), solvent (2.0 mL), additives (10 mol%), 5Å MS (80-100 mg), reflux (150 °C), 24 hours. [b] Isolated yields. [c] 20 mol% of phosphoric acid (**PA**) was used. [d] Reaction was performed with 10 mol% of $Mo(CO)_6$ and 12 mol% of DPEPhos. [e] Reaction was performed at 100 °C. [f] Reaction was performed with 2 mol% of $Ru_3(CO)_{12}$ and 6 mol% of DPEPhos.

Table S3. Evaluation of ligand[a]

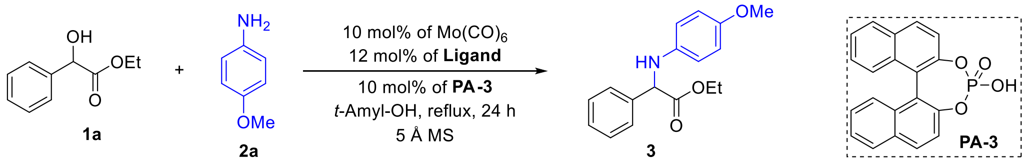

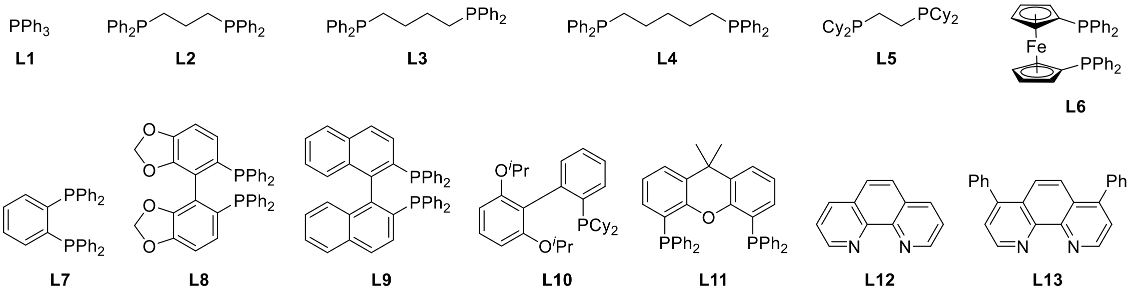

entry	**1a**:**2a**	ligand	yield of **3** (%)[b]
1	3:1	**L1**	23
2	3:1	**L2**	34
3	3:1	**L3**	75
4	3:1	**L4**	62
5	3:1	**L5**	21
6	3:1	**L6**	25
7	3:1	**L7**	16
8	3:1	**L8**	63
9	3:1	**L9**	35
10	3:1	**L10**	10
11	3:1	**L11**	38
12	3:1	**L12**	12
13	3:1	**L13**	10
14	2:1	**L3**	68
15	1:1	**L3**	52
16[c]	3:1	**L3**	62

[a] Reaction conditions: $Mo(CO)_6$ (10 mol%), ligand (12 mol%), **1a** (0.6 mmol, 3 equiv.), *p*-anisidine **2a** (0.2 mmol, 1 equiv.), *t*-Amyl-OH (2.0 mL), PA-**3** (10 mol%), 5Å MS (80-100 mg), reflux (150 °C), 24 hours. [b] Isolated yields. [c] Reaction was performed with 5 mol% of $Mo(CO)_6$ and 6 mol% of **L3**.

General procedure A for molybdenum-catalysed amination of α-hydroxy esters via borrowing hydrogen.

To an oven dried two-necked round-bottom flask equipped with a magnetic stir bar, 5Å MS (80-100 mg), $Mo(CO)_6$ (5.3 mg, 10 mol%), ligand **L3** (10.2 mg, 12 mol%), phosphoric acid PA-**3** (7.0 mg, 10 mol%), α-hydroxy ester **1a** (108.12 mg, 0.6 mmol), and *p*-anisidine **2a** (24.63 mg, 0.2 mmol) were added. Anhydrous *t*-amyl-OH (2 mL) was added and one neck of the flask was then fitted with reflux condenser and the other neck was sealed tightly using screw cap and paraffin film. The flask was then purged with nitrogen through reflux condenser and the resulting mixture was refluxed at 150 ˚C for 24 hours. The reaction mixture was then transferred to a 50 mL round bottom flask and the solvent was removed *in vacuo* with the aid of a rotary evaporator. The obtained residue was purified by flash column chromatography on silica gel to afford pure α-amino ester **3**. Other products (**4**–**46**) were prepared by following the same procedure.

ethyl 2-((4-methoxyphenyl)amino)-2-phenylacetate (3)[4a]

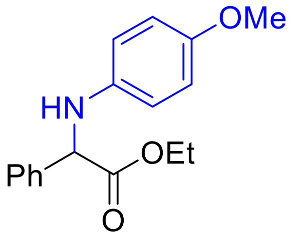

3

Compound **3** was prepared according to the general procedure A and was purified by flash column chromatography (Petroleum ether/EtOAc = 30:1) to afford the title compound as a light-yellow oil with 75% of isolated yield (42.8 mg). **^{1}H NMR** (400 MHz, $CDCl_3$) δ 7.48 (d, *J* = 7.2 Hz, 2H), 7.37–7.29 (m, 3H), 6.71 (d, *J* = 8.2 Hz, 2H), 6.53 (d, *J* = 8.2 Hz, 2H), 5.00 (s, 1H), 4.71 (bs, 1H), 4.27–4.08 (m, 2H), 3.70 (s, 3H), 1.20 (t, *J* = 7.2 Hz, 3H); **^{13}C NMR** (100 MHz, $CDCl_3$) δ 172.0, 152.4, 140.2, 137.8, 128.7, 128.1, 127.2, 114.8, 114.7, 61.7, 61.6, 55.7, 14.0. All other data are in accordance with the reported literature.[4a]

ethyl 2-phenyl-2-(phenylamino)acetate (4)[4a]

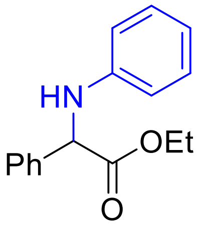

4

Compound **4** was prepared according to the general procedure A and was purified by flash column chromatography (Petroleum ether/EtOAc = 50:1) to afford the title compound as a

light-yellow oil with 72% of isolated yield (36.7 mg). **^{1}H NMR** (400 MHz, $CDCl_3$) δ 7.49 (d, *J* = 7.2 Hz, 2H), 7.36–7.28 (m, 3H), 7.11 (t, *J* = 6.2 Hz, 2H), 6.69 (t, *J* = 6.5 Hz, 1H), 6.55 (t, *J* = 7.2 Hz, 2H), 5.01 (s, 1H), 4.96 (bs, 1H), 4.27–4.09 (m, 2H), 1.21 (t, *J* = 7.2 Hz, 3H); **^{13}C NMR** (100 MHz, $CDCl_3$) δ 171.8, 146.0, 137.7, 129.2, 128.8, 128.2, 127.2, 118.0, 113.4, 61.8, 60.8, 14.0. All other data are in accordance with the reported literature.[4a]

ethyl 2-phenyl-2-(p-tolylamino)acetate (5)[4b]

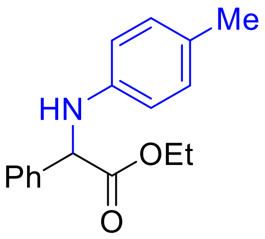

5

Compound **5** was prepared according to the general procedure A and was purified by flash column chromatography (Petroleum ether/EtOAc = 50:1) to afford the title compound as a light-yellow oil with 78% of isolated yield (41.6 mg). **^{1}H NMR** (400 MHz, $CDCl_3$) δ 7.49 (d, *J* = 6.8 Hz, 2H), 7.36–7.29 (m, 3H), 6.92 (d, *J* = 7.5 Hz, 2H), 6.48 (d, *J* = 7.5 Hz, 2H), 5.03 (s, 1H), 4.82 (bs, 1H), 4.27–4.09 (m, 2H), 2.19 (s, 3H), 1.21 (t, *J* = 7.2 Hz, 3H); **^{13}C NMR** (100 MHz, $CDCl_3$) δ 171.9, 143.7, 137.8, 129.7, 128.7, 128.1, 127.2, 127.1, 113.5, 61.7, 61.0, 20.4, 14.0. All other data are in accordance with the reported literature.[4b]

ethyl 2-((4-bromophenyl)amino)-2-phenylacetate (6)[4b]

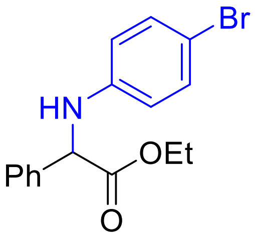

6

Compound **6** was prepared according to the general procedure A and was purified by flash column chromatography (Petroleum ether/EtOAc = 30:1) to afford the title compound as a light-yellow oil with 67% of isolated yield (44.8 mg). **^{1}H NMR** (400 MHz, $CDCl_3$) δ 7.46 (d, *J* = 7.4 Hz, 2H), 7.37–7.29 (m, 3H), 7.19 (d, *J* = 7.4 Hz, 2H), 6.42 (d, *J* = 7.4 Hz, 2H), 5.00 (s, 1H), 4.28–4.09 (m, 2H), 1.21 (t, *J* = 7.2 Hz, 3H); **^{13}C NMR** (100 MHz, $CDCl_3$) δ 171.4, 144.8, 137.1, 131.9, 128.9, 128.4, 127.1, 115.0, 109.7, 62.0, 60.6, 14.0. All other data are in accordance with the reported literature.[4b]

butyl 4-((2-ethoxy-2-oxo-1-phenylethyl)amino)benzoate (7)

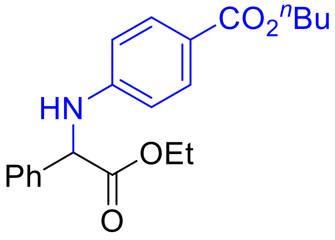

7

Compound **7** was prepared according to the general procedure A and was purified by flash column chromatography (Petroleum ether/EtOAc = 30:1) to afford the title compound as a light-yellow oil with 62% of isolated yield (44.1 mg). **^{1}H NMR** (400 MHz, $CDCl_3$) δ 7.81 (d, *J* = 6.2 Hz, 2H), 7.47 (d, *J* = 6.2 Hz, 2H), 7.37–7.30 (m, 3H), 6.51 (d, *J* = 6.4 Hz, 2H), 5.44 (bs, 1H), 5.10 (s, 1H), 4.30–4.10 (m, 4H), 1.72–1.64 (m, 2H), 1.47–1.37 (m, 2H), 1.21 (t, *J* = 7.2 Hz, 3H), 0.94 (t, *J* = 7.2 Hz, 3H); **^{13}C NMR** (100 MHz, $CDCl_3$) δ 171.2, 166.7, 149.4, 136.9, 131.4, 128.9, 128.4, 127.1, 119.6, 112.3, 64.1, 62.1, 60.1, 30.9, 19.3, 14.0, 13.8; **HRMS (ESI)** m/z: calcd. for $C_{21}H_{26}NO_4$ $[M+H]^+$ 356.1856; Found 356.1858.

ethyl 2-phenyl-2-(o-tolylamino)acetate (9)[4a]

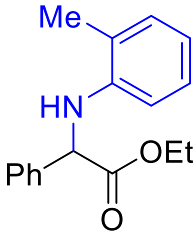

8

Compound **8** was prepared according to the general procedure A and was purified by flash column chromatography (Petroleum ether/EtOAc = 50:1) to afford the title compound as a light-yellow oil with 74% of isolated yield (39.8 mg). **^{1}H NMR** (400 MHz, $CDCl_3$) δ 7.50 (d, *J* = 7.4 Hz, 2H), 7.36–7.28 (m, 3H), 7.01 (d, *J* = 7.4 Hz, 1H), 6.96 (t, *J* = 7.4 Hz, 1H), 6.63 (d, *J* = 7.4 Hz, 1H), 6.33 (d, *J* = 7.4 Hz, 1H), 5.09 (s, 1H), 4.89 (bs, 1H), 4.27–4.09 (m, 2H), 2.27 (s, 3H), 1.20 (t, *J* = 7.2 Hz, 3H); **^{13}C NMR** (100 MHz, $CDCl_3$) δ 172.2, 144.0, 137.8, 130.2, 128.8, 128.1, 127.1, 127.0, 122.4, 117.6, 110.7, 61.8, 60.8, 17.5, 14.0; **HRMS (ESI)** m/z: calcd. for $C_{17}H_{20}NO_2$ $[M+H]^+$ 270.1489; Found 270.1486.

ethyl 2-((2-benzoylphenyl)amino)-2-phenylacetate (9)

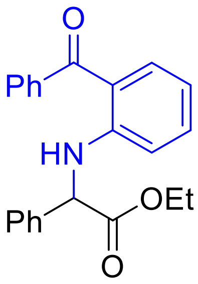

9

Compound **9** was prepared according to the general procedure A and was purified by flash column chromatography (Petroleum ether/EtOAc = 30:1) to afford the title compound as a light-yellow oil with 61% of isolated yield (43.8 mg). **^{1}H NMR** (400 MHz, $CDCl_3$) δ 9.57 (bs, 1H), 7.66 (d, *J* = 7.4 Hz, 2H), 7.56–7.43 (m, 6H), 7.28–7.24 (m, 4H), 6.57 (t, *J* = 7.2 Hz, 1H), 6.52 (d, *J* = 7.4 Hz, 1H), 5.20 (d, *J* = 2.7 Hz, 1H), 4.30–4.13 (m, 2H), 2.27 (s, 3H), 1.23 (t, *J* = 7.2 Hz, 3H); **^{13}C NMR** (100 MHz, $CDCl_3$) δ 199.2, 170.8, 149.4, 140.2, 137.1, 135.4, 134.7, 130.9, 129.2, 128.9, 128.3, 128.0, 127.2, 118.4, 114.9, 112.3, 61.9, 60.5, 14.1; **HRMS (ESI)** m/z: calcd. for $C_{23}H_{22}NO_3$ $[M+H]^+$ 360.1594; Found 360.1592.

ethyl 2-((3-acetamidophenyl)amino)-2-phenylacetate (10)

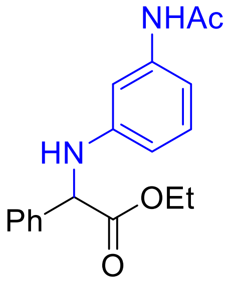

10

Compound **10** was prepared according to the general procedure A and was purified by flash column chromatography (Petroleum ether/EtOAc = 20:1) to afford the title compound as a light-yellow oil with 56% of isolated yield (35.0 mg). **^{1}H NMR** (400 MHz, $CDCl_3$) δ 7.57 (bs, 1H), 7.47 (d, *J* = 6.4 Hz, 2H), 7.34–7.25 (m, 3H), 6.99 (t, *J* = 8.4 Hz, 2H), 6.67 (d, *J* = 8.0 Hz, 1H), 6.25 (d, *J* = 8.0 Hz, 1H), 5.04 (s, 1H), 5.01 (bs, 1H), 4.27–4.07 (m, 2H), 2.04 (s, 3H), 1.19 (t, *J* = 6.8 Hz, 3H); **^{13}C NMR** (100 MHz, $CDCl_3$) δ 171.7, 168.5, 146.6, 139.1, 137.6, 129.6, 128.8, 128.2, 127.2, 109.4, 109.1, 105.0, 61.9, 60.7, 24.6, 14.0; **HRMS (ESI)** m/z: calcd. for $C_{18}H_{21}N_2O_3$ $[M+H]^+$ 313.1547; Found 313.1542.

ethyl 2-((2,6-dimethylphenyl)amino)-2-phenylacetate (11)

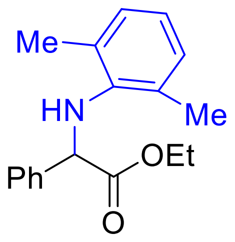

11

Compound **11** was prepared according to the general procedure A and was purified by flash column chromatography (Petroleum ether/EtOAc = 30:1) to afford the title compound as a light-yellow oil with 22% of isolated yield (12.5 mg). **^{1}H NMR** (400 MHz, $CDCl_3$) δ 7.35 (d, *J* = 7.2 Hz, 2H), 7.27–7.20 (m, 3H), 6.85 (d, *J* = 7.2 Hz, 2H), 6.69 (t, *J* = 13.5 Hz, 1H), 4.85 (s, 1H), 4.35 (bs, 1H), 4.12–3.98 (m, 2H), 2.18 (s, 3H), 2.17 (s, 3H), 1.09 (t, *J* = 6.4 Hz, 3H); **^{13}C NMR** (100 MHz, $CDCl_3$) δ 173.4, 144.3, 138.7, 129.0, 128.7, 128.6, 128.0, 127.0, 121.7, 63.9, 61.5, 19.0, 14.0; **HRMS (ESI)** m/z: calcd. for $C_{18}H_{22}NO_2$ $[M+H]^+$ 284.1645; Found 284.1643.

ethyl 2-((4-methoxy-3,5-dimethylphenyl)amino)-2-phenylacetate (12)

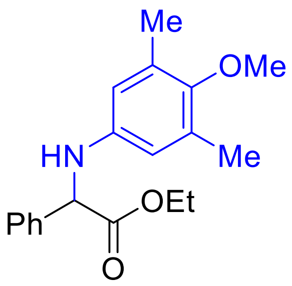

12

Compound **12** was prepared according to the general procedure A and was purified by flash column chromatography (Petroleum ether/EtOAc = 30:1) to afford the title compound as a light-yellow oil with 81% of isolated yield (50.8 mg). **^{1}H NMR** (400 MHz, $CDCl_3$) δ 7.48 (d, *J* = 7.2 Hz, 2H), 7.37–7.28 (m, 3H), 6.23 (s, 2H), 4.98 (s, 1H), 4.67 (bs, 1H), 4.26–4.07 (m, 2H), 3.60 (s, 3H), 2.16 (s, 6H), 1.20 (t, *J* = 6.2 Hz, 3H); **^{13}C NMR** (100 MHz, $CDCl_3$) δ 172.1, 149.4, 142.2, 140.0, 131.4, 128.8, 128.2, 127.2, 113.5, 61.7, 61.3, 59.9, 16.3, 14.1; **HRMS (ESI)** m/z: calcd. for $C_{19}H_{24}NO_3$ $[M+H]^+$ 314.1751; Found 314.1745.

ethyl 2-phenyl-2-((3,4,5-trimethoxyphenyl)amino)acetate (13)

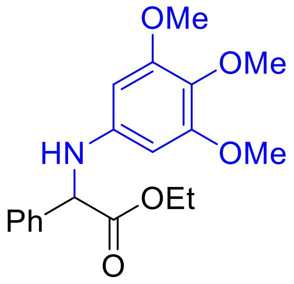

13

Compound **13** was prepared according to the general procedure A and was purified by flash column chromatography (Petroleum ether/EtOAc = 30:1) to afford the title compound as a light-yellow oil with 85% of isolated yield (58.7 mg). **^{1}H NMR** (400 MHz, $CDCl_3$) δ 7.49 (d, *J* = 7.2 Hz, 2H), 7.37–7.28 (m, 3H), 5.80 (s, 2H), 5.01 (s, 1H), 4.88 (bs, 1H), 4.28–4.09 (m, 2H), 3.72 (s, 9H), 1.21 (t, *J* = 6.2 Hz, 3H); **^{13}C NMR** (100 MHz, $CDCl_3$) δ 171.8, 153.8, 142.8, 137.8, 130.4, 128.9, 128.3, 127.2, 91.1, 61.9, 61.3, 61.0, 55.8, 14.1; **HRMS (ESI)** m/z: calcd. for $C_{19}H_{24}NO_5$ $[M+H]^+$ 346.1649; Found 346.1644.

ethyl 2-((3-chloro-4-fluorophenyl)amino)-2-phenylacetate (14)[4c]

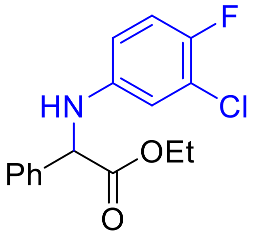

14

Compound **14** was prepared according to the general procedure A and was purified by flash column chromatography (Petroleum ether/EtOAc = 30:1) to afford the title compound as a white solid with 64% of isolated yield (39.4 mg). **^{1}H NMR** (400 MHz, $CDCl_3$) δ 7.49 (d, *J* = 7.4 Hz, 2H), 7.41–7.34 (m, 3H), 6.90 (t, *J* = 8.9 Hz, 1H), 6.58 (d, *J* = 2.4 Hz, 1H), 6.40 (d, *J* = 7.4 Hz, 1H), 5.00 (s, 1H), 5.00 (bs, 1H), 4.31–4.12 (m, 2H), 1.24 (t, *J* = 7.2 Hz, 3H); **^{13}C NMR** (100 MHz, $CDCl_3$) δ 171.4, 152.4, 150.0, 142.8, 136.9, 128.9, 128.4, 127.1, 121.1, 121.0, 116.9, 116.7, 114.5, 112.5, 62.0, 60.9, 14.0. All other data are in accordance with that reported in literature.[4c]

ethyl 2-(naphthalen-2-ylamino)-2-phenylacetate (15)[4d]

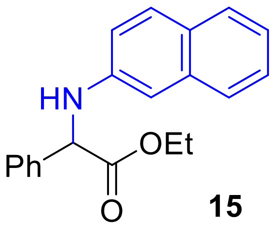

15

Compound **15** was prepared according to the general procedure A and was purified by flash column chromatography (Petroleum ether/EtOAc = 30:1) to afford the title compound as a light-yellow semi solid with 74% of isolated yield (45.2 mg). **^{1}H NMR** (400 MHz, $CDCl_3$) δ 7.62 (d, *J* = 7.4 Hz, 2H), 7.54–7.47 (m, 3H), 7.36–7.27 (m, 4H), 7.16 (t, *J* = 8.8 Hz, 1H), 6.94 (d, *J* = 8.8 Hz, 1H), 6.64 (s, 1H), 5.18 (s, 1H), 5.14 (bs, 1H), 4.27–4.09 (m, 2H), 1.21 (t, *J* = 7.2 Hz, 3H); **^{13}C NMR** (100 MHz, $CDCl_3$) δ 171.8, 143.6, 137.5, 134.9, 129.1, 128.9, 128.3, 127.8, 127.6, 127.3, 126.3, 126.1, 122.3, 118.0, 105.7, 61.9, 60.8, 14.1; **HRMS (ESI)** m/z: calcd. for $C_{20}H_{20}NO_2$ $[M+H]^+$ 306.1489; Found 306.1487.

ethyl 2-(benzylamino)-2-phenylacetate (16)[4a]

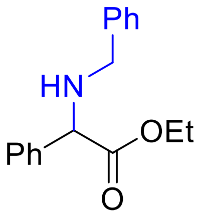

16

Compound **16** was prepared according to the general procedure A on 0.5 mmol scale, and was purified by flash column chromatography (Petroleum ether/EtOAc = 20:1) to afford the title compound as a colorless oil with 10% of isolated yield (13.5 mg). **^{1}H NMR** (400 MHz, $CDCl_3$) δ 7.39–7.22 (m, 10H), 4.37 (s, 1H), 4.22–4.06 (m, 2H), 3.73 (s, 2H), 2.30 (bs, 1H), 1.19 (t, *J* = 7.2 Hz, 3H); **^{13}C NMR** (100 MHz, $CDCl_3$) δ 173.0, 139.6, 138.2, 128.7, 128.4, 128.0, 127.6, 127.1, 64.5, 61.2, 51.4, 14.2. Other data are in accordance with the reported literature.[4a]

ethyl 2-(cyclohexylamino)-2-phenylacetate (17)[4a]

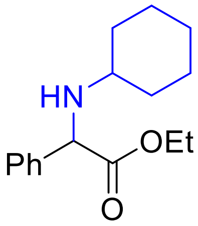

17

Compound **17** was prepared according to the general procedure A on 0.5 mmol scale, and was purified by flash column chromatography (Petroleum ether/EtOAc = 20:1) to afford the title compound as a colorless oil with 8% of isolated yield (10.5 mg). **^{1}H NMR** (400 MHz, $CDCl_3$) δ 7.38–7.28 (m, 5H), 4.51 (s, 1H), 4.23–4.09 (m, 2H), 2.37–2.34 (m, 1H), 2.05 (s, 1H), 1.87–1.82 (m, 2H), 1.72–1.70 (m, 2H), 1.59–1.57 (m, 1H), 1.22–1.16 (m, 8H); **^{13}C NMR** (100 MHz, $CDCl_3$) δ 173.6, 138.8, 128.6, 127.8, 127.3, 62.4, 61.1, 54.4, 33.5, 33.3, 26.0, 24.8, 14.1. All other data are in accordance with that reported in literature.[4a]

ethyl 2-phenyl-2-(pyrrolidin-1-yl)acetate (18)[4e]

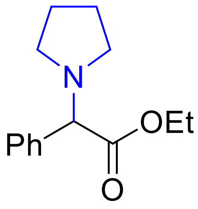

18

Compound **18** was prepared according to the general procedure A on 0.5 mmol scale, and was purified by flash column chromatography (Petroleum ether/EtOAc = 30:1) to afford the title compound as a light-yellow oil with 6% of isolated yield (7.0 mg). **^{1}H NMR** (400 MHz, $CDCl_3$) δ 7.48 (d, *J* = 6.8 Hz, 2H), 7.35–7.30 (m, 3H), 4.24–4.08 (m, 2H), 3.90 (s, 1H), 2.58–2.42 (m, 4H), 1.83–1.79 (m, 4H), 1.20 (t, *J* = 7.2 Hz, 3H); **^{13}C NMR** (100 MHz, $CDCl_3$) δ 171.7, 137.5, 128.5, 128.4, 128.2, 74.0, 60.9, 52.5, 23.4, 14.1. All other data are in accordance with that reported in literature.[4e]

ethyl 2-morpholino-2-phenylacetate (19)[4a]

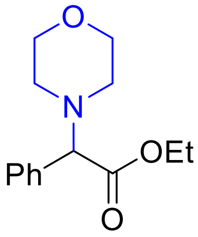

19

Compound **19** was prepared according to the general procedure A on 0.5 mmol scale, and was purified by flash column chromatography (Petroleum ether/EtOAc = 30:1) to afford the title compound as a light-yellow oil with 12% of isolated yield (15.0 mg). **^{1}H NMR** (400 MHz, $CDCl_3$) δ 7.46 (d, *J* = 7.2 Hz, 2H), 7.38–7.34 (m, 3H), 4.25–4.09 (m, 2H), 3.98 (s, 1H), 3.76–3.73 (m, 4H), 2.49–2.46 (m, 4H), 1.22 (t, *J* = 7.2 Hz, 3H); **^{13}C NMR** (100 MHz, $CDCl_3$) δ 171.2, 128.9, 128.6, 128.4, 74.5, 66.8, 61.0, 51.6, 14.1. All other data are in accordance with that reported in literature.[4a]

ethyl 2-(allyl(methyl)amino)-2-phenylacetate (20)

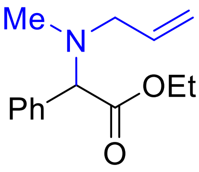

20

Compound **20** was prepared according to the general procedure A on 0.5 mmol scale, and was purified by flash column chromatography (Petroleum ether/EtOAc = 30:1) to afford the title compound as a light-yellow oil with 7% of isolated yield (7.0 mg). **^{1}H NMR** (400 MHz, $CDCl_3$)

δ 7.35 (d, *J* = 7.2 Hz, 2H), 7.28–7.22 (m, 3H), 5.85–5.75 (m, 1H), 5.08 (d, *J* = 7.6 Hz, 1H), 5.05 (s, 1H), 4.18–4.02 (m, 3H), 3.09–2.91 (m, 2H), 2.18 (s, 3H), 1.14 (t, *J* = 7.2 Hz, 3H); **^{13}C NMR** (100 MHz, $CDCl_3$) δ 171.8, 136.4, 135.0, 128.8, 128.4, 128.1, 117.9, 72.2, 60.7, 57.7, 39.2, 14.1; **HRMS (ESI)** m/z: calcd. for $C_{14}H_{20}NO_2$ $[M+H]^+$ 234.1489; Found 356.1490.

methyl 2-((4-methoxyphenyl)amino)-2-phenylacetate (21)[4f]

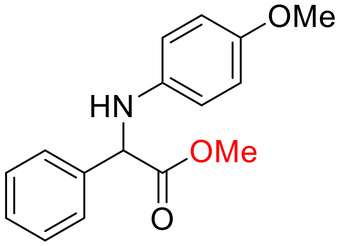

21

Compound **21** was prepared according to the general procedure A and was purified by flash column chromatography (Petroleum ether/EtOAc = 30:1) to afford the title compound as a white solid with 63% of isolated yield (34.2 mg). **^{1}H NMR** (400 MHz, $CDCl_3$) δ 7.53 (d, *J* = 7.2 Hz, 2H), 7.41–7.31 (m, 3H), 6.75 (dd, *J* = 3.8, 8.2 Hz, 2H), 6.57 (dd, *J* = 3.8, 8.2 Hz, 2H), 5.06 (s, 1H), 4.73 (bs, 1H), 3.76 (s, 3H), 3.74 (s, 3H); **^{13}C NMR** (100 MHz, $CDCl_3$) δ 172.5, 152.5, 140.1, 137.7, 128.8, 128.2, 127.2, 114.8, 114.7, 61.6, 55.6, 52.7. All other data are in accordance with that reported in literature.[4f]

benzyl 2-((4-methoxyphenyl)amino)-2-phenylacetate (22)[4f]

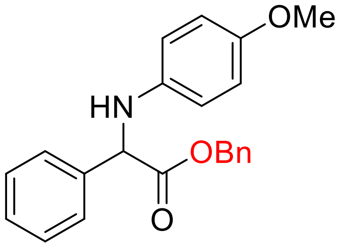

22

Compound **22** was prepared according to the general procedure A and was purified by flash column chromatography (Petroleum ether/EtOAc = 30:1) to afford the title compound as yellow oil with 74% of isolated yield (51.4 mg). **^{1}H NMR** (400 MHz, $CDCl_3$) δ 7.48 (d, *J* = 7.2 Hz, 2H), 7.36–7.28 (m, 6H), 7.17 (d, *J* = 5.4 Hz, 2H), 6.71 (d, *J* = 7.4 Hz, 2H), 6.54 (d, *J* = 7.4 Hz, 2H), 5.21–5.06 (m, 3H), 4.75 (bs, 1H), 3.71 (s, 3H); **^{13}C NMR** (100 MHz, $CDCl_3$) δ 171.9, 152.5, 140.1, 137.6, 135.3, 128.8, 128.5, 128.3, 127.9, 127.3, 114.8, 67.2, 61.8, 55.7. All other data are in accordance with that reported in literature.[4f]

isopropyl 2-((4-methoxyphenyl)amino)-2-phenylacetate (23)[4f]

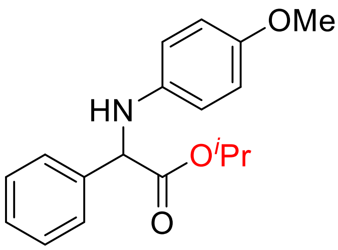

23

Compound **23** was prepared according to the general procedure A and was purified by flash column chromatography (Petroleum ether/EtOAc = 50:1) to afford the title compound as light-yellow oil with 76% of isolated yield (45.5 mg). **^{1}H NMR** (400 MHz, $CDCl_3$) δ 7.48 (d, *J* = 6.4 Hz, 2H), 7.36–7.28 (m, 3H), 6.72 (d, *J* = 6.8 Hz, 2H), 6.53 (d, *J* = 6.8 Hz, 2H), 5.05–5.00 (m, 1H), 4.97 (s, 1H), 4.70 (bs, 1H), 3.70 (s, 3H), 1.26 (d, *J* = 4.8 Hz, 3H), 1.08 (d, *J* = 4.8 Hz, 3H); **^{13}C NMR** (100 MHz, $CDCl_3$) δ 171.5, 152.4, 140.3, 137.9, 128.7, 128.1, 127.2, 114.8, 114.7, 69.3, 61.8, 55.7, 21.8, 21.4. All other data are in accordance with that reported in literature.[4f]

cyclohexyl 2-((4-methoxyphenyl)amino)-2-phenylacetate (24)[4g]

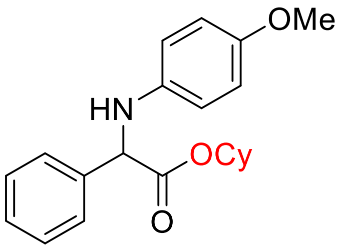

24

Compound **24** was prepared according to the general procedure A and was purified by flash column chromatography (Petroleum ether/EtOAc = 30:1) to afford the title compound as a colorless oil with 82% of isolated yield (55.6 mg). **^{1}H NMR** (400 MHz, $CDCl_3$) δ 7.53 (d, *J* = 6.7 Hz, 2H), 7.39–7.30 (m, 3H), 6.75 (d, *J* = 7.0 Hz, 2H), 6.57 (d, *J* = 7.0 Hz, 2H), 5.03 (s, 1H), 4.87–4.81 (m, 1H), 4.75 (bs, 1H), 3.73 (s, 3H), 1.89–1.84 (m, 1H), 1.76–1.71 (m, 1H), 1.65–1.48 (m, 3H), 1.41–1.25 (m, 5H); **^{13}C NMR** (100 MHz, $CDCl_3$) δ 171.5, 152.4, 140.3, 138.1, 128.7, 128.1, 127.2, 114.8, 114.7, 73.9, 61.8, 55.7, 31.4, 30.9, 25.3, 23.4, 23.2. All other data are in accordance with that reported in literature.[4g]

tert-butyl 2-((4-methoxyphenyl)amino)-2-phenylacetate (25)[4g]

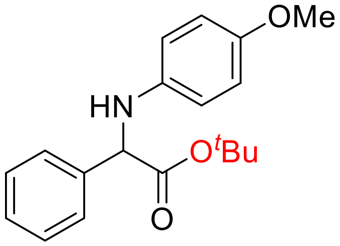

25

Compound **25** was prepared according to the general procedure A and was purified by flash column chromatography (Petroleum ether/EtOAc = 30:1) to afford the title compound as a white solid with 79% of isolated yield (49.5 mg). **^{1}H NMR** (400 MHz, $CDCl_3$) δ 7.48 (d, *J* = 6.4 Hz, 2H), 7.35–7.25 (m, 3H), 6.71 (d, *J* = 7.0 Hz, 2H), 6.52 (d, *J* = 7.0 Hz, 2H), 4.90 (s, 1H), 4.66 (bs, 1H), 3.70 (s, 3H), 1.37 (s, 9H); **^{13}C NMR** (100 MHz, $CDCl_3$) δ 171.1, 152.3, 140.4, 138.4, 128.6, 127.9, 127.1, 114.8, 114.6, 82.2, 62.1, 55.7, 27.8. All other data are in accordance with that reported in literature.[4g]

3,3,5-trimethylcyclohexyl 2-((4-methoxyphenyl)amino)-2-phenylacetate (26)

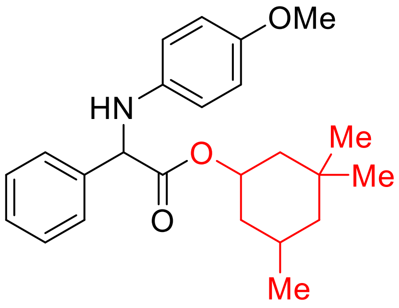

26

Compound **26** was prepared according to the general procedure A and was purified by flash column chromatography (Petroleum ether/EtOAc = 50:1) to afford the title compound as colorless oil with 58% of isolated yield (44.2 mg). **^{1}H NMR** (400 MHz, $CDCl_3$) δ 7.40 (d, *J* = 6.7 Hz, 2H), 7.29–7.17 (m, 3H), 6.64 (d, *J* = 7.2 Hz, 2H), 6.46 (d, *J* = 7.2 Hz, 2H), 4.88 (s, 1H), 4.86–4.79 (m, 1H), 4.60 (bs, 1H), 3.63 (s, 3H), 1.93–1.36 (m, 3H), 1.25–1.03 (m, 2H), 0.92–0.68 (m, 11H); **^{13}C NMR** (100 MHz, $CDCl_3$) δ 171.6, 152.4, 140.3, 138.0, 128.7, 128.1, 127.1, 114.8, 114.7, 72.7, 61.8, 55.7, 47.4, 43.3, 40.3, 32.9, 32.2, 27.1, 27.0, 25.4, 22.3; **HRMS (ESI)** m/z: calcd. for $C_{24}H_{32}NO_3$ $[M+H]^+$ 382.2377; Found 382.2375.

ethyl 2-(4-methoxyphenyl)-2-((4-methoxyphenyl)amino)acetate (27)

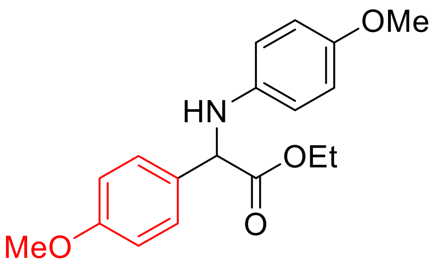

27

Compound **27** was prepared according to the general procedure A and was purified by flash column chromatography (Petroleum ether/EtOAc = 30:1) to afford the title compound as a light-yellow oil with 76% of isolated yield (47.9 mg). **^{1}H NMR** (400 MHz, $CDCl_3$) δ 7.40 (d, *J* = 7.8 Hz, 2H), 6.87 (d, *J* = 7.8 Hz, 2H), 6.72 (d, *J* = 7.8 Hz, 2H), 6.53 (d, *J* = 7.8 Hz, 2H), 4.94 (s, 1H), 4.68 (bs, 1H), 4.26–4.08 (m, 2H), 3.79 (s, 3H), 3.70 (s, 3H), 1.21 (t, *J* = 7.2 Hz, 3H); **^{13}C NMR** (100 MHz, $CDCl_3$) δ 172.3, 159.5, 152.4, 140.3, 129.8, 128.4, 114.8, 114.7, 114.2, 61.6, 61.1, 55.7, 55.3, 14.1; **HRMS (ESI)** m/z: calcd. for $C_{18}H_{22}NO_4$ $[M+H]^+$ 316.1543; Found 316.1542.

ethyl 2-(4-isopropylphenyl)-2-((4-methoxyphenyl)amino)acetate (28)

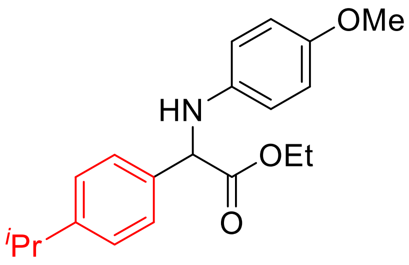

28

Compound **28** was prepared according to the general procedure A and was purified by flash column chromatography (Petroleum ether/EtOAc = 30:1) to afford the title compound as yellow oil with 78% of isolated yield (51.1 mg). **^{1}H NMR** (400 MHz, $CDCl_3$) δ 7.39 (d, *J* = 6.8 Hz, 2H), 7.20 (d, *J* = 6.8 Hz, 2H), 6.73 (d, *J* = 6.8 Hz, 2H), 6.56 (d, *J* = 6.8 Hz, 2H), 4.97 (s, 1H), 4.75 (bs, 1H), 4.27–4.07 (m, 2H), 3.70 (s, 3H), 2.92–2.85 (m, 1H), 1.29–1.19 (m, 9H); **^{13}C NMR** (100 MHz, $CDCl_3$) δ 172.3, 152.4, 148.8, 140.4, 135.1, 127.1, 126.9, 114.8, 114.7, 61.6, 61.5, 55.7, 33.8, 23.9, 14.1; **HRMS (ESI)** m/z: calcd. for $C_{20}H_{26}NO_3$ $[M+H]^+$ 328.1907; Found 328.1905.

ethyl 2-(4-fluorophenyl)-2-((4-methoxyphenyl)amino)acetate (29)

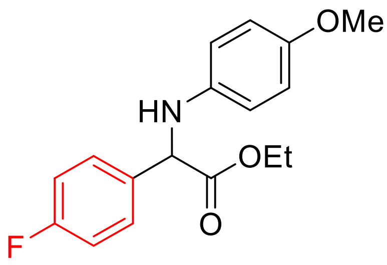

29

Compound **29** was prepared according to the general procedure A and was purified by flash column chromatography (Petroleum ether/EtOAc = 30:1) to afford the title compound as yellow oil with 62% of isolated yield (37.6 mg). **^{1}H NMR** (400 MHz, $CDCl_3$) δ 7.49–7.44 (m, 2H), 7.05–7.00 (m, 2H), 6.72 (d, *J* = 7.8 Hz, 2H), 6.51 (d, *J* = 7.8 Hz, 2H), 4.97 (s, 1H), 4.71 (bs, 1H), 4.26–4.09 (m, 2H), 3.70 (s, 3H), 1.21 (t, *J* = 7.2 Hz, 3H); **^{13}C NMR** (100 MHz, $CDCl_3$) δ 171.8, 163.8, 161.4, 152.5, 140.0, 133.7, 128.9, 128.8, 115.8, 115.6, 114.8, 61.9, 61.0, 55.7, 14.0; **HRMS (ESI)** m/z: calcd. for $C_{17}H_{19}FNO_3$ $[M+H]^+$ 304.1343; Found 304.1345.

ethyl 2-(2,5-dimethylphenyl)-2-((4-methoxyphenyl)amino)acetate (30)

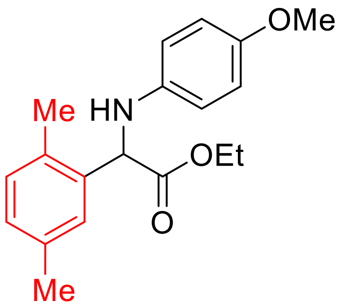

30

Compound **30** was prepared according to the general procedure A and was purified by flash column chromatography (Petroleum ether/EtOAc = 30:1) to afford the title compound as yellow oil with 72% of isolated yield (45.1 mg). **^{1}H NMR** (400 MHz, $CDCl_3$) δ 7.20 (s, 1H), 7.08 (d, *J* = 7.0 Hz, 1H), 7.00 (d, *J* = 7.0 Hz, 1H), 6.73 (d, *J* = 7.2 Hz, 2H), 6.51 (d, *J* = 7.2 Hz, 2H), 5.16 (s, 1H), 4.55 (bs, 1H), 4.27–4.06 (m, 2H), 3.70 (s, 3H), 2.47 (s, 3H), 2.26 (s, 3H), 1.20 (t, *J* = 7.2 Hz, 3H); **^{13}C NMR** (100 MHz, $CDCl_3$) δ 172.6, 152.5, 140.7, 136.0, 135.9, 133.4, 130.7, 128.8, 126.9, 114.9, 114.6, 61.5, 58.3, 55.7, 21.1, 19.1, 14.1; **HRMS (ESI)** m/z: calcd. for $C_{19}H_{24}NO_3$ $[M+H]^+$ 314.1751; Found 314.1747.

ethyl 2-((4-methoxyphenyl)amino)-2-(naphthalen-2-yl)acetate (31)

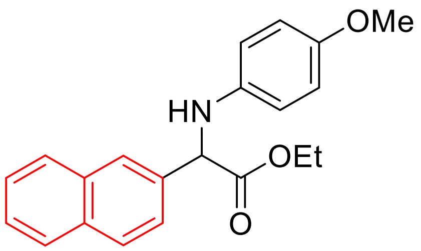

31

Compound **31** was prepared according to the general procedure A and was purified by flash column chromatography (Petroleum ether/EtOAc = 30:1) to afford the title compound as yellow oil with 76% of isolated yield (50.9 mg). **^{1}H NMR** (400 MHz, $CDCl_3$) δ 8.36 (d, *J* = 8.2 Hz, 1H), 7.94–7.85 (m, 2H), 7.70–7.45 (m, 4H), 6.76 (d, *J* = 7.0 Hz, 2H), 6.60 (d, *J* = 7.0 Hz, 2H), 5.82 (s, 1H), 4.78 (bs, 1H), 4.32–4.11 (m, 2H), 3.73 (s, 3H), 1.19 (t, *J* = 7.2 Hz, 3H); **^{13}C NMR** (100 MHz, $CDCl_3$) δ 172.0, 152.5, 140.5, 134.1, 133.6, 131.3, 129.0, 128.9, 126.5, 125.8, 125.5, 124.9, 123.5, 114.8, 114.5, 61.7, 58.3, 55.6, 14.0; **HRMS (ESI)** m/z: calcd. for $C_{21}H_{22}NO_3$ $[M+H]^+$ 336.1594; Found 336.1592.

ethyl 2-(6-bromonaphthalen-2-yl)-2-((4-methoxyphenyl)amino)acetate (32)

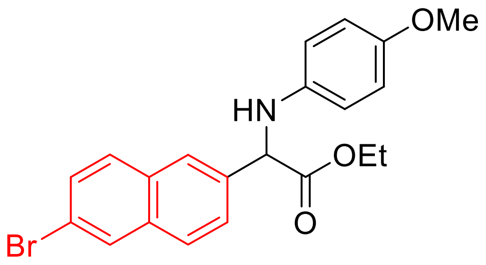

32

Compound **32** was prepared according to the general procedure A and was purified by flash column chromatography (Petroleum ether/EtOAc = 20:1) to afford the title compound as yellow solid with 68% of isolated yield (56.3 mg). **^{1}H NMR** (400 MHz, $CDCl_3$) δ 7.98 (s, 1H), 7.93 (s, 1H), 7.75–7.62 (m, 3H), 7.54 (d, *J* = 8.8 Hz, 1H), 6.70 (d, *J* = 7.2 Hz, 2H), 6.55 (d, *J* = 7.2 Hz, 2H), 5.13 (s, 1H), 4.83 (bs, 1H), 4.27–4.08 (m, 2H), 3.69 (s, 3H), 1.20 (t, *J* = 7.2 Hz, 3H); **^{13}C NMR** (100 MHz, $CDCl_3$) δ 171.7, 152.6, 140.0, 136.1, 134.3, 131.8, 129.8, 129.7, 129.6, 127.7, 126.3, 126.1, 120.2, 114.9, 114.8, 62.0, 61.8, 55.7, 14.1; **HRMS (ESI)** m/z: calcd. for $C_{21}H_{21}BrNO_3$ $[M+H]^+$ 414.0699; Found 414.0695.

ethyl 2-(2,3-dihydrobenzofuran-5-yl)-2-((4-methoxyphenyl)amino)acetate (33)

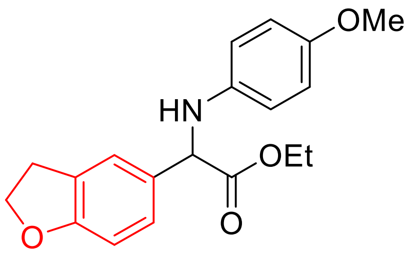

33

Compound **33** was prepared according to the general procedure A and was purified by flash column chromatography (Petroleum ether/EtOAc = 30:1) to afford the title compound as a light-yellow oil with 78% of isolated yield (51.0 mg). ^{1}**H NMR** (400 MHz, $CDCl_3$) δ 7.30 (s, 1H), 7.21 (d, *J* = 7.7 Hz, 1H), 6.76–6.71 (m, 3H), 6.55 (d, *J* = 6.6 Hz, 2H), 4.19 (s, 1H), 4.56 (t, *J* = 7.8 Hz, 2H), 4.27–4.07 (m, 2H), 3.71 (s, 3H), 3.18 (t, *J* = 7.8 Hz, 2H), 1.22 (t, *J* = 7.2 Hz, 3H); ^{13}**C NMR** (100 MHz, $CDCl_3$) δ 172.4, 160.1, 152.5, 140.2, 129.7, 127.7, 127.4, 123.6, 121.2, 114.8, 109.4, 71.4, 61.6, 61.4, 55.7, 29.7, 14.1; **HRMS (ESI)** m/z: calcd. for $C_{19}H_{22}NO_4$ $[M+H]^+$ 328.1543; Found 328.1541.

methyl 2-(2,3-dihydrobenzofuran-5-yl)-2-((4-methoxyphenyl)amino)acetate (34)

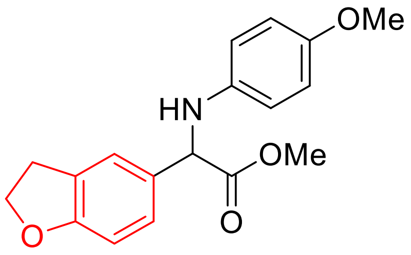

34

Compound **34** was prepared according to the general procedure A and was purified by flash column chromatography (Petroleum ether/EtOAc = 30:1) to afford the title compound as yellow solid with 53% of isolated yield (33.2 mg). ^{1}**H NMR** (400 MHz, $CDCl_3$) δ 7.33 (s, 1H), 7.24 (d, *J* = 8.0 Hz, 1H), 6.79–6.74 (m, 3H), 6.57 (d, *J* = 6.9 Hz, 2H), 4.96 (s, 1H), 4.67 (bs, 1H), 4.58 (t, *J* = 7.8 Hz, 2H), 3.74 (s, 3H), 3.73 (s, 3H), 3.20 (t, *J* = 7.8 Hz, 2H); ^{13}**C NMR** (100 MHz, $CDCl_3$) δ 173.0, 160.2, 152.5, 140.3, 129.7, 127.8, 127.4, 123.7, 114.8, 114.7, 109.4, 71.4, 61.3, 55.7, 52.6, 29.6; **HRMS (ESI)** m/z: calcd. for $C_{18}H_{20}NO_4$ $[M+H]^+$ 314.1387; Found 314.1385.

ethyl 2-(benzo[d][1,3]dioxol-5-yl)-2-((4-methoxyphenyl)amino)acetate (35)

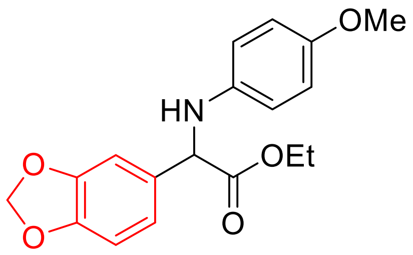

35

Compound **35** was prepared according to the general procedure A and was purified by flash column chromatography (Petroleum ether/EtOAc = 30:1) to afford the title compound as a light-yellow oil with 76% of isolated yield (50.0 mg). **^{1}H NMR** (400 MHz, $CDCl_3$) δ 6.97–6.95 (m, 2H), 6.77 (d, *J* = 8.2 Hz, 1H), 6.72 (d, *J* = 7.4 Hz, 2H), 6.52 (d, *J* = 7.4 Hz, 2H), 5.94 (s, 2H), 4.90 (s, 1H), 4.64 (bs, 1H), 4.27–4.08 (m, 2H), 3.71 (s, 3H), 1.22 (t, *J* = 7.2 Hz, 2H); **^{13}C NMR** (100 MHz, $CDCl_3$) δ 172.0, 152.4, 148.1, 147.5, 140.1, 131.8, 120.8, 114.8, 114.7, 108.4, 107.5, 101.2, 61.7, 61.3, 55.7, 14.1; **HRMS (ESI)** m/z: calcd. for $C_{18}H_{20}NO_5$ $[M+H]^+$ 330.1336; Found 330.1332.

methyl 2-(benzo[d][1,3]dioxol-5-yl)-2-((4-methoxyphenyl)amino)acetate (36)

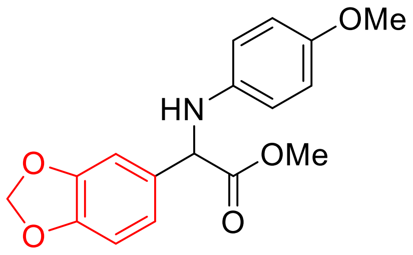

36

Compound **36** was prepared according to the general procedure A and was purified by flash column chromatography (Petroleum ether/EtOAc = 30:1) to afford the title compound as yellow solid with 64% of isolated yield (40.3 mg). **^{1}H NMR** (400 MHz, $CDCl_3$) δ 6.96 (s, 2H), 6.78 (d, *J* = 8.2 Hz, 1H), 6.72 (d, *J* = 7.4 Hz, 2H), 6.52 (d, *J* = 7.4 Hz, 2H), 5.95 (s, 2H), 4.91 (s, 1H), 4.69 (bs, 1H), 3.72 (s, 3H), 3.71 (s, 3H); **^{13}C NMR** (100 MHz, $CDCl_3$) δ 172.5, 152.5, 148.1, 147.6, 140.0, 131.6, 120.8, 114.8, 114.7, 108.5, 107.5, 101.2, 61.3, 55.7, 52.8; **HRMS (ESI)** m/z: calcd. for $C_{18}H_{18}NO_5$ $[M+H]^+$ 316.1179; Found 316.1175.

ethyl 2-((4-methoxyphenyl)amino)-2-(thiophen-2-yl)acetate (37)

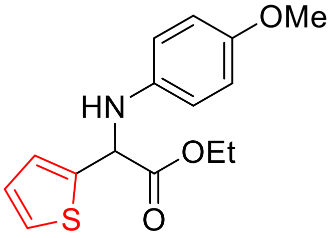

37

Compound **37** was prepared according to the general procedure A and was purified by flash column chromatography (Petroleum ether/EtOAc = 50:1) to afford the title compound as yellow oil with 68% of isolated yield (39.6 mg). **^{1}H NMR** (400 MHz, $CDCl_3$) δ 7.25–7.23 (m, 1H), 7.14–7.10 (m, 1H), 6.99–6.94 (m, 1H), 6.76 (d, *J* = 7.2 Hz, 2H), 6.63 (d, *J* = 7.2 Hz, 2H), 5.26 (s, 1H), 4.76 (bs, 1H), 4.28–4.16 (m, 2H), 3.73 (s, 3H), 1.26 (t, *J* = 7.2 Hz, 3H); **^{13}C NMR** (100 MHz, $CDCl_3$) δ 171.1, 153.0, 141.6, 140.0, 127.0, 125.5, 125.4, 115.3, 114.8, 62.0, 58.0, 55.7, 14.1; **HRMS (ESI)** m/z: calcd. for $C_{15}H_{18}NO_3S$ $[M+H]^+$ 292.1002; Found 292.1004.

methyl 2-((4-methoxyphenyl)amino)-2-(thiophen-2-yl)acetate (38)

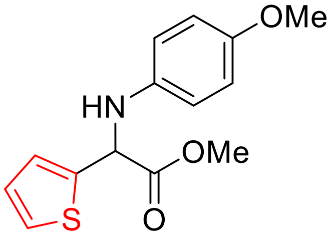

38

Compound **38** was prepared according to the general procedure A and was purified by flash column chromatography (Petroleum ether/EtOAc = 50:1) to afford the title compound as yellow solid with 56% of isolated yield (31.0 mg). **^{1}H NMR** (400 MHz, $CDCl_3$) δ 7.25–7.23 (m, 1H), 7.13–7.11 (m, 1H), 6.99–6.96 (m, 1H), 6.75 (d, *J* = 7.6 Hz, 2H), 6.61 (d, *J* = 7.6 Hz, 2H), 5.28 (s, 1H), 4.63 (bs, 1H), 3.77 (s, 3H), 3.72 (s, 3H); **^{13}C NMR** (100 MHz, $CDCl_3$) δ 171.7, 153.0, 141.5, 139.9, 127.1, 125.6, 125.5, 115.2, 114.8, 57.8, 55.6, 52.9; **HRMS (ESI)** m/z: calcd. for $C_{14}H_{16}NO_3S$ $[M+H]^+$ 278.0845; Found 278.0843.

ethyl 2-((4-methoxyphenyl)amino)-2-(pyren-2-yl)acetate (39)

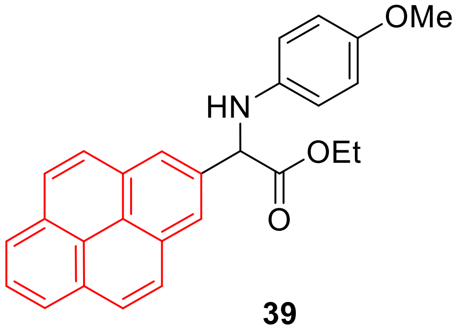

39

Compound **39** was prepared according to the general procedure A and was purified by flash column chromatography (Petroleum ether/EtOAc = 30:1) to afford the title compound as yellow solid with 70% of isolated yield (57.3 mg). **^{1}H NMR** (400 MHz, $CDCl_3$) δ 8.60 (d, *J* = 9.2 Hz, 1H), 8.23–8.12 (m, 5H), 8.08–8.00 (m, 3H), 6.67 (d, *J* = 8.3 Hz, 2H), 6.55 (d, *J* = 8.3 Hz, 2H), 6.06 (s, 1H), 4.98 (bs, 1H), 4.27–4.04 (m, 2H), 3.63 (s, 3H), 1.10 (t, *J* = 7.2 Hz, 3H); **^{13}C NMR** (100 MHz, $CDCl_3$) δ 172.3, 152.5, 140.4, 131.5, 131.3, 131.2, 130.7, 129.1, 128.2, 127.7, 127.4, 126.1, 125.5, 125.3, 125.2, 124.8, 124.5, 122.8, 114.9, 114.6, 61.9, 58.4, 55.6, 14.0; **HRMS (ESI)** m/z: calcd. for $C_{27}H_{24}NO_3$ $[M+H]^+$ 410.1751; Found 410.1745.

methyl 2-((4-methoxyphenyl)amino)-2-(pyren-2-yl)acetate (40)

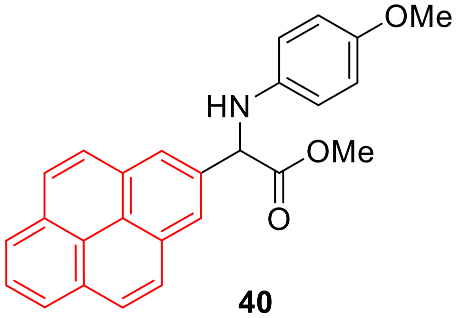

40

Compound **40** was prepared according to the general procedure A and was purified by flash column chromatography (Petroleum ether/EtOAc = 30:1) to afford the title compound as yellow solid with 51% of isolated yield (40.3 mg). **^{1}H NMR** (400 MHz, $CDCl_3$) δ 8.56 (d, *J* = 9.2 Hz, 1H), 8.21–8.11 (m, 5H), 8.06–7.98 (m, 3H), 6.65 (d, *J* = 8.0 Hz, 2H), 6.55 (d, *J* = 8.0 Hz, 2H), 6.06 (s, 1H), 5.01 (bs, 1H), 3.66 (s, 3H), 3.62 (s, 3H); **^{13}C NMR** (100 MHz, $CDCl_3$) δ 172.9, 152.6, 140.3, 131.4, 131.3, 131.2, 130.7, 129.1, 128.4, 127.8, 127.4, 126.2, 125.6, 125.4, 125.3, 125.2, 124.8, 124.6, 122.6, 114.9, 114.7, 58.3, 55.6, 52.9; **HRMS (ESI)** m/z: calcd. for $C_{26}H_{22}NO_3$ $[M+H]^+$ 396.1594; Found 396.1590.

ethyl (4-methoxyphenyl)alaninate (41)[4h]

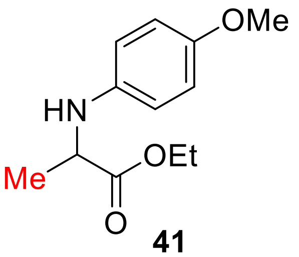

41

Compound **41** was prepared according to the general procedure A and was purified by flash column chromatography (Petroleum ether/EtOAc = 50:1) to afford the title compound as yellow oil with 62% of isolated yield (27.6 mg). **^{1}H NMR** (400 MHz, $CDCl_3$) δ 6.77 (d, J = 7.4 Hz, 2H), 6.60 (d, J = 7.4 Hz, 2H), 4.20–4.14 (m, 2H), 4.08–4.02 (m, 1H), 3.73 (s, 3H), 1.45 (d, J = 6.7 Hz, 3H), 1.24 (t, J = 7.2 Hz, 3H); **^{13}C NMR** (100 MHz, $CDCl_3$) δ 174.9, 152.8, 140.7, 115.2, 114.9, 61.0, 55.7, 53.2, 19.0, 14.2. All other data are in accordance with that reported in literature.[4h]

methyl (4-methoxyphenyl)alaninate (42)

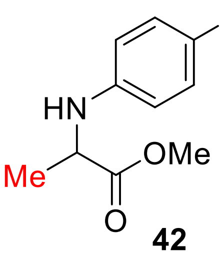

42

Compound **42** was prepared according to the general procedure A and was purified by flash column chromatography (Petroleum ether/EtOAc = 50:1) to afford the title compound as yellow oil with 58% of isolated yield (24.2 mg). **^{1}H NMR** (400 MHz, $CDCl_3$) δ 6.77 (d, J = 8.0 Hz, 2H), 6.59 (d, J = 8.0 Hz, 2H), 4.10–4.04 (m, 1H), 3.74 (s, 3H), 3.71 (s, 3H), 1.46 (d, J = 6.4 Hz, 3H); **^{13}C NMR** (100 MHz, $CDCl_3$) δ 175.4, 152.8, 140.7, 115.1, 114.9, 55.7, 53.1, 52.2, 19.1. **HRMS (ESI)** m/z: calcd. for $C_{11}H_{16}NO_3$ $[M+H]^+$ 210.1125; Found 210.1123.

ethyl 2-((4-methoxyphenyl)amino)hexanoate (43)[4i]

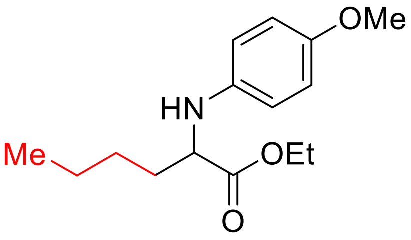

43

Compound **43** was prepared according to the general procedure A and was purified by flash column chromatography (Petroleum ether/EtOAc = 50:1) to afford the title compound as light-yellow oil with 74% of isolated yield (39.2 mg). **^{1}H NMR** (400 MHz, $CDCl_3$) δ 6.78 (d, J

= 8.2 Hz, 2H), 6.62 (d, *J* = 8.2 Hz, 2H), 4.17 (q, *J* = 7.2 Hz, 2H), 3.98 (t, *J* = 6.2 Hz, 1H), 3.88 (bs, 1H), 3.76 (s, 3H), 1.86–1.72 (m, 2H), 1.45–1.34 (m, 2H), 1.26 (t, *J* = 8.2 Hz, 3H), 0.93 (d, *J* = 6.2 Hz, 3H); **^{13}C NMR** (100 MHz, $CDCl_3$) δ 174.6, 152.7, 141.1, 115.1, 114.9, 60.9, 57.9, 55.7, 32.9, 27.8, 22.5, 14.3, 13.9. All other data are in accordance with that reported in literature.[4i]

ethyl 2-((4-methoxyphenyl)amino)-4-phenylbutanoate (44)[2f]

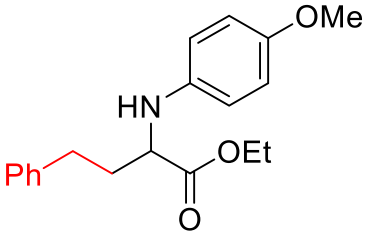

44

Compound **44** was prepared according to the general procedure A and was purified by flash column chromatography (Petroleum ether/EtOAc = 30:1) to afford the title compound as light-yellow oil with 78% of isolated yield (48.8 mg). **^{1}H NMR** (400 MHz, $CDCl_3$) δ 7.35–7.30 (m, 2H), 7.28–7.22 (m, 3H), 6.79 (d, *J* = 7.2 Hz, 2H), 6.61 (d, *J* = 7.2 Hz, 2H), 4.19 (q, *J* = 7.2 Hz, 2H), 4.01 (t, *J* = 6.5 Hz, 1H), 3.93 (bs, 1H), 3.77 (s, 3H), 2.81 (t, *J* = 7.2 Hz, 2H), 2.22–2.01 (m, 2H), 1.27 (t, *J* = 7.2 Hz, 3H); **^{13}C NMR** (100 MHz, $CDCl_3$) δ 174.3, 152.8, 141.0, 140.9, 128.5, 128.4, 126.1, 115.2, 114.9, 61.0, 57.3, 55.7, 34.8, 31.9, 14.3. All other data are in accordance with that reported in literature.[2f]

ethyl 7-chloro-2-((4-methoxyphenyl)amino)heptanoate (45)

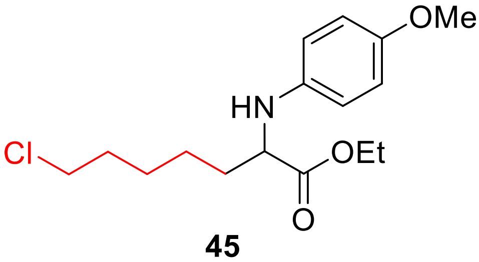

45

Compound **45** was prepared according to the general procedure A and was purified by flash column chromatography (Petroleum ether/EtOAc = 50:1) to afford the title compound as a light-yellow oil with 62% of isolated yield (38.9 mg). **^{1}H NMR** (400 MHz, $CDCl_3$) δ 6.76 (d, *J* = 7.8 Hz, 2H), 6.60 (d, *J* = 7.8 Hz, 2H), 4.15 (q, *J* = 7.4 Hz, 2H), 3.96 (t, *J* = 6.2 Hz, 1H), 3.90 (bs, 1H), 3.73 (s, 3H), 3.53 (t, *J* = 6.4 Hz, 2H), 1.87–1.72 (m, 4H), 1.48–1.45 (m, 4H), 1.23 (t, *J* = 7.2 Hz, 3H); **^{13}C NMR** (100 MHz, $CDCl_3$) δ 174.4, 152.8, 141.0, 115.2, 114.9, 61.0, 57.8, 55.7, 44.9, 33.0, 32.4, 26.6, 25.0, 14.3; **HRMS (ESI)** m/z: calcd. for $C_{16}H_{25}ClNO_3$ $[M+H]^+$ 314.1517; Found 314.1513.

ethyl 2-cyclohexyl-2-((4-methoxyphenyl)amino)acetate (46)[4i]

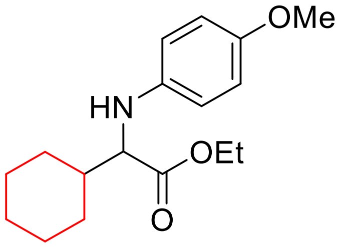

46

Compound **46** was prepared according to the general procedure A and was purified by flash column chromatography (Petroleum ether/EtOAc = 50:1) to afford the title compound as light-yellow oil with 36% of isolated yield (21.0 mg). **^{1}H NMR** (400 MHz, $CDCl_3$) δ 6.78 (d, *J* = 7.0 Hz, 2H), 6.52 (d, *J* = 7.0 Hz, 2H), 4.10–4.04 (m, 2H), 3.79 (bs, 1H), 3.68 (t, *J* = 6.2 Hz, 1H), 3.65 (s, 3H), 1.80–1.58 (m, 6H), 1.25–1.05 (m, 8H); **^{13}C NMR** (100 MHz, $CDCl_3$) δ 173.0, 151.6, 140.6, 114.2, 113.8, 62.4, 59.7, 54.7, 40.3, 28.6, 28.2, 25.2, 25.1, 25.0, 13.3; **HRMS (ESI)** m/z: calcd. for $C_{17}H_{26}NO_3$ $[M+H]^+$ 292.1907; Found 292.1904.

Scaled-up Experiments and Synthesis of Benazepril *tert*-Butyl Ester

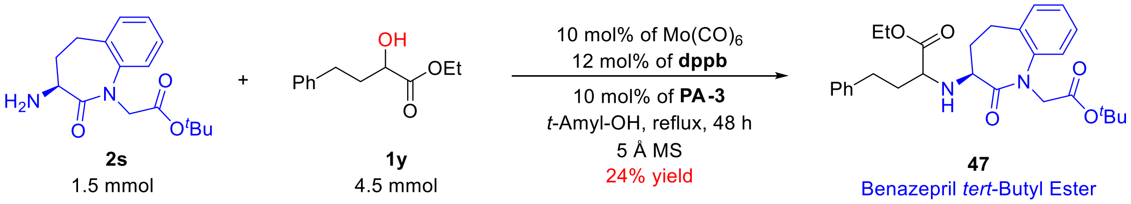

Scaled-up Experiment: To an oven dried two-necked round-bottom flask equipped with a magnetic stir bar, 5Å MS (250-300 mg), $Mo(CO)_6$ (39.6 mg, 10 mol%), **dppb** (76.8 mg, 12 mol%), phosphoric acid **PA-3** (52.2 mg, 10 mol%), α-hydroxy ester **1y** (937.2 mg, 4.5 mmol), and amine (*S*)-**2s** (435.54 mg, 1.5 mmol) were added. Anhydrous *t*-amyl-OH (10 mL) was added and one neck of the flask was then fitted with reflux condenser and the other neck was sealed tightly using screw cap and paraffin film. The flask was then purged with nitrogen through reflux condenser and the resulting mixture was refluxed at 150 ˚C for 48 hours. The reaction mixture was then transferred to a 50 mL round bottom flask and the solvent was removed *in vacuo* with the aid of a rotary evaporator. The obtained residue was purified by flash column chromatography on silica gel to afford pure Benazepril *tert*-Butyl Ester **47** in 24% of isolated yield (173.0 mg).

Ethyl-2-(((*S*)-1-(2-(tert-butoxy)-2-oxoethyl)-2-oxo-2,3,4,5-tetrahydro-1H-benzo[b]azepin-3-yl)amino)-4-phenylbutanoate (47)[5]

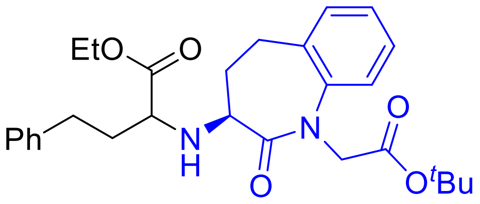

47

Compound **47** was obtained as yellow oil; **¹H NMR** (400 MHz, $CDCl_3$) δ 7.30–7.10 (m, 9H), 4.61 (d, *J* = 17.2 Hz, 1H), 4.32 (d, *J* = 17.2 Hz, 1H), 4.10–4.00 (m, 2H), 3.30–3.22 (m, 3H), 2.77–2.64 (m, 2H), 2.60–2.55 (m, 1H), 2.45–2.34 (m, 1H), 2.22 (bs, 1H), 2.04–1.88 (m, 3H), 1.44 (s, 9H), 1.21 (t, *J* = 6.8 Hz, 3H); **¹³C NMR** (100 MHz, $CDCl_3$) δ 174.0, 173.9, 167.9, 141.7, 141.1, 136.2, 129.4, 128.4, 128.3, 127.7, 126.6, 125.7, 122.1, 81.9, 60.7, 59.3, 56.8, 50.9, 38.0, 34.3, 31.5, 28.5, 28.0, 14.2; **HRMS (ESI)** m/z: calcd. for $C_{28}H_{37}N_2O_5$ $[M+H]^+$ 481.2697; Found 481.2693.

Optimization study for the asymmetric amination of α-hydroxy esters

Table S4. Evaluation of different parameters[a]

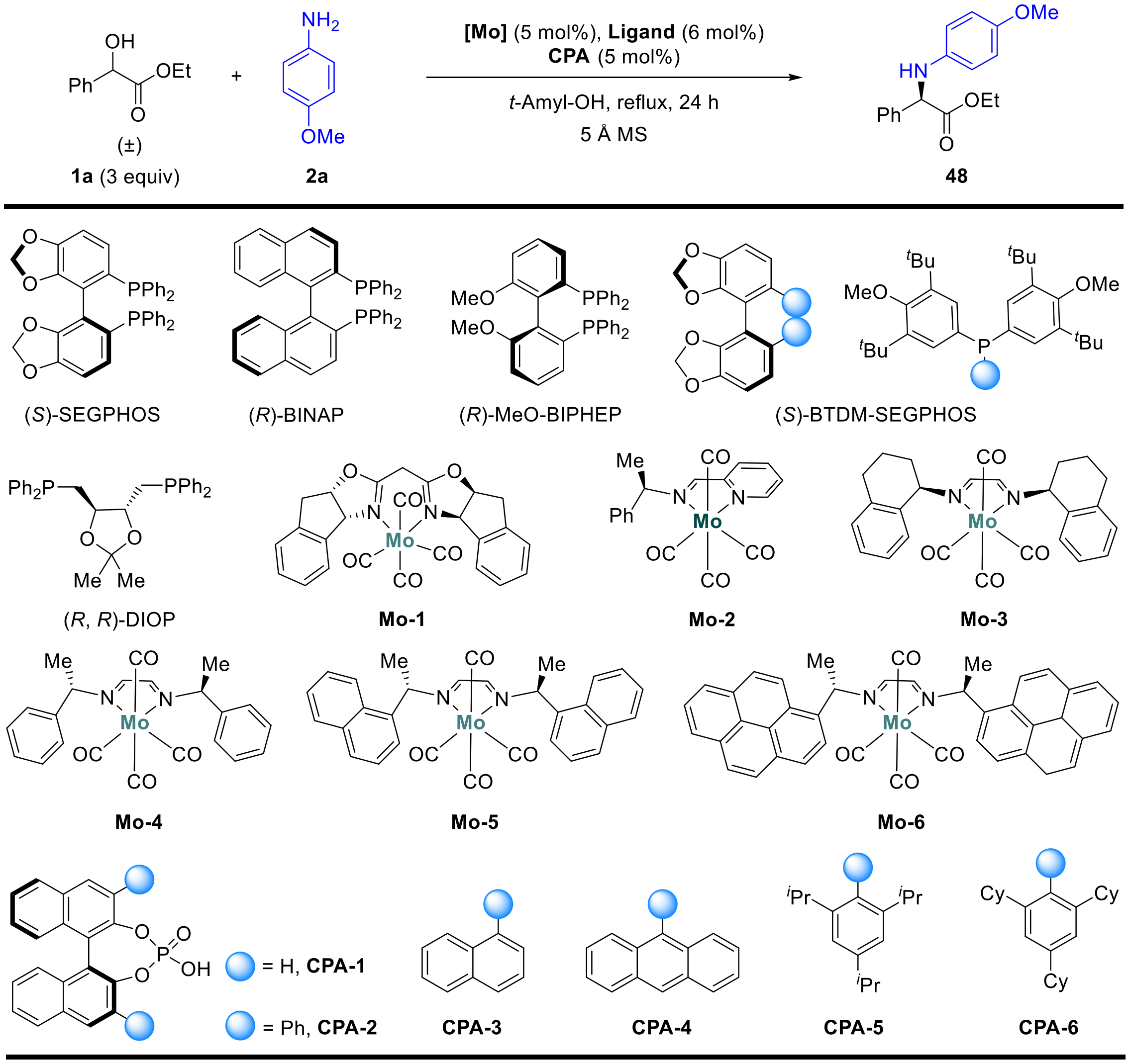

entry	[Mo]	**Ligand**	**CPA**	yield of **3** (%)[b]	ee of **3** (%)[c]
1	$Mo(CO)_6$	(S)-SEGPHOS	CPA-**1**	65	8
2	$Mo(CO)_6$	(*R*)-BINAP	CPA-**1**	52	5
3	$Mo(CO)_6$	(*R*)-MeO-BIPHEP	CPA-**1**	37	2
4	$Mo(CO)_6$	(S)-BTDM-SEGPHOS	CPA-**1**	44	14
5	$Mo(CO)_6$	(*R*, *R*)-DIOP	CPA-**1**	12	0
6	**Mo-1**	--	CPA-**1**	16	15
7	**Mo-2**	--	CPA-**1**	38	3
8	**Mo-3**	--	CPA-**1**	52	22

9	**Mo-4**	--	CPA-**1**	27	17
10	**Mo-5**	--	CPA-**1**	66	27
11	**Mo-6**	--	CPA-**1**	36	24
12	**Mo-5**	--	CPA-**2**	42	30
13	**Mo-5**	--	CPA-**3**	46	67
14	**Mo-5**	--	CPA-**4**	62	53
15	**Mo-5**	--	CPA-**5**	67	72
16	**Mo-5**	--	CPA-**6**	51	70
17	**Mo-6**	--	CPA-**5**	38	59
18	$Mo(CO)_6$	(S)-BTDM-SEGPHOS	CPA-**5**	72	45

[a] Reaction conditions: [**Mo**] (5 mol%), Ligand (6 mol%), **1a** (0.6 mmol, 3 equiv.), *p*-anisidine **2a** (0.2 mmol, 1 equiv.), solvent (2.0 mL), CPA (5 mol%), 5Å MS (80-100 mg), reflux (150 °C), 24 hours. [b] Isolated yields. [c] Determined by HPLC using a Chiral AS-H column.

Table S5. Evaluation of α-hydroxy ester[a]

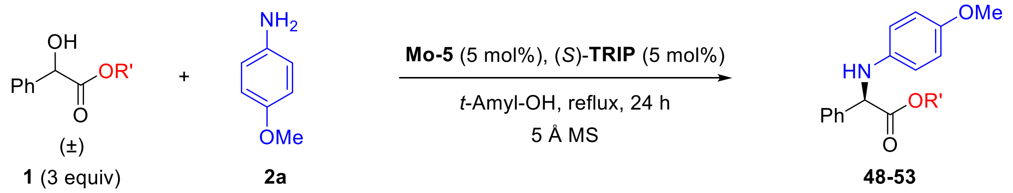

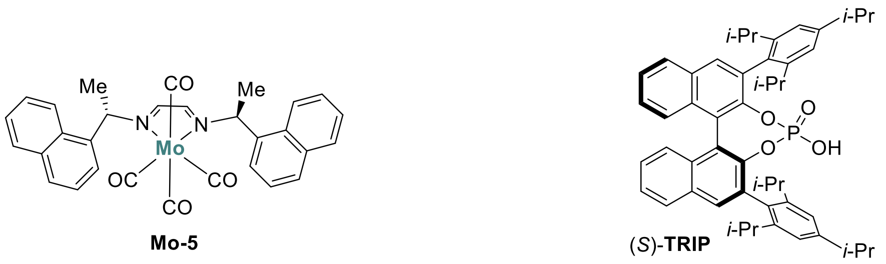

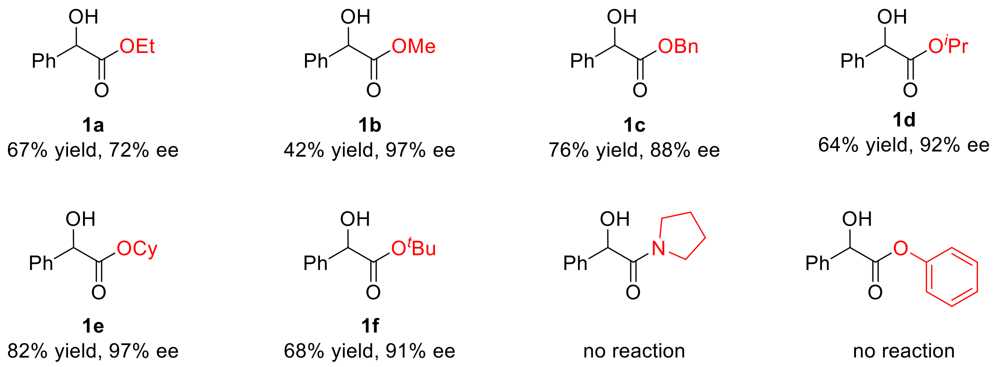

[a] Reaction conditions: [**Mo**] (5 mol%), (*S*)-TRIP (5 mol%), **1** (0.6 mmol, 3 equiv.), *p*-anisidine **2a** (0.2 mmol, 1 equiv.), solvent (2.0 mL), 5Å MS (80-100 mg), reflux (150 °C), 24 hours. [b] Isolated yields. [c] Determined by HPLC using Chiral columns.

General procedure B for molybdenum-catalysed asymmetric amination of α-hydroxy esters.

To an oven dried two-necked round-bottom flask equipped with a magnetic stir bar, 5Å MS (100-150 mg), Mo-**5** complex (5.7 mg, 5 mol%), chiral phosphoric acid (*S*)-TRIP (7.5 mg, 5 mol%), α-hydroxy ester **1a** (108.12 mg, 0.6 mmol), and *p*-anisidine **2a** (24.63 mg, 0.2 mmol) were added. Anhydrous *t*-amyl-OH (2 mL) was added and one neck of the flask was then fitted with reflux condenser and the other neck was sealed tightly using screw cap and paraffin film. The flask was then purged with nitrogen through reflux condenser and the resulting mixture was refluxed at 150 ˚C for 24 hours. The reaction mixture was then transferred to a 50 mL round bottom flask and the solvent was removed *in vacuo* with the aid of a rotary evaporator. The obtained residue was purified by flash column chromatography on silica gel to afford pure α-amino ester **48**. Other products (**49**–**71**) were prepared by following the same procedure.

ethyl (*R*)-2-((4-methoxyphenyl)amino)-2-phenylacetate (48)[6a]

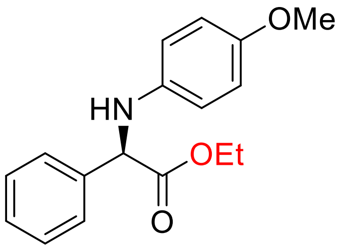

48

Compound **48** was prepared according to general **procedure B** and was obtained as yellow oil (38.2 mg, 67% yield, 72% ee). **$[\alpha]^{25}_{D}$** = –39.9 (*c* = 0.4, $CHCl_3$). Lit. data:[6a] $[\alpha]^{22}_{D}$ = +32.5 (*c* = 1.0, $CHCl_3$); **^{1}H NMR** (400 MHz, $CDCl_3$) δ 7.49 (d, *J* = 7.2 Hz, 2H), 7.37–7.29 (m, 3H), 6.72 (d, *J* = 7.6 Hz, 2H), 6.53 (d, *J* = 7.6 Hz, 2H), 5.00 (s, 1H), 4.67 (bs, 1H), 4.26–4.09 (m, 2H), 3.70 (s, 3H), 1.20 (t, *J* = 6.2 Hz, 3H); **^{13}C NMR** (100 MHz, $CDCl_3$) δ 172.0, 152.4, 140.2, 137.9, 128.8, 128.1, 127.2, 114.8, 114.7, 61.7, 55.7, 14.0; **HRMS (ESI)** m/z: calcd. for $C_{17}H_{20}NO_3$ $[M+H]^+$ 286.1438; Found 286.1436; **HPLC conditions:** Chiralcel AS-H column, 254 nm, flow rate: 1 ml/min, *i*-PrOH/hexanes = 5/95, t_{minor} = 10.1 min, t_{major} = 11.4 min; 72% ee.

methyl (*R*)-2-((4-methoxyphenyl)amino)-2-phenylacetate (49)[6a]

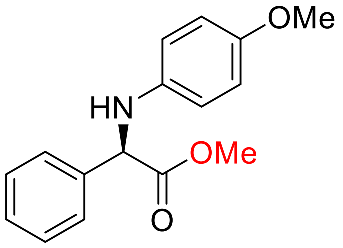

49

Compound **49** was prepared through general **procedure B** and was obtained as white solid (22.8 mg, 42% yield, 97% ee). **$[\alpha]^{25}_D$** = +48.9 (c = 1.1, $CHCl_3$). Lit. data:[6a] $[\alpha]^{22}_D$ = +56.8 (c = 1.0, $CHCl_3$); **^{1}H NMR** (400 MHz, $CDCl_3$) δ 7.49 (d, J = 7.2 Hz, 2H), 7.36–7.29 (m, 3H), 6.71 (d, J = 7.6 Hz, 2H), 6.53 (d, J = 7.6 Hz, 2H), 5.01 (s, 1H), 4.68 (bs, 1H), 3.70 (s, 3H), 3.69 (s, 3H); **^{13}C NMR** (100 MHz, $CDCl_3$) δ 172.5, 152.4, 140.1, 137.7, 128.8, 128.2, 127.2, 114.8, 114.7, 61.6, 55.6, 52.6; **HRMS (ESI)** m/z: calcd. for $C_{16}H_{18}NO_3$ $[M+H]^+$ 272.1281; Found 272.1275; **HPLC conditions:** Chiralcel IC-H column, 254 nm, flow rate: 1 ml/min, *i*-PrOH/hexanes = 5/95, t_{minor} = 14.2 min, t_{major} = 16.4 min; 97% ee.

benzyl (*R*)-2-((4-methoxyphenyl)amino)-2-phenylacetate (50)[6a]

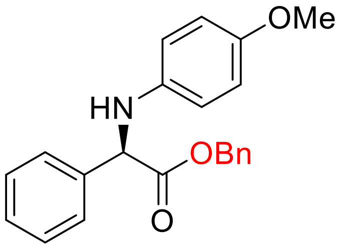

50

Compound **50** was prepared through general **procedure B** and was obtained as white solid (52.8 mg, 76% yield, 88% ee). **$[\alpha]^{25}_D$** = –14.7 (c = 1.3, $CHCl_3$). Lit. data:[6a] $[\alpha]^{22}_D$ = +35.0 (c = 1.0, $CHCl_3$); ^{1}H NMR (400 MHz, $CDCl_3$) δ 7.47 (d, J = 6.0 Hz, 2H), 7.35–7.27 (m, 6H), 7.16 (d, J = 6.2 Hz, 2H), 6.70 (d, J = 6.4 Hz, 2H), 6.52 (d, J = 6.4 Hz, 2H), 5.20–5.05 (m, 3H), 4.67 (bs, 1H), 3.68 (s, 3H); **^{13}C NMR** (100 MHz, $CDCl_3$) δ 171.9, 152.5, 140.1, 137.6, 135.3, 128.8, 128.5, 127.8, 127.3, 114.8, 114.7, 67.2, 61.7, 55.6; **HRMS (ESI)** m/z: calcd. for $C_{22}H_{22}NO_3$ $[M+H]^+$ 348.1594; Found 348.1592; **HPLC conditions:** Chiralcel IC-H column, 254 nm, flow rate: 1 ml/min, *i*-PrOH/hexanes = 5/95, t_{major} = 13.7 min, t_{minor} = 18.2 min; 88% ee.

isopropyl (*R*)-2-((4-methoxyphenyl)amino)-2-phenylacetate (51)[6a]

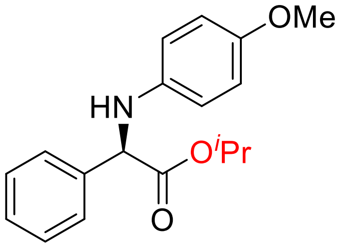

51

Compound **51** was prepared through general **procedure B** and was obtained as light-yellow oil (38.3 mg, 64% yield, 92% ee). **$[\alpha]^{25}_D$** = +67.2 (c = 0.6, $CHCl_3$). Lit. data:[6a] $[\alpha]^{22}_D$ = +66.0 (c = 1.0, $CHCl_3$); **^{1}H NMR** (400 MHz, $CDCl_3$) δ 7.49 (d, J = 6.4 Hz, 2H), 7.36–7.25 (m, 3H), 6.71 (d, J = 6.8 Hz, 2H), 6.54 (d, J = 6.8 Hz, 2H), 5.07–5.00 (m, 1H), 4.97 (s, 1H), 4.70 (bs, 1H),

3.70 (s, 3H), 1.26 (d, *J* = 4.8 Hz, 3H), 1.08 (d, *J* = 4.8 Hz, 3H); **^{13}C NMR** (100 MHz, $CDCl_3$) δ 171.5, 152.4, 140.3, 137.9, 128.7, 128.0, 127.1, 114.8, 114.7, 69.3, 61.7, 55.7, 21.7, 21.3; **HRMS (ESI)** m/z: calcd. for $C_{18}H_{22}NO_3$ $[M+H]^+$ 300.1594; Found 300.1592; **HPLC conditions:** Chiralcel AS-H column, 254 nm, flow rate: 1 ml/min, *i*-PrOH/hexanes = 5/95, t_{minor} = 7.2 min, t_{major} = 8.5 min; 92% ee.

cyclohexyl (*R*)-2-((4-methoxyphenyl)amino)-2-phenylacetate (52)

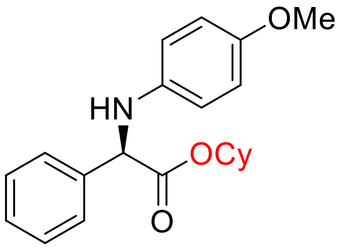

52

Compound **52** was prepared through general **procedure B** and was obtained as white solid (55.6 mg, 82% yield, 97% ee). **$[\alpha]^{25}_D$** = –66.3 (*c* = 1.4, $CHCl_3$); **^{1}H NMR** (400 MHz, $CDCl_3$) δ 7.53 (d, *J* = 6.7 Hz, 2H), 7.39–7.28 (m, 3H), 6.75 (d, *J* = 7.0 Hz, 2H), 6.57 (d, *J* = 7.0 Hz, 2H), 5.03 (s, 1H), 4.87–4.81 (m, 1H), 4.75 (bs, 1H), 3.74 (s, 3H), 1.89–1.84 (m, 1H), 1.75–1.71 (m, 1H), 1.65–1.48 (m, 4H), 1.41–1.25 (m, 4H); **^{13}C NMR** (100 MHz, $CDCl_3$) δ 171.4, 152.3, 140.3, 138.1, 128.6, 128.0, 127.1, 114.8, 114.6, 739, 61.7, 55.6, 31.4, 30.9, 25.2, 23.3, 23.1; **HRMS (ESI)** m/z: calcd. for $C_{21}H_{26}NO_3$ $[M+H]^+$ 340.1907; Found 340.1904; **HPLC conditions:** Chiralcel AS-H column, 254 nm, flow rate: 1 ml/min, *i*-PrOH/hexanes = 5/95, t_{minor} = 10.25 min, t_{major} = 12.93 min; 97% ee.

tert-butyl (*R*)-2-((4-methoxyphenyl)amino)-2-phenylacetate (**53**)[6a]

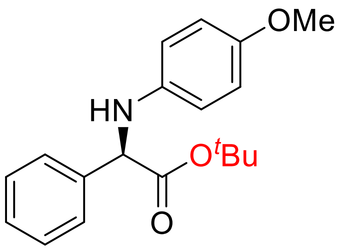

53

Compound **53** was prepared through general **procedure B** and was obtained as white solid (42.6 mg, 68% yield, 91% ee). **$[\alpha]^{25}_D$** = –57.6 (*c* = 0.45, $CHCl_3$). Lit. data: $[\alpha]^{22}_D$ = +58.2 (*c* = 1.0, $CHCl_3$); **^{1}H NMR** (400 MHz, $CDCl_3$) δ 7.47 (d, *J* = 6.4 Hz, 2H), 7.35–7.24 (m, 3H), 6.71 (d, *J* = 7.0 Hz, 2H), 6.52 (d, *J* = 7.0 Hz, 2H), 4.89 (s, 1H), 4.66 (bs, 1H), 3.70 (s, 3H), 1.37 (s, 9H); **^{13}C NMR** (100 MHz, $CDCl_3$) δ 171.1, 152.2, 140.4, 138.4, 128.6, 127.9, 127.1, 114.8, 114.6,

82.2, 62.1, 55.7, 27.8; **HRMS (ESI)** m/z: calcd. for $C_{19}H_{24}NO_3$ $[M+H]^+$ 314.1751; Found 314.1753; **HPLC conditions:** Chiralcel AS-H column, 254 nm, flow rate: 1 ml/min, *i*-PrOH/hexanes = 5/95, t_{minor} = 8.52 min, t_{major} = 10.47 min; 91% ee.

methyl (*R*)-2-((4-methoxyphenyl)amino)-2-(naphthalen-2-yl)acetate (**54**)[6b]

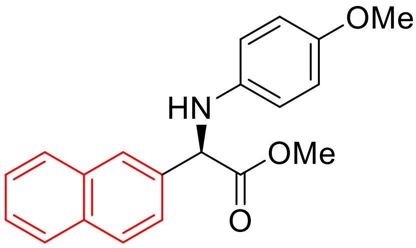

54

Compound **54** was prepared through general **procedure B** and was obtained as light-yellow solid (29.5 mg, 46% yield, 83% ee). $[\alpha]^{25}_D$ = +30.9 (*c* = 0.9, $CHCl_3$). Lit. data:[6b] $[\alpha]^{20}_D$ = +130.6 (*c* = 1.6, $CHCl_3$); **^{1}H NMR** (400 MHz, $CDCl_3$) δ 7.95 (s, 1H), 7.83–7.80 (m, 3H), 7.61 (dd, *J* = 2.0, 8.8 Hz, 1H), 7.48–7.43 (m, 2H), 6.70 (d, *J* = 8.8 Hz, 2H), 6.54 (d, *J* = 8.8 Hz, 2H), 5.17 (s, 1H), 4.76 (bs, 1H), 3.71 (s, 3H), 3.68 (s, 3H); **^{13}C NMR** (100 MHz, $CDCl_3$) δ 172.5, 152.6, 140.2, 135.4, 133.4, 133.3, 128.8, 128.1, 127.8, 126.5, 126.4, 126.3, 125.0, 114.9, 114.8, 61.9, 55.7, 52.9; **HRMS (ESI)** m/z: calcd. for $C_{20}H_{20}NO_3$ $[M+H]^+$ 322.1438; Found 322.1432; **HPLC conditions:** Chiralcel AS-H column, 254 nm, flow rate: 1 ml/min, *i*-PrOH/hexanes = 5/95, t_{minor} = 12.76 min, t_{major} = 15.11 min; 83% ee.

cyclohexyl (*R*)-2-((4-methoxyphenyl)amino)-2-(naphthalen-2-yl)acetate (55)

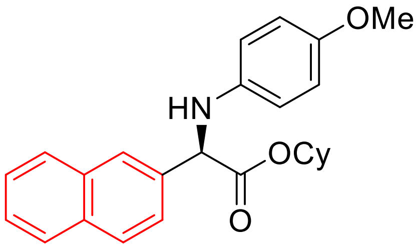

55

Compound **55** was prepared through general **procedure B** and was obtained as light-yellow oil (53.7 mg, 69% yield, 95% ee). $[\alpha]^{25}_D$ = +61.8 (*c* = 0.92, $CHCl_3$); **^{1}H NMR** (400 MHz, $CDCl_3$) δ 7.96 (s, 1H), 7.83–7.80 (m, 3H), 7.61 (d, *J* = 9.2 Hz, 1H), 7.47–7.45 (m, 2H), 6.71 (d, *J* = 8.4 Hz, 2H), 6.57 (d, *J* = 8.4 Hz, 2H), 5.15 (s, 1H), 4.97 (bs, 1H), 4.81 (s, 1H), 3.68 (s, 3H), 1.85–1.12 (m, 10H); **^{13}C NMR** (100 MHz, $CDCl_3$) δ 171.3, 152.4, 140.3, 135.6, 133.3, 133.1, 128.4, 128.0, 127.6, 126.2, 126.1, 126.0, 125.0, 114.8, 114.7, 74.1, 61.9, 55.7, 31.4, 31.0, 25.2, 23.4, 23.2; **HRMS (ESI)** m/z: calcd. for $C_{25}H_{28}NO_3$ $[M+H]^+$ 390.2064; Found 390.2062; **HPLC**

conditions: Chiralcel AS-H column, 254 nm, flow rate: 1 ml/min, *i*-PrOH/hexanes = 5/95, t_{major} = 10.12 min, t_{minor} = 12.73 min; 95% ee.

methyl (*R*)-2-((4-methoxyphenyl)amino)-2-(thiophen-2-yl)acetate (56)

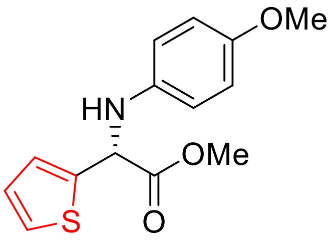

56

Compound **56** was prepared through general **procedure A** and was obtained as light-yellow oil (23.2 mg, 42% yield, 32% ee). $[\alpha]^{25}_{D}$ = –15.9 (*c* = 0.75, $CHCl_3$); **^{1}H NMR** (400 MHz, $CDCl_3$) δ 7.24 (s, 1H), 7.12 (s, 1H), 6.98–6.95 (m, 1H), 6.75 (d, *J* = 7.6 Hz, 2H), 6.61 (d, *J* = 7.6 Hz, 2H), 5.28 (s, 1H), 4.66 (bs, 1H), 3.77 (s, 3H), 3.71 (s, 3H); **^{13}C NMR** (100 MHz, $CDCl_3$) δ 171.6, 152.9, 141.4, 139.9, 127.0, 125.6, 125.5, 115.2, 114.8, 57.8, 55.6, 52.9; **HRMS (ESI)** m/z: calcd. for $C_{14}H_{16}NO_3S$ $[M+H]^+$ 278.0845; Found 278.0843; **HPLC conditions:** Chiralcel AS-H column, 254 nm, flow rate: 1 ml/min, *i*-PrOH/hexanes = 5/95, t_{major} = 17.78 min, t_{minor} = 21.34 min; 29% ee.

cyclohexyl (*R*)-2-((4-methoxyphenyl)amino)-2-(thiophen-2-yl)acetate (57)

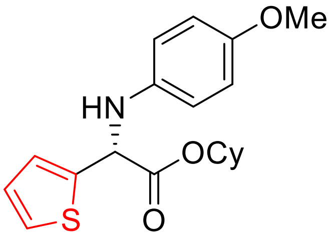

57

Compound **57** was prepared through general **procedure A** and was obtained as light-yellow oil (46.2 mg, 67% yield, 93% ee). $[\alpha]^{25}_{D}$ = +36.8 (*c* = 0.3, $CHCl_3$); **^{1}H NMR** (400 MHz, $CDCl_3$) δ 7.23 (s, 1H), 7.13 (s, 1H), 6.99–6.95 (m, 1H), 6.75 (d, *J* = 7.6 Hz, 2H), 6.62 (d, *J* = 7.6 Hz, 2H), 5.24 (s, 1H), 4.87–4.82 (m, 1H), 3.66 (bs, 1H), 3.72 (s, 3H), 1.83–1.26 (m, 10H); **^{13}C NMR** (100 MHz, $CDCl_3$) δ 170.5, 152.9, 141.9, 140.1, 126.9, 125.4, 125.2, 115.2, 114.8, 74.4, 58.1, 55.7, 31.4, 31.0, 25.2, 23.4, 23.3; **HRMS (ESI)** m/z: calcd. for $C_{19}H_{24}NO_3S$ $[M+H]^+$ 346.1471; Found 346.1467; **HPLC conditions:** Chiralcel AS-H column, 254 nm, flow rate: 1 ml/min, *i*-PrOH/hexanes = 5/95, t_{major} = 10.02 min, t_{minor} = 14.20 min; 93% ee.

methyl (*S*)-2-((4-methoxyphenyl)amino)propanoate (58)[6c]

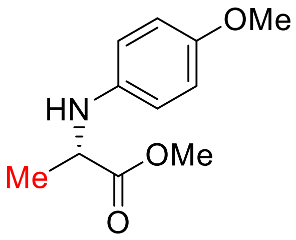

58

Compound **59** was prepared through general **procedure A** and was obtained as light-yellow oil (16.3 mg, 39% yield, 70% ee). **[α]$^{25}_{D}$** = –29.2 (*c* = 0.34, $CHCl_3$). Lit. data:[6c] [α]$^{25}_{D}$ = +55.5 (*c* = 0.65, $CHCl_3$); **^{1}H NMR** (400 MHz, $CDCl_3$) δ 6.77 (d, *J* = 8.4 Hz, 2H), 6.59 (d, *J* = 8.4 Hz, 2H), 4.11–4.05 (m, 1H), 3.86 (bs, 1H), 3.74 (s, 3H), 3.71 (s, 3H), 1.46 (d, *J* = 6.8 Hz, 3H); **^{13}C NMR** (100 MHz, $CDCl_3$) δ 175.4, 152.8, 140.6, 115.0, 114.9, 55.7, 53.0, 52.1, 19.1; **HRMS (ESI)** m/z: calcd. for $C_{11}H_{16}NO_3$ [M+H]$^+$ 210.1125; Found 210.1122; **HPLC conditions:** Chiralcel AS-H column, 254 nm, flow rate: 1 ml/min, *i*-PrOH/hexanes = 5/95, t_{minor} = 23.12 min, t_{major} = 27.56 min; 70% ee.

cyclohexyl (*S*)-2-((4-methoxyphenyl)amino)propanoate (59)

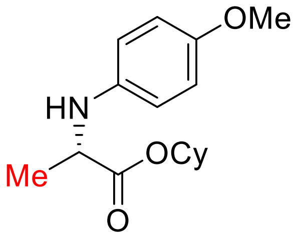

59

Compound **59** was prepared through general **procedure A** and was obtained as light-yellow oil (25.5 mg, 46% yield, 80% ee). **[α]$^{25}_{D}$** = –43.4 (*c* = 0.86, $CHCl_3$); **^{1}H NMR** (400 MHz, $CDCl_3$) δ 6.76 (d, *J* = 7.8 Hz, 2H), 6.59 (d, *J* = 7.8 Hz, 2H), 4.80–4.75 (m, 1H), 4.06–4.01 (m, 1H), 3.89 (bs, 1H), 3.73 (s, 3H), 1.81–1.63 (m, 4H), 1.54–1.26 (m, 9H); **^{13}C NMR** (100 MHz, $CDCl_3$) δ 174.3, 152.6, 140.8, 115.0, 114.8, 73.2, 55.6, 53.2, 31.5, 31.3, 25.3, 23.5, 23.3, 19.0; **HRMS (ESI)** m/z: calcd. for $C_{16}H_{24}NO_3$ [M+H]$^+$ 278.1751; Found 278.1748; **HPLC conditions:** Chiralcel AD-H column, 254 nm, flow rate: 1 ml/min, *i*-PrOH/hexanes = 5/95, t_{minor} = 8.05 min, t_{major} = 9.54 min; 80% ee.

ethyl (*S*)-2-((4-methoxyphenyl)amino)-4-phenylbutanoate (60)[6d]

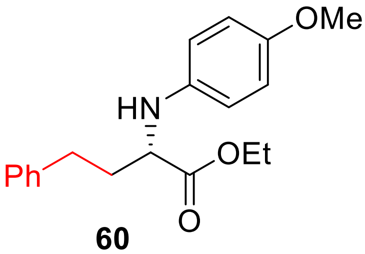

60

Compound **60** was prepared through general **procedure A** and was obtained as light-yellow oil (29.4 mg, 47% yield, 45% ee). **[α]$^{25}_{D}$** = +9.4 (c = 0.27, $CHCl_3$). Lit. data:[6d] [α]$^{20}_{D}$ = +35.2 (c = 0.027, acetone); **^{1}H NMR** (400 MHz, $CDCl_3$) δ 7.30–7.26 (m, 2H), 7.22–7.18 (m, 3H), 6.75 (d, J = 7.0 Hz, 2H), 6.57 (d, J = 7.0 Hz, 2H), 4.18–4.12 (m, 2H), 3.99–3.95 (m, 1H), 3.89 (bs, 1H), 3.73 (s, 3H), 2.77 (t, J = 7.2 Hz, 2H), 2.18–1.98 (m, 2H), 1.23 (t, J = 7.2 Hz, 3H); **^{13}C NMR** (100 MHz, $CDCl_3$) δ 174.3, 152.7, 141.0, 140.9, 128.5, 128.4, 126.1, 115.2, 114.8, 61.0, 57.3, 55.7, 34.7, 31.8, 14.2; **HRMS (ESI)** m/z: calcd. for $C_{19}H_{24}NO_3$ $[M+H]^+$ 314.1751; Found 314.1753; **HPLC conditions:** Chiralcel OJ-H column, 254 nm, flow rate: 1 ml/min, *i*-PrOH/hexanes = 10/90, t_{major} = 21.4 min, t_{minor} = 28.87 min; 45% ee.

cyclohexyl (S)-2-((4-methoxyphenyl)amino)-4-phenylbutanoate (61)

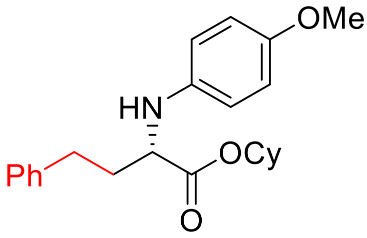

61

Compound **61** was prepared through general **procedure A** and was obtained as light-yellow oil (46.3 mg, 63% yield, 67% ee). **[α]$^{25}_{D}$** = +12.1 (c = 1.2, $CHCl_3$); **^{1}H NMR** (400 MHz, $CDCl_3$) δ 7.28–7.25 (m, 2H), 7.20–7.18 (m, 3H), 6.75 (d, J = 8.0 Hz, 2H), 6.58 (d, J = 8.0 Hz, 2H), 4.82–4.77 (m, 1H), 3.98–3.95 (m, 1H), 3.77 (bs, 1H), 3.72 (s, 3H), 2.76 (t, J = 7.2 Hz, 2H), 2.17–1.97 (m, 2H), 1.81–1.67 (m, 4H), 1.53–1.21 (m, 6H); **^{13}C NMR** (100 MHz, $CDCl_3$) δ 173.7, 152.7, 141.1, 141.0, 128.5, 128.4, 126.1, 115.2, 114.8, 73.4, 57.5, 55.7, 34.9, 31.9, 31.6, 31.5, 25.3, 23.7, 23.6; **HRMS (ESI)** m/z: calcd. for $C_{23}H_{30}NO_3$ $[M+H]^+$ 368.2220; Found 368.2217; **HPLC conditions:** Chiralcel OJ-H column, 254 nm, flow rate: 1 ml/min, *i*-PrOH/hexanes = 10/90, t_{minor} = 16.52 min, t_{major} = 22.42 min; 67% ee.

ethyl (*S*)-7-chloro-2-((4-methoxyphenyl)amino)heptanoate (62)

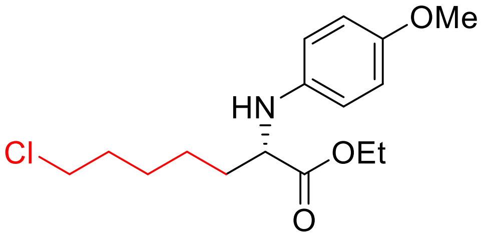

62

Compound **62** was prepared through general **procedure A** and was obtained as light-yellow oil (32.0 mg, 51% yield, 54% ee). **[α]$^{25}_{D}$** = +15.6 (c = 0.64, $CHCl_3$); **^{1}H NMR** (400 MHz, $CDCl_3$) δ 6.76 (d, J = 8.4 Hz, 2H), 6.60 (d, J = 8.4 Hz, 2H), 4.19–4.13 (m, 2H), 3.98–3.94 (m, 1H), 3.73

(s, 3H), 3.53 (t, J = 7.2 Hz, 2H), 1.86–1.71 (m, 4H), 1.52–1.43 (m, 4H), 1.23 (t, J = 7.2 Hz, 3H); **^{13}C NMR** (100 MHz, $CDCl_3$) δ 174.4, 152.7, 141.0, 115.1, 114.8, 60.9, 57.7, 55.7, 44.9, 33.0, 32.3, 26.6, 24.9, 14.2; **HRMS (ESI)** m/z: calcd. for $C_{16}H_{25}ClNO_3$ $[M+H]^+$ 314.1517; Found 314.1514; **HPLC conditions:** Chiralcel OJ-H column, 254 nm, flow rate: 1 ml/min, *i*-PrOH/hexanes = 10/90, t_{minor} = 7.50 min, t_{major} = 9.25 min; 54% ee.

cyclohexyl (*S*)-7-chloro-2-((4-methoxyphenyl)amino)heptanoate (63)

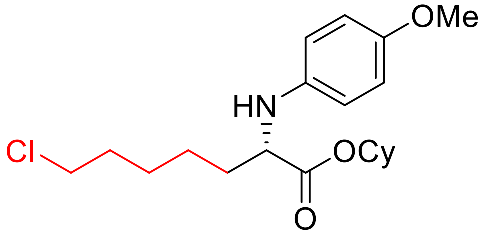

63

Compound **63** was prepared through general **procedure A** and was obtained as light-yellow oil (58.1 mg, 79% yield, 82% ee). **$[\alpha]^{25}_{D}$** = +18.9 (c = 0.71, $CHCl_3$); **^{1}H NMR** (400 MHz, $CDCl_3$) δ 6.76 (d, J = 7.8 Hz, 2H), 6.60 (d, J = 7.8 Hz, 2H), 4.78 (s, 1H), 3.95 (t, J = 6.7 Hz, 1H), 3.85 (bs, 1H), 3.73 (s, 3H), 3.53 (t, J = 6.8 Hz, 2H), 1.81–1.63 (m, 8H), 1.53–1.26 (m, 10H); **^{13}C NMR** (100 MHz, $CDCl_3$) δ 173.8, 152.7, 141.1, 115.1, 114.8, 73.2, 57.8, 55.7, 44.9, 33.0, 32.4, 31.6, 31.5, 26.6, 25.3, 24.9, 23.6, 23.5; **HRMS (ESI)** m/z: calcd. for $C_{20}H_{31}ClNO_3$ $[M+H]^+$ 368.1987; Found 368.1985; **HPLC conditions:** Chiralcel OJ-H column, 254 nm, flow rate: 1 ml/min, *i*-PrOH/hexanes = 10/90, t_{minor} = 8.43 min, t_{major} = 11.17 min; 82% ee.

ethyl (*S*)-2-cyclohexyl-2-((4-methoxyphenyl)amino)acetate (64)[6e]

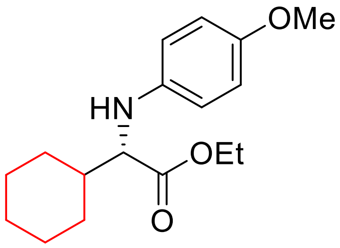

64

Compound **64** was prepared through general **procedure A** and was obtained as light-yellow oil (14.0 mg, 24% yield, 32% ee). **$[\alpha]^{25}_{D}$** = +15.3 (c = 0.46, $CHCl_3$); **^{1}H NMR** (400 MHz, $CDCl_3$) δ 6.78 (d, J = 8.2 Hz, 2H), 6.62 (d, J = 8.2 Hz, 2H), 4.20–4.14 (m, 2H), 3.89 (bs, 1H), 3.79 (s, 1H), 3.75 (s, 3H), 1.90–1.68 (m, 6H), 1.35–1.15 (m, 8H); **^{13}C NMR** (100 MHz, $CDCl_3$) δ 174.0, 152.6, 141.7, 115.2, 114.8, 63.4, 60.7, 55.7, 41.3, 29.7, 29.2, 26.2, 26.1, 26.0, 14.3; **HRMS (ESI)** m/z: calcd. for $C_{17}H_{26}NO_3$ $[M+H]^+$ 292.1907; Found 292.1904; **HPLC conditions:** Chiralcel AS-H column, 254 nm, flow rate: 1 ml/min, *i*-PrOH/hexanes = 5/95, t_{minor} = 5.76 min, t_{major} = 6.72 min; 32% ee.

cyclohexyl (*R*)-2-phenyl-2-(phenylamino)acetate (65)

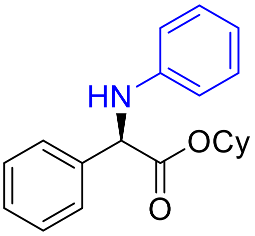

65

Compound **65** was prepared through general **procedure A** and was obtained as a white solid (39.6 mg, 64% yield, 95% ee). **[α]^{25}$_{D}$** = +61.5 (*c* = 0.30, $CHCl_3$); **^{1}H NMR** (400 MHz, $CDCl_3$) δ 7.50 (d, *J* = 8.2 Hz, 2H), 7.36-7.28 (m, 3H), 7.11 (t, *J* = 8.0 Hz, 2H), 6.69 (t, *J* = 8.0 Hz, 1H), 6.56 (d, *J* = 8.2 Hz, 2H), 5.04 (s, 1H), 4.98 (bs, 1H), 4.84–4.78 (m, 1H), 1.85–1.22 (m, 10H); **^{13}C NMR** (100 MHz, $CDCl_3$) δ 171.2, 146.1, 138.0, 129.2, 128.7, 128.1, 127.1, 117.9, 113.4, 74.0, 60.9, 31.4, 30.9, 25.3, 23.4, 23.1; **HRMS (ESI)** m/z: calcd. for $C_{20}H_{24}NO_2$ $[M+H]^+$ 310.1802; Found 310.1801; **HPLC conditions:** Chiralcel AS-H column, 254 nm, flow rate: 1 ml/min, *i*-PrOH/hexanes = 5/95, t_{minor} = 6.32 min, t_{major} = 7.64 min; 95% ee.

cyclohexyl (*R*)-2-phenyl-2-(*p*-tolylamino)acetate (66)

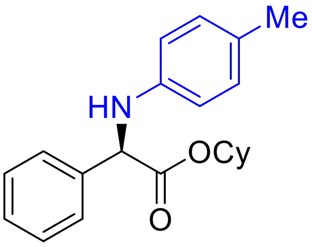

66

Compound **66** was prepared through general **procedure A** and was obtained as a white solid (49.2 mg, 76% yield, 90% ee). **[α]^{25}$_{D}$** = +64.8 (*c* = 0.50, $CHCl_3$); **^{1}H NMR** (400 MHz, $CDCl_3$) δ 7.49 (d, *J* = 7.6 Hz, 2H), 7.35–7.24 (m, 3H), 6.92 (d, *J* = 7.8 Hz, 2H), 6.48 (d, *J* = 7.8 Hz, 2H), 5.04 (s, 1H), 4.84–4.78 (m, 2H), 2.19 (s, 3H), 1.84–1.24 (m, 10H); **^{13}C NMR** (100 MHz, $CDCl_3$) δ 171.4, 143.8, 138.1, 129.7, 128.7, 128.0, 127.2, 127.1, 113.5, 74.0, 61.2, 31.4, 30.9, 25.3, 23.4, 23.2, 20.4; **HRMS (ESI)** m/z: calcd. for $C_{21}H_{26}NO_2$ $[M+H]^+$ 324.1958; Found 324.1951; **HPLC conditions:** Chiralcel AS-H column, 254 nm, flow rate: 1 ml/min, *i*-PrOH/hexanes = 5/95, t_{major} = 6.93 min, t_{minor} = 8.61 min; 90% ee.

cyclohexyl (*R*)-2-((4-bromophenyl)amino)-2-phenylacetate (67)

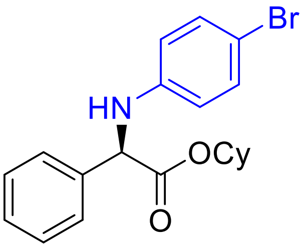

67

Compound **67** was prepared through general **procedure A** and was obtained as a white solid (45.0 mg, 58% yield, 90% ee). $[\alpha]^{25}_{D}$ = +65.7 (*c* = 0.25, $CHCl_3$); **^{1}H NMR** (400 MHz, $CDCl_3$) δ 7.46 (d, *J* = 6.8 Hz, 2H), 7.36–7.31 (m, 3H), 7.18 (d, *J* = 8.4 Hz, 2H), 6.43 (d, *J* = 8.4 Hz, 2H), 5.03 (s, 1H), 4.99 (bs, 1H), 4.83–4.78 (m, 1H), 1.83–1.25 (m, 10H); **^{13}C NMR** (100 MHz, $CDCl_3$) δ 170.9, 144.9, 137.4, 131.9, 128.8, 128.3, 127.1, 115.0, 109.6, 74.3, 60.7, 31.4, 30.9, 25.2, 23.3, 23.1; **HRMS (ESI)** m/z: calcd. for $C_{20}H_{23}BrNO_2$ $[M+H]^+$ 388.0907; Found 388.0901; **HPLC conditions:** Chiralcel AS-H column, 254 nm, flow rate: 1 ml/min, *i*-PrOH/hexanes = 5/95, t_{major} = 8.58 min, t_{minor} = 10.83 min; 90% ee.

butyl (*R*)-4-((2-(cyclohexyloxy)-2-oxo-1-phenylethyl)amino)benzoate (68)

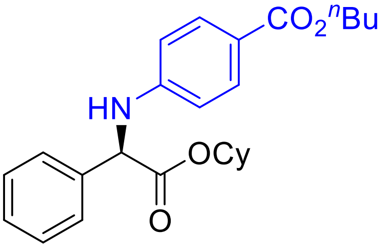

68

Compound **68** was prepared through general **procedure A** and was obtained as a white solid (58.9 mg, 72% yield, 95% ee). $[\alpha]^{25}_{D}$ = +83.4 (*c* = 0.30, $CHCl_3$); **^{1}H NMR** (400 MHz, $CDCl_3$) δ 7.81 (d, *J* = 7.8 Hz, 2H), 7.47 (d, *J* = 7.2 Hz, 2H), 7.36–7.30 (m, 3H), 6.52 (d, *J* = 7.8 Hz, 2H), 5.45 (s, 1H), 5.10 (bs, 1H), 4.82 (s, 1H), 4.23 (t, *J* = 6.4 Hz, 2H), 1.84–1.65 (m, 4H), 1.59–1.26 (m, 10H), 0.95 (t, *J* = 6.9 Hz, 3H); **^{13}C NMR** (100 MHz, $CDCl_3$) δ 170.6, 166.8, 149.5, 137.1, 131.4, 128.8, 128.3, 127.0, 119.5, 112.3, 74.4, 64.1, 60.2, 31.4, 30.9, 30.8, 25.2, 23.3, 23.1, 19.3, 13.8; **HRMS (ESI)** m/z: calcd. for $C_{25}H_{32}NO_4$ $[M+H]^+$ 410.2326; Found 410.2328; **HPLC conditions:** Chiralcel IC-H column, 254 nm, flow rate: 1 ml/min, *i*-PrOH/hexanes = 5/95, t_{minor} = 5.60 min, t_{major} = 6.03 min; 95% ee.

cyclohexyl (*R*)-2-phenyl-2-((3,4,5-trimethoxyphenyl)amino)acetate (69)

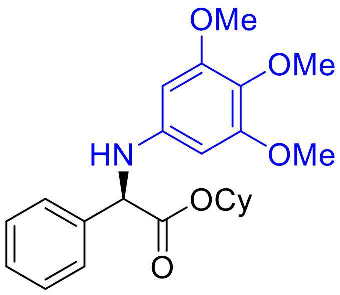

69

Compound **69** was prepared through general **procedure A** and was obtained as a white solid (63.1 mg, 79% yield, 96% ee). **[α]$^{25}_{D}$** = +30.1 (*c* = 0.23, $CHCl_3$); **^{1}H NMR** (400 MHz, $CDCl_3$) δ 7.87 (d, *J* = 6.8 Hz, 2H), 7.54–7.45 (m, 3H), 6.24 (s, 2H), 4.91–4.85 (m, 1H), 4.43–4.36 (m, 1H), 3.93 (bs, 1H), 3.83 (s, 6H), 3.82 (s, 3H), 1.64–1.25 (m, 10H); **^{13}C NMR** (100 MHz, $CDCl_3$) δ 164.6, 160.6, 153.3, 146.3, 135.2, 133.8, 131.8, 128.7, 127.9, 97.3, 74.4, 61.0, 60.9, 56.1, 31.2, 25.1, 23.3; **HRMS (ESI)** m/z: calcd. for $C_{23}H_{30}NO_5$ $[M+H]^+$ 400.2118; Found 400.2116; **HPLC conditions:** Chiralcel IC-H column, 254 nm, flow rate: 1 ml/min, *i*-PrOH/hexanes = 5/95, t_{minor} = 5.28 min, t_{major} = 6.16 min; 96% ee.

cyclohexyl (*R*)-2-((3-chloro-4-fluorophenyl)amino)-2-phenylacetate (70)

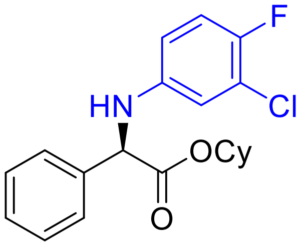

70

Compound **70** was prepared through general **procedure A** and was obtained as a white solid (40.5 mg, 56% yield, 86% ee). **[α]$^{25}_{D}$** = +61.0 (*c* = 0.34, $CHCl_3$); **^{1}H NMR** (400 MHz, $CDCl_3$) δ 7.46 (d, *J* = 7.4 Hz, 2H), 7.37–7.30 (m, 3H), 6.88 (t, *J* = 8.4 Hz, 1H), 6.57–6.53 (m, 1H), 6.40–6.35 (m, 1H), 4.95 (s, 2H), 4.84–4.78 (m, 1H), 1.84–1.80 (m, 1H), 1.72–1.67 (m, 1H), 1.58–1.55 (m, 1H), 1.51–1.45 (m, 3), 1.37–1.24 (m, 4H); **^{13}C NMR** (100 MHz, $CDCl_3$) δ 170.8, 152.4, 150.0, 142.9, 137.2, 128.9, 128.4, 127.1, 121.2, 121.0, 116.9, 116.7, 114.5, 112.5, 74.3, 61.1, 31.4, 30.9, 25.2, 23.3, 23.1; **HRMS (ESI)** m/z: calcd. for $C_{20}H_{22}ClFNO_2$ $[M+H]^+$ 362.1318; Found 362.1321; **HPLC conditions:** Chiralcel IC-H column, 254 nm, flow rate: 1 ml/min, *i*-PrOH/hexanes = 5/95, t_{major} = 8.13 min, t_{minor} = 10.13 min; 86% ee.

cyclohexyl (*R*)-2-(naphthalen-2-ylamino)-2-phenylacetate (71)

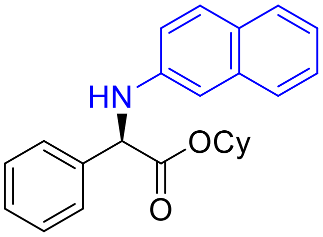

71

Compound **71** was prepared through general **procedure A** and was obtained as a white solid (51.7 mg, 72% yield, 96% ee). **[α]$^{25}_{D}$** = +115.6 (*c* = 0.43, $CHCl_3$); **^{1}H NMR** (400 MHz, $CDCl_3$) δ 7.65–7.48 (m, 5H), 7.37–7.28 (m, 4H), 7.17 (t, *J* = 7.6 Hz, 1H), 6.96 (d, *J* = 8.8 Hz, 1H), 6.65 (s, 1H), 5.18 (s, 1H), 5.15 (bs, 1H), 4.83 (s, 1H), 1.87–1.82 (m, 1H), 1.72–1.69 (m, 1H), 1.62–1.26 (m, 8H); **^{13}C NMR** (100 MHz, $CDCl_3$) δ 171.2, 143.6, 137.6, 134.9, 129.0, 128.7, 128.2, 127.7, 127.6, 127.1, 126.2, 126.0, 122.2, 118.0, 105.6, 74.1, 60.9, 31.4, 309, 25.2, 23.4, 23.1; **HRMS (ESI)** m/z: calcd. for $C_{24}H_{26}NO_2$ $[M+H]^+$ 360.1958; Found 360.1956; **HPLC conditions:** Chiralcel AS-H column, 254 nm, flow rate: 1 ml/min, *i*-PrOH/hexanes = 5/95, t_{minor} = 6.03 min, t_{major} = 7.28 min; 96% ee.

Synthesis of 52 on large scale:

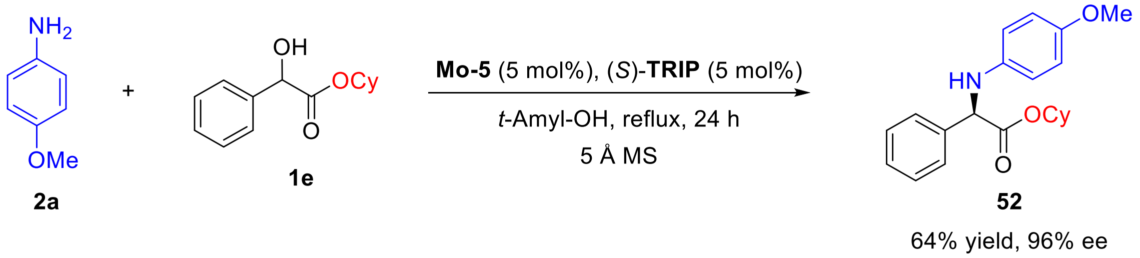

To an oven dried two-necked round-bottom flask equipped with a magnetic stir bar, 5Å MS (250-500 mg), Mo-**5** complex (57.2 mg, 5 mol%), chiral phosphoric acid (*S*)-TRIP (76.0 mg, 5 mol%), α-hydroxy ester **1e** (1.41 g, 6 mmol), and *p*-anisidine **2a** (246.3 mg, 2 mmol) were added. Anhydrous *t*-amyl-OH (20 mL) was added and one neck of the flask was then fitted with reflux condenser and the other neck was sealed tightly using screw cap and paraffin film. The flask was then purged with nitrogen through reflux condenser and the resulting mixture was refluxed at 150 ˚C for 48 hours. The reaction mixture was then transferred to a 100 mL round bottom flask and the solvent was removed *in vacuo* with the aid of a rotary evaporator. The obtained residue was purified by flash column chromatography on silica gel to afford pure α-amino ester **52** with 64% (434.5 mg) of isolated yield and 96% of enantioselectivity.

Synthesis of compound 73:

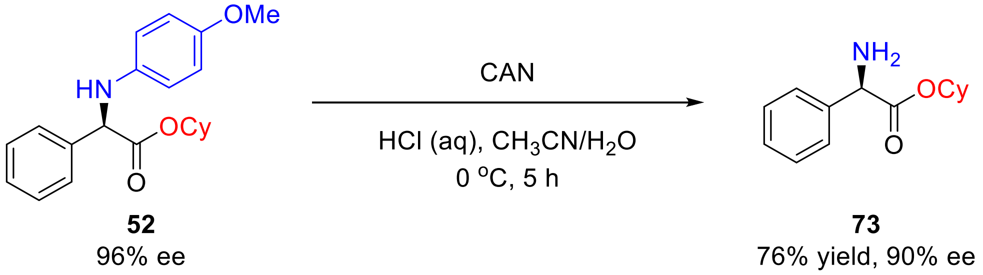

According to the literature,[7b] cyclohexyl (*R*)-2-((4-methoxyphenyl)amino)-2-phenylacetate **52** (339.44 mg, 1.0 mmol) was dissolved in acetonitrile/water (4 mL/2 mL), and hydrochloric acid (1 M, 2 mL) was added. Then ceric ammonium nitrate (CAN) aqueous solution (2.03 g dissolved in 3 mL water, 3.71 mmol) was added in four portions to the reaction mixture in an ice bath. After stirring for 5 h, the resulting dark brown solution was diluted with EtOAc (10 mL) and extracted with water (3 × 5 mL). The combined aqueous layer was treated with saturated $NaHSO_3$ solution (5 mL) and was then extract the aqueous layer. The collected organic layer was dried over Na_2SO_4, and concentrated in vacuo. The crude product was purified by silica-gel column chromatography. The obtained product **73** was a colorless oily liquid with 76% (177.3 mg) of isolated yield and 90% of enantioselectivity. **$[\alpha]^{25}_D$** = +30.5 (*c* = 0.70, $CHCl_3$); **^{1}H NMR** (400 MHz, $CDCl_3$) δ 7.39–7.25 (m, 5H), 4.82–4.75 (m, 1H), 4.58 (s, 1H), 1.98 (bs, 2H), 1.84–1.80 (m, 1H), 1.69–1.66 (m, 2H), 1.56–1.45 (m, 3H), 1.40–1.20 (m, 4H); **^{13}C NMR** (100 MHz, $CDCl_3$) δ 173.4, 140.6, 128.6, 127.8, 126.7, 73.5, 58.9, 31.4, 31.0, 25.2, 23.5, 23.3; **HRMS (ESI)** m/z: calcd. for $C_{14}H_{20}NO_2$ $[M+H]^+$ 234.1489; Found 234.1487; **HPLC conditions:** Chiralcel IC-H column, 254 nm, flow rate: 1 ml/min, *i*-PrOH/hexanes = 30/70, t_{major} = 7.89 min, t_{minor} = 8.69 min; 90% ee.

Synthesis of (–)-Flamprop-methyl (74):

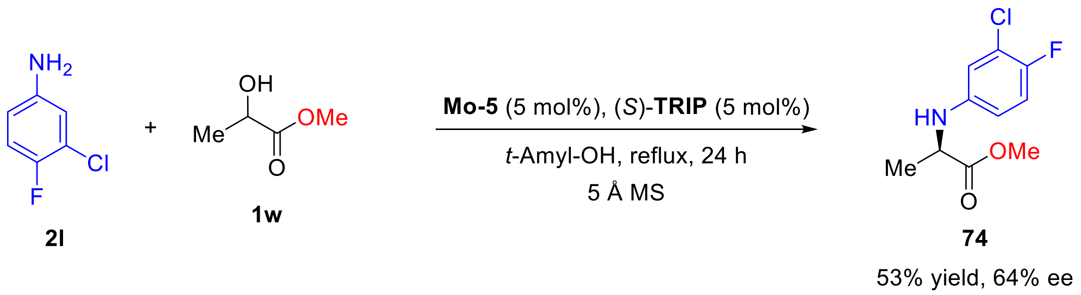

To an oven dried two-necked round-bottom flask equipped with a magnetic stir bar, 5Å MS (250-500 mg), Mo-**5** complex (57.2 mg, 5 mol%), chiral phosphoric acid (*S*)-TRIP (76.0 mg, 5 mol%), α-hydroxy ester **1w** (2.03 g, 6 mmol), and 3-chloro-4-fluoroaniline **2l** (291.12 mg, 2 mmol) were added. Anhydrous *t*-amyl-OH (20 mL) was added and one neck of the flask was then fitted with reflux condenser and the other neck was sealed tightly using screw cap and paraffin film. The flask was then purged with nitrogen through reflux condenser and the resulting mixture was refluxed at 150 ˚C for 48 hours. The reaction mixture was then transferred to a 100 mL round bottom flask and the solvent was removed *in vacuo* with the aid of a rotary evaporator. The obtained residue was purified by flash column chromatography on silica gel to afford pure α-amino ester **74** with 53% (245.5 mg) of isolated yield and 64% of enantioselectivity. **$[\alpha]^{25}_D$** = +19.1 (*c* = 1.2, $CHCl_3$). Lit. data:[7a] $[\alpha]^{20}_D$ = –39.7 (*c* = 0.42, CH_2Cl_2); **^{1}H NMR** (400 MHz, $CDCl_3$) δ 6.93 (t, *J* = 8.8 Hz, 1H), 6.60 (dd, *J* = 6.6, 2.8 Hz, 1H), 6.43 (dt, *J* = 8.9, 3.3 Hz, 1H), 4.15 (s, 1H), 4.08–4.01 (m, 1H), 3.73 (s, 3H), 1.45 (d, *J* = 6.8 Hz, 3H); **^{13}C NMR** (100 MHz, $CDCl_3$) δ 174.7, 152.6, 150.2, 143.5, 143.5, 1213, 121.1, 117.0, 116.8, 114.5, 112.7, 112.6, 52.4, 52.3, 18.8; **HRMS (ESI)** m/z: calcd. for $C_{10}H_{12}ClFNO_2$ $[M+H]^+$ 232.0535; Found 232.0530; **HPLC conditions:** Chiralcel OJ-H column, 254 nm, flow rate: 1 ml/min, *i*-PrOH/hexanes = 5/95, t_{major} = 5.92 min, t_{minor} = 7.77 min; 64% ee.

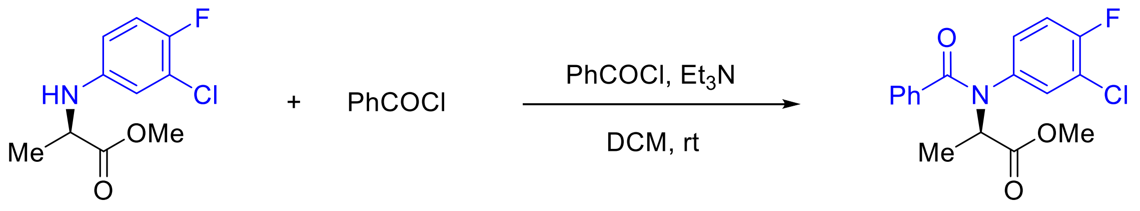

74 **75**

87% yield, 63% ee

According to the literature,[7b] benzoyl chloride (140.6 mg, 1.0 mmol) was added dropwise to the solution of **74** (115.8 mg, 0.5 mmol) in CH_2Cl_2 (5.0 mL) and stirred for 20 minutes under a nitrogen atmosphere. The triethylamine (50.6 mg, 0.5 mmol) was slowly added and stirred at room temperature for overnight. The resulting mixture was purified by flash column chromatography (EA/PE = 1:10) to afford pure **75** with 87% (146.0 mg) of isolated yield and 63% of enantioselectivity. **$[\alpha]^{25}_D$** = –15.2 (*c* = 1.6, $CHCl_3$). Lit. data:[7b] $[\alpha]^{20}_D$ = +34.6 (*c* = 0.5, $CHCl_3$); **^{1}H NMR** (400 MHz, $CDCl_3$) δ 7.92–7.19 (m, 6H), 7.05–6.94 (m, 2H), 5.09–5.03 (m, 1H), 3.80 (s, 3H), 1.45 (d, *J* = 6.6 Hz, 3H); **^{13}C NMR** (100 MHz, $CDCl_3$) δ 172.1, 170.6, 158.3, 155.8, 137.9, 135.3, 133.5, 131.4, 130.1, 129.5, 128.2, 128.0, 121.3, 121.1, 116.8, 116.6, 56.7, 52.5, 52.4, 15.4; **HRMS (ESI)** m/z: calcd. for $C_{17}H_{16}ClFNO_3$ $[M+H]^+$ 336.0797; Found 336.0794;

HPLC conditions: Chiralcel OD-H column, 254 nm, flow rate: 1 ml/min, *i*-PrOH/hexanes = 5/95, t_{minor} = 12.89 min, t_{major} = 16.43 min; 63% ee.

Procedures for Mechanistic Experiments

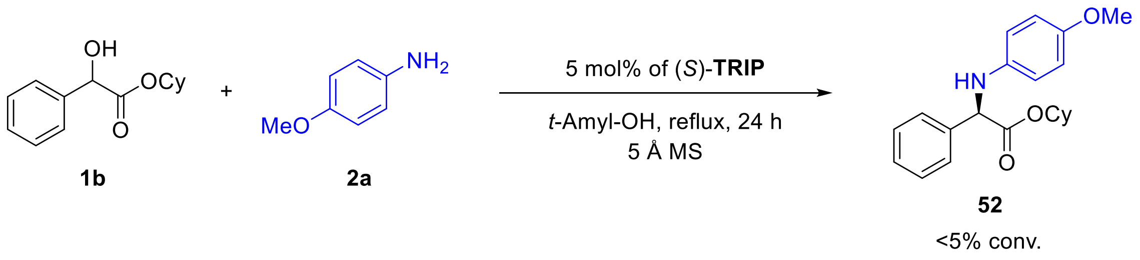

The reaction was set up following the asymmetric amination general procedure B without the addition of **Mo-5** complex. After cooling to room temperature, the crude reaction mixture was passed through a short silica-packed column, concentrated under reduced pressure and was analyzed by ^{1}H NMR.

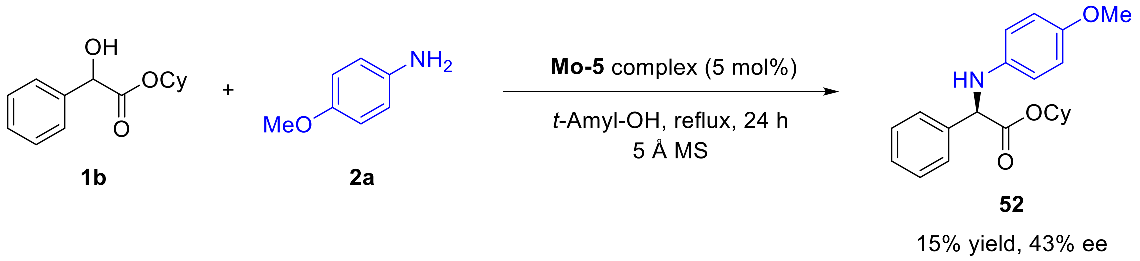

The reaction was set up following the asymmetric amination general procedure B without the addition of (*S*)-TRIP. After cooling the reaction to room temperature, the crude reaction mixture was purified by column chromatography and compound **52** was collected in 15% of isolated yield with 43% of enantioselectivity.

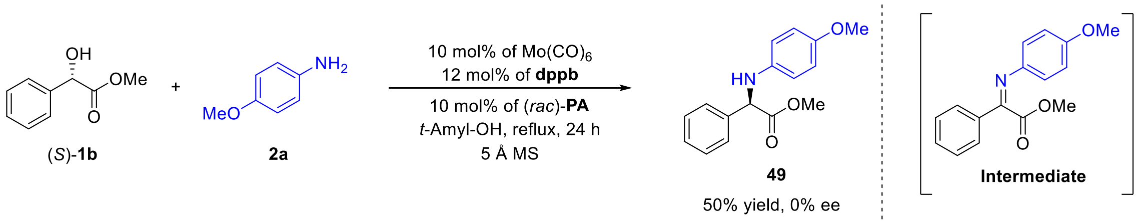

The reaction was set up for the amination of chiral ester (*S*)-**1b** by following general amination procedure A. After cooling the reaction to room temperature, the crude reaction mixture was

purified by column chromatography and compound **49** was collected in 50% of isolated yield with 0% of enantioselectivity.

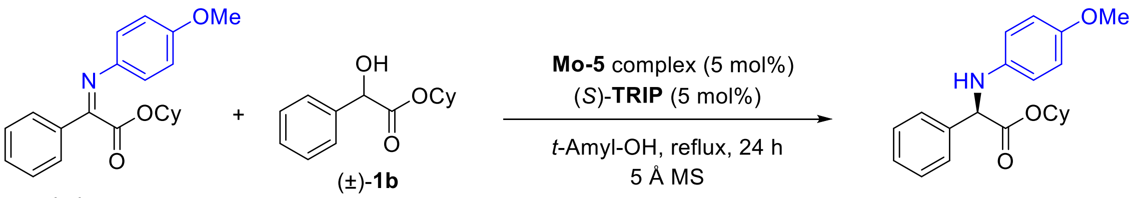

α-imino ester (±)-**1b** **52** 76% yield, 90% ee

To an oven dried two-necked round-bottom flask equipped with a magnetic stir bar, 5Å MS (80-100 mg), Mo-**5** complex (5.7 mg, 10 mol%), chiral phosphoric acid (*S*)-**TRIP** (7.5 mg, 5 mol%), α-ketimin-ester (67.48 mg, 0.2 mmol), and α-hydroxy ester **1b** (46.86 mg, 0.2 mmol) were added. Anhydrous *t*-amyl-OH (2 mL) was added and the reaction was refluxed for 24 hours according to general procedure B. The reaction mixture was then transferred to a 50 mL round bottom flask and the solvent was removed *in vacuo* with the aid of a rotary evaporator. The obtained residue was purified by flash column chromatography on silica gel to afford pure α-amino ester **52** in 76% of isolated yield with 90% of enantioselectivity.

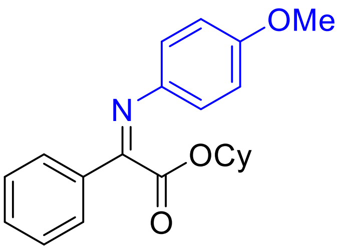

α-imino ester

Preparation of imine ester: This compound was prepared according to the previously reported literature procedure and was collected as yellow solid.[1a] **^{1}H NMR** (400 MHz, $CDCl_3$) δ 7.87 (d, *J* = 6.8 Hz, 2H), 7.51–7.43 (m, 3H), 6.96 (d, *J* = 8.2 Hz, 2H), 6.86 (d, *J* = 8.2 Hz, 2H), 4.93–4.87 (m, 1H), 3.80 (s, 3H), 1.66–1.16 (m, 10H); **^{13}C NMR** (100 MHz, $CDCl_3$) δ 165.0, 159.9, 157.2, 143.4, 134.2, 131.4, 128.6, 127.8, 121.2, 114.0, 74.3, 55.5, 31.2, 25.1, 23.4; **HRMS (ESI)** m/z: calcd. for $C_{21}H_{24}NO_3$ $[M+H]^+$ 338.1751; Found 338.1749.

General procedure for nonlinear effect (NLE) studies

NLE studies with achiral phosphoric acid (*rac*-TRIP):

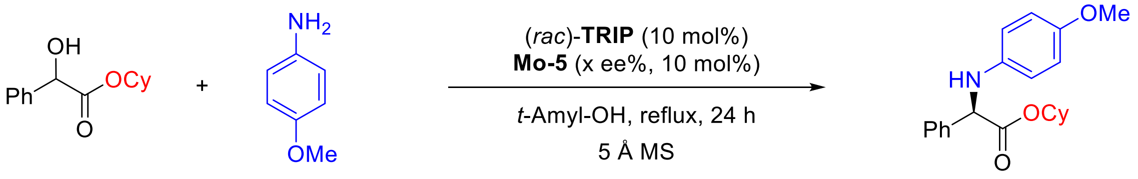

1e **2a** **52**

Combining the certain amounts of optically pure (*S*)-**Mo-5** with pure (*R*)-**Mo-5** leads to the formation of the specified ee values of **Mo-5**. Several reactions containing the mixed catalyst (**Mo-5**) of racemic, 20%, 40%, 60%, 80% and 100% (>99%) ee were performed in parallel (Figure S1). A linear plot of ee_{prod} (the enantiomeric excess of product) as a function of $ee_{Mo\text{-}5}$ (the enantiomeric excess of catalyst **Mo-5**) was obtained (R^2 = 0.9973). The absence of an NLE indicated that one chiral catalyst **Mo-5** was involved in the catalytic cycle.

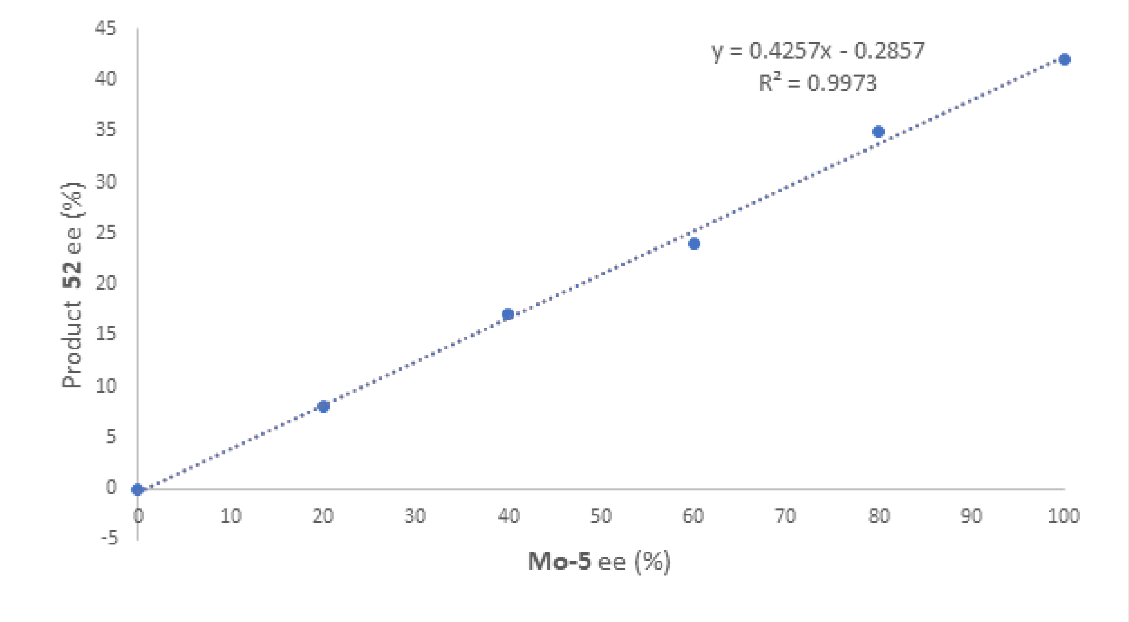

Entry	*ee* of Mo-5 (%)	*ee* of product 52 (%)
**1**	0	0
**2**	20	8
**3**	40	17
**4**	60	24
**5**	80	35
**6**	100	42

Figure S1. Nonlinear effect studies of achiral phosphoric acid (*rac*-TRIP).

General Procedure: To an oven dried two-necked round-bottom flask equipped with a magnetic stir bar, 5Å MS (100-150 mg), Mo-**5** complex (11.4 mg, x ee %, 10 mol%), achiral phosphoric acid (*rac*-TRIP) (15.0 mg, 10 mol%), *α*-hydroxy ester **1e** (140.58 mg, 0.6 mmol), and

p-anisidine **2a** (24.63 mg, 0.2 mmol) were added. Anhydrous ***t***-amyl-OH (2 mL) was added and one neck of the flask was then fitted with reflux condenser and the other neck was sealed tightly using screw cap and paraffin film. The flask was then purged with nitrogen through reflux condenser and the resulting mixture was refluxed at 150 ˚C for 24 hours. The reaction mixture was then transferred to a 50 mL round bottom flask and the solvent was removed ***in vacuo*** with the aid of a rotary evaporator. The obtained residue was purified by flash column chromatography on silica gel to afford pure α-amino ester **52**. A graph of ee of product vs. ee of catalyst was then plotted.

NLE studies with chiral phosphoric acid (*S*-TRIP):

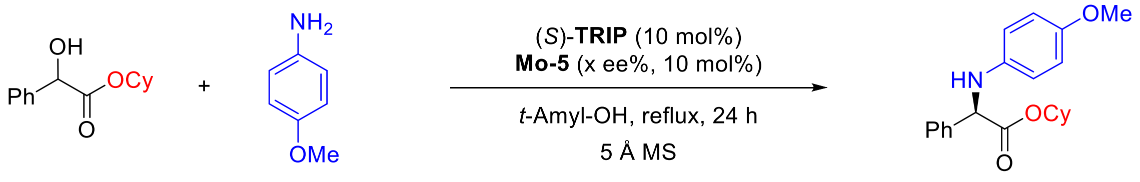

1e **2a** **52**

Combining the certain amounts of optically pure (*S*)-**Mo-5** with pure (*R*)-**Mo-5** leads to the formation of the specified ee values of **Mo-5**. Several reactions containing the mixed catalyst (**Mo-5**) of 0% (racemic), 20%, 40%, 60%, 80% and 100% (optically pure (*S*)-Mo-5) were performed in parallel (Figure S2) and a positive nonlinear effect was obtained.

General Procedure: To an oven dried two-necked round-bottom flask equipped with a magnetic stir bar, 5Å MS (100-150 mg), Mo-**5** complex (11.4 mg, x ee %, 10 mol%), chiral phosphoric acid (*S*)-TRIP (15.0 mg, 10 mol%), α-hydroxy ester **1e** (140.58 mg, 0.6 mmol), and ***p***-anisidine **2a** (24.63 mg, 0.2 mmol) were added. Anhydrous ***t***-amyl-OH (2 mL) was added and one neck of the flask was then fitted with reflux condenser and the other neck was sealed tightly using screw cap and paraffin film. The flask was then purged with nitrogen through reflux condenser and the resulting mixture was refluxed at 150 ˚C for 24 hours. The reaction mixture was then transferred to a 50 mL round bottom flask and the solvent was removed ***in vacuo*** with the aid of a rotary evaporator. The obtained residue was purified by flash column chromatography

on silica gel to afford pure α-amino ester **52**. A graph of ee of product vs. ee of catalyst was then plotted.

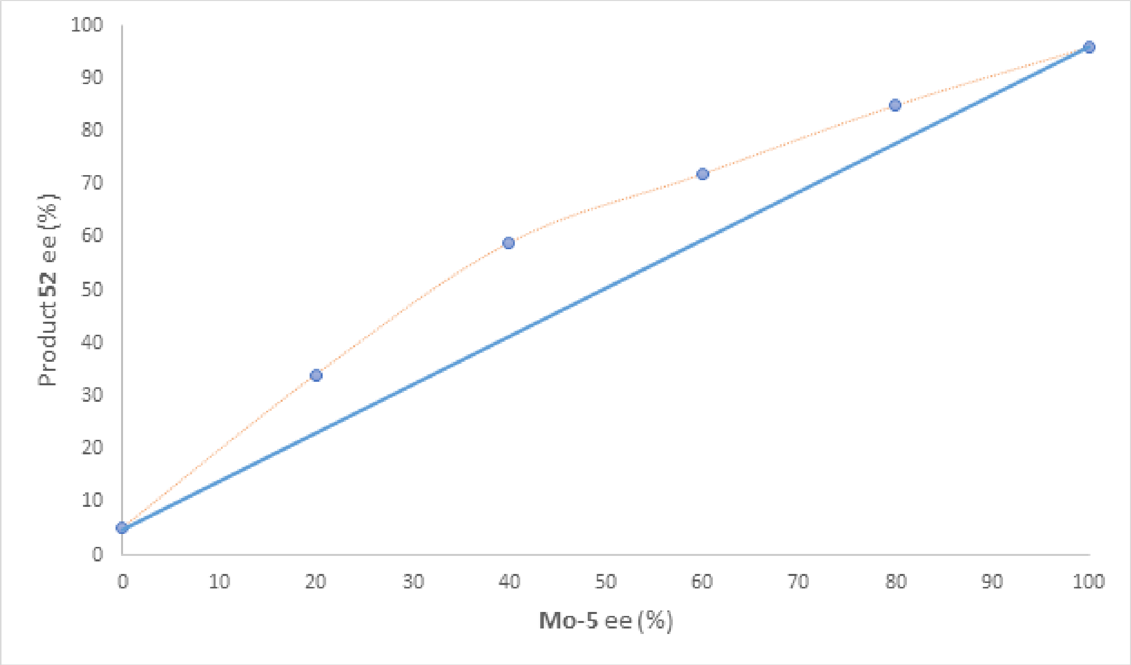

Entry	*ee* of Mo-5 (%)	*ee* of product 52 (%)
**1**	0	<5
**2**	20	34
**3**	40	59
**4**	60	72
**5**	80	85
**6**	100	96

Figure S2. Nonlinear effect studies of chiral phosphoric acid (*S*-TRIP).

X-ray crystallography of compound 53

A single-crystal of compound **53** was obtained through slow evaporation from its solution in *n*-hexane. The structure of **53** was then determined by X-ray crystallographic analysis (CCDC No: 2325435). Diffraction data were collected on a Bruker SMART Apex-II CCD-based X-ray diffractometer with Cu-Kα radiation. The empirical absorption correction was applied by using the SADABS program. The structure was solved using direct method, and refined by full-matrix

least-squares on F^2 (G.M. Sheldrick, SHELXTL2008, program of crystal structure refinement, University of Göttingen, Germany, 1997). H-atoms were refined isotopically, while all other atoms were refined anisotropically. The crystallographic data is summarized in Table S6 and the diagram is shown in Figure S3.

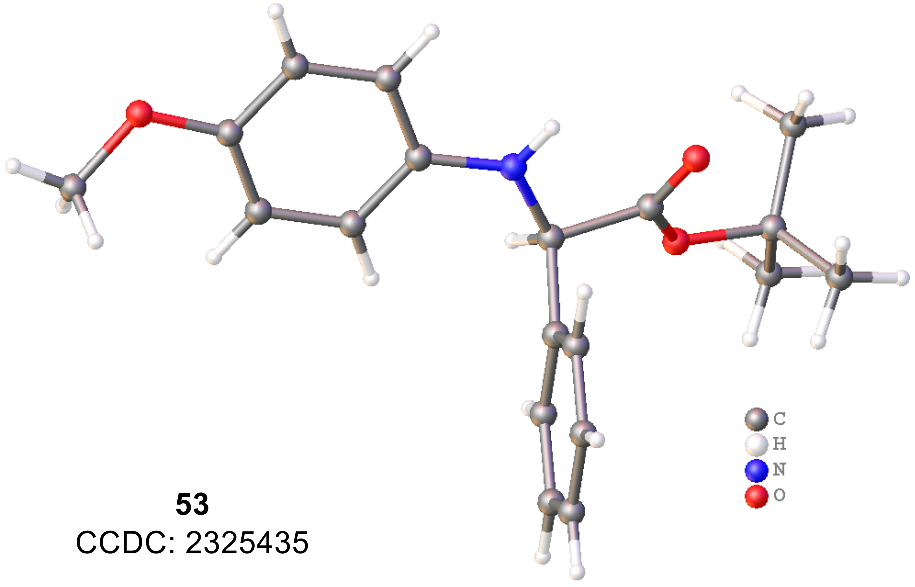

53
CCDC: 2325435

Figure S3. X-ray of compound **53**.

Table S6. Crystal data and structure refinement for compound **53**.

Identification code	A
Empirical formula	$C_{19}H_{23}NO_3$
Formula weight	313.38
Temperature/K	193.00
Crystal system	monoclinic
Space group	$P2_1$
a/Å	5.8435(3)
b/Å	10.3279(5)
c/Å	14.3119(8)
α/°	90
β/°	95.862(2)

γ/°	90
Volume/Å^3	859.22(8)
Z	2
ρ_{calc}g/cm^3	1.211
μ/mm^{-1}	0.081
F(000)	336.0
Crystal size/mm^3	0.12 × 0.1 × 0.09
Radiation	MoKα (λ = 0.71073)
2Θ range for data collection/°	4.872 to 52.736
Index ranges	$-7 \leq h \leq 7$, $-12 \leq k \leq 11$, $-17 \leq l \leq 17$
Reflections collected	7479
Independent reflections	2689 [R_{int} = 0.0503, R_{sigma} = 0.0527]
Data/restraints/parameters	2689/1/212
Goodness-of-fit on F^2	1.109
Final R indexes [I>=2σ (I)]	R_1 = 0.0439, wR_2 = 0.0897
Final R indexes [all data]	R_1 = 0.0660, wR_2 = 0.1014
Largest diff. peak/hole / e Å^{-3}	0.15/-0.17
Flack parameter	0.3(10)

X-ray crystallography of Mo-5 complex.

A single-crystal of Mo-complex **5** was obtained through slow evaporation from its solution in dichloromethane/*n*-hexane. The structure of Mo-complex **5** was then determined by X-ray crystallographic analysis (CCDC No: 2325435). Diffraction data were collected on a Bruker SMART Apex-II CCD-based X-ray diffractometer with Cu-Kα radiation. The empirical absorption correction was applied by using the SADABS program. The structure was solved using direct method, and refined by full-matrix least-squares on F^2 (G.M. Sheldrick, SHELXTL2008, program of crystal structure refinement, University of Göttingen, Germany, 1997). H-atoms

were refined isotopically, while all other atoms were refined anisotropically. The crystallographic data is summarized in Table S7 and the diagram is shown in Figure S4.

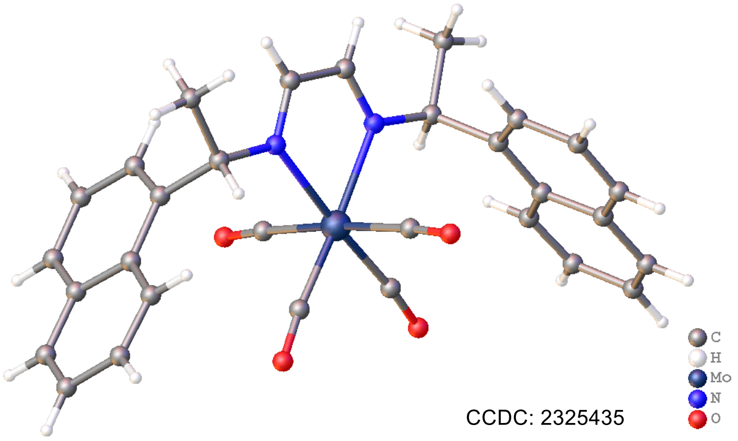

Figure S4. X-ray of Mo-5 complex.

Table S7. Crystal data and structure refinement for compound **Mo-5 complex**.

Identification code	230520_WQANGZ_154251_0m
Empirical formula	$C_{30}H_{24}MoN_2O_4$
Formula weight	572.45
Temperature/K	193.00
Crystal system	hexagonal
Space group	$P6_5/22$
a/Å	7.3913(3)
b/Å	7.3913(3)
c/Å	80.695(5)
α/°	90
β/°	90
γ/°	120
Volume/Å^3	3817.8(4)
Z	6
ρ_{calc}g/cm^3	1.494

μ/mm^{-1}	3.045
F(000)	1752.0
Crystal size/mm^3	0.13 × 0.12 × 0.1
Radiation	GaKα (λ = 1.34139)
2Θ range for data collection/°	5.716 to 120.69
Index ranges	-9 ≤ h ≤ 8, -8 ≤ k ≤ 8, -93 ≤ l ≤ 102
Reflections collected	21211
Independent reflections	2784 [Rint = 0.0882, Rsigma = 0.0682]
Data/restraints/parameters	2784/0/169
Goodness-of-fit on F^2	1.154
Final R indexes [I>=2σ (I)]	R1 = 0.0879, wR2 = 0.1587
Final R indexes [all data]	R1 = 0.1025, wR2 = 0.1659
Largest diff. peak/hole / e Å^{-3}	0.71/-1.13
Flack parameter	0.11(7)

References:

1. a) D. Liu, B. Li, J. Chen, I. D. Gridnev, D. Yan, W. Zhang, *Nat. Commun.*, **2020**, *11*, 5935; b) G. Gu, T. Yang, J. Lu, J. Wen, L. Dang, X. Zhang, *Org. Chem. Front.*, **2018**, *5*, 1209
2. a) H. H. San, S. J. Wang, M. Jiang, X. Y. Tang, *Org. Lett.*, **2018**, *20*, 4672; b) Z. Tang, Q. Jiang, L. Peng, X. Xu, J. Li, R. Qiu, C.-T. Au, *Green Chem.*, **2017**, *19*, 5396; c) L.-Q. Lu, Y. Li, K. Junge, M. Beller, *Angew. Chem. Int. Ed.*, **2013**, *52*, 8382; d) J. Kwaitkowski, J. Majer, P. Kwiatkowski, J. Jurczak, *Synthesis* **2008**, *20*, 3237; e) J. C. Lung, L. M. Geary, T.-Y. Chen, J. R. Zbieg, M. J. Krishe, *J. Am. Chem. Soc.*, **2012**, *134*, 15700; f) S. M. Nicolle, C. J. Hayes, C. J. Moody, *Chem. Eur. J.* **2015**, *21*, 4576; g) E. W. Hei Ng, K.-H. Low, P. Chiu, *J. Am. Chem. Soc.*, **2018**, *140*, 3537.
3. a) R. T. Kumah, N. Tsaulwayo, B. A. Xulu, S. O. Ojwach, *Dalt. Trans*. **2019**, *48*, 13630; b) T. Lundrigan, E. N. Welsh, T. Hynes, C.-H. Tien, M. R. Adams, K. R. Roy, K. N. Robertson,

A. W. H. Speed, *J. Am. Chem. Soc*., **2019**, *141*, 14083; c) E. Braconi, A. C. Gotzinger, N. Cramer, *J. Am. Chem. Soc*. **2020**, *142*, 19819.

4. a) L. Li, P. Sivaguru, X. Han, S. Karmaker, X. Bi, *Org. Chem. Front*., **2023**, *10*, 1746; b) C. Qian, J. Chen, M. Fu, S. Zhu, W.-H. Chen, H. Jiang, W. Zeng, *Org. Biomol. Chem*., **2013**, *11*, 6013; c) K. Ramakrishna, C. Sivasankar, *J. Organomet. Chem*. **2016**, *805*, 122; d) H. He, K. Yan, J. Li, R. Lai, Y. Luo, M. Guan, Y. Wu, *Synthesis* **2020**, *52*, 3065; e) I. Mizota, Y. Tadano, Y. Nakamura, T. Haramiishi, M. Hotta, M. Shimizu, *Org. Lett*., **2019**, *21*, 2663; f) G. Shen, H. Liu, J. Chen, Z. He, Y. Zhou, L. Wang, Y. Luo, Z. Su, B. Fan, *Org. Biomol. Chem*., **2021**, *19*, 3601; g) W. Wu, H. Zhao, J. Chen, F. Zhang, B. Fan, *Chem. Eur. J.* **2020**, *28*, e202202460; h) J. Aziz, J.-D. Brion, A. Hamze, M. Alami, *Adv. Synth. Catal*. **2013**, *355*, 2417; i) Q. Li, H. Sun, F. Yan, Y. Zhao, Y. Zhang, C. Zhou, M.-y. Han, H. Li, X. Sui, *Green Chem*., **2023**, *25*, 6226.
5. C.-Y. Chang, T.-K. Yang, *Tetrahedron: Asymmetry* **2003**, *14*, 2239.
6. a) D. Enders, A. Rembiak, B. A. Stöckel, *Adv. Synth. Catal*., **2013**, *355*, 1937; b) C. Zhu, T. Akiyama, *Adv. Synth. Catal*., **2010**, *352*, 1846; c) J. E. Taylor, D. S. B. Daniels, A. D. Smith, *Org. Lett*., **2013**, *15*, 6058; d) X. Xiao, W. Zhang, X. Lu, Y. Deng, H. Jiang, W. Zeng, *Adv. Synth. Catal*., **2016**, *358*, 2497; e) W. Notz, S.-i. Watanable, N. S. Chowdari, G. Zhong, J. M. Betancort, F. Tanaka, C. F. Barbas, *Adv. Synth. Catal*., **2004**, *346*, 1131.
7. a) K. Liang, Q. Zhang, C. Guo, *Nat. Synth*., **2023**, *2*, 1184; b) B. Liu, B. Li, J. Chen, I. D. Gridnev, D. Yan, W. Zhang, Nat. Commun., 2020, 11, 5935.

^{1}H NMR of Compound **1m** ($CDCl_3$, 400 MHz):

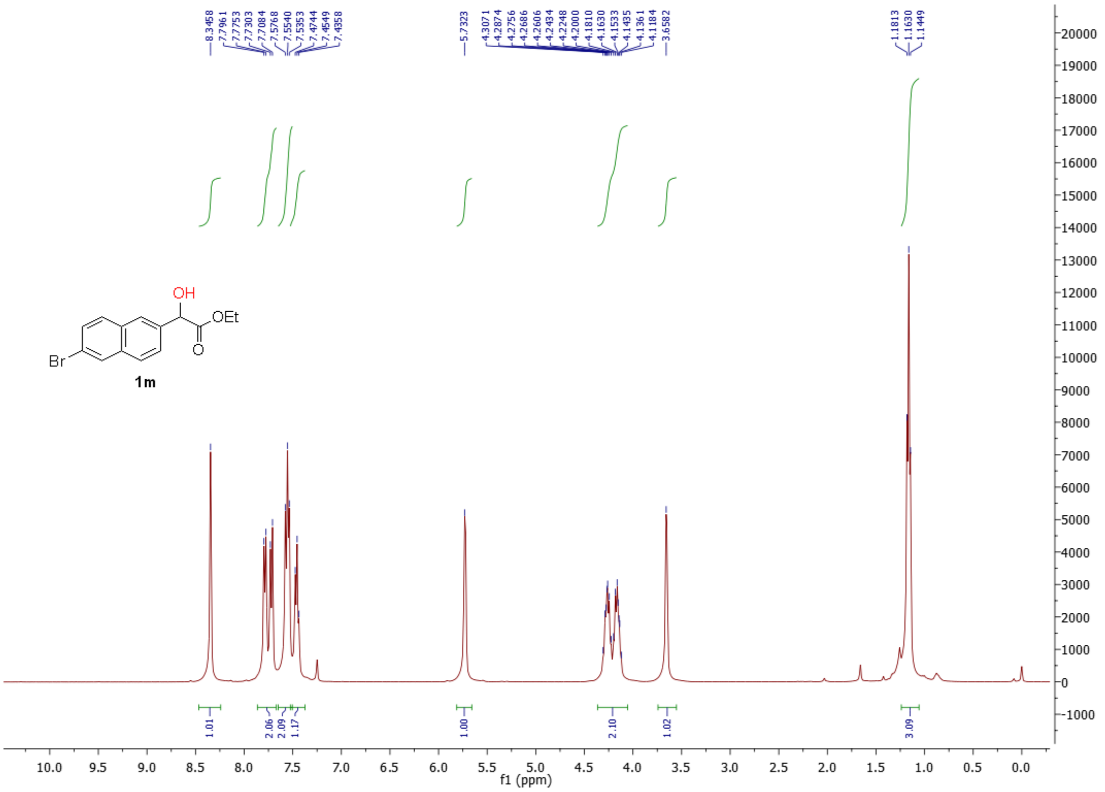

^{13}C NMR of Compound **1m** ($CDCl_3$, 100 MHz):

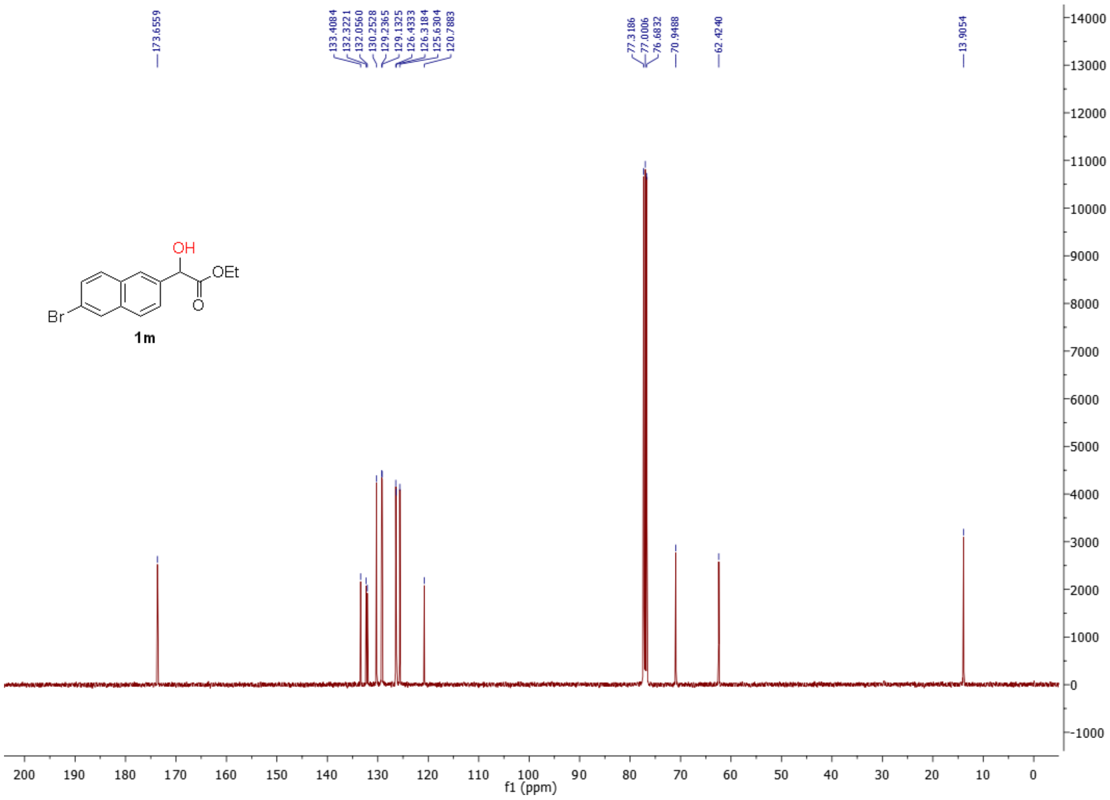

^{1}H NMR of Compound **1n** ($CDCl_3$, 400 MHz):

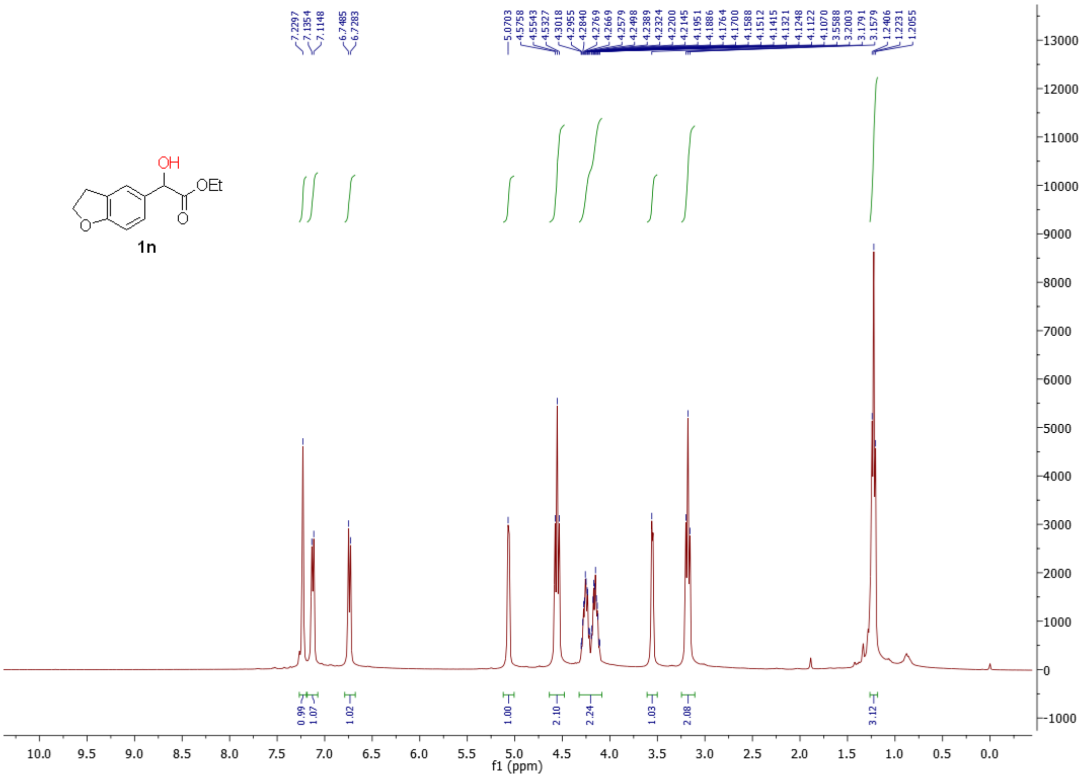

^{13}C NMR of Compound **1n** ($CDCl_3$, 100 MHz):

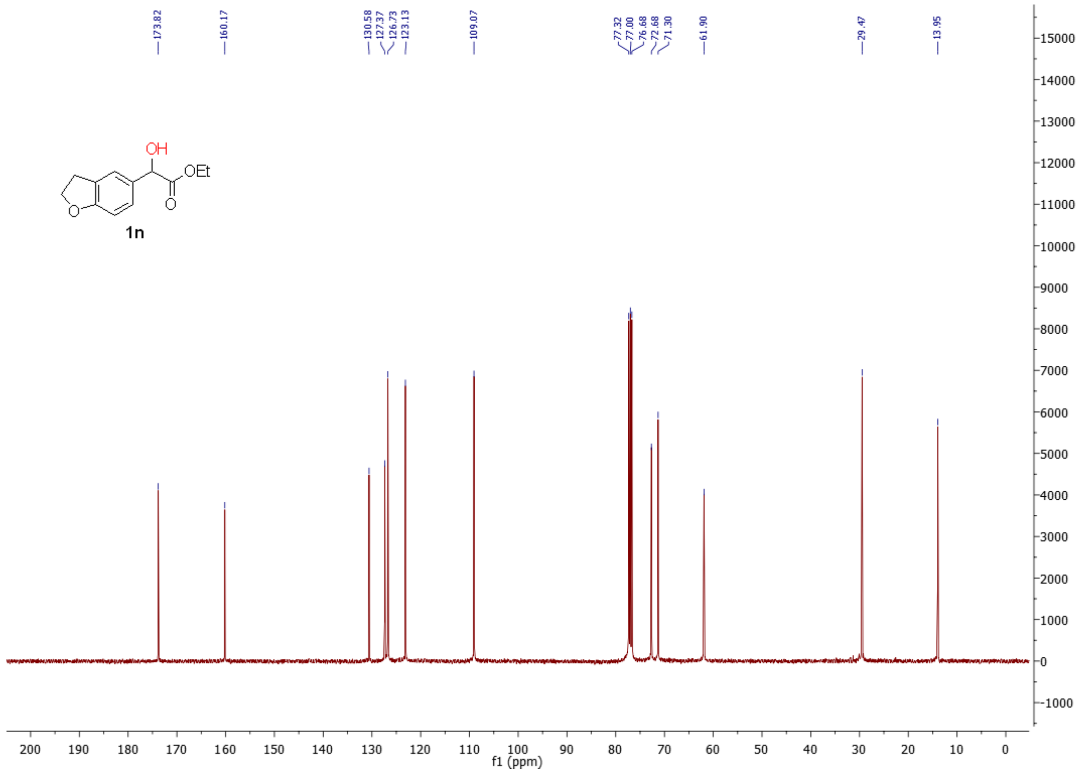

^{1}H NMR of Compound **1o** ($CDCl_3$, 400 MHz):

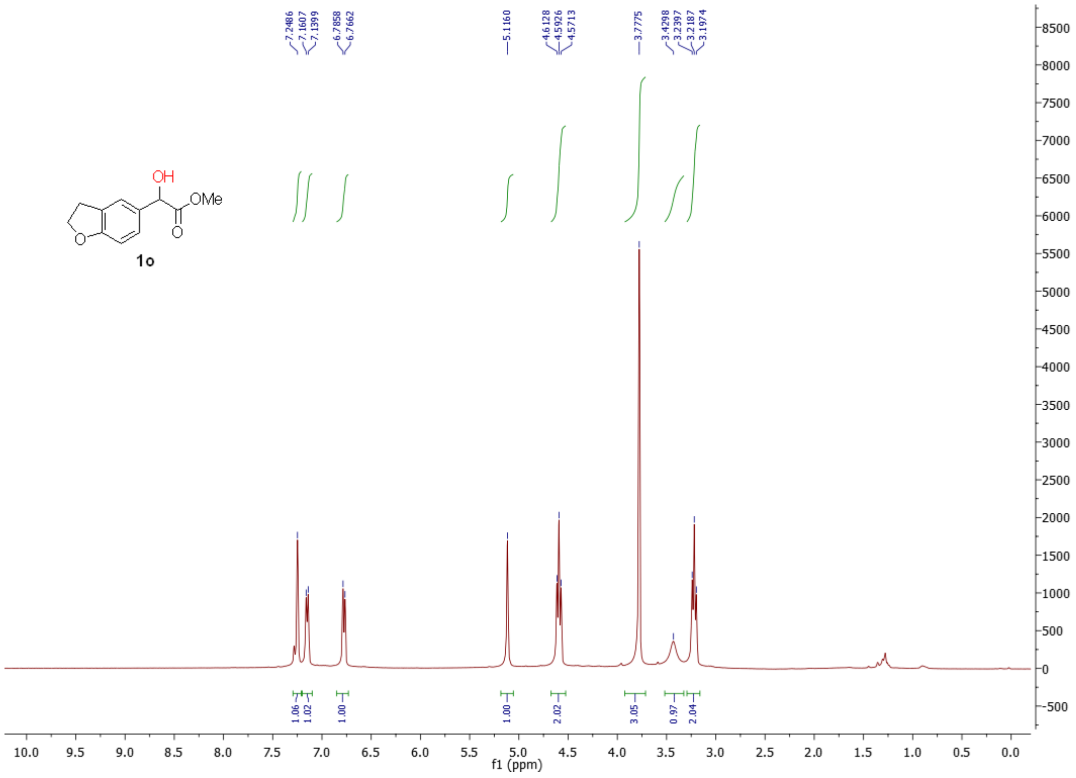

^{13}C NMR of Compound **1o** ($CDCl_3$, 100 MHz):

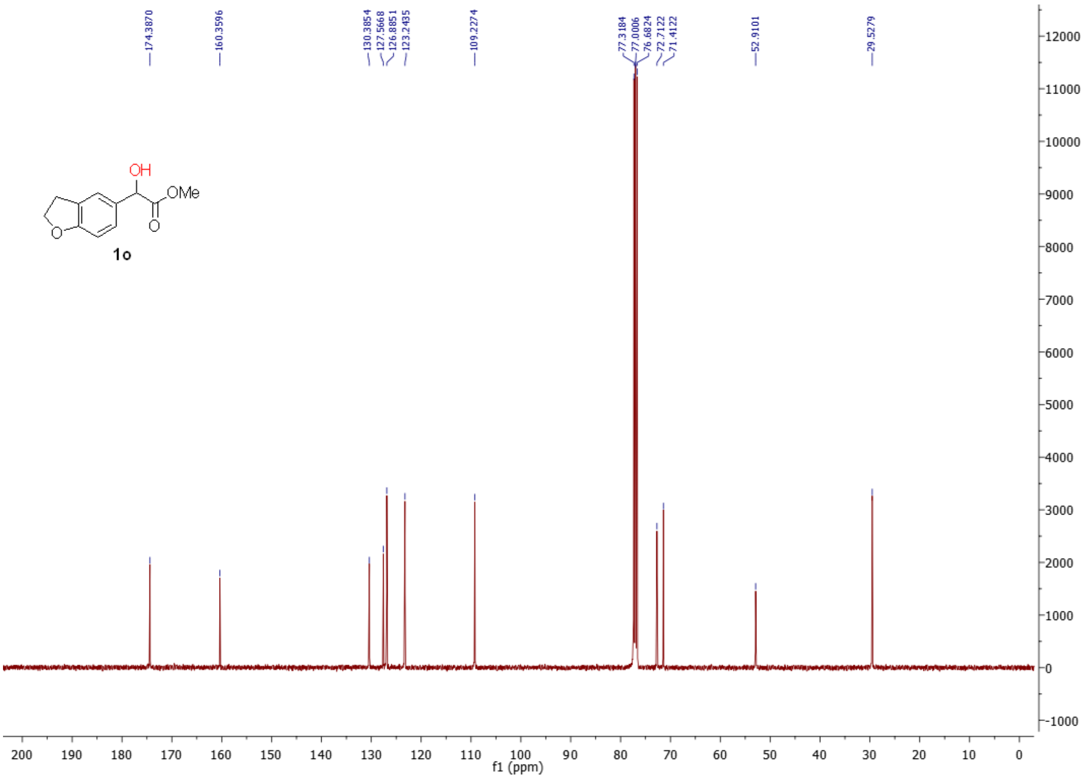

^{1}H NMR of Compound **1q** ($CDCl_3$, 400 MHz):

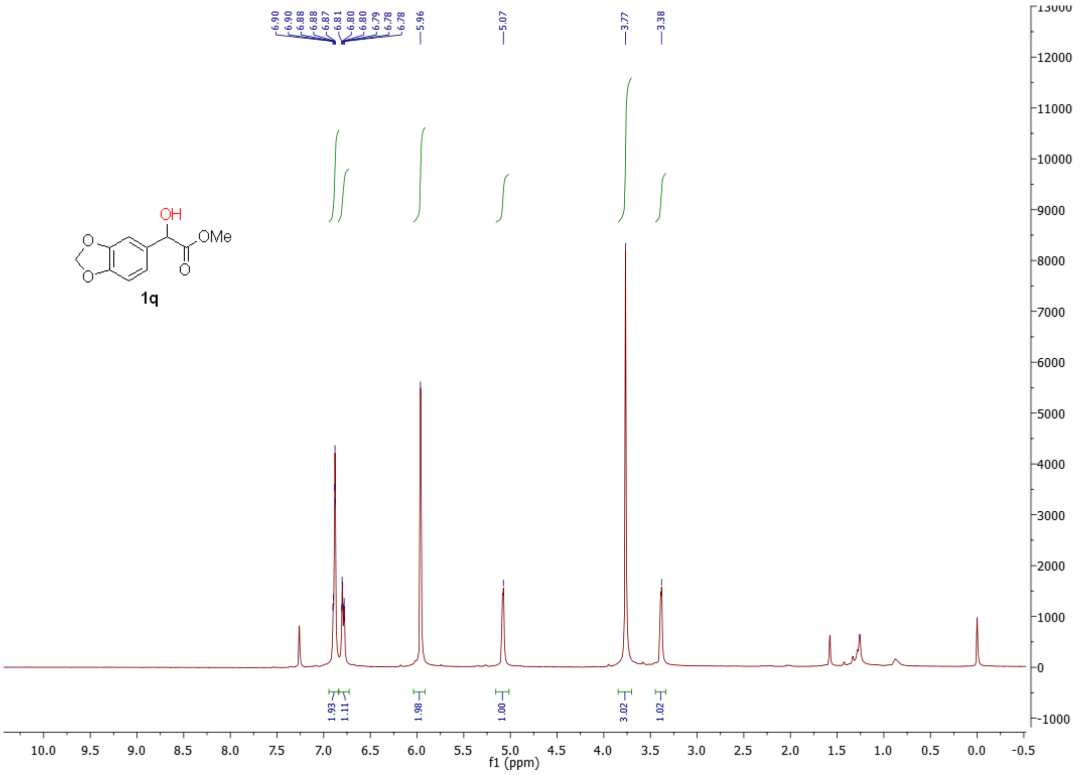

^{13}C NMR of Compound **1q** ($CDCl_3$, 100 MHz):

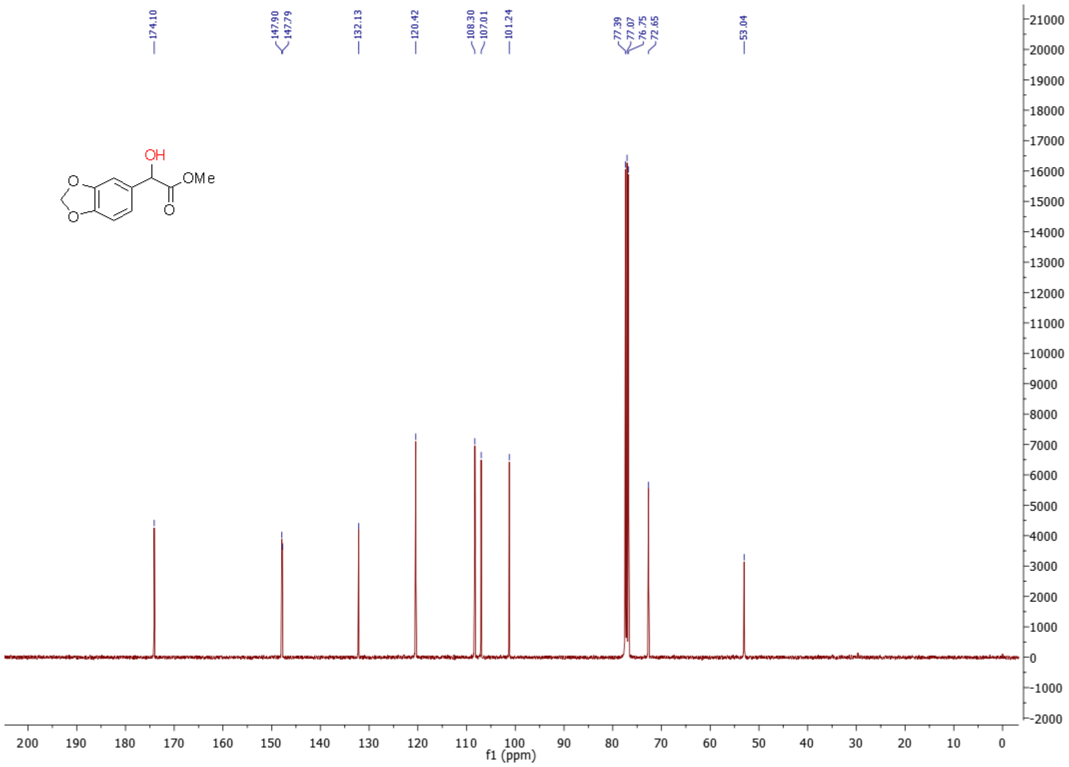

^{1}H NMR of Compound **1s** ($CDCl_3$, 400 MHz):

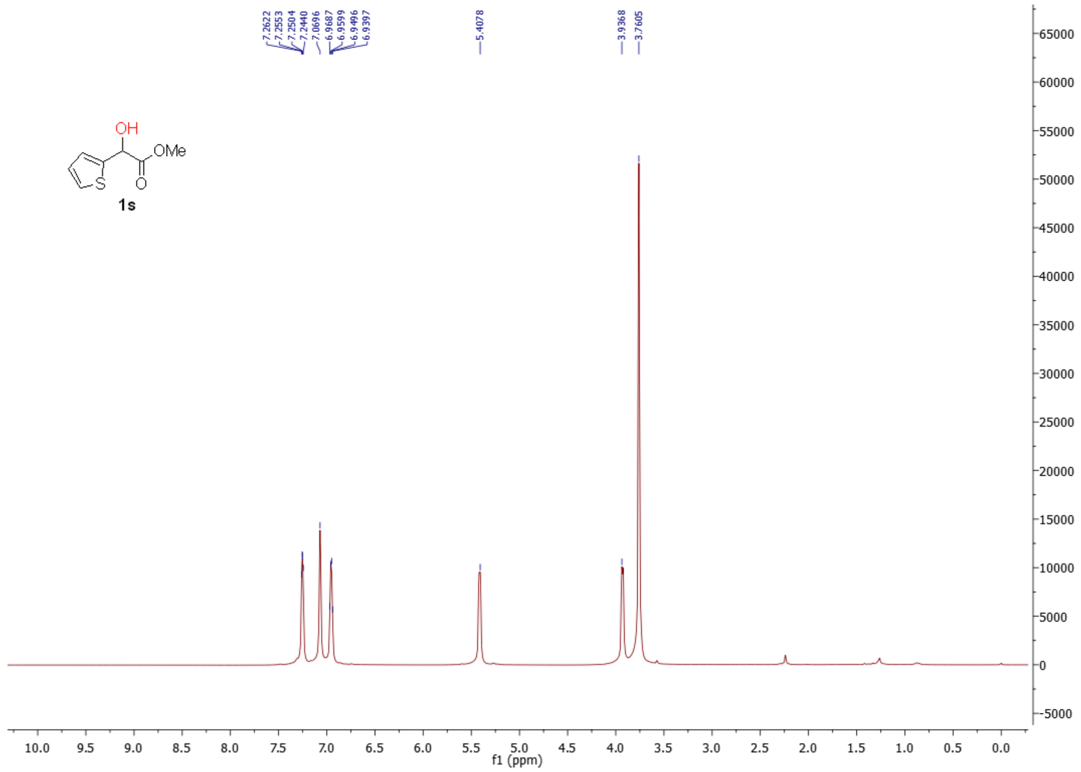

^{13}C NMR of Compound **1s** ($CDCl_3$, 100 MHz):

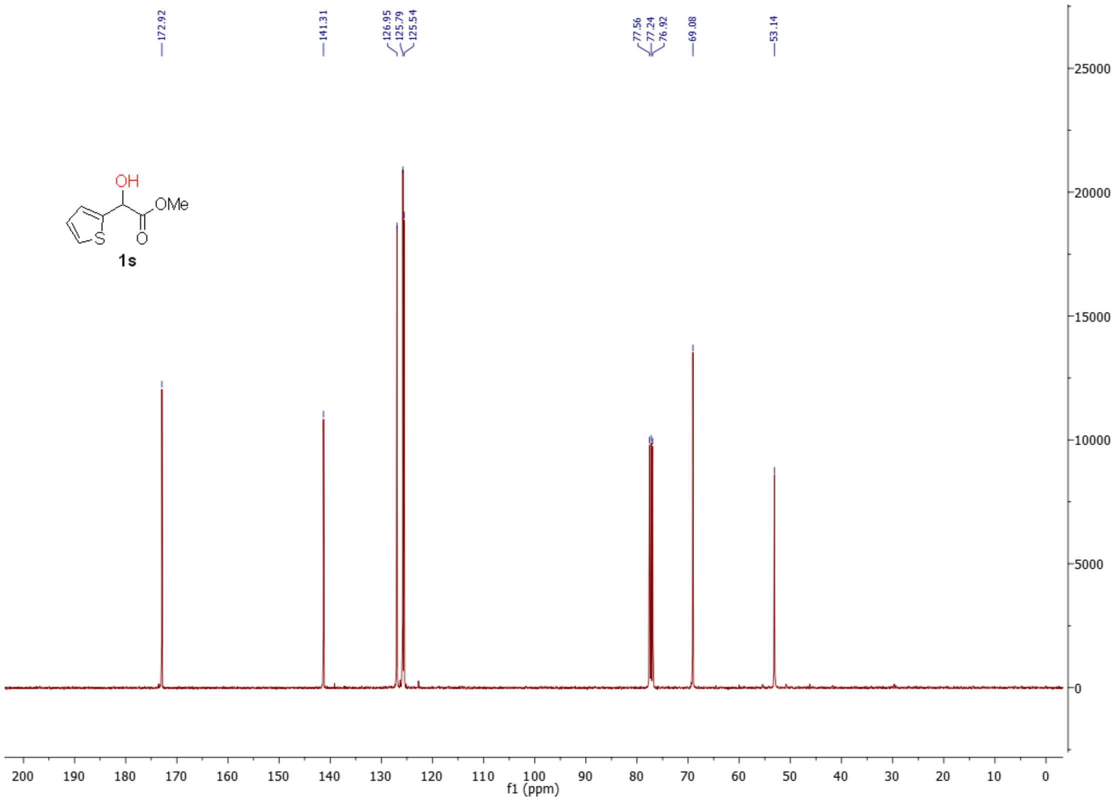

^{1}H NMR of Compound **1t** ($CDCl_3$, 400 MHz):

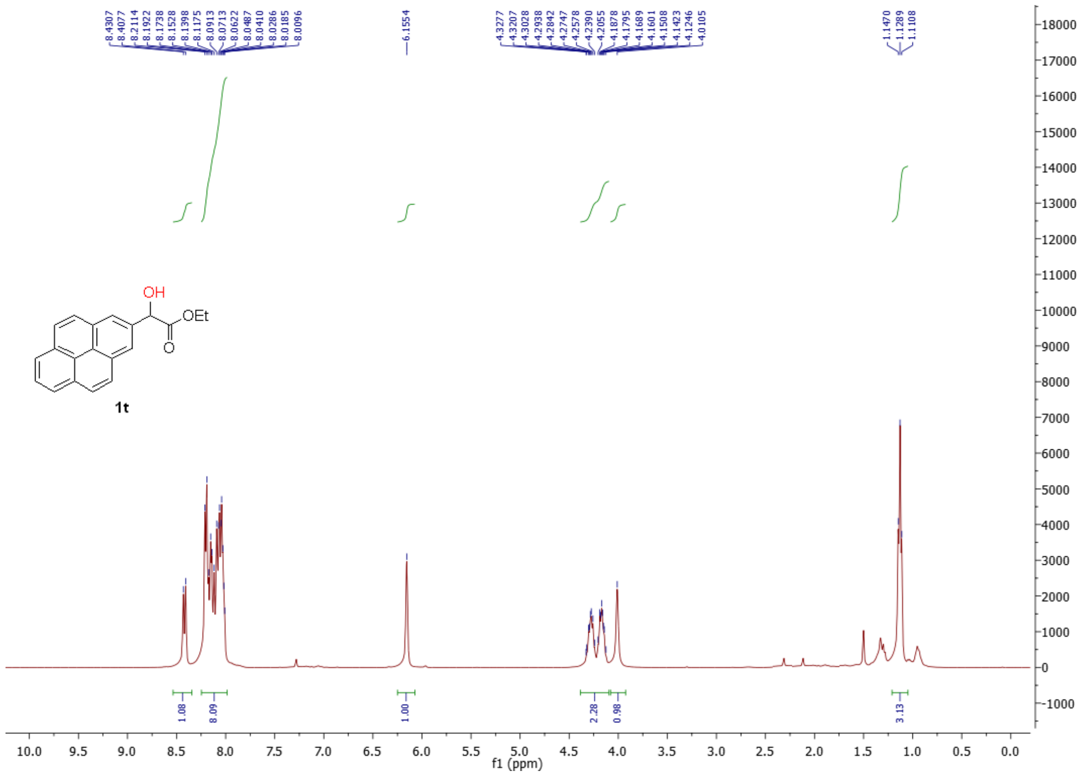

^{13}C NMR of Compound **1t** ($CDCl_3$, 100 MHz):

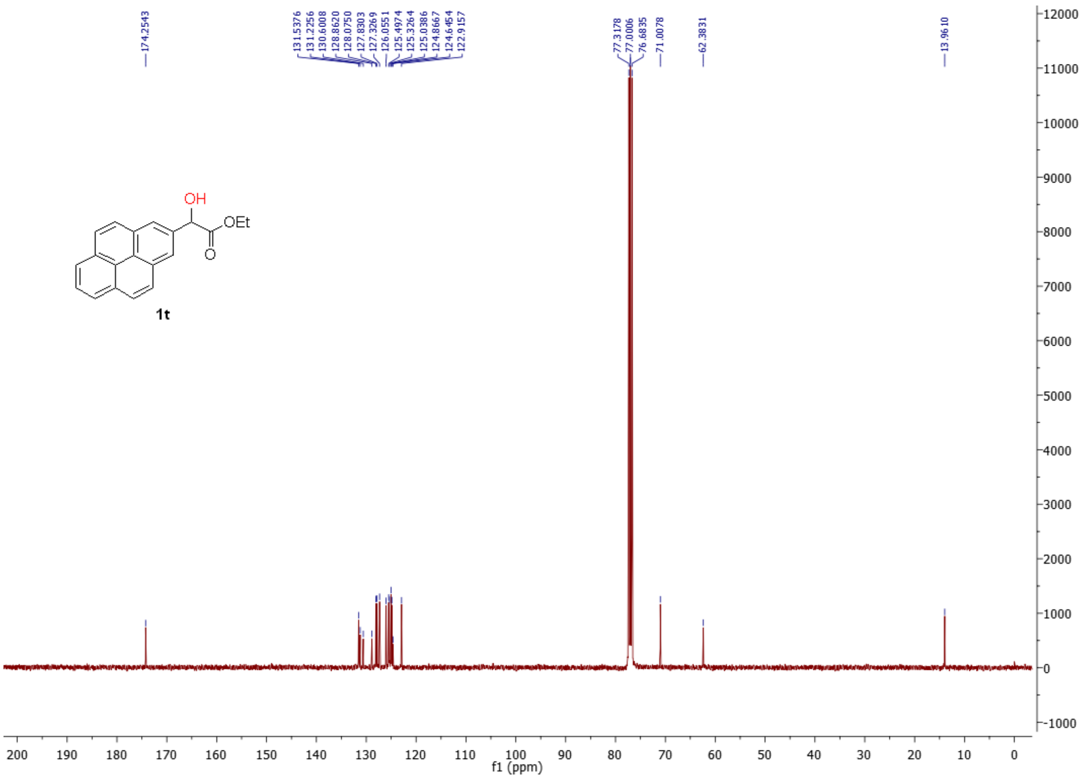

^{1}H NMR of Compound **1u** ($CDCl_3$, 400 MHz):

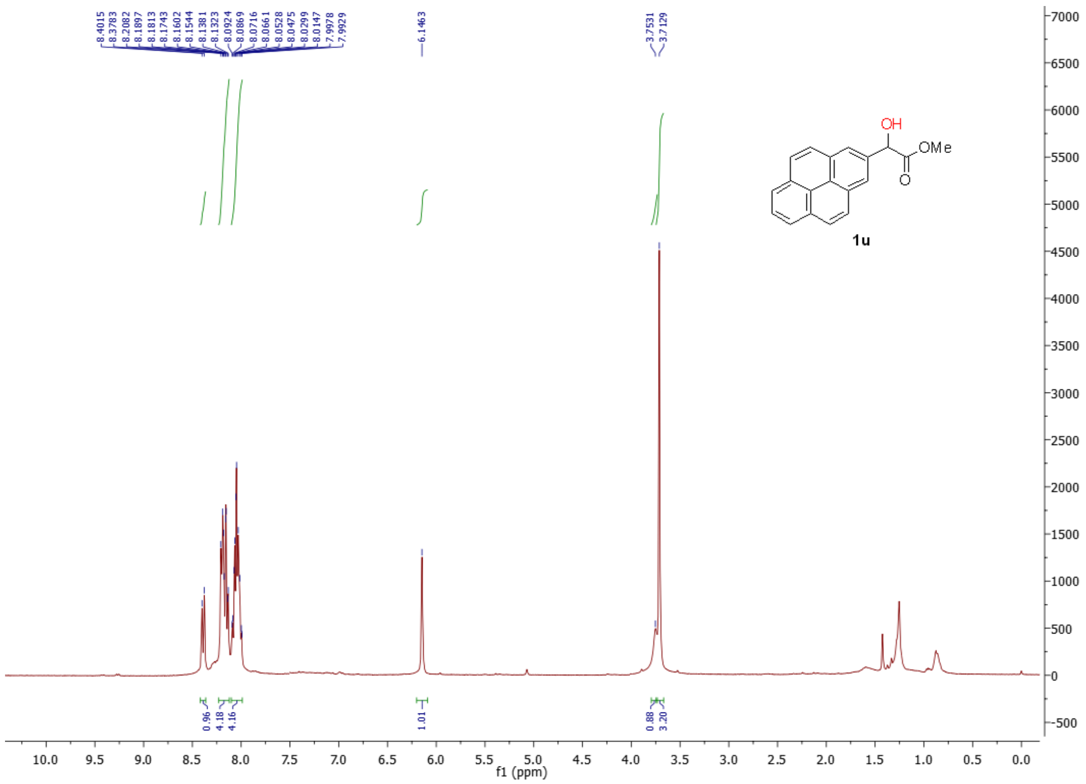

^{13}C NMR of Compound **1u** ($CDCl_3$, 100 MHz):

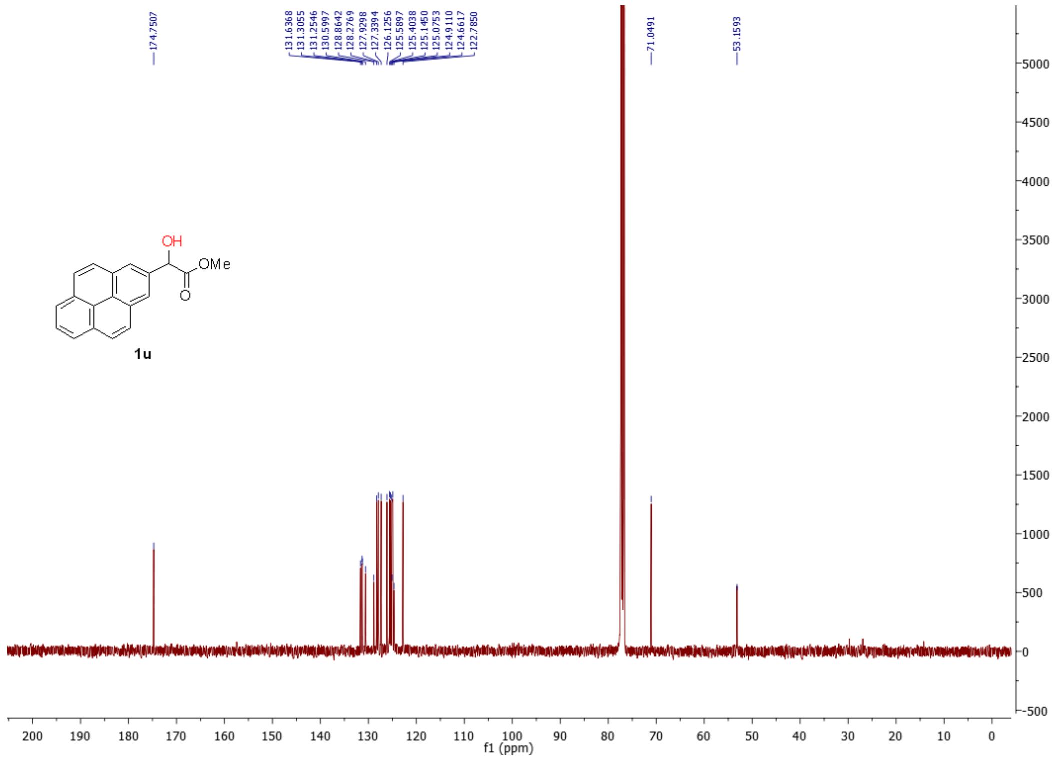

^{1}H NMR of Compound **1z** ($CDCl_3$, 400 MHz):

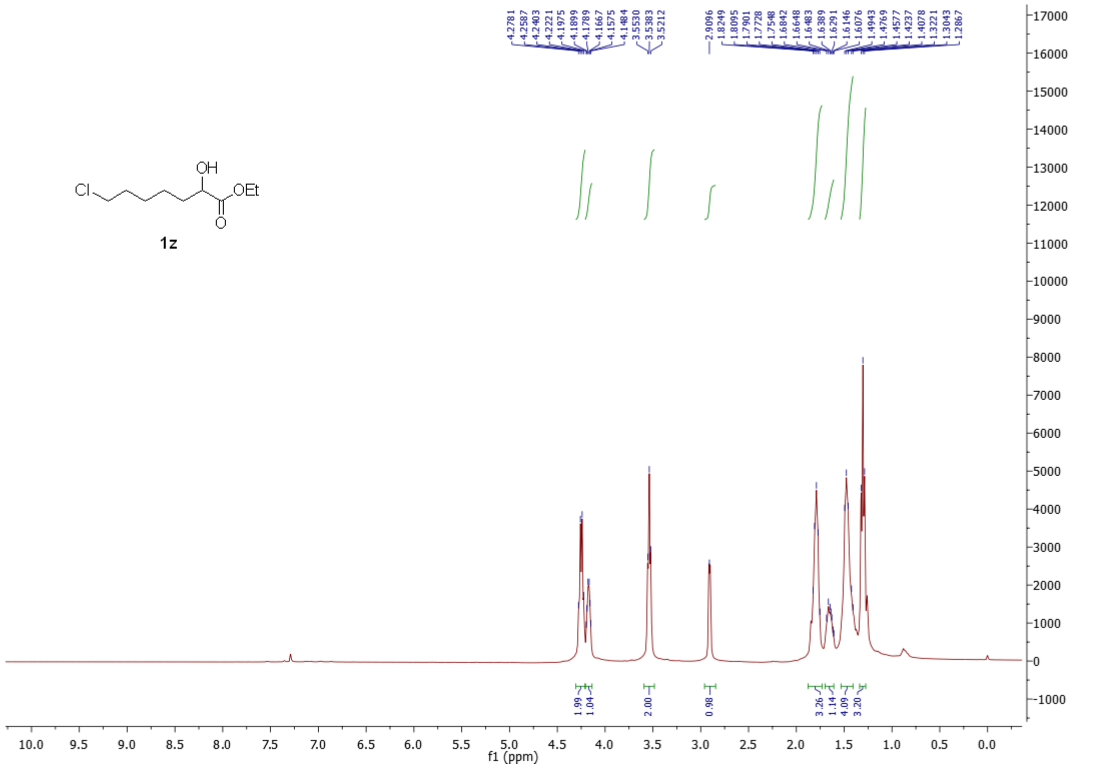

^{13}C NMR of Compound **1z** ($CDCl_3$, 100 MHz):

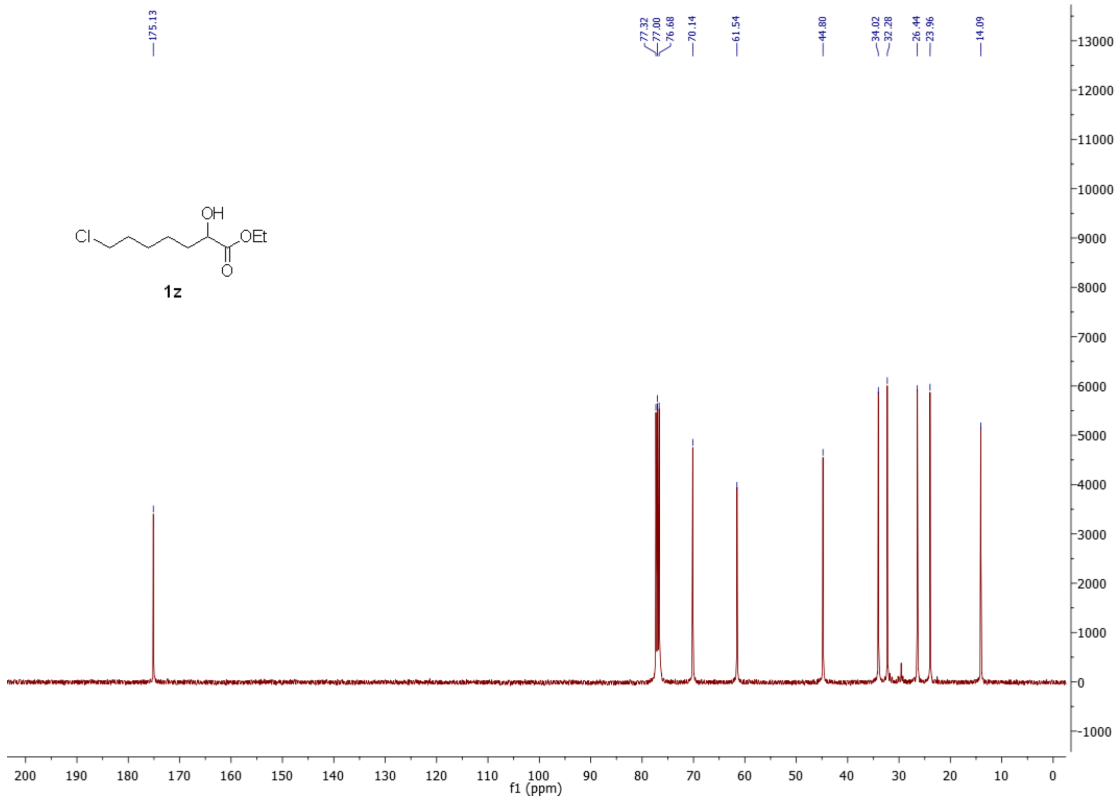

^{1}H NMR of Compound **1zc** ($CDCl_3$, 400 MHz):

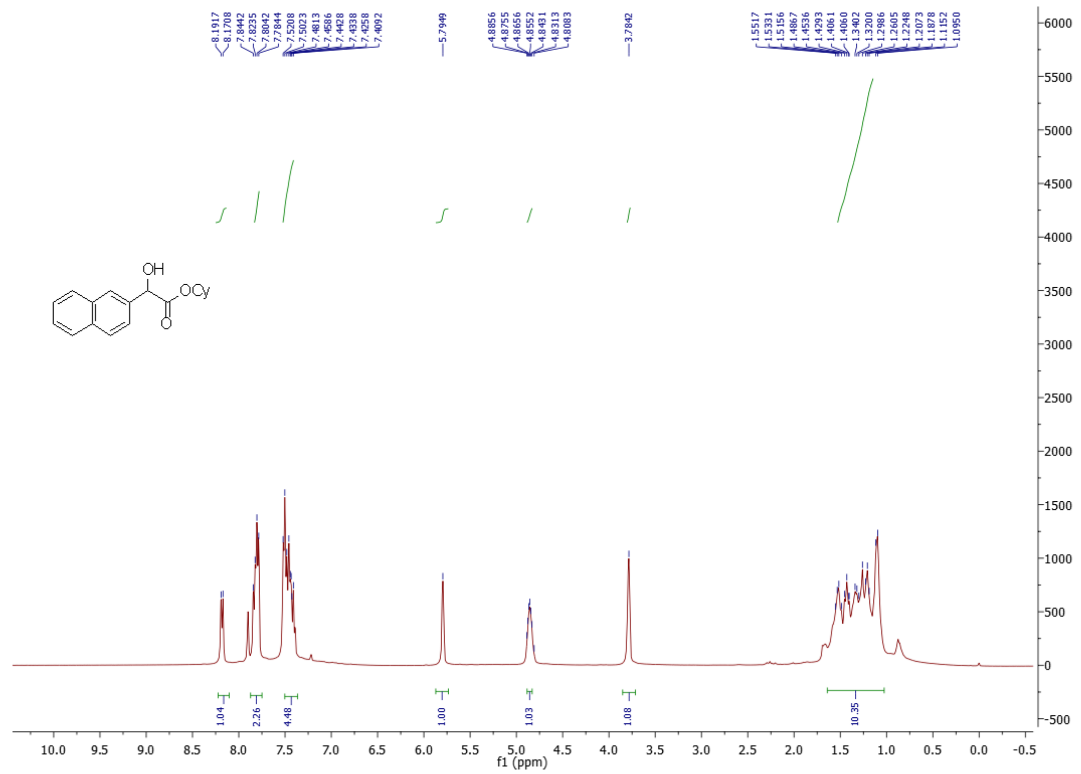

^{13}C NMR of Compound **1zc** ($CDCl_3$, 100 MHz):

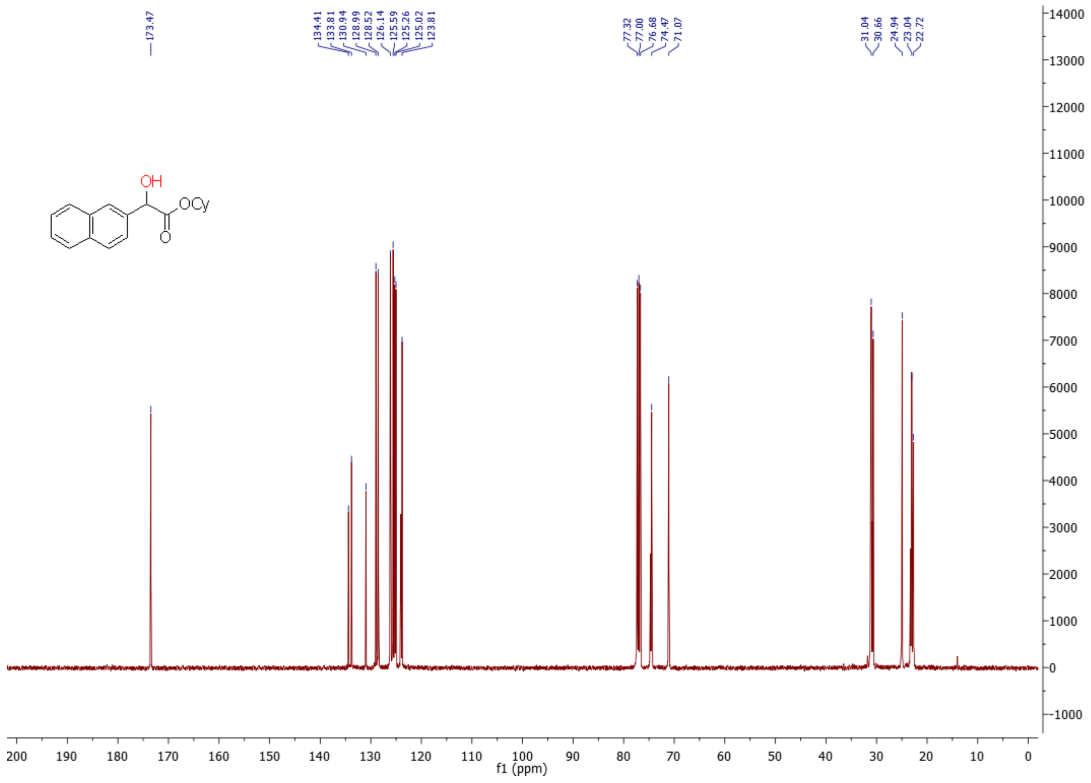

^{1}H NMR of Compound **1zd** ($CDCl_3$, 400 MHz):

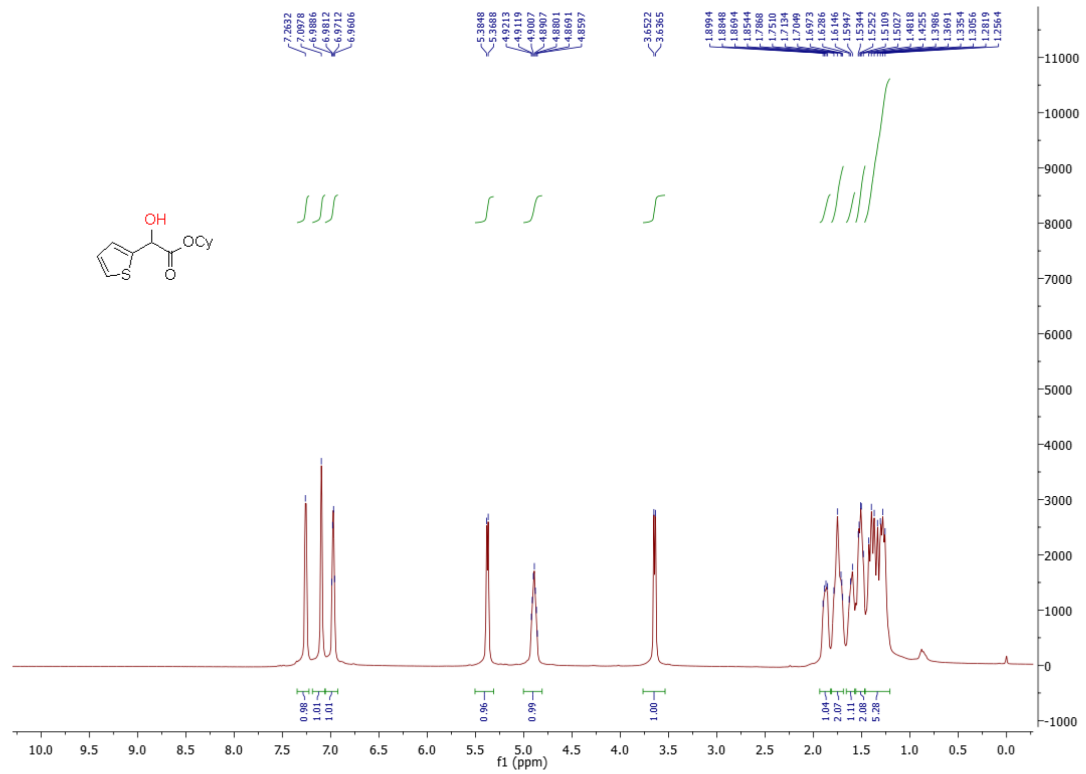

^{13}C NMR of Compound **1zd** ($CDCl_3$, 100 MHz):

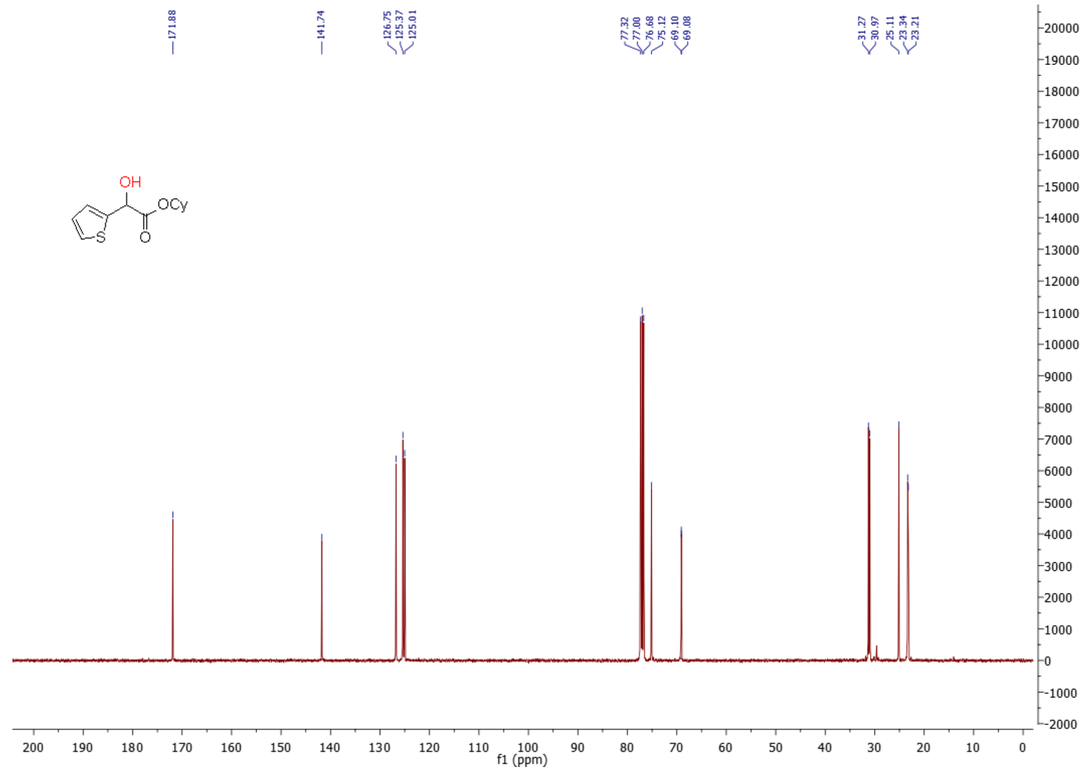

^{1}H NMR of Compound **1zf** ($CDCl_3$, 400 MHz):

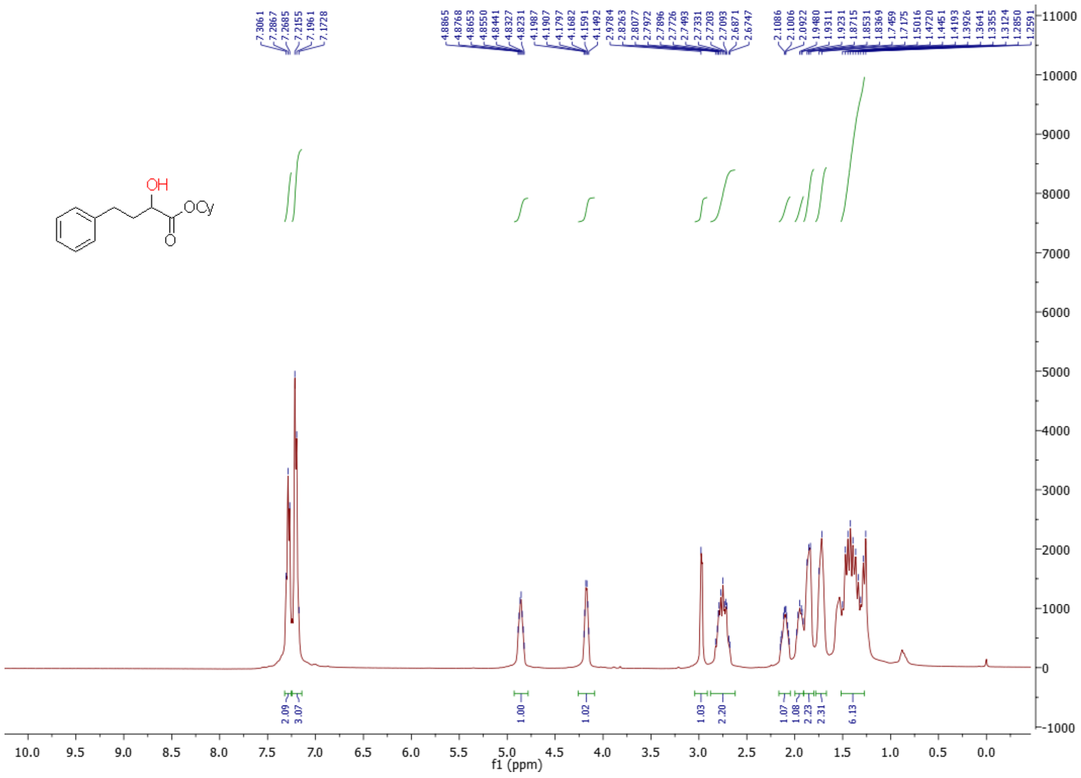

^{13}C NMR of Compound **1zf** ($CDCl_3$, 100 MHz):

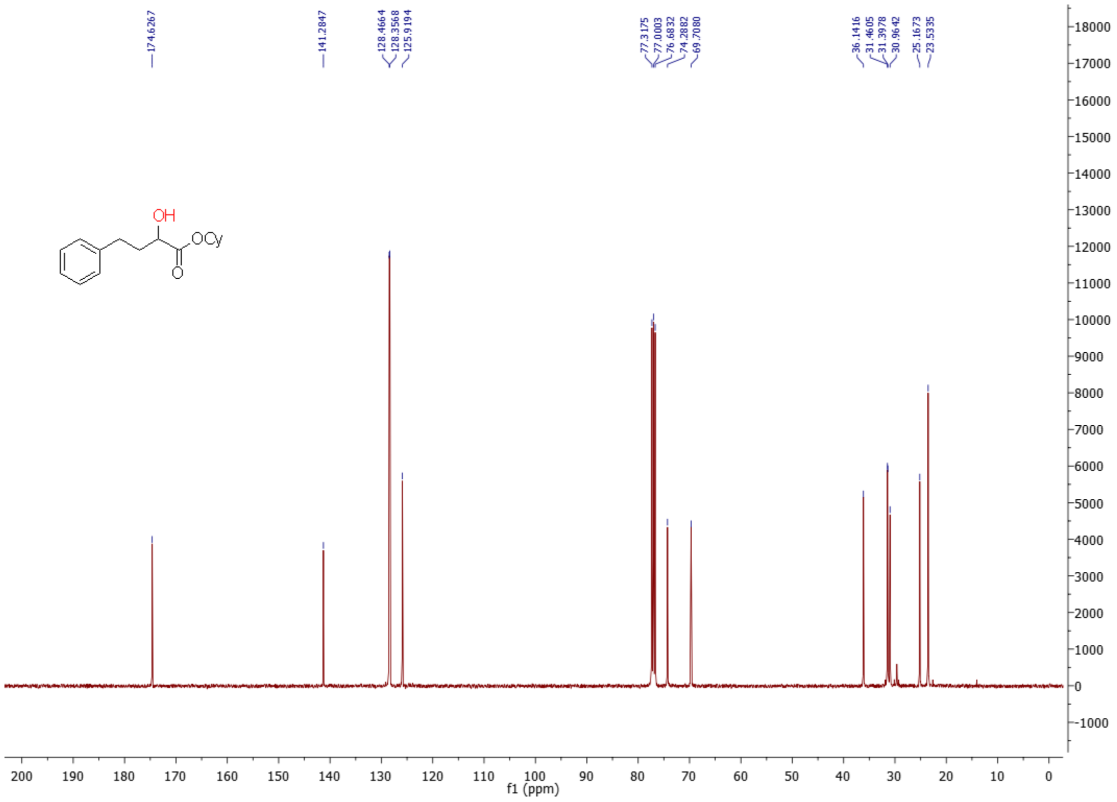

^{1}H NMR of Compound **1zg** ($CDCl_3$, 400 MHz):

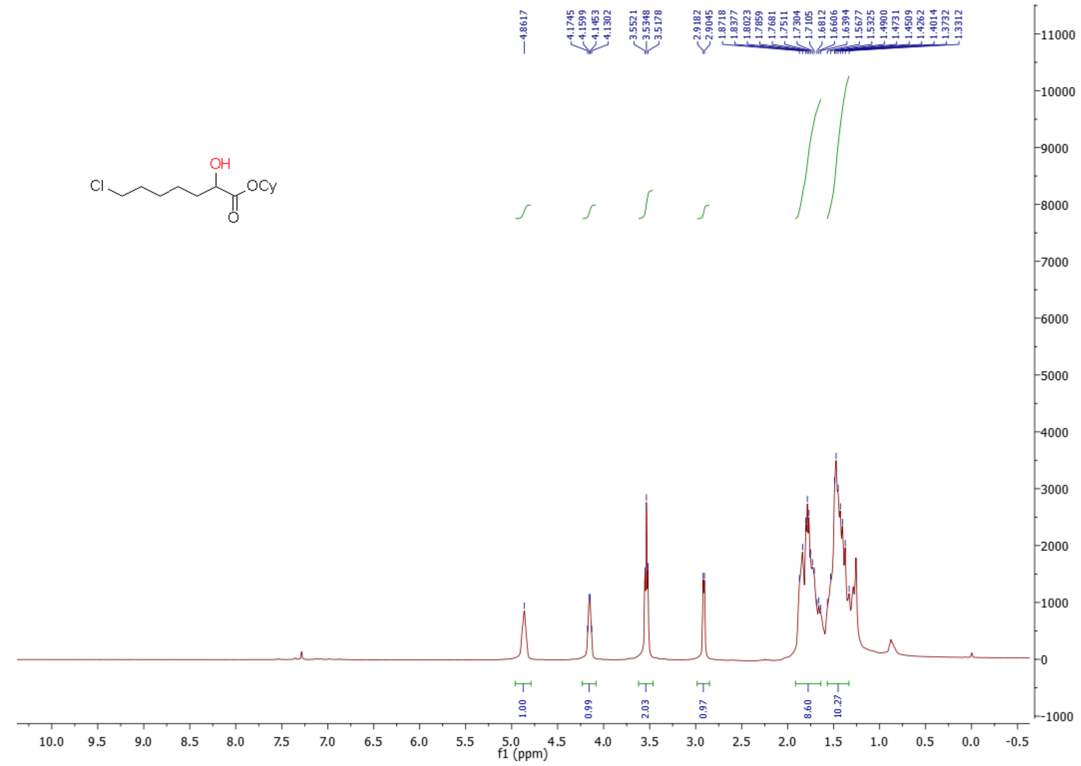

^{13}C NMR of Compound **1zg** ($CDCl_3$, 100 MHz):

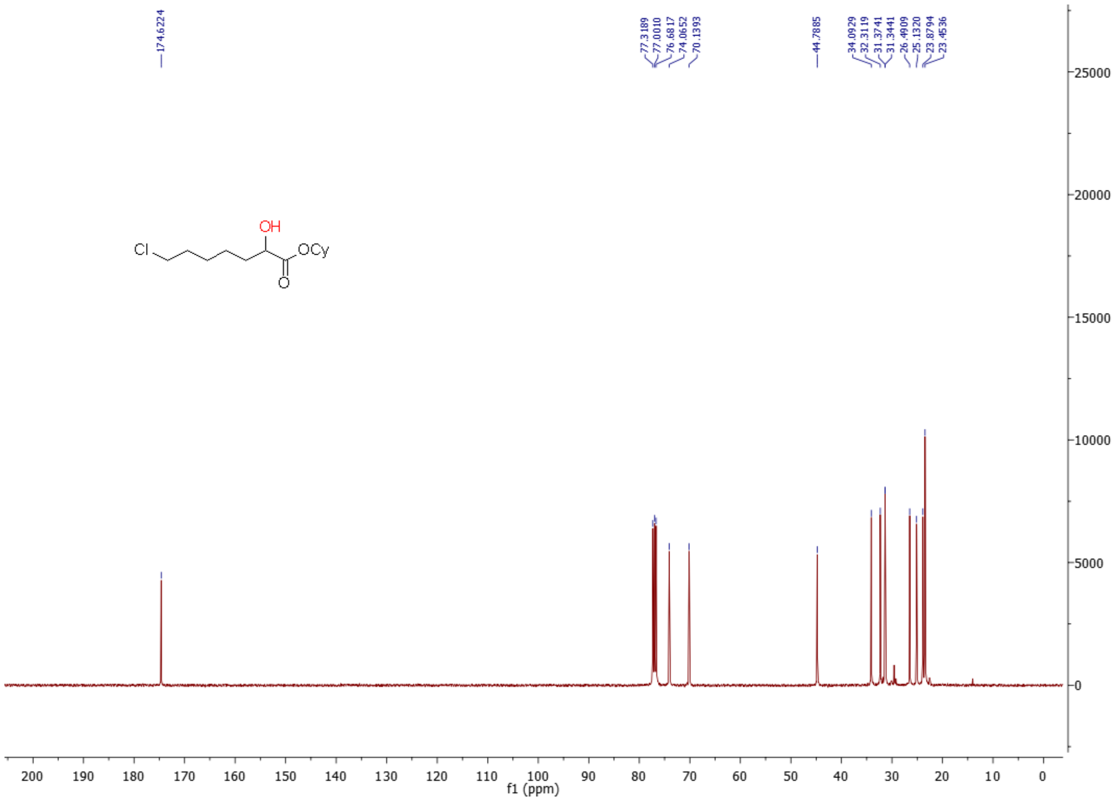

^{1}H NMR of **Mo-1 complex** ($CDCl_3$, 400 MHz):

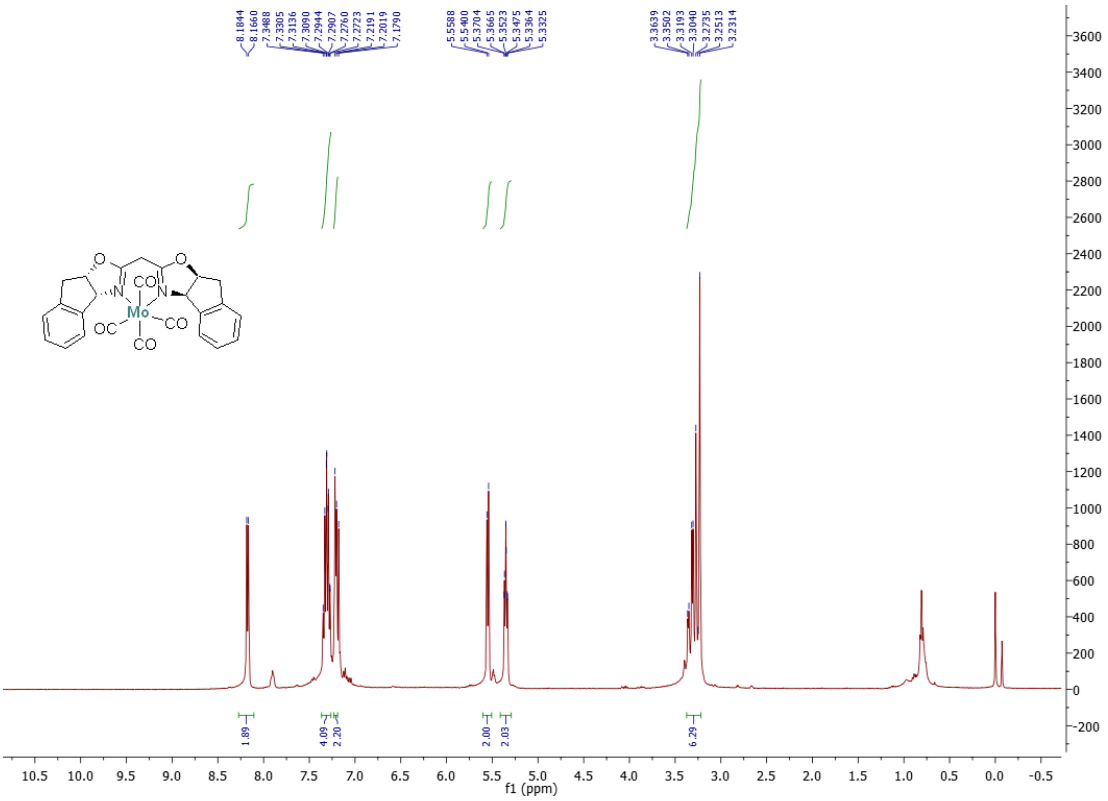

^{13}C NMR of **Mo-1 complex** ($CDCl_3$, 100 MHz):

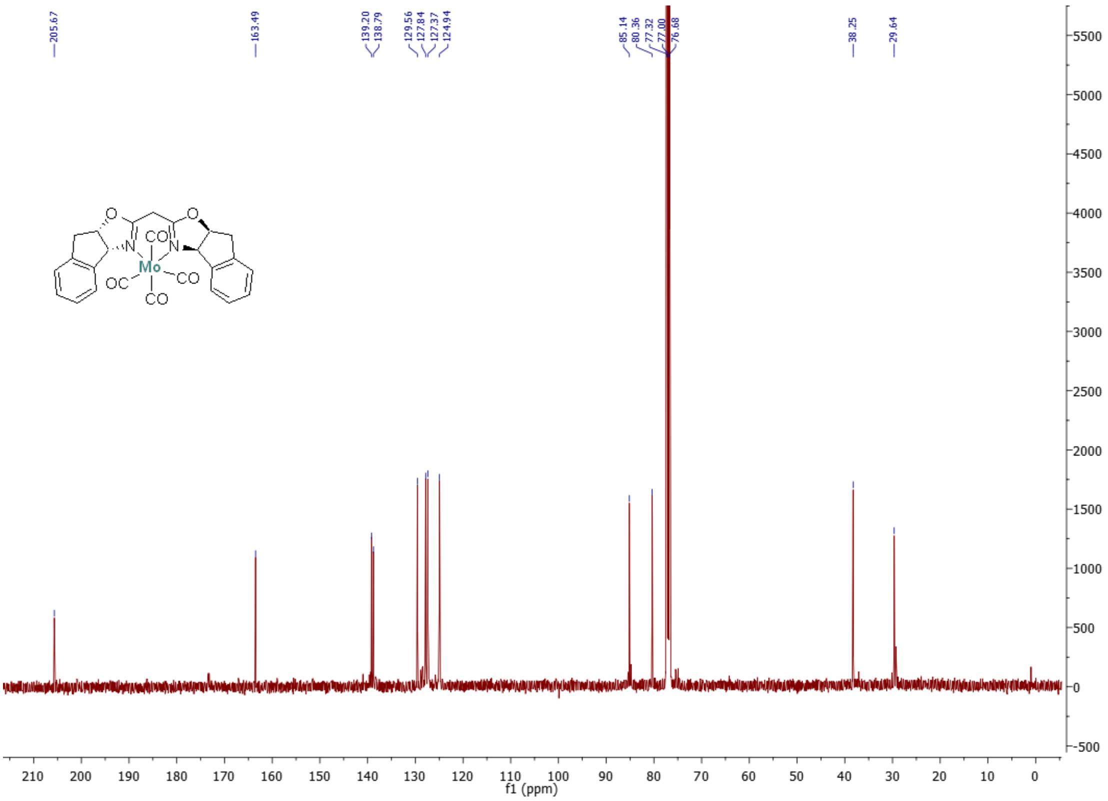

^{1}H NMR of **ligand-b** ($CDCl_3$, 400 MHz):

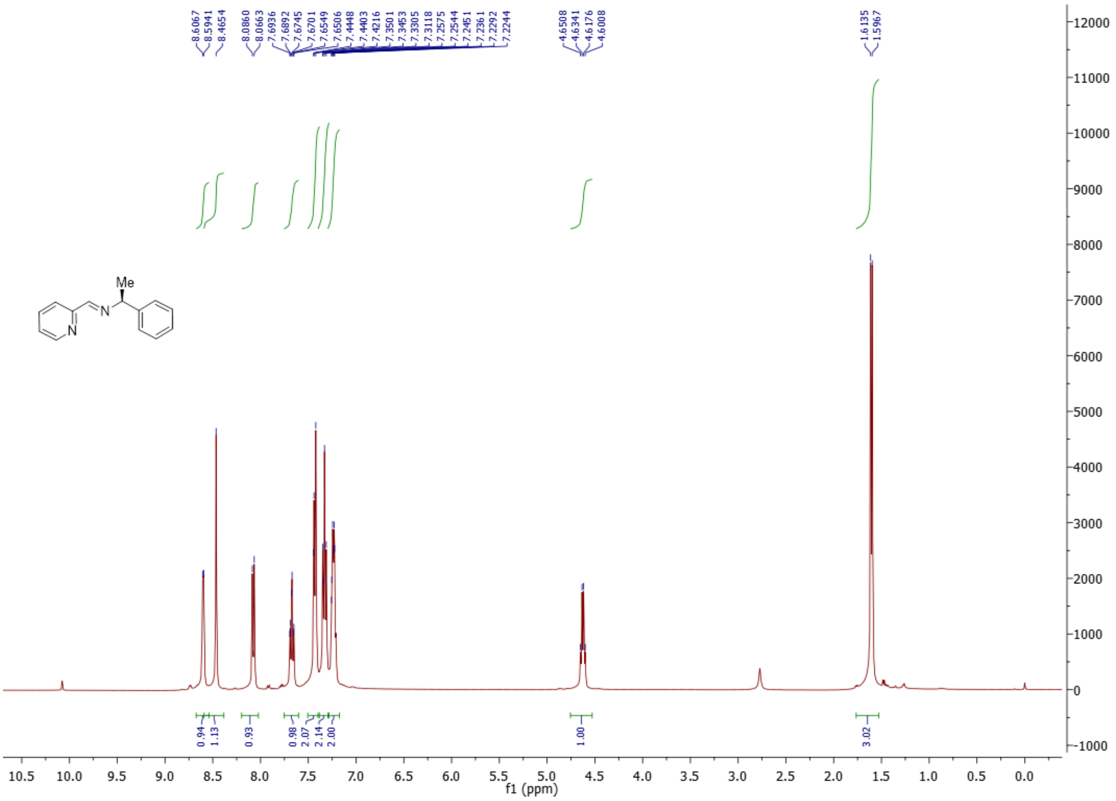

^{13}C NMR of **ligand-b** ($CDCl_3$, 100 MHz):

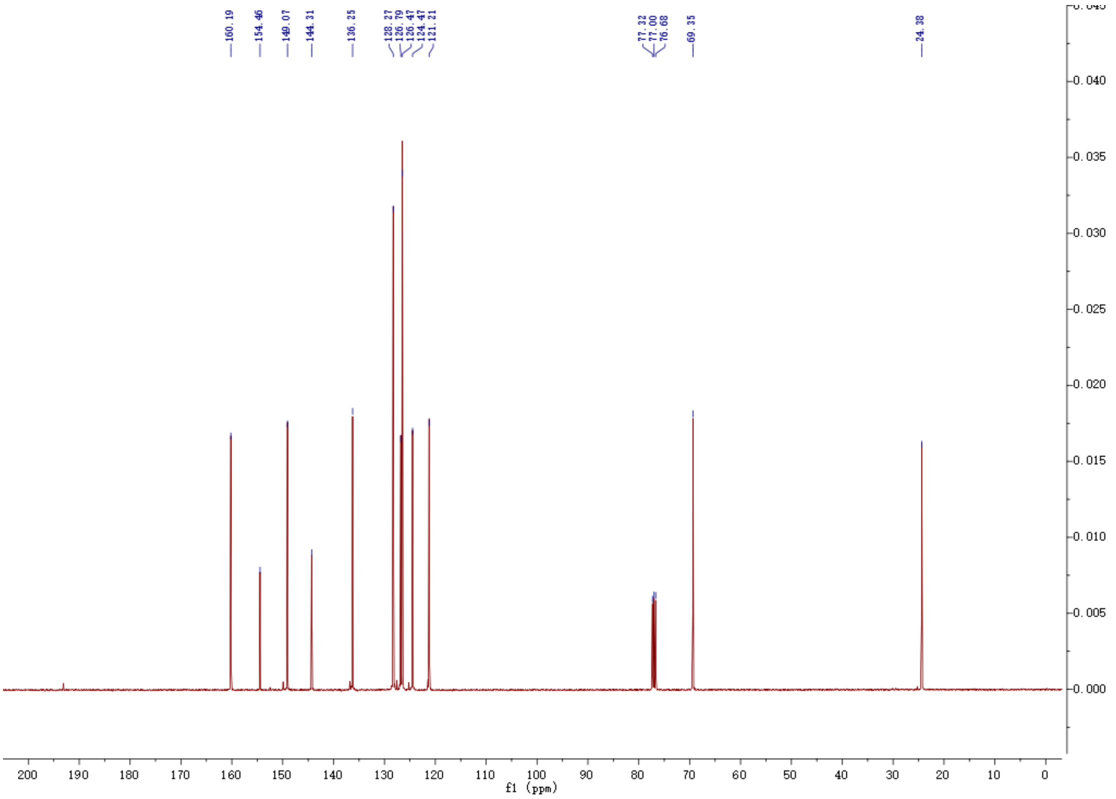

^{1}H NMR of **Mo-2 complex** ($CDCl_3$, 400 MHz):

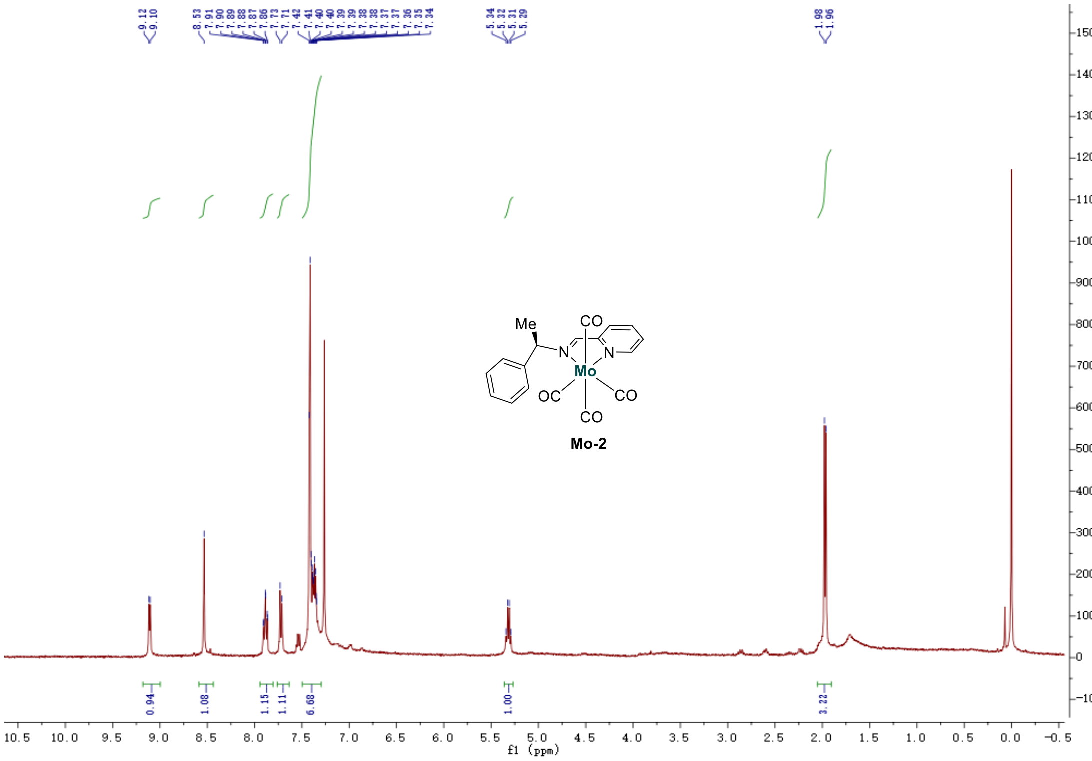

^{13}C NMR of **Mo-2 complex** ($CDCl_3$, 100 MHz):

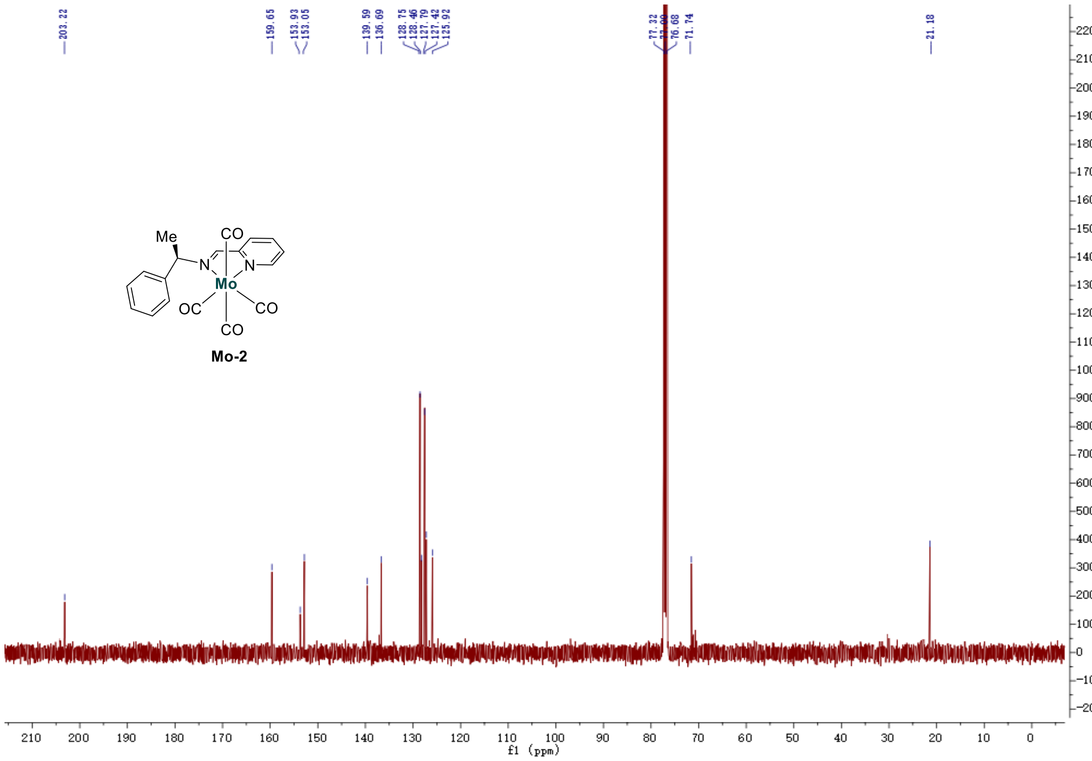

^{1}H NMR of **ligand-c** ($CDCl_3$, 400 MHz):

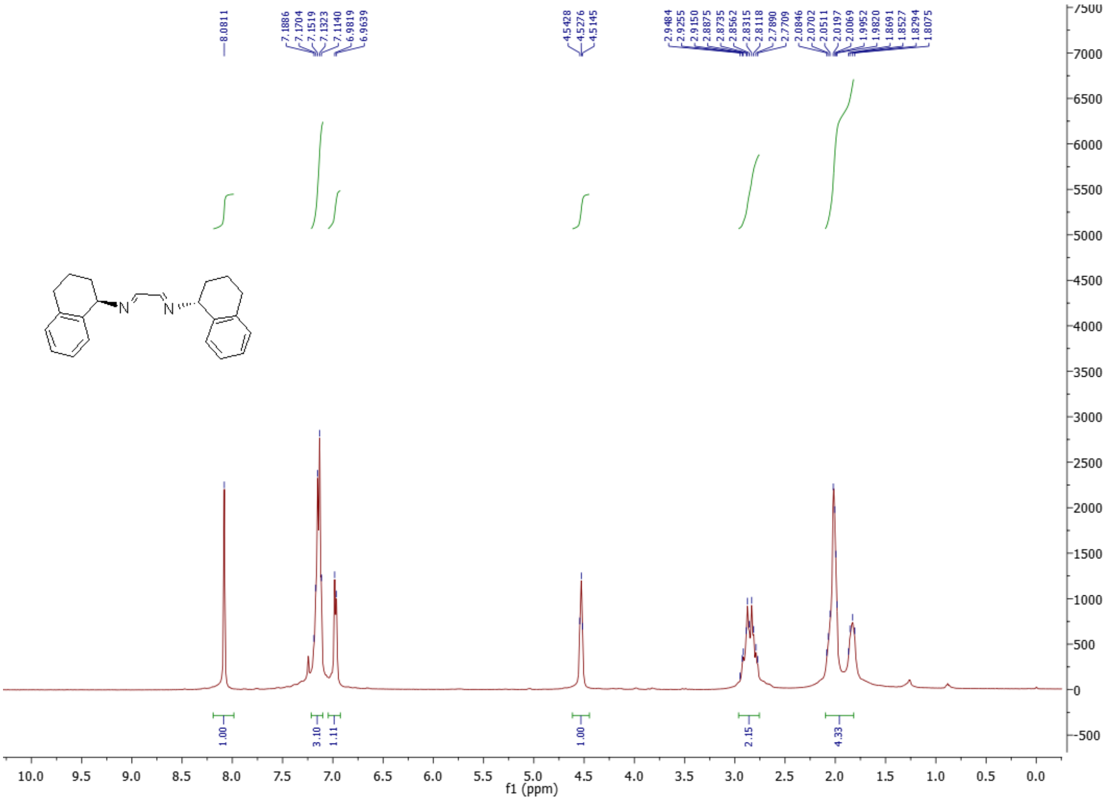

8.

^{13}C NMR of **ligand-c** ($CDCl_3$, 100 MHz):

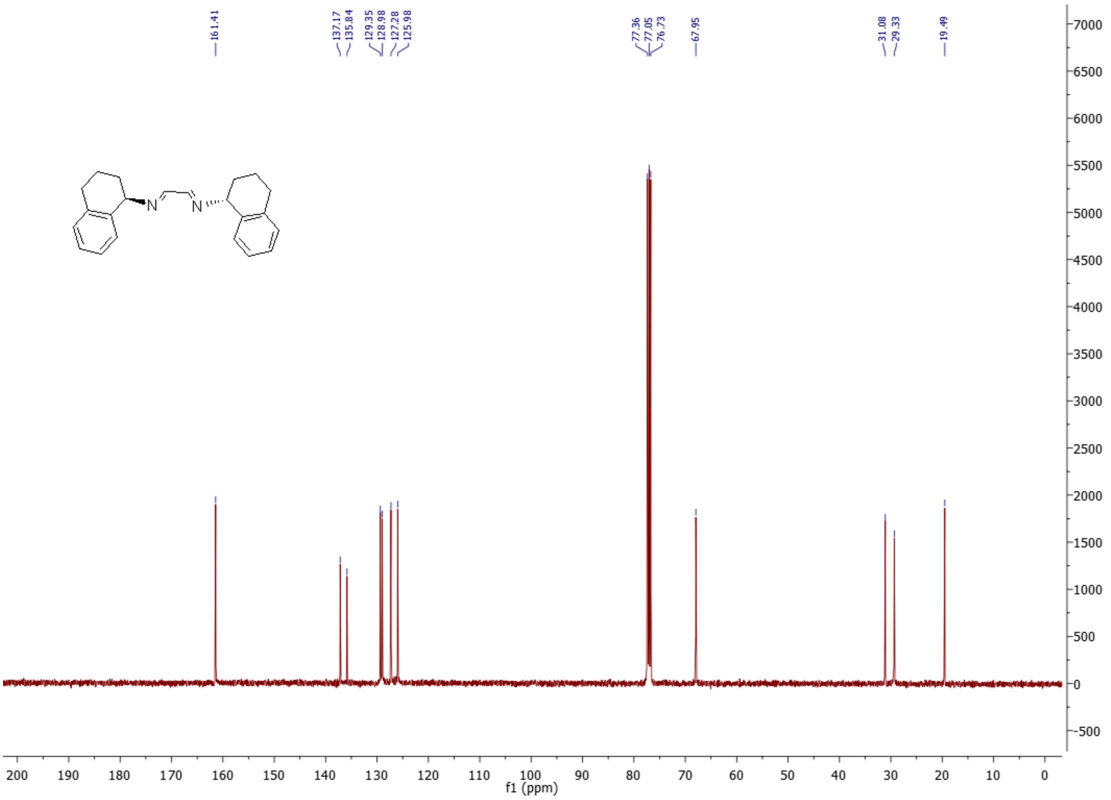

^{1}H NMR of **Mo-3 complex** ($CDCl_3$, 400 MHz):

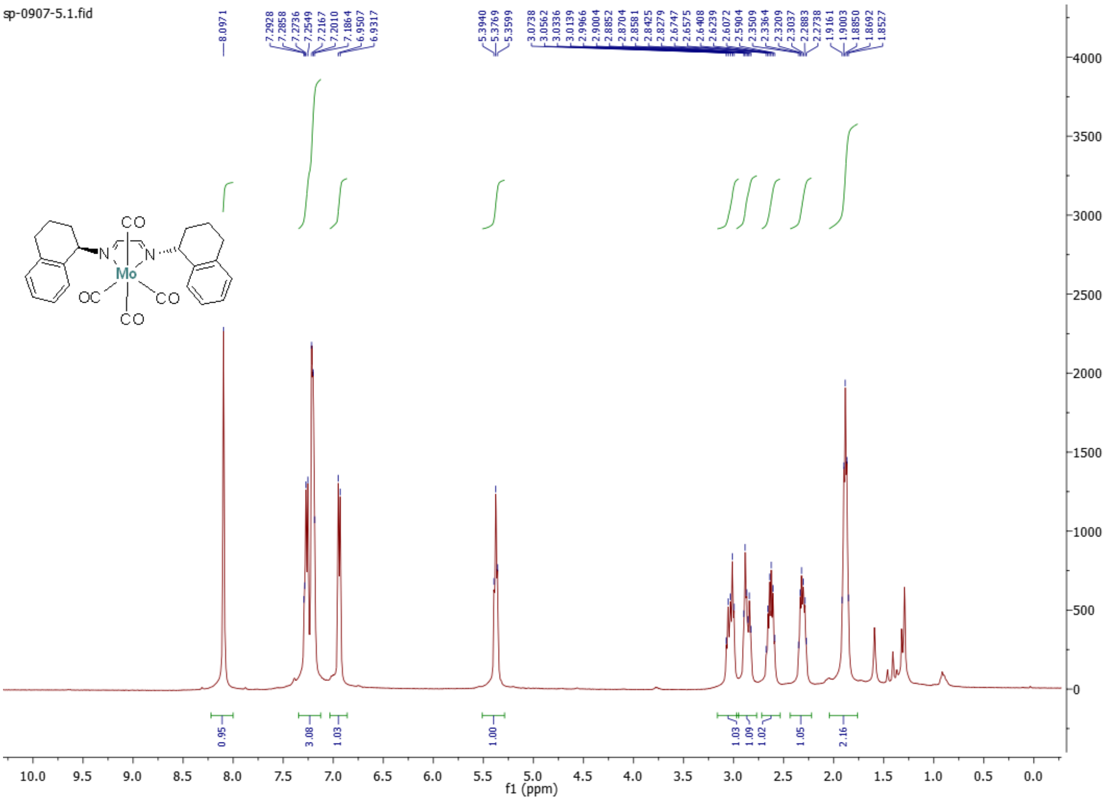

^{13}C NMR of **Mo-3 complex** ($CDCl_3$, 100 MHz):

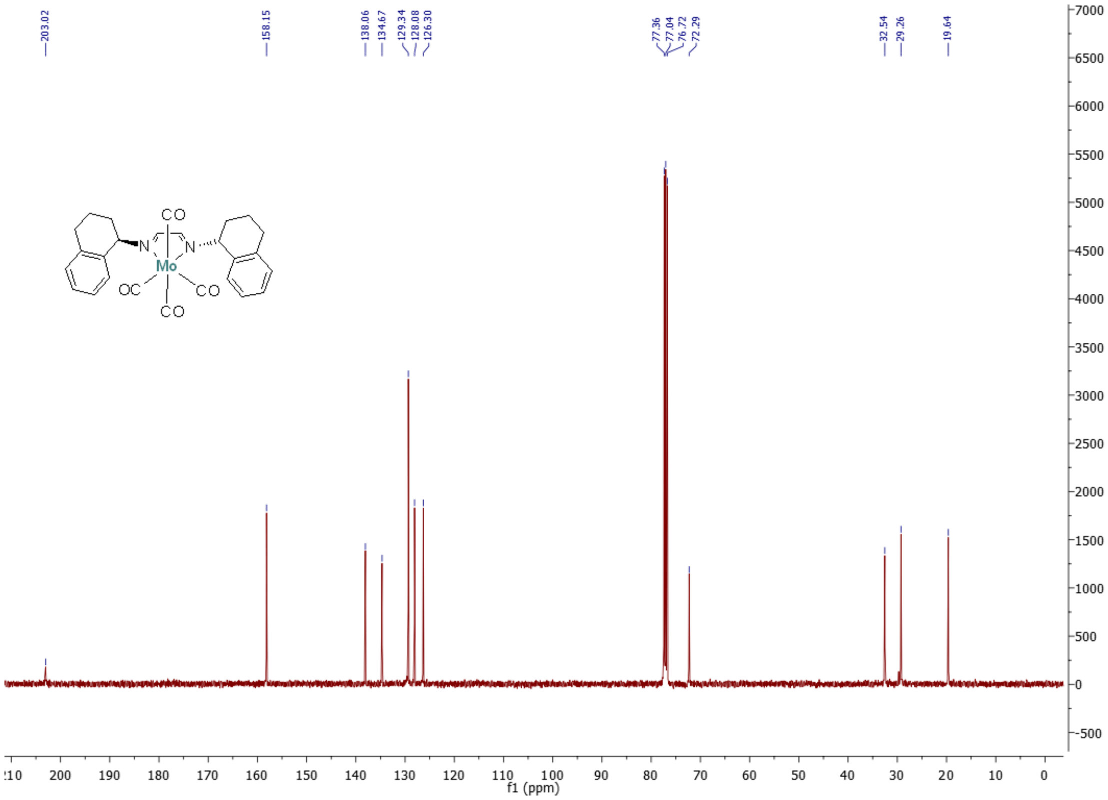

^{1}H NMR of **ligand-d** ($CDCl_3$, 400 MHz):

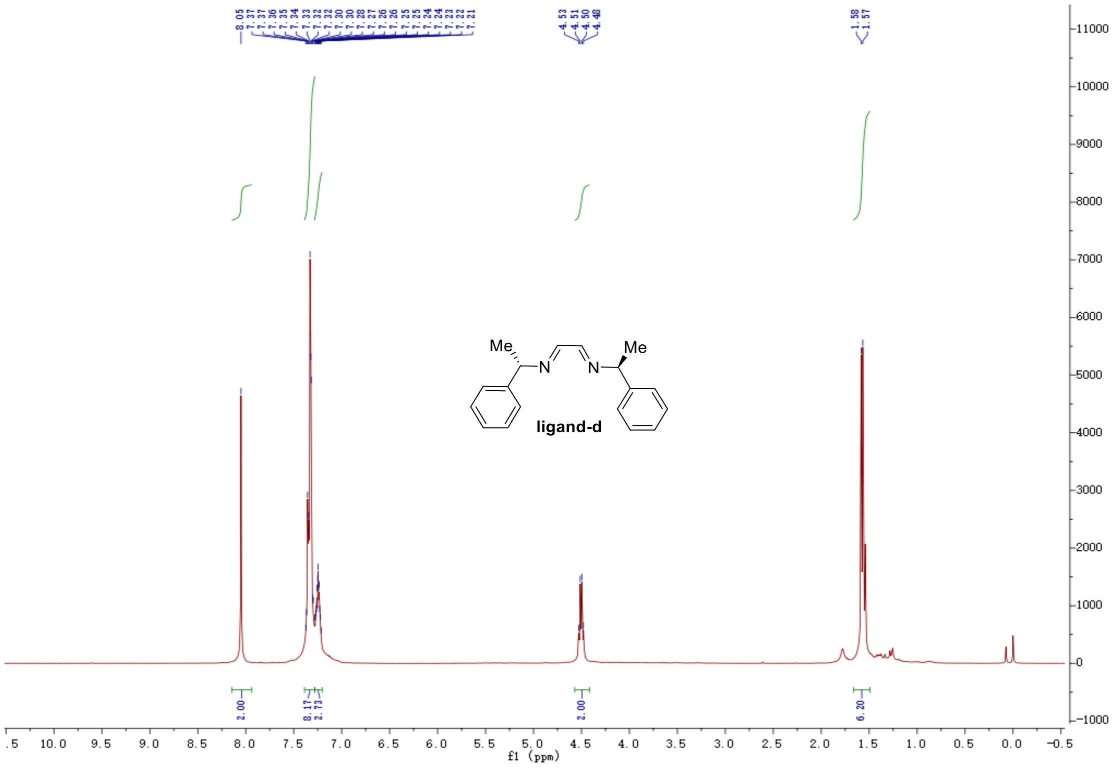

^{1}H NMR of **ligand-d** ($CDCl_3$, 400 MHz):

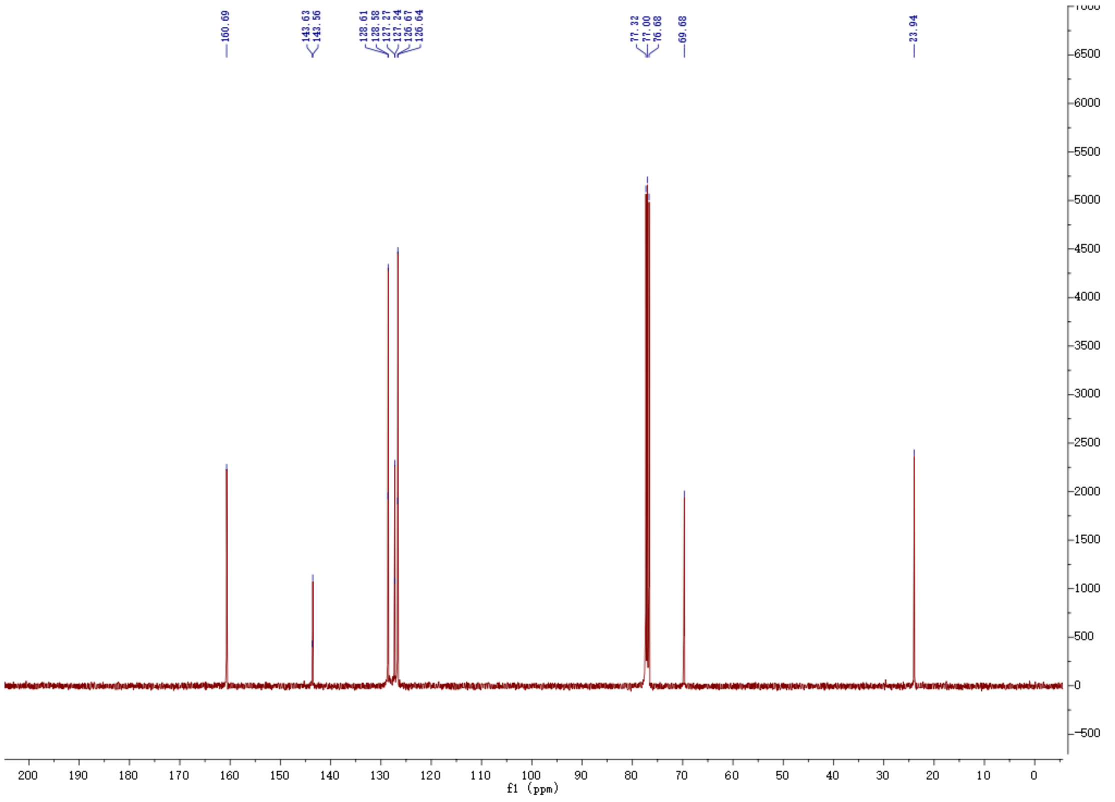

^{1}H NMR of **Mo-4 complex** ($CDCl_3$, 400 MHz):

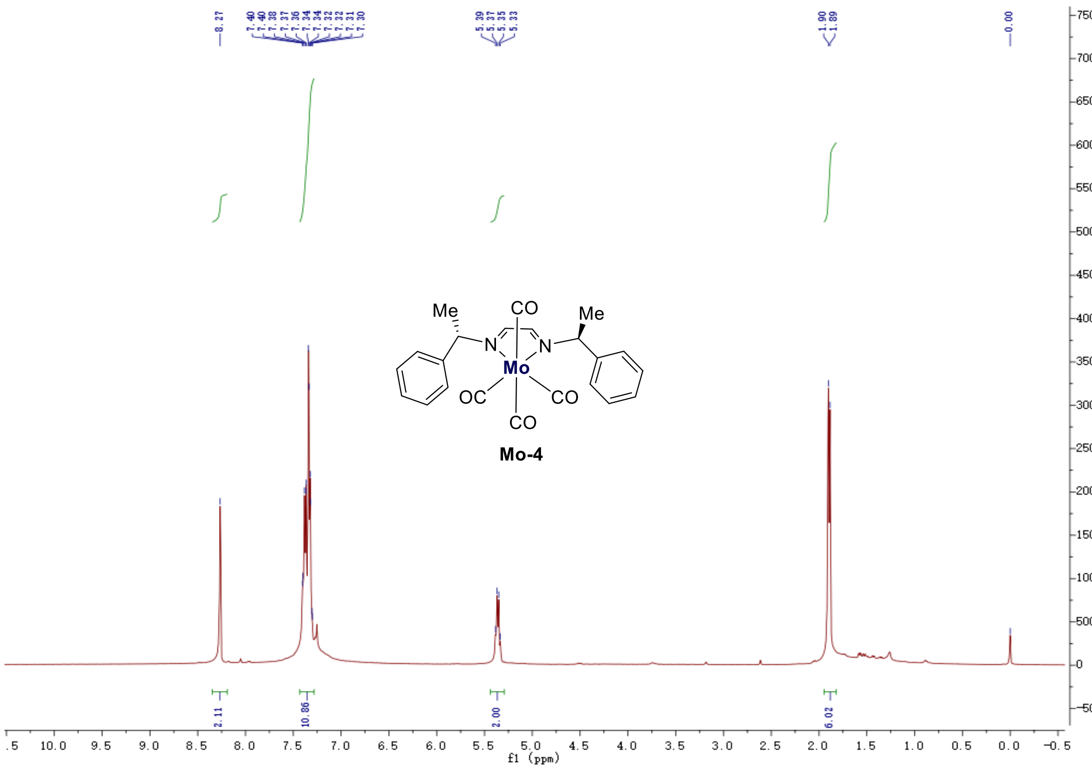

^{1}H NMR of **Mo-4 complex** ($CDCl_3$, 400 MHz):

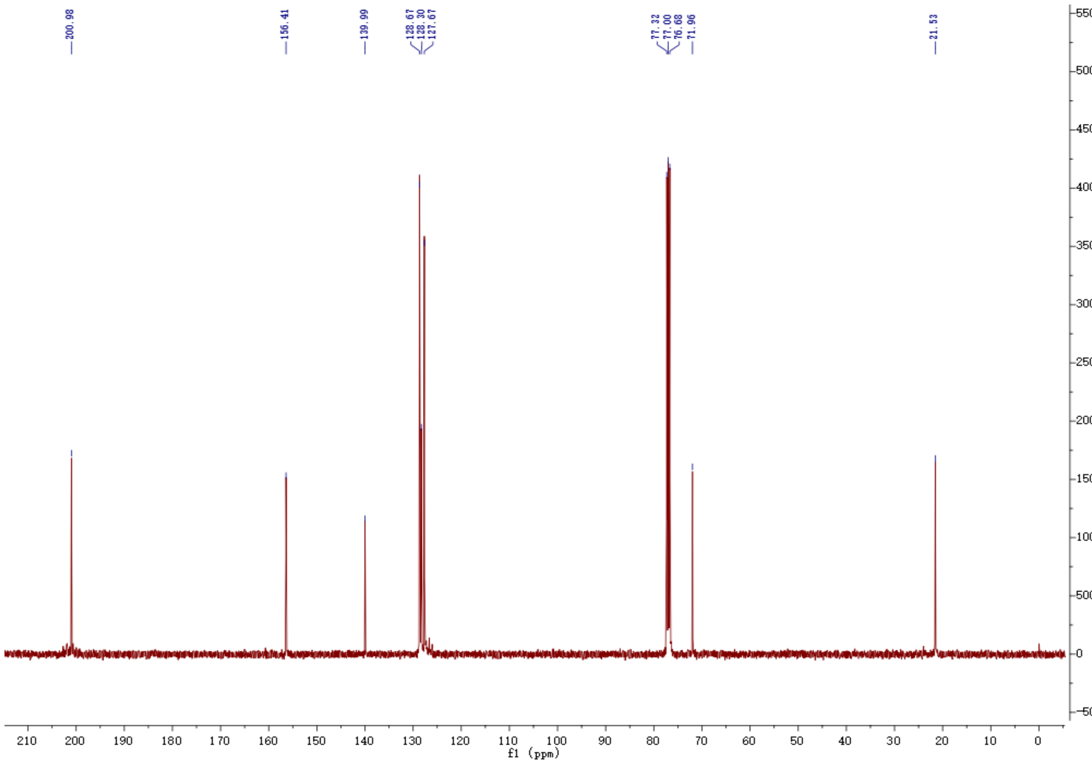

^{1}H NMR of **ligand-e** ($CDCl_3$, 400 MHz):

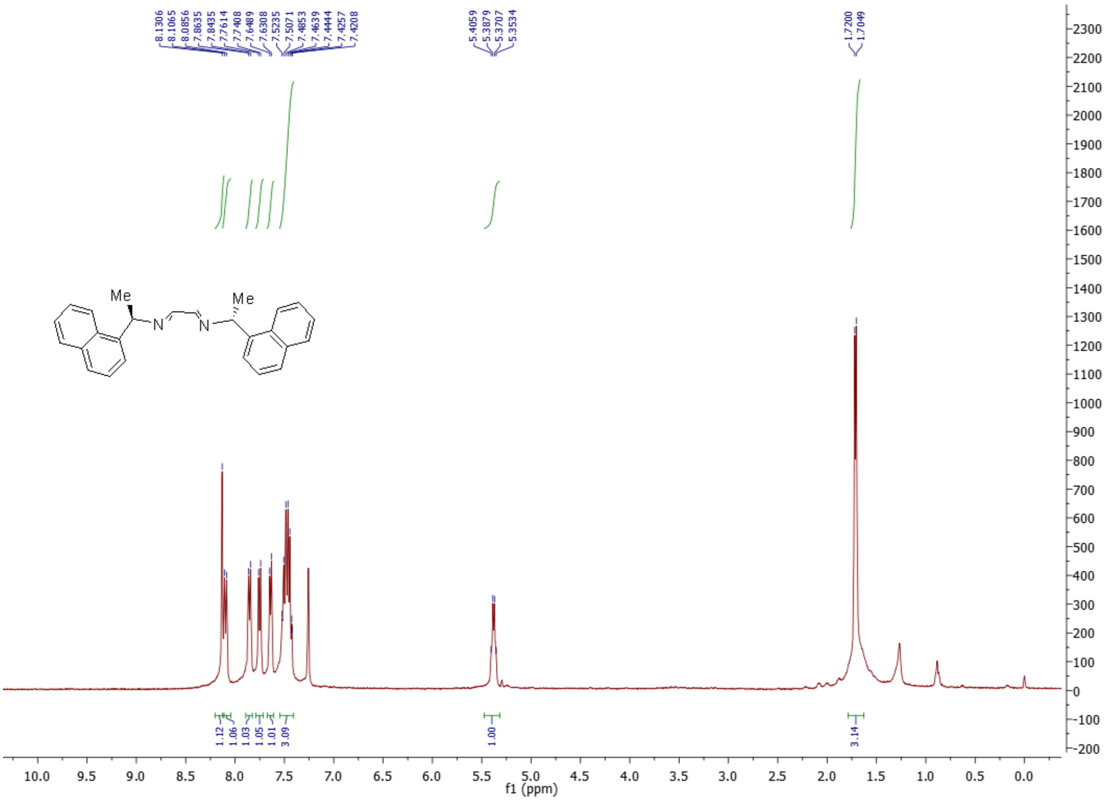

^{1}H NMR of **ligand-e** ($CDCl_3$, 400 MHz):

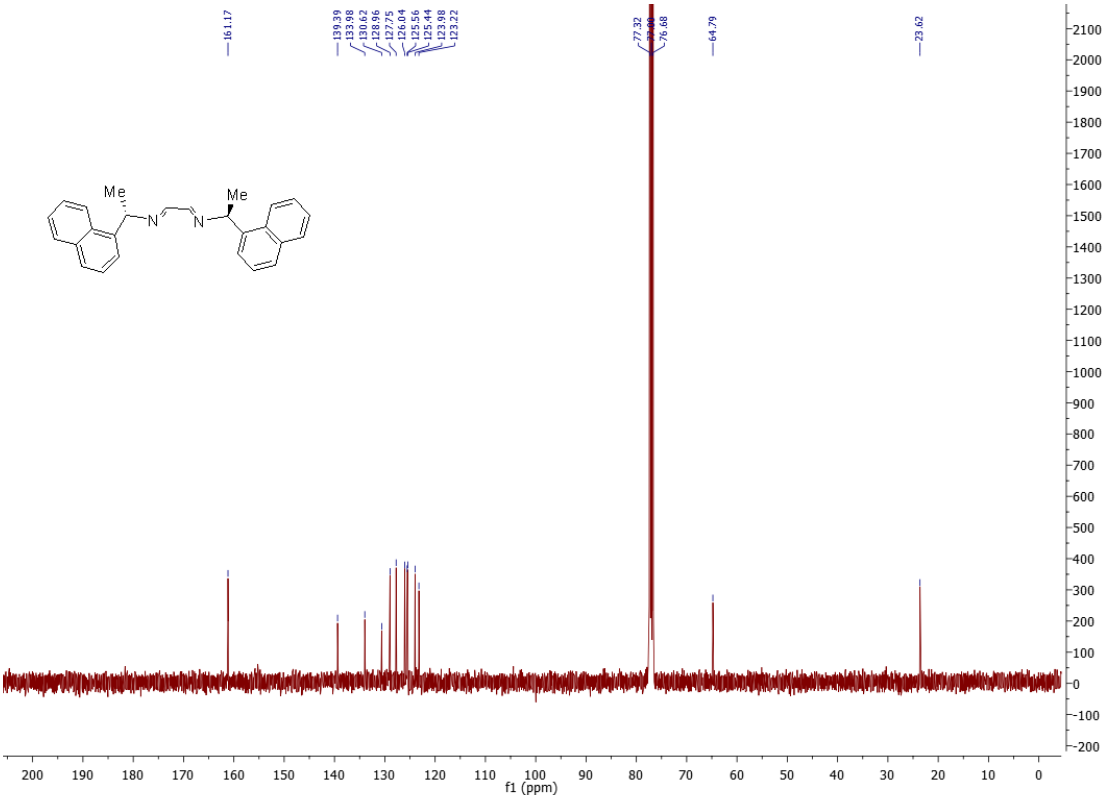

^{1}H NMR of **Mo-5 complex** ($CDCl_3$, 400 MHz):

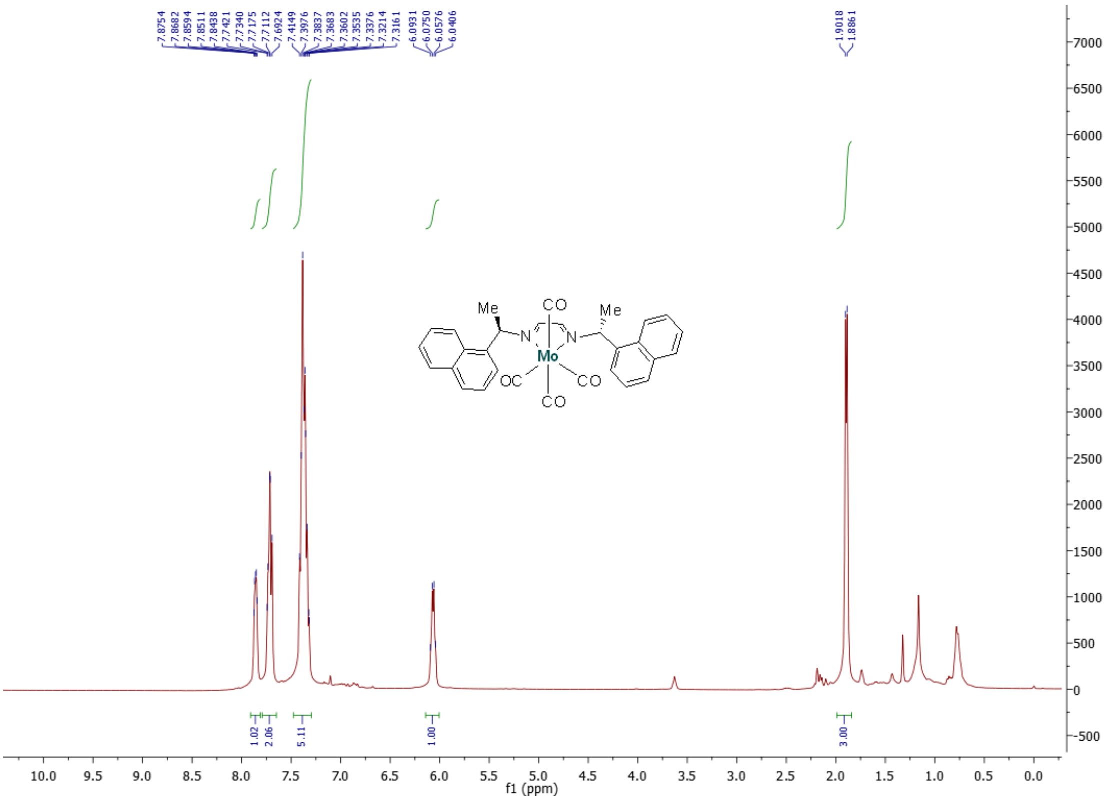

^{1}H NMR of **Mo-5 complex** ($CDCl_3$, 400 MHz):

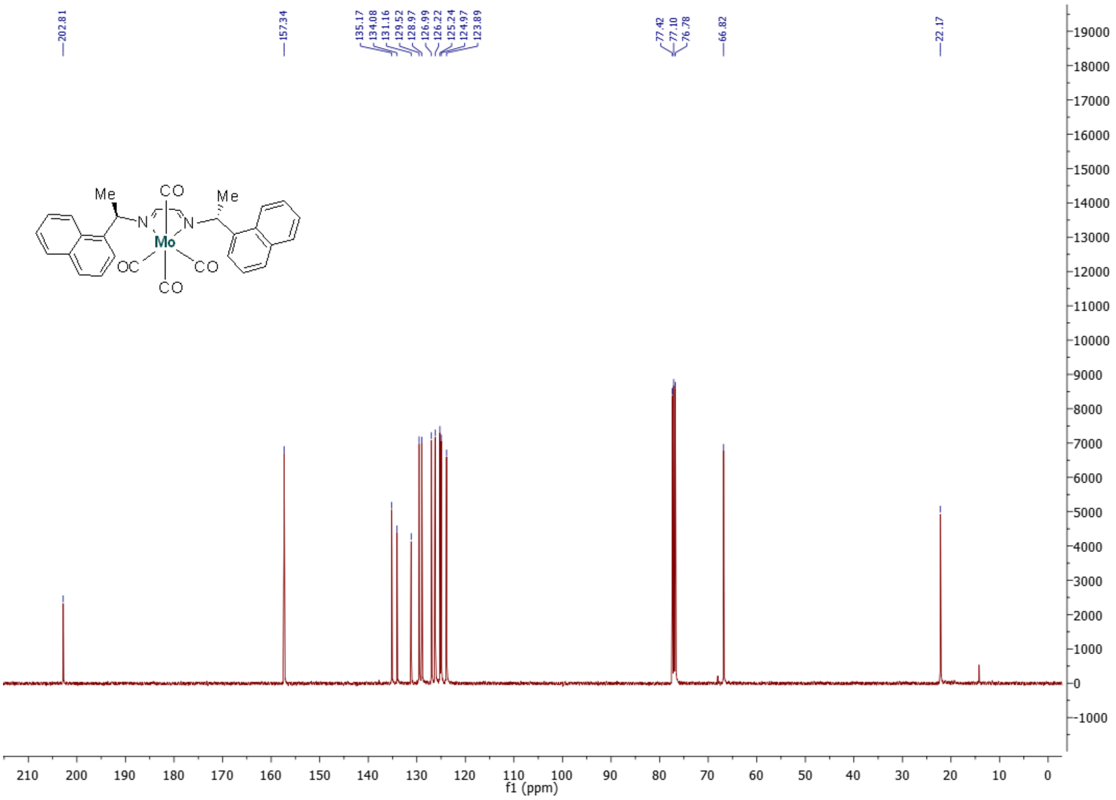

^{1}H NMR of Ellman's imine ($CDCl_3$, 400 MHz):

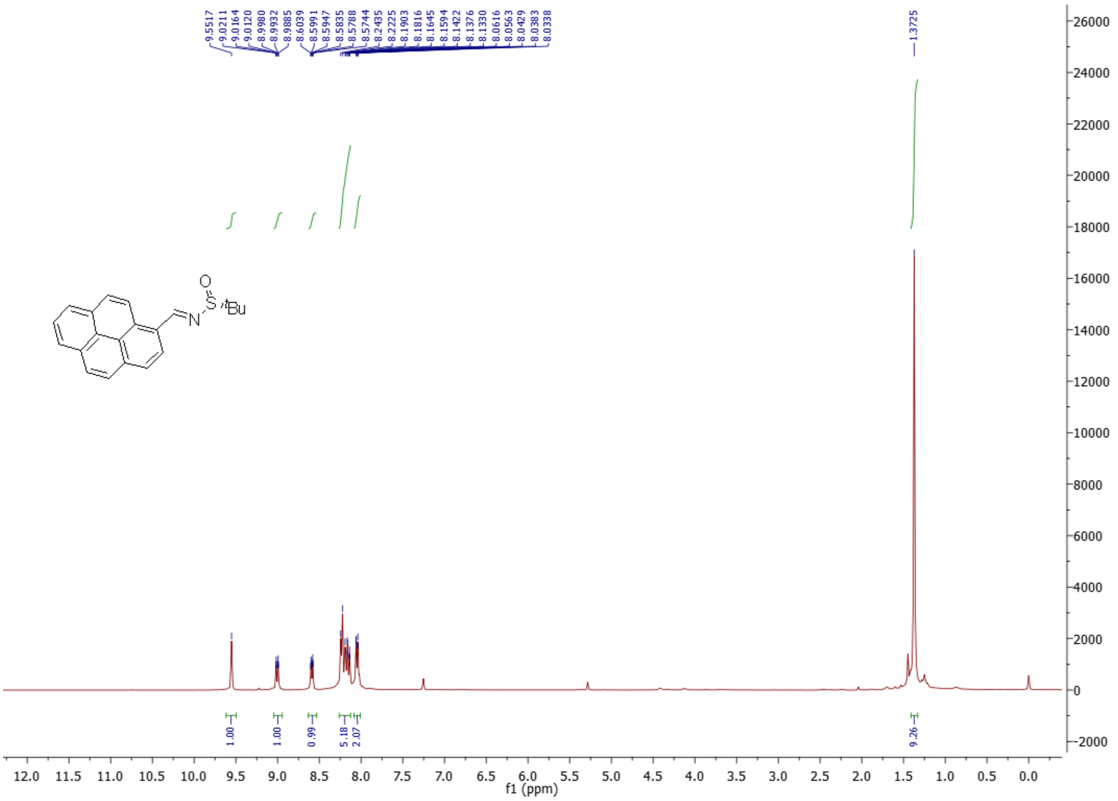

^{1}H NMR of Ellman's imine ($CDCl_3$, 400 MHz):

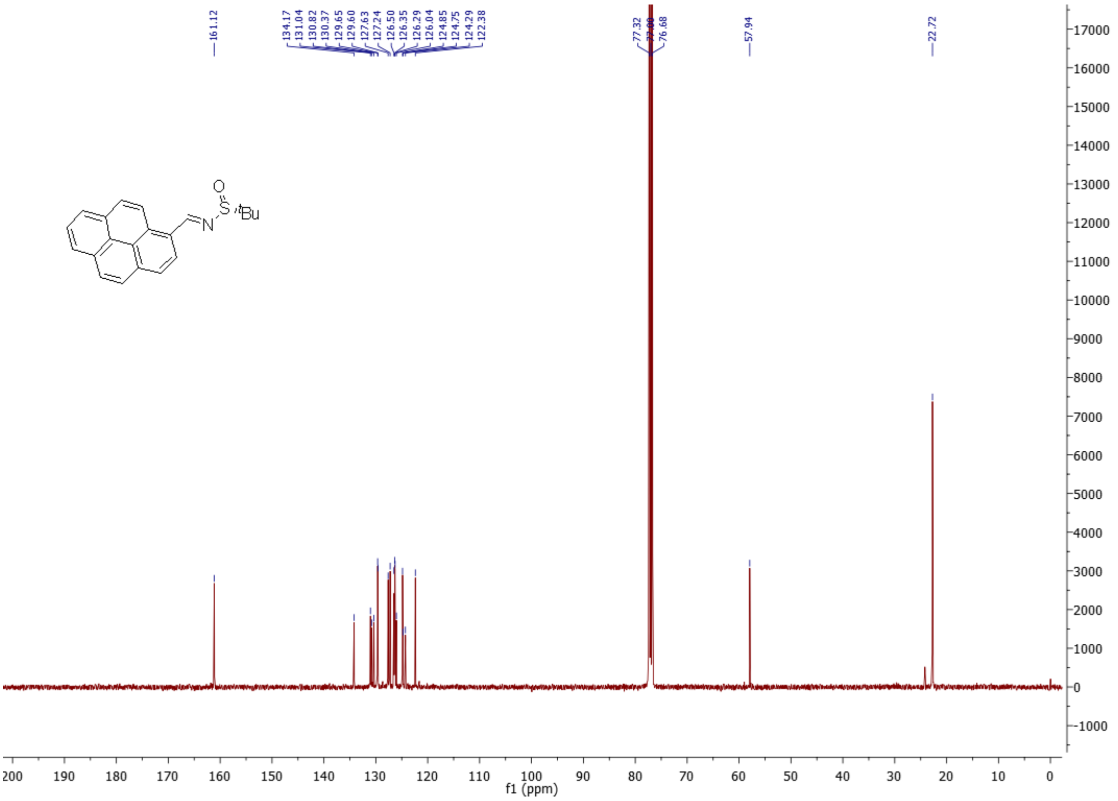

^{1}H NMR of protected amine ($CDCl_3$, 400 MHz):

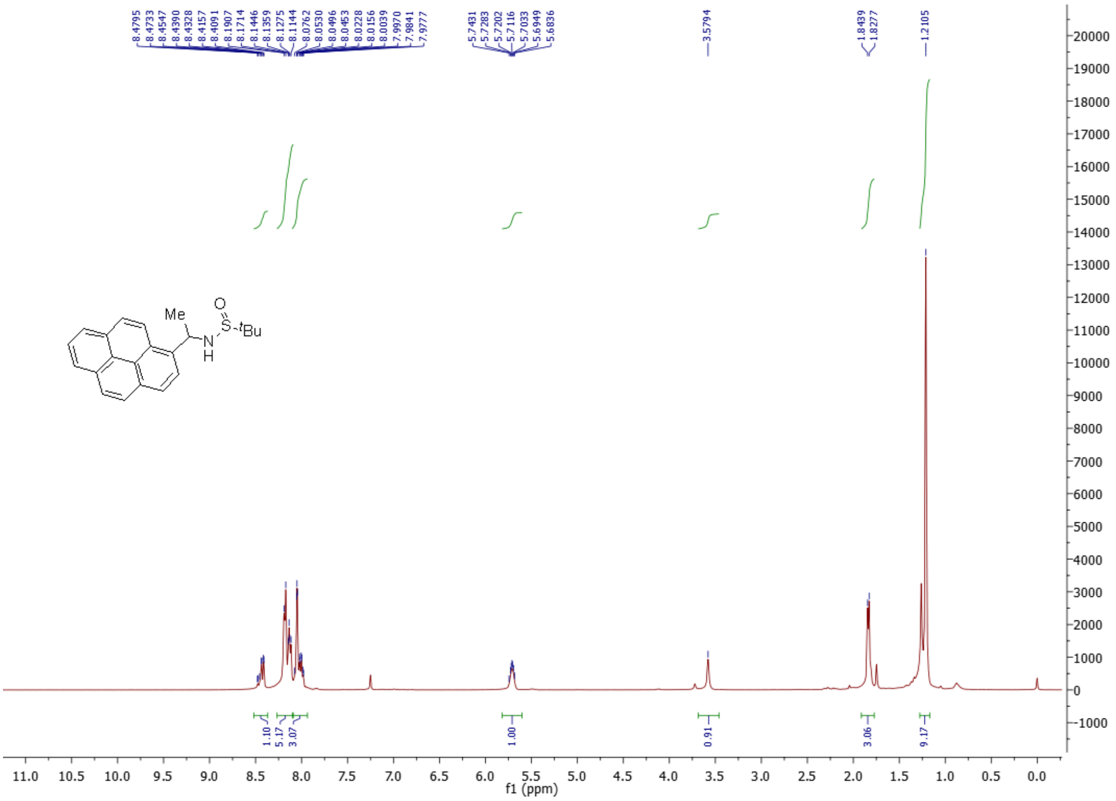

^{1}H NMR of protected amine ($CDCl_3$, 400 MHz):

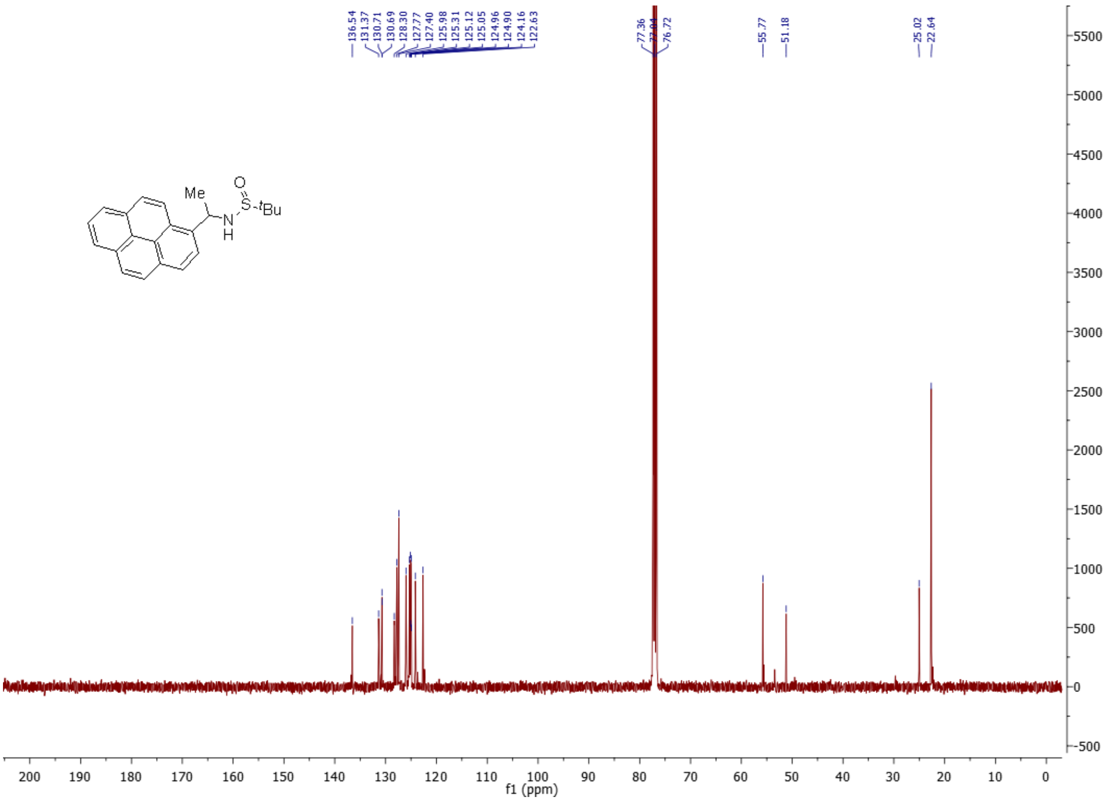

^{1}H NMR of unprotected amine ($CDCl_3$, 400 MHz):

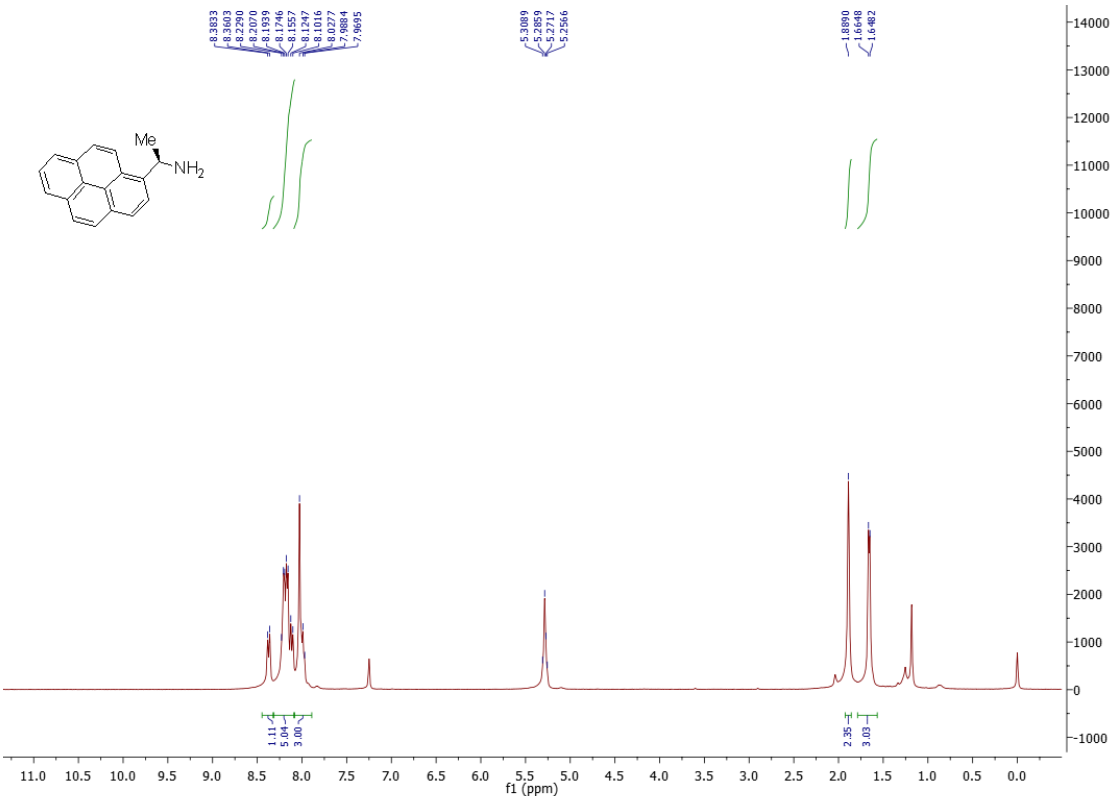

^{1}H NMR of unprotected amine ($CDCl_3$, 400 MHz):

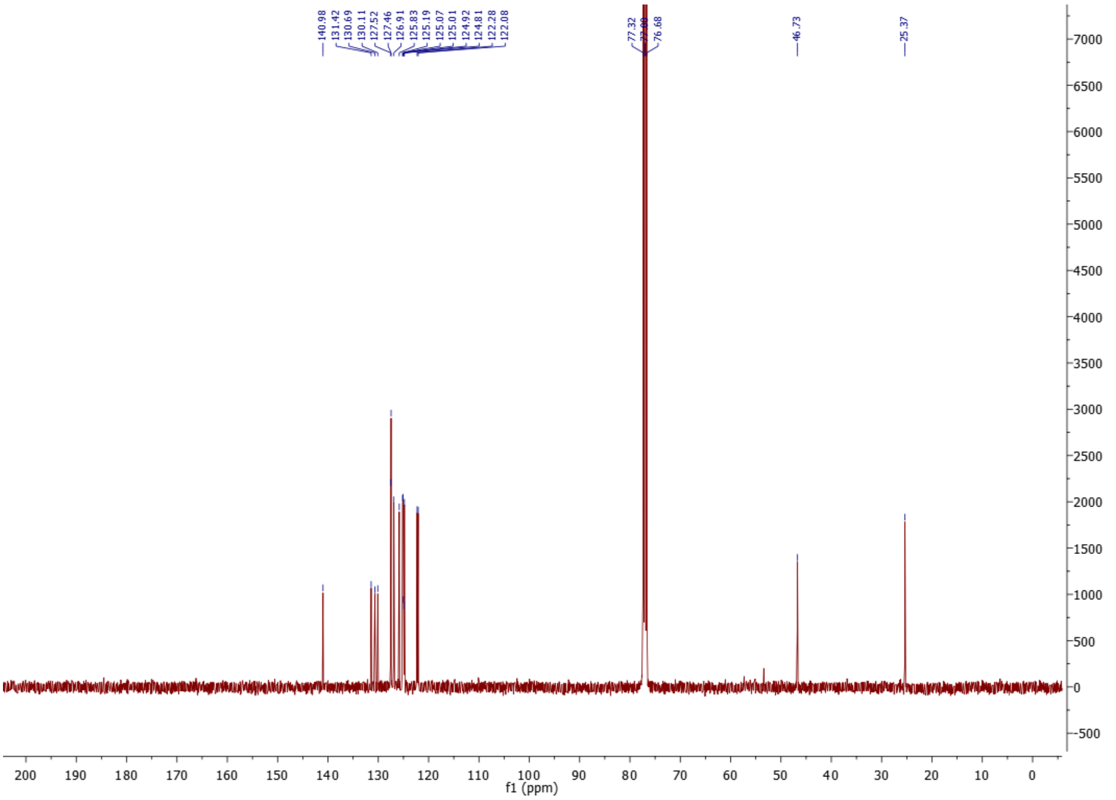

^{1}H NMR of **ligand-f** ($CDCl_3$, 400 MHz):

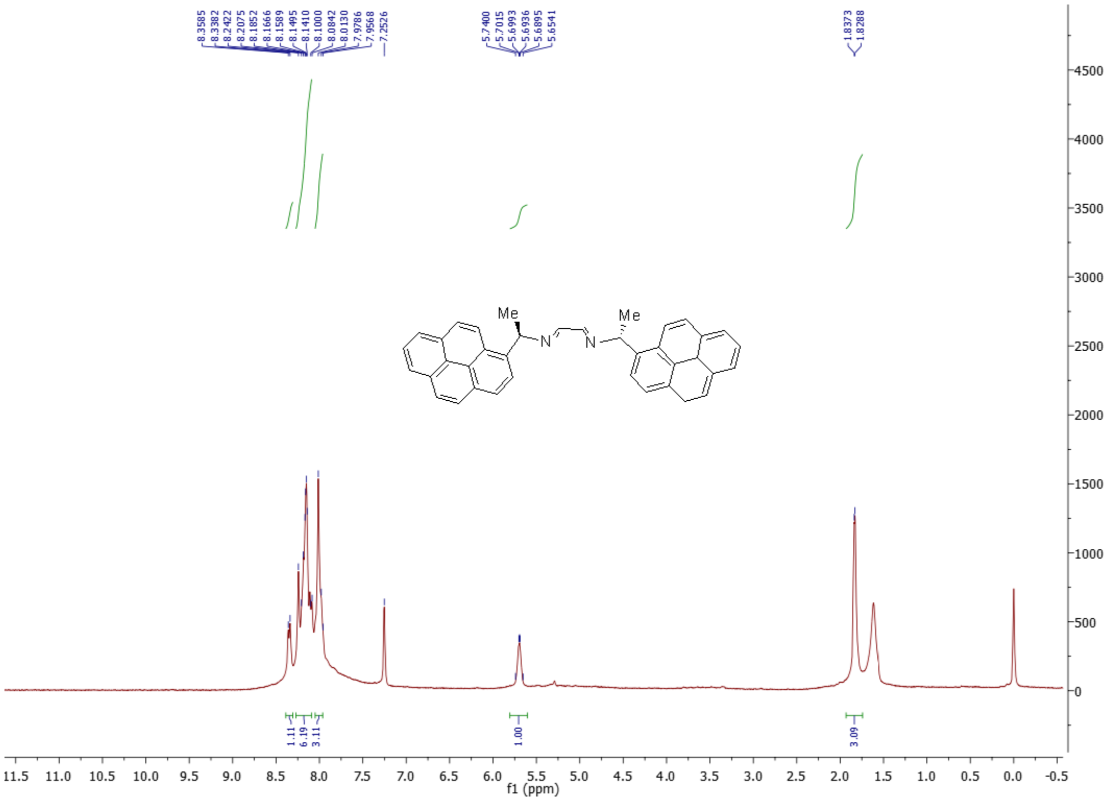

^{1}H NMR of **ligand-f** ($CDCl_3$, 400 MHz):

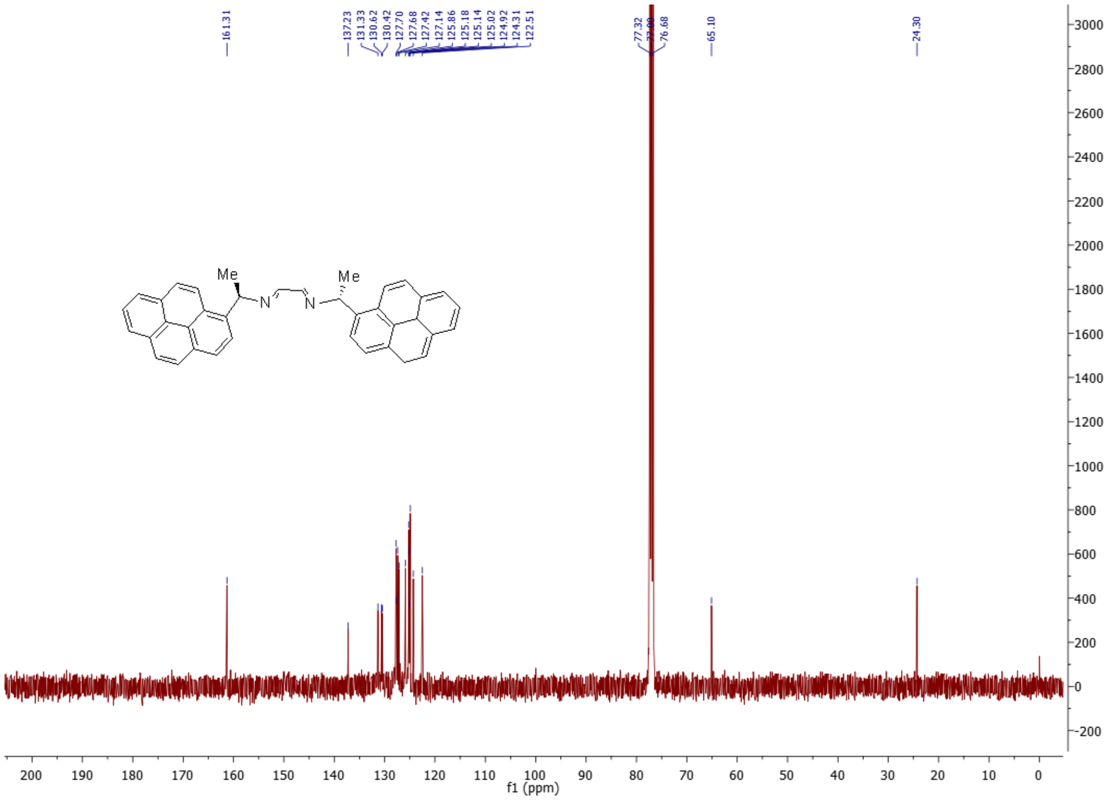

^{1}H NMR of **Mo-6 complex** ($CDCl_3$, 400 MHz):

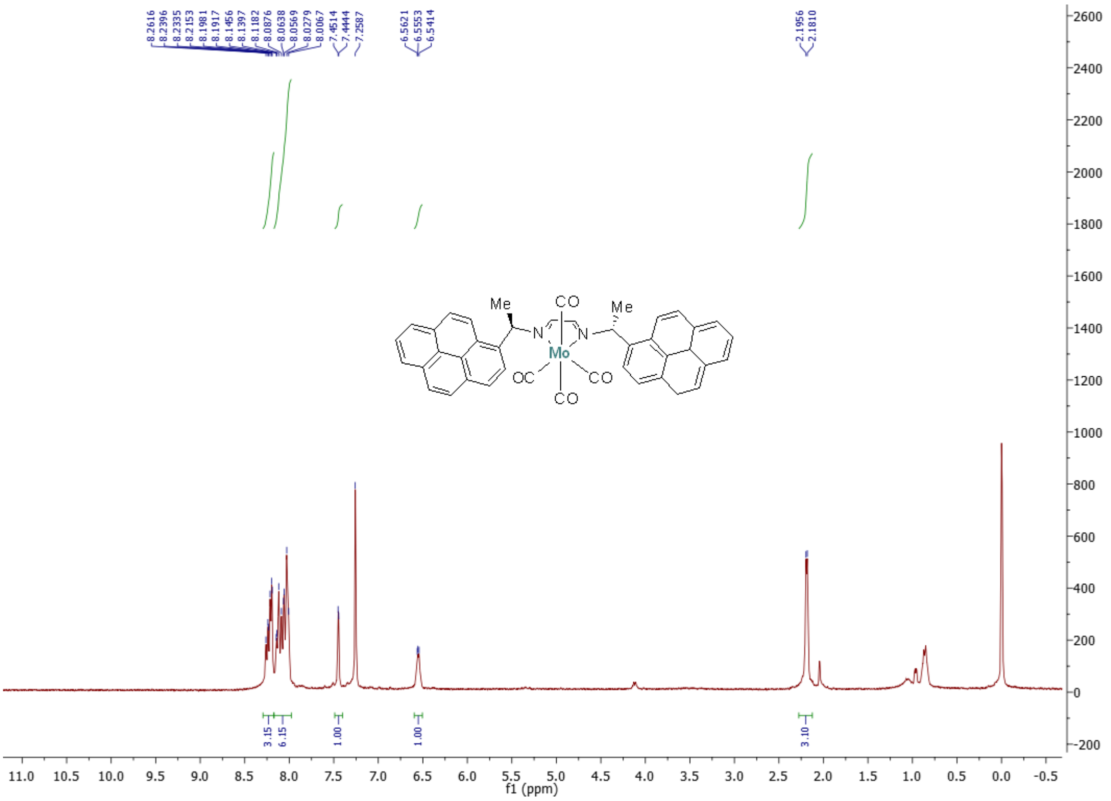

^{1}H NMR of **Mo-6 complex** ($CDCl_3$, 400 MHz):

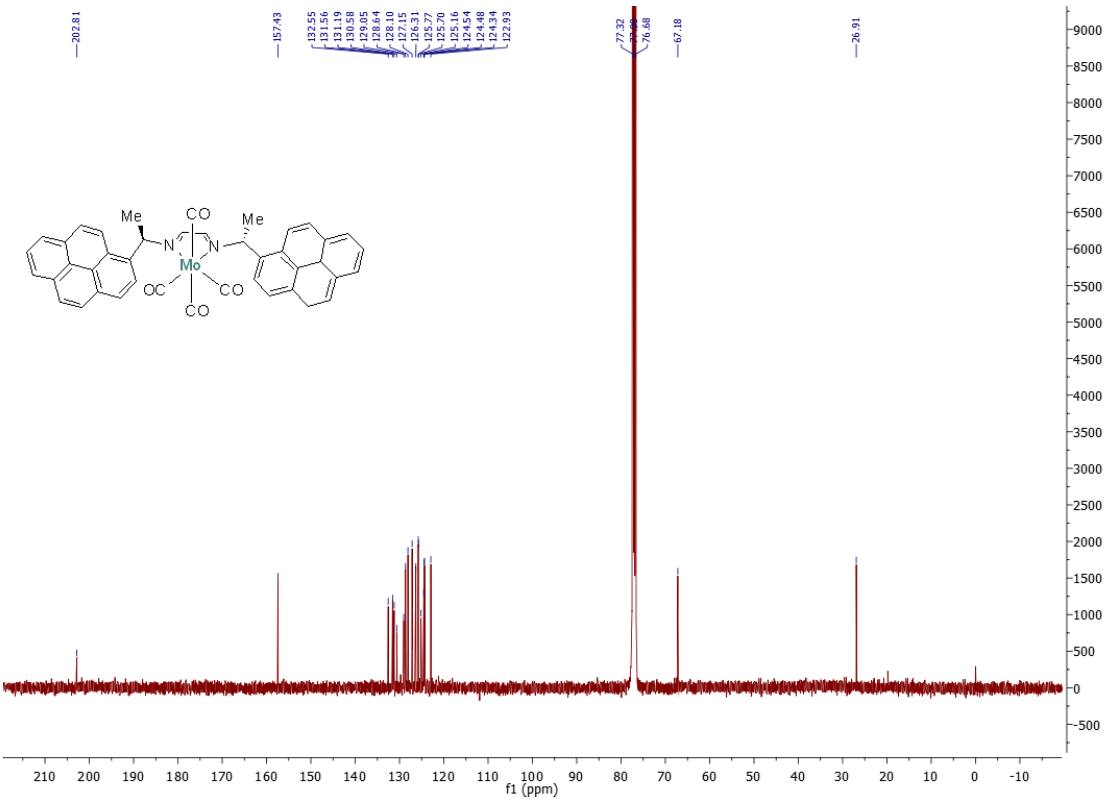

^{1}H NMR of Compound **3** ($CDCl_3$, 400 MHz):

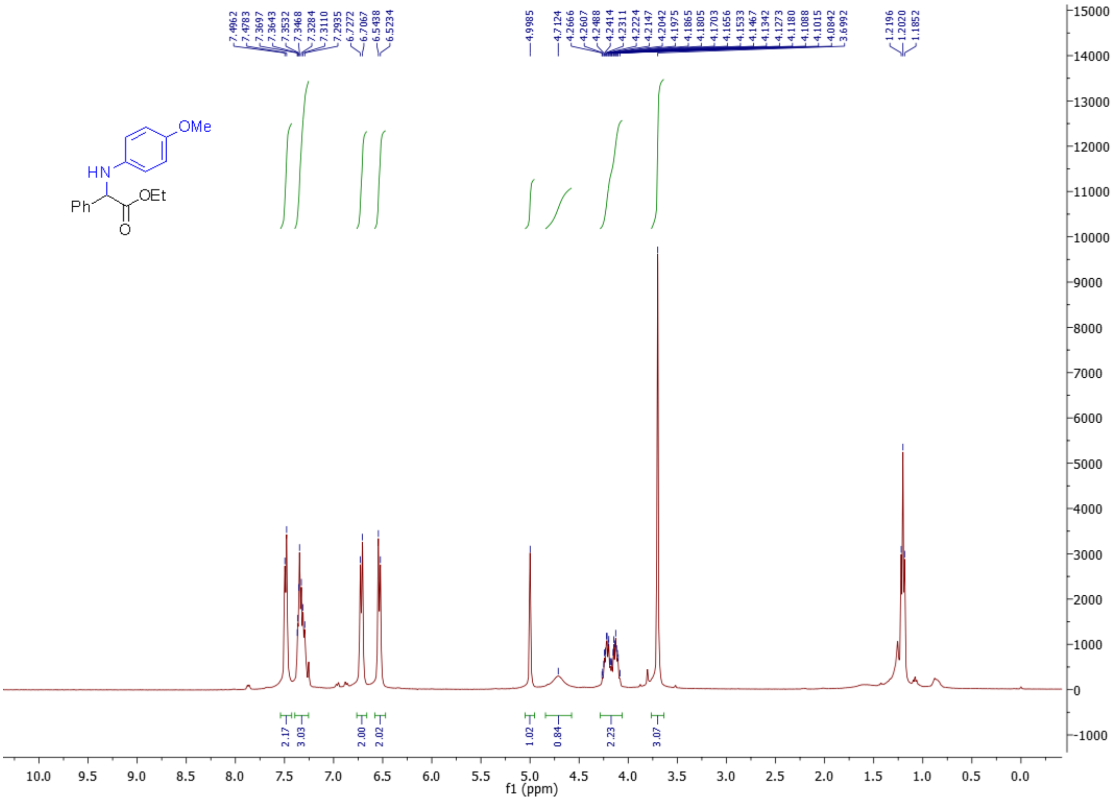

^{13}C NMR of Compound **3** ($CDCl_3$, 100 MHz):

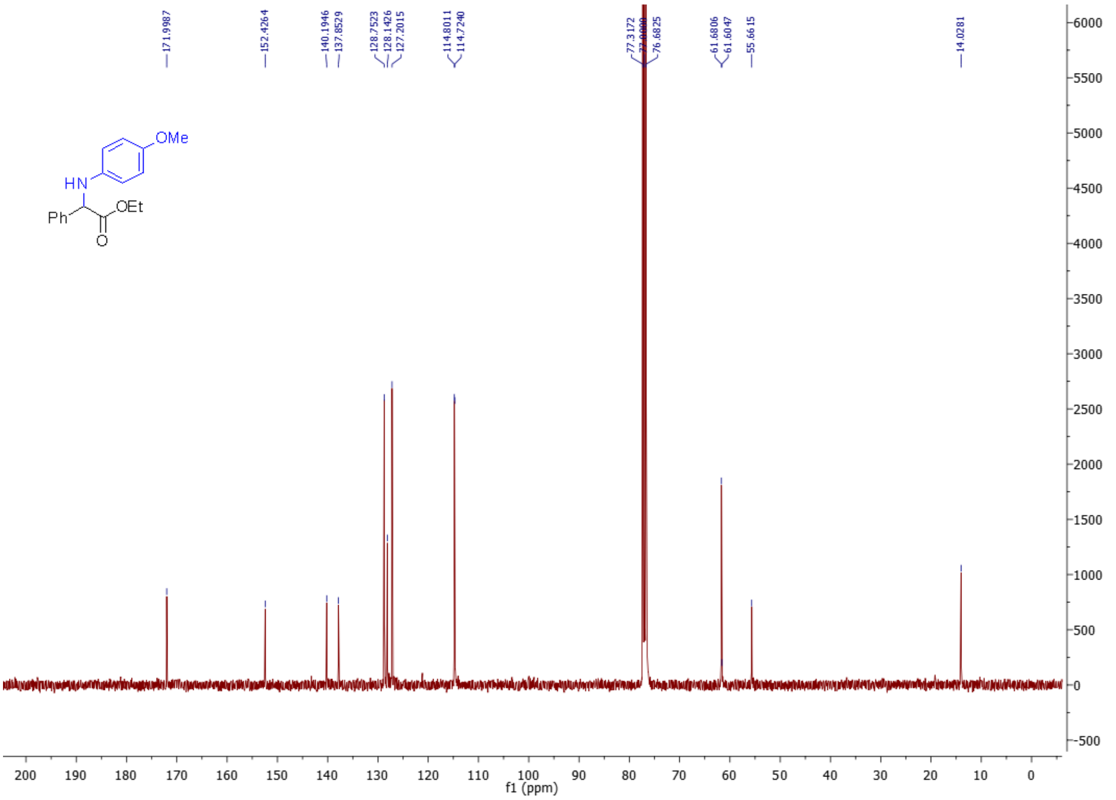

^{1}H NMR of Compound **4** ($CDCl_3$, 400 MHz):

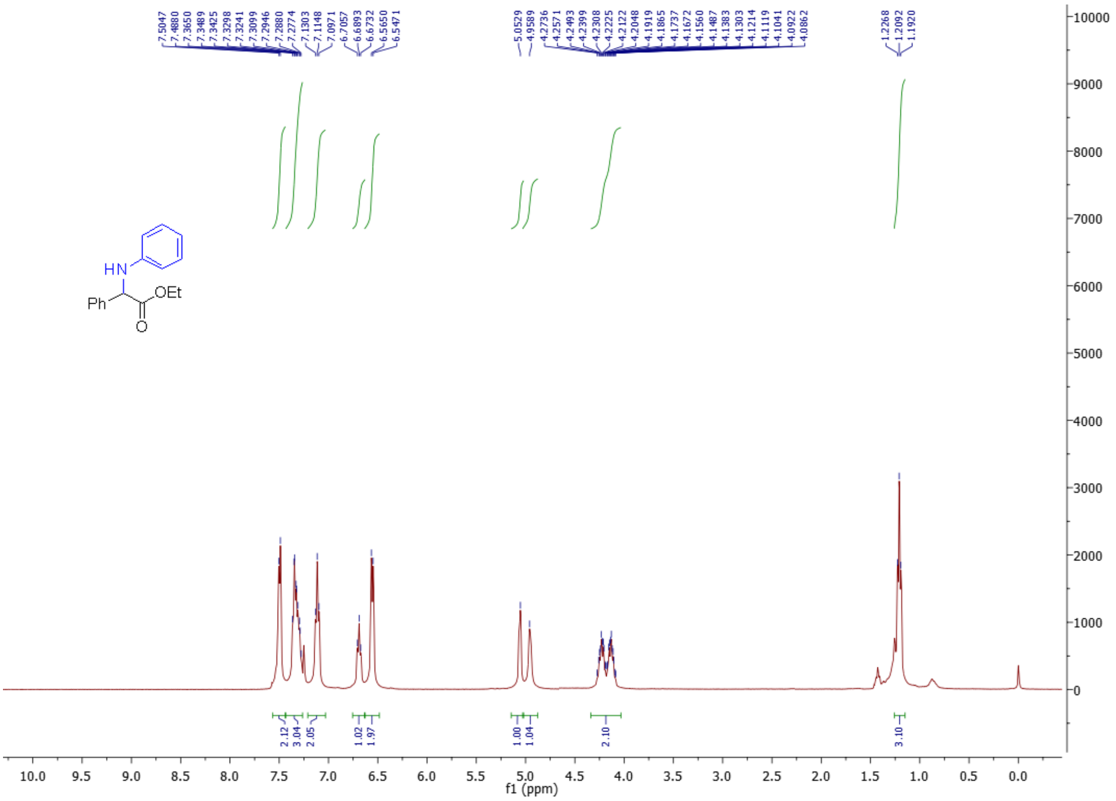

^{13}C NMR of Compound **4** ($CDCl_3$, 100 MHz):

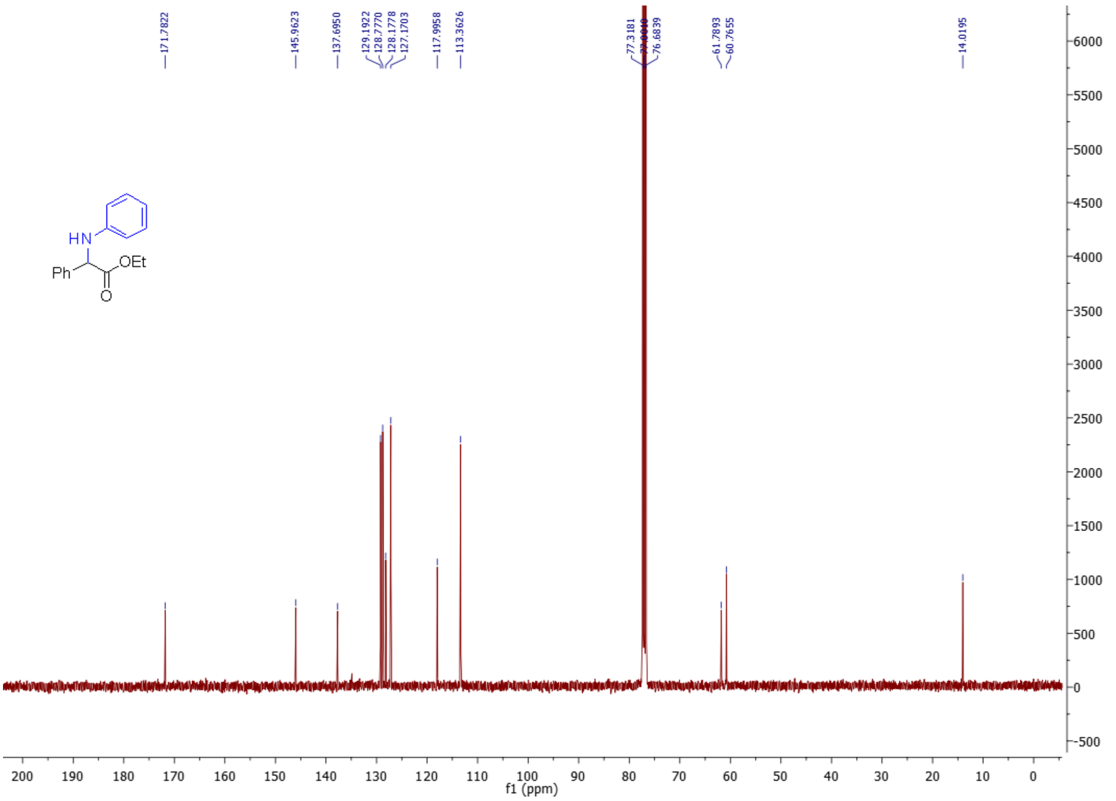

^{1}H NMR of Compound **5** ($CDCl_3$, 400 MHz):

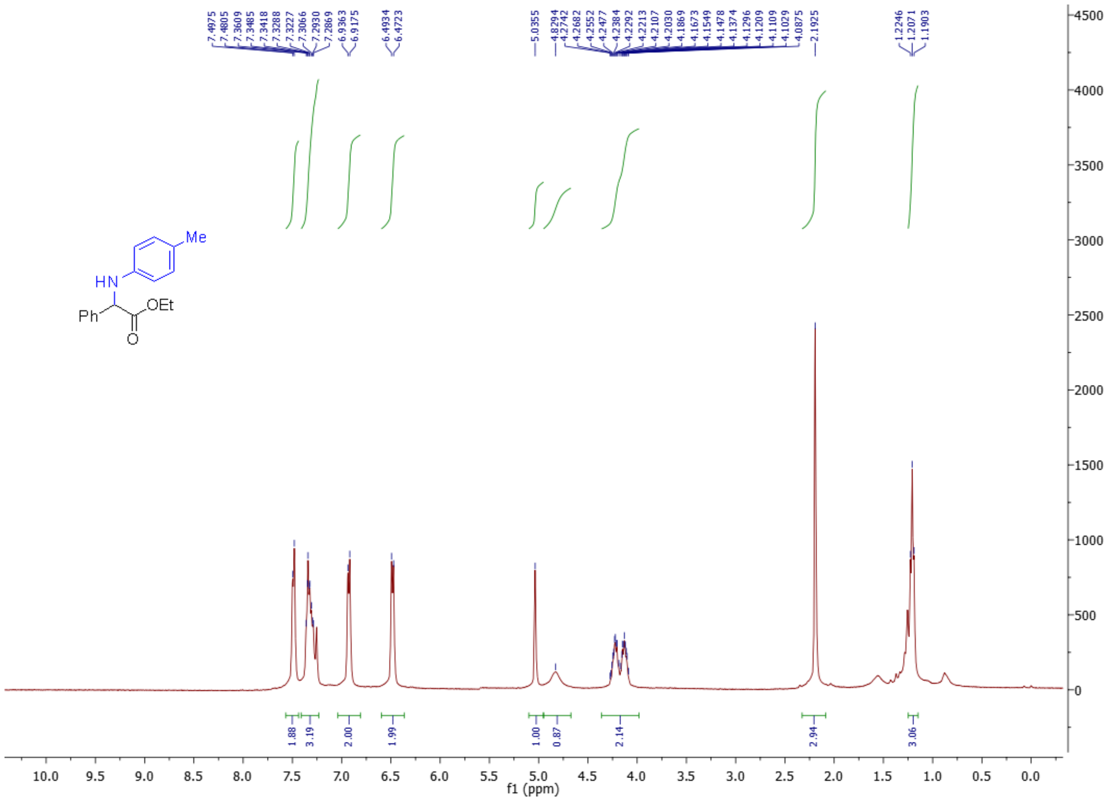

^{13}C NMR of Compound **5** ($CDCl_3$, 100 MHz):

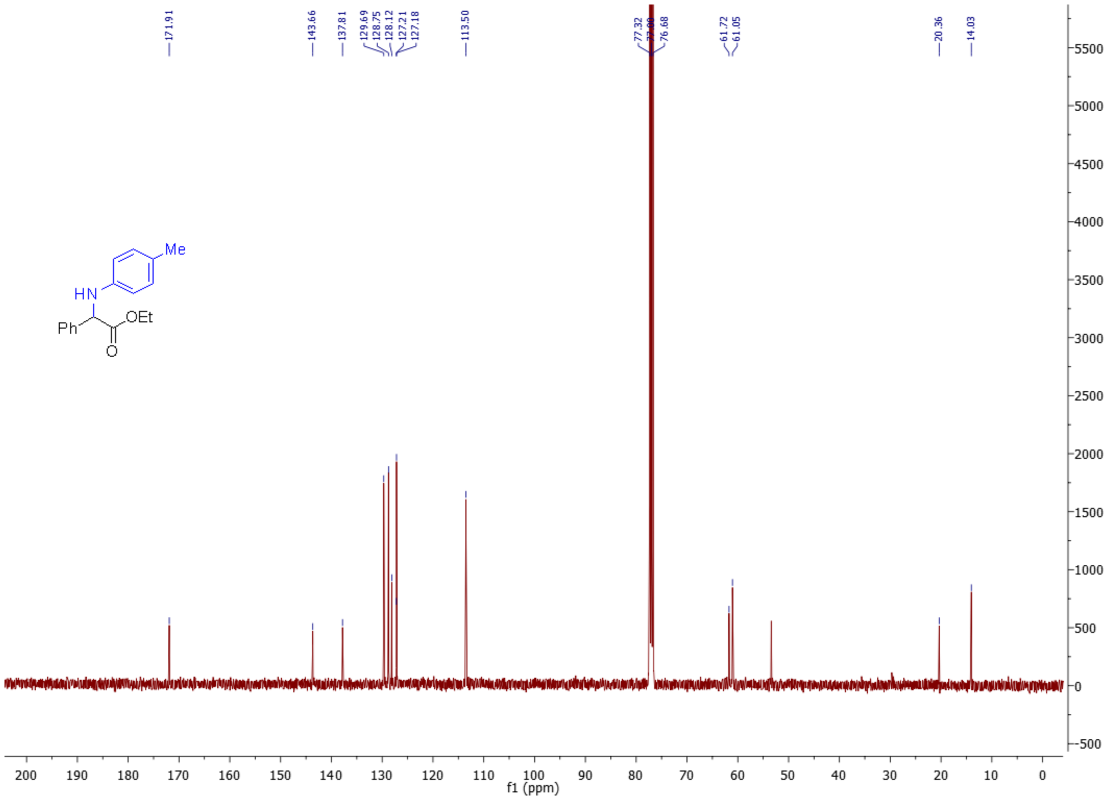

^{1}H NMR of Compound **6** ($CDCl_3$, 400 MHz):

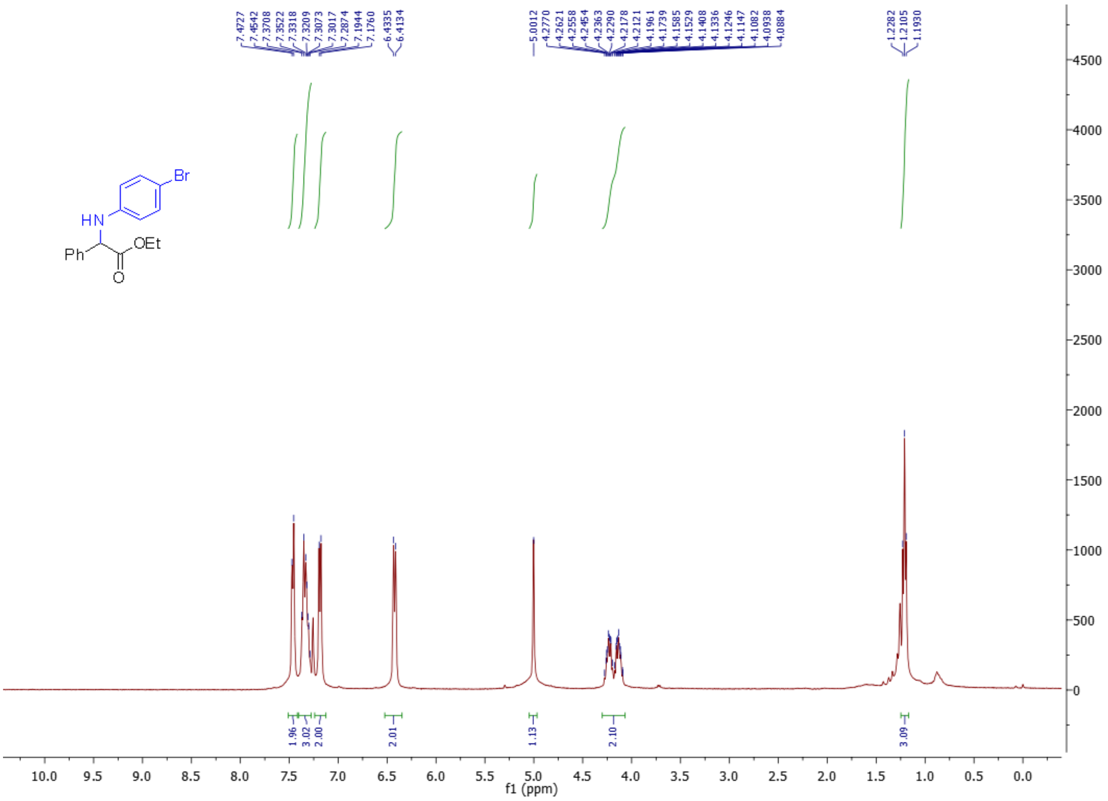

^{13}C NMR of Compound **6** ($CDCl_3$, 100 MHz):

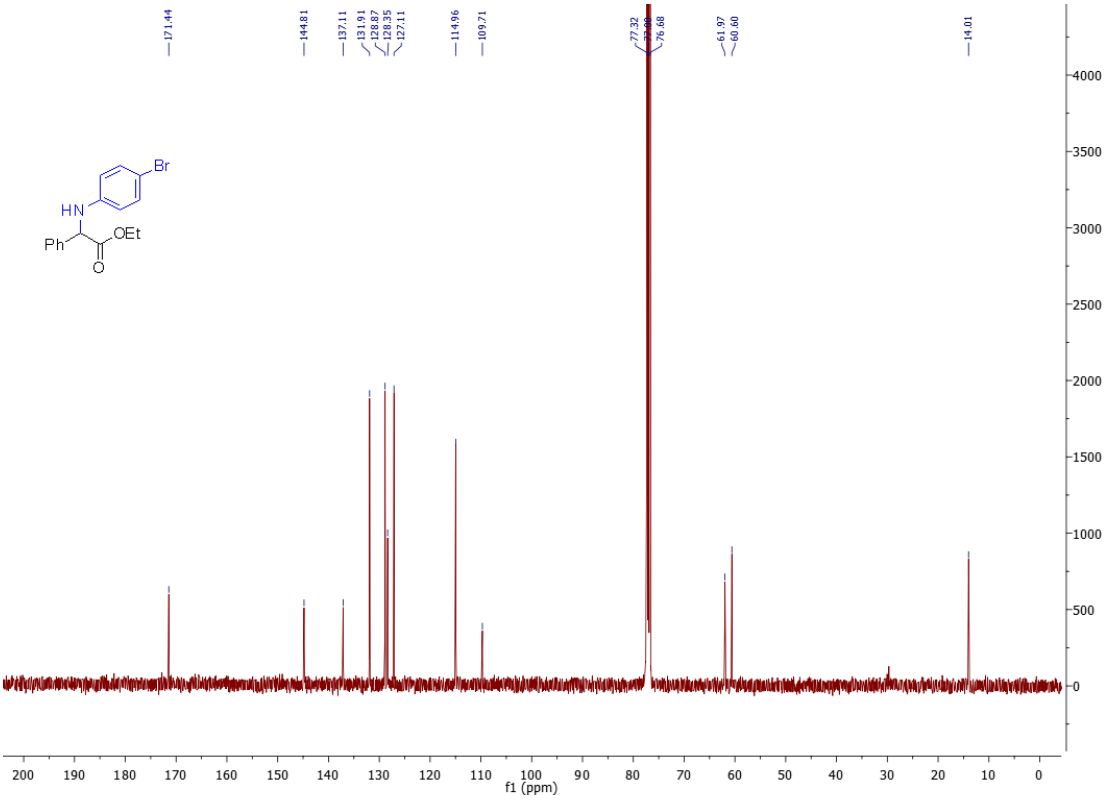

^{1}H NMR of Compound **7** ($CDCl_3$, 400 MHz):

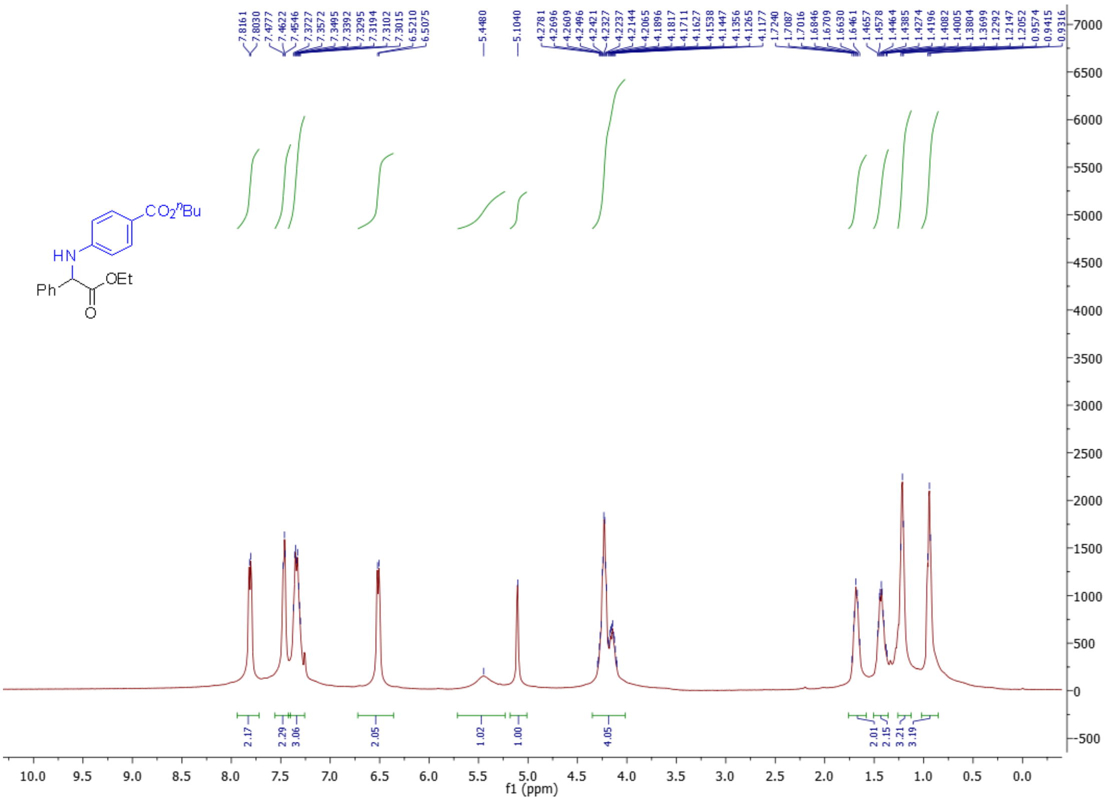

^{13}C NMR of Compound **7** ($CDCl_3$, 100 MHz):

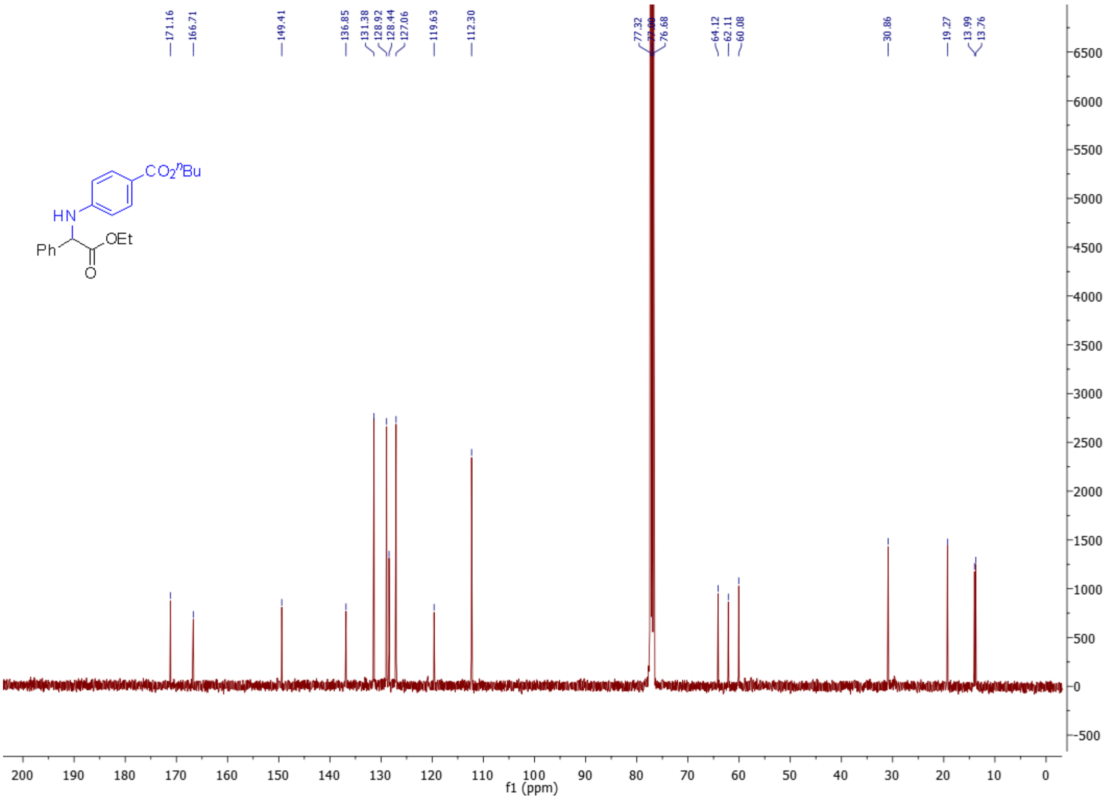

^{1}H NMR of Compound **8** ($CDCl_3$, 400 MHz):

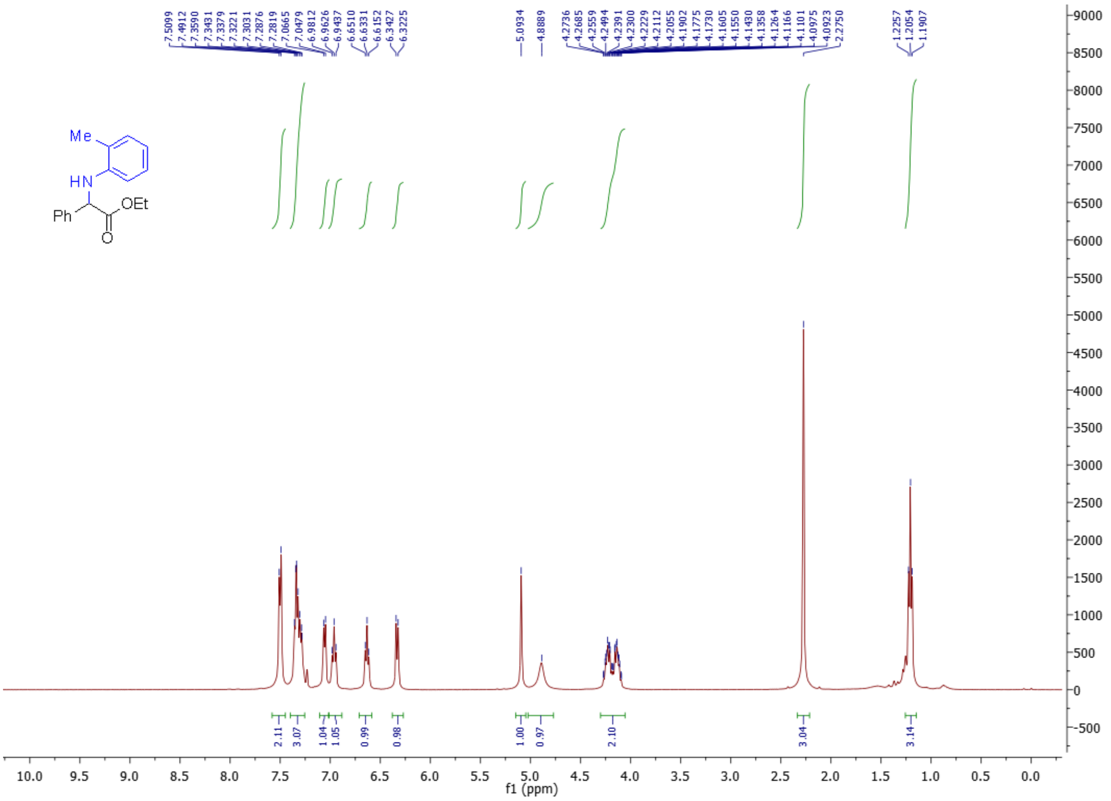

^{13}C NMR of Compound **8** ($CDCl_3$, 100 MHz):

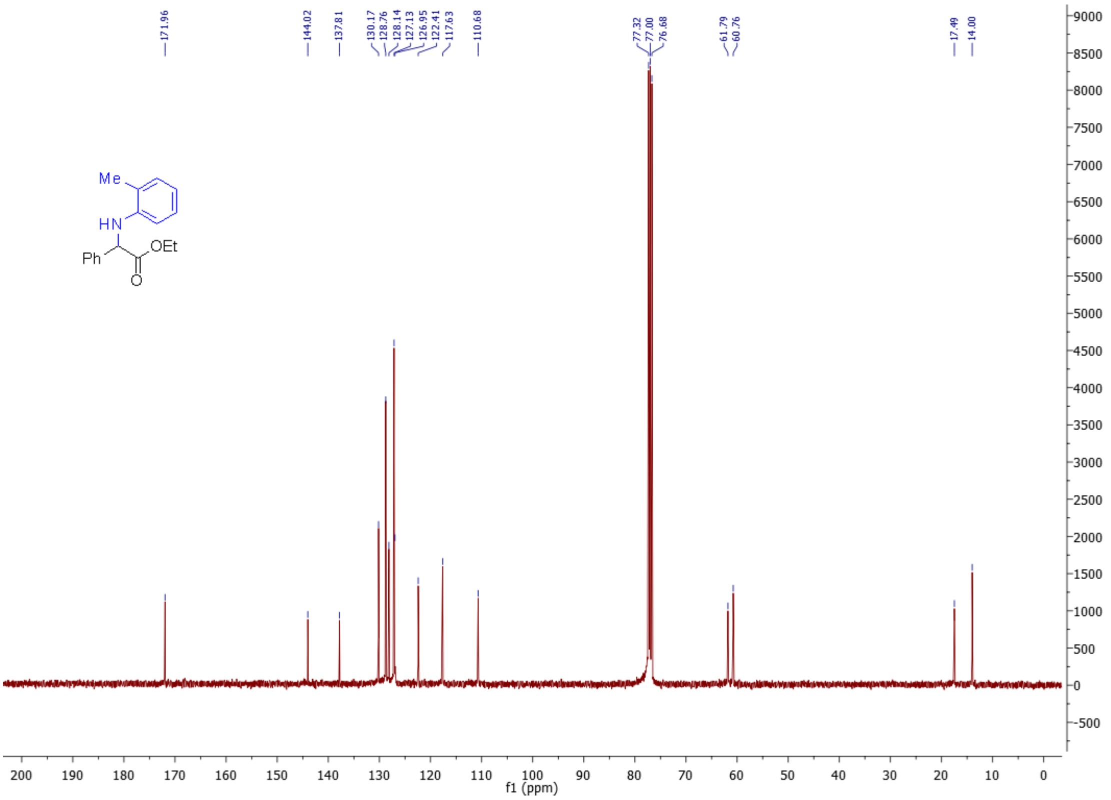

^{1}H NMR of Compound **9** ($CDCl_3$, 400 MHz):

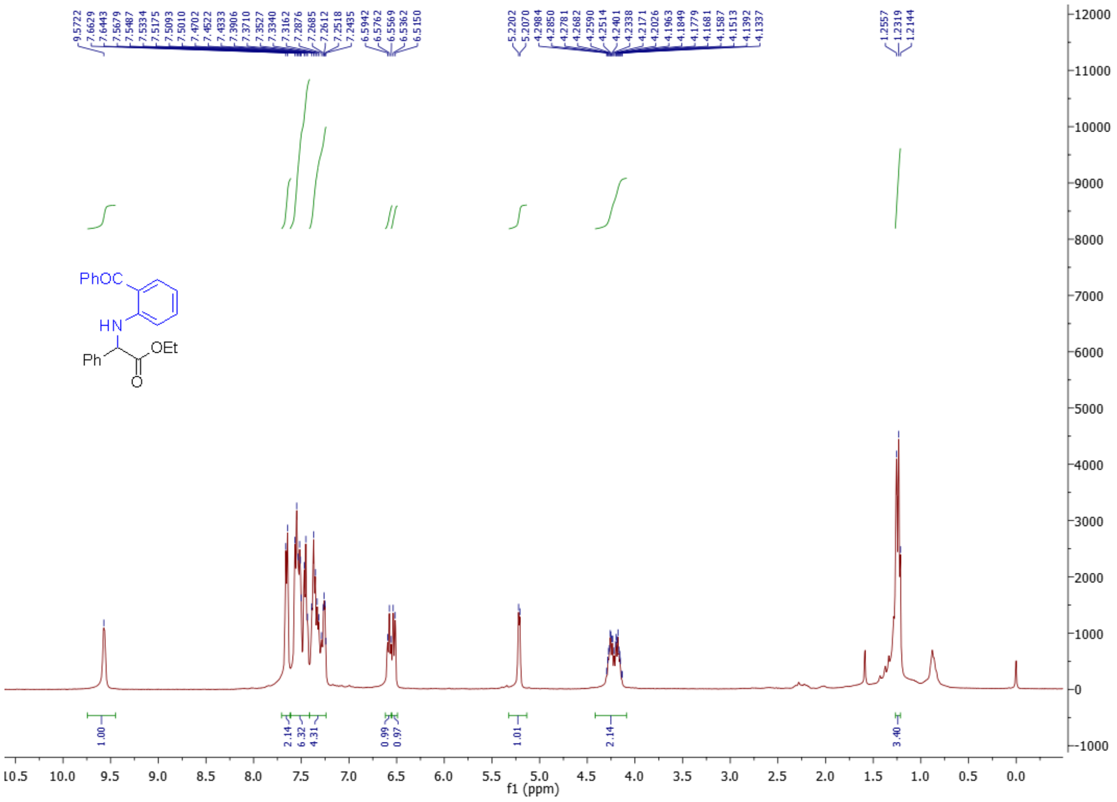

^{13}C NMR of Compound **9** ($CDCl_3$, 100 MHz):

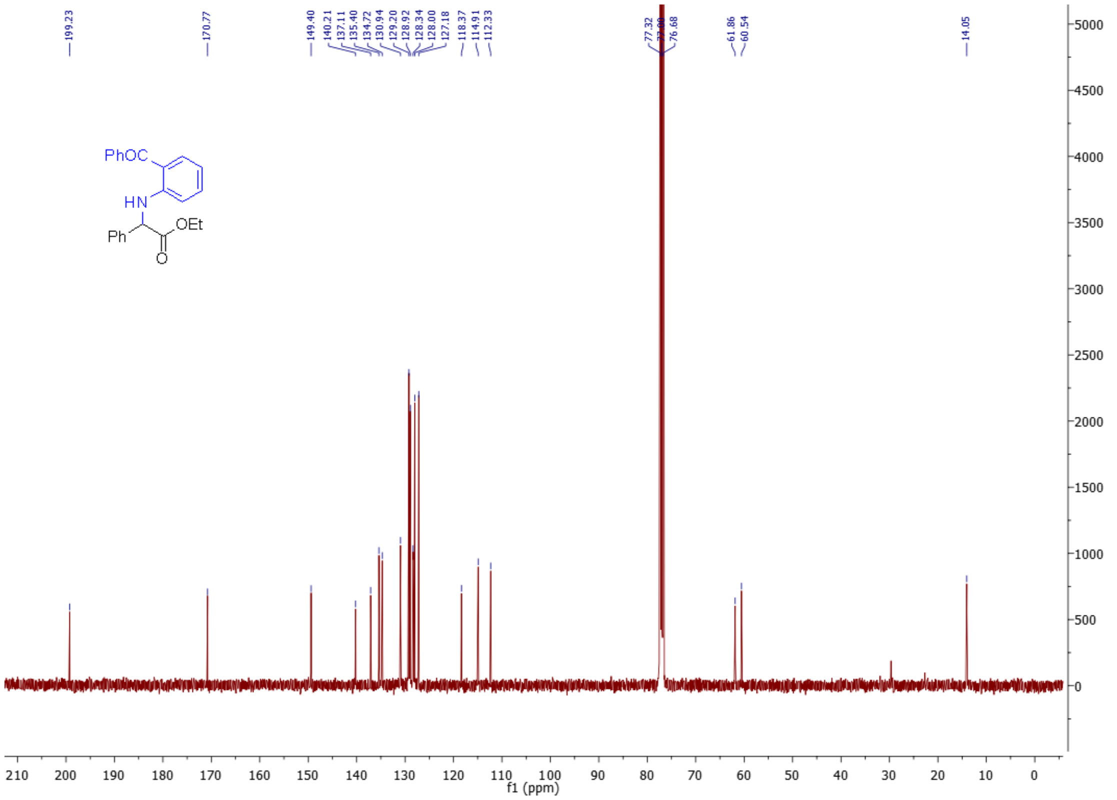

^{1}H NMR of Compound **10** ($CDCl_3$, 400 MHz):

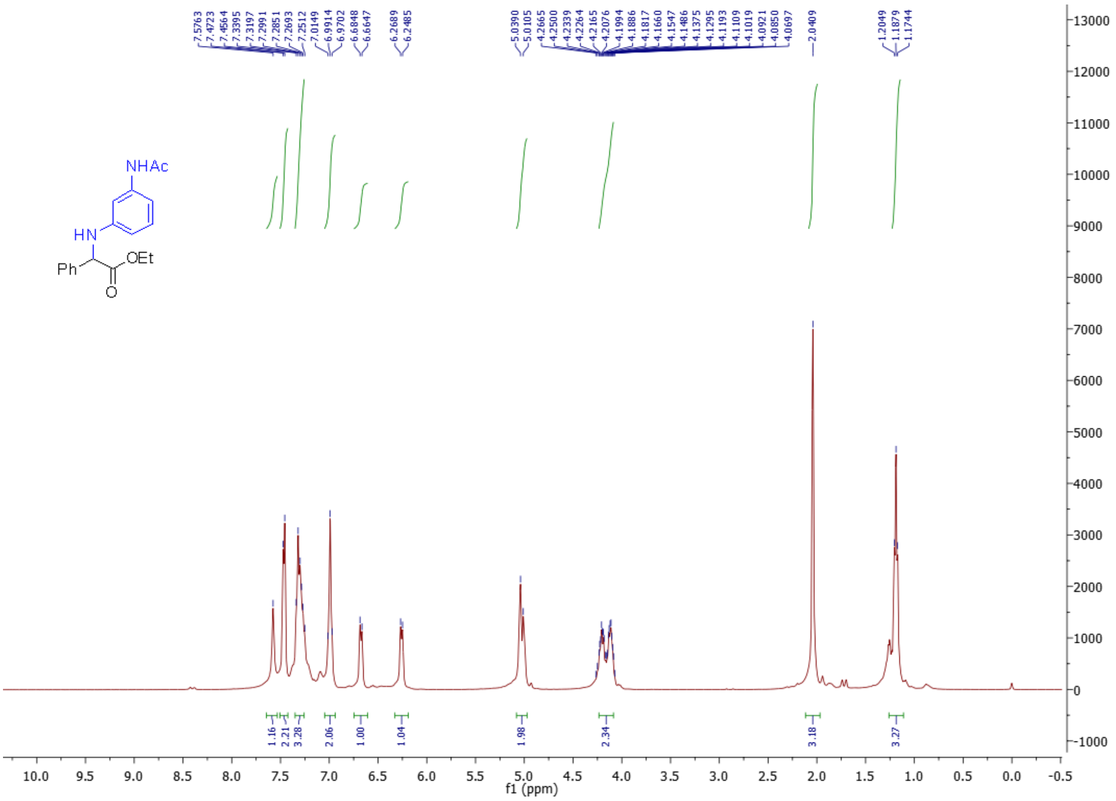

^{13}C NMR of Compound **10** ($CDCl_3$, 100 MHz):

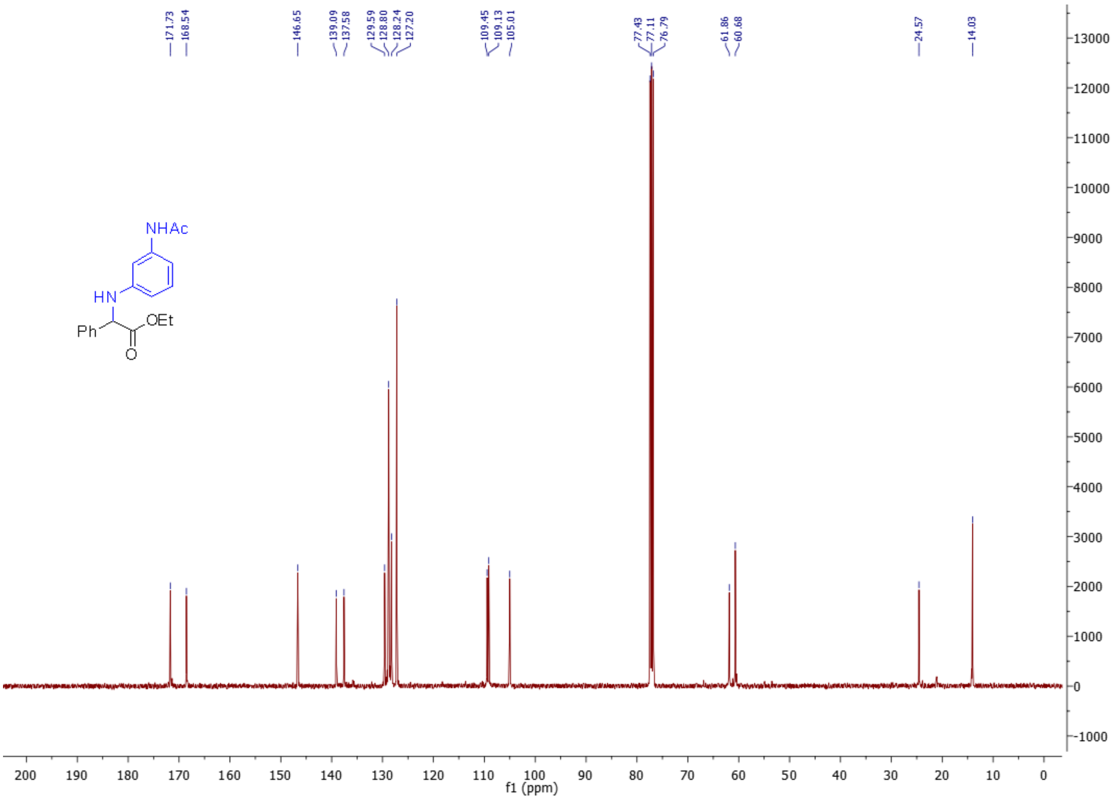

^{1}H NMR of Compound **11** ($CDCl_3$, 400 MHz):

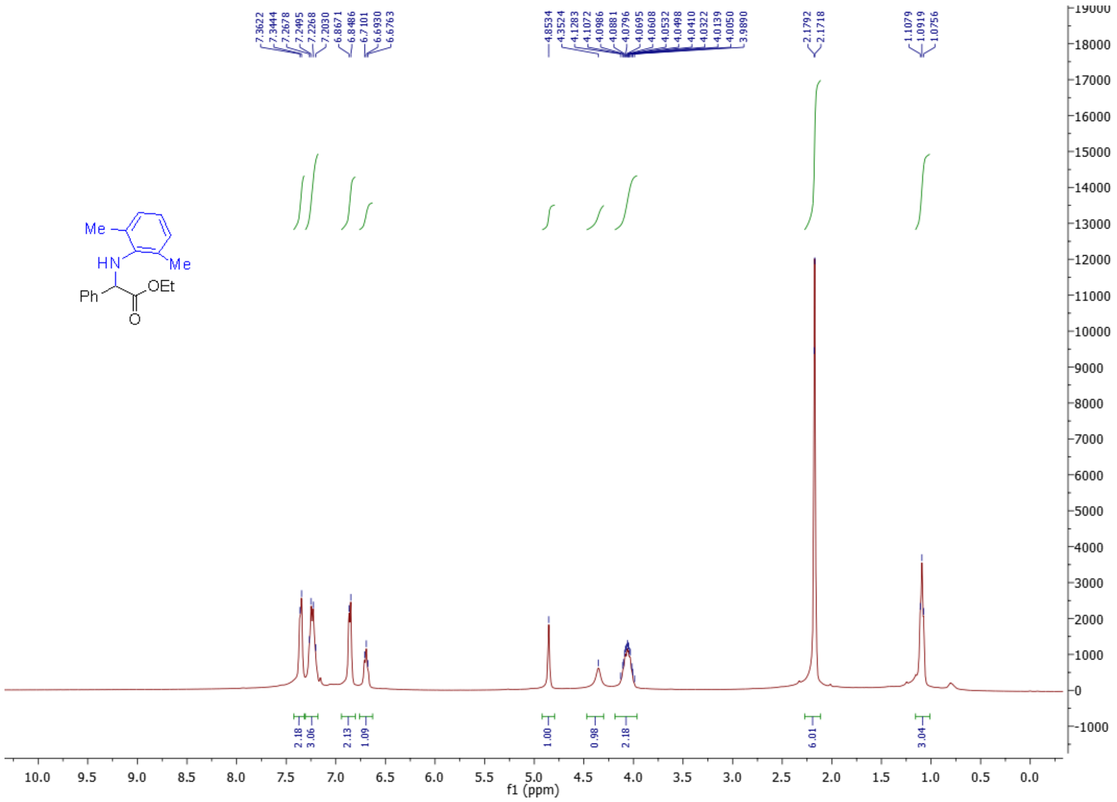

^{13}C NMR of Compound **11** ($CDCl_3$, 100 MHz):

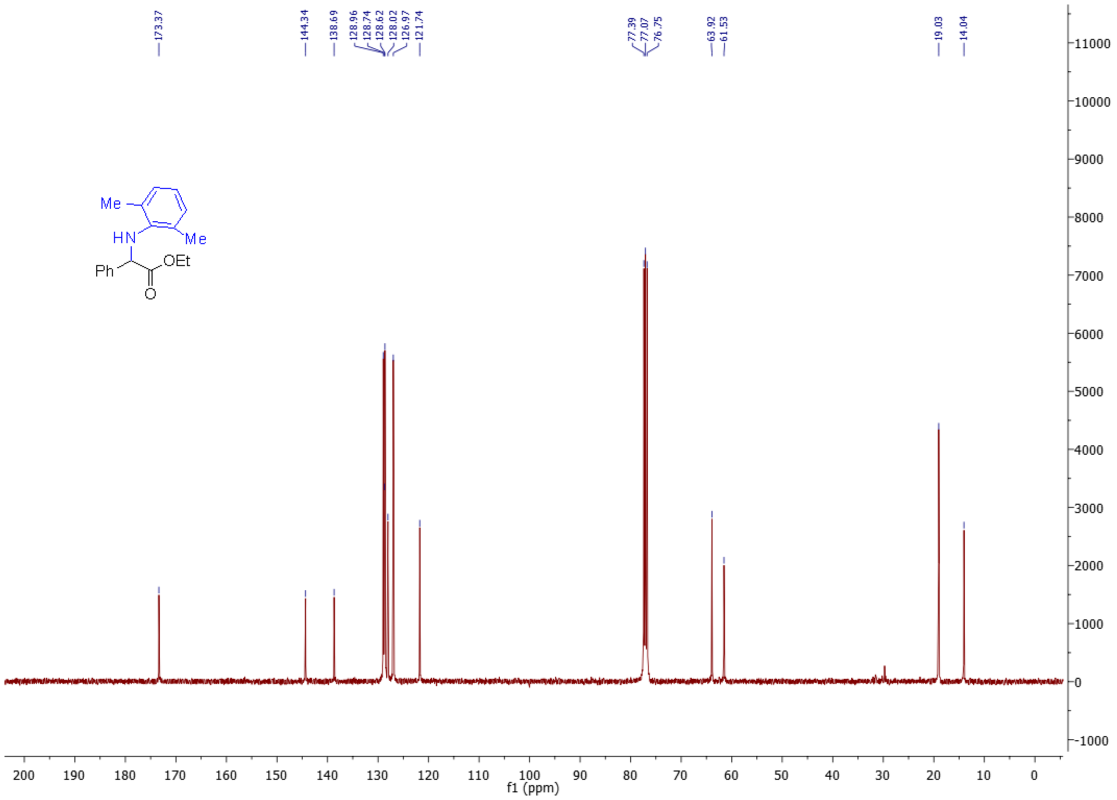

^{1}H NMR of Compound **12** ($CDCl_3$, 400 MHz):

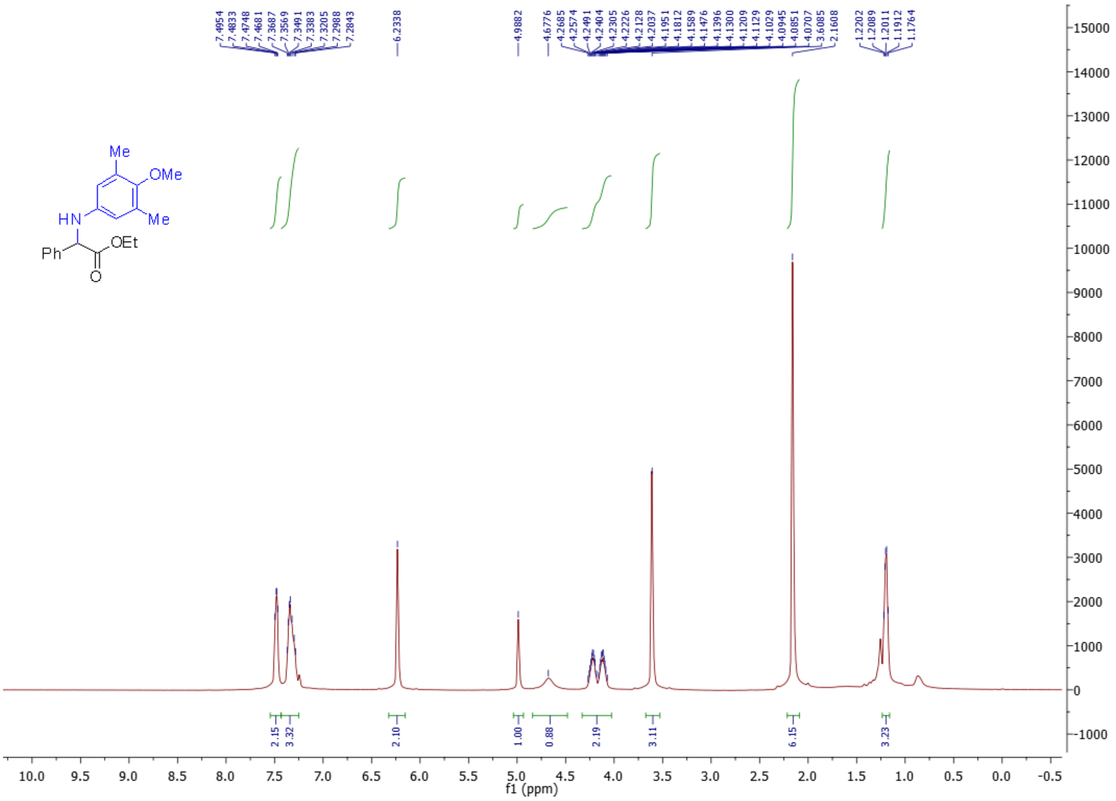

^{13}C NMR of Compound **12** ($CDCl_3$, 100 MHz):

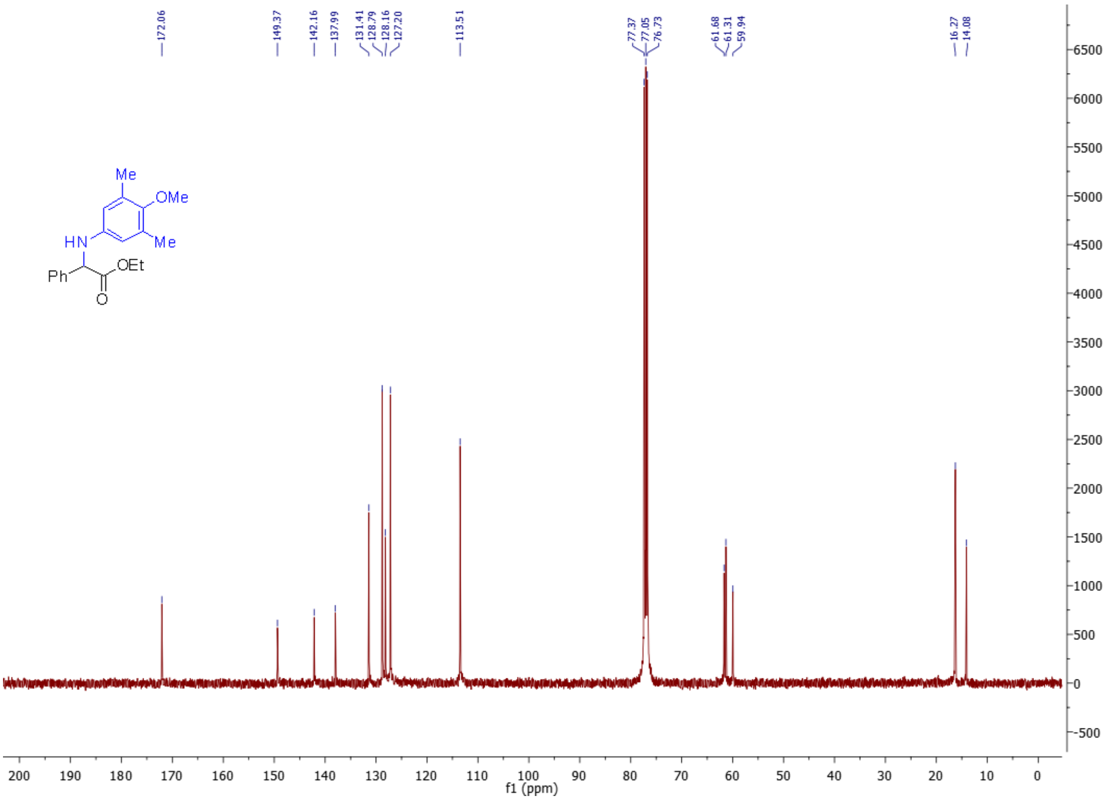

^{1}H NMR of Compound **13** ($CDCl_3$, 400 MHz):

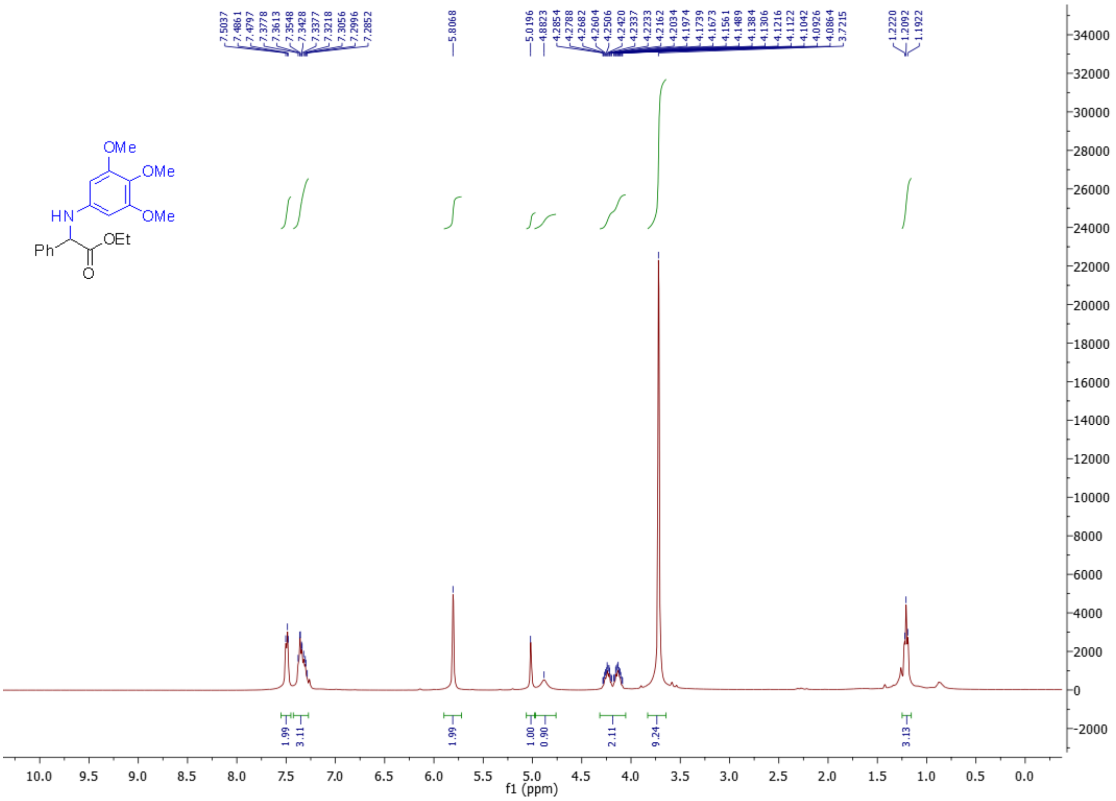

^{13}C NMR of Compound **13** ($CDCl_3$, 100 MHz):

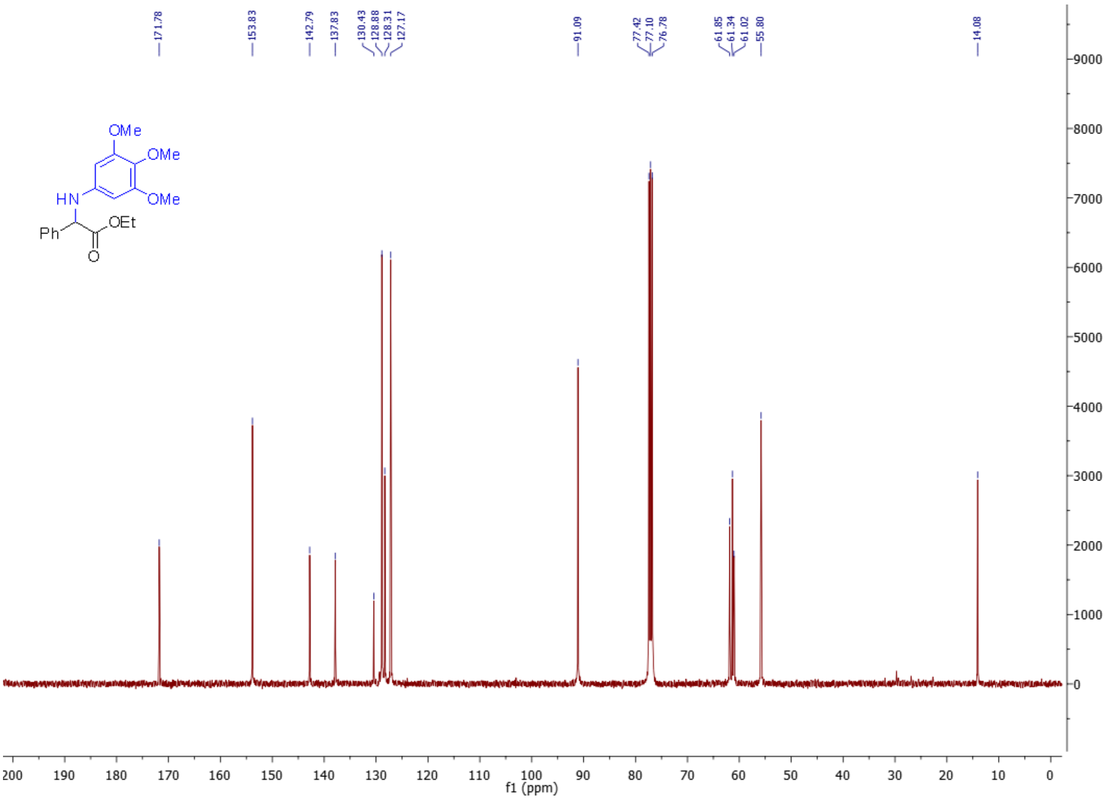

^{1}H NMR of Compound **14** ($CDCl_3$, 400 MHz):

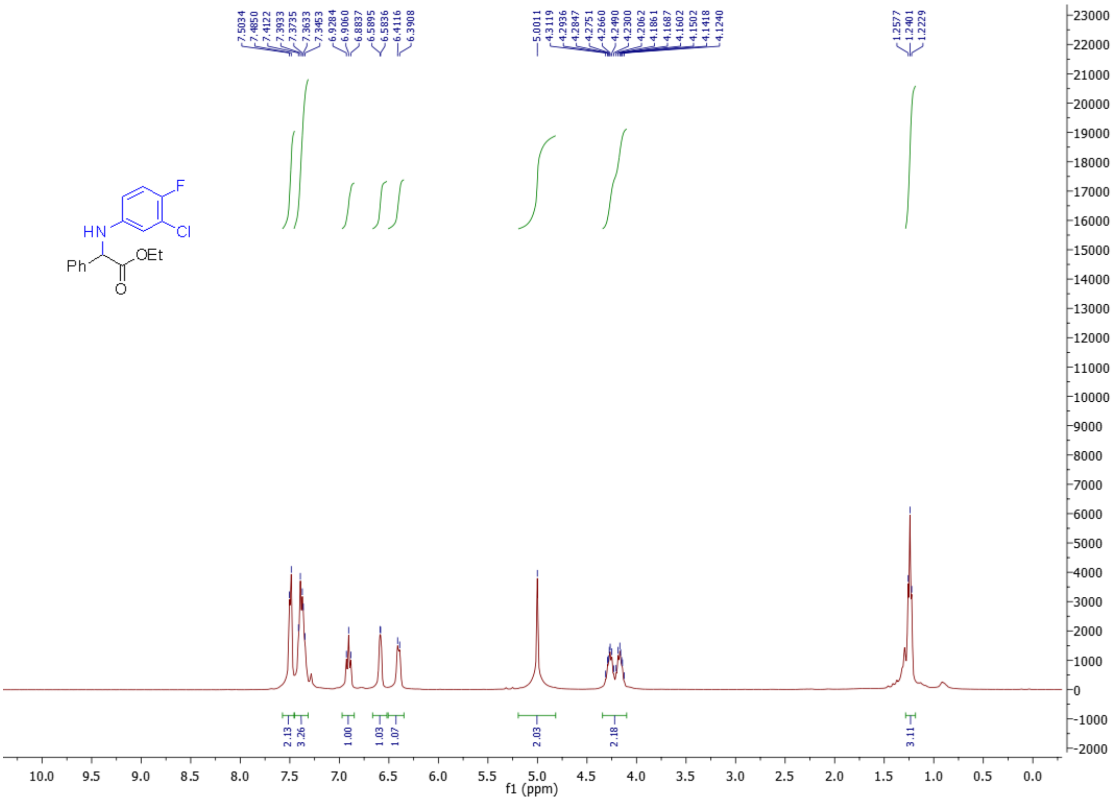

^{13}C NMR of Compound **14** ($CDCl_3$, 100 MHz):

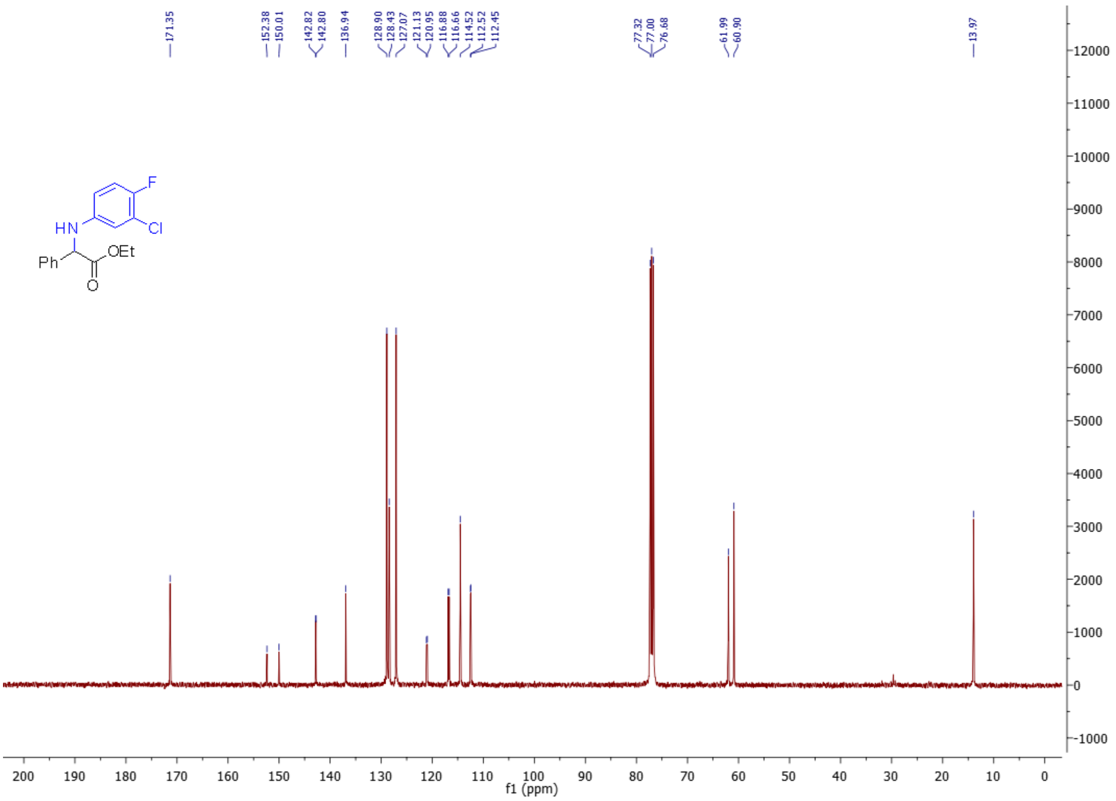

^{1}H NMR of Compound **15** ($CDCl_3$, 400 MHz):

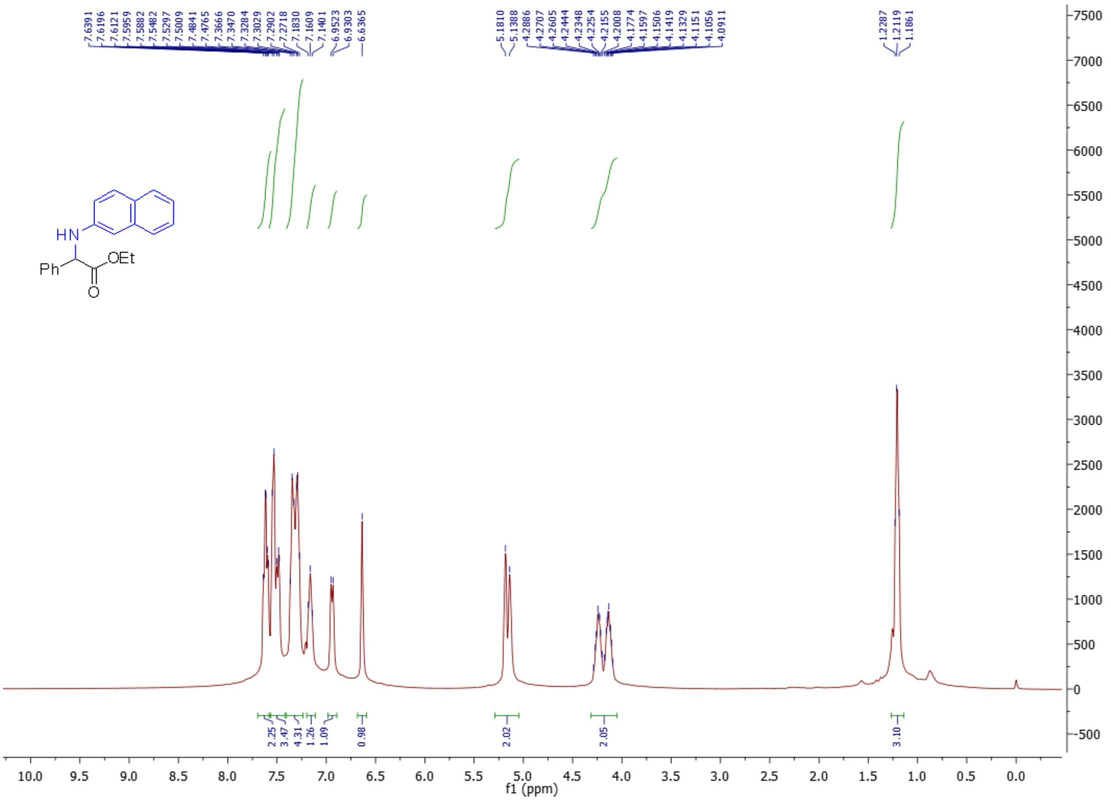

^{13}C NMR of Compound **15** ($CDCl_3$, 100 MHz):

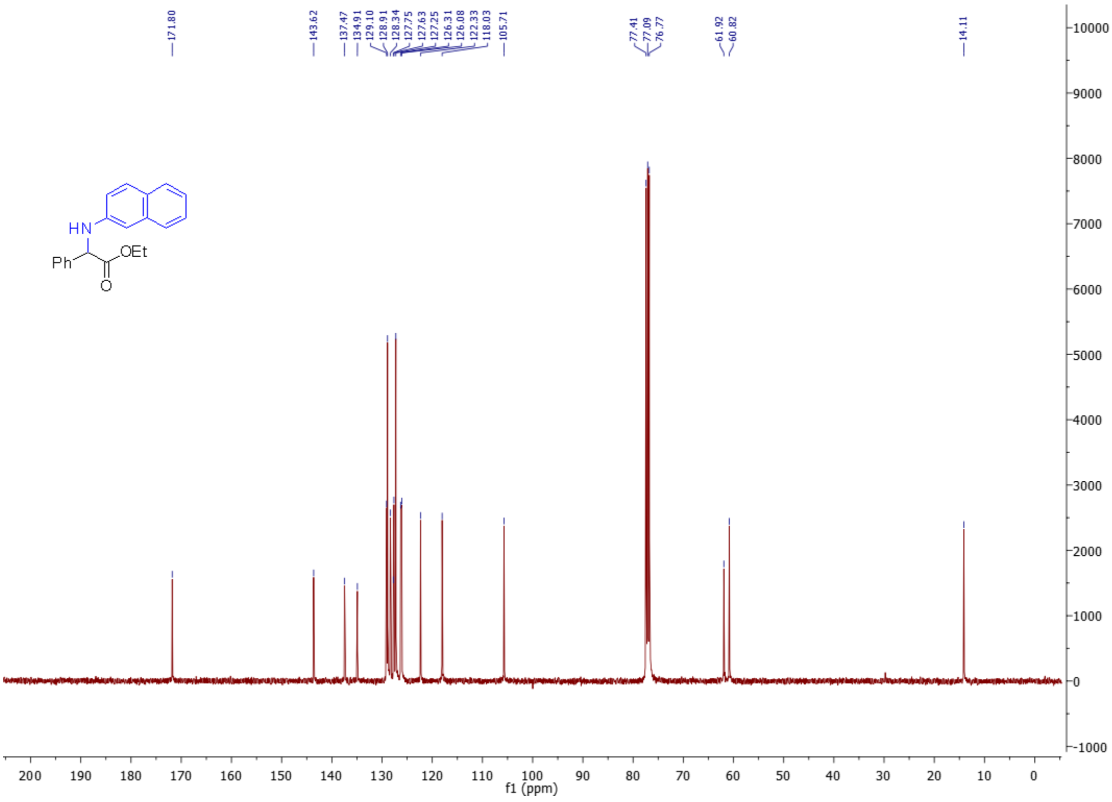

^{1}H NMR of Compound **16** ($CDCl_3$, 400 MHz):

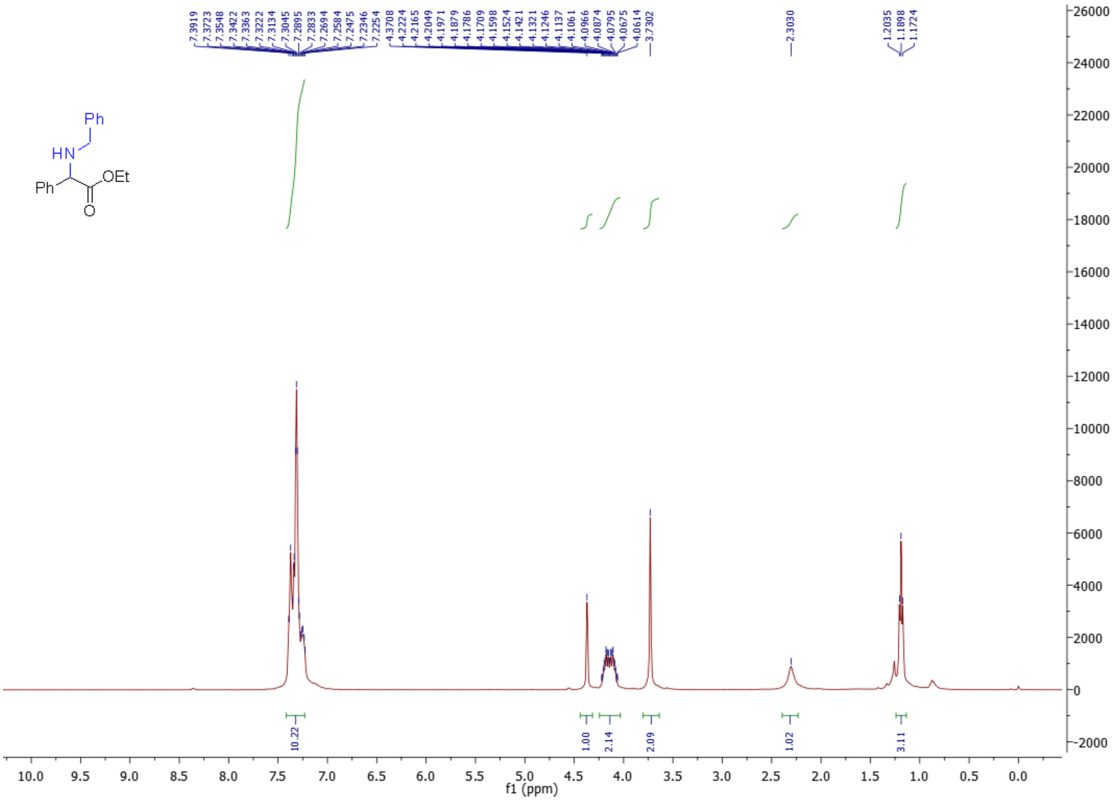

^{13}C NMR of Compound **16** ($CDCl_3$, 100 MHz):

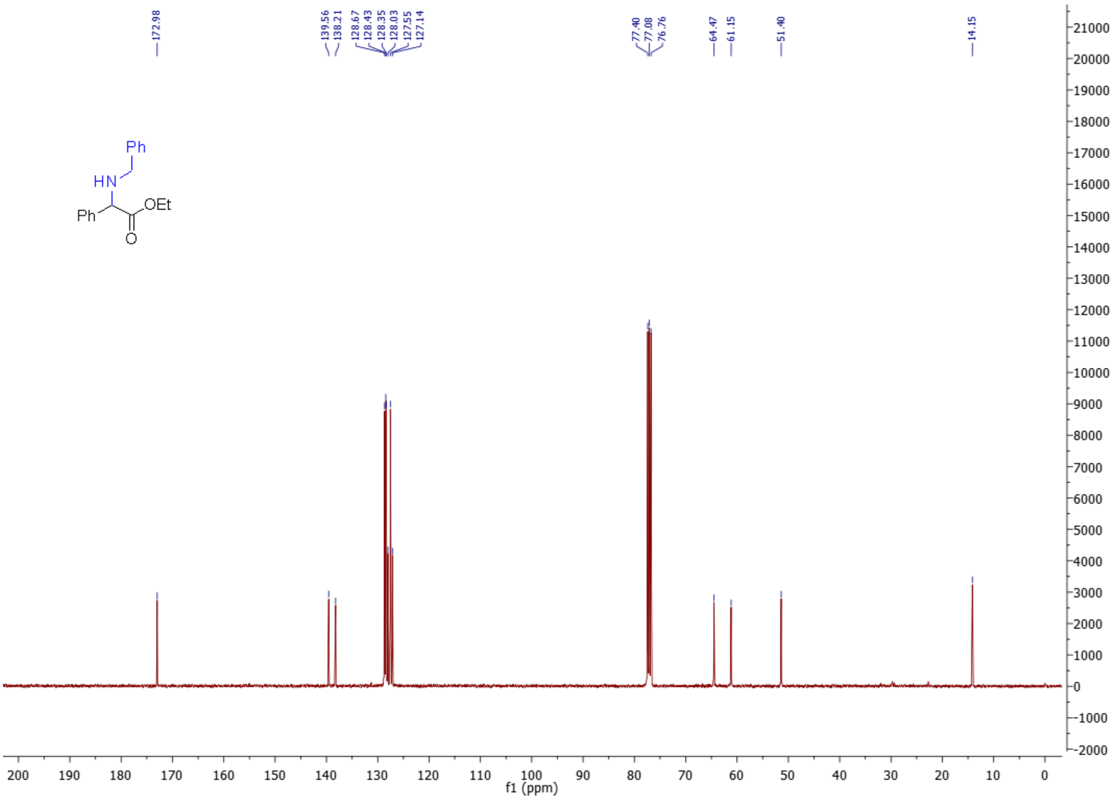

^{1}H NMR of Compound **17** ($CDCl_3$, 400 MHz):

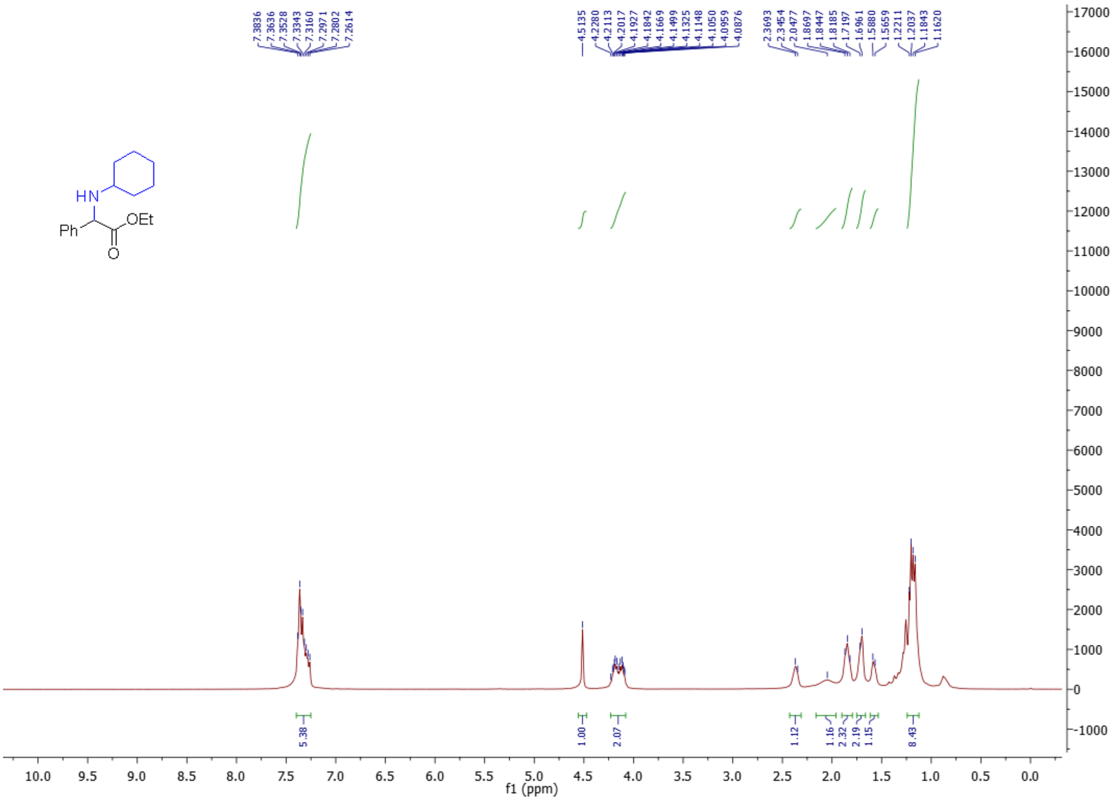

^{13}C NMR of Compound **17** ($CDCl_3$, 100 MHz):

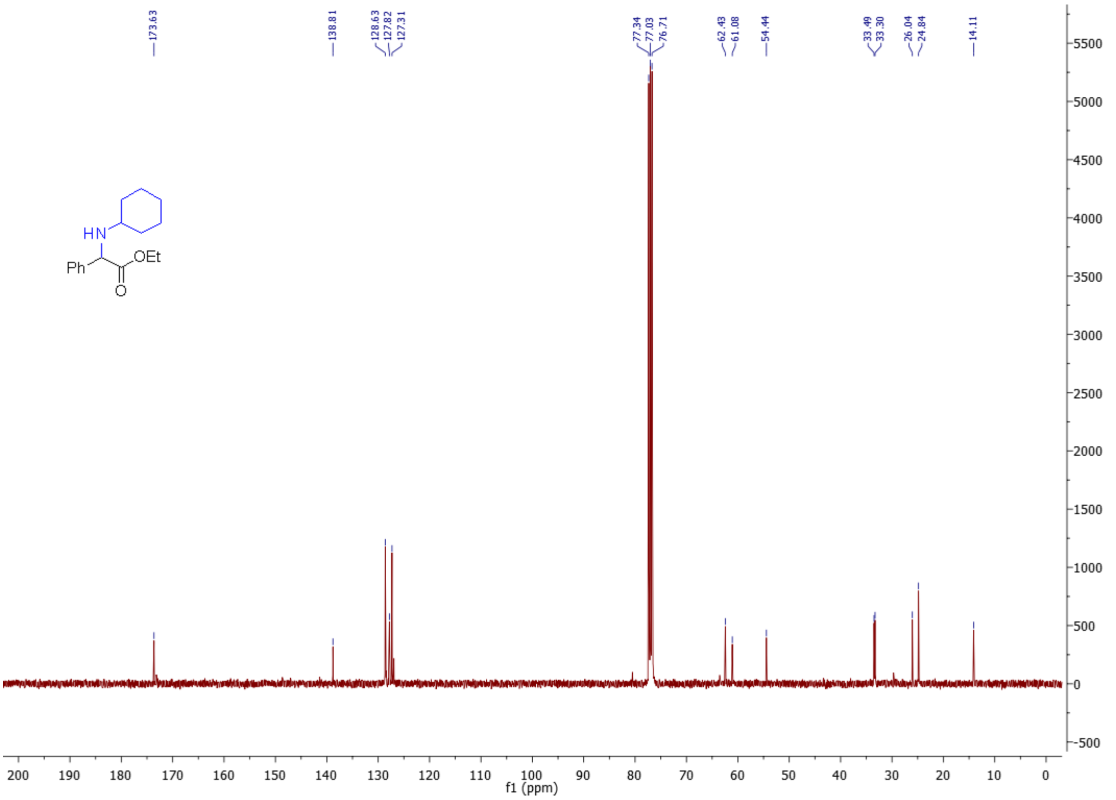

^{1}H NMR of Compound **18** ($CDCl_3$, 400 MHz):

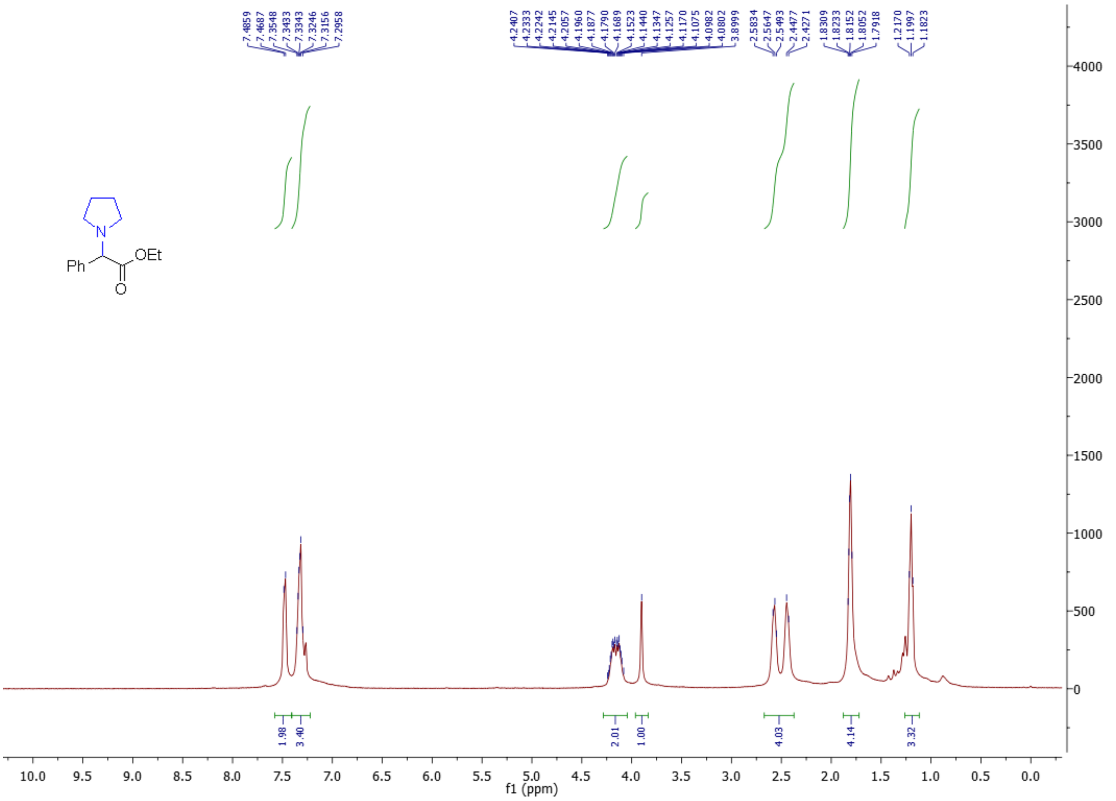

^{13}C NMR of Compound **18** ($CDCl_3$, 100 MHz):

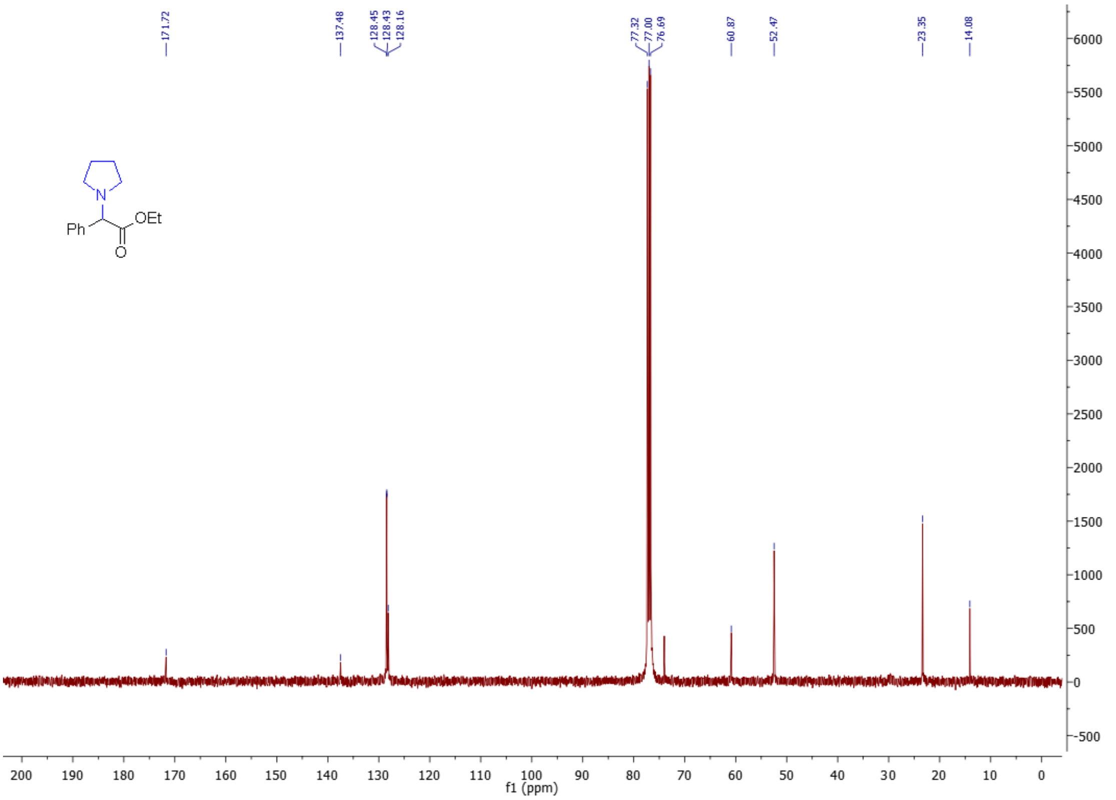

^{1}H NMR of Compound **19** ($CDCl_3$, 400 MHz):

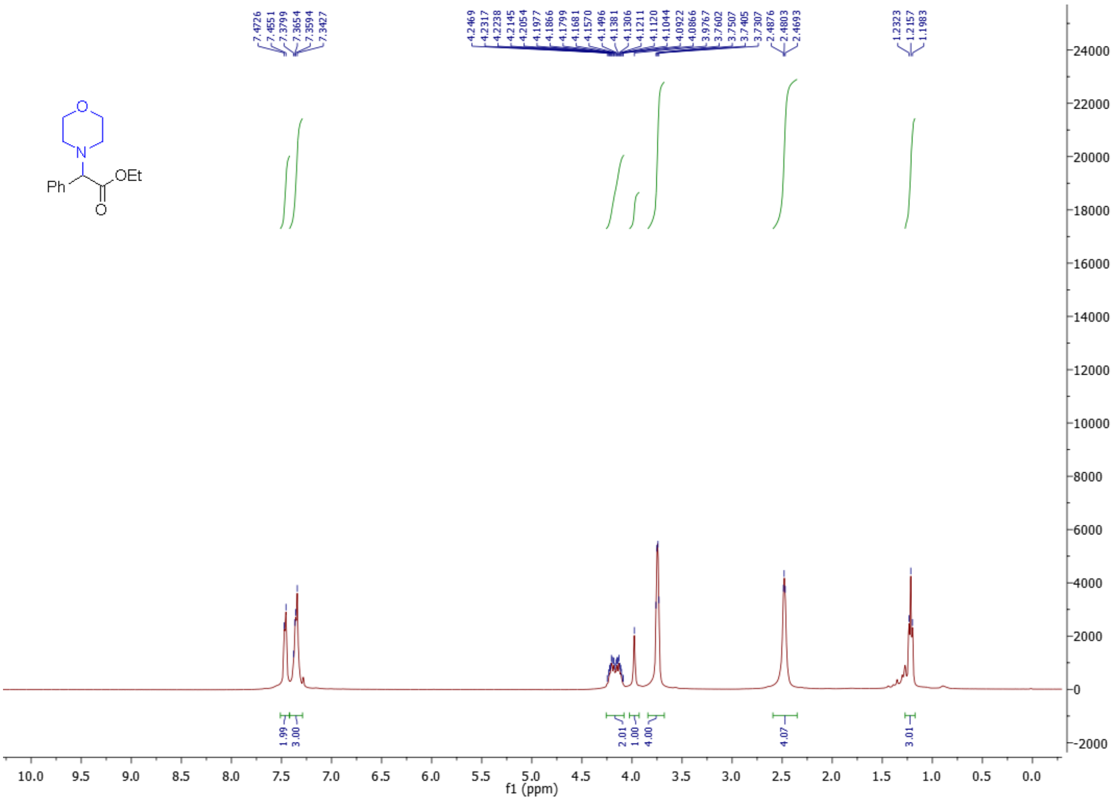

^{13}C NMR of Compound **19** ($CDCl_3$, 100 MHz):

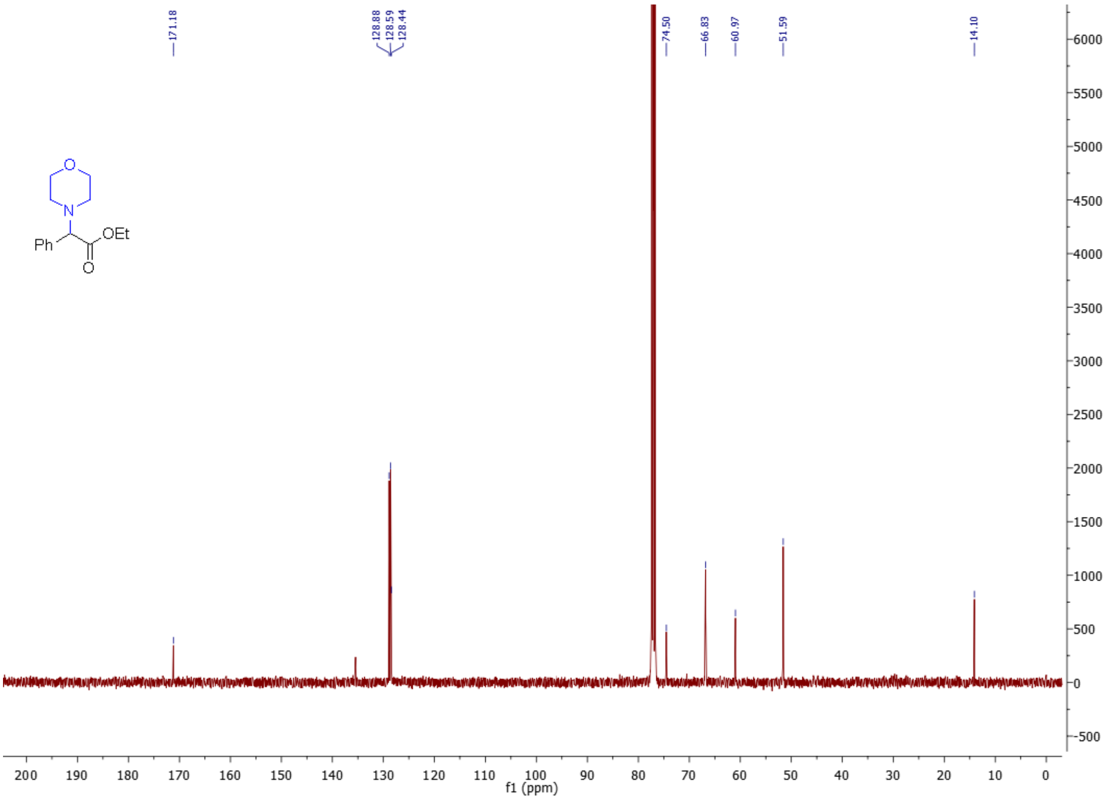

^{1}H NMR of Compound **20** ($CDCl_3$, 400 MHz):

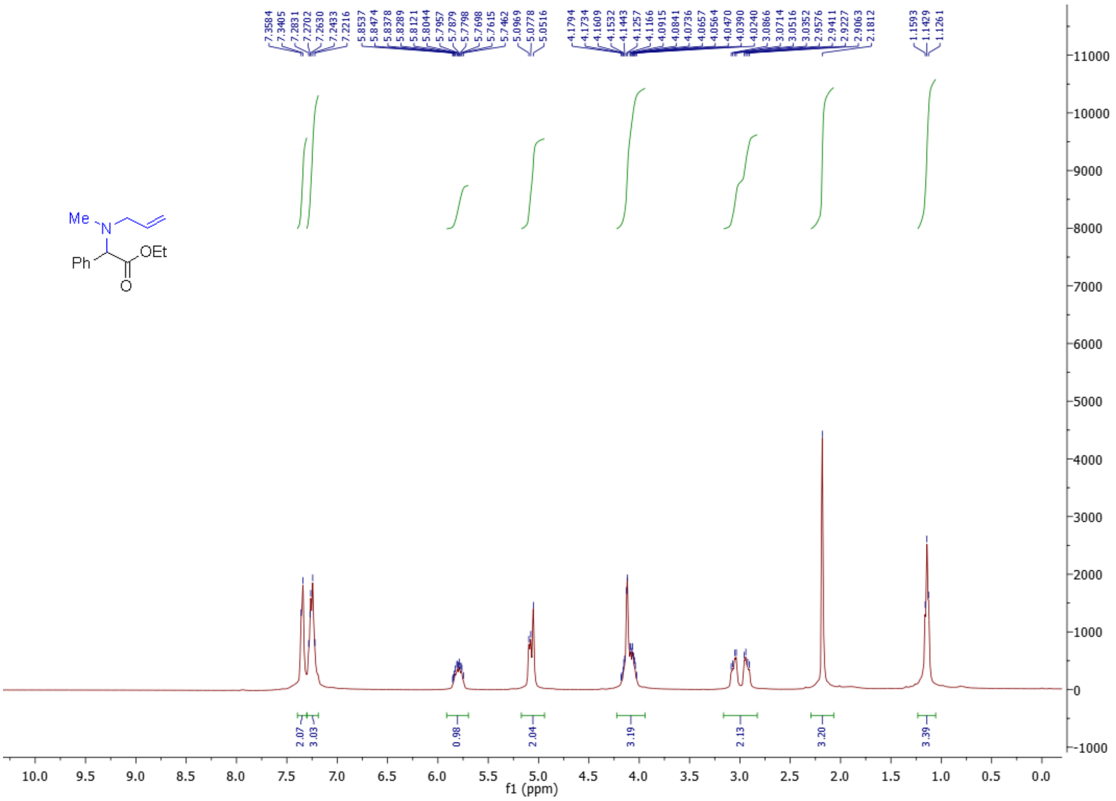

^{13}C NMR of Compound **20** ($CDCl_3$, 100 MHz):

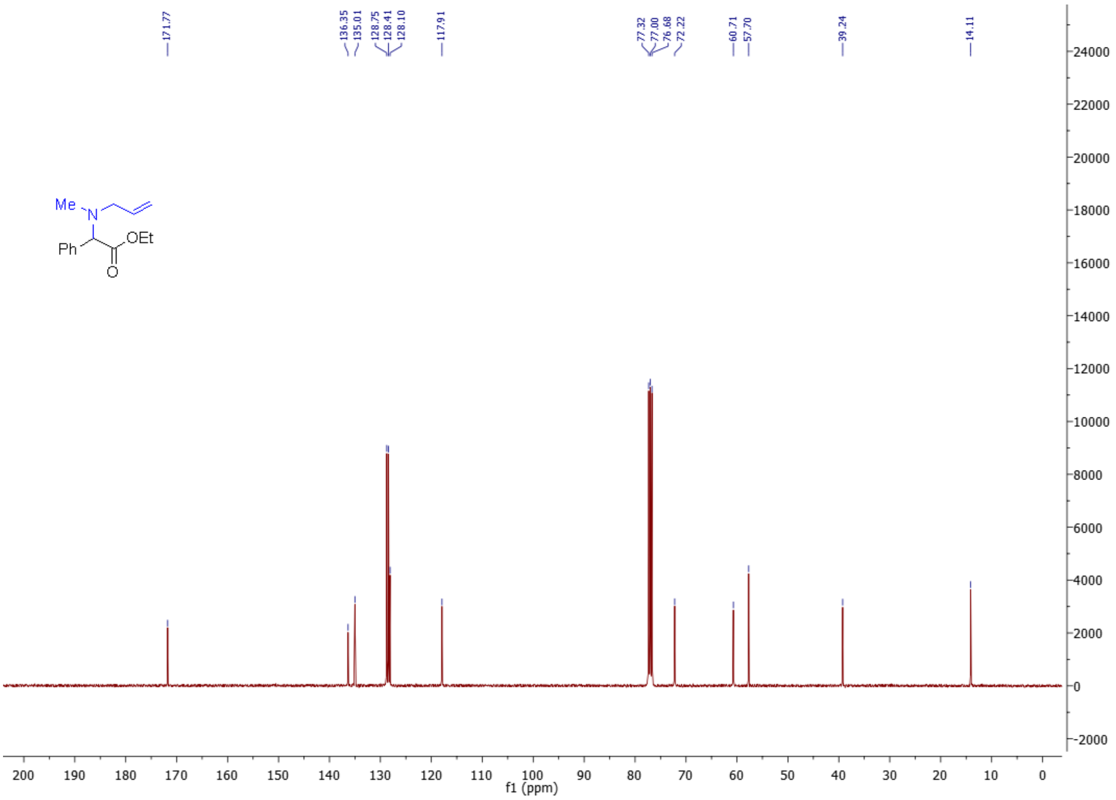

^{1}H NMR of Compound **21** ($CDCl_3$, 400 MHz):

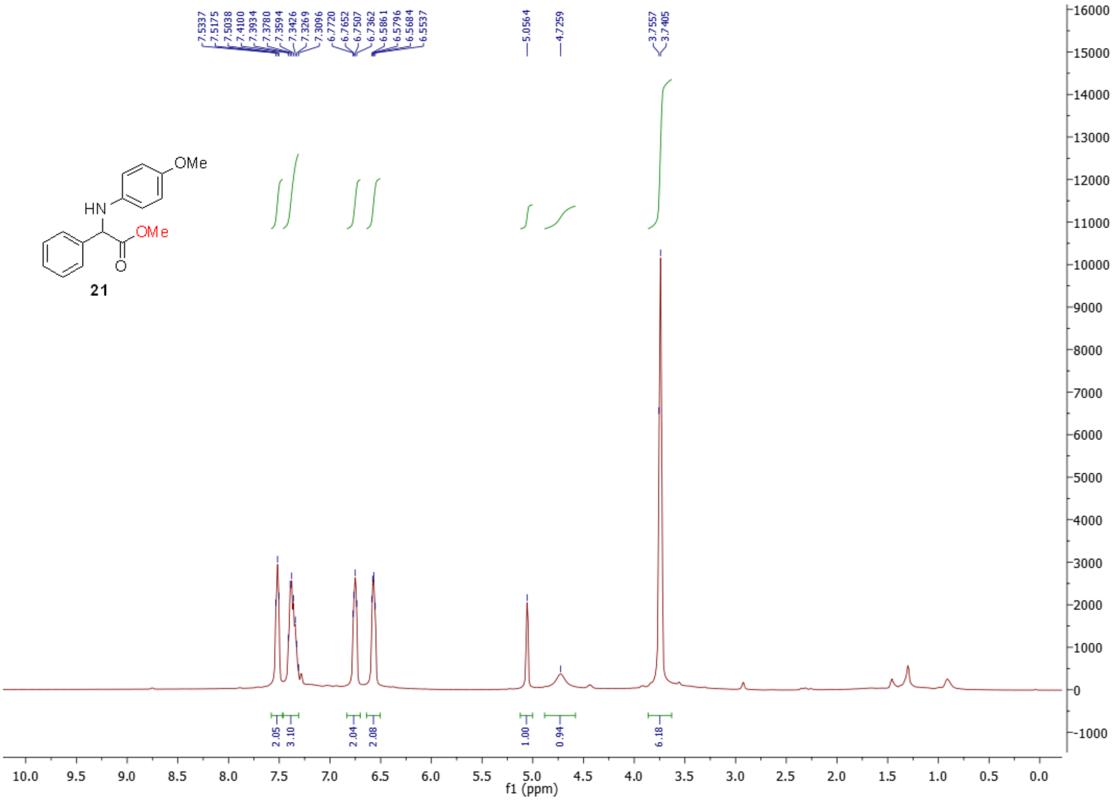

^{13}C NMR of Compound **21** ($CDCl_3$, 100 MHz):

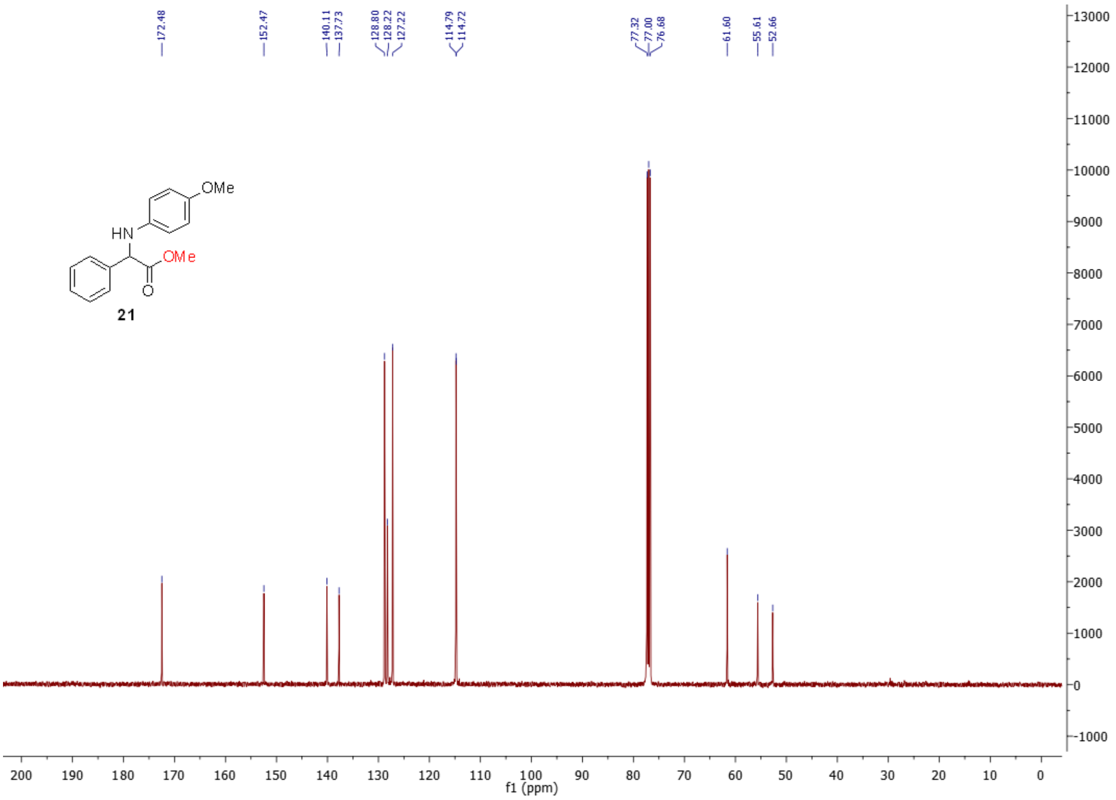

^{1}H NMR of Compound **22** ($CDCl_3$, 400 MHz):

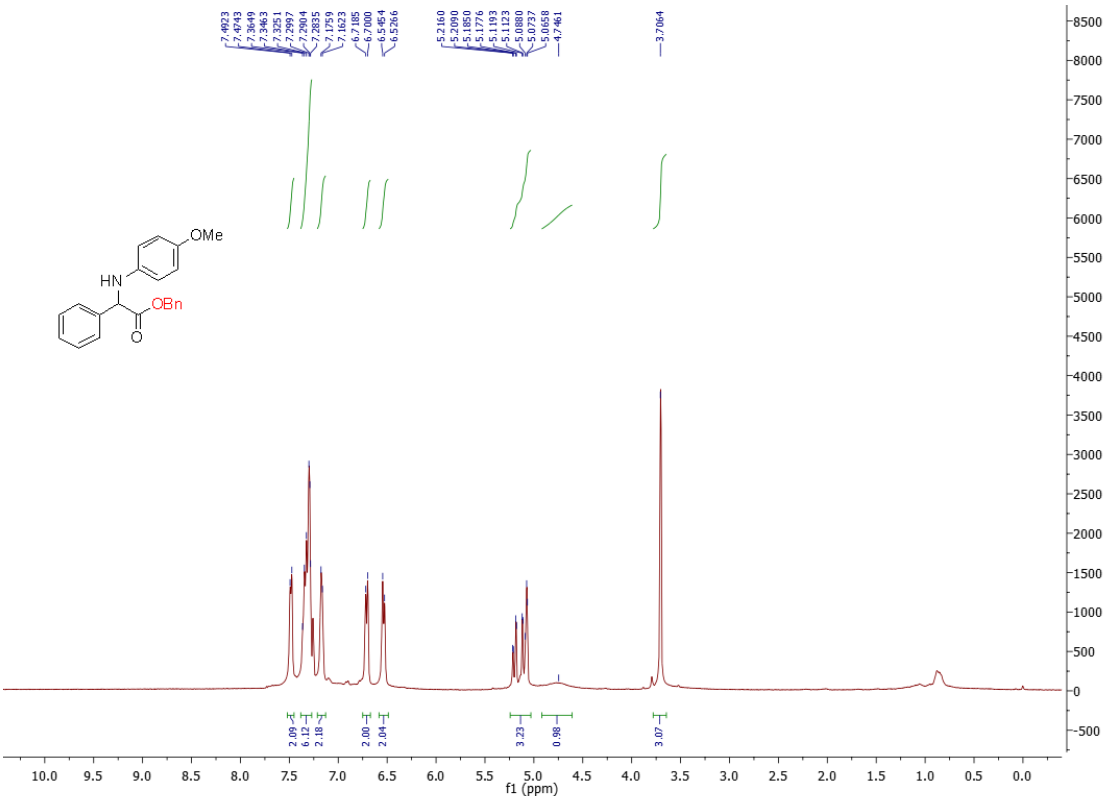

^{13}C NMR of Compound **22** ($CDCl_3$, 100 MHz):

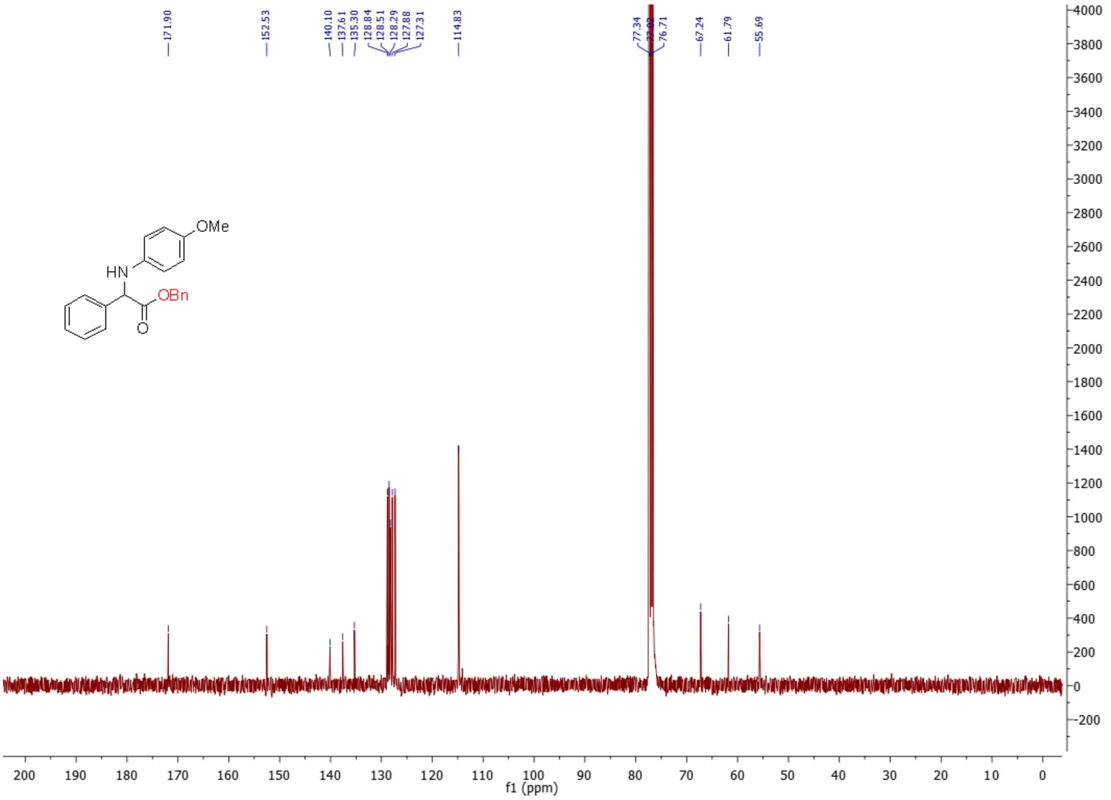

^{1}H NMR of Compound **23** ($CDCl_3$, 400 MHz):

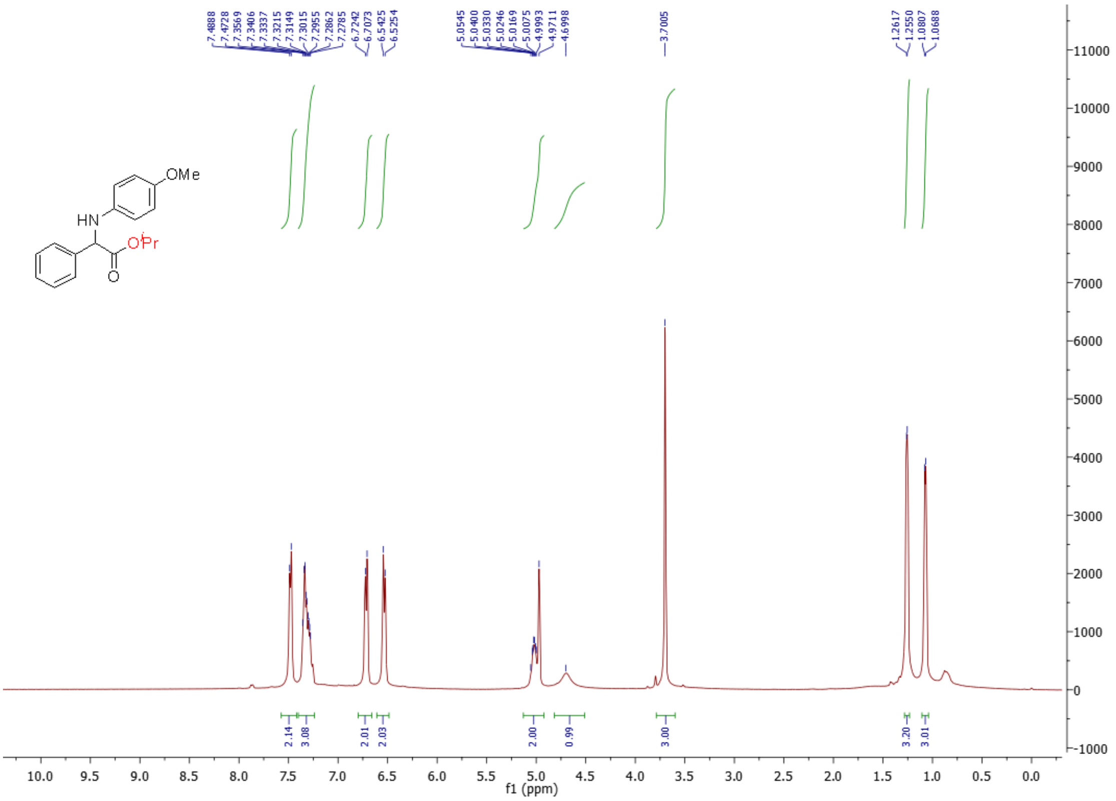

^{13}C NMR of Compound **23** ($CDCl_3$, 100 MHz):

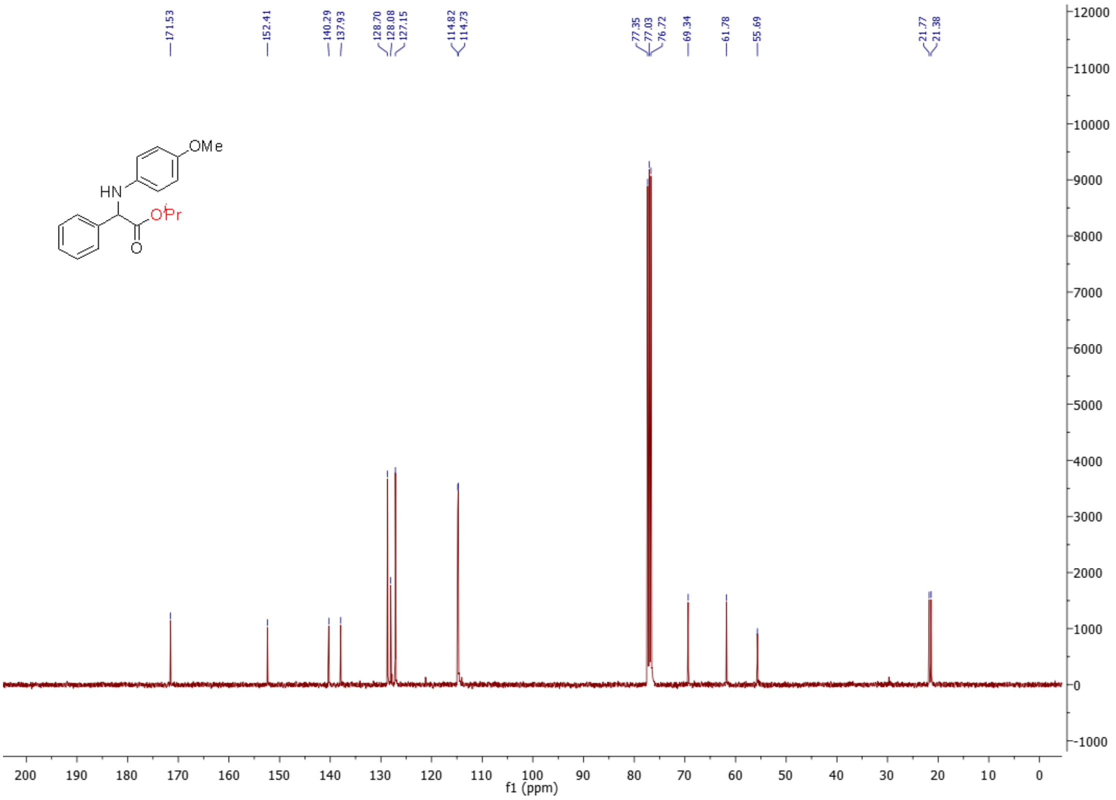

^{1}H NMR of Compound **24** ($CDCl_3$, 400 MHz):

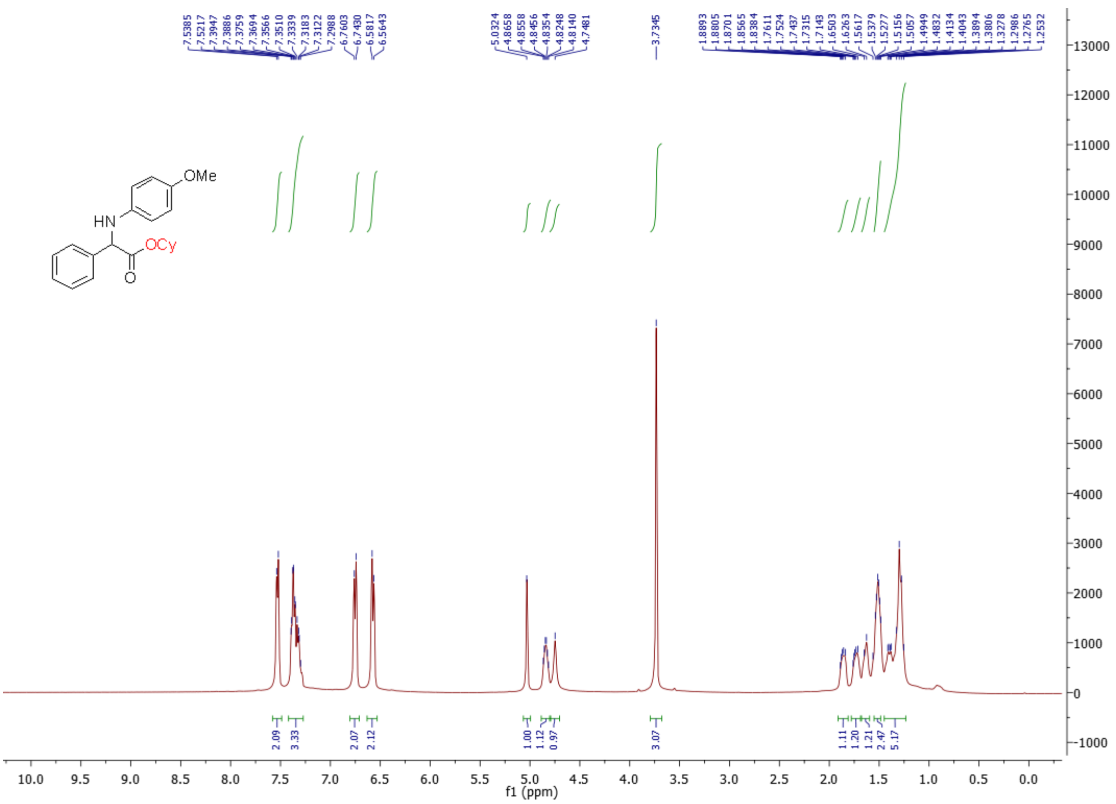

^{13}C NMR of Compound **24** ($CDCl_3$, 100 MHz):

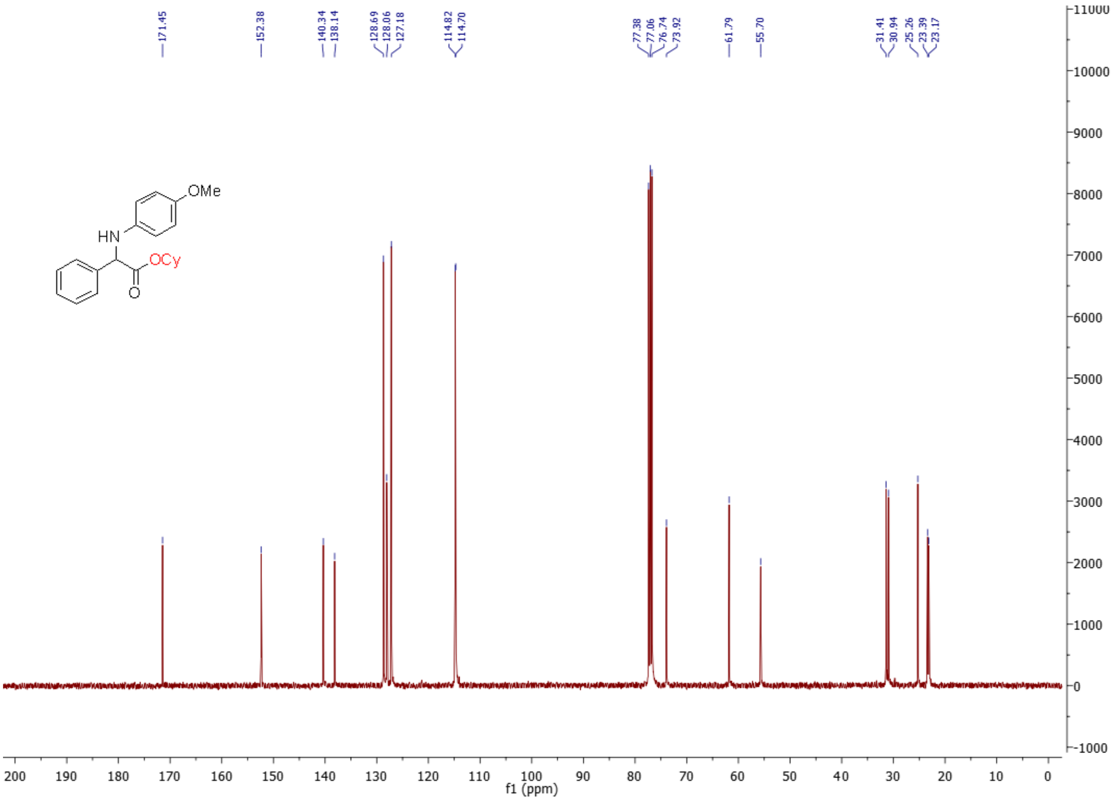

^{1}H NMR of Compound **25** ($CDCl_3$, 400 MHz):

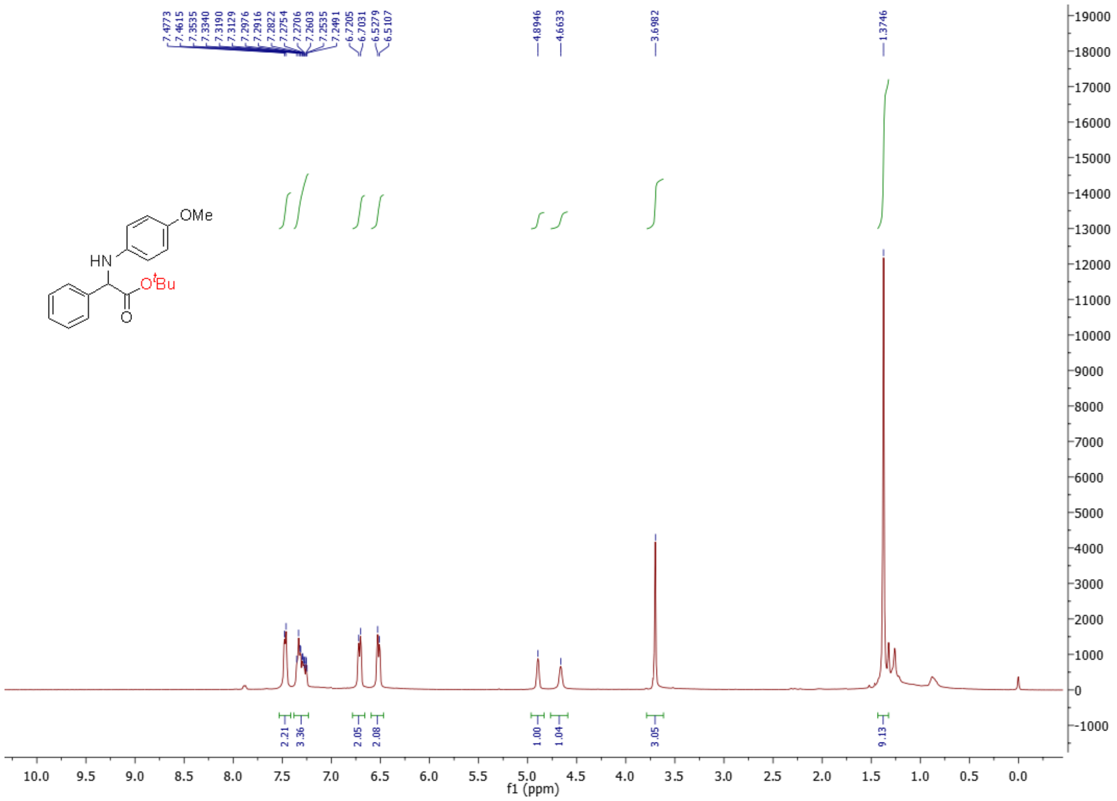

^{13}C NMR of Compound **25** ($CDCl_3$, 100 MHz):

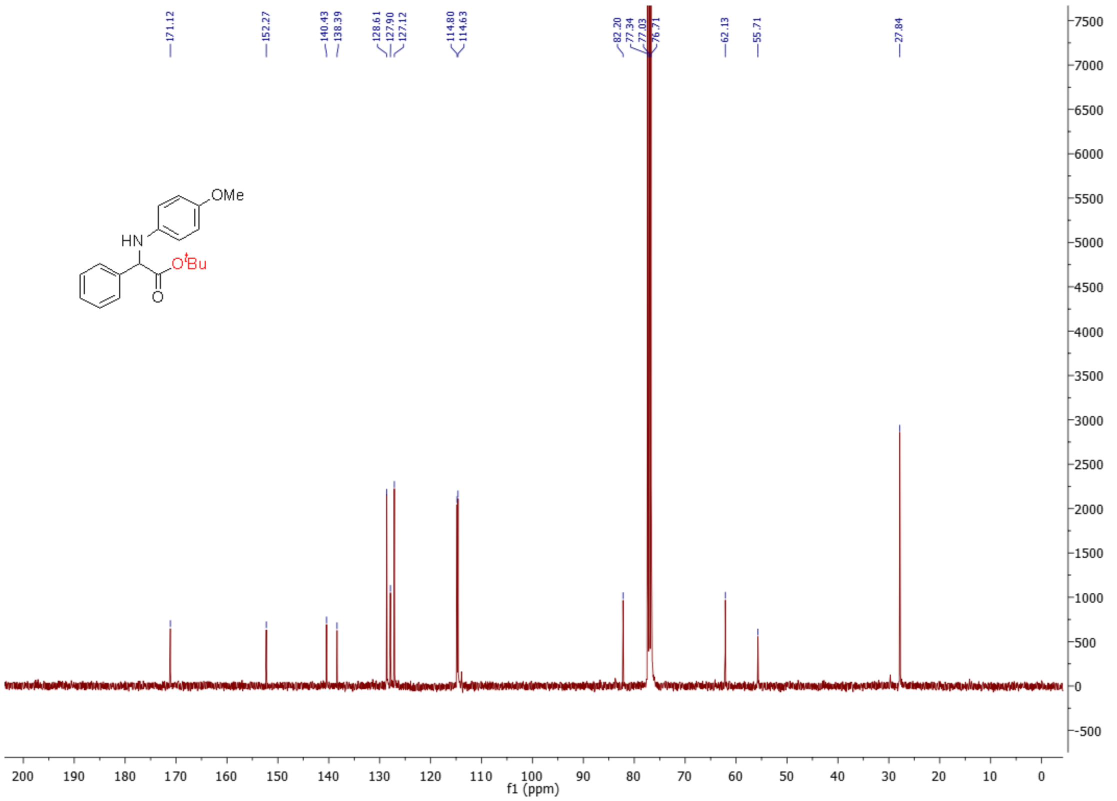

^{1}H NMR of Compound **26** ($CDCl_3$, 400 MHz):

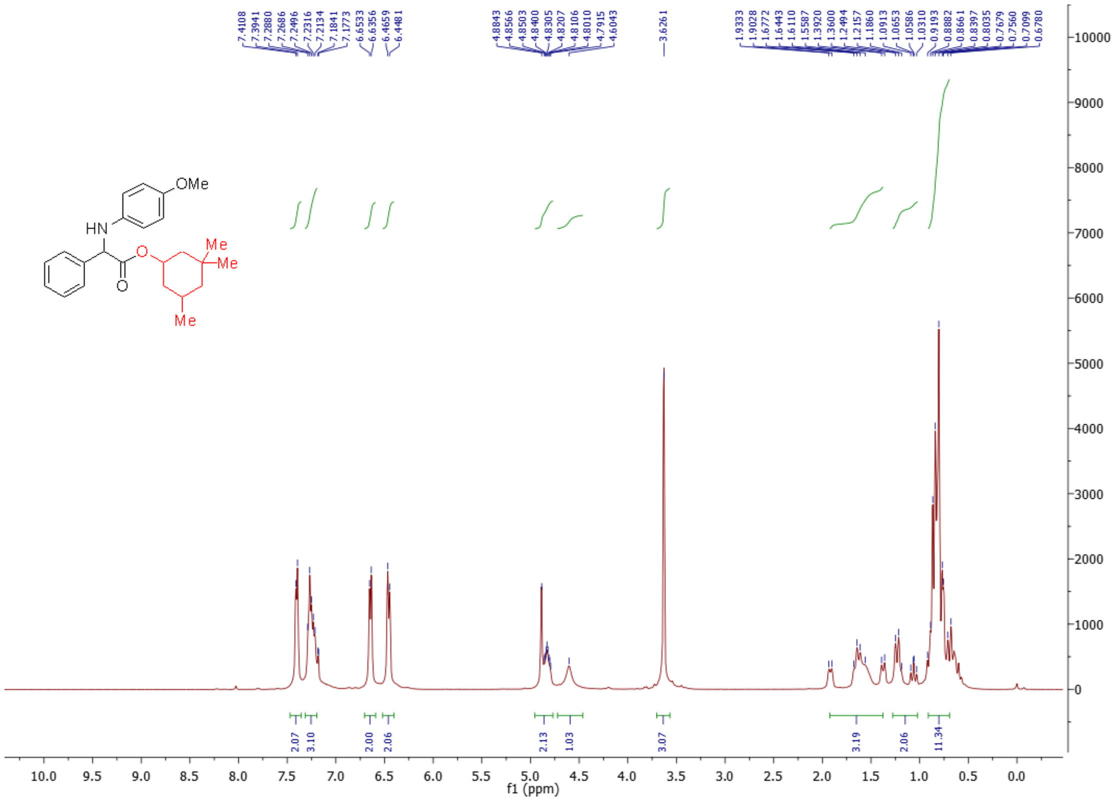

^{13}C NMR of Compound **26** ($CDCl_3$, 100 MHz):

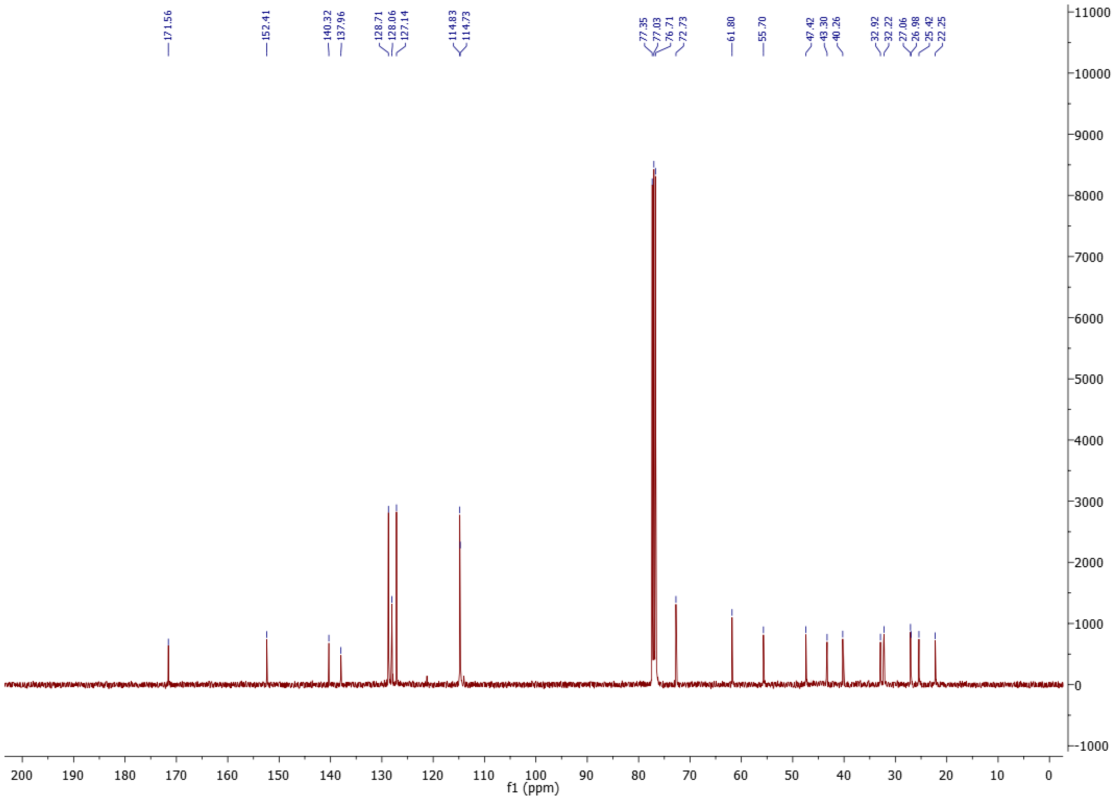

^{1}H NMR of Compound **27** ($CDCl_3$, 400 MHz):

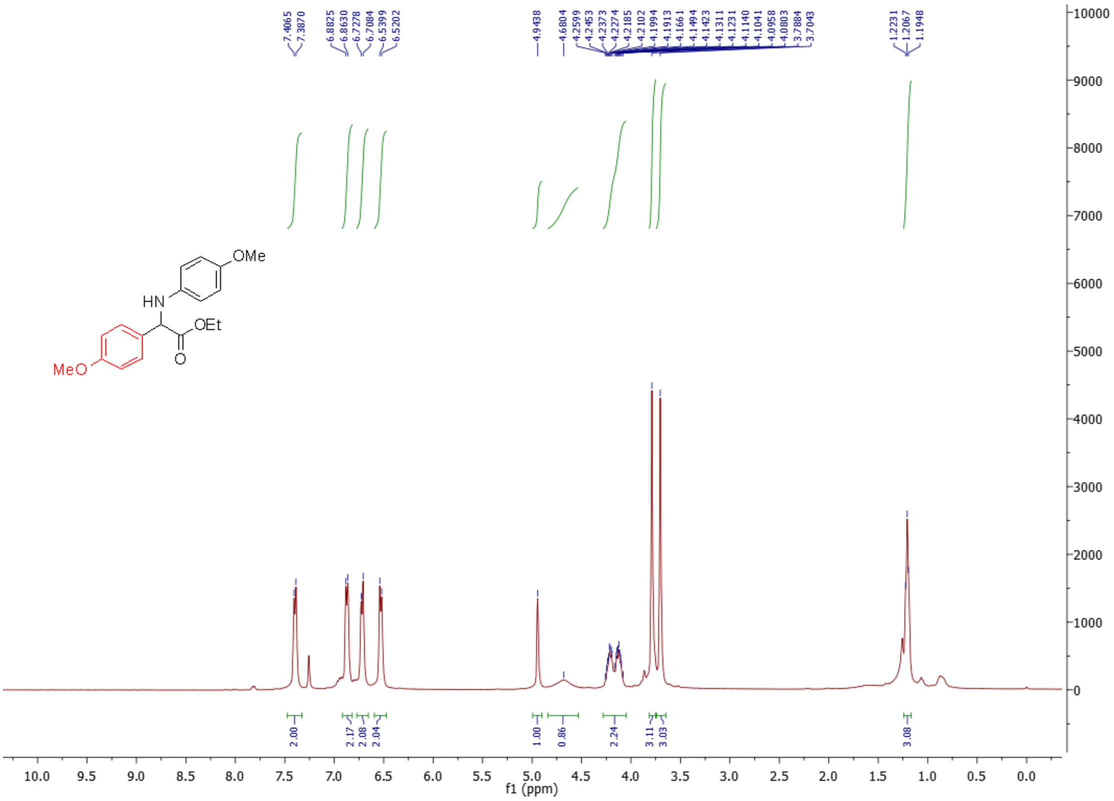

^{13}C NMR of Compound **27** ($CDCl_3$, 100 MHz):

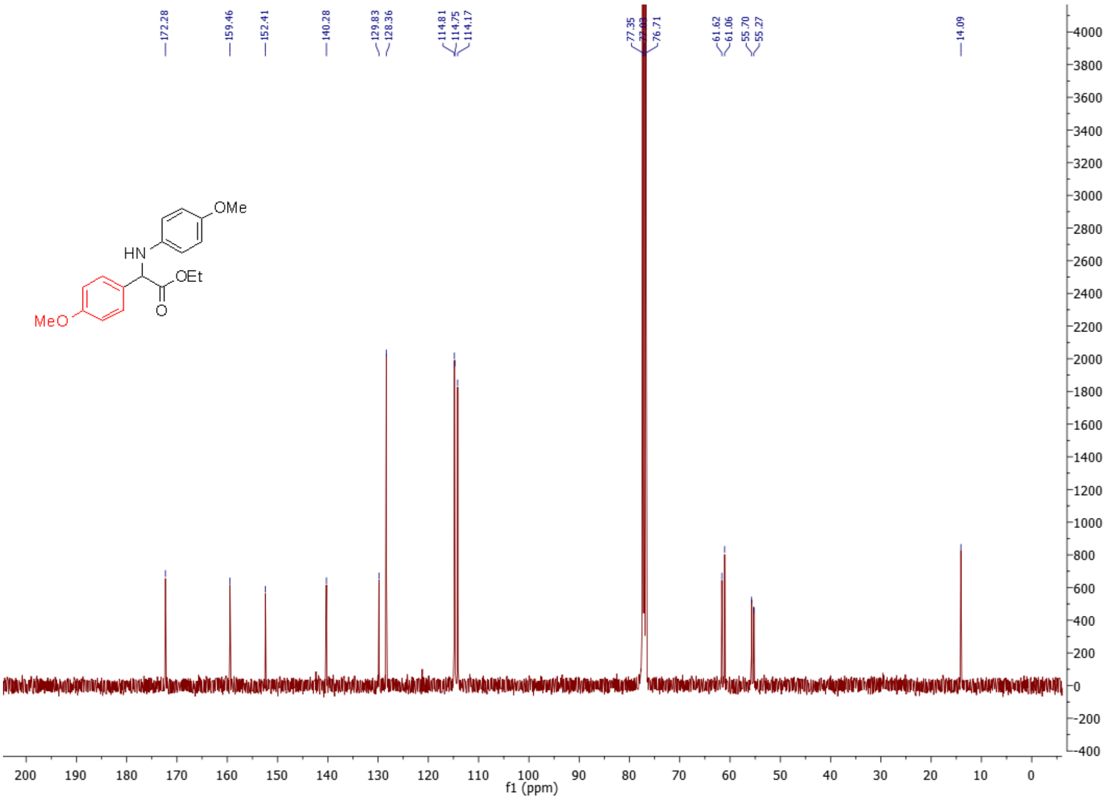

^{1}H NMR of Compound **28** ($CDCl_3$, 400 MHz):

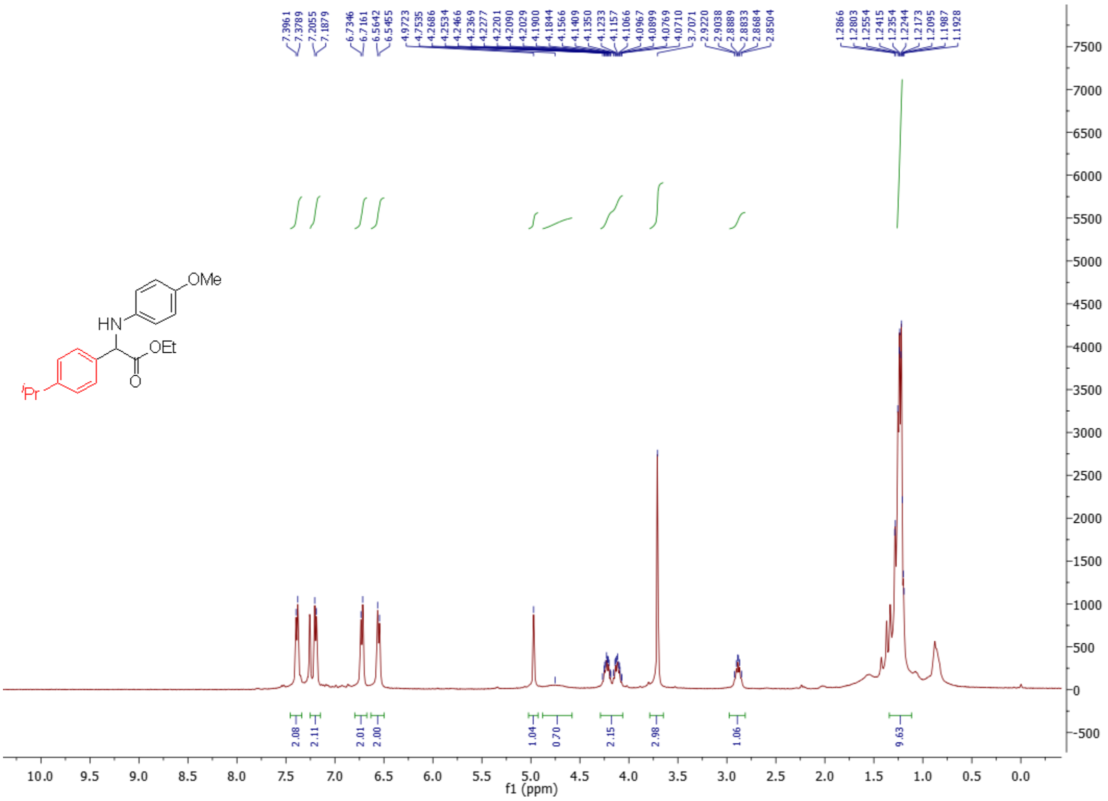

^{13}C NMR of Compound **28** ($CDCl_3$, 100 MHz):

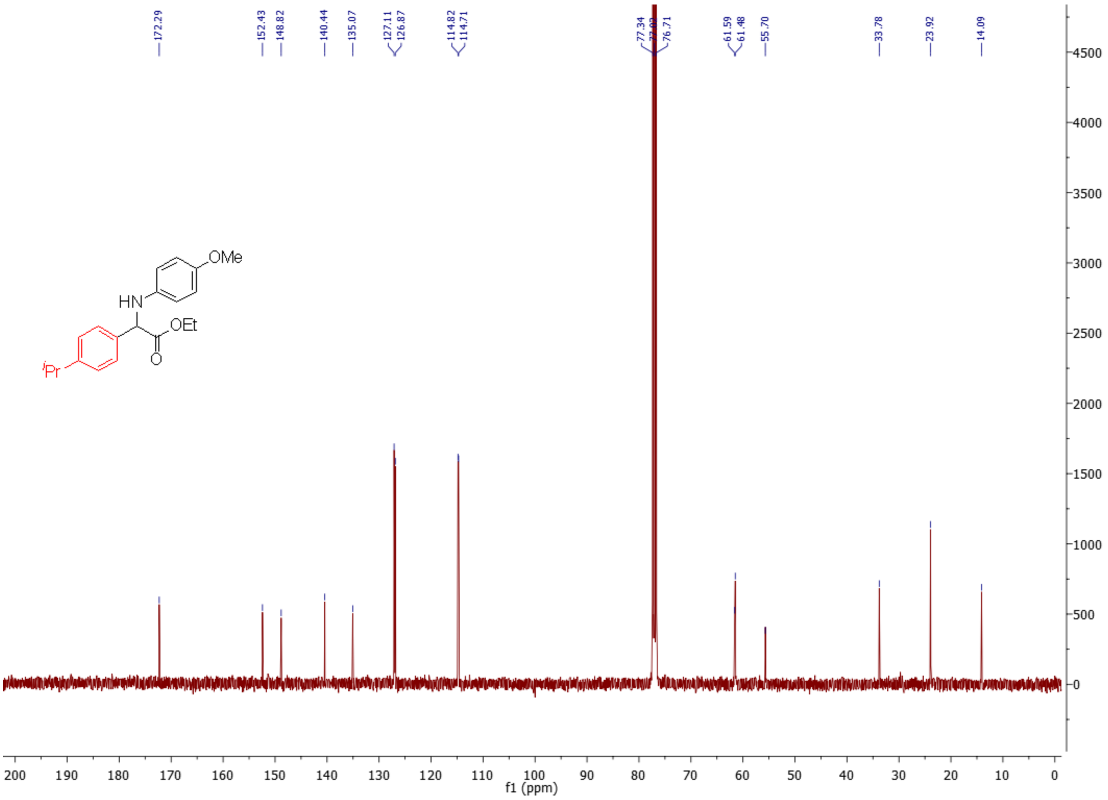

^{1}H NMR of Compound **29** ($CDCl_3$, 400 MHz):

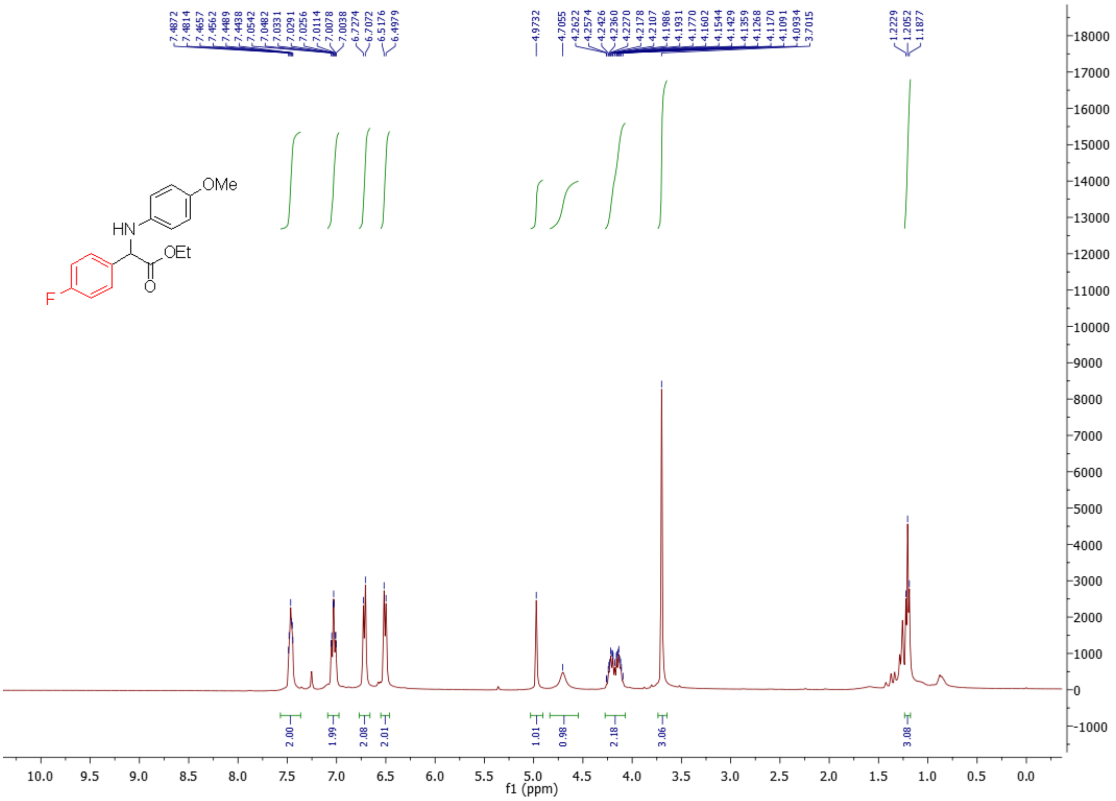

^{13}C NMR of Compound **29** ($CDCl_3$, 100 MHz):

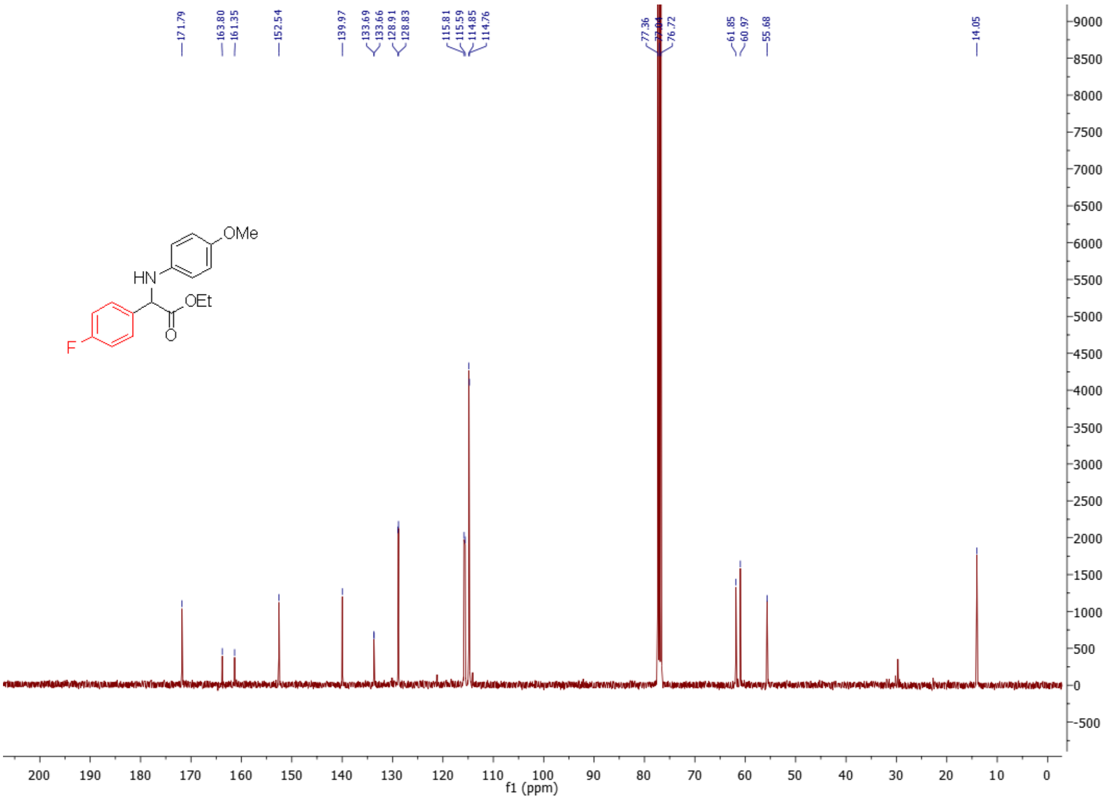

^{1}H NMR of Compound **30** ($CDCl_3$, 400 MHz):

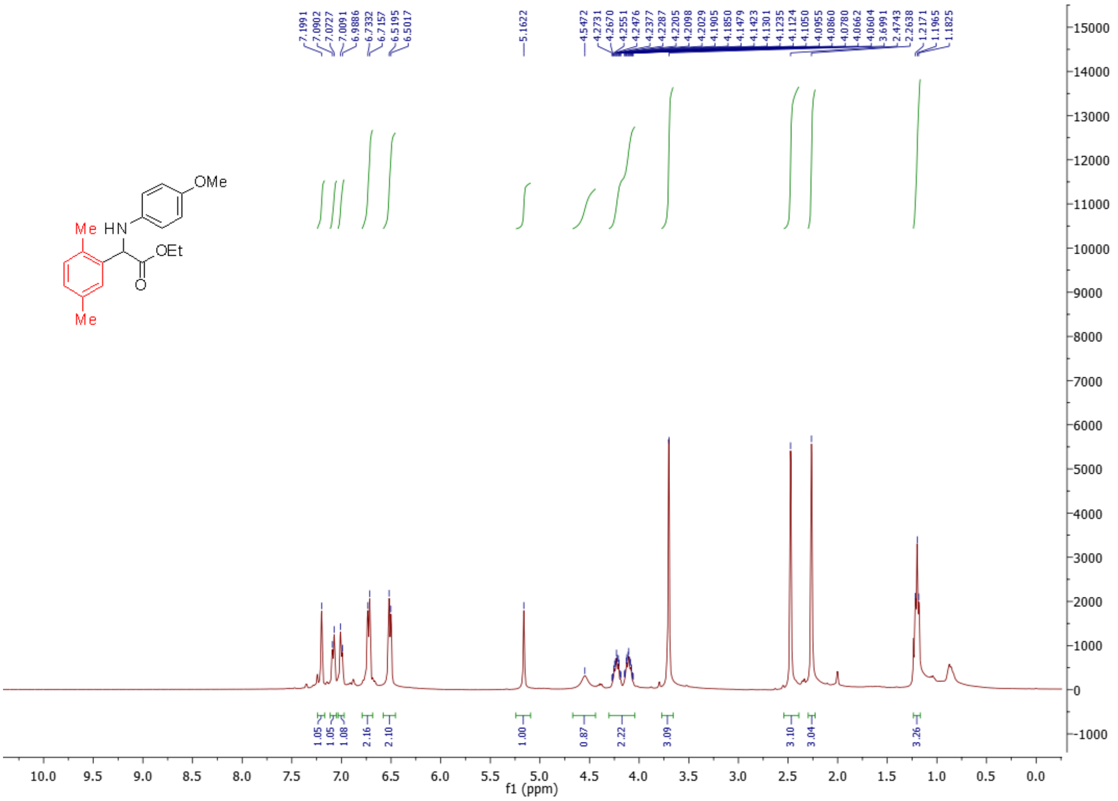

^{13}C NMR of Compound **30** ($CDCl_3$, 100 MHz):

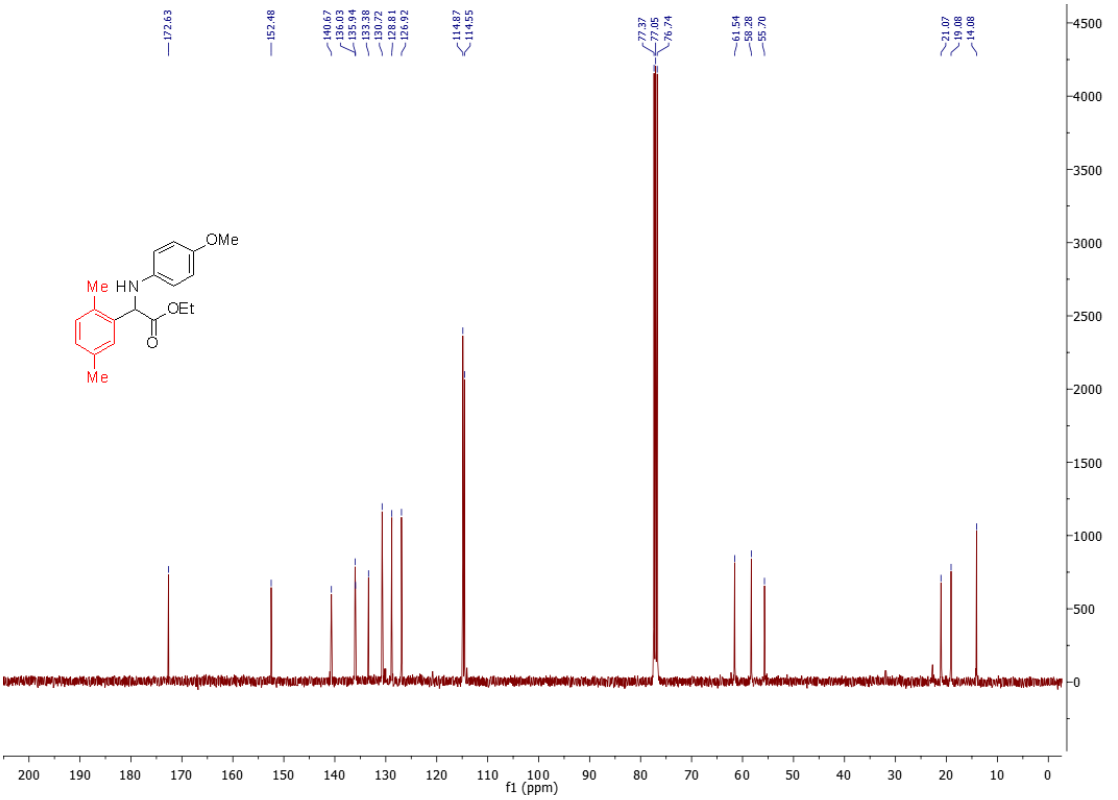

^{1}H NMR of Compound **31** ($CDCl_3$, 400 MHz):

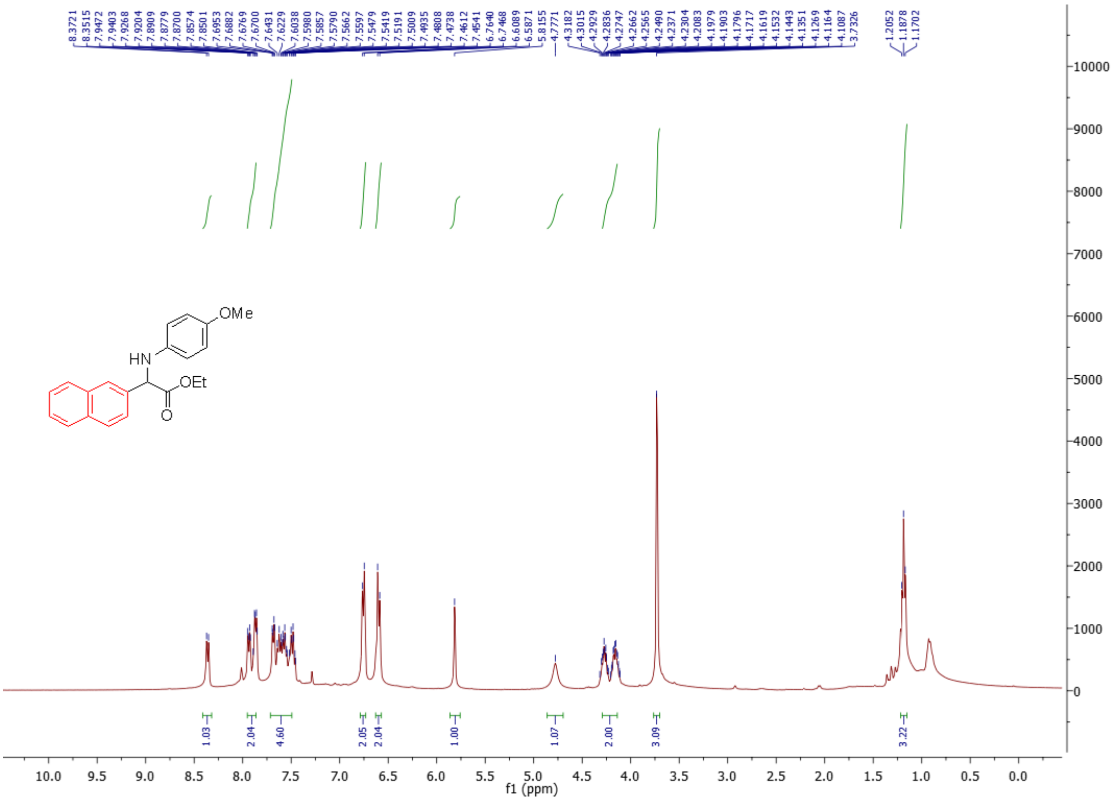

^{13}C NMR of Compound **31** ($CDCl_3$, 100 MHz):

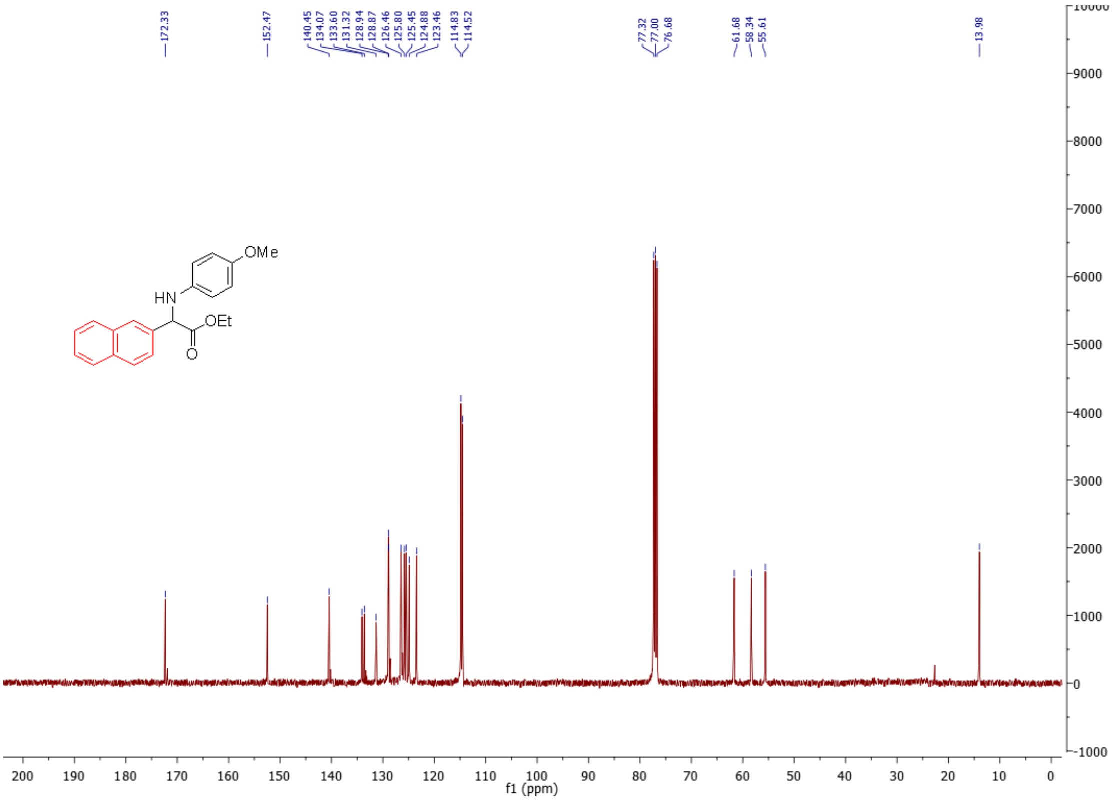

^{1}H NMR of Compound **32** ($CDCl_3$, 400 MHz):

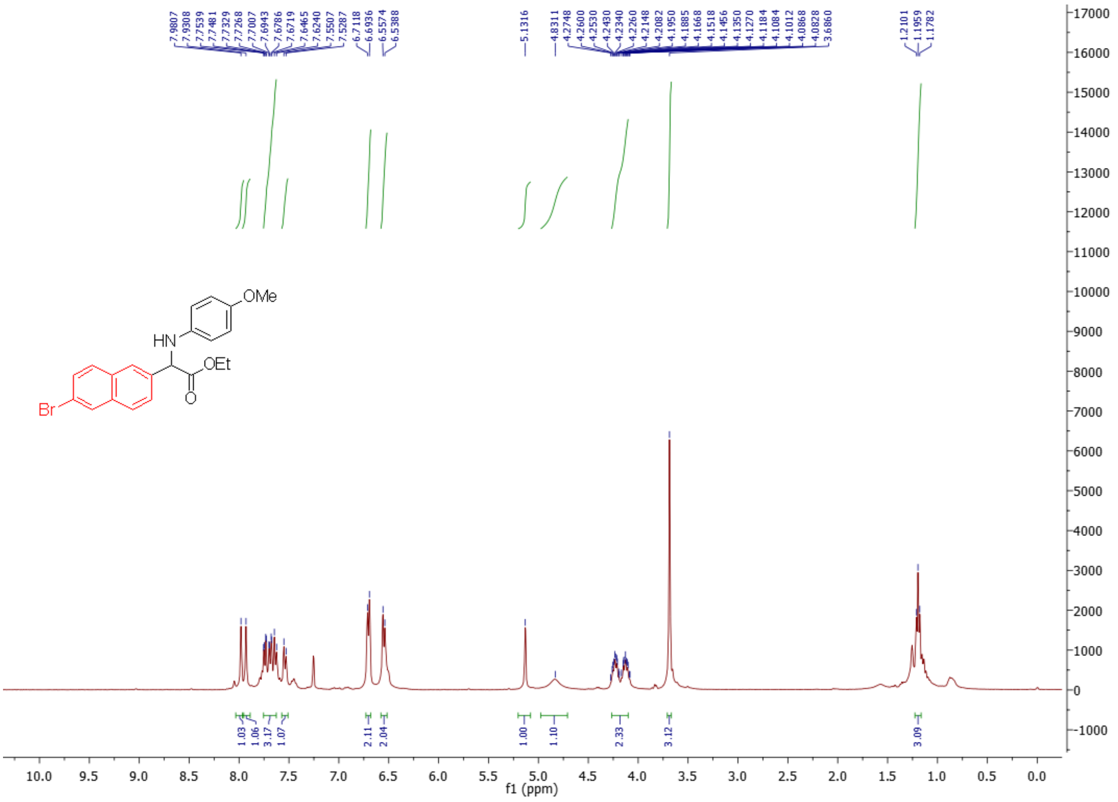

^{13}C NMR of Compound **32** ($CDCl_3$, 100 MHz):

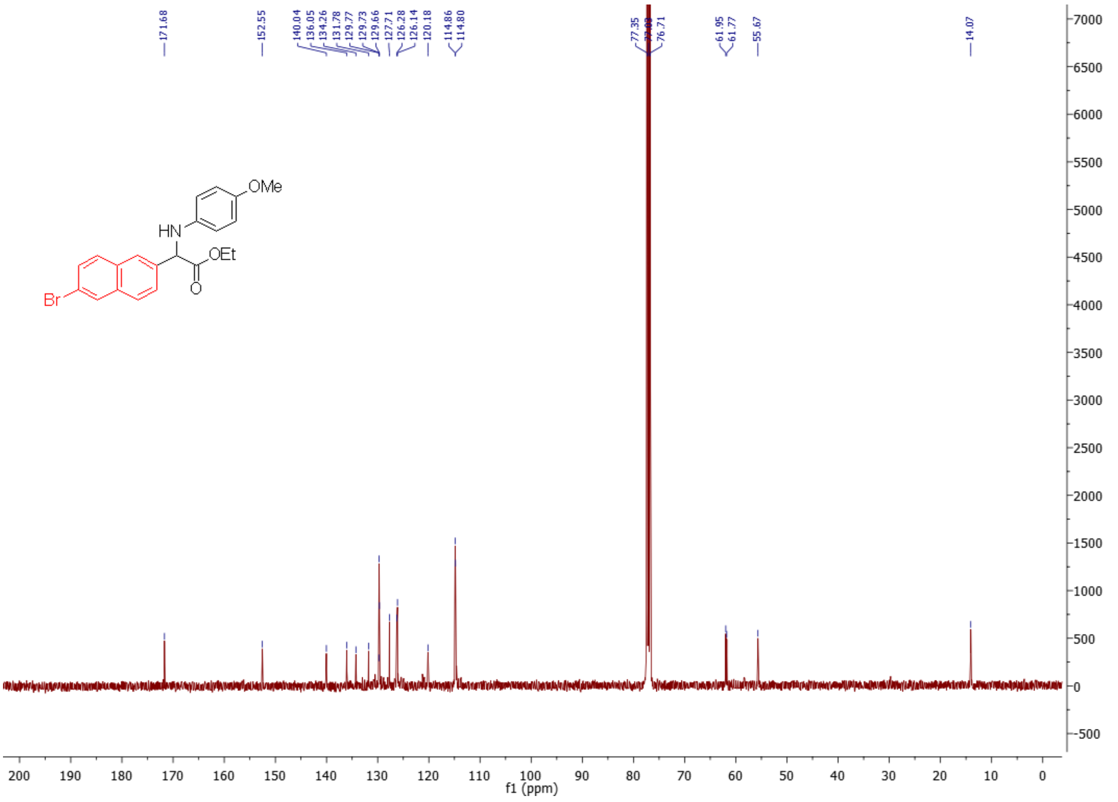

^{1}H NMR of Compound **33** ($CDCl_3$, 400 MHz):

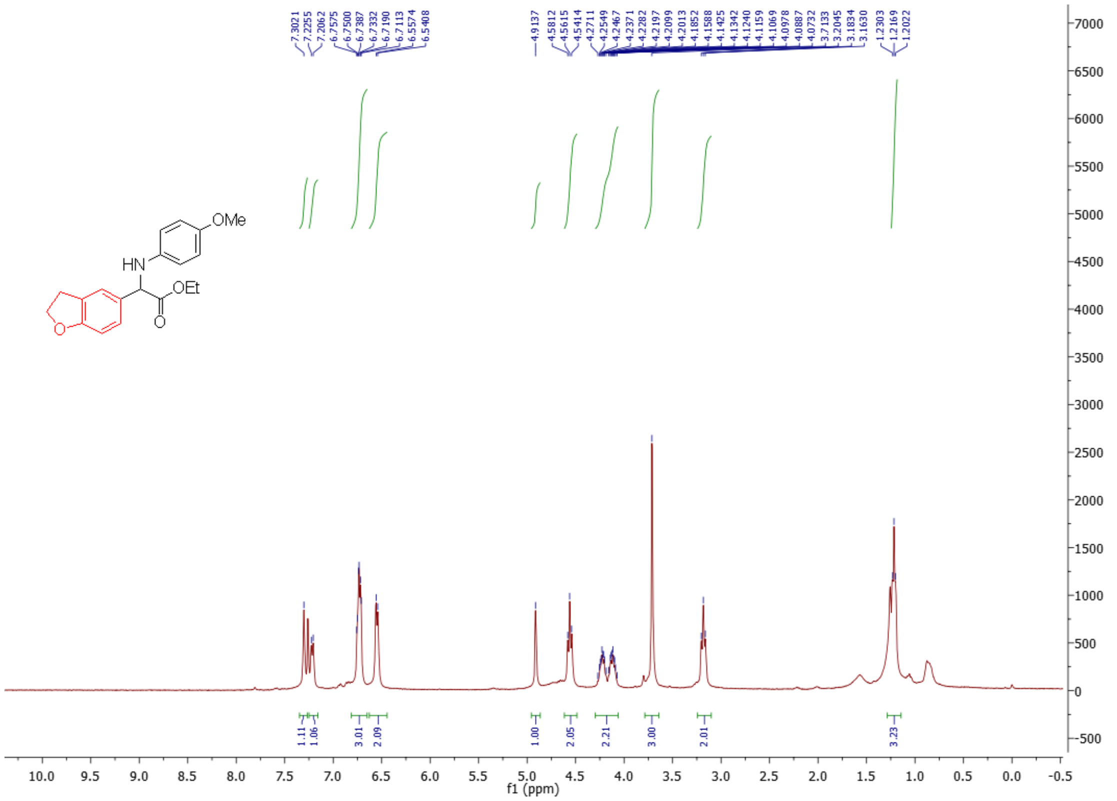

^{13}C NMR of Compound **33** ($CDCl_3$, 100 MHz):

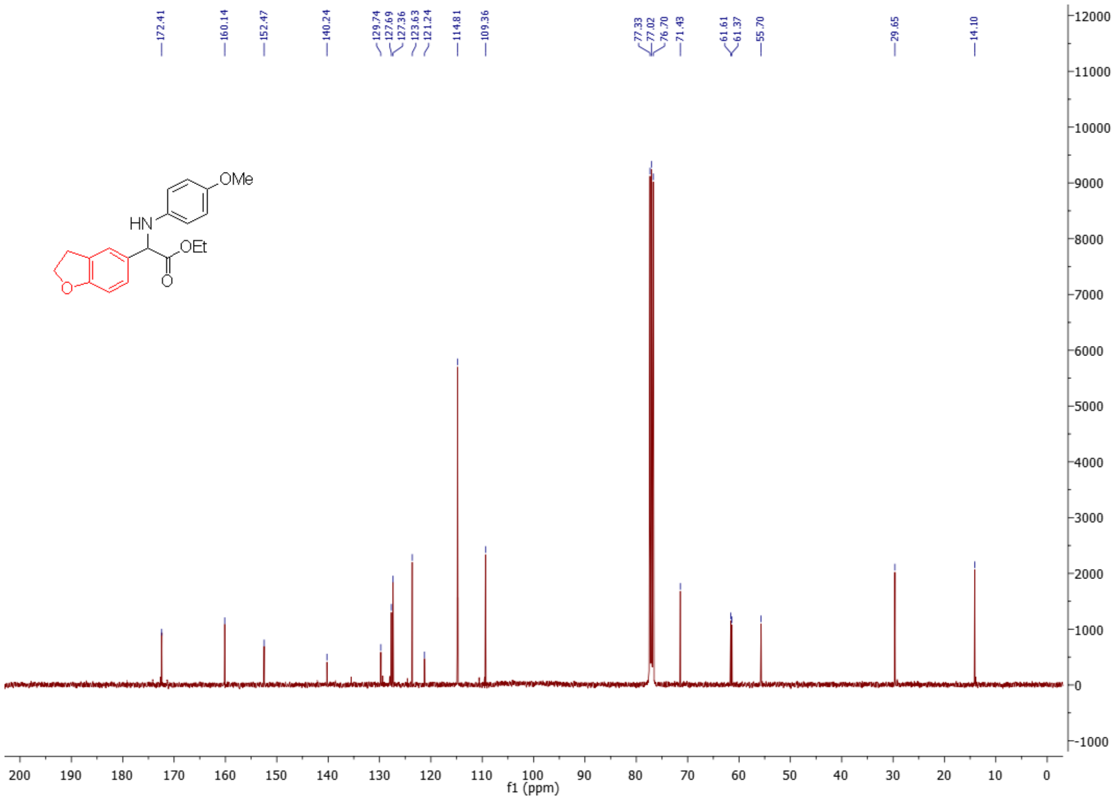

^{1}H NMR of Compound **34** ($CDCl_3$, 400 MHz):

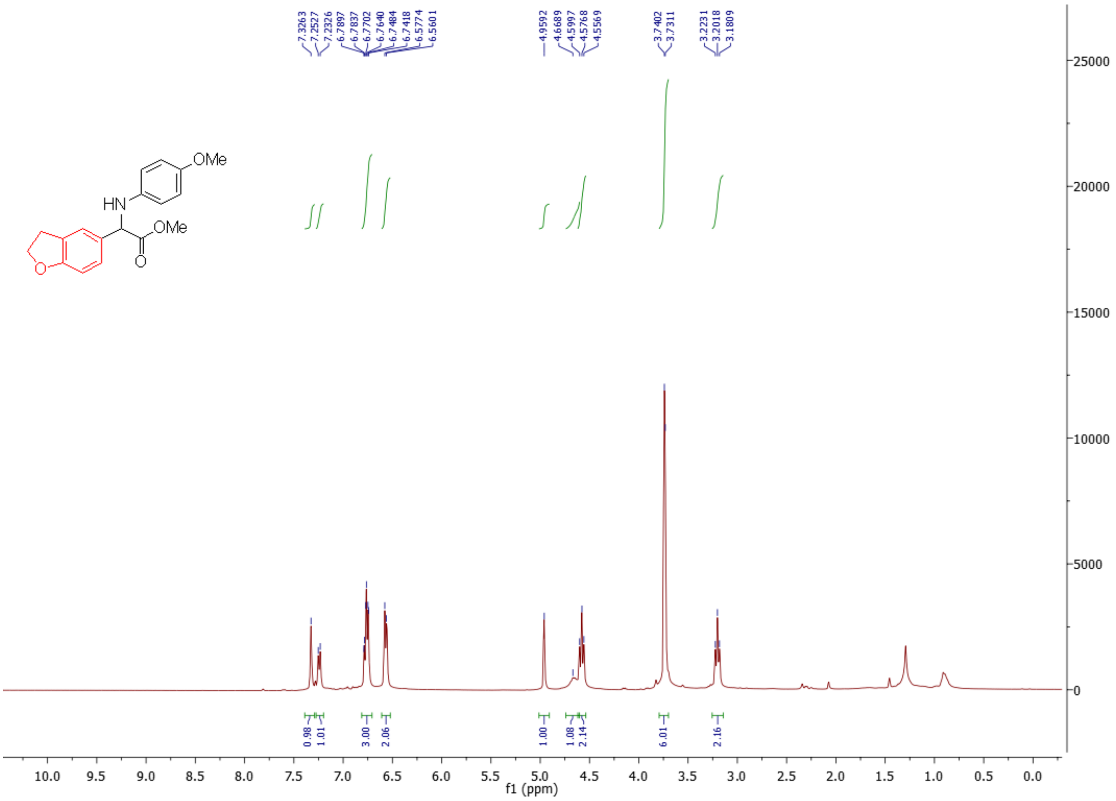

^{13}C NMR of Compound **34** ($CDCl_3$, 100 MHz):

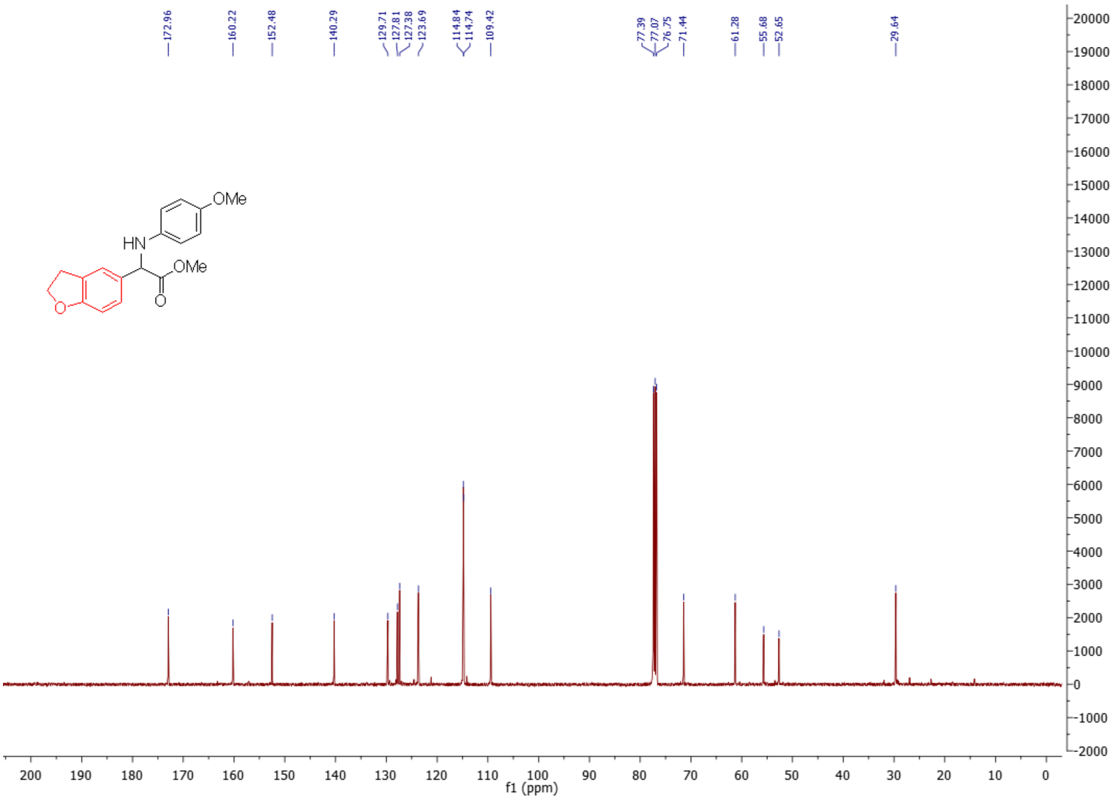

^{1}H NMR of Compound **35** ($CDCl_3$, 400 MHz):

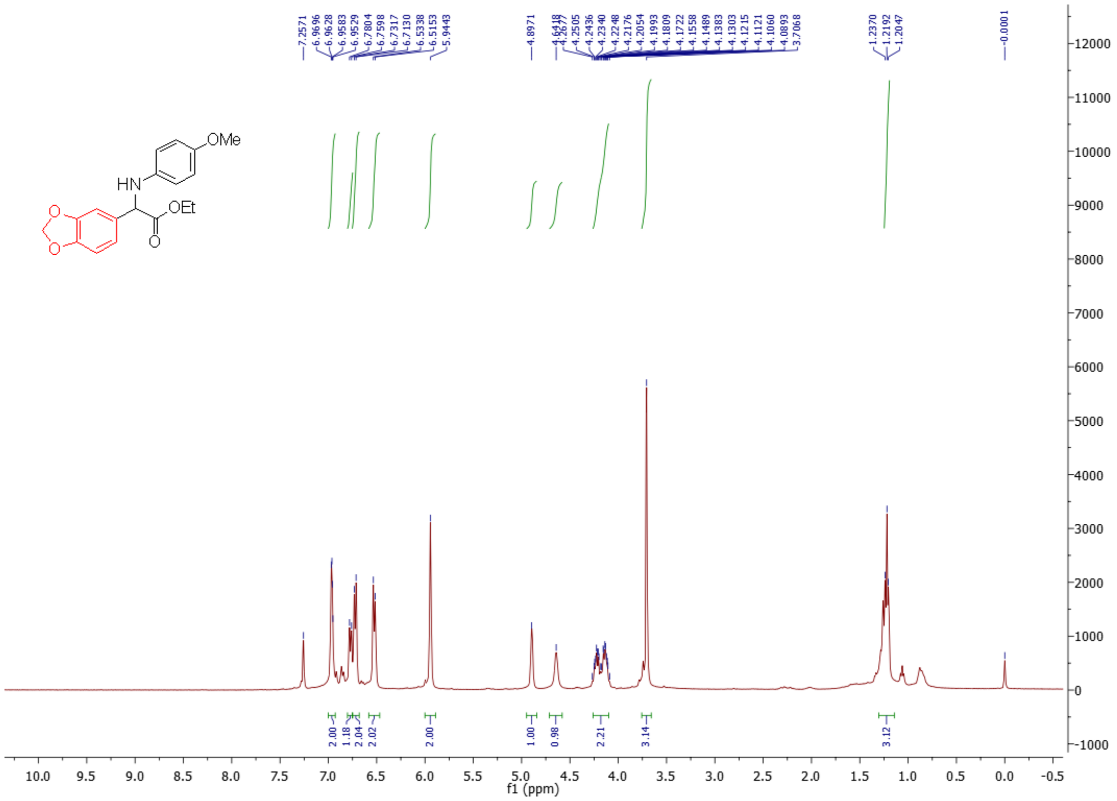

^{13}C NMR of Compound **35** ($CDCl_3$, 100 MHz):

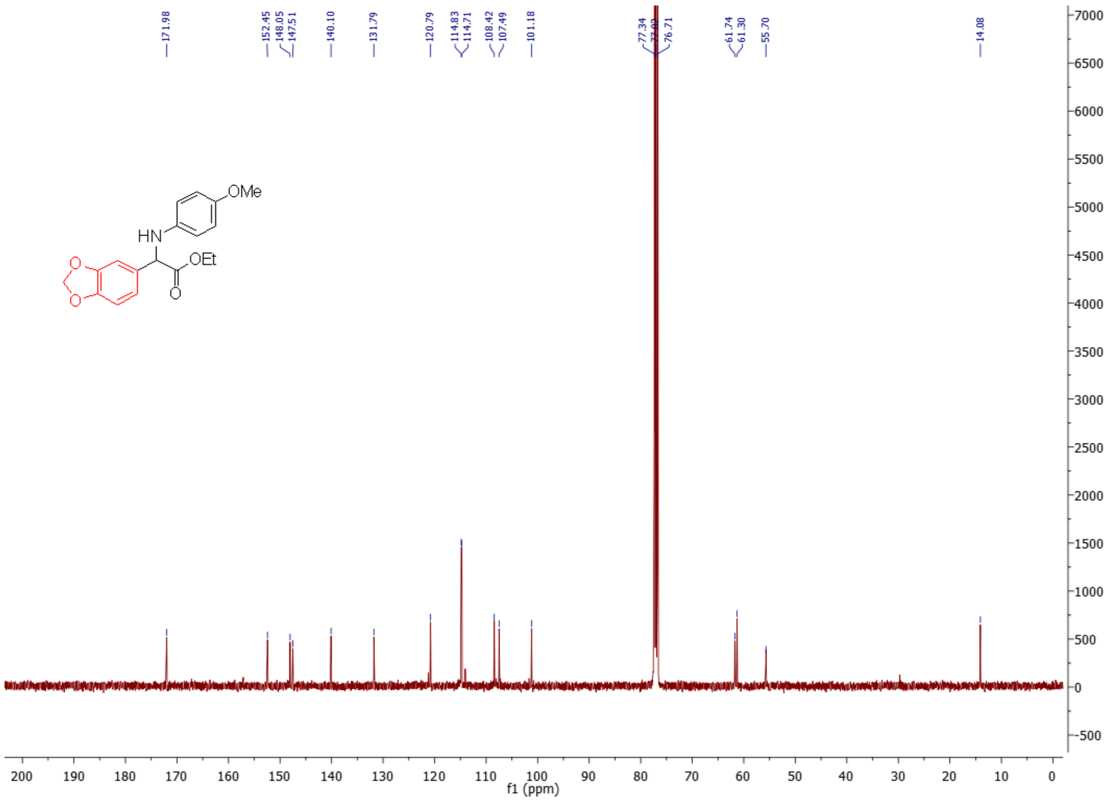

^{1}H NMR of Compound **36** ($CDCl_3$, 400 MHz):

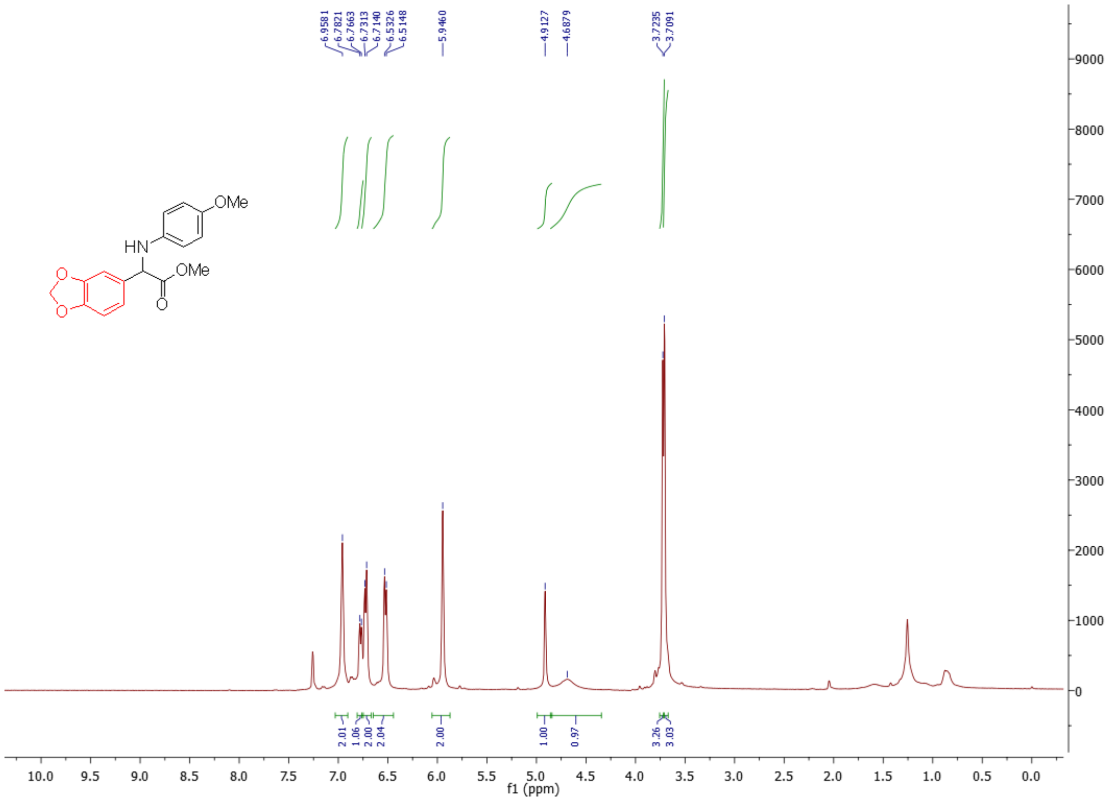

^{13}C NMR of Compound **36** ($CDCl_3$, 100 MHz):

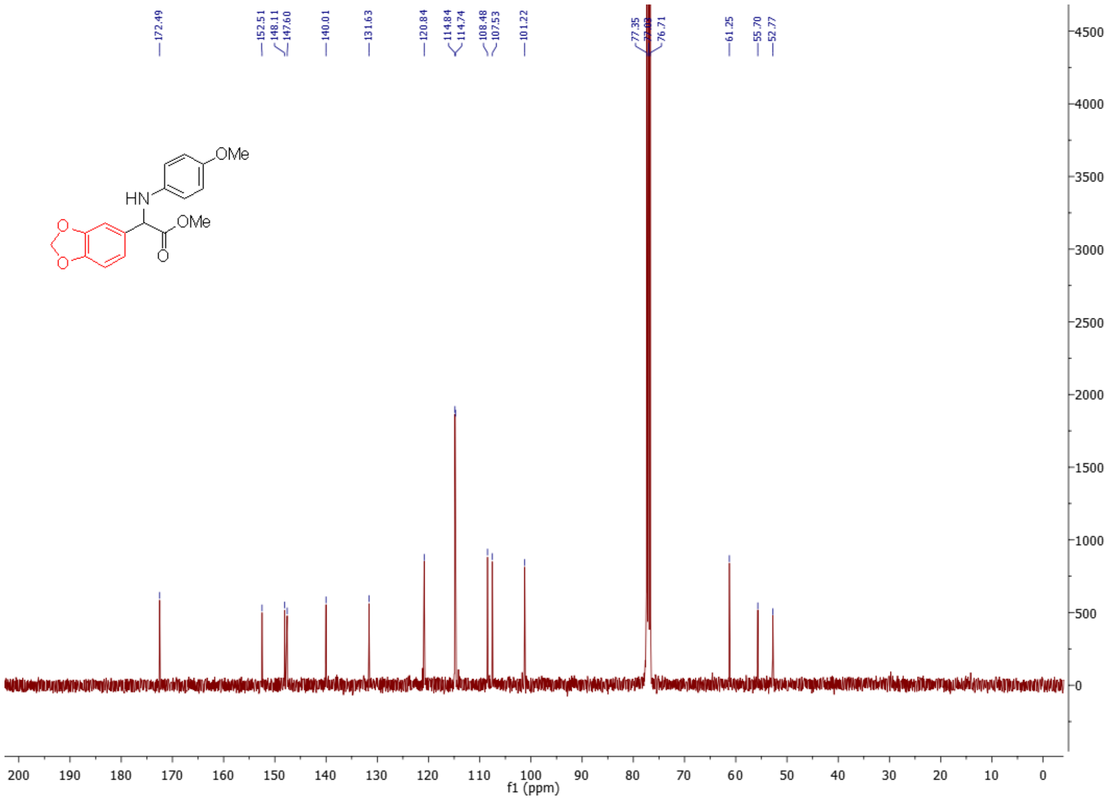

^{1}H NMR of Compound **37** ($CDCl_3$, 400 MHz):

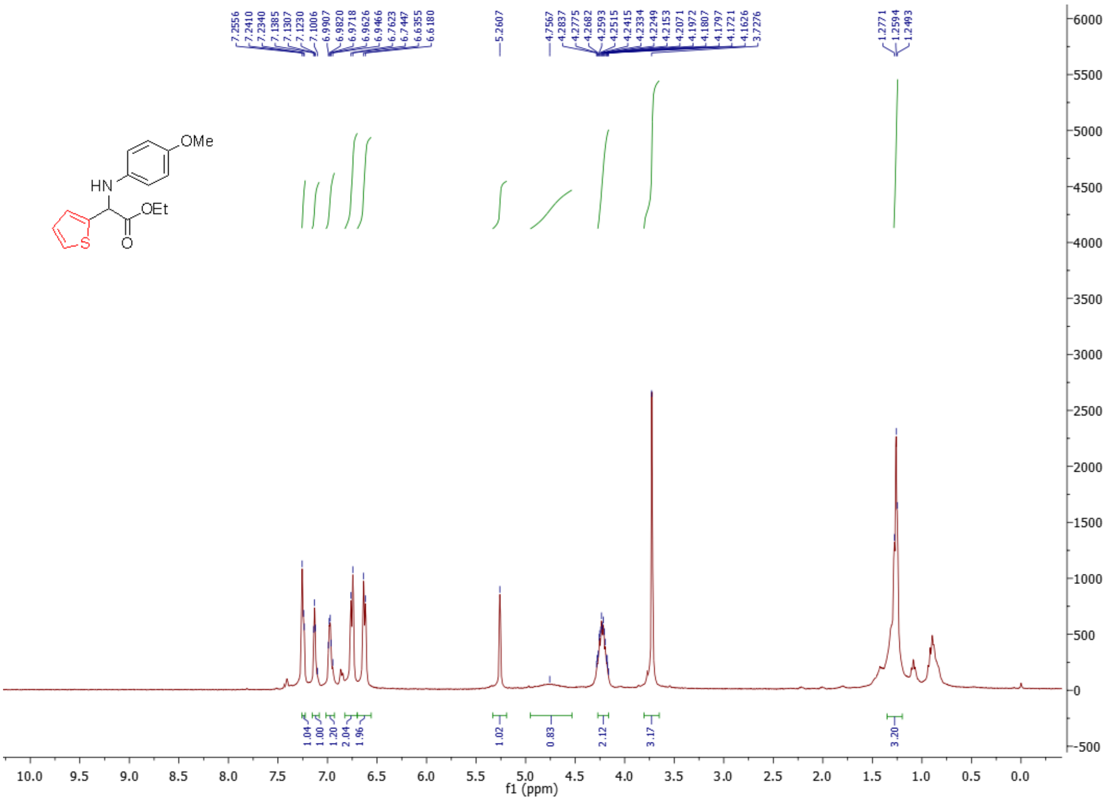

^{13}C NMR of Compound **37** ($CDCl_3$, 100 MHz):

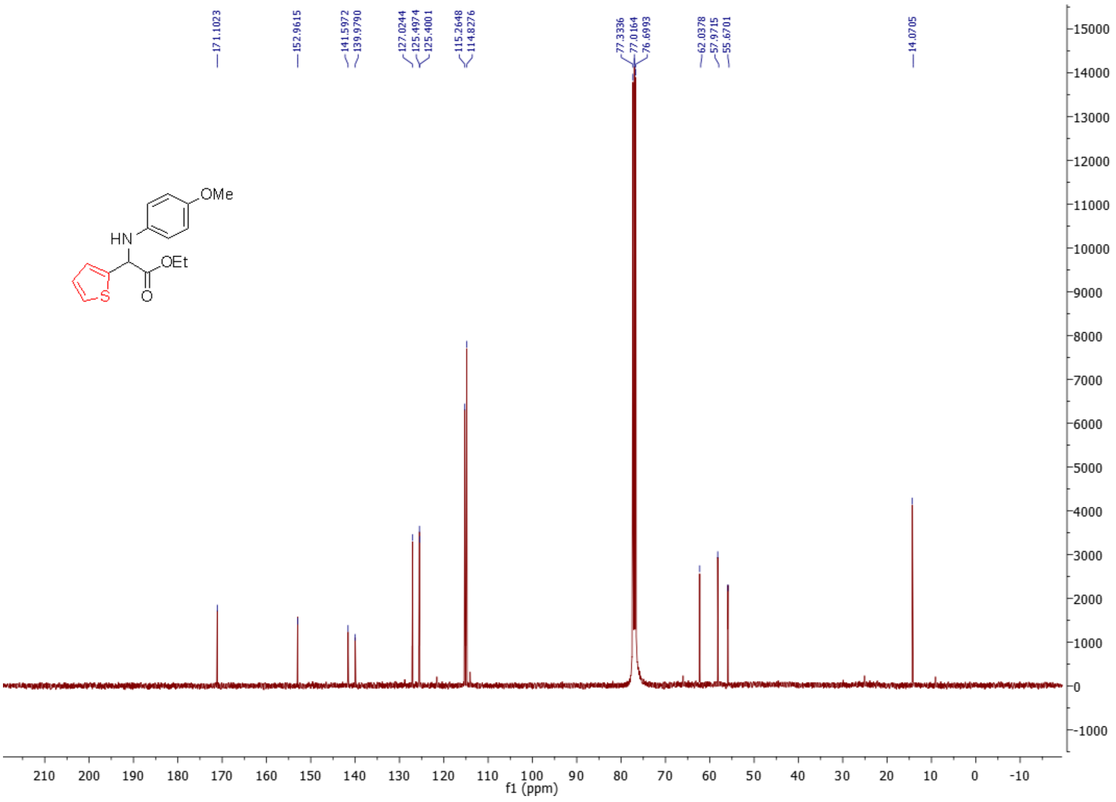

^{1}H NMR of Compound **38** ($CDCl_3$, 400 MHz):

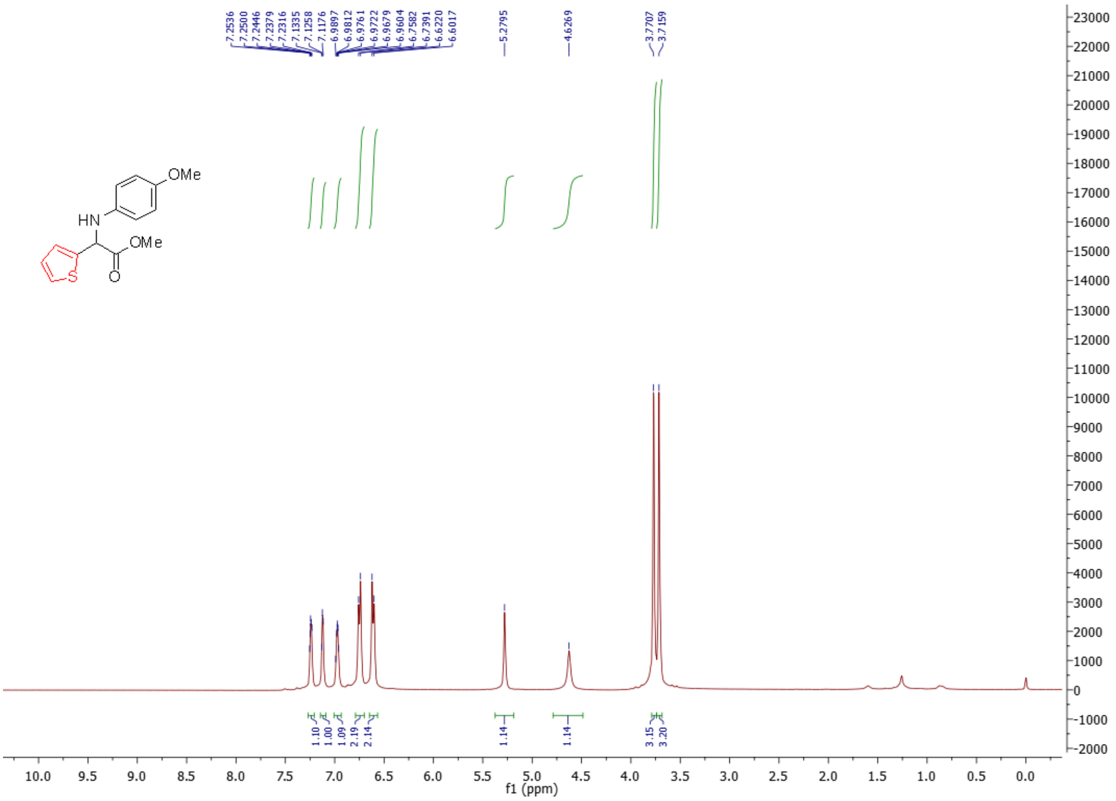

^{13}C NMR of Compound **38** ($CDCl_3$, 100 MHz):

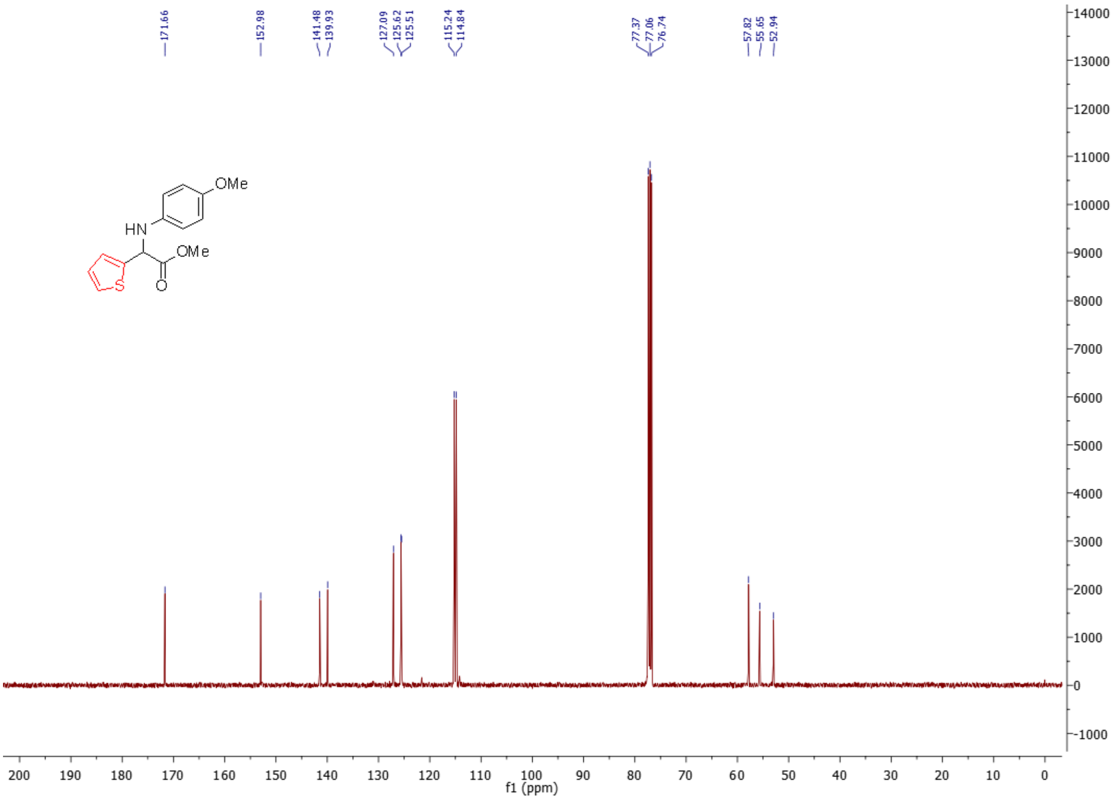

^{1}H NMR of Compound **39** ($CDCl_3$, 400 MHz):

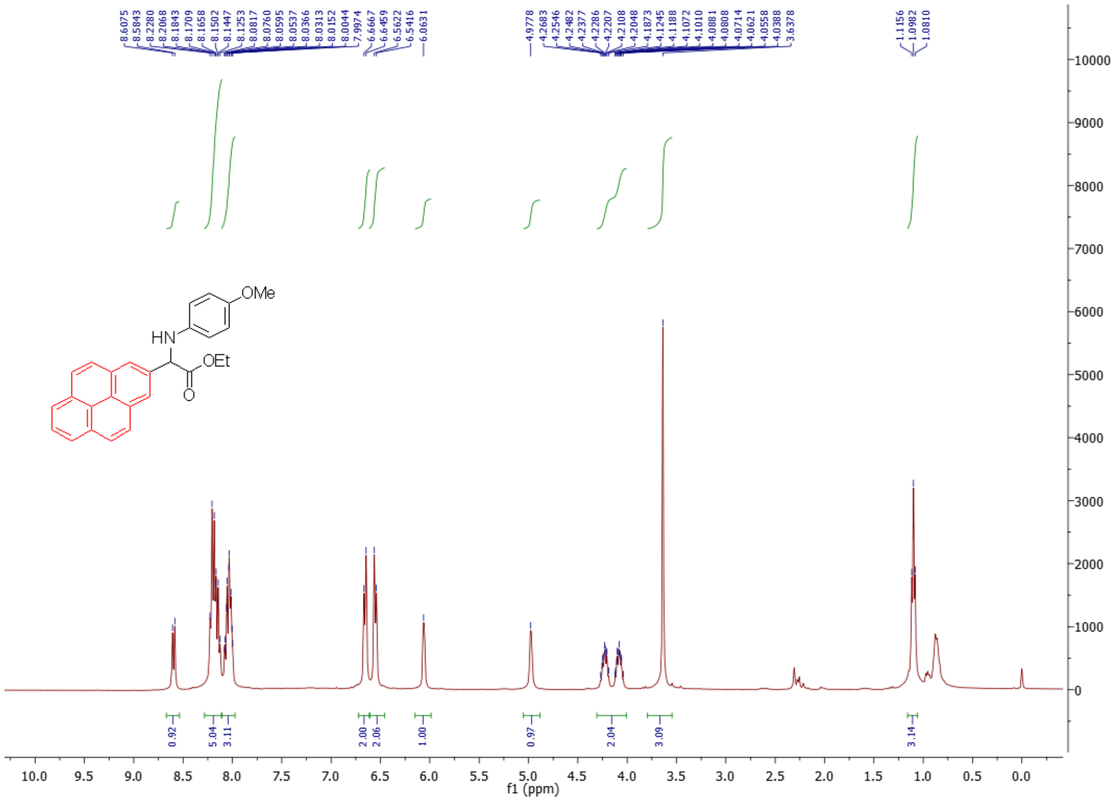

^{13}C NMR of Compound **39** ($CDCl_3$, 100 MHz):

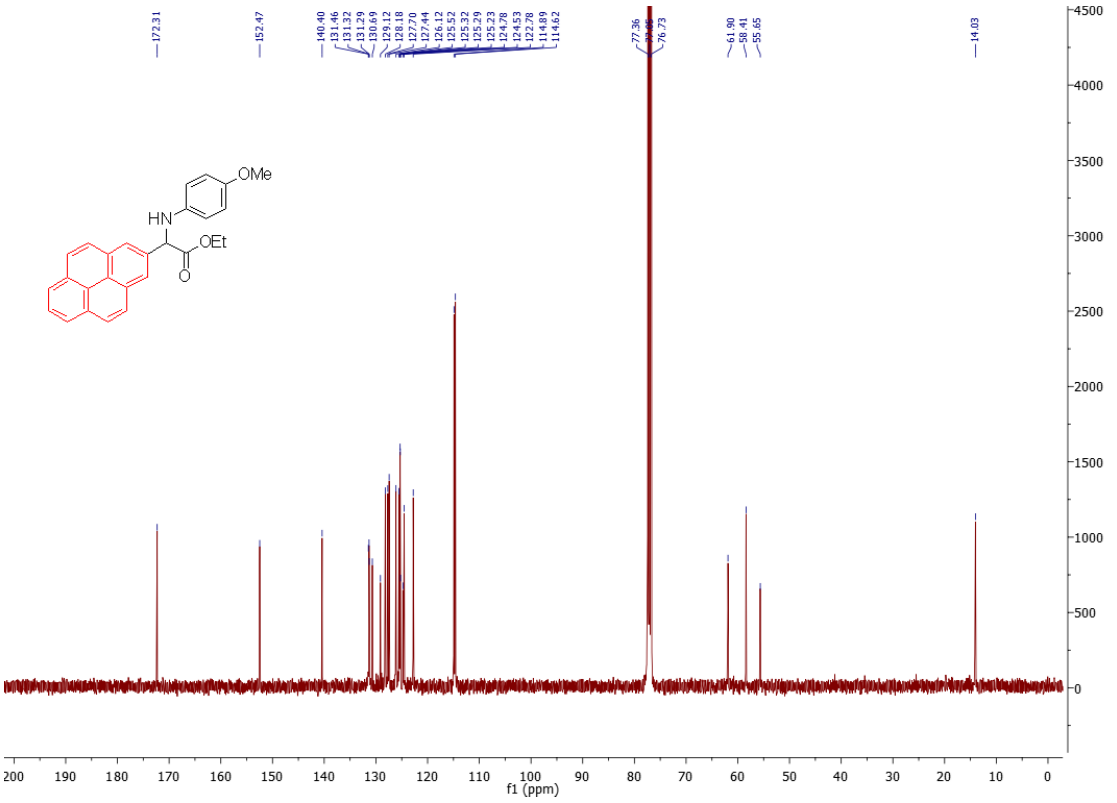

^{1}H NMR of Compound **40** ($CDCl_3$, 400 MHz):

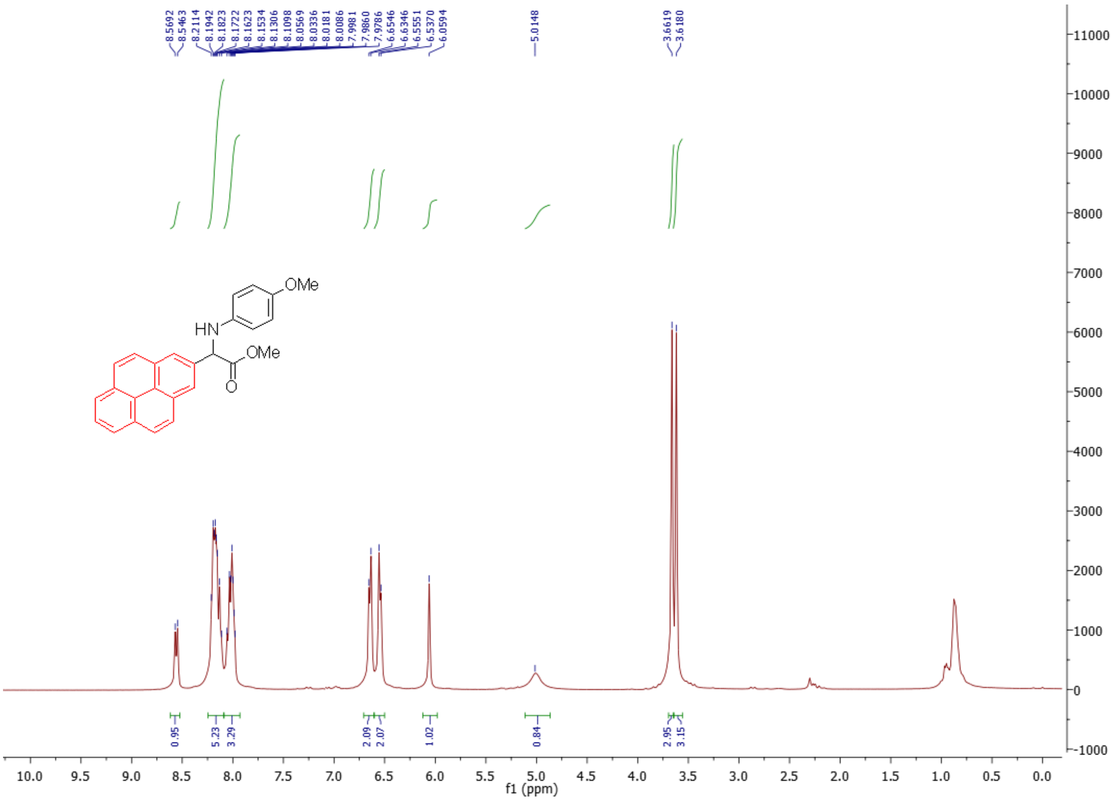

^{13}C NMR of Compound **40** ($CDCl_3$, 100 MHz):

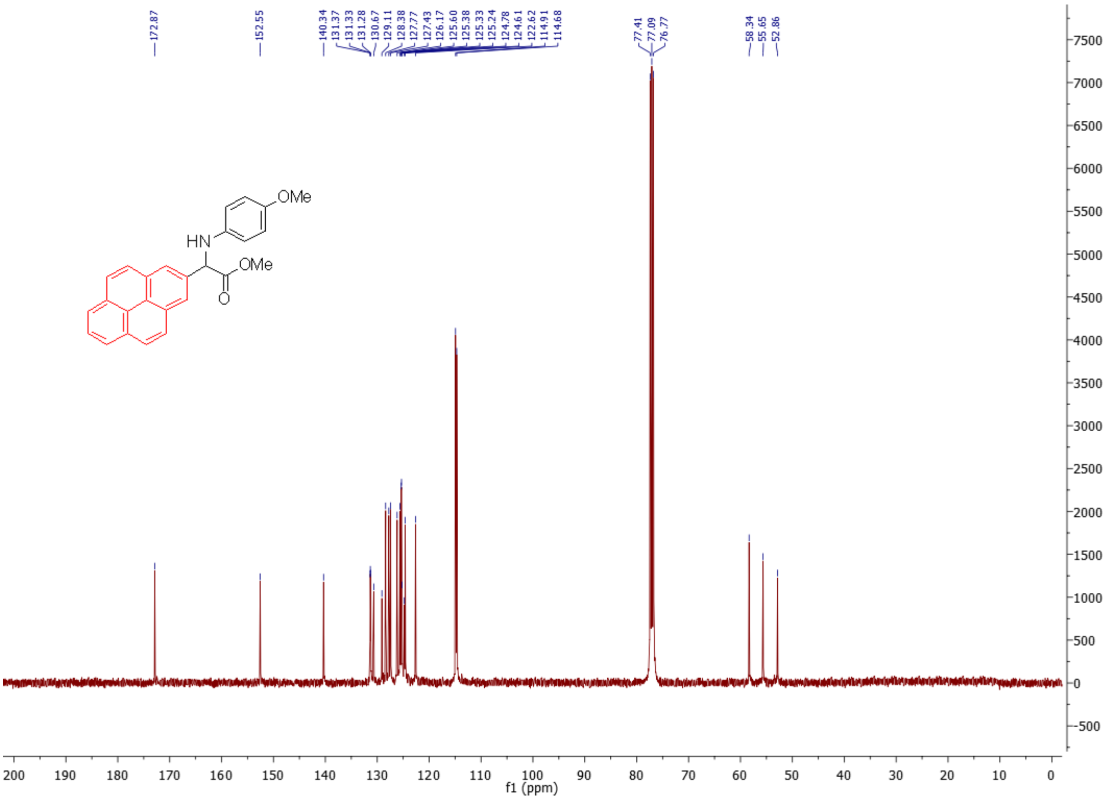

^{1}H NMR of Compound **41** ($CDCl_3$, 400 MHz):

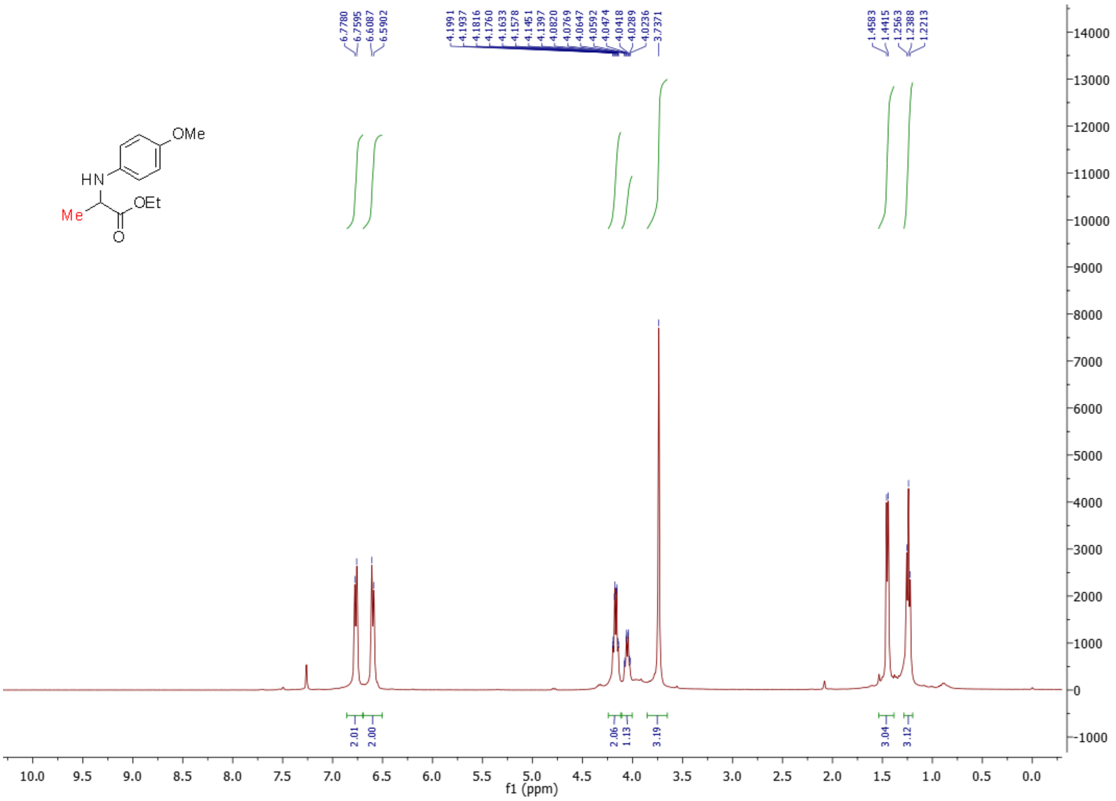

^{13}C NMR of Compound **41** ($CDCl_3$, 100 MHz):

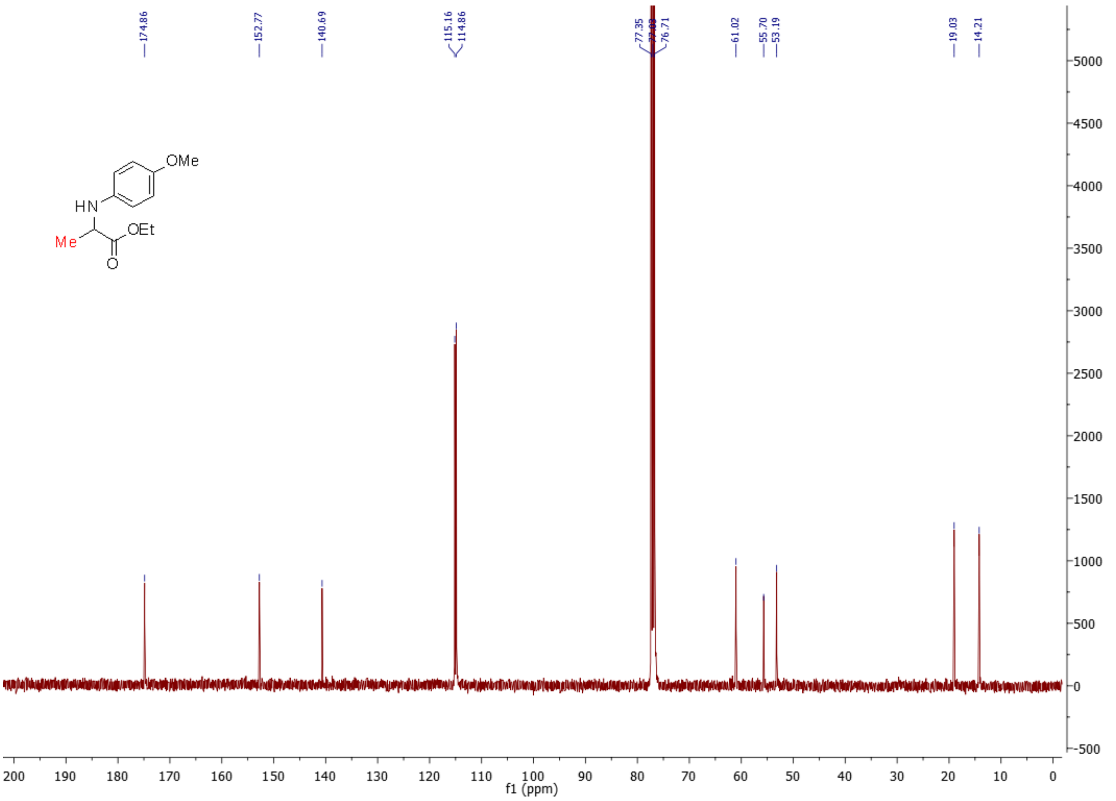

^{1}H NMR of Compound **42** ($CDCl_3$, 400 MHz):

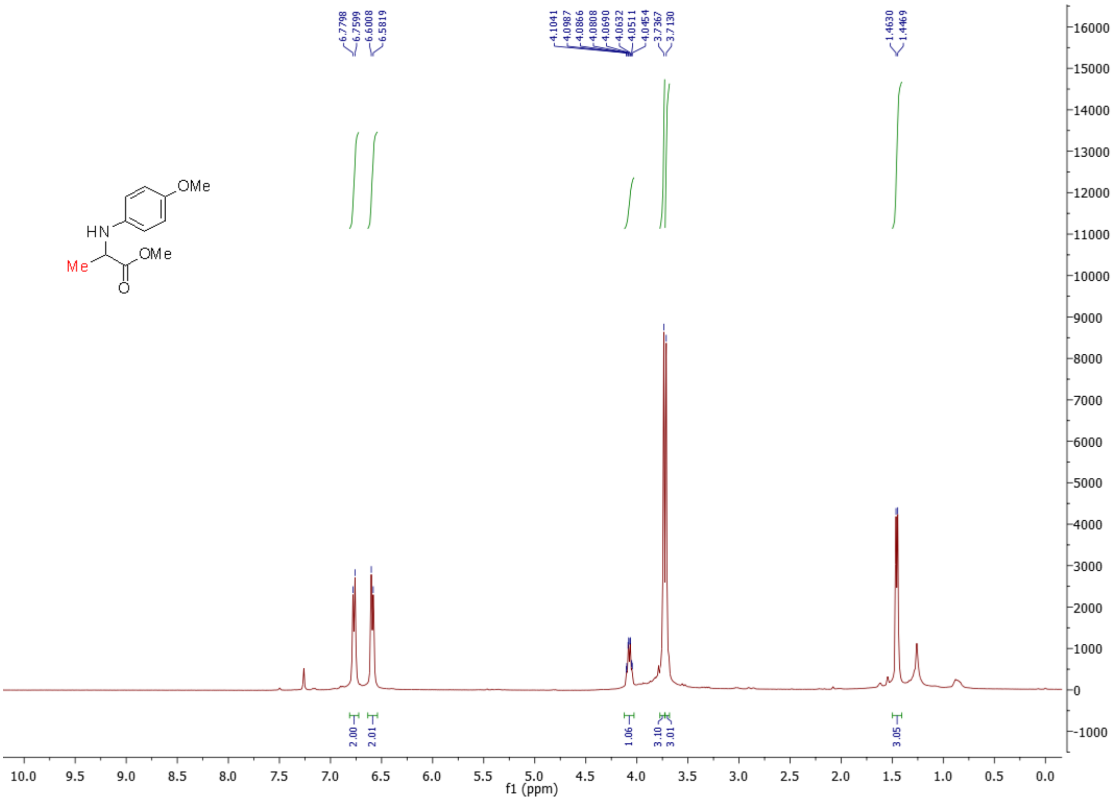

^{13}C NMR of Compound **42** ($CDCl_3$, 100 MHz):

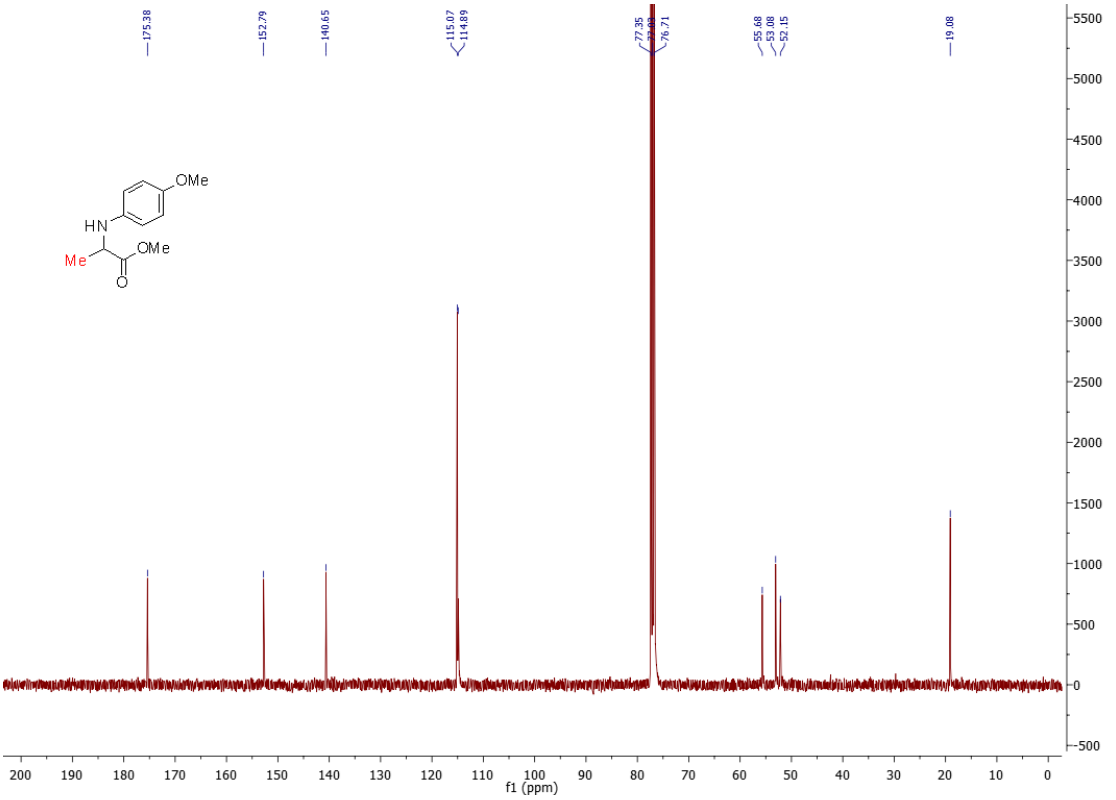

^{1}H NMR of Compound **43** ($CDCl_3$, 400 MHz):

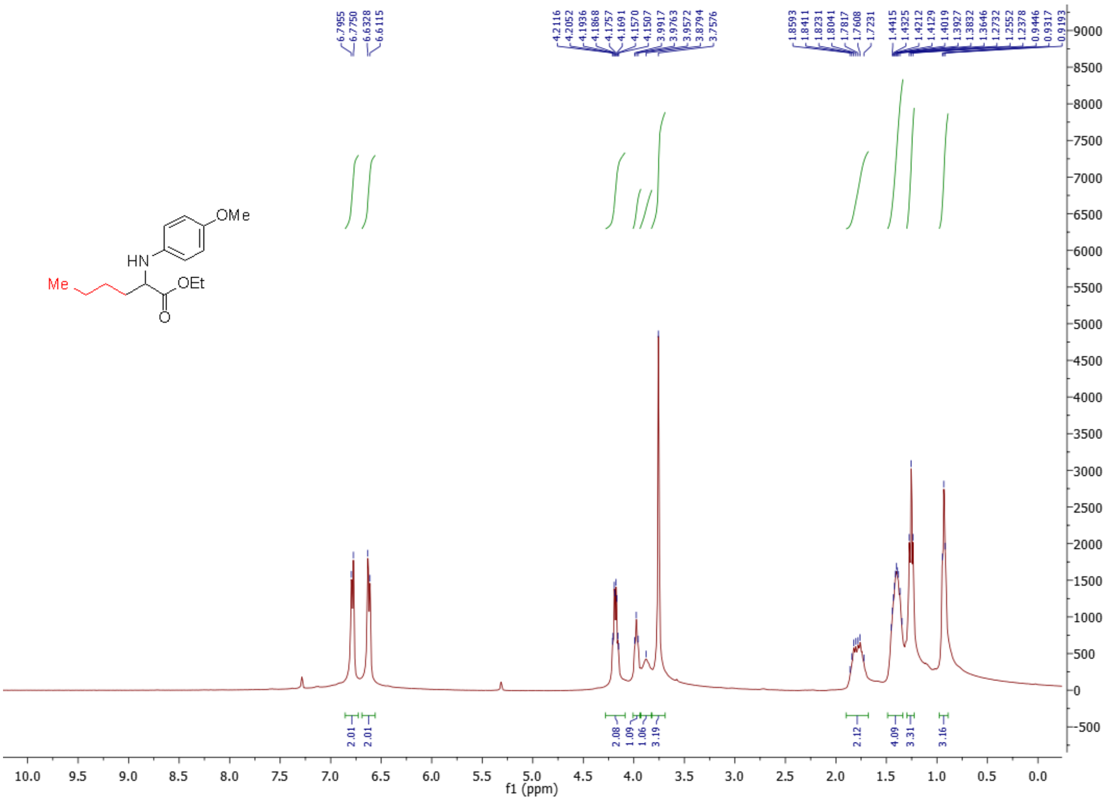

^{13}C NMR of Compound **43** ($CDCl_3$, 100 MHz):

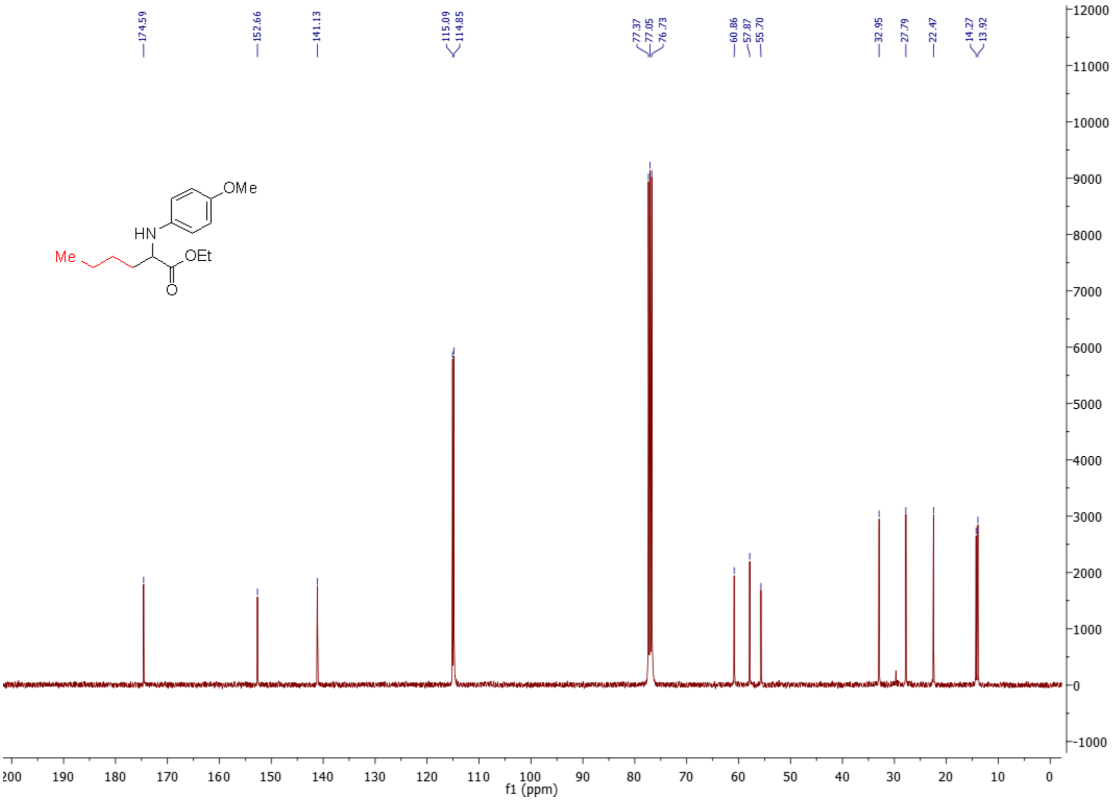

^{1}H NMR of Compound **44** ($CDCl_3$, 400 MHz):

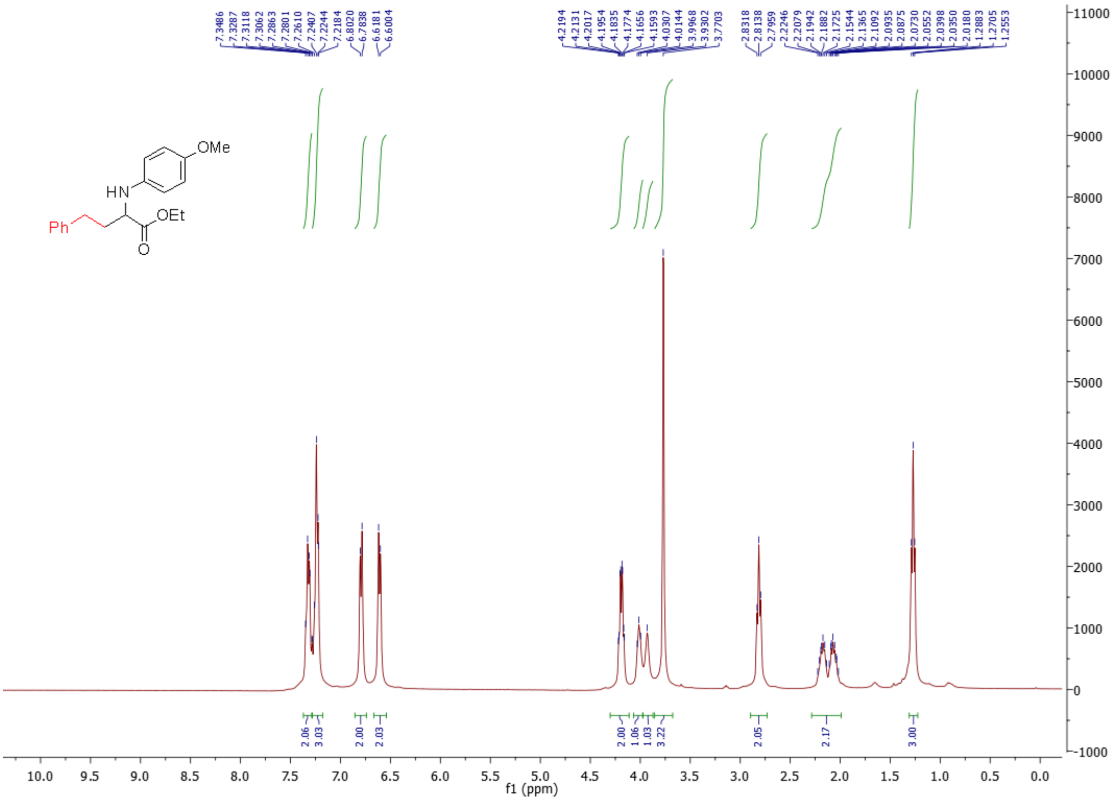

^{13}C NMR of Compound **44** ($CDCl_3$, 100 MHz):

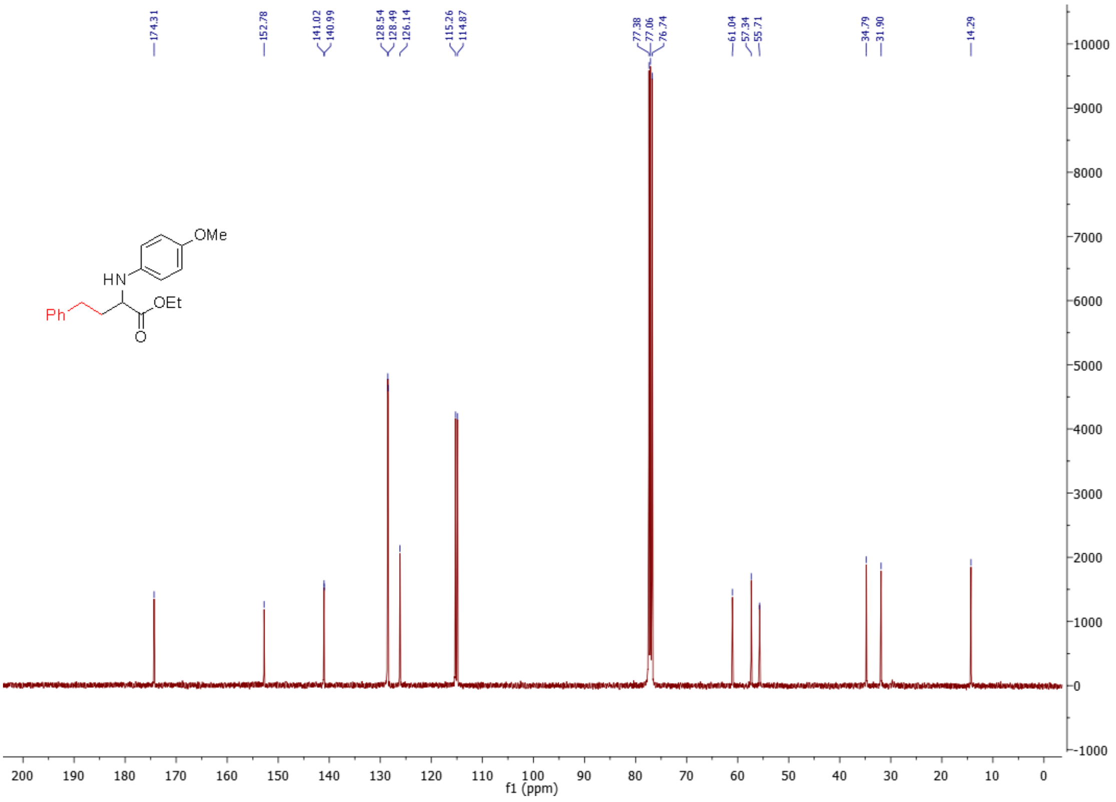

^{1}H NMR of Compound **45** ($CDCl_3$, 400 MHz):

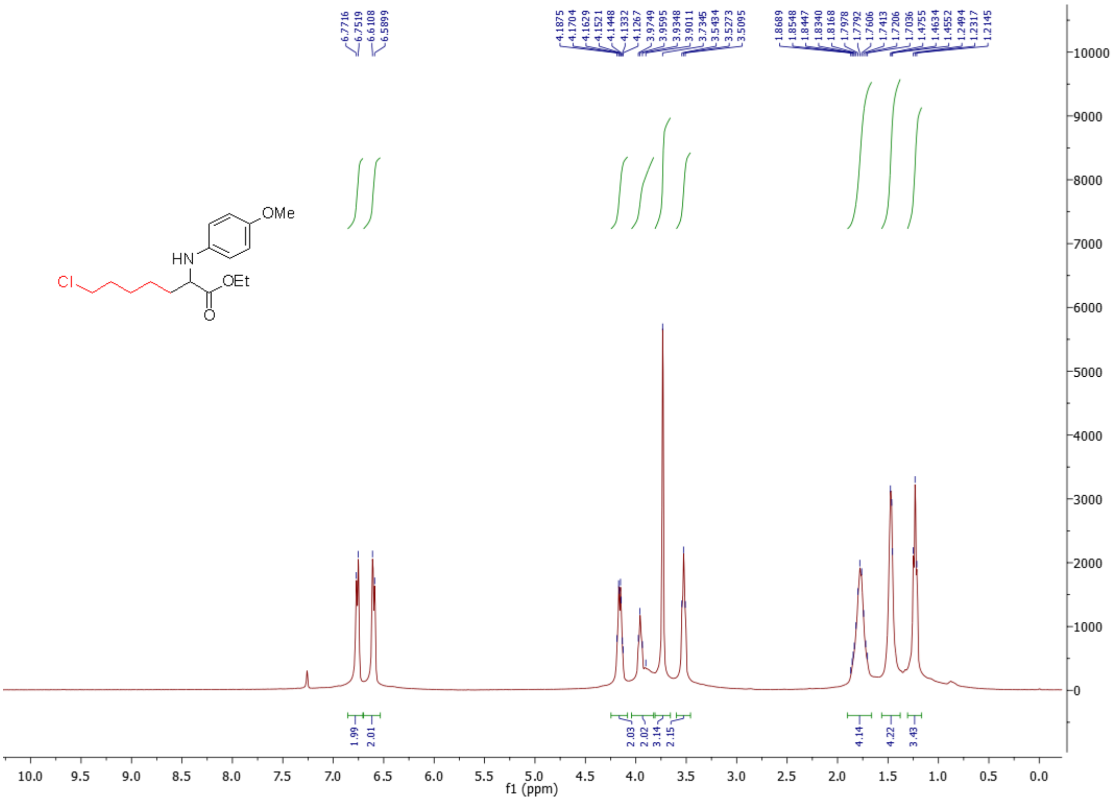

^{13}C NMR of Compound **45** ($CDCl_3$, 100 MHz):

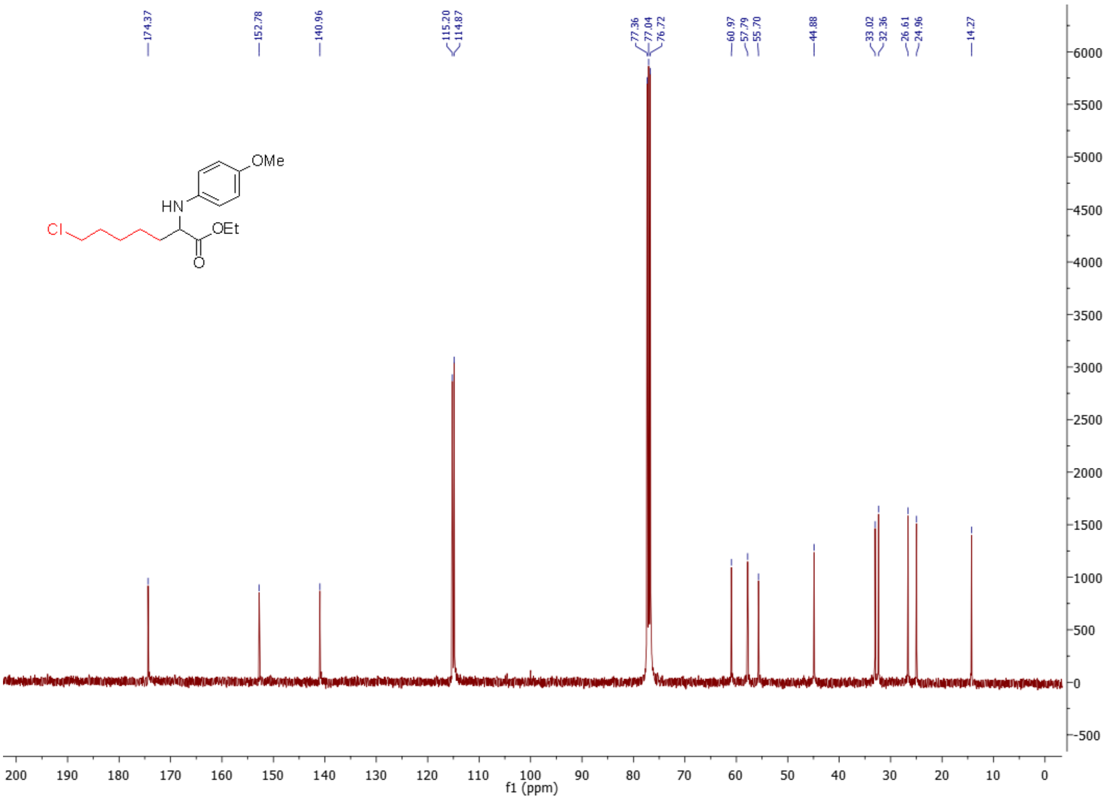

^{1}H NMR of Compound **46** ($CDCl_3$, 400 MHz):

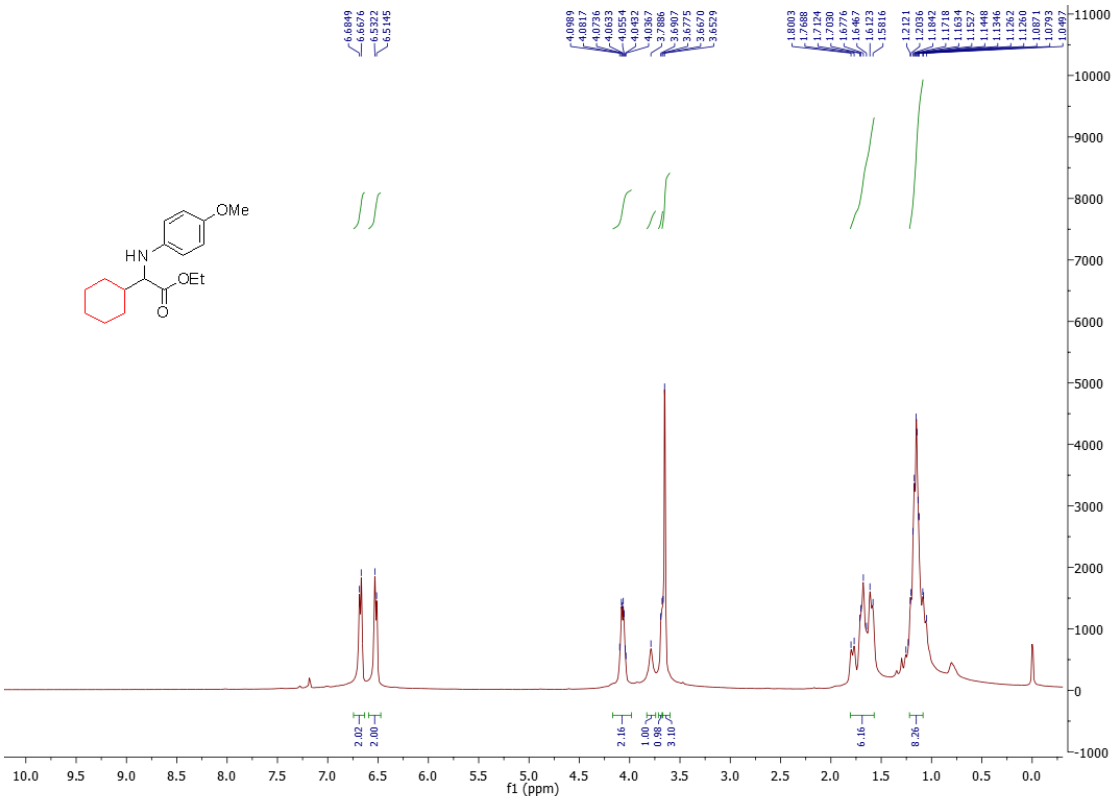

^{13}C NMR of Compound **46** ($CDCl_3$, 100 MHz):

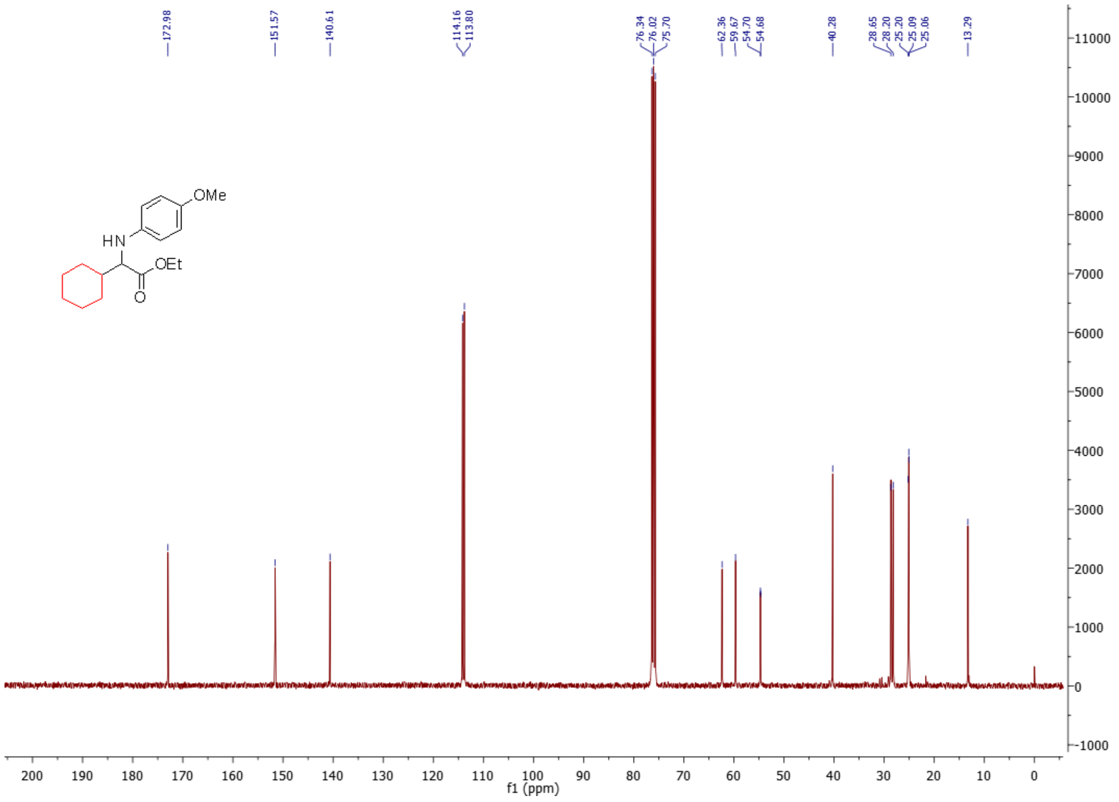

^{1}H NMR of Compound **47** ($CDCl_3$, 400 MHz):

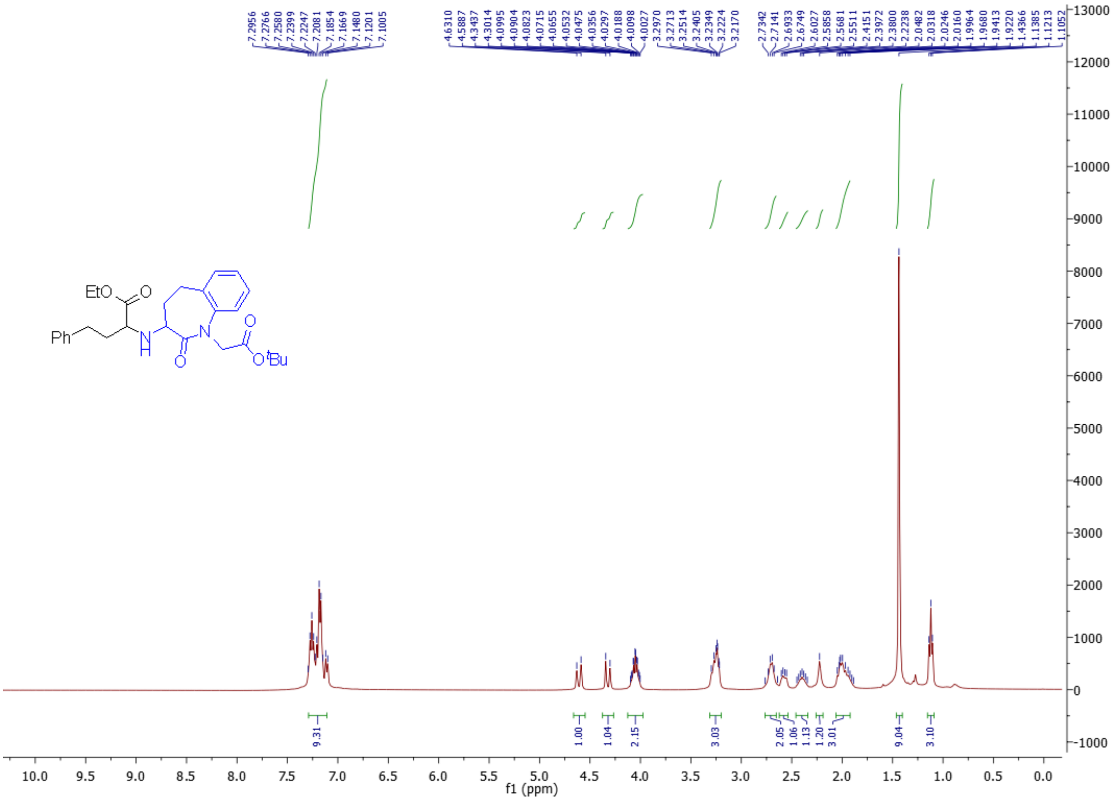

^{13}C NMR of Compound **47** ($CDCl_3$, 100 MHz):

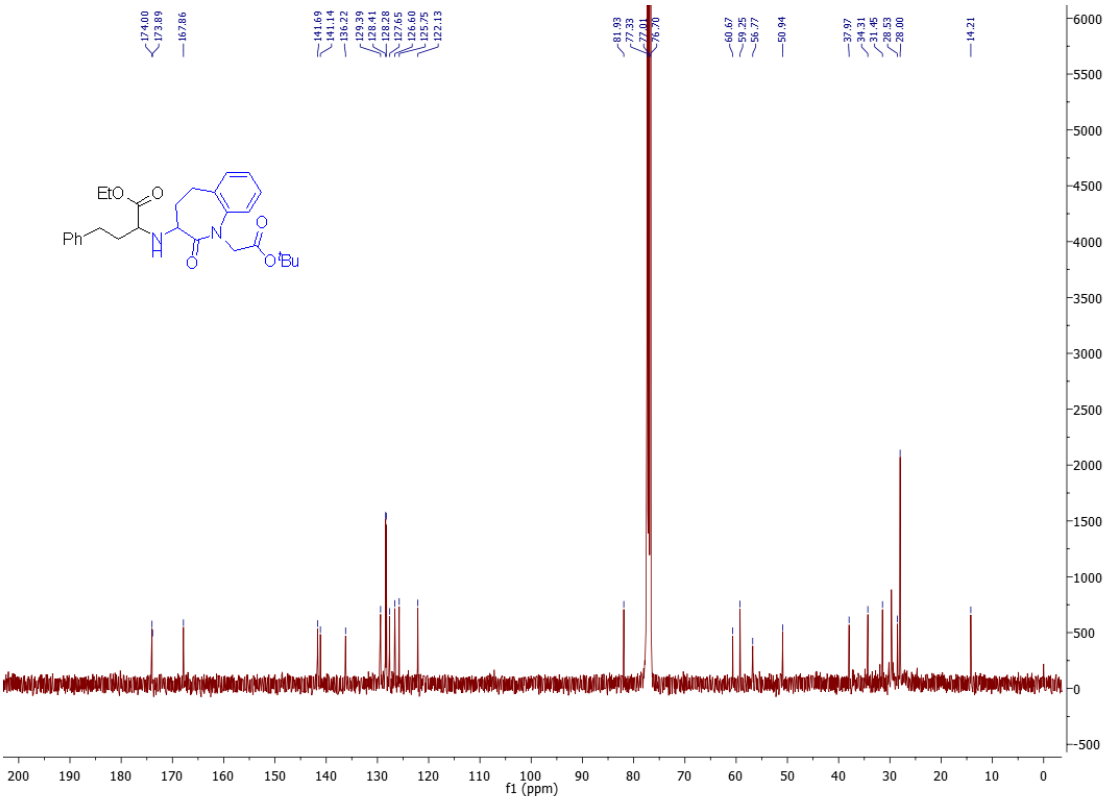

^{1}H NMR of Compound **48** ($CDCl_3$, 400 MHz):

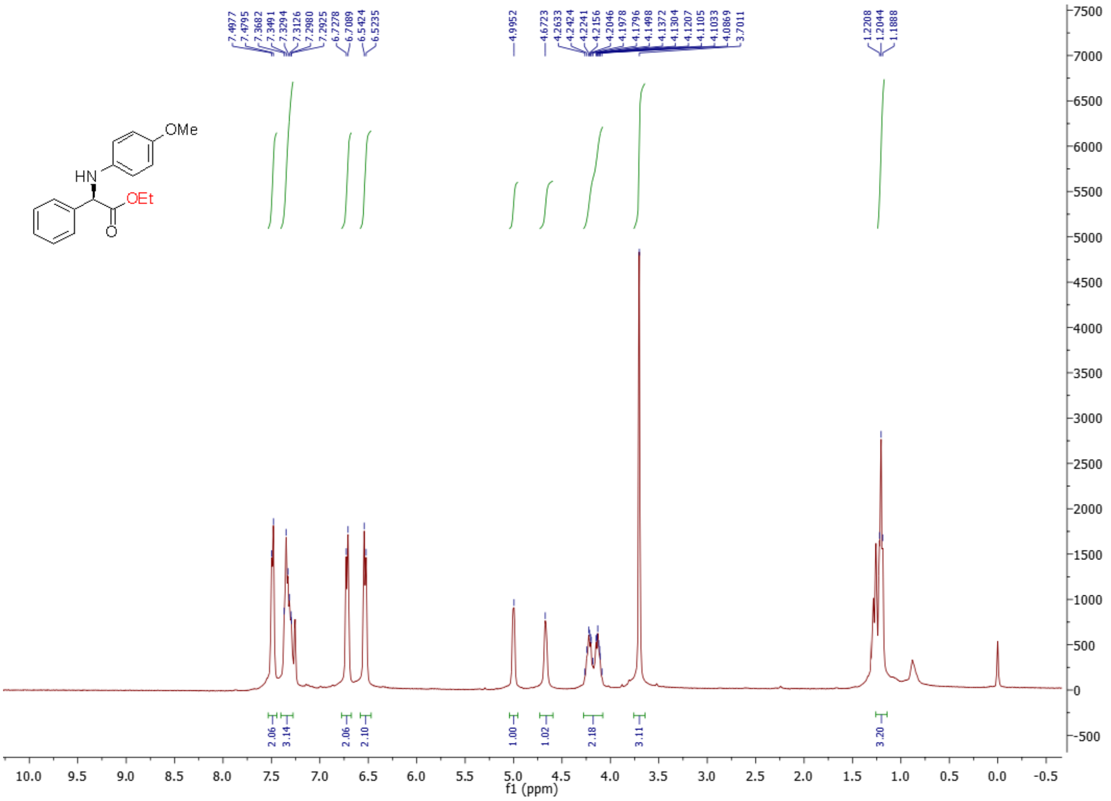

^{13}C NMR of Compound **48** ($CDCl_3$, 100 MHz):

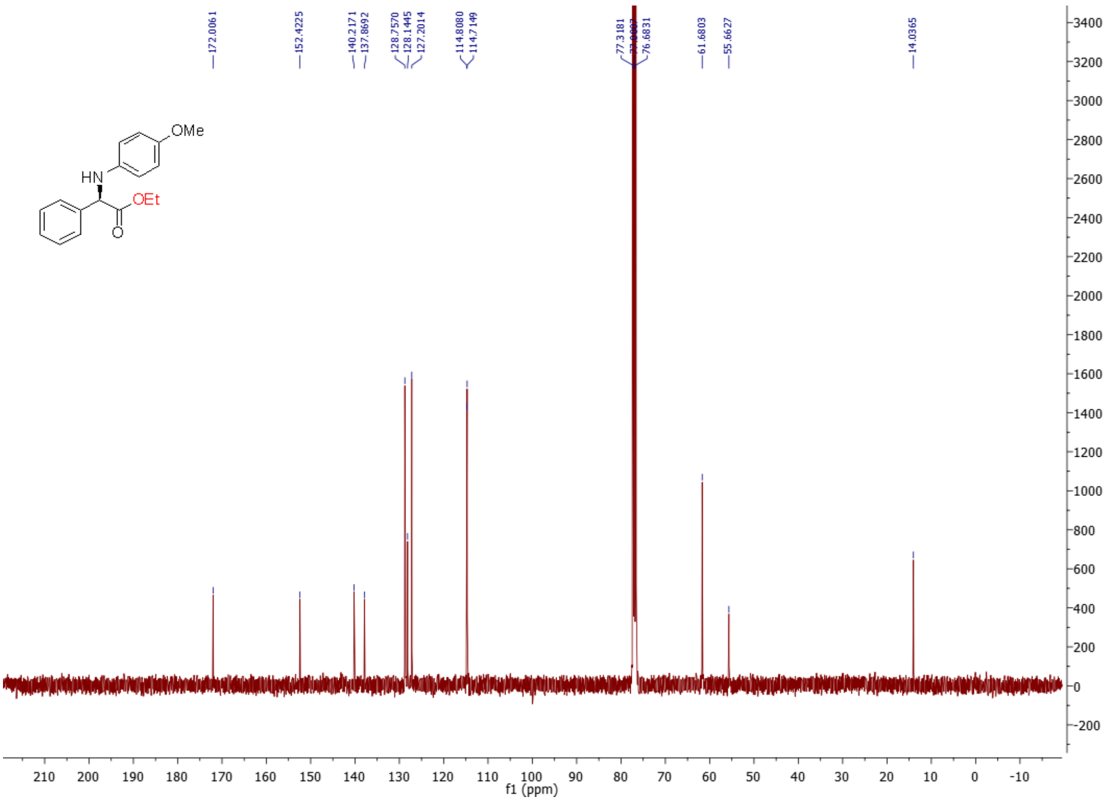

^{1}H NMR of Compound **49** ($CDCl_3$, 400 MHz):

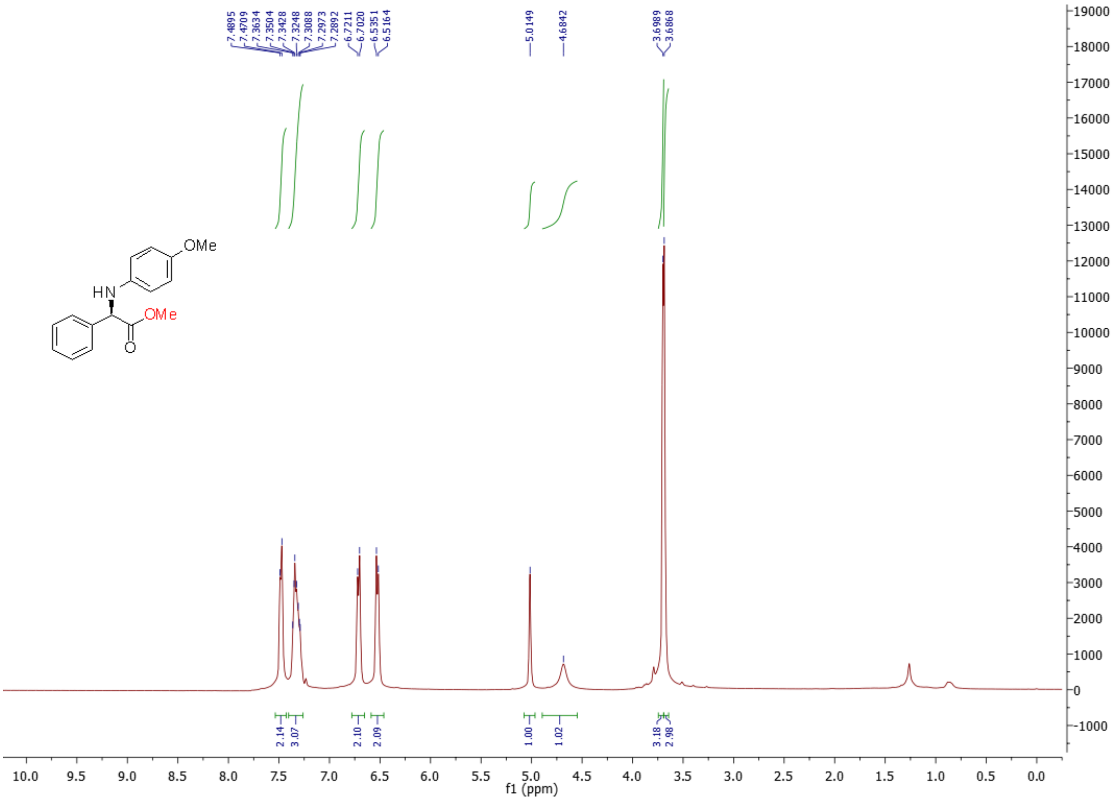

^{13}C NMR of Compound **49** ($CDCl_3$, 100 MHz):

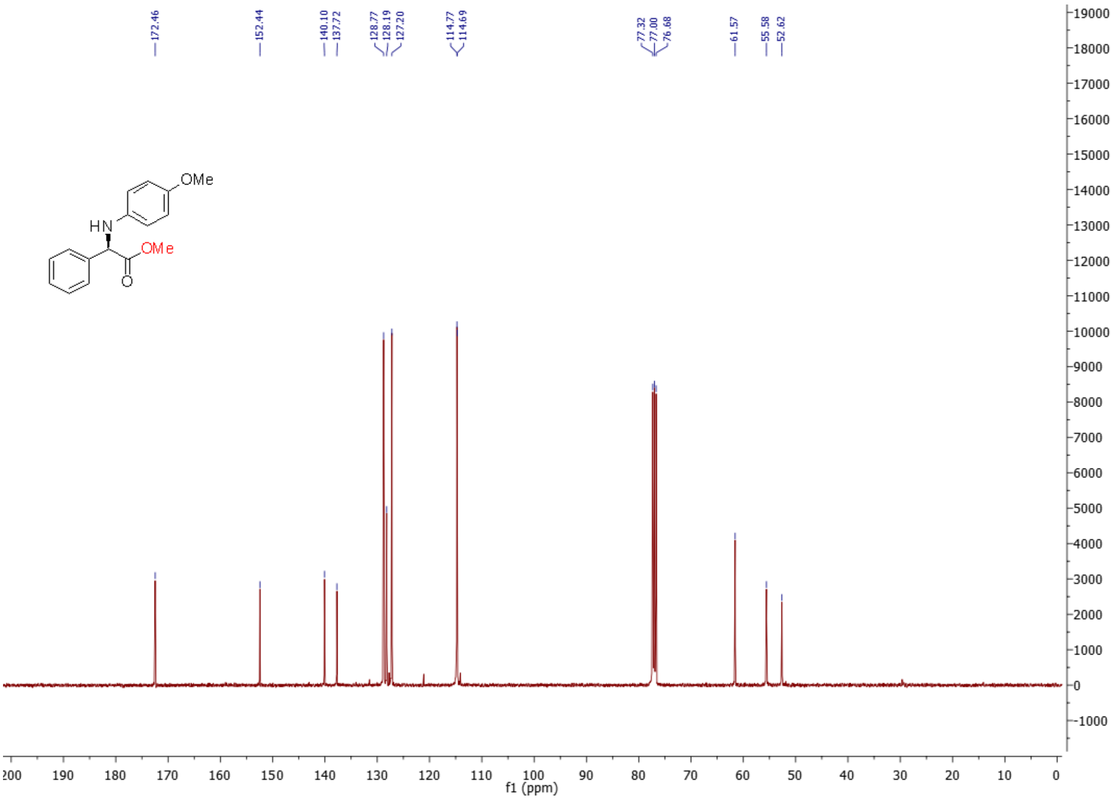

^{1}H NMR of Compound **50** ($CDCl_3$, 400 MHz):

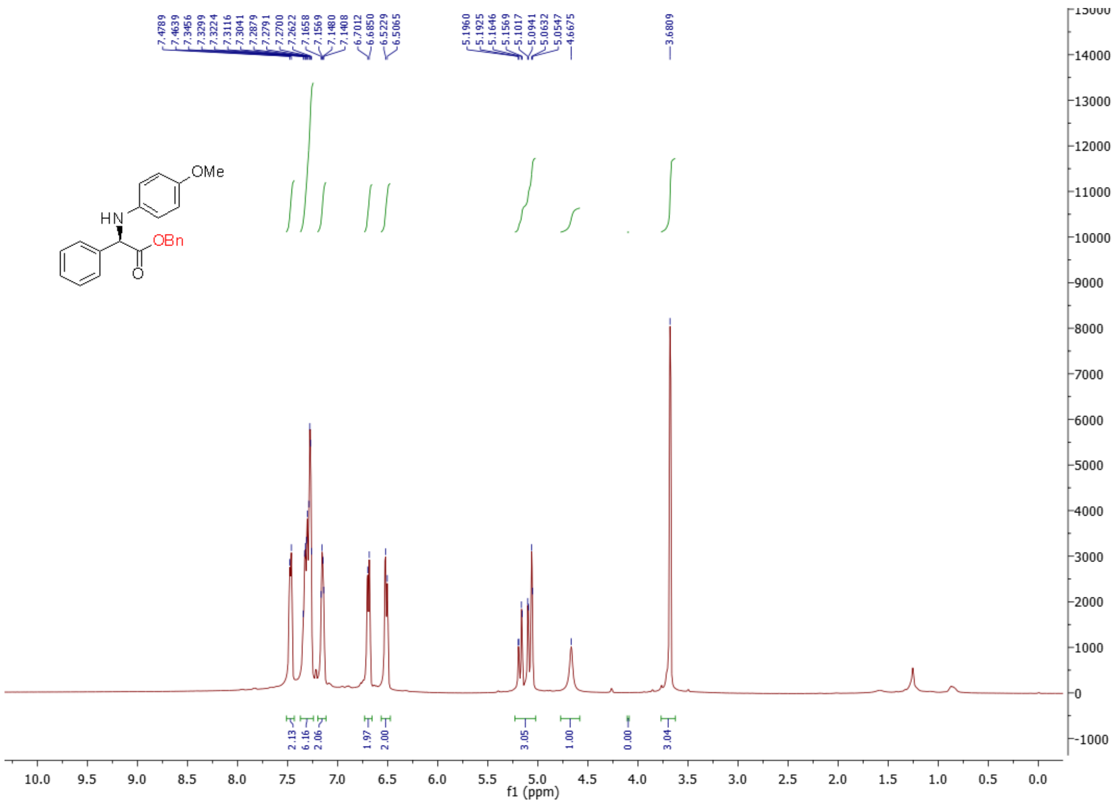

^{13}C NMR of Compound **50** ($CDCl_3$, 100 MHz):

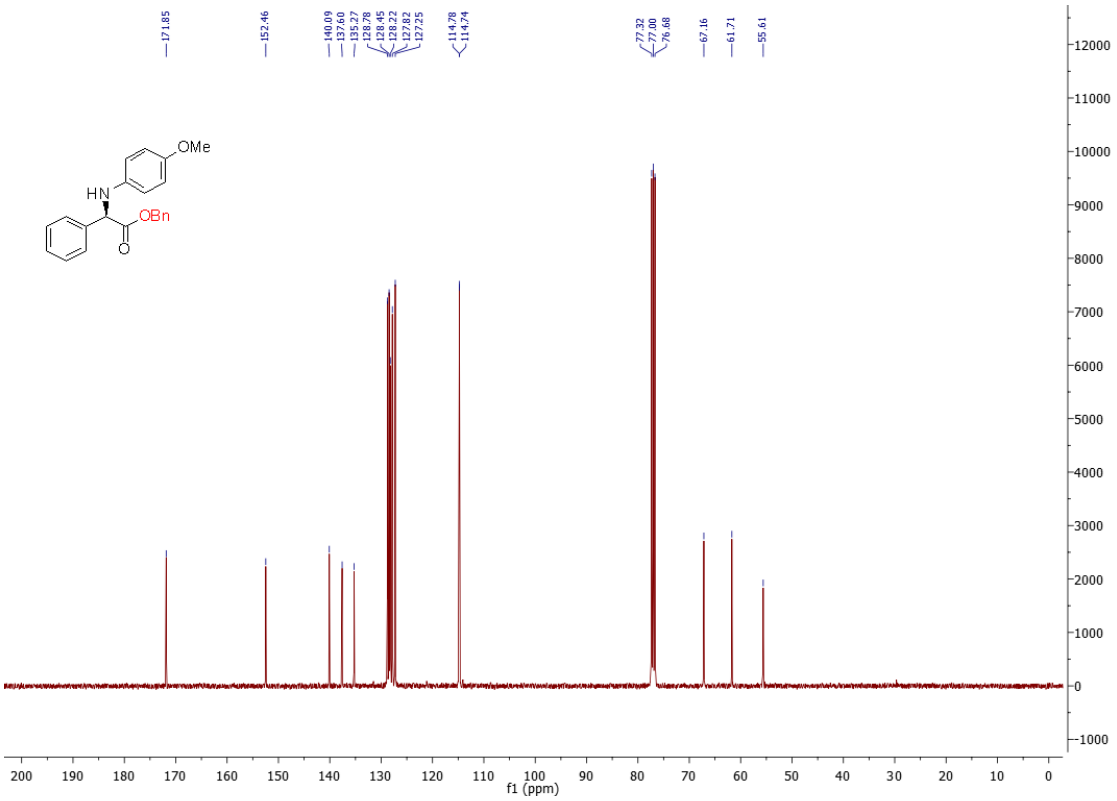

^{1}H NMR of Compound **51** ($CDCl_3$, 400 MHz):

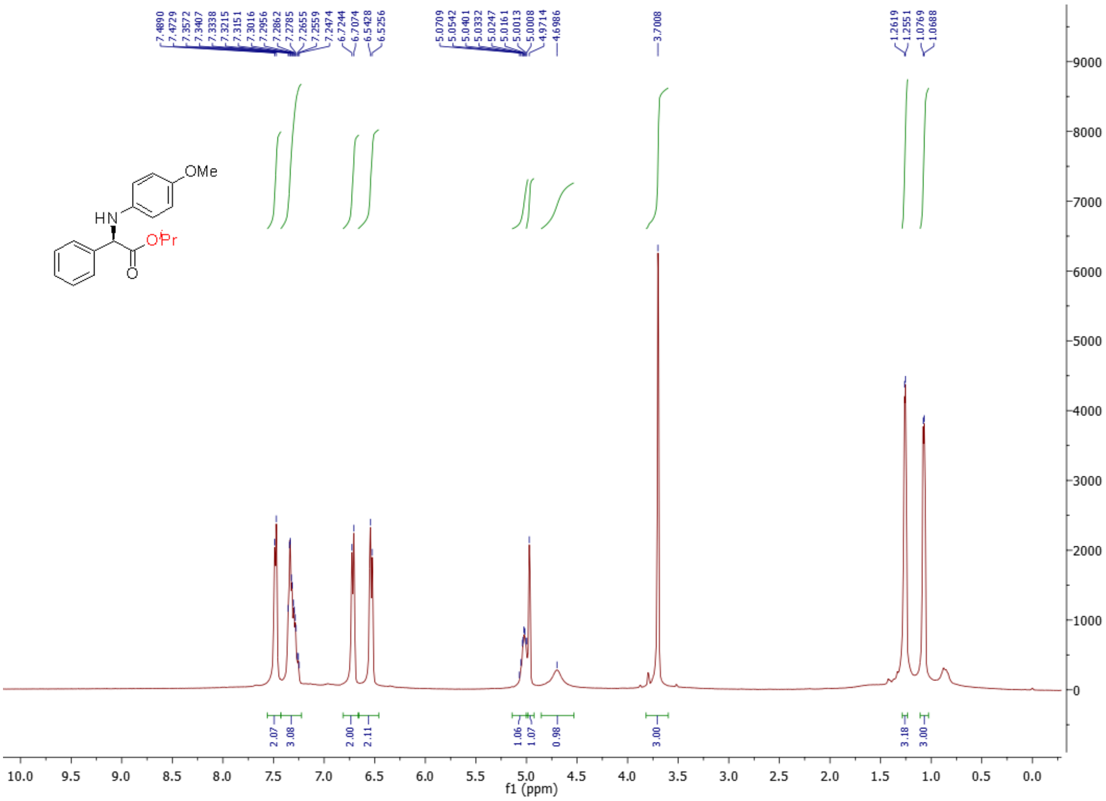

^{13}C NMR of Compound **51** ($CDCl_3$, 100 MHz):

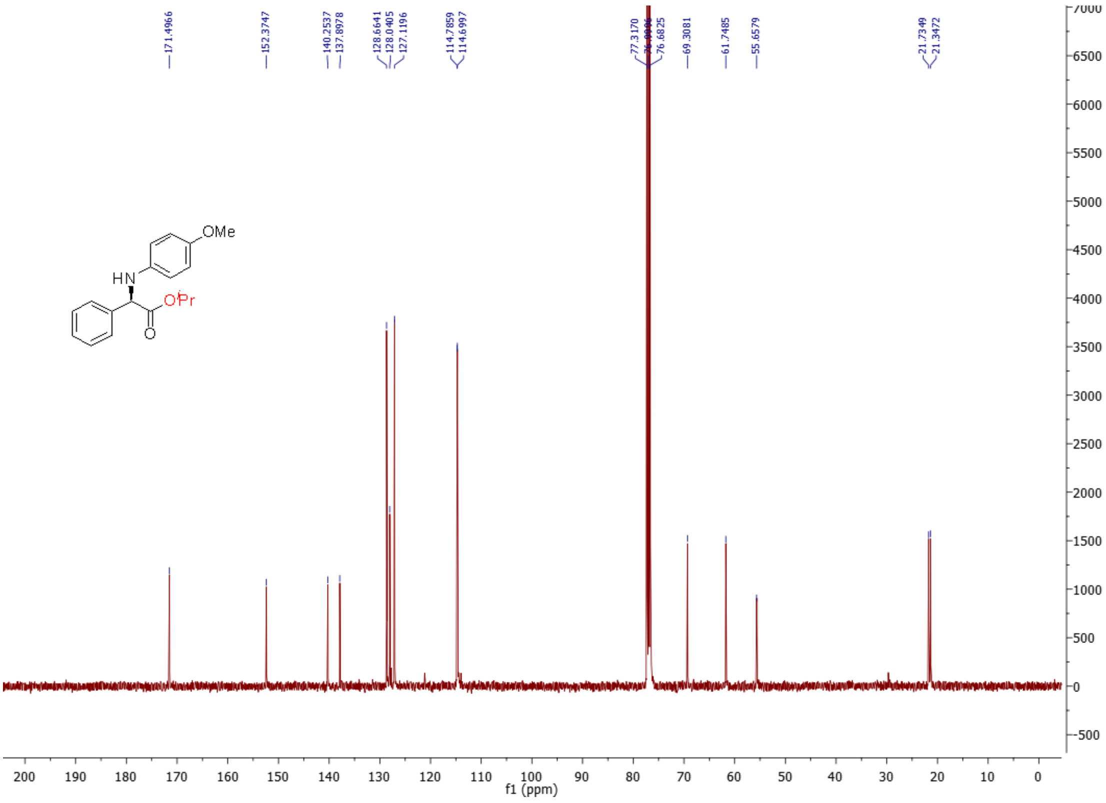

^{1}H NMR of Compound **52** ($CDCl_3$, 400 MHz):

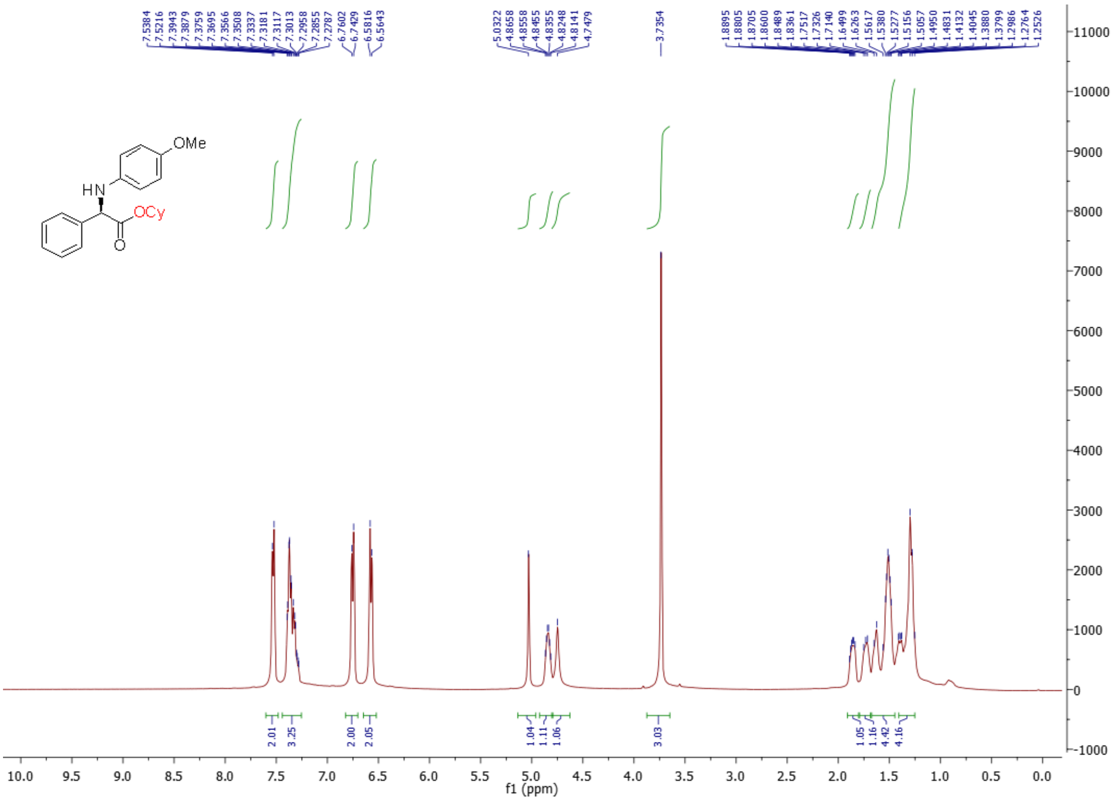

^{13}C NMR of Compound **52** ($CDCl_3$, 100 MHz):

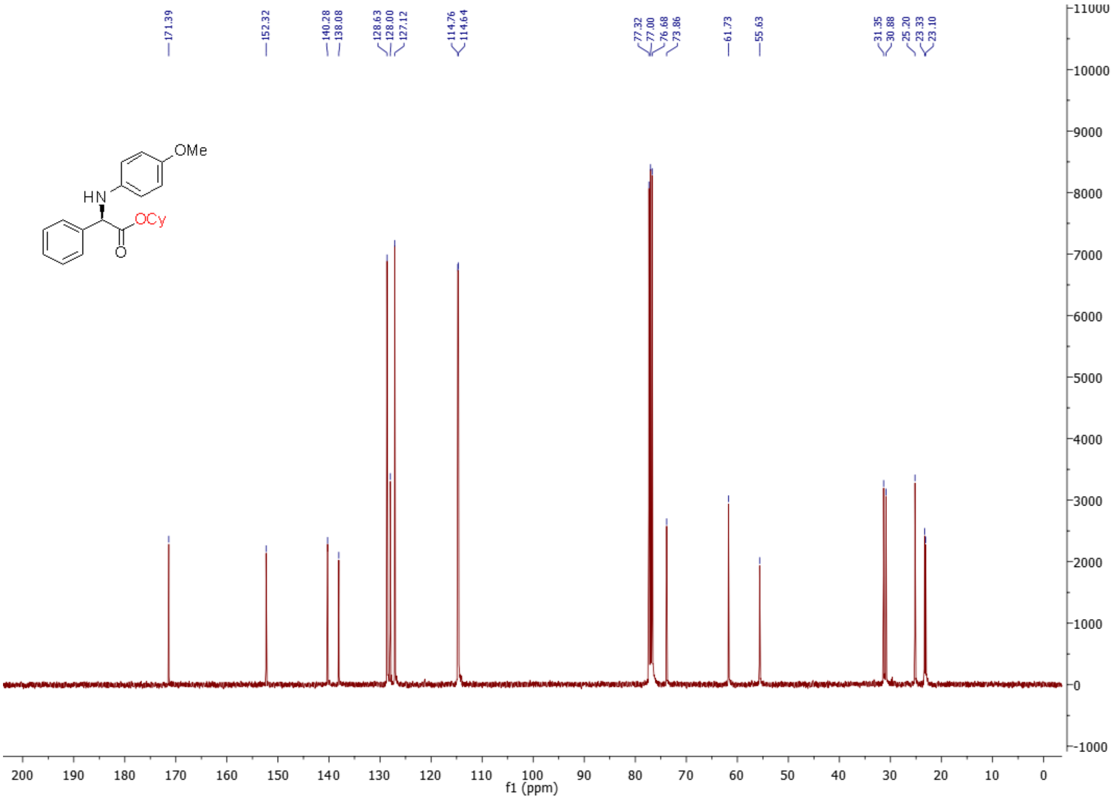

^{1}H NMR of Compound **53** ($CDCl_3$, 400 MHz):

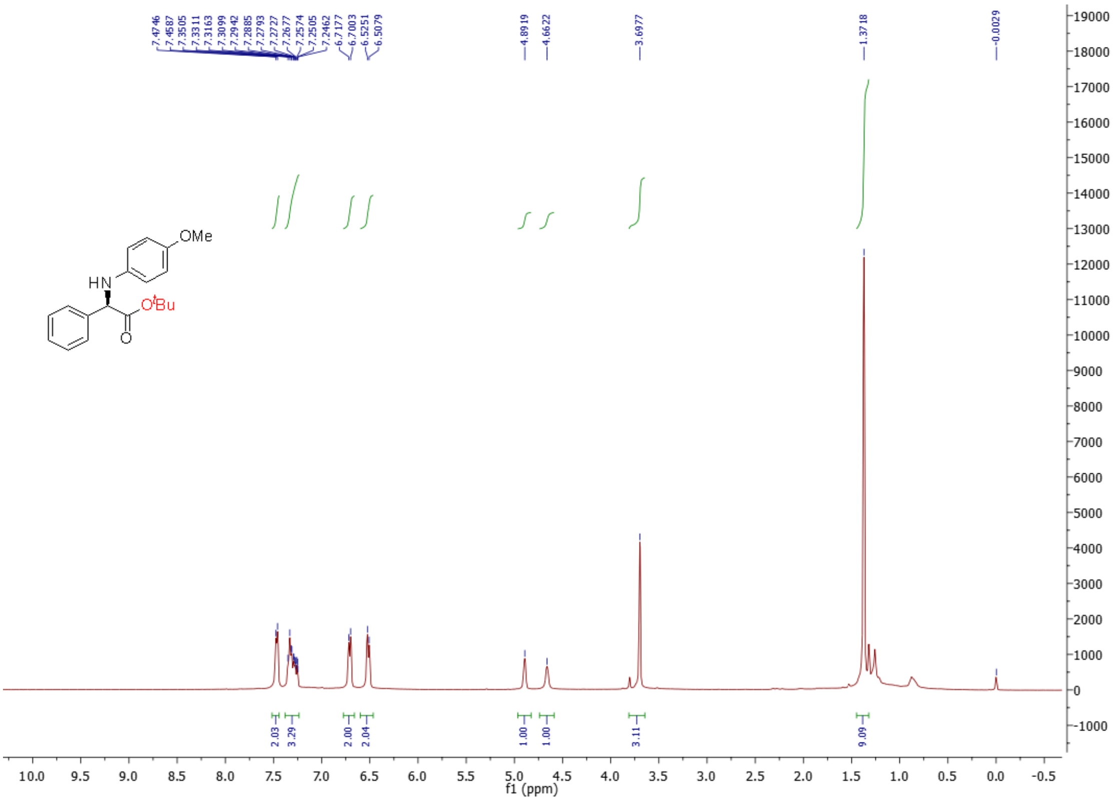

^{13}C NMR of Compound **53** ($CDCl_3$, 100 MHz):

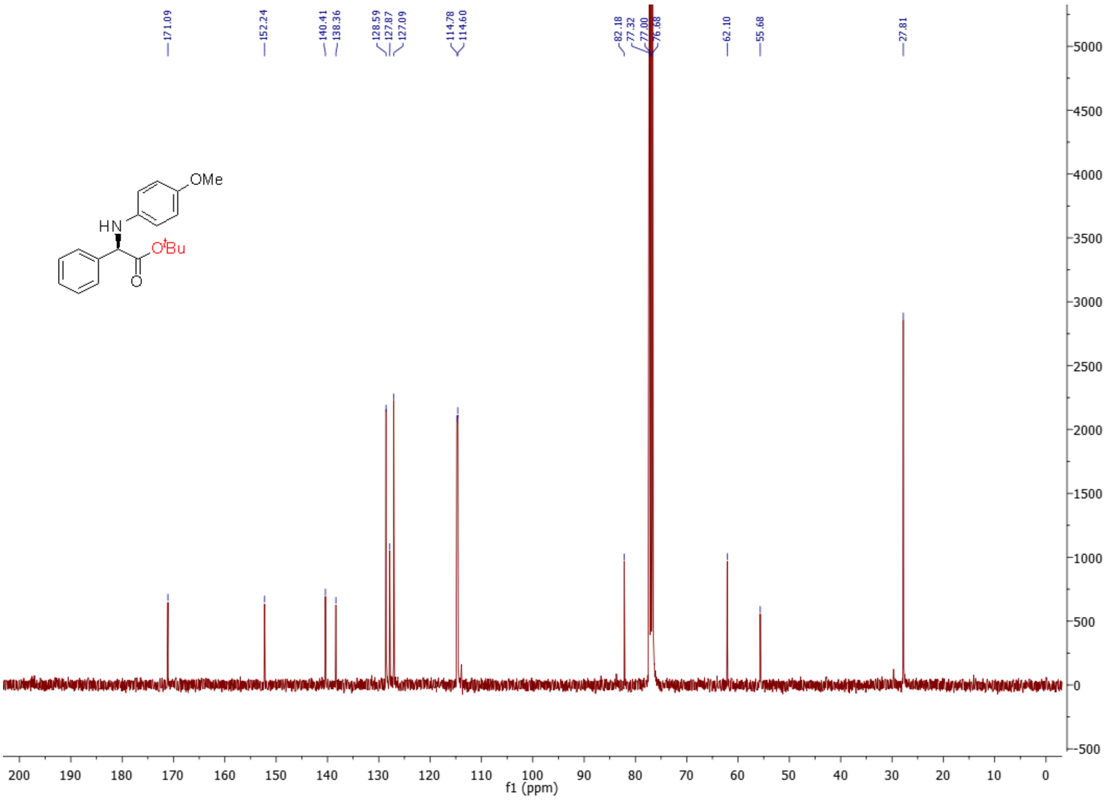

^{1}H NMR of Compound **54** ($CDCl_3$, 400 MHz):

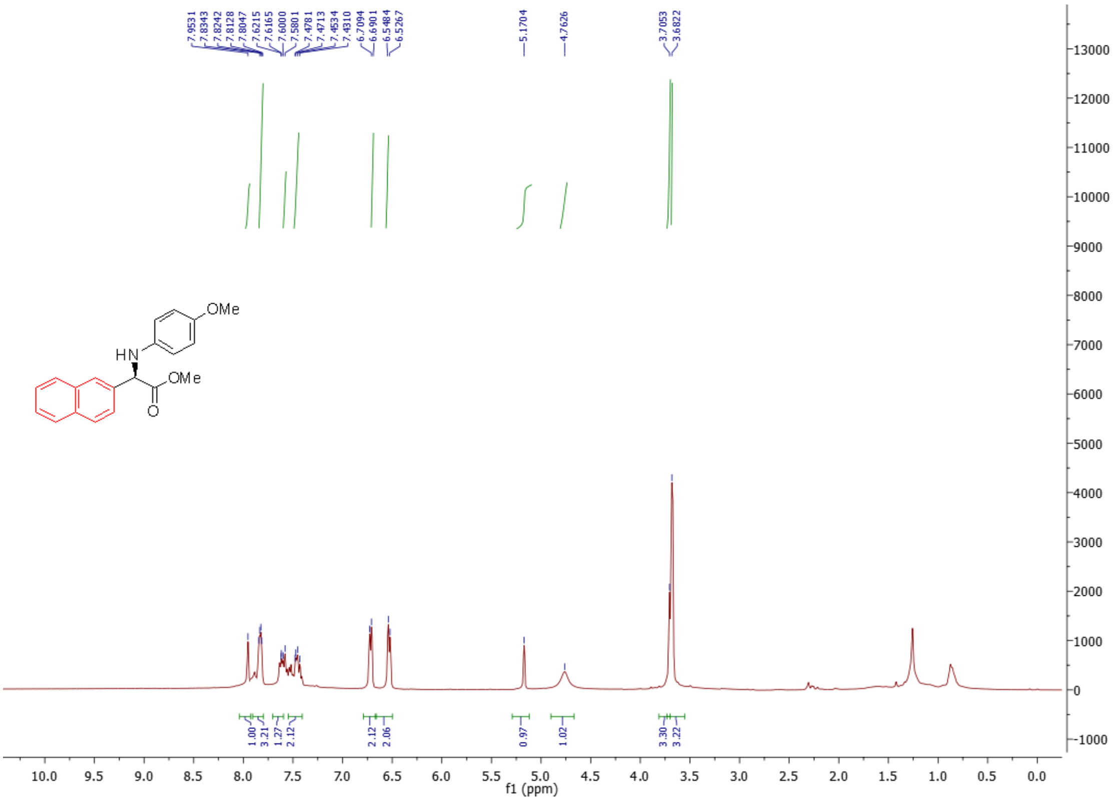

^{1}H NMR of Compound **54** ($CDCl_3$, 400 MHz):

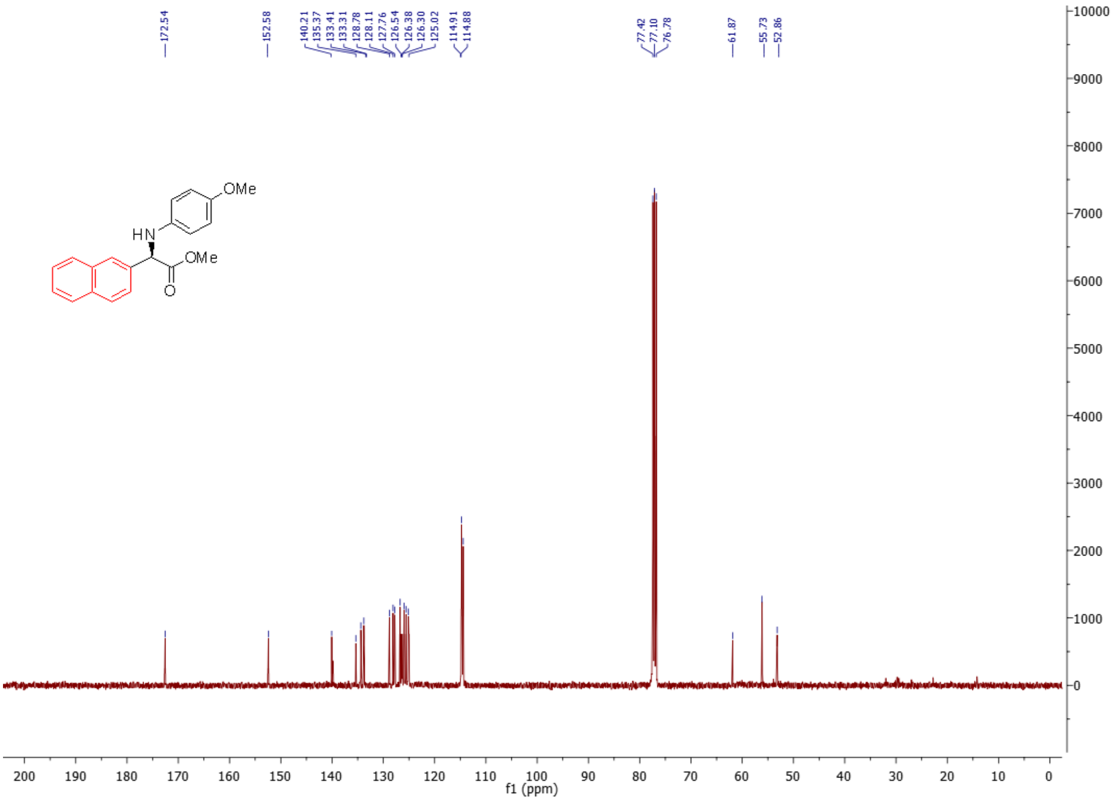

^{1}H NMR of Compound **55** ($CDCl_3$, 400 MHz):

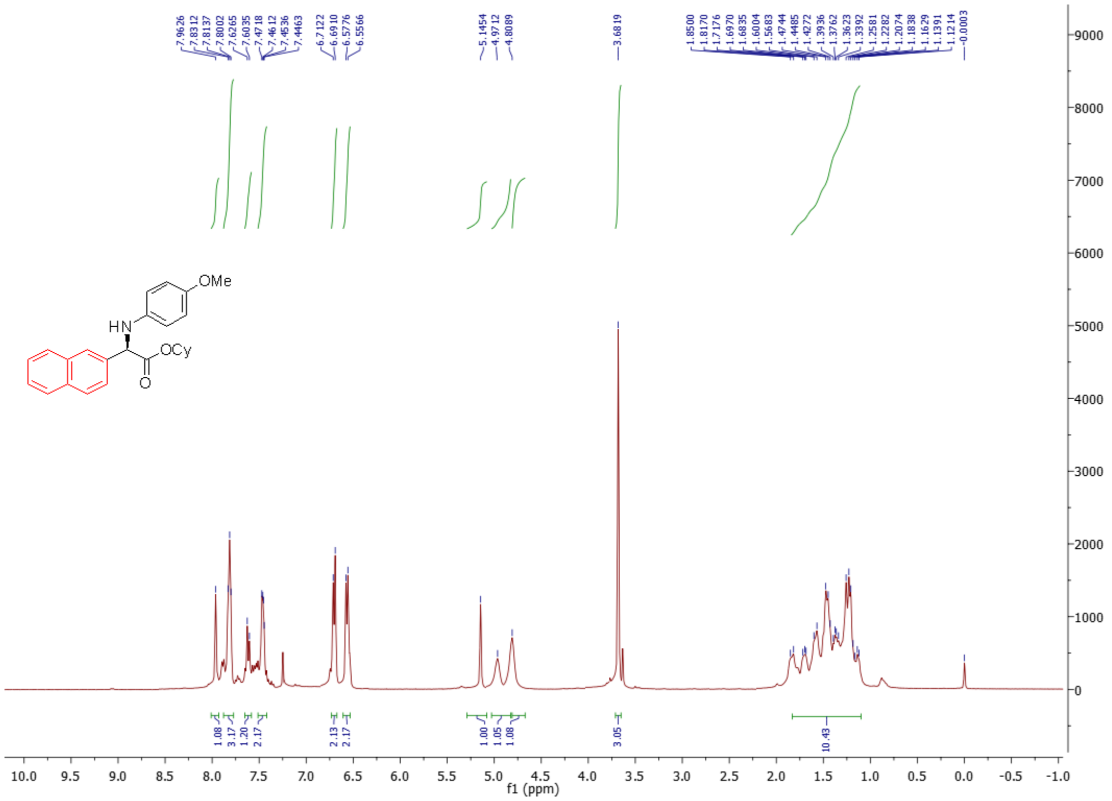

^{1}H NMR of Compound **55** ($CDCl_3$, 400 MHz):

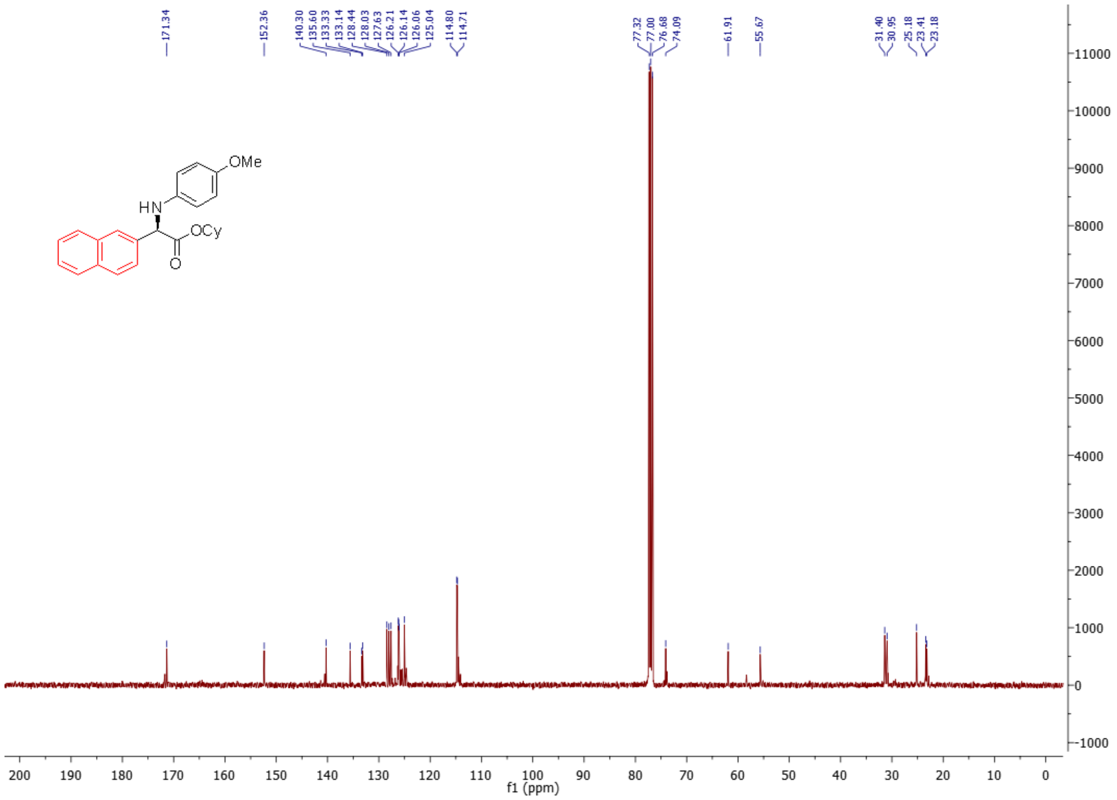

^{1}H NMR of Compound **56** ($CDCl_3$, 400 MHz):

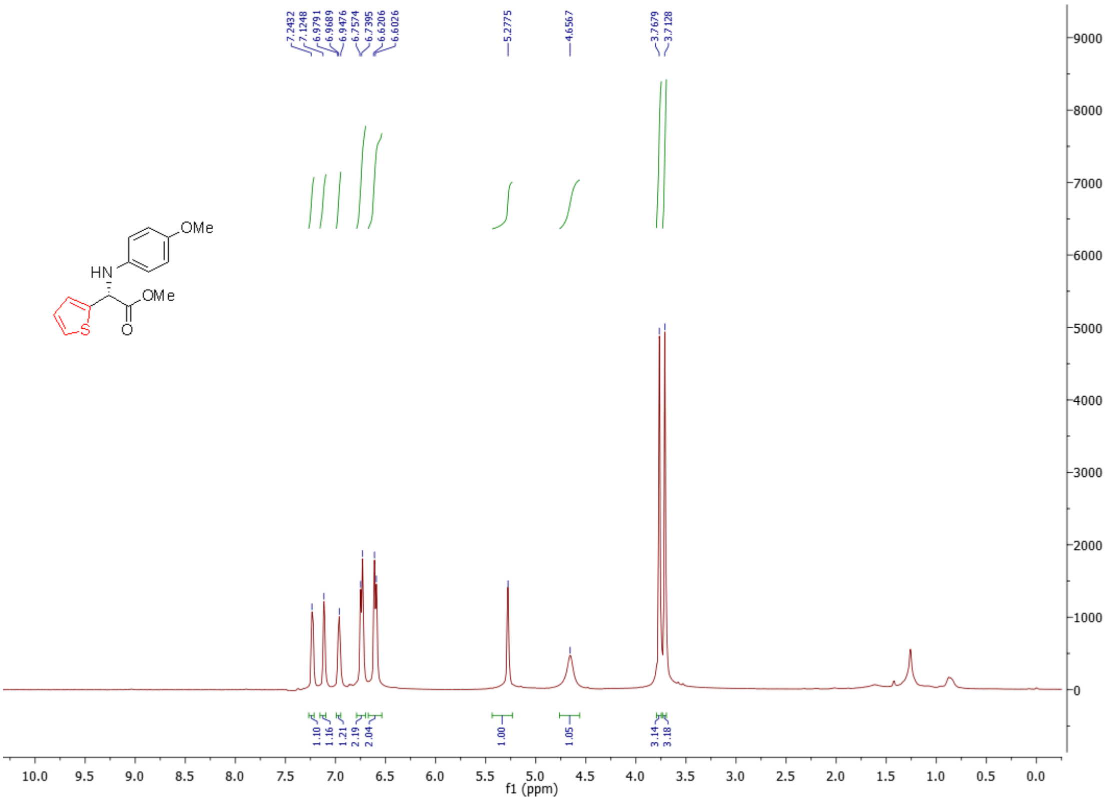

^{1}H NMR of Compound **56** ($CDCl_3$, 400 MHz):

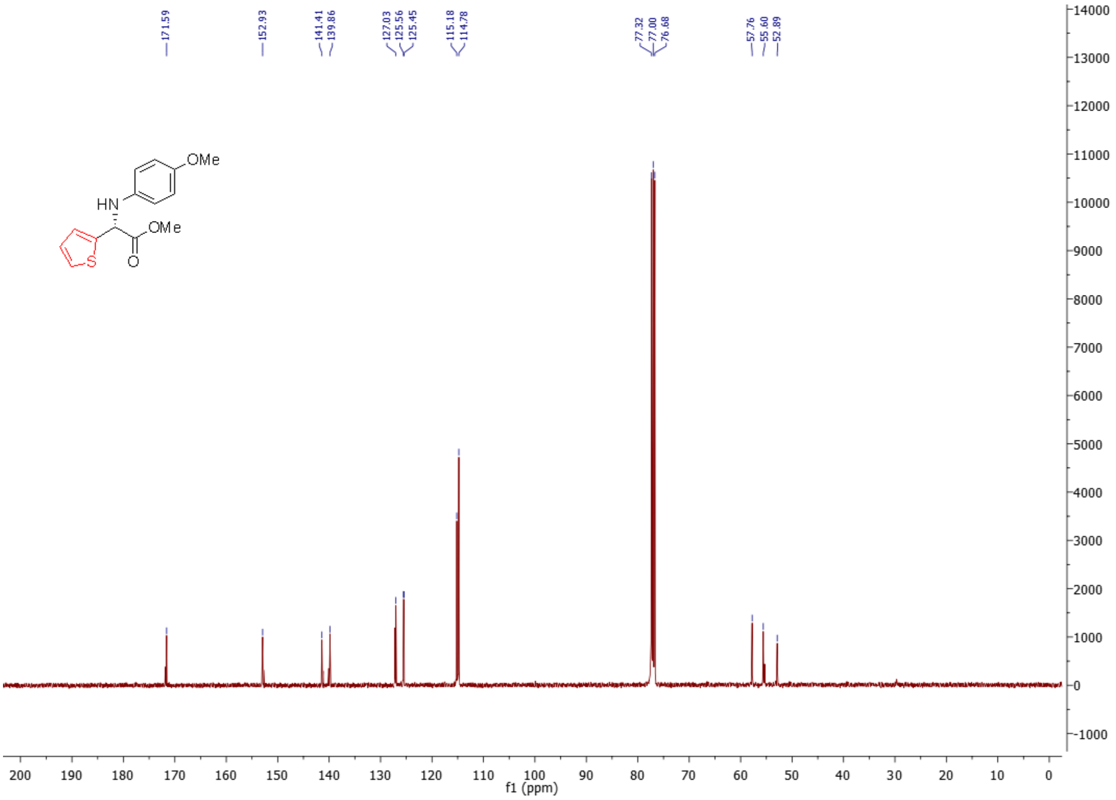

^{1}H NMR of Compound **57** ($CDCl_3$, 400 MHz):

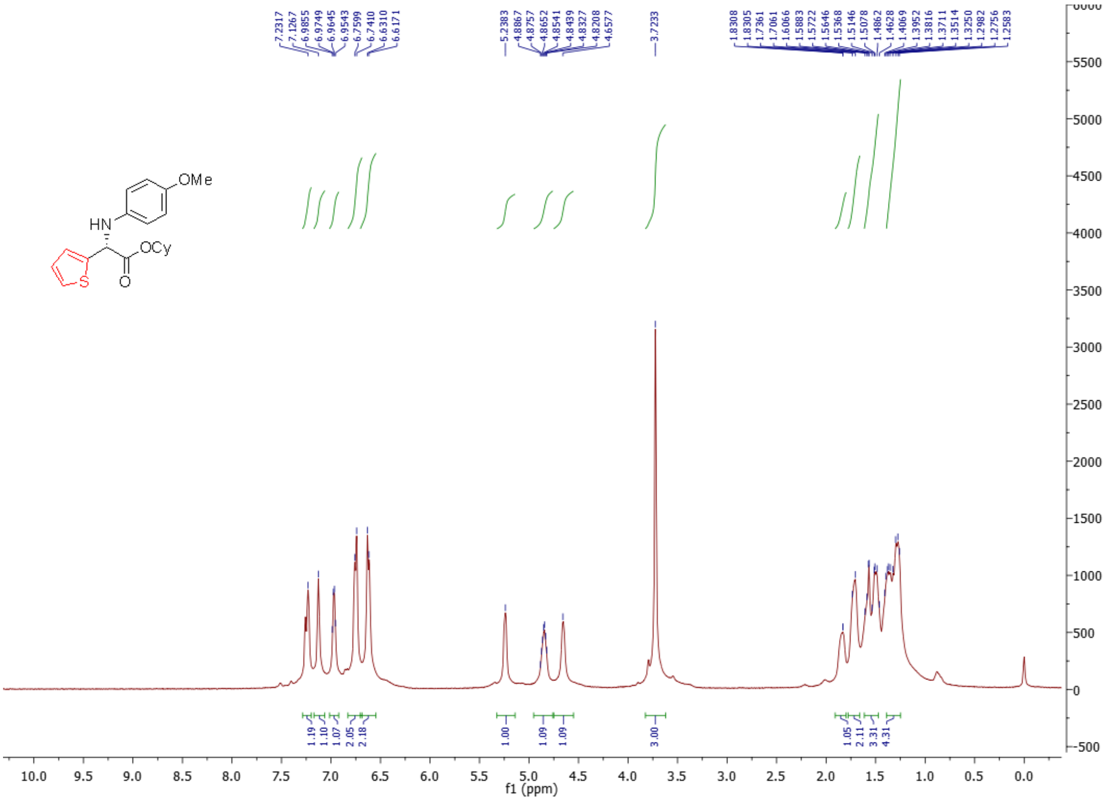

^{1}H NMR of Compound **57** ($CDCl_3$, 400 MHz):

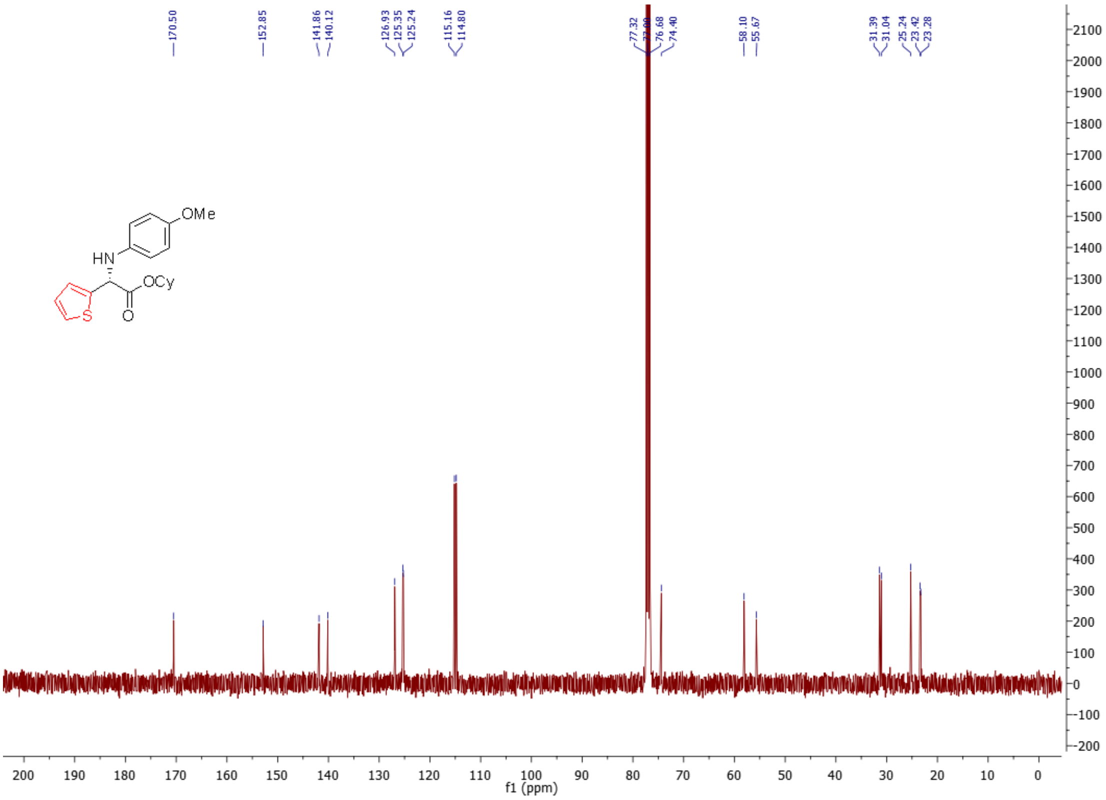

^{1}H NMR of Compound **58** ($CDCl_3$, 400 MHz):

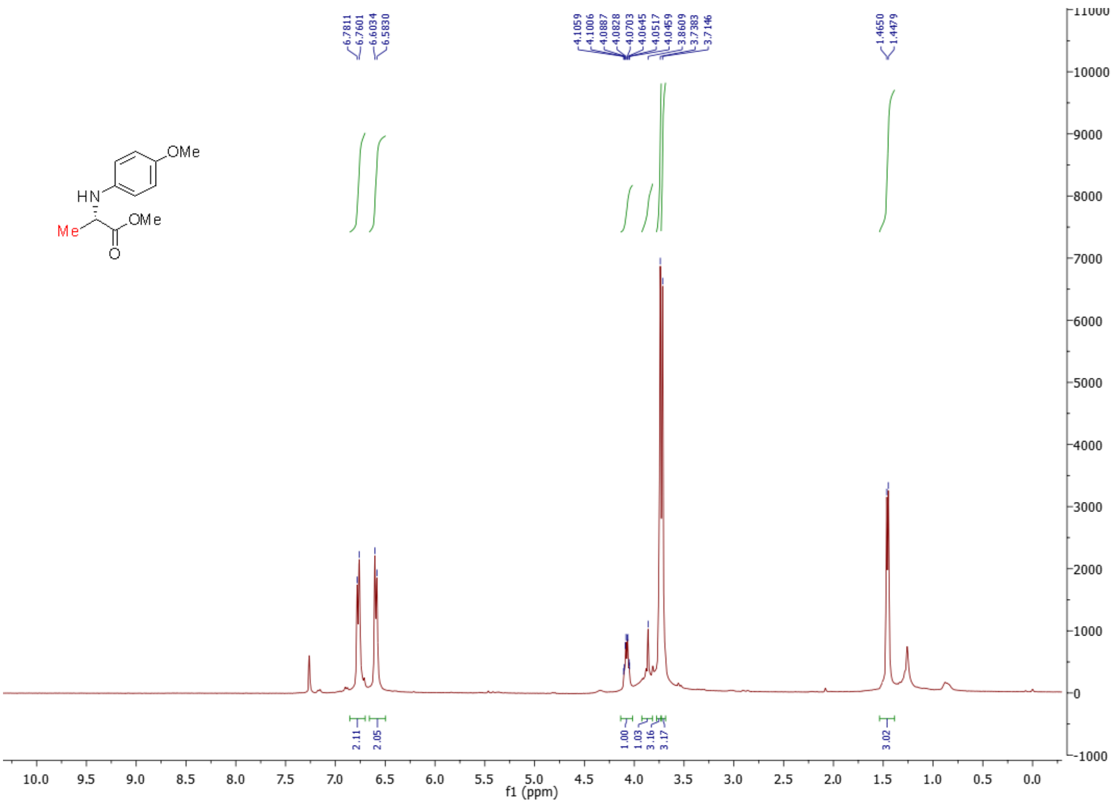

^{1}H NMR of Compound **58** ($CDCl_3$, 400 MHz):

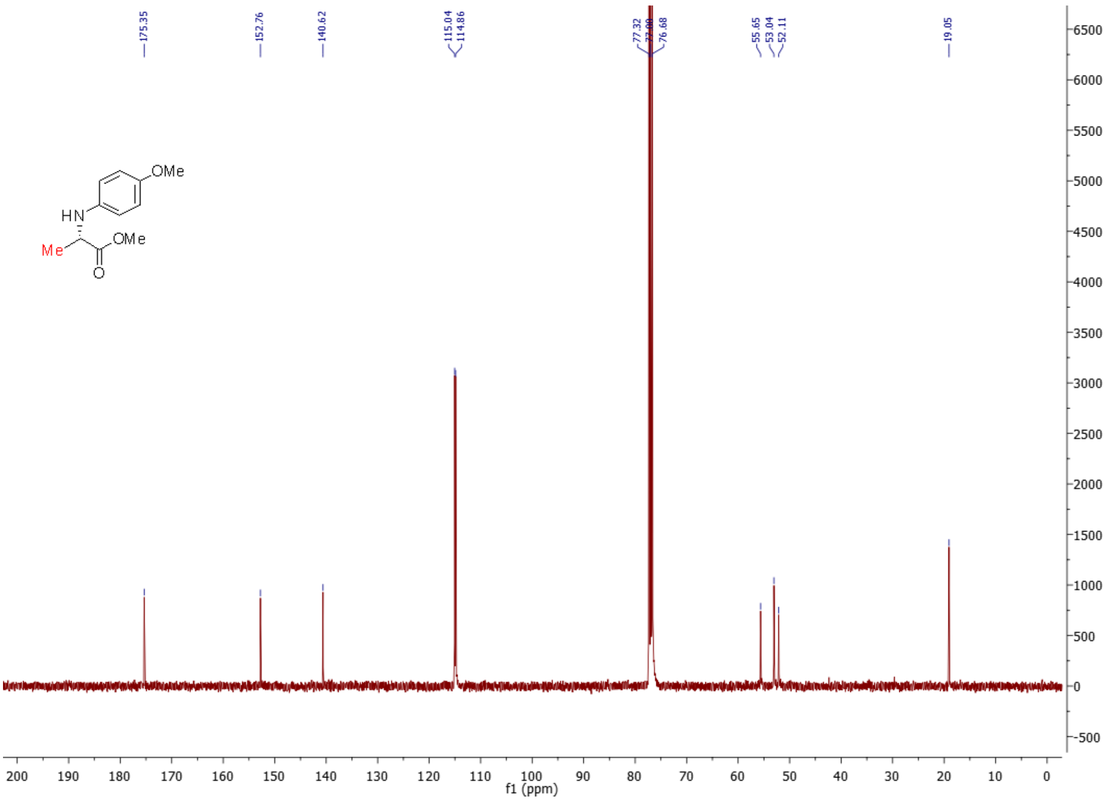

^{1}H NMR of Compound **59** ($CDCl_3$, 400 MHz):

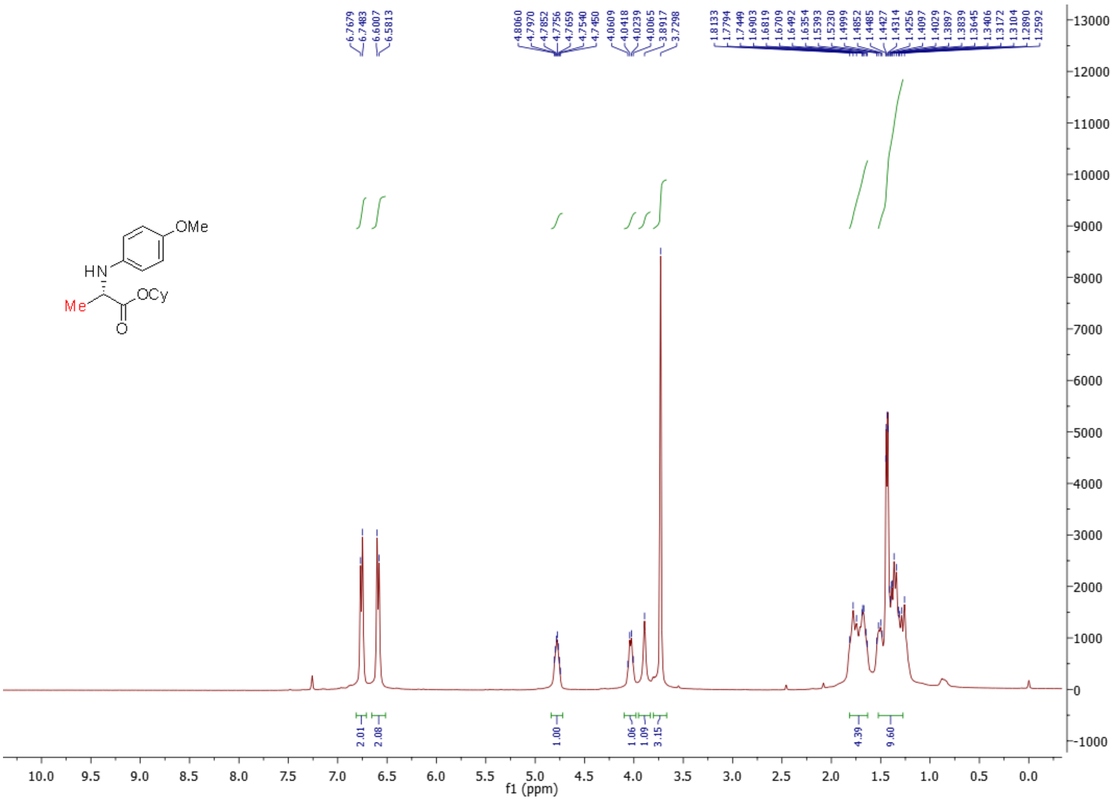

^{1}H NMR of Compound **59** ($CDCl_3$, 400 MHz):

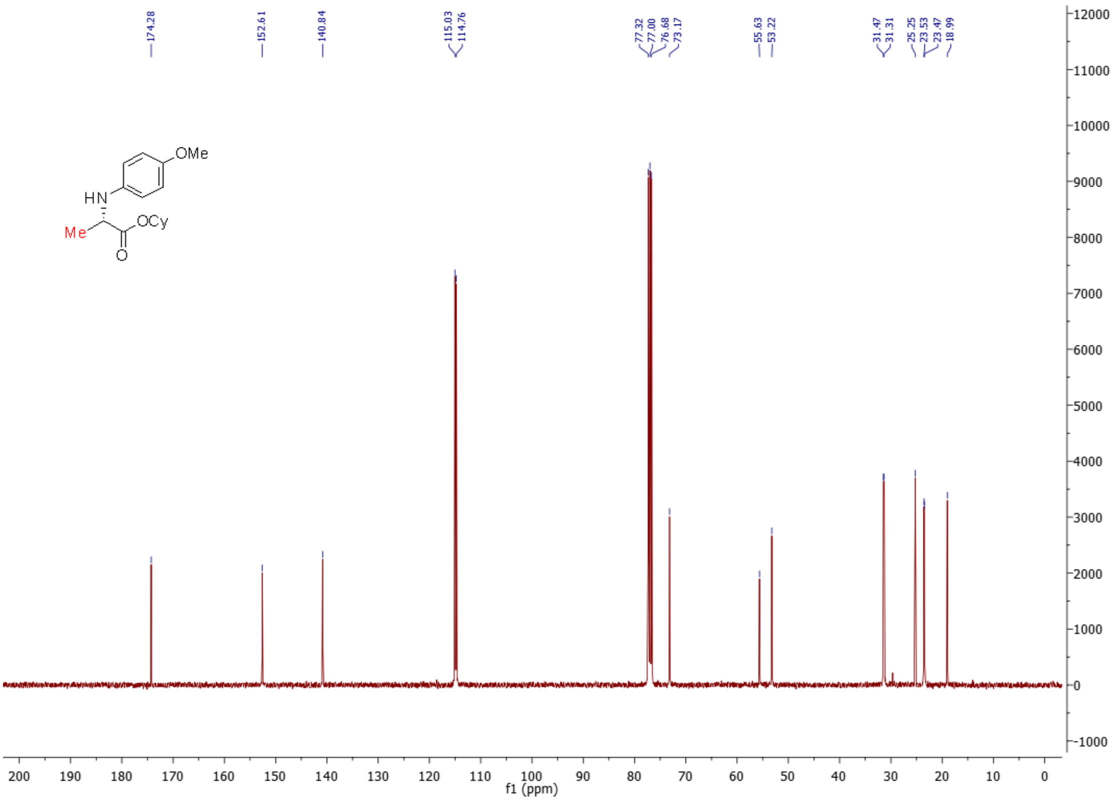

^{1}H NMR of Compound **60** ($CDCl_3$, 400 MHz):

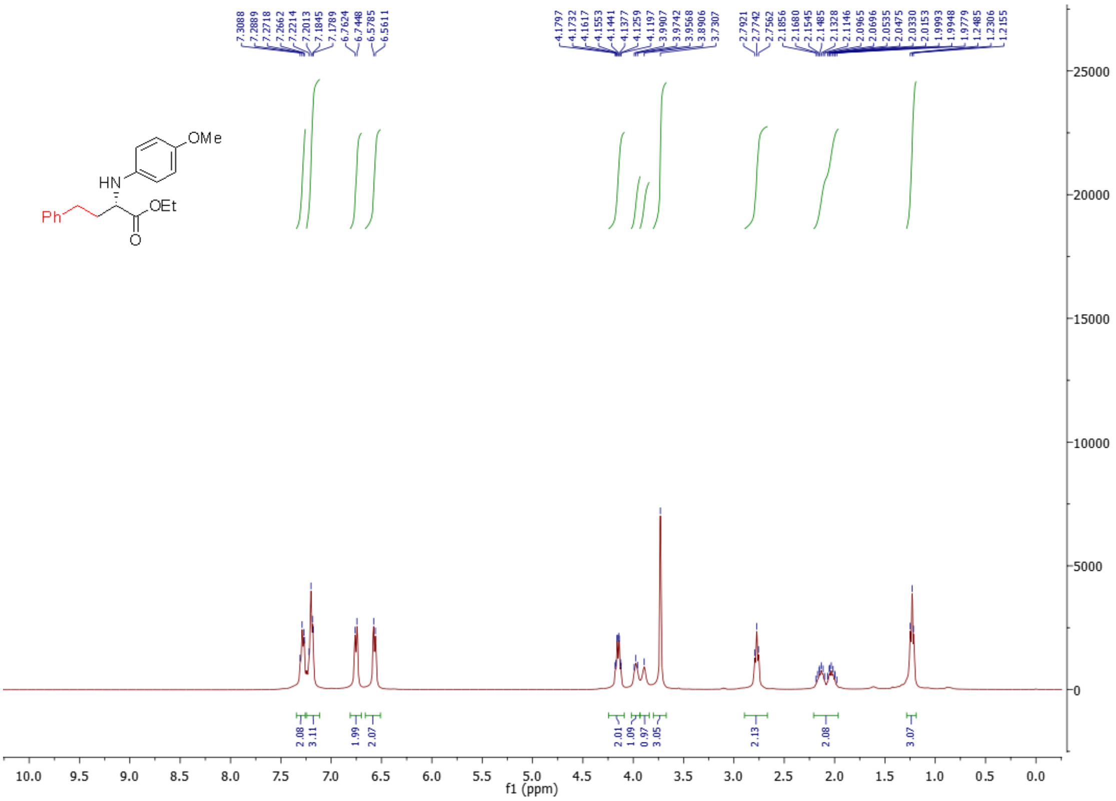

^{1}H NMR of Compound **60** ($CDCl_3$, 400 MHz):

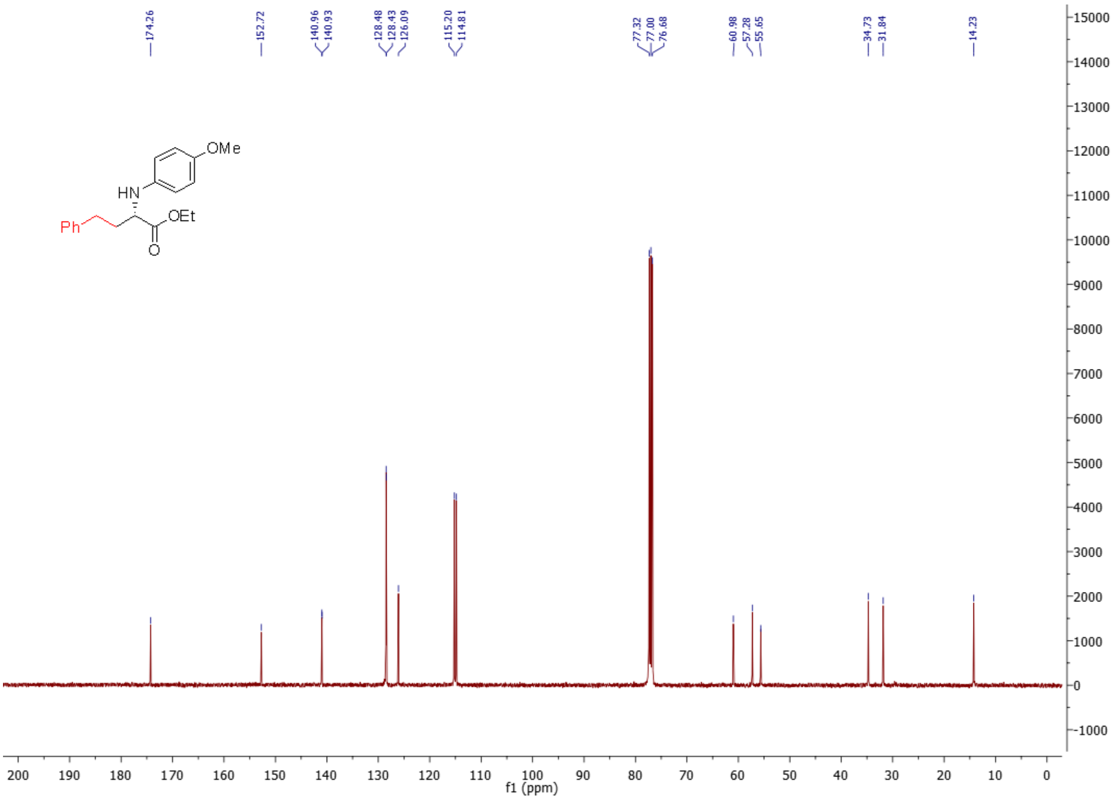

^{1}H NMR of Compound **61** ($CDCl_3$, 400 MHz):

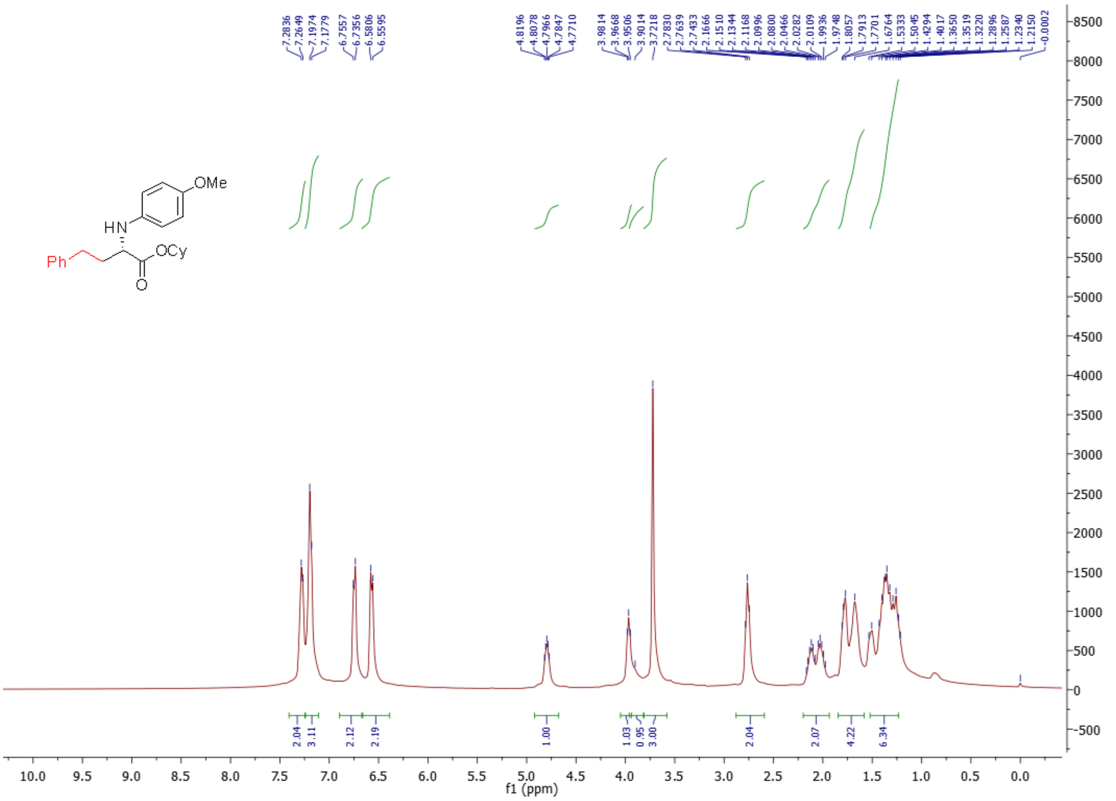

^{1}H NMR of Compound **61** ($CDCl_3$, 400 MHz):

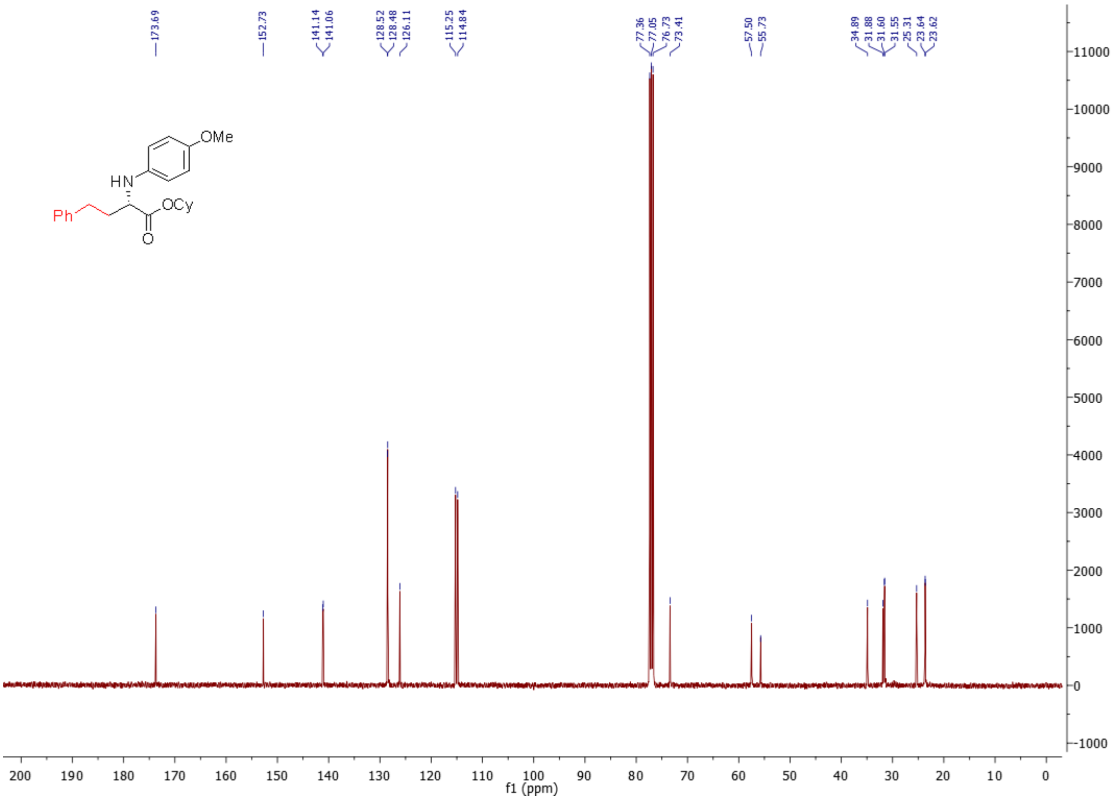

^{1}H NMR of Compound **62** ($CDCl_3$, 400 MHz):

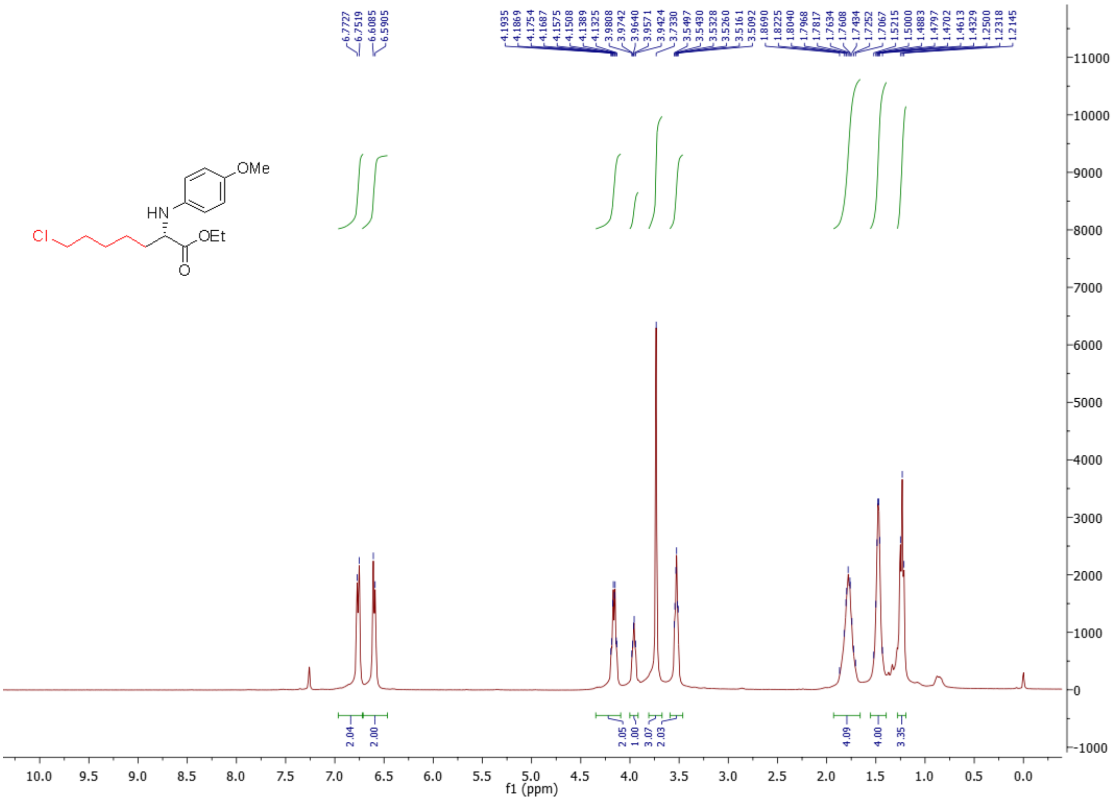

^{1}H NMR of Compound **62** ($CDCl_3$, 400 MHz):

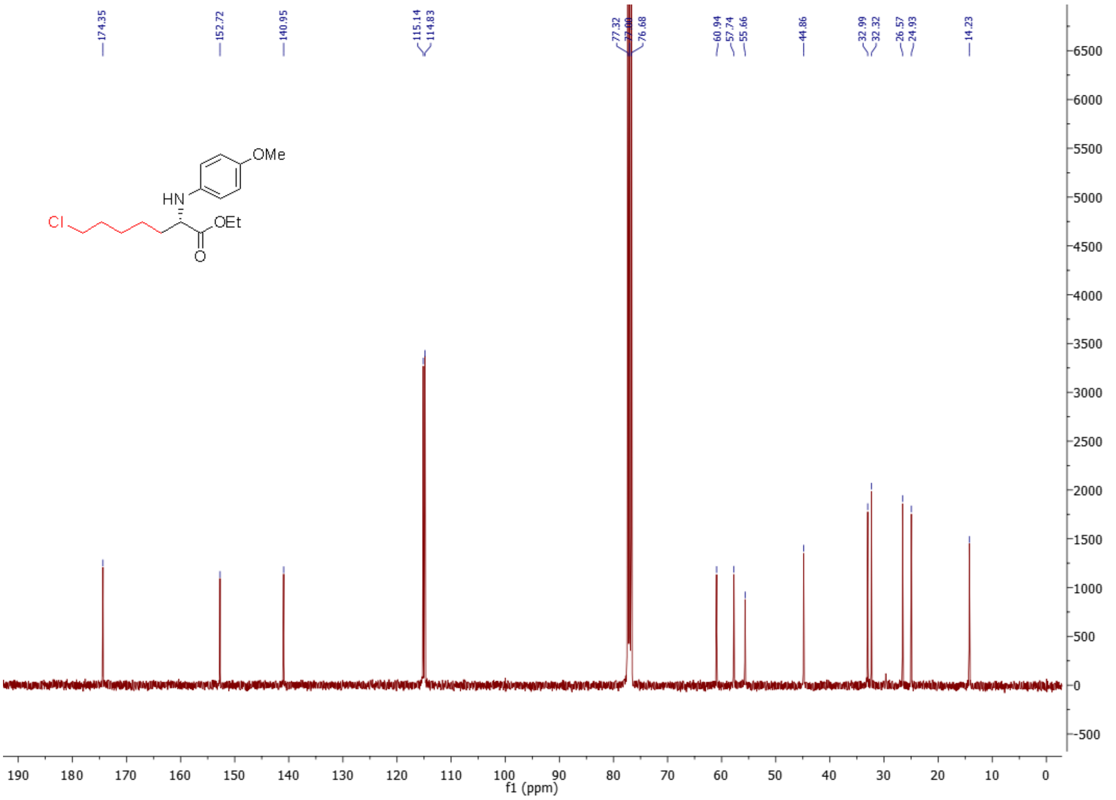

^{1}H NMR of Compound **63** ($CDCl_3$, 400 MHz):

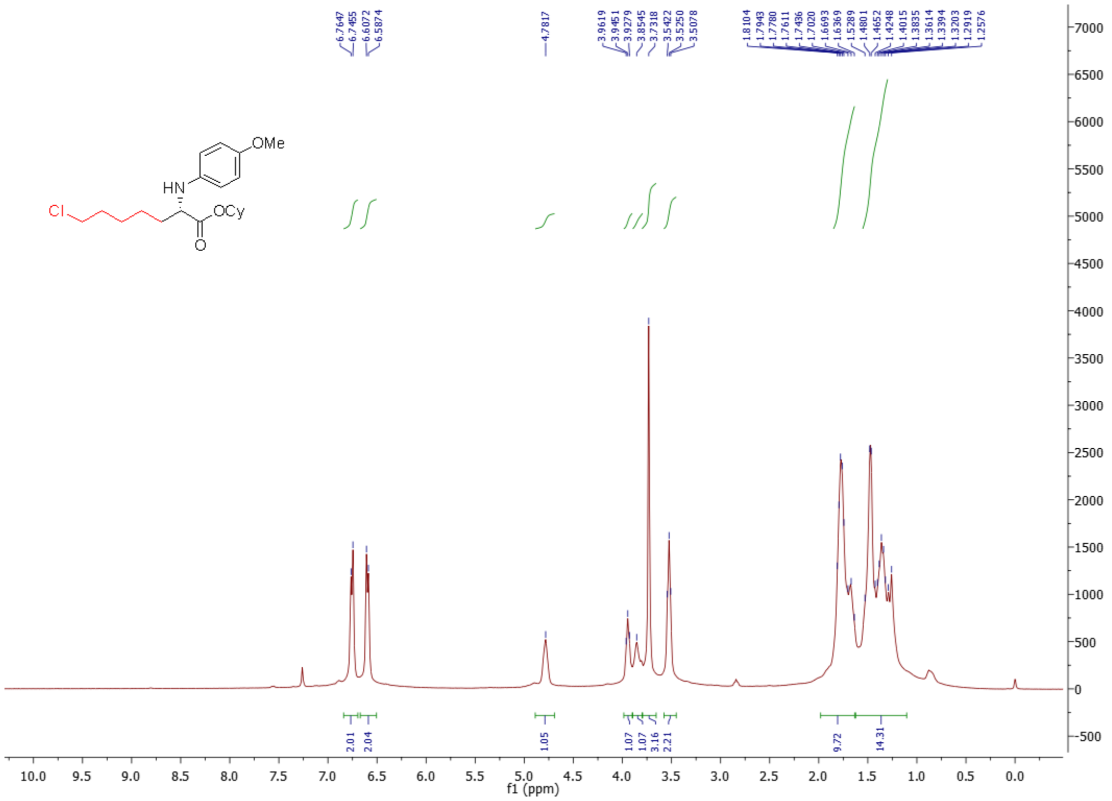

^{1}H NMR of Compound **63** ($CDCl_3$, 400 MHz):

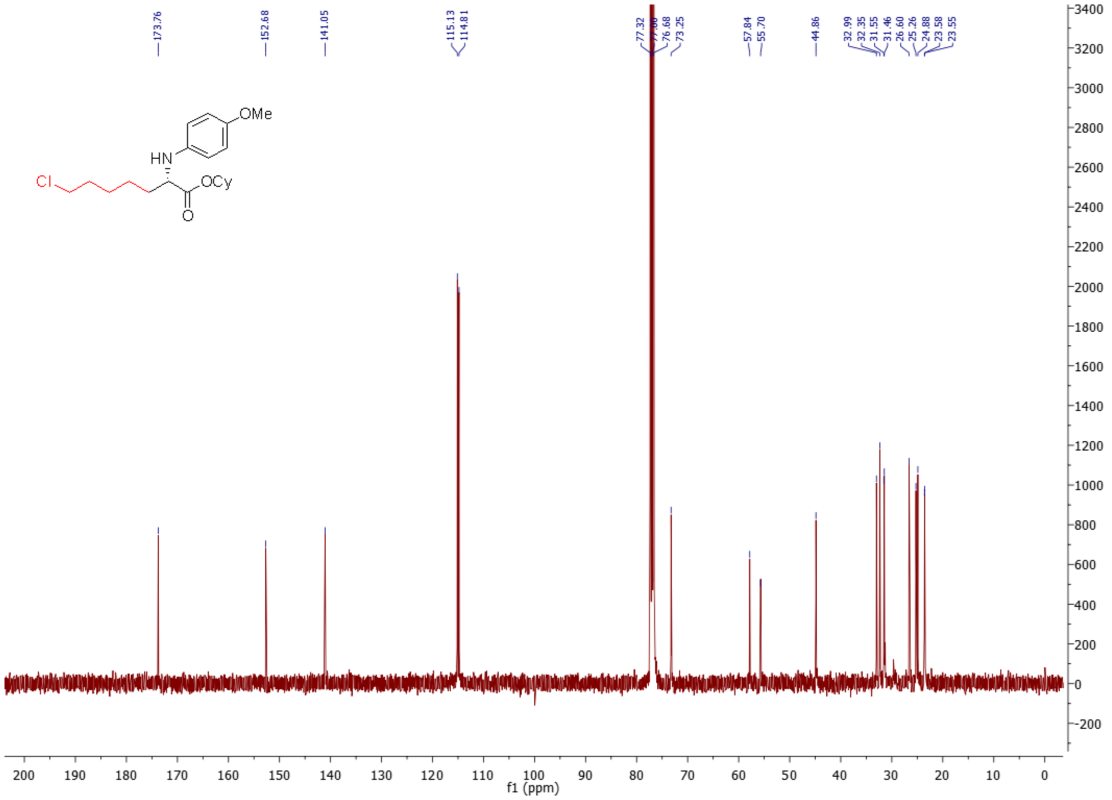

^{1}H NMR of Compound **64** ($CDCl_3$, 400 MHz):

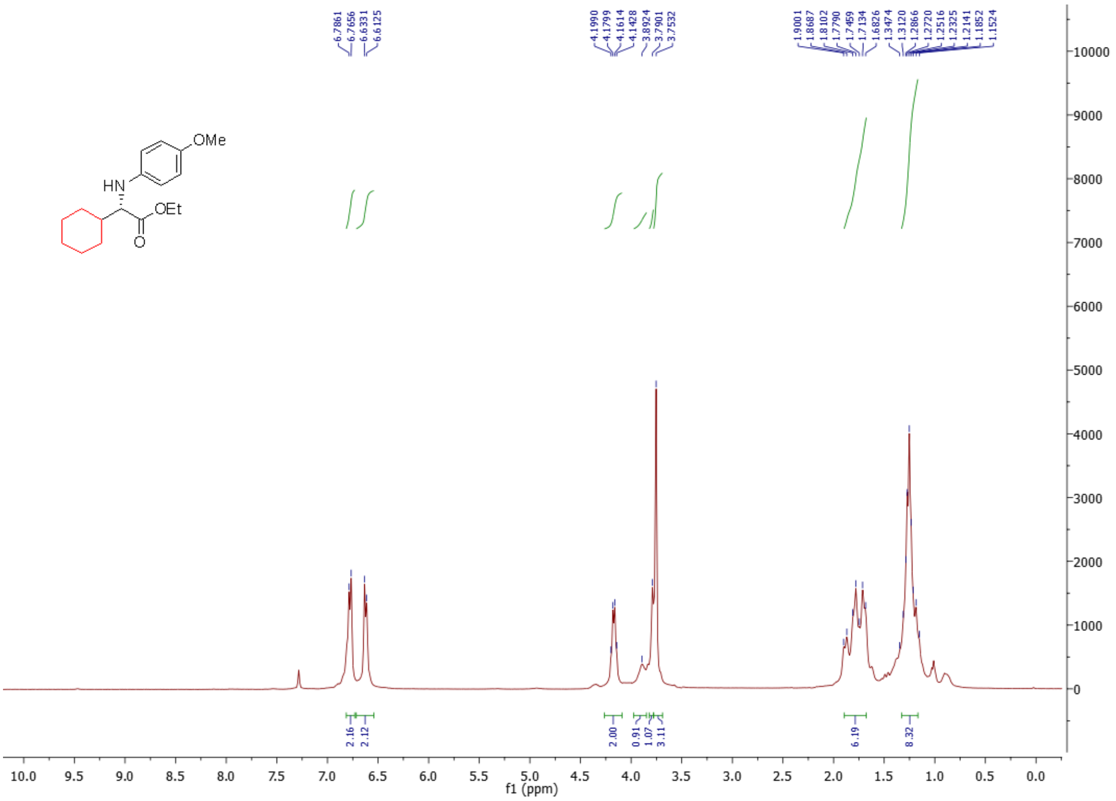

^{1}H NMR of Compound **64** ($CDCl_3$, 400 MHz):

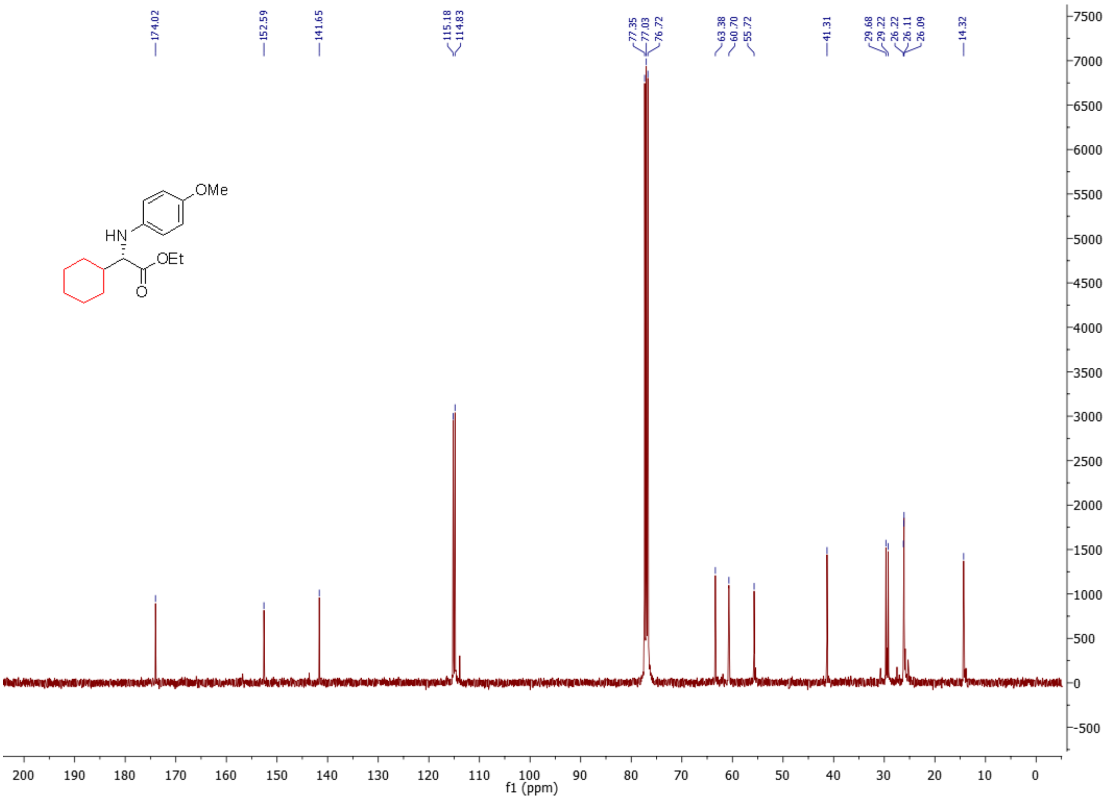

^{1}H NMR of Compound **65** ($CDCl_3$, 400 MHz):

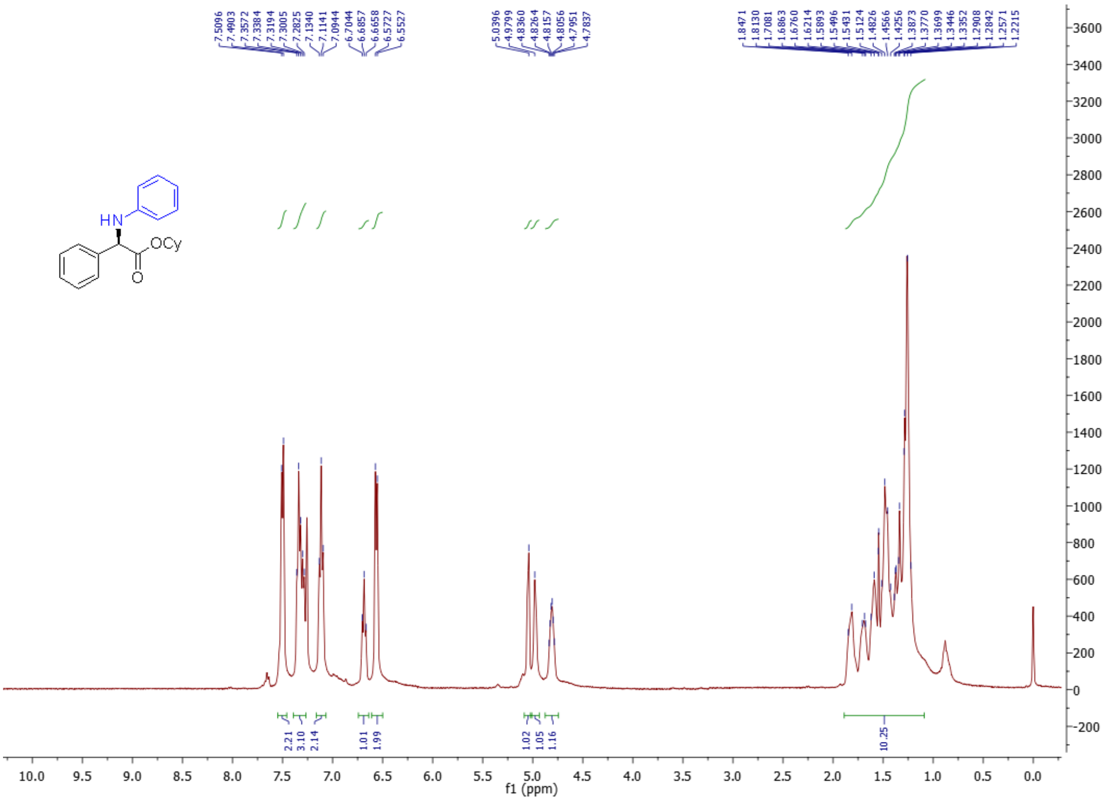

^{1}H NMR of Compound **65** ($CDCl_3$, 400 MHz):

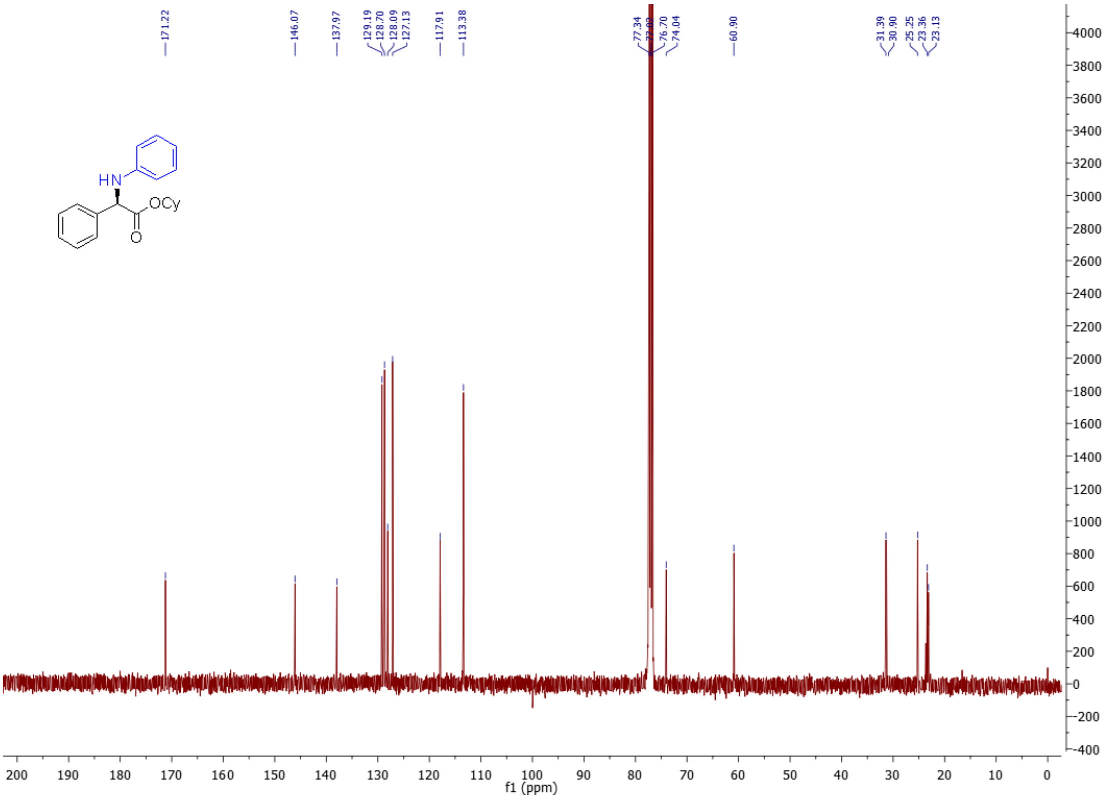

^{1}H NMR of Compound **66** ($CDCl_3$, 400 MHz):

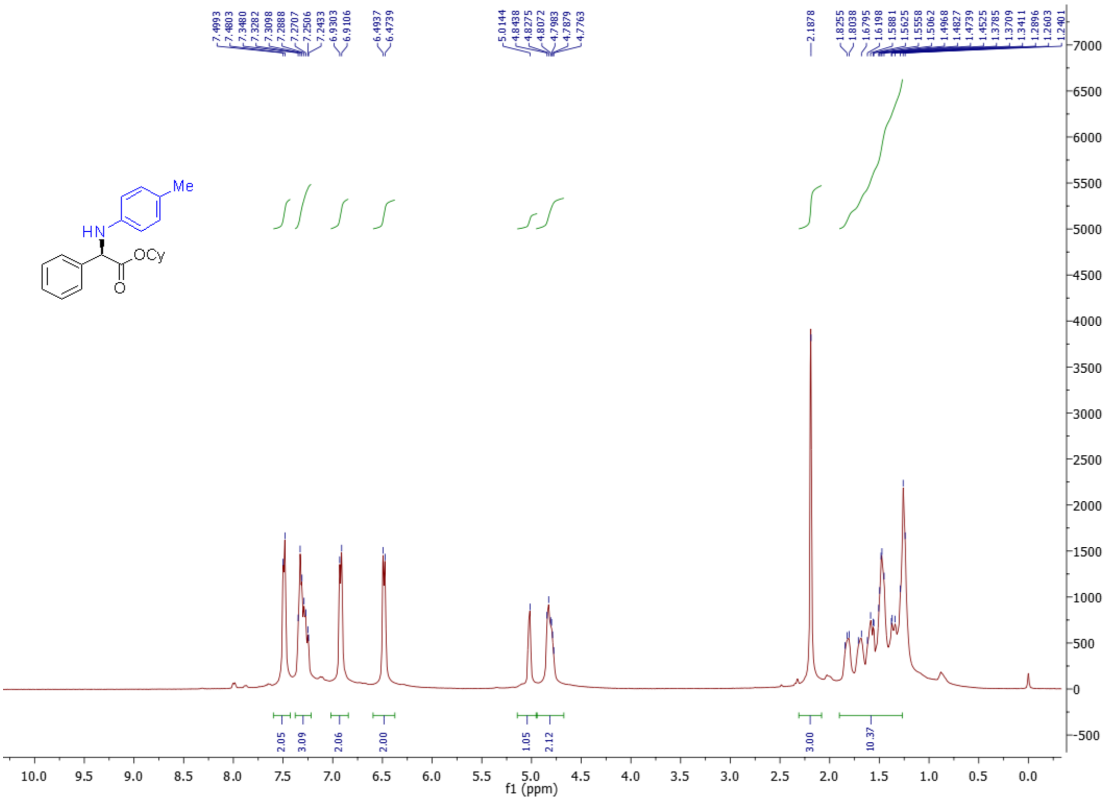

^{1}H NMR of Compound **66** ($CDCl_3$, 400 MHz):

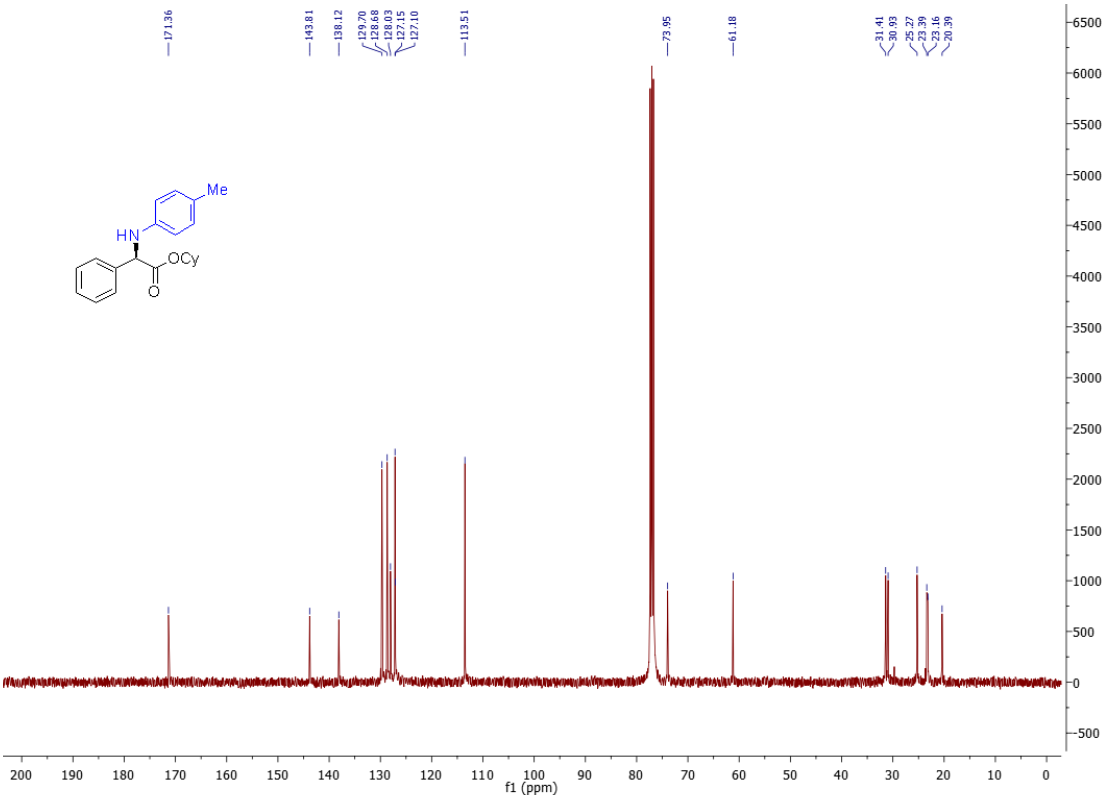

^{1}H NMR of Compound **67** ($CDCl_3$, 400 MHz):

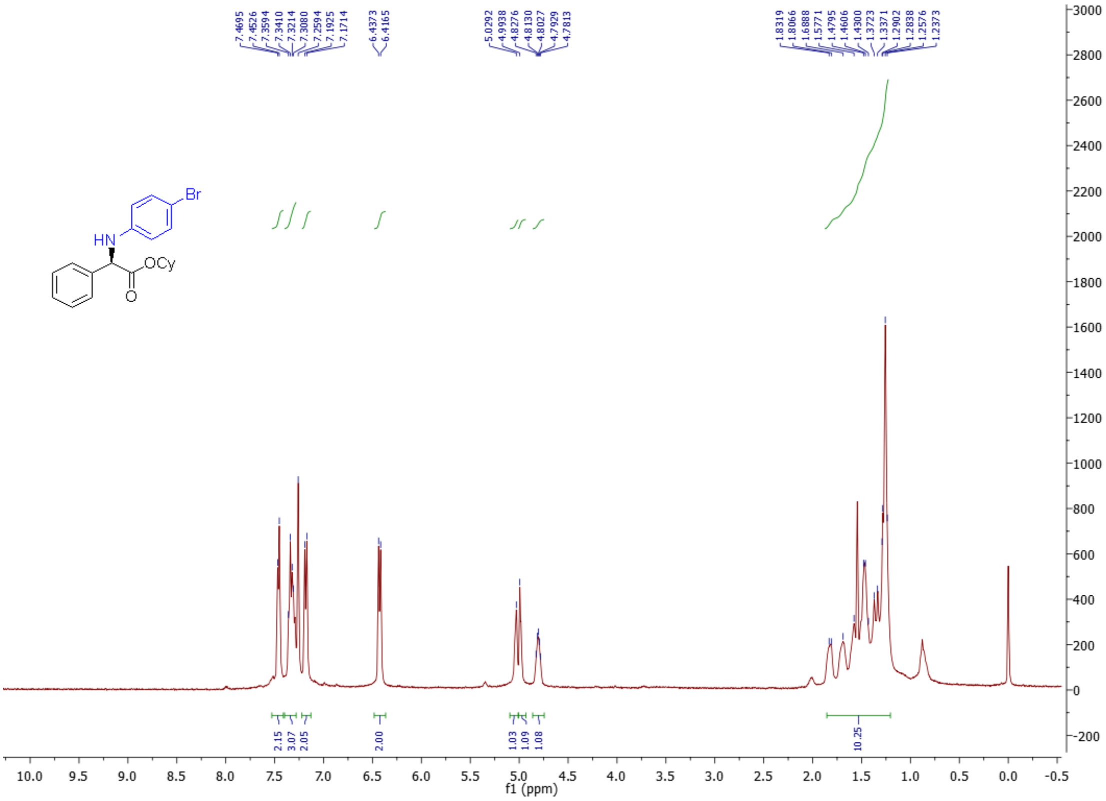

^{1}H NMR of Compound **67** ($CDCl_3$, 400 MHz):

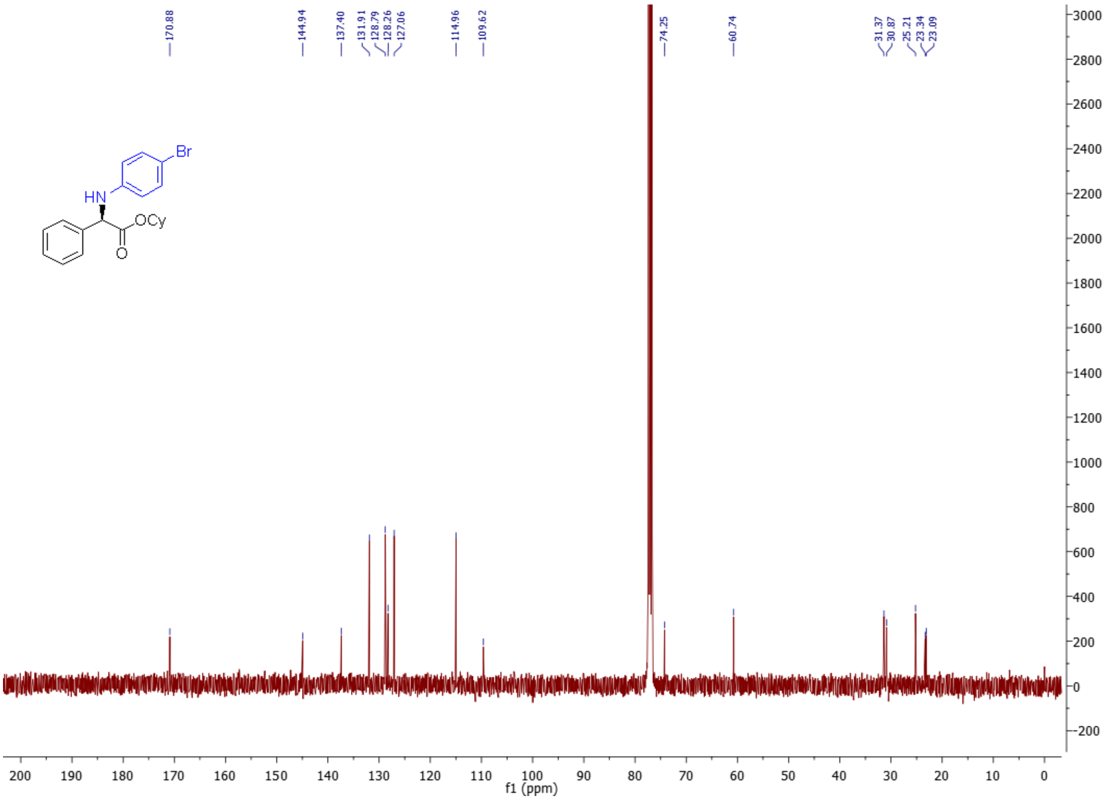

^{1}H NMR of Compound **68** ($CDCl_3$, 400 MHz):

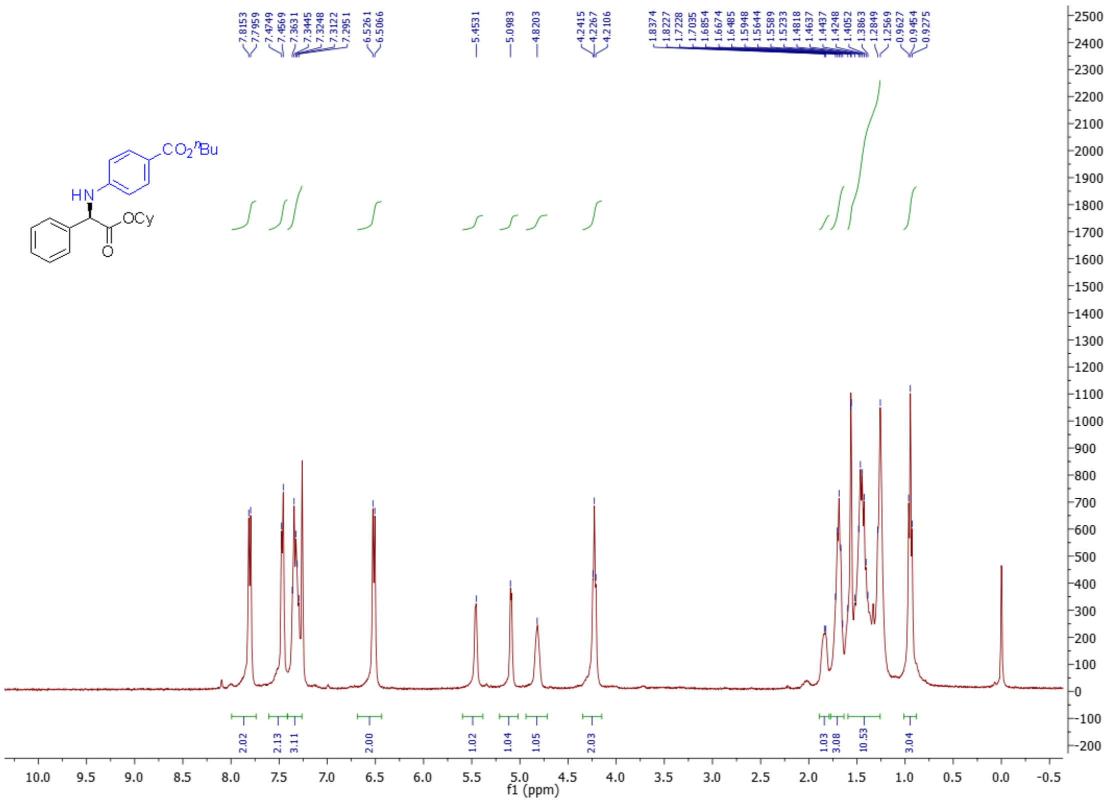

^{1}H NMR of Compound **68** ($CDCl_3$, 400 MHz):

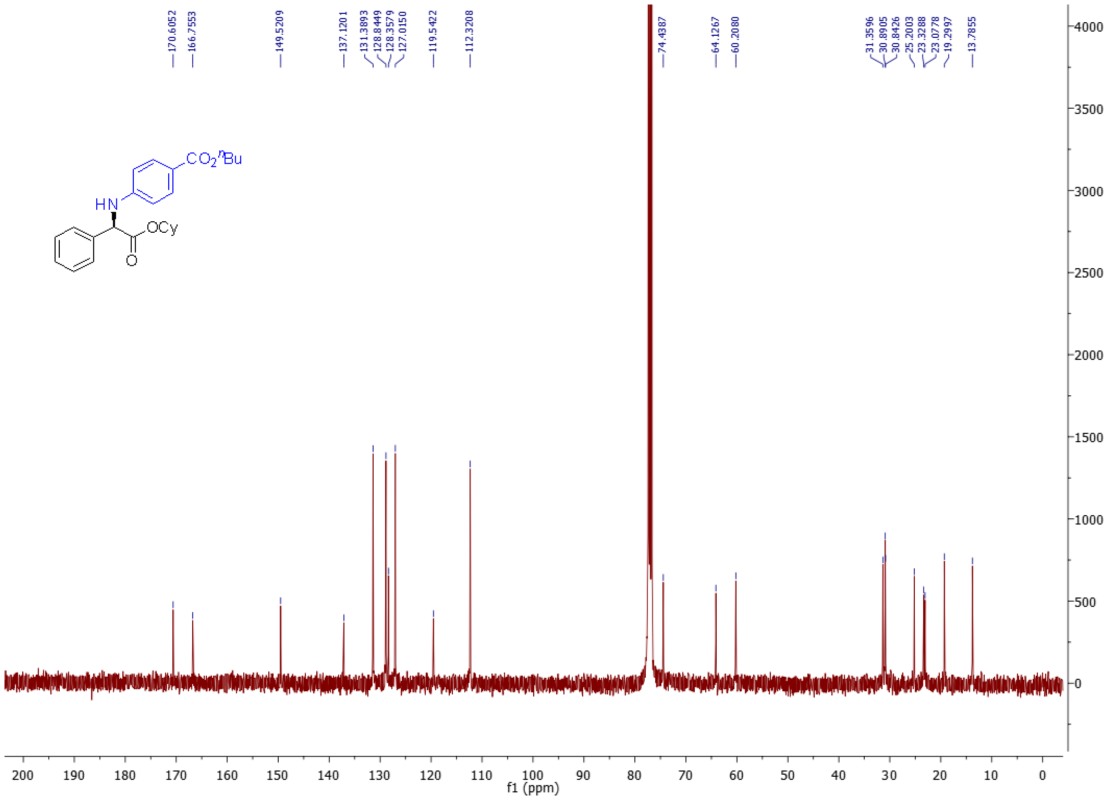

^{1}H NMR of Compound **69** ($CDCl_3$, 400 MHz):

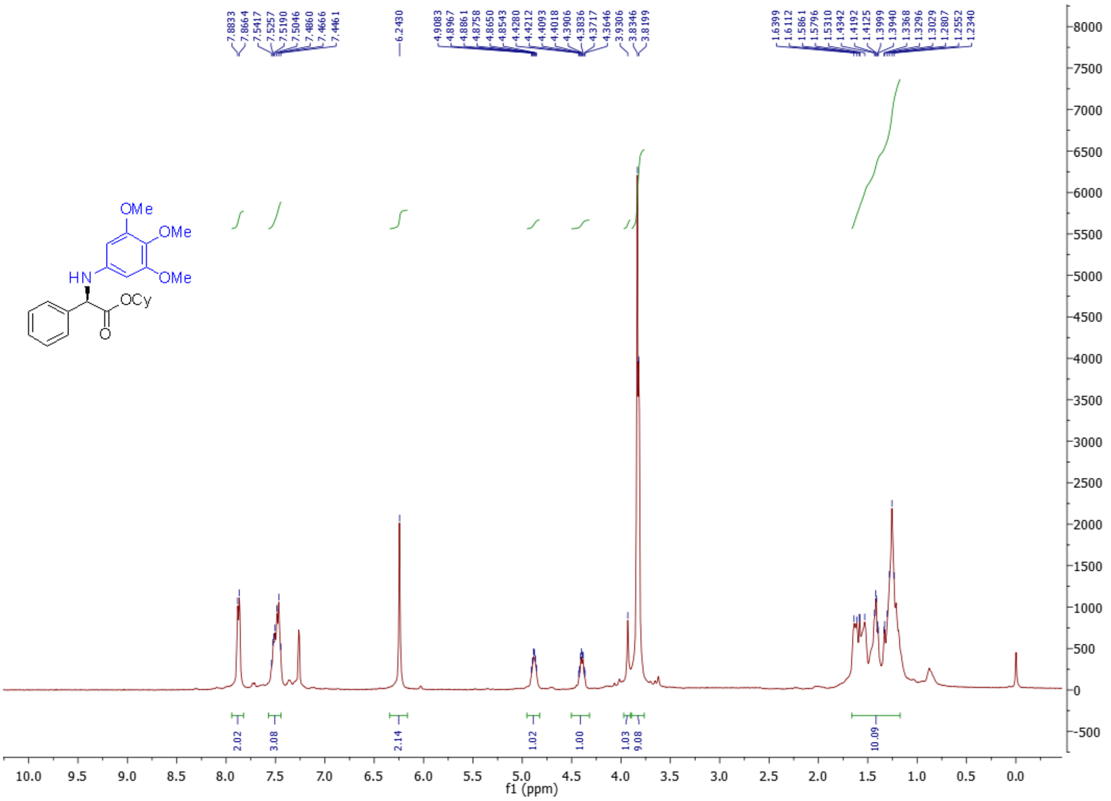

^{1}H NMR of Compound **69** ($CDCl_3$, 400 MHz):

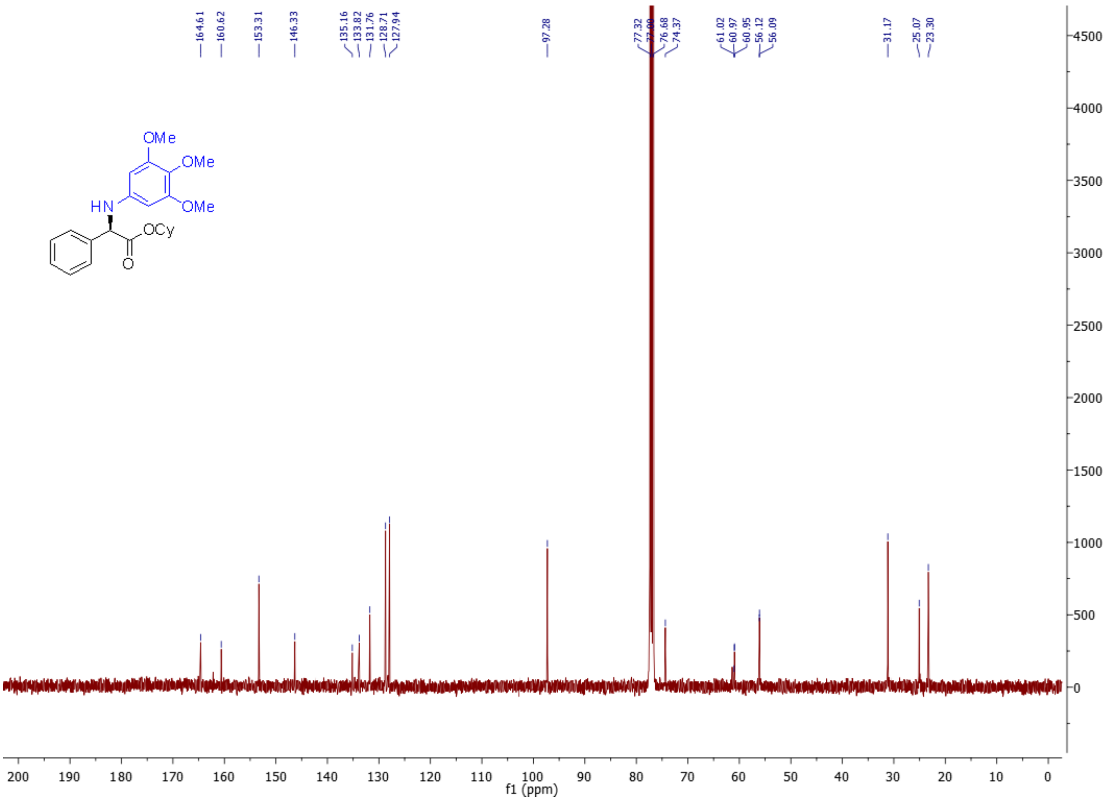

^{1}H NMR of Compound **70** ($CDCl_3$, 400 MHz):

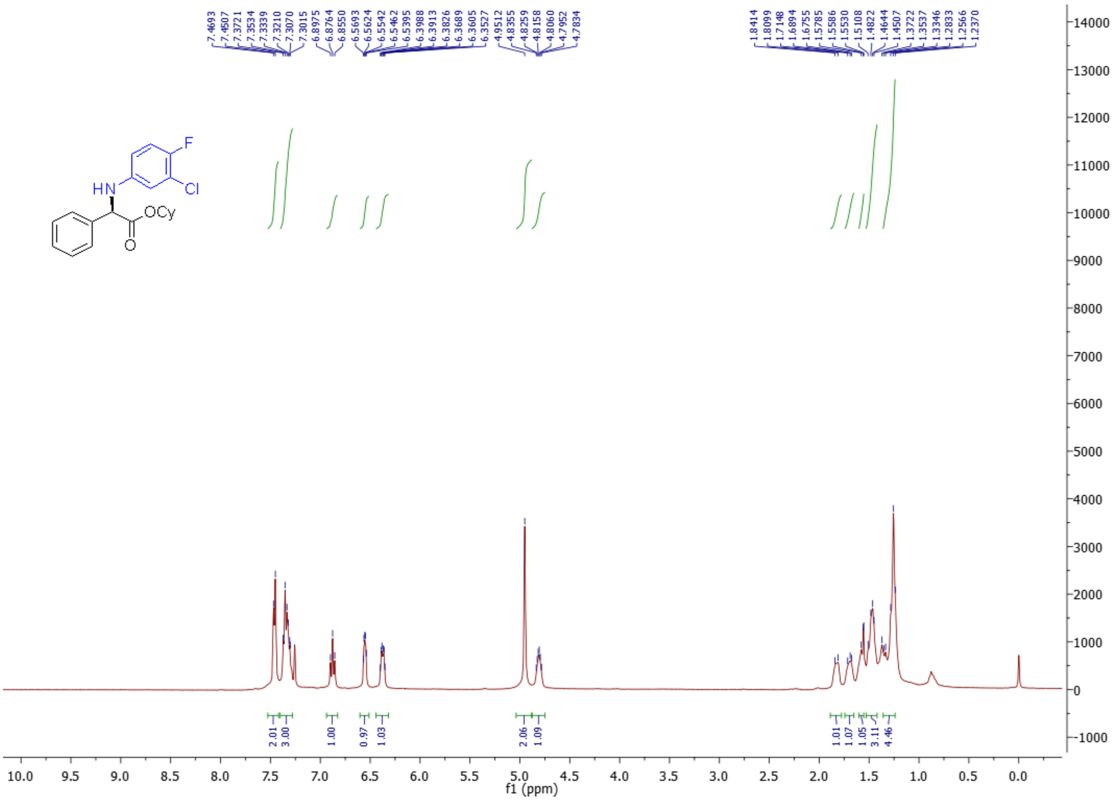

^{1}H NMR of Compound **70** ($CDCl_3$, 400 MHz):

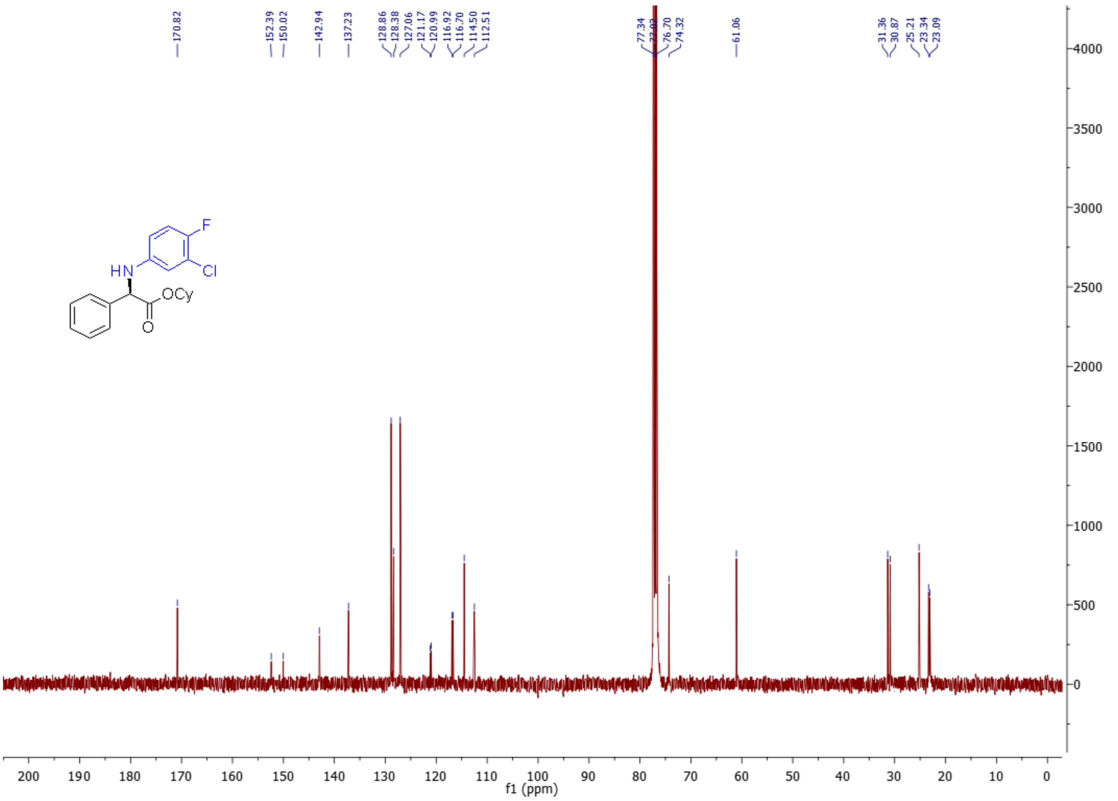

^{1}H NMR of Compound **71** ($CDCl_3$, 400 MHz):

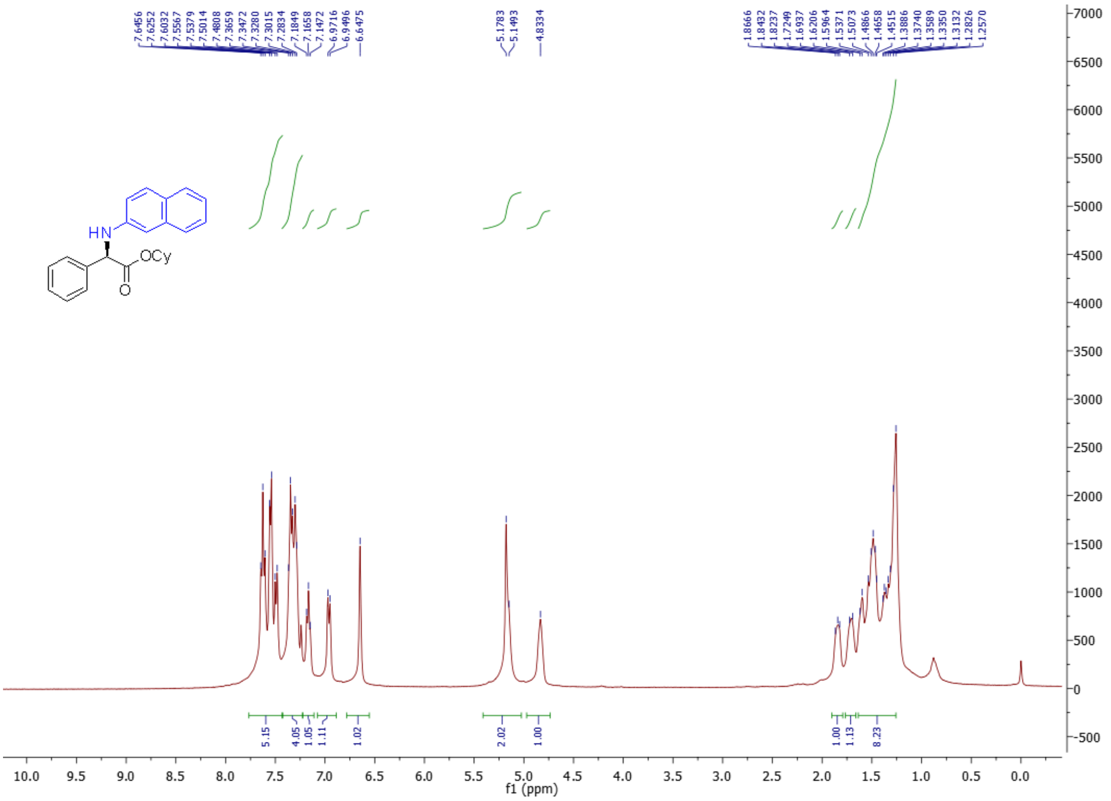

^{1}H NMR of Compound **71** ($CDCl_3$, 400 MHz):

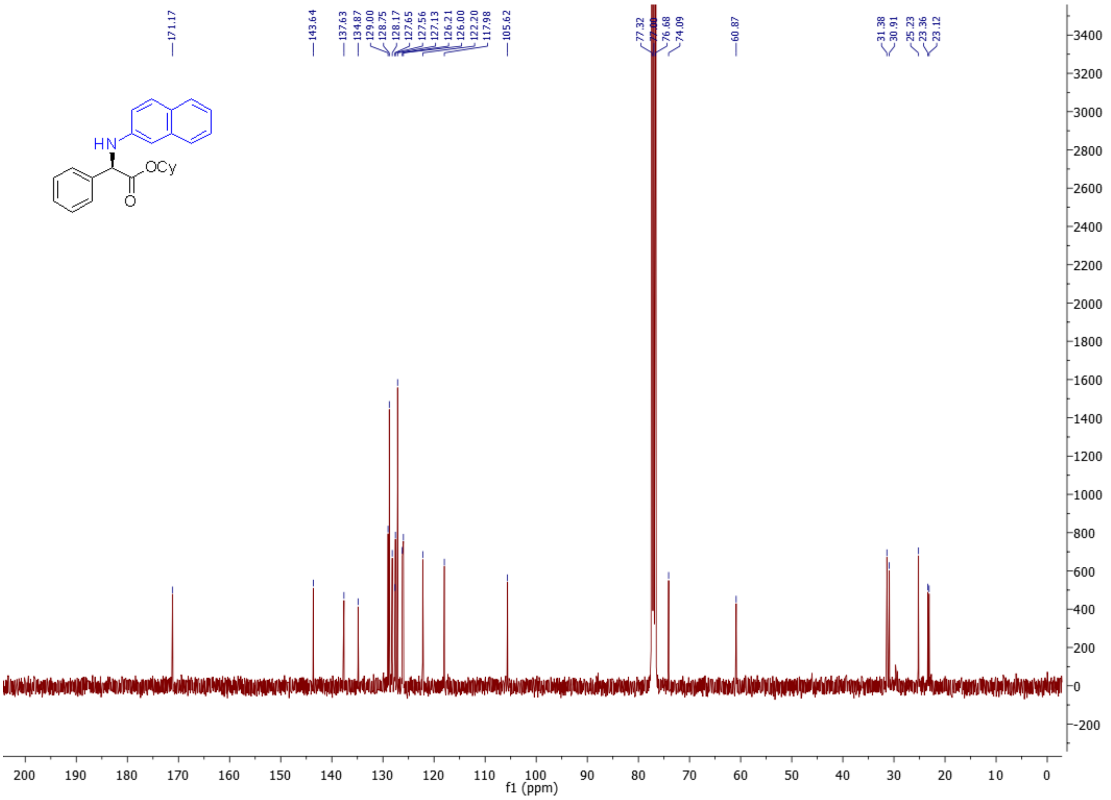

^{1}H NMR of Compound **73** ($CDCl_3$, 400 MHz):

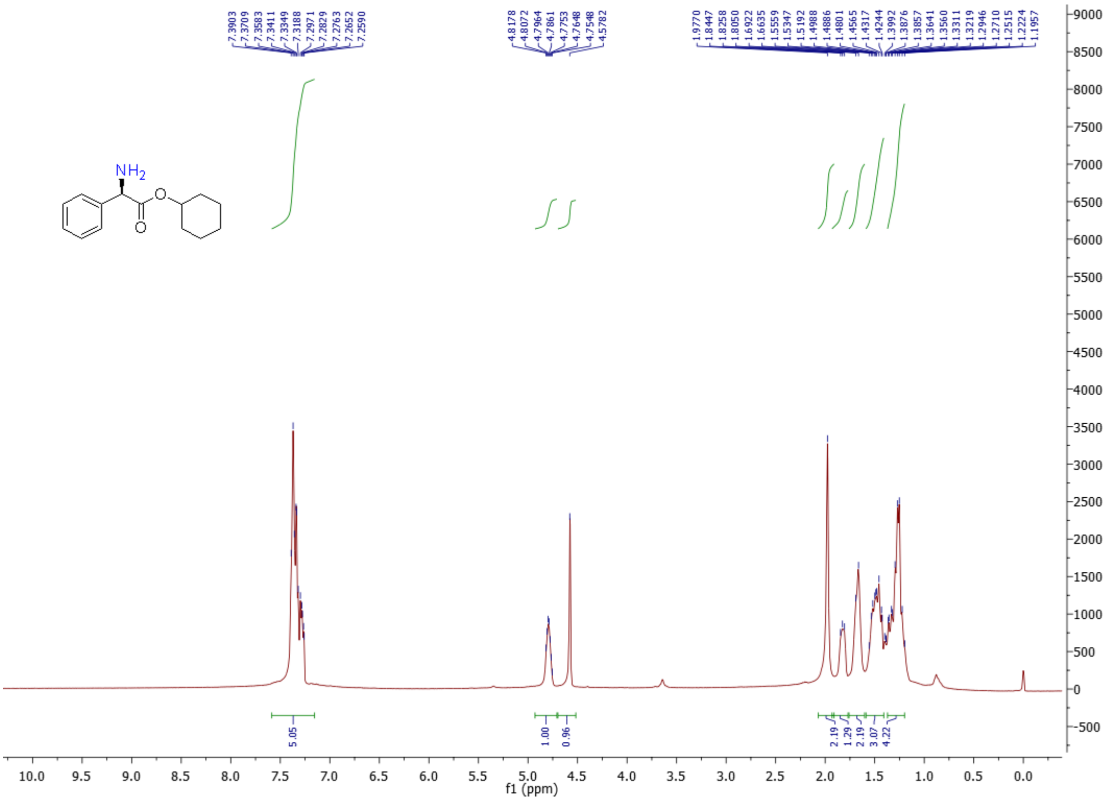

^{1}H NMR of Compound **73** ($CDCl_3$, 400 MHz):

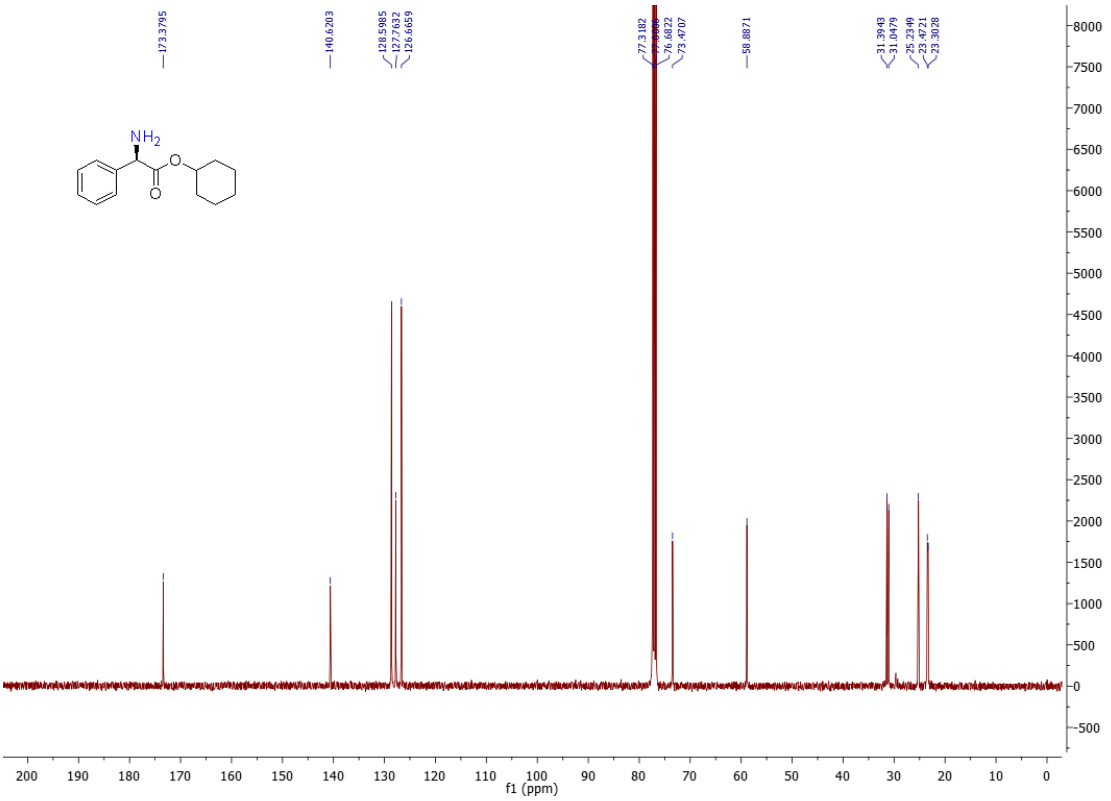

^{1}H NMR of Compound **74** ($CDCl_3$, 400 MHz):

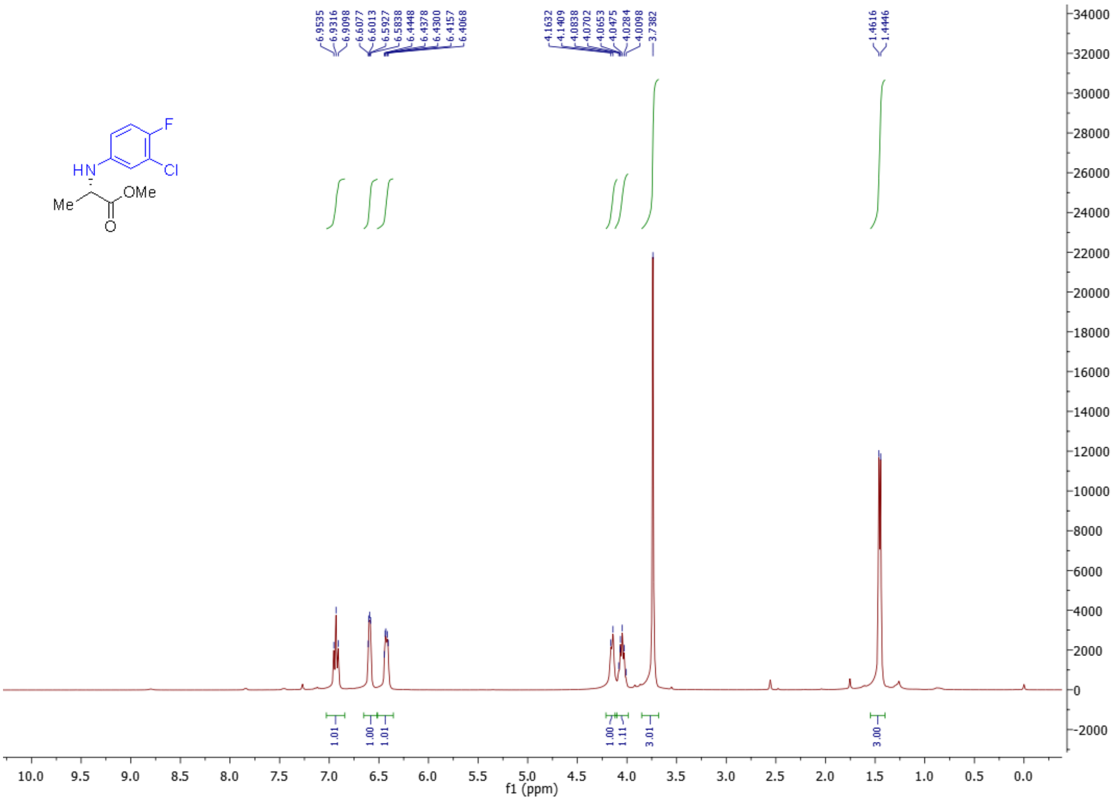

^{1}H NMR of Compound **74** ($CDCl_3$, 400 MHz):

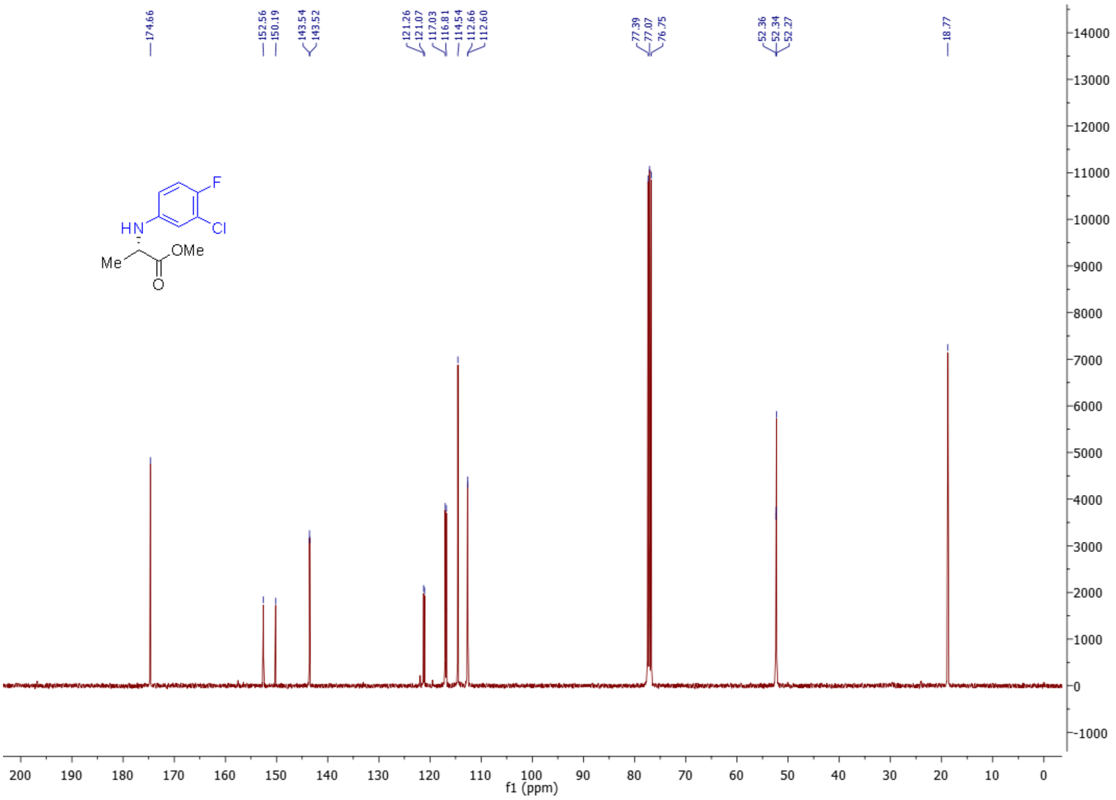

^{1}H NMR of Compound **75** ($CDCl_3$, 400 MHz):

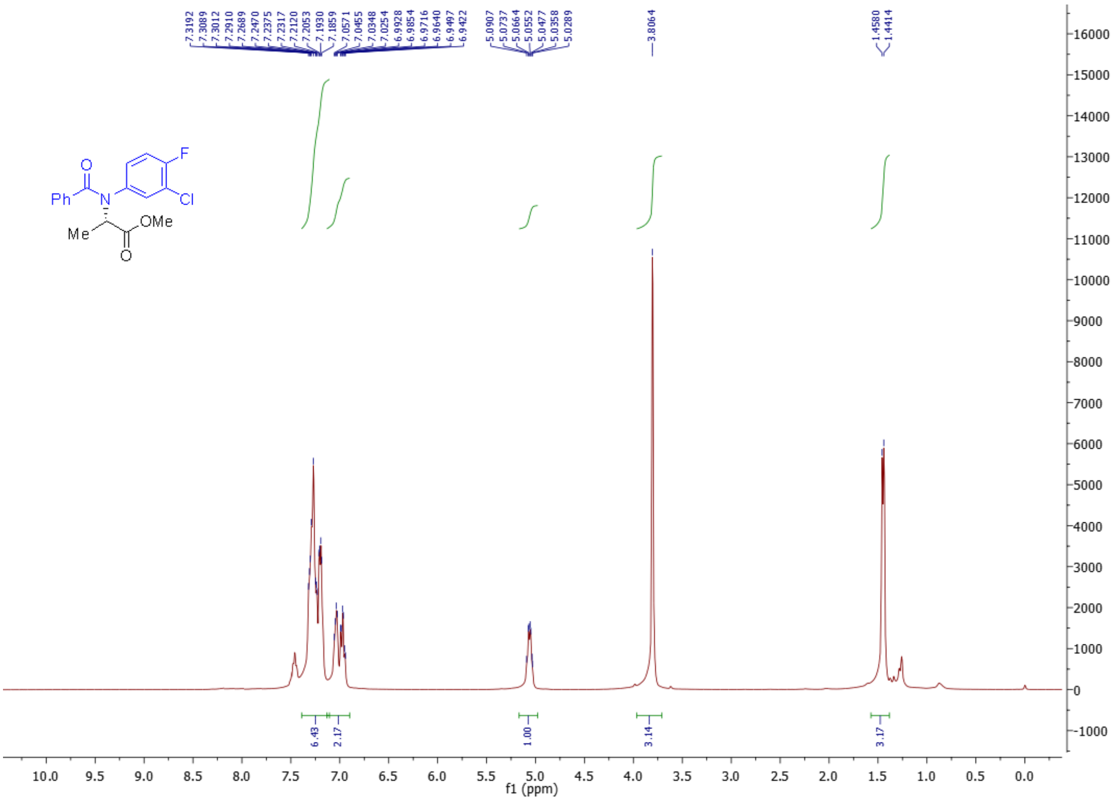

^{1}H NMR of Compound **75** ($CDCl_3$, 400 MHz):

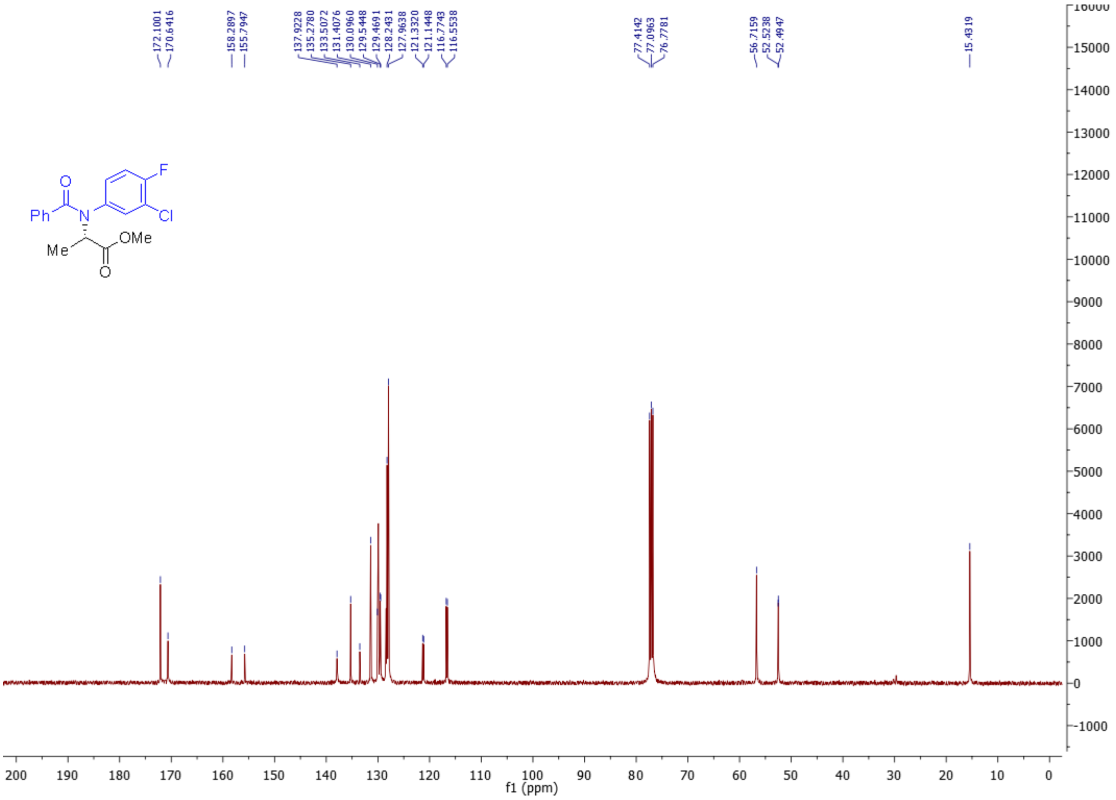

^{1}H NMR of compound α-**imine ester** ($CDCl_3$, 400 MHz):

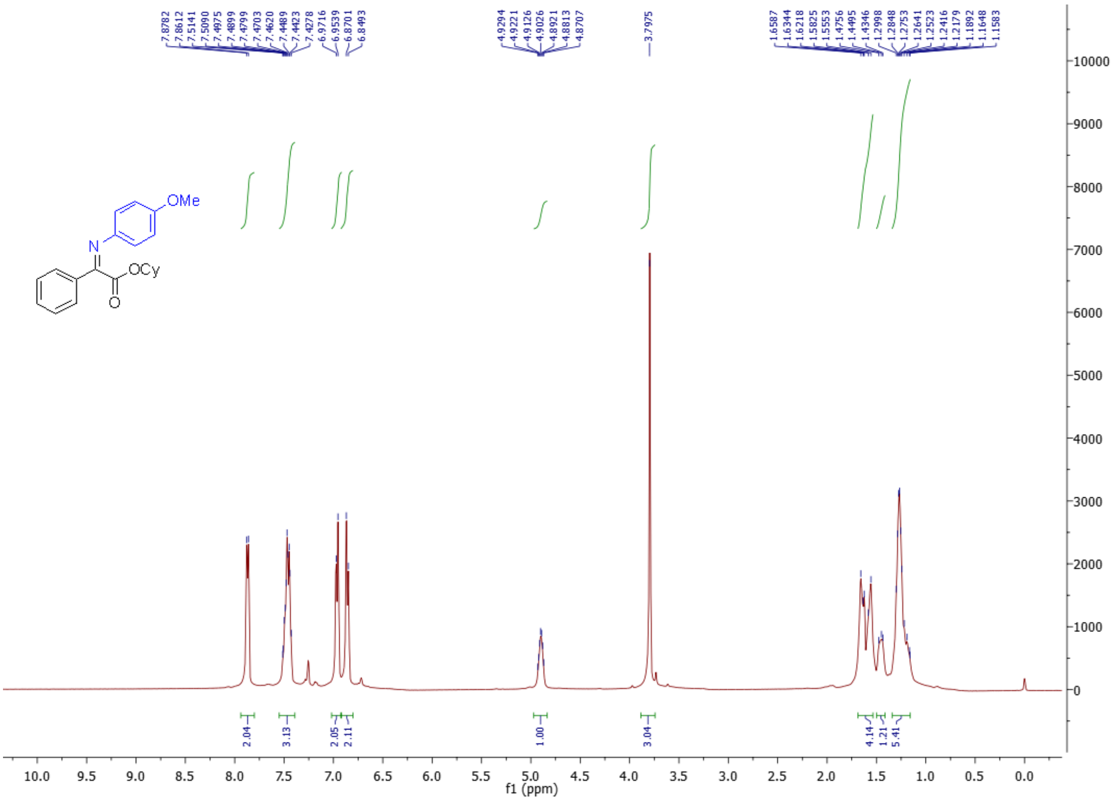

^{1}H NMR of Compound α-**imine ester** ($CDCl_3$, 400 MHz):

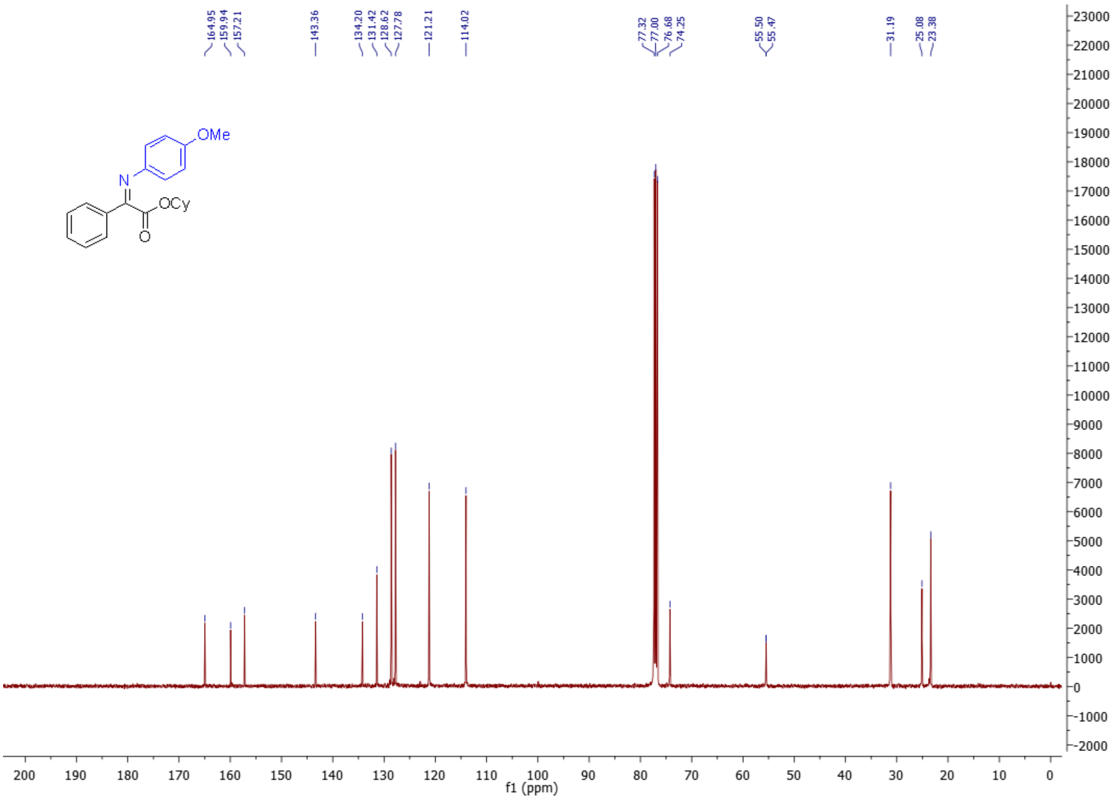

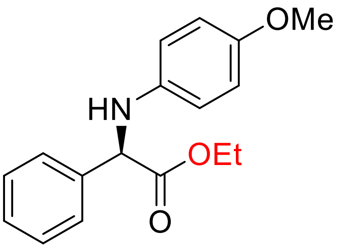

48

Comment:

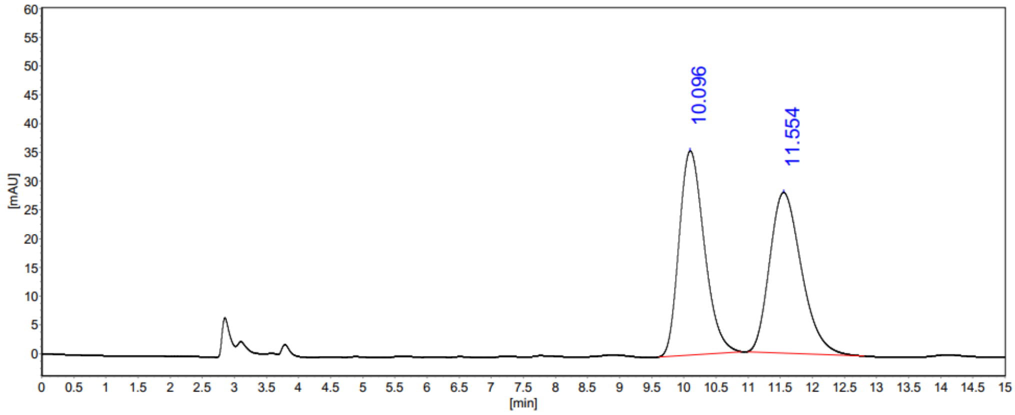

Analysis Results

No.	Compound	R.Time	Height	Area	Area%	Conc.	Type
1		10.096	35486.5	968429.3	50.1744	50.1744	+ BB
2		11.554	27916.5	961695.7	49.8256	49.8256	+ BB
	**Total:**		**63403.0**	**1930125.0**	**100.0000**	**100.0000**	

Comment:

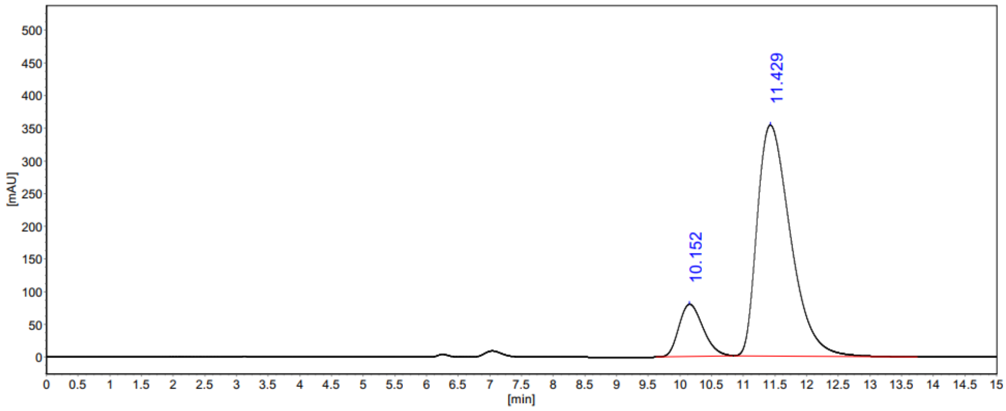

Analysis Results

No.	Compound	R.Time	Height	Area	Area%	Conc.	Type
1		10.152	79833.2	2108159.0	14.0188	14.0188	+ BB
2		11.429	353178.7	12929932.3	85.9812	85.9812	+ BB
	**Total:**		**433011.9**	**15038091.3**	**100.0000**	**100.0000**	

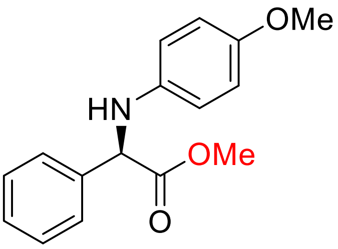

49

Comment:

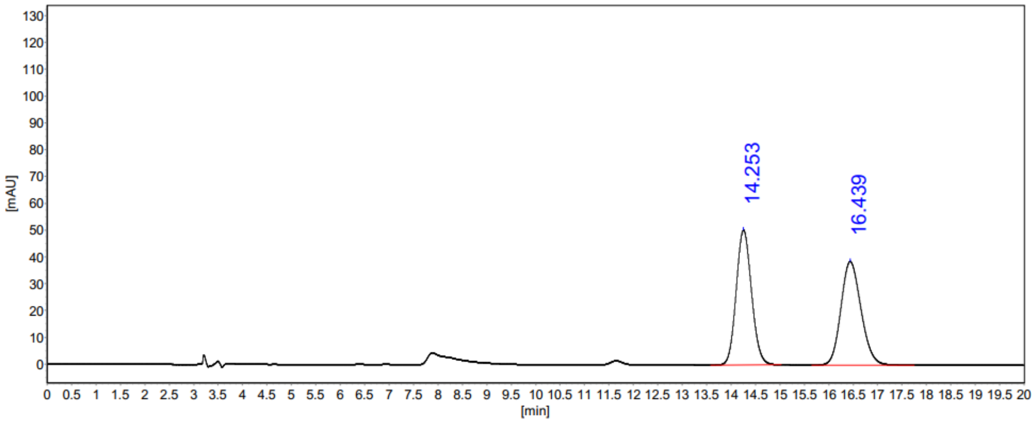

Analysis Results

No.	Compound	R.Time	Height	Area	Area%	Conc.	Type
1		14.253	50321.9	1098764.0	49.8962	49.8962	+ BB
2		16.439	38655.6	1103335.7	50.1038	50.1038	+ BB
	**Total:**		**88977.5**	**2202099.7**	**100.0000**	**100.0000**	

Comment:

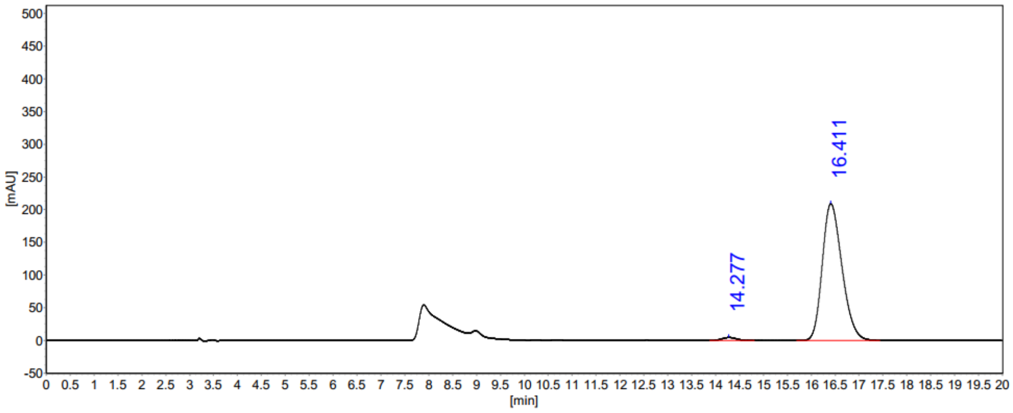

Analysis Results

No.	Compound	R.Time	Height	Area	Area%	Conc.	Type
1		14.277	4514.7	92980.3	1.5278	1.5278	+ BB
2		16.411	209266.1	5993001.3	98.4722	98.4722	+ BB
	**Total:**		**213780.8**	**6085981.5**	**100.0000**	**100.0000**	

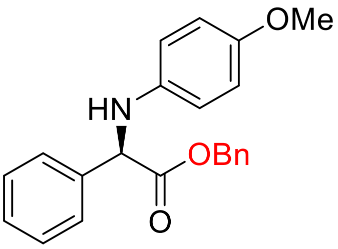

50

Comment:

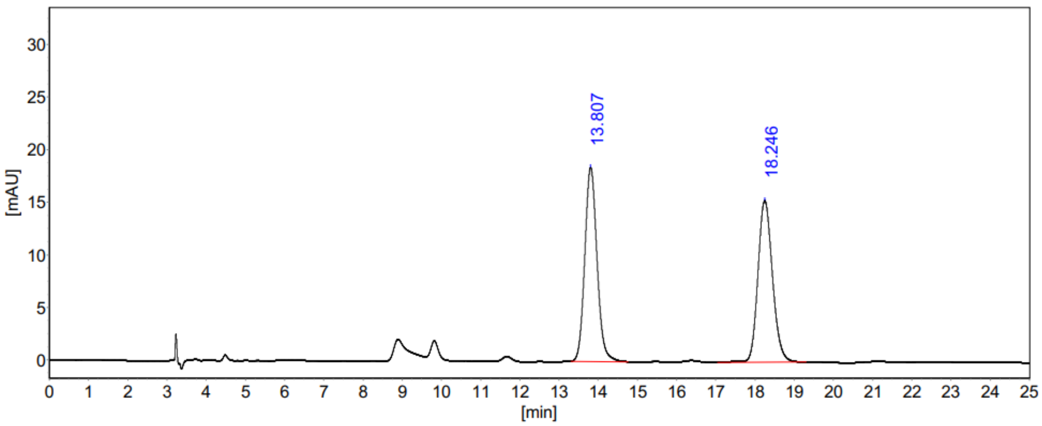

Analysis Results

No.	Compound	R.Time	Height	Area	Area%	Conc.	Type
1		13.807	18467.2	399658.2	50.2047	50.2047	+ BB
2		18.246	15366.9	396398.7	49.7953	49.7953	+ BB
	**Total:**		**33834.1**	**796056.9**	**100.0000**	**100.0000**	

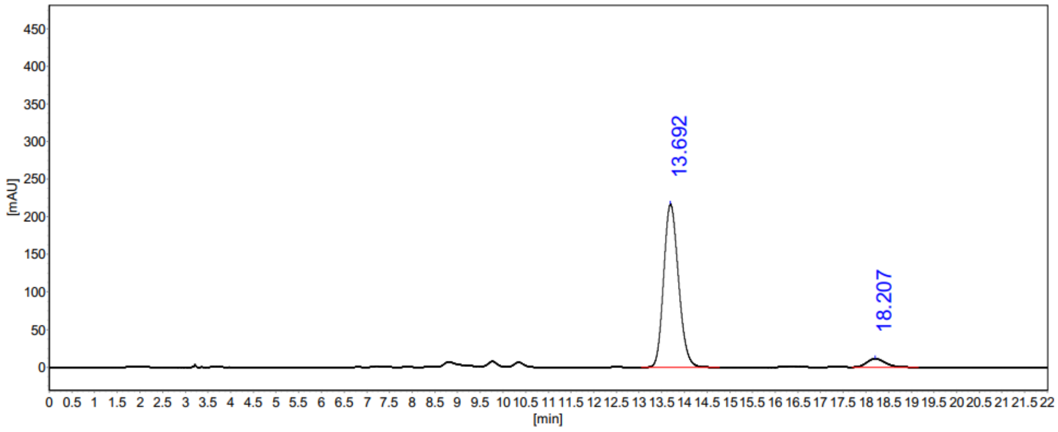

Analysis Results

No.	Compound	R.Time	Height	Area	Area%	Conc.	Type
1		13.692	217073.9	4927350.8	93.9986	93.9986	+ BB
2		18.207	11248.5	314587.2	6.0014	6.0014	+ BB
	**Total:**		**228322.4**	**5241938.1**	**100.0000**	**100.0000**	

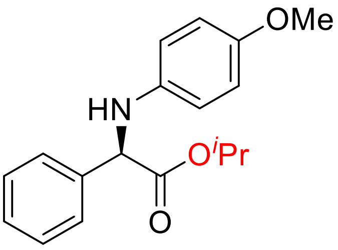

51

Comment:

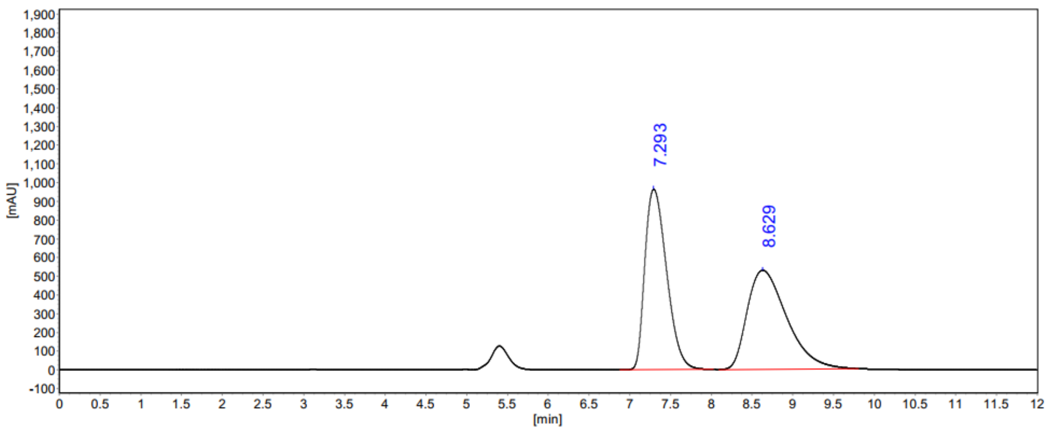

Analysis Results

No.	Compound	R.Time	Height	Area	Area%	Conc.	Type
1		7.293	964457.7	17909784.7	50.0682	50.0682	+ BB
2		8.629	528450.6	17861027.4	49.9318	49.9318	+ BB
	**Total:**		**1492908.3**	**35770812.0**	**100.0000**	**100.0000**	

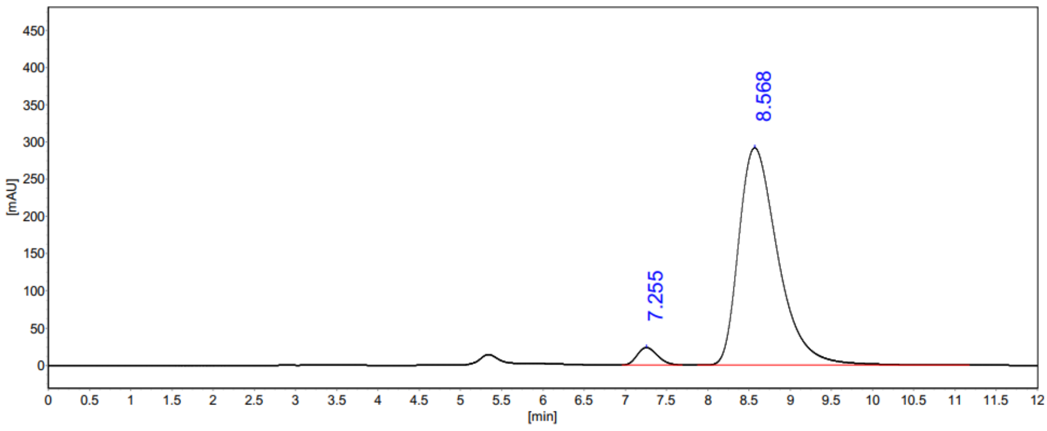

Analysis Results

No.	Compound	R.Time	Height	Area	Area%	Conc.	Type
1		7.255	23492.1	401204.0	3.9829	3.9829	+ BB
2		8.568	292269.4	9671987.5	96.0171	96.0171	+ BB
	**Total:**		**315761.5**	**10073191.5**	**100.0000**	**100.0000**	

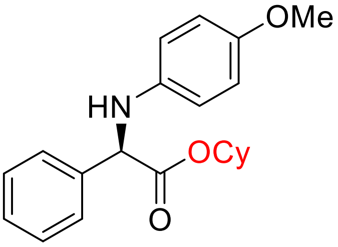

52

Comment:

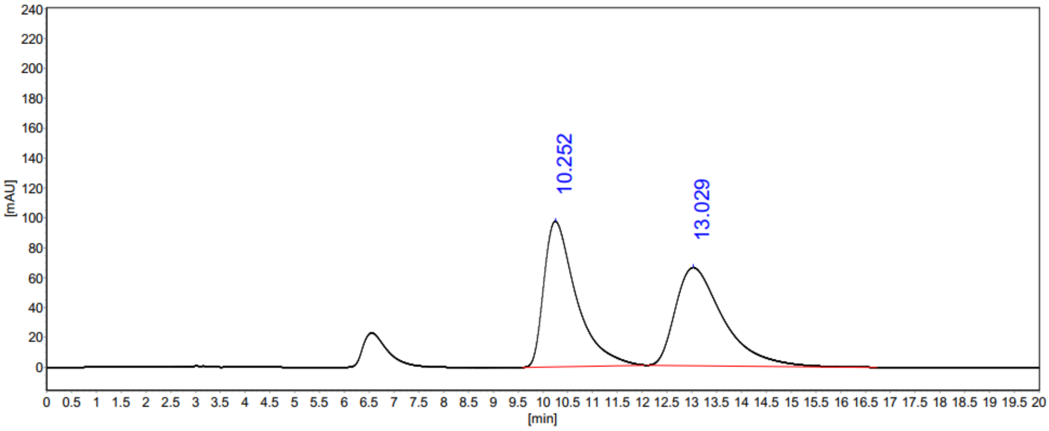

Analysis Results

No.	Compound	R.Time	Height	Area	Area%	Conc.	Type
1		10.252	97428.6	4410297.4	50.1615	50.1615	+ BB
2		13.029	65580.9	4381906.1	49.8385	49.8385	+ BB
	**Total:**		**163009.5**	**8792203.5**	**100.0000**	**100.0000**	

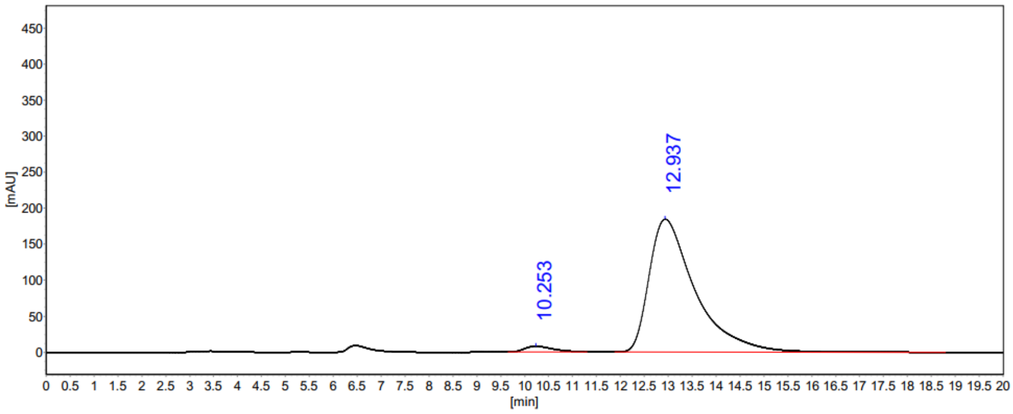

Analysis Results

No.	Compound	R.Time	Height	Area	Area%	Conc.	Type
1		10.253	6066.4	186101.7	1.4845	1.4845	+ BB
2		12.937	184612.8	12349903.1	98.5155	98.5155	+ BB
	**Total:**		**190679.2**	**12536004.9**	**100.0000**	**100.0000**	

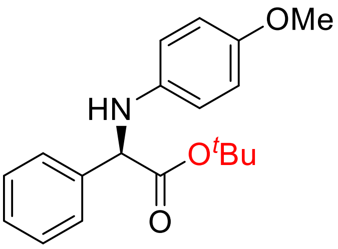

53

Comment:

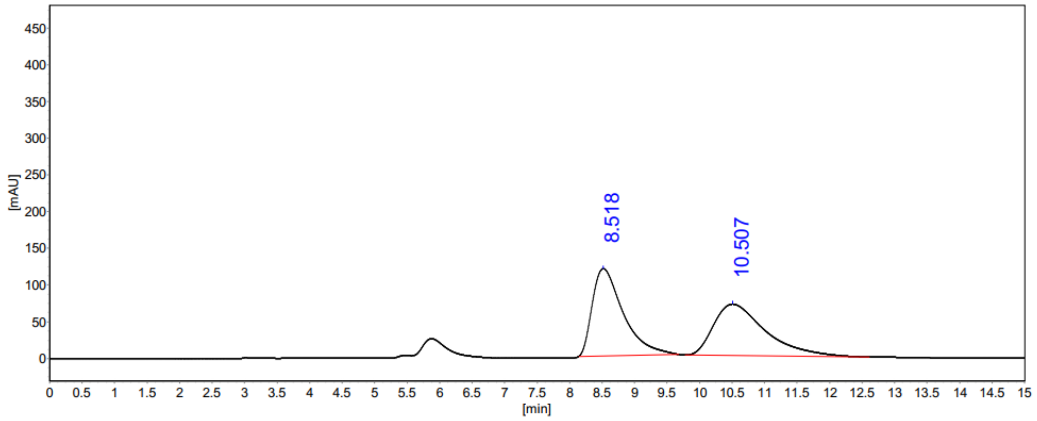

Analysis Results

No.	Compound	R.Time	Height	Area	Area%	Conc.	Type
1		8.518	119000.4	3932630.5	50.2167	50.2167	+ BB
2		10.507	70142.6	3898684.9	49.7833	49.7833	+ BB
	**Total:**		**189143.0**	**7831315.4**	**100.0000**	**100.0000**	

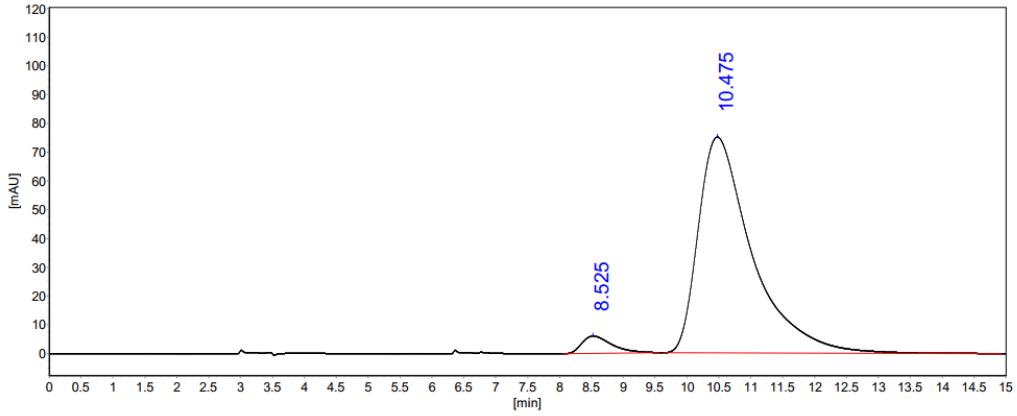

Analysis Results

No.	Compound	R.Time	Height	Area	Area%	Conc.	Type
1		8.525	6037.9	199040.6	4.3224	4.3224	+ BB
2		10.475	75063.7	4405825.7	95.6776	95.6776	+ BB
	**Total:**		**81101.6**	**4604866.3**	**100.0000**	**100.0000**	

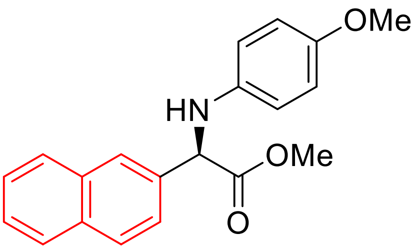

54

Comment:

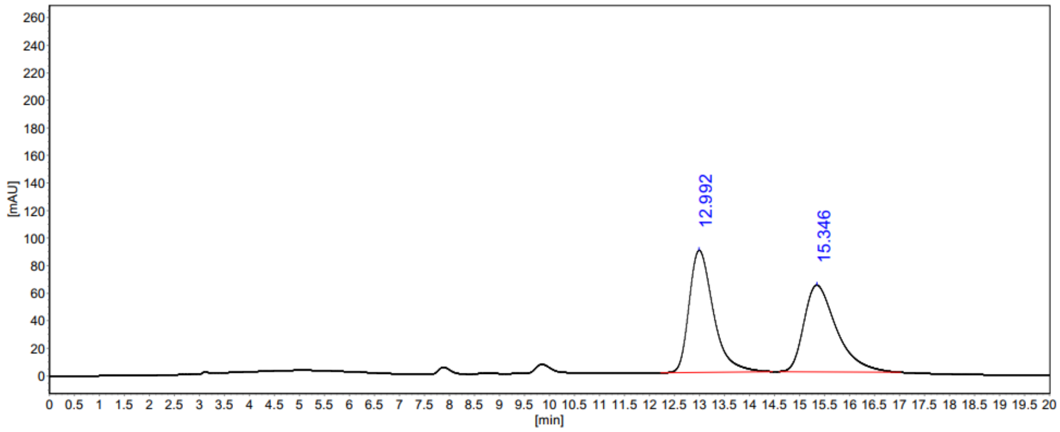

Analysis Results

No.	Compound	R.Time	Height	Area	Area%	Conc.	Type
1		12.992	88635.5	2978843.6	50.9619	50.9619	+ BB
2		15.346	62909.5	2866395.3	49.0381	49.0381	+ BB
	**Total:**		**151545.0**	**5845238.9**	**100.0000**	**100.0000**	

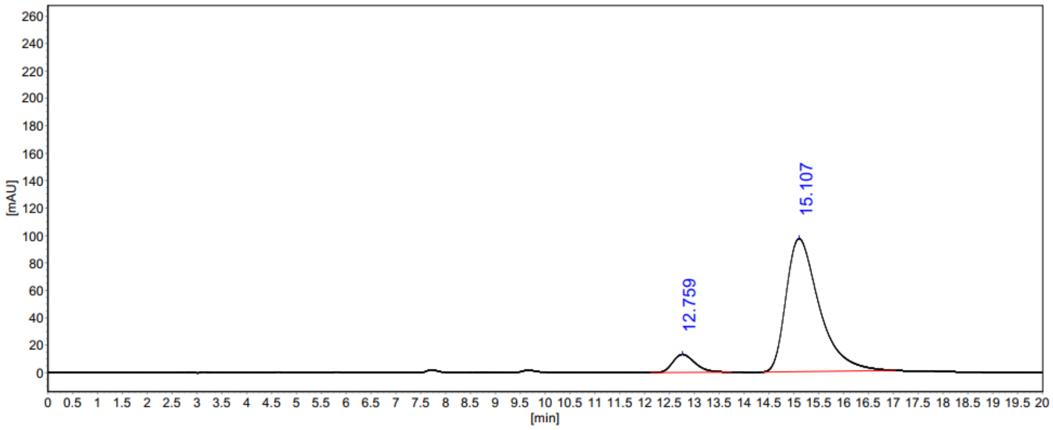

Analysis Results

No.	Compound	R.Time	Height	Area	Area%	Conc.	Type
1		12.759	13110.9	409369.9	8.3147	8.3147	+ BB
2		15.107	97039.9	4514106.8	91.6853	91.6853	+ BB
	**Total:**		**110150.8**	**4923476.7**	**100.0000**	**100.0000**	

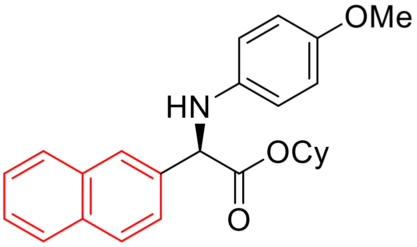

55

Comment:

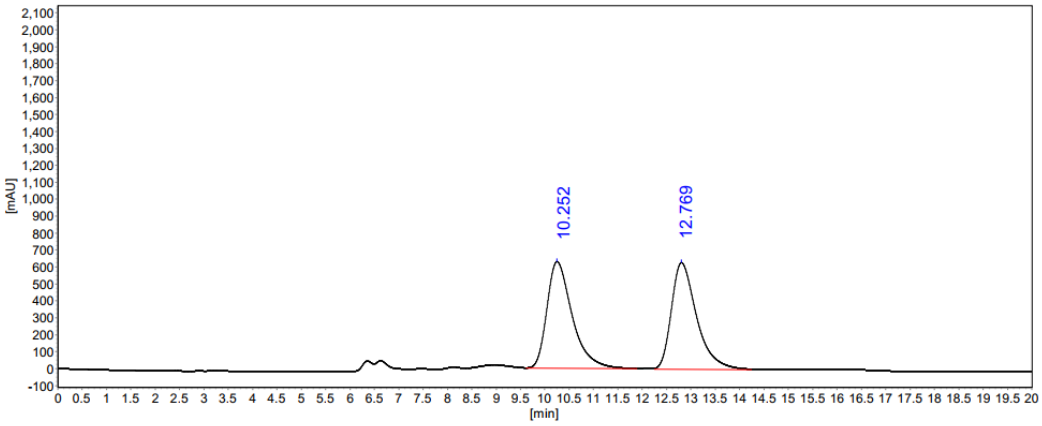

Analysis Results

No.	Compound	R.Time	Height	Area	Area%	Conc.	Type
1		10.252	627745.0	22578395.2	50.4988	50.4988	+ BB
2		12.769	259126.4	22132378.4	49.5012	49.5012	+ BB
	**Total:**		**886871.4**	**44710773.6**	**100.0000**	**100.0000**	

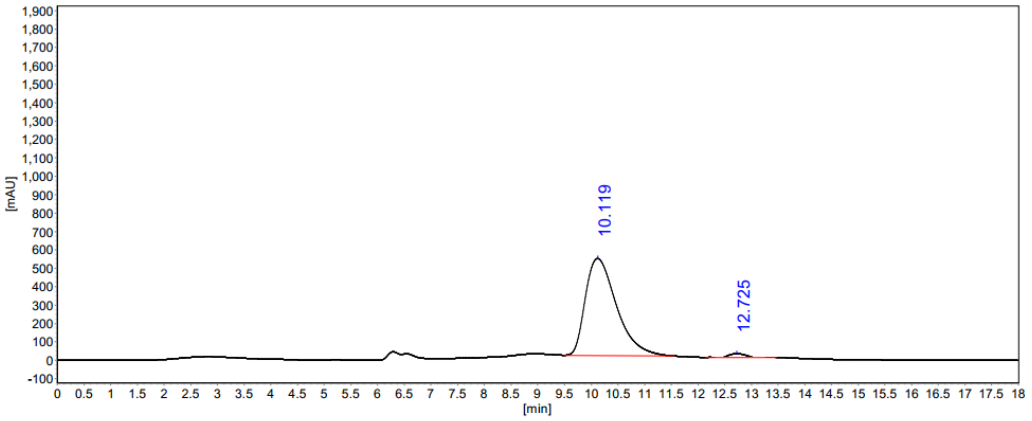

Analysis Results

No.	Compound	R.Time	Height	Area	Area%	Conc.	Type
1		10.119	526920.6	21390902.3	97.4557	97.4557	+ BB
2		12.725	43033.1	1538056.7	2.5443	2.5443	+ BB
	**Total:**		**569953.7**	**22928959.0**	**100.0000**	**100.0000**	

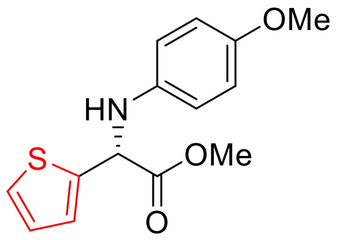

56

Comment:

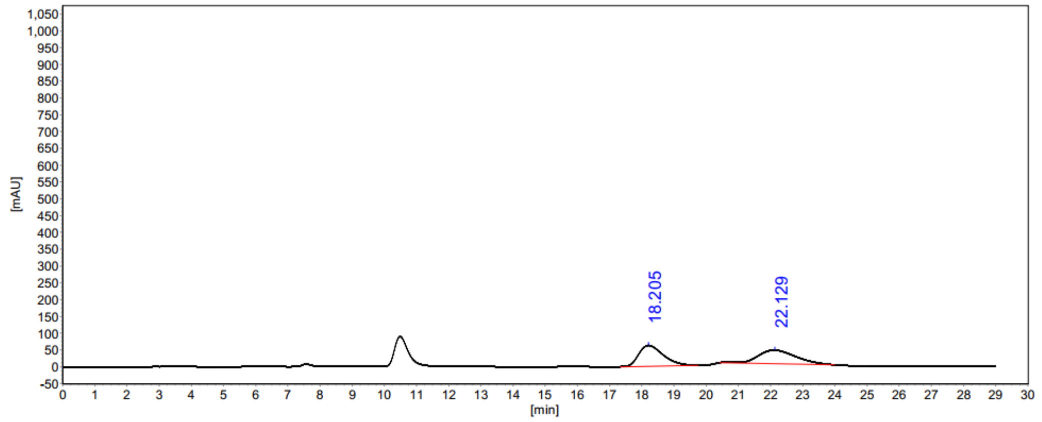

Analysis Results

No.	Compound	R.Time	Height	Area	Area%	Conc.	Type
1		18.205	62053.3	3316915.9	49.5861	49.5861	+ BB
2		22.129	40322.6	3372285.1	50.4139	50.4139	+ BB
	**Total:**		**102375.9**	**6689201.0**	**100.0000**	**100.0000**	

Comment:

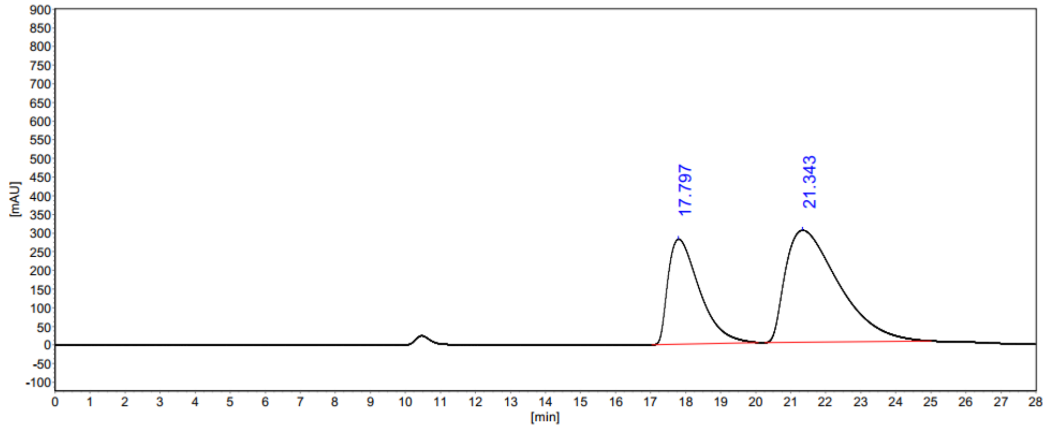

Analysis Results

No.	Compound	R.Time	Height	Area	Area%	Conc.	Type
1		17.797	281855.0	17609321.6	35.6153	35.6153	+ BB
2		21.343	300610.0	31833868.2	64.3847	64.3847	+ BB
	**Total:**		**582465.0**	**49443189.8**	**100.0000**	**100.0000**	

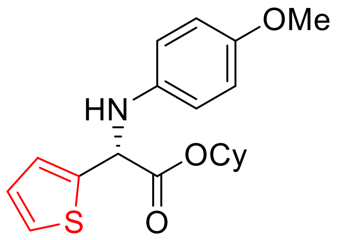

57

Comment:

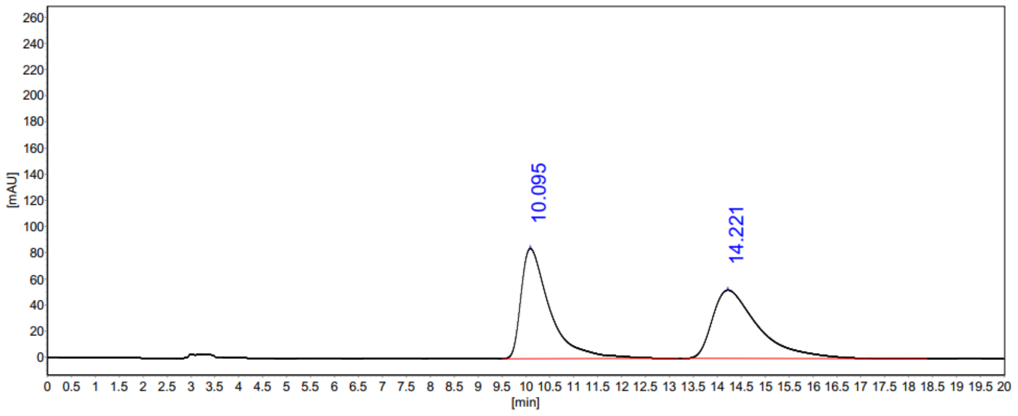

Analysis Results

No.	Compound	R.Time	Height	Area	Area%	Conc.	Type
1		10.095	84166.6	3529003.7	50.0911	50.0911	+ BB
2		14.221	52278.9	3516163.0	49.9089	49.9089	+ BB
	**Total:**		**136445.5**	**7045166.7**	**100.0000**	**100.0000**	

Comment:

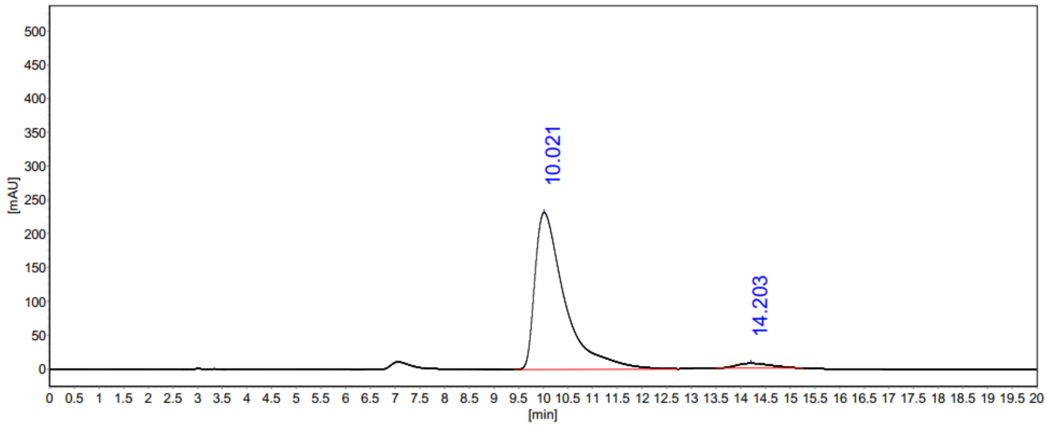

Analysis Results

No.	Compound	R.Time	Height	Area	Area%	Conc.	Type
1		10.021	231987.0	9723134.1	96.4494	96.4494	+ BB
2		14.203	7147.2	357942.8	3.5506	3.5506	+ BB
	**Total:**		**239134.2**	**10081076.9**	**100.0000**	**100.0000**	

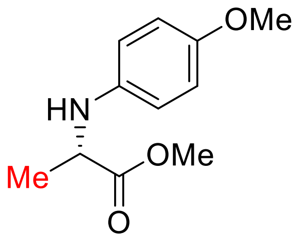

58

Comment:

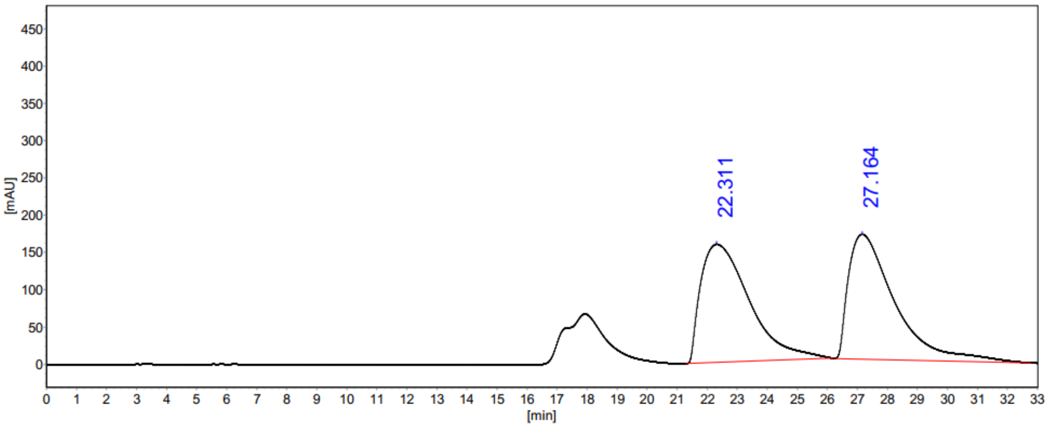

Analysis Results

No.	Compound	R.Time	Height	Area	Area%	Conc.	Type
1		22.311	158577.4	17892048.7	50.0402	50.0402	+ BB
2		27.164	167349.5	17863281.1	49.9598	49.9598	+ BB
	**Total:**		**325926.9**	**35755329.8**	**100.0000**	**100.0000**	

Comment:

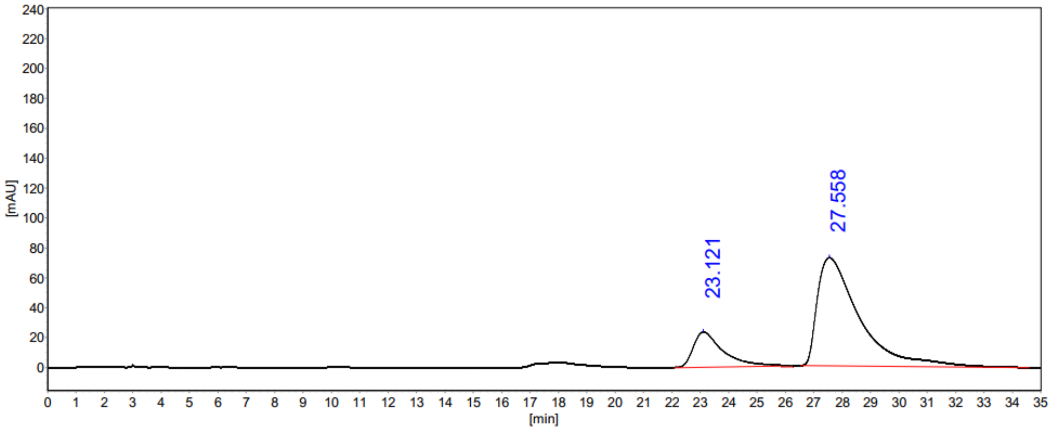

Analysis Results

No.	Compound	R.Time	Height	Area	Area%	Conc.	Type
1		23.121	19854.2	1303697.8	14.8563	14.8563	+ BB
2		27.558	72255.9	7471673.5	85.1437	85.1437	+ BB
	**Total:**		**92110.1**	**8775371.3**	**100.0000**	**100.0000**	

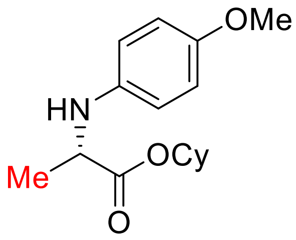

59

Comment:

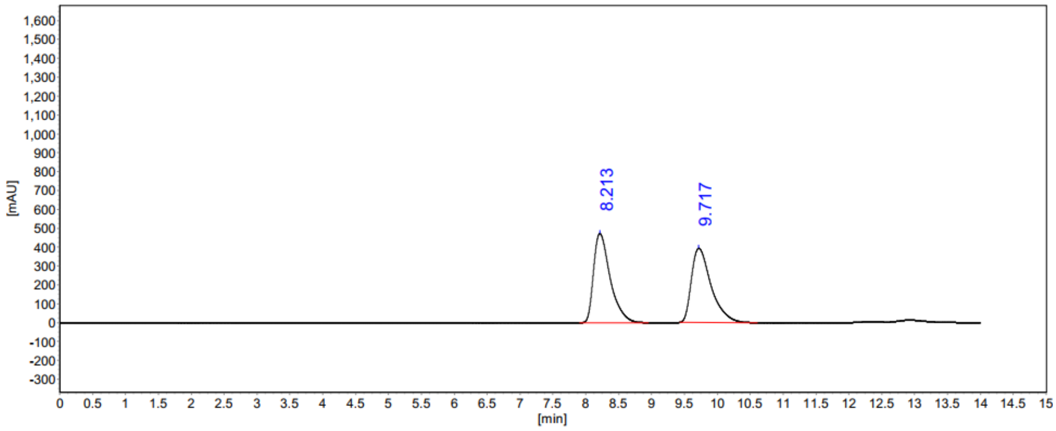

Analysis Results

No.	Compound	R.Time	Height	Area	Area%	Conc.	Type
1		8.213	472335.3	8322814.3	50.4214	50.4214	+ BB
2		9.717	393677.7	8183685.8	49.5786	49.5786	+ BB
	**Total:**		**866013.0**	**16506500.1**	**100.0000**	**100.0000**	

Comment:

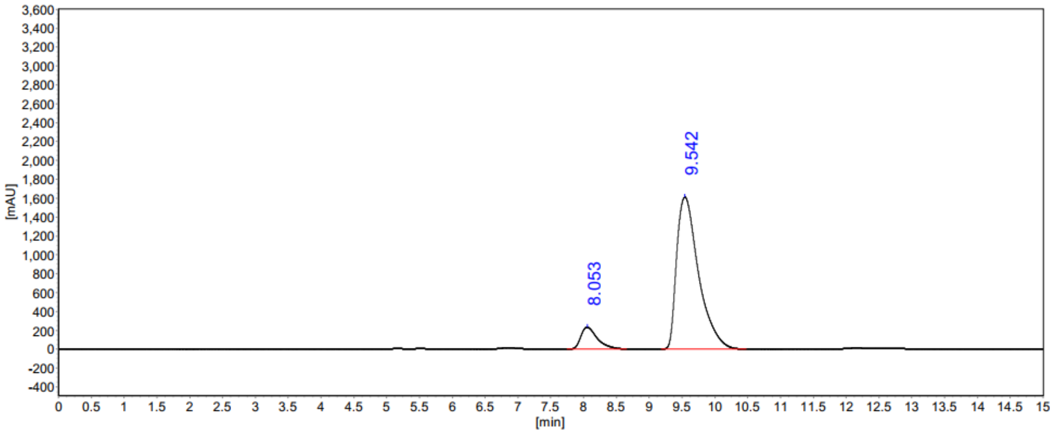

Analysis Results

No.	Compound	R.Time	Height	Area	Area%	Conc.	Type
1		8.053	231647.9	4052521.9	9.7924	9.7924	+ BB
2		9.542	1611534.8	37331944.5	90.2076	90.2076	+ BB
	**Total:**		**1843182.7**	**41384466.4**	**100.0000**	**100.0000**	

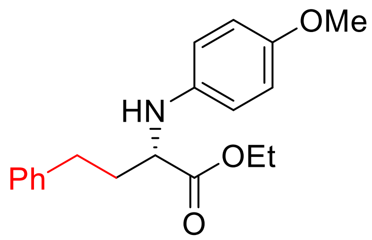

60

Comment:

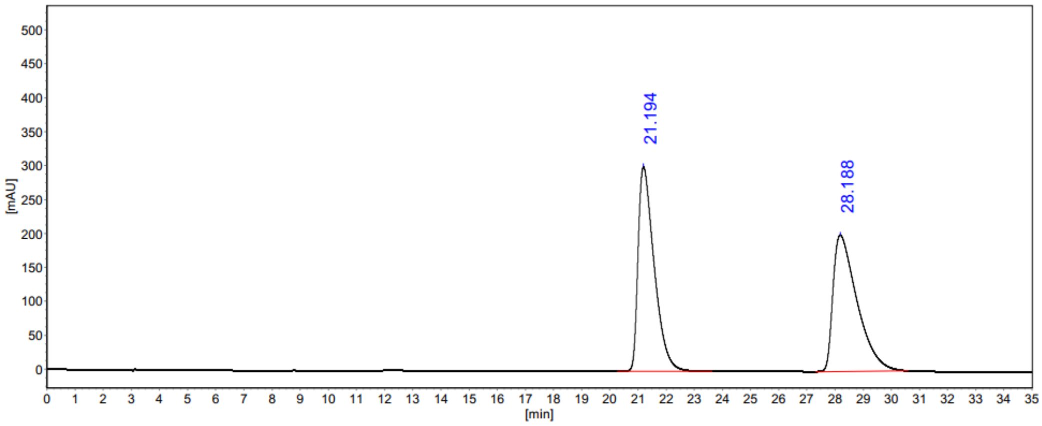

Analysis Results

No.	Compound	R.Time	Height	Area	Area%	Conc.	Type
1		21.194	301281.9	11954627.6	50.0431	50.0431	+ BB
2		28.188	201015.6	11934031.7	49.9569	49.9569	+ BB
	**Total:**		**502297.5**	**23888659.3**	**100.0000**	**100.0000**	

Comment:

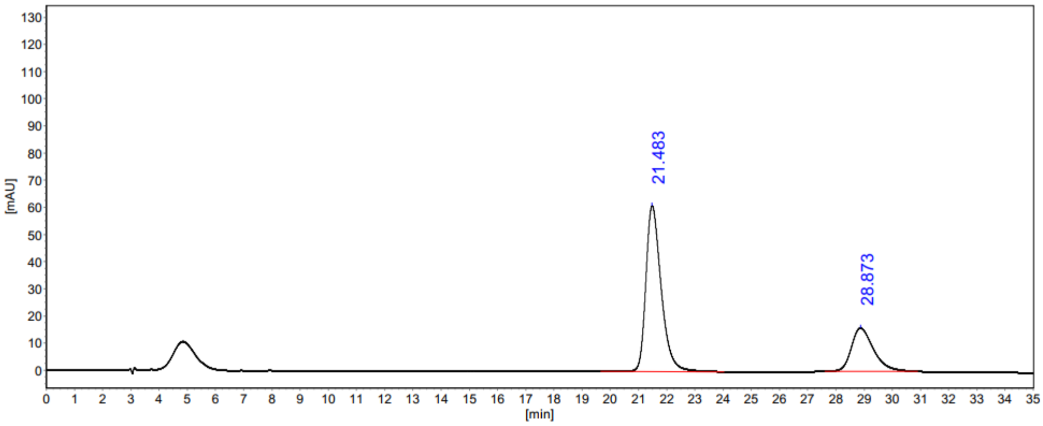

Analysis Results

No.	Compound	R.Time	Height	Area	Area%	Conc.	Type
1		21.483	61126.6	2306662.6	72.4141	72.4141	+ BB
2		28.873	15968.6	878716.3	27.5859	27.5859	+ BB
	**Total:**		**77095.2**	**3185378.9**	**100.0000**	**100.0000**	

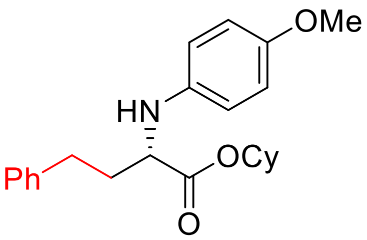

61

Comment:

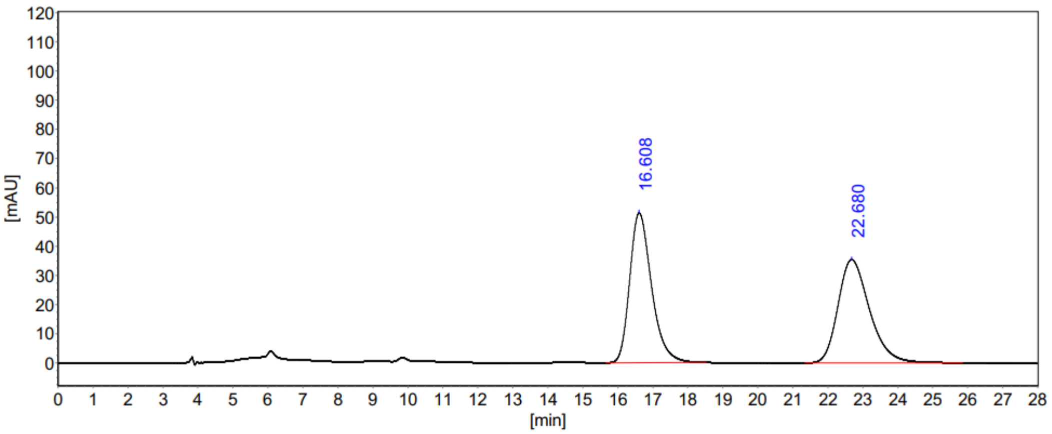

Analysis Results

No.	Compound	R.Time	Height	Area	Area%	Conc.	Type
1		16.608	51330.0	2224810.8	50.0409	50.0409	+ BB
2		22.680	35396.8	2221174.4	49.9591	49.9591	+ BB
	**Total:**		**86726.8**	**4445985.2**	**100.0000**	**100.0000**	

Comment:

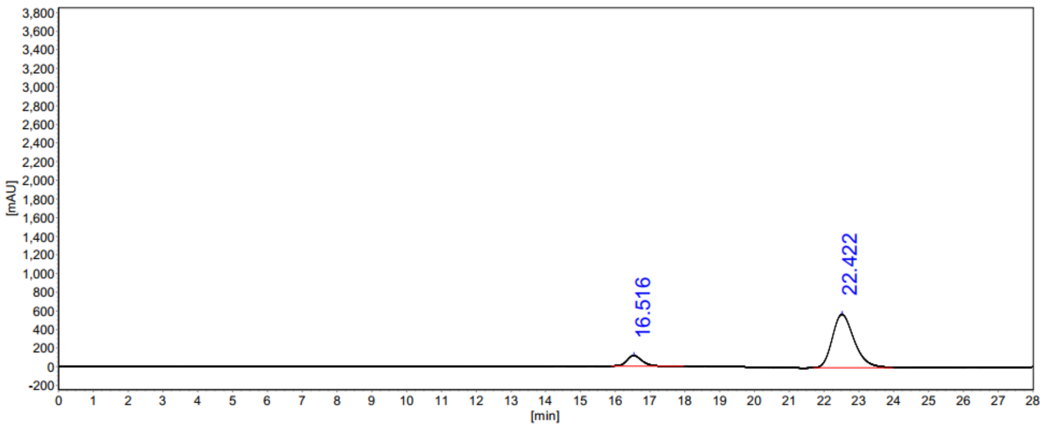

Analysis Results

No.	Compound	R.Time	Height	Area	Area%	Conc.	Type
1		16.516	27538.0	883299.4	16.4139	16.4139	+ BB
2		22.422	74297.8	4498112.2	83.5861	83.5861	+ BB
	**Total:**		**101835.8**	**5381411.6**	**100.0000**	**100.0000**	

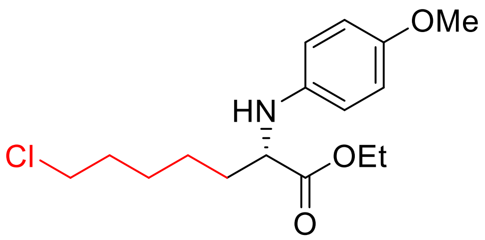

62

Comment:

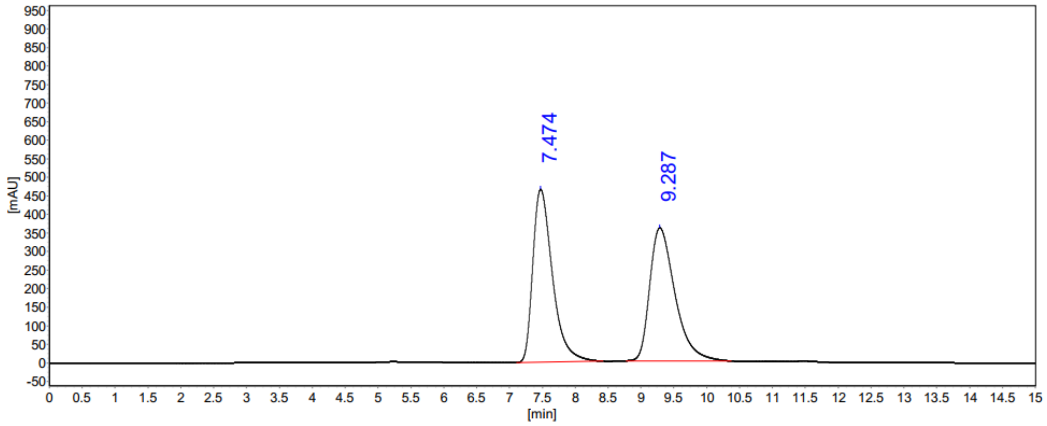

Analysis Results

No.	Compound	R.Time	Height	Area	Area%	Conc.	Type
1		7.474	466299.1	9681816.9	50.3084	50.3084	+ BB
2		9.287	358702.6	9563118.4	49.6916	49.6916	+ BB
	**Total:**		**825001.7**	**19244935.3**	**100.0000**	**100.0000**	

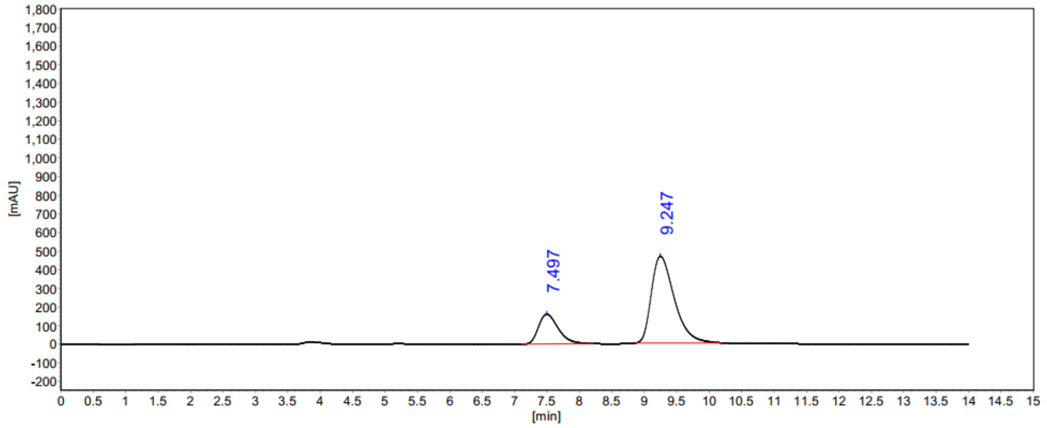

Analysis Results

No.	Compound	R.Time	Height	Area	Area%	Conc.	Type
1		7.497	159838.7	3399511.8	22.8524	22.8524	+ BB
2		9.247	466340.6	11476451.7	77.1476	77.1476	+ BB
	**Total:**		**626179.3**	**14875963.5**	**100.0000**	**100.0000**	

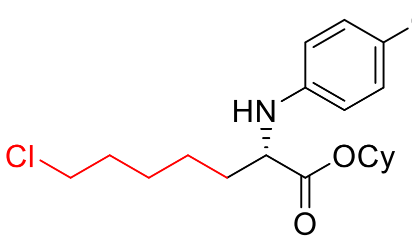

63

Comment:

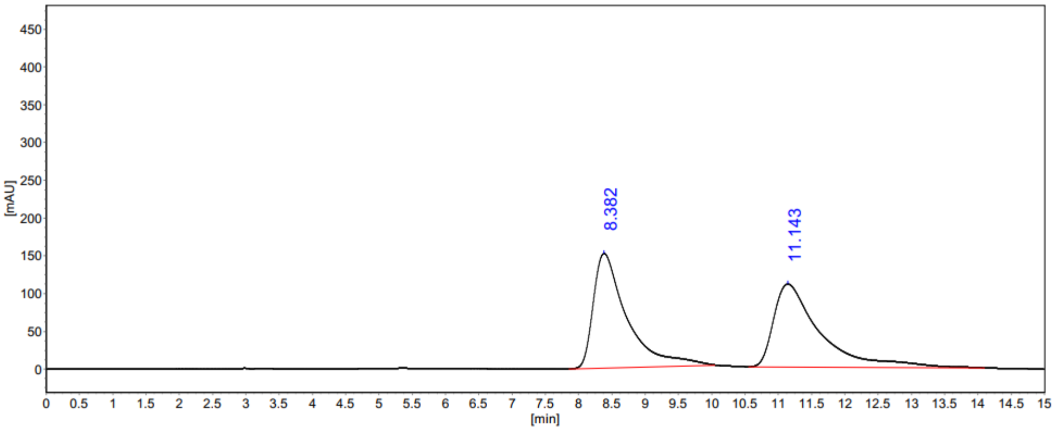

Analysis Results

No.	Compound	R.Time	Height	Area	Area%	Conc.	Type
1		8.382	151620.9	5313460.1	50.0630	50.0630	+ BB
2		11.143	109647.5	5300093.8	49.9370	49.9370	+ BB
	**Total:**		**261268.4**	**10613553.9**	**100.0000**	**100.0000**	

Comment:

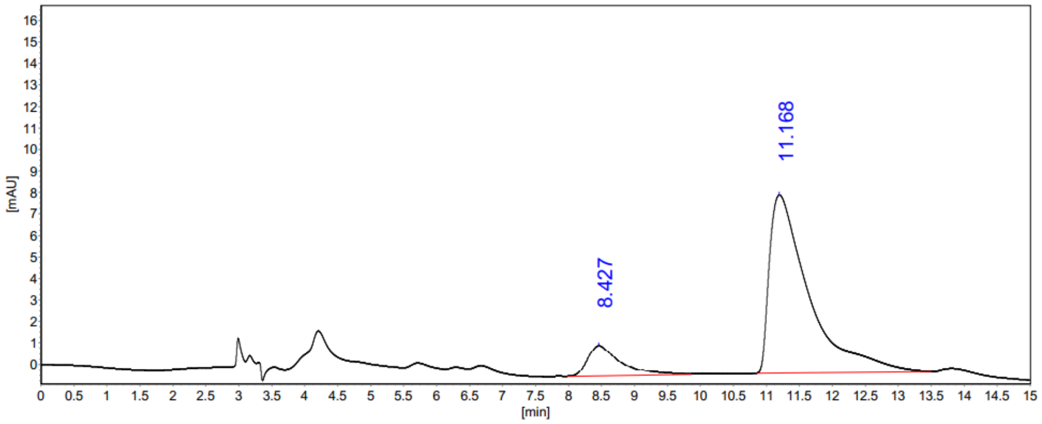

Analysis Results

No.	Compound	R.Time	Height	Area	Area%	Conc.	Type
1		8.427	2399.2	47417.4	8.8862	8.8862	+ BB
2		11.168	9677.5	486189.2	91.1138	91.1138	+ BB
	**Total:**		**12076.7**	**533606.6**	**100.0000**	**100.0000**	

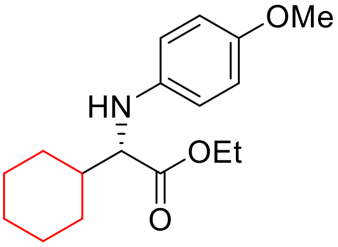

64

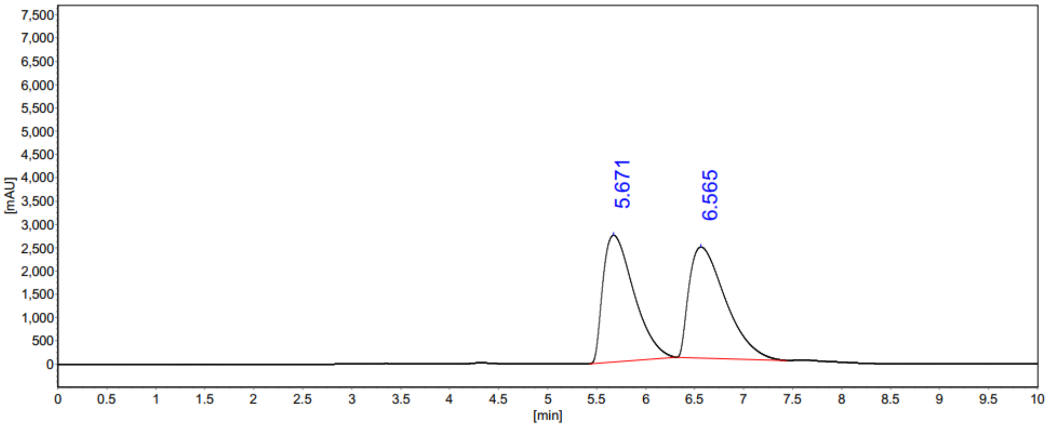

Analysis Results

No.	Compound	R.Time	Height	Area	Area%	Conc.	Type
1		5.671	2719776.4	59671390.8	49.1720	49.1720	+ BB
2		6.565	2383686.0	61680917.8	50.8280	50.8280	+ BB
	**Total:**		**5103462.4**	**121352308.6**	**100.0000**	**100.0000**	

Comment:

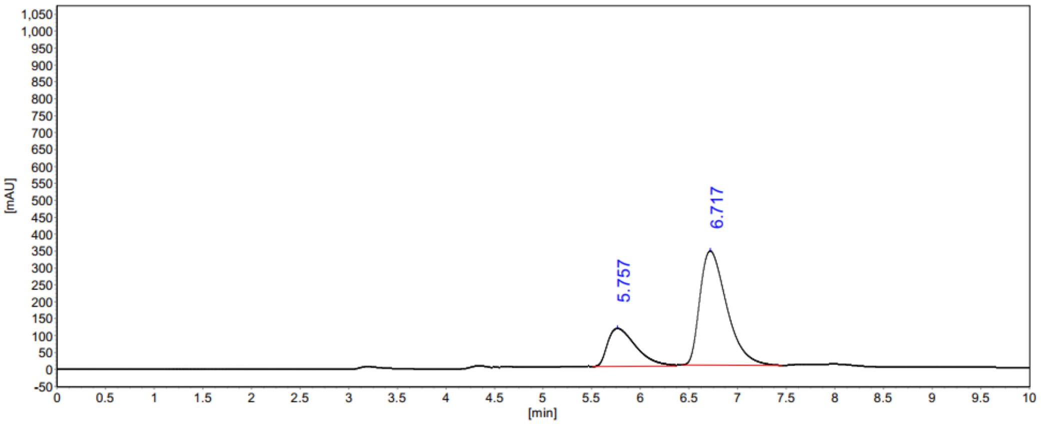

Analysis Results

No.	Compound	R.Time	Height	Area	Area%	Conc.	Type
1		5.757	228175.1	3324416.5	34.0006	34.0006	+ BB
2		6.717	337283.6	6453116.3	65.9994	65.9994	+ BB
	**Total:**		**565458.7**	**9777532.7**	**100.0000**	**100.0000**	

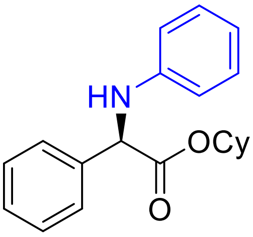

65

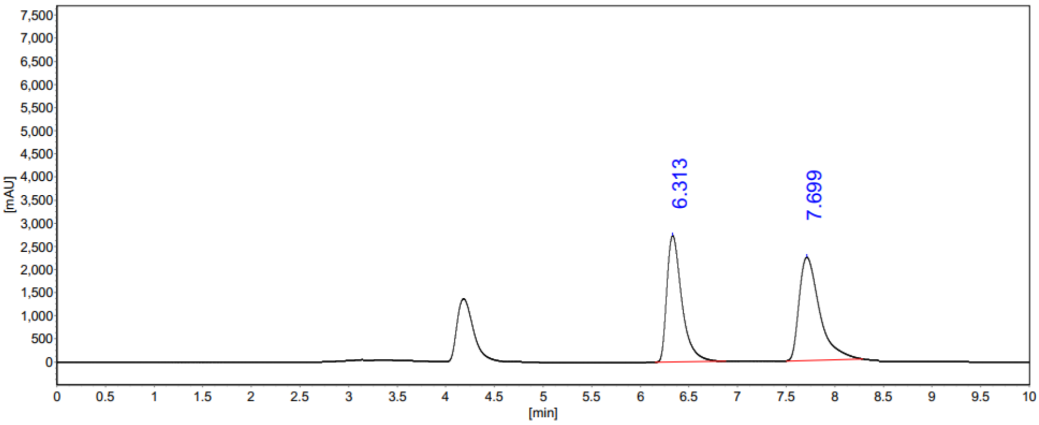

Analysis Results

No.	Compound	R.Time	Height	Area	Area%	Conc.	Type
1		6.313	1367221.2	16300019.2	49.7434	49.7434	+ BB
2		7.699	1246368.6	16468185.2	50.2566	50.2566	+ BB
	**Total:**		**2613589.8**	**32768204.4**	**100.0000**	**100.0000**	

Comment:

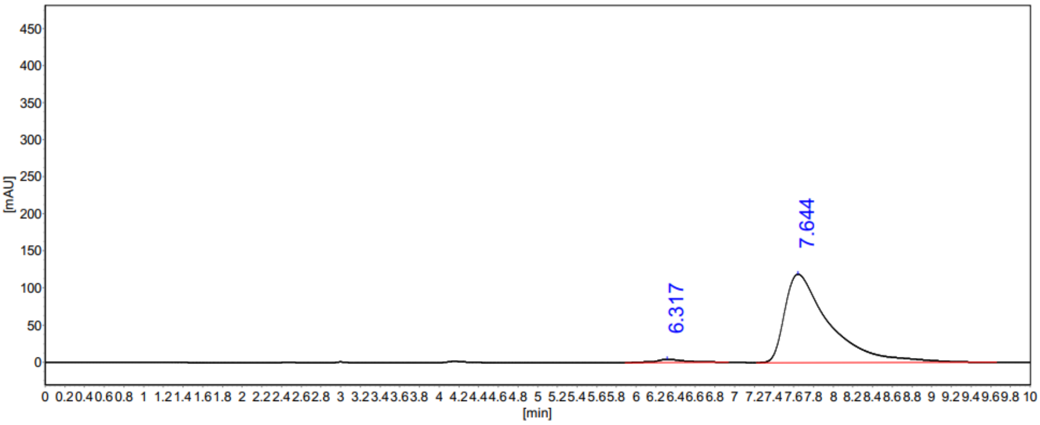

Analysis Results

No.	Compound	R.Time	Height	Area	Area%	Conc.	Type
1		6.317	4316.4	93810.2	2.4347	2.4347	+ BB
2		7.644	119140.0	3759261.4	97.5653	97.5653	+ BB
	**Total:**		**123456.4**	**3853071.6**	**100.0000**	**100.0000**	

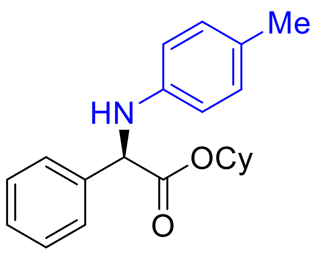

66

Comment:

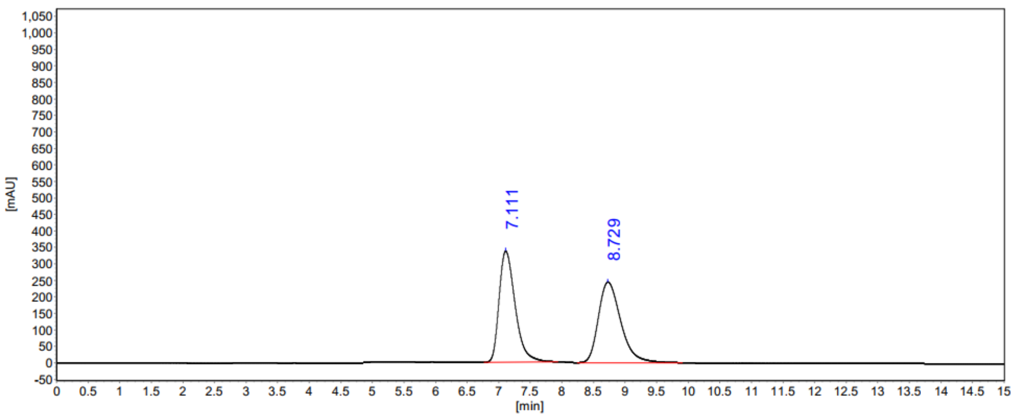

Analysis Results

No.	Compound	R.Time	Height	Area	Area%	Conc.	Type
1		7.111	336805.5	5959667.8	49.7096	49.7096	+ BB
2		8.729	244434.8	6029311.7	50.2904	50.2904	+ BB
	**Total:**		**581240.3**	**11988979.5**	**100.0000**	**100.0000**	

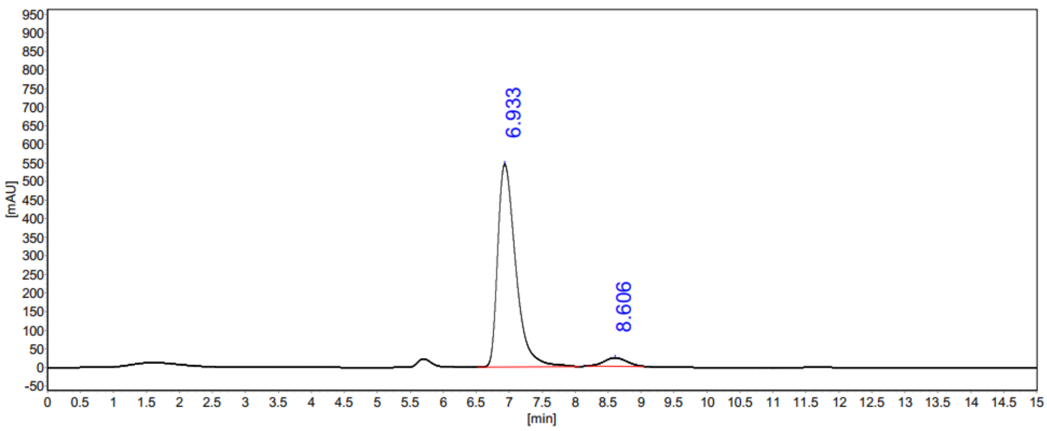

Analysis Results

No.	Compound	R.Time	Height	Area	Area%	Conc.	Type
1		6.933	545844.1	10398392.0	95.0044	95.0044	+ BB
2		8.606	22766.1	546780.1	4.9956	4.9956	+ BB
	**Total:**		**568610.2**	**10945172.0**	**100.0000**	**100.0000**	

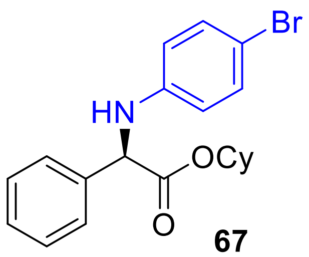

67

Comment:

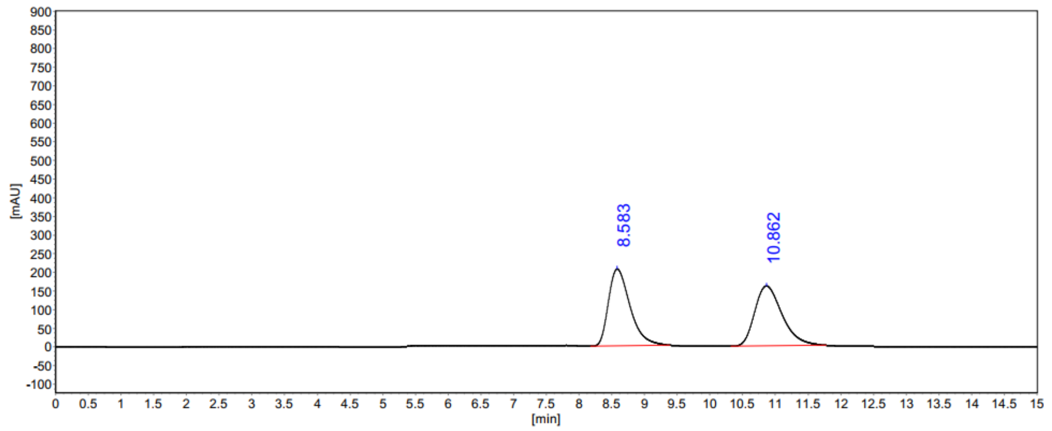

Analysis Results

No.	Compound	R.Time	Height	Area	Area%	Conc.	Type
1		8.583	206374.3	4749541.5	50.2135	50.2135	+ BB
2		10.862	160910.9	4709152.6	49.7865	49.7865	+ BB
	**Total:**		**367285.2**	**9458694.1**	**100.0000**	**100.0000**	

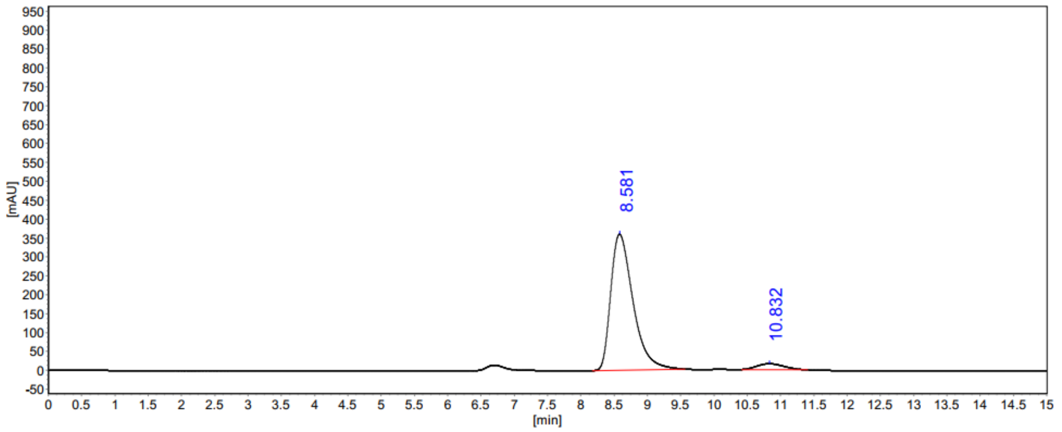

Analysis Results

No.	Compound	R.Time	Height	Area	Area%	Conc.	Type
1		8.581	359810.3	8357857.9	94.8852	94.8852	+ BB
2		10.832	15786.1	450533.0	5.1148	5.1148	+ BB
	**Total:**		**375596.4**	**8808390.9**	**100.0000**	**100.0000**	

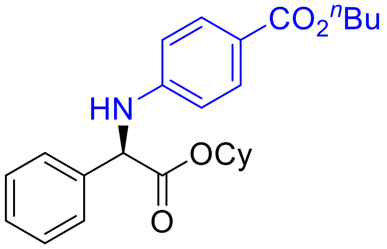

68

Comment:

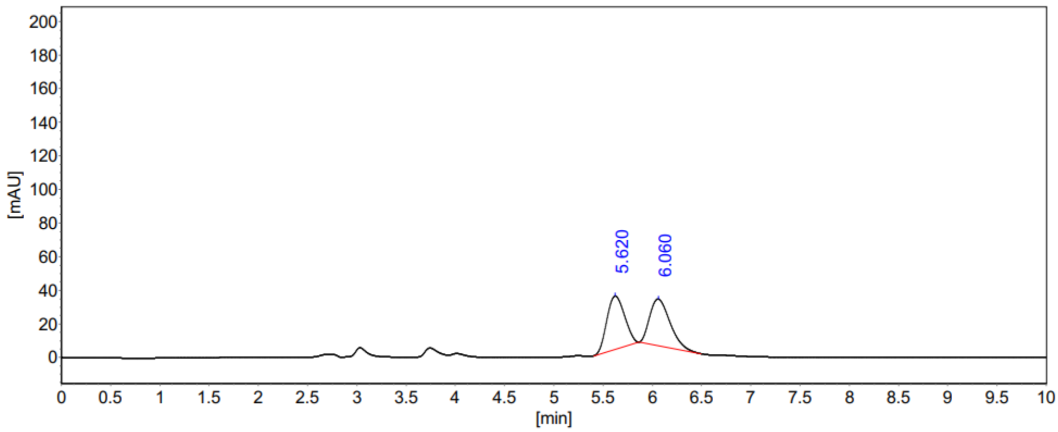

Analysis Results

No.	Compound	R.Time	Height	Area	Area%	Conc.	Type
1		5.620	31795.1	398474.9	49.7930	49.7930	+ BB
2		6.060	27844.3	401788.8	50.2070	50.2070	+ BB
	**Total:**		**59639.4**	**800263.7**	**100.0000**	**100.0000**	

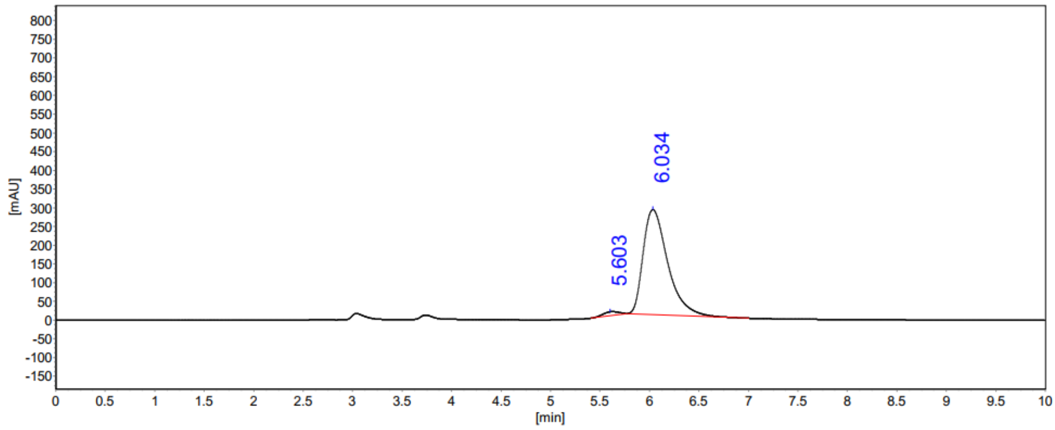

Analysis Results

No.	Compound	R.Time	Height	Area	Area%	Conc.	Type
1		5.603	10330.8	113436.0	2.2862	2.2862	+ BB
2		6.034	280310.0	4848264.1	97.7138	97.7138	+ BB
	**Total:**		**290640.8**	**4961700.1**	**100.0000**	**100.0000**	

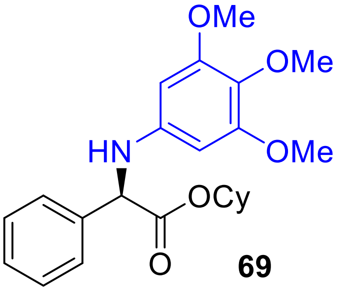

69

Comment:

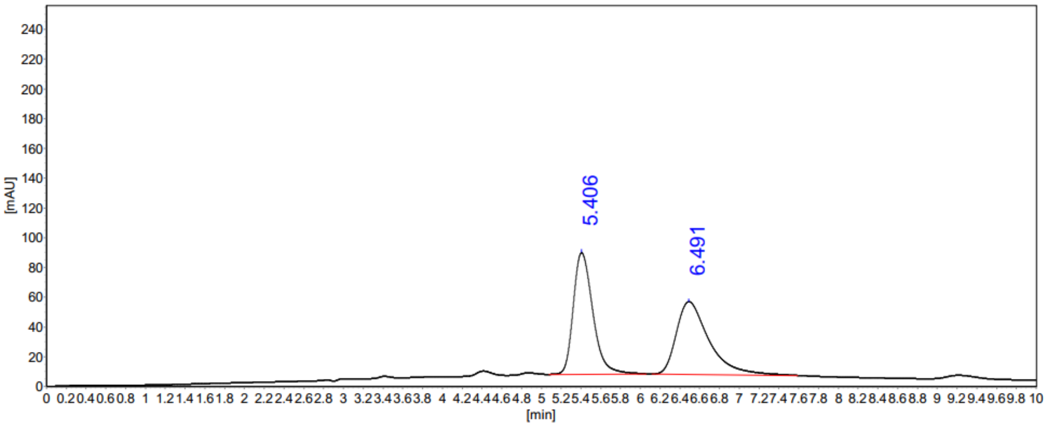

Analysis Results

No.	Compound	R.Time	Height	Area	Area%	Conc.	Type
1		5.406	81757.0	1122259.1	50.0054	50.0054	+ BB
2		6.491	48914.9	1122017.3	49.9946	49.9946	+ BB
	**Total:**		**130671.9**	**2244276.4**	**100.0000**	**100.0000**	

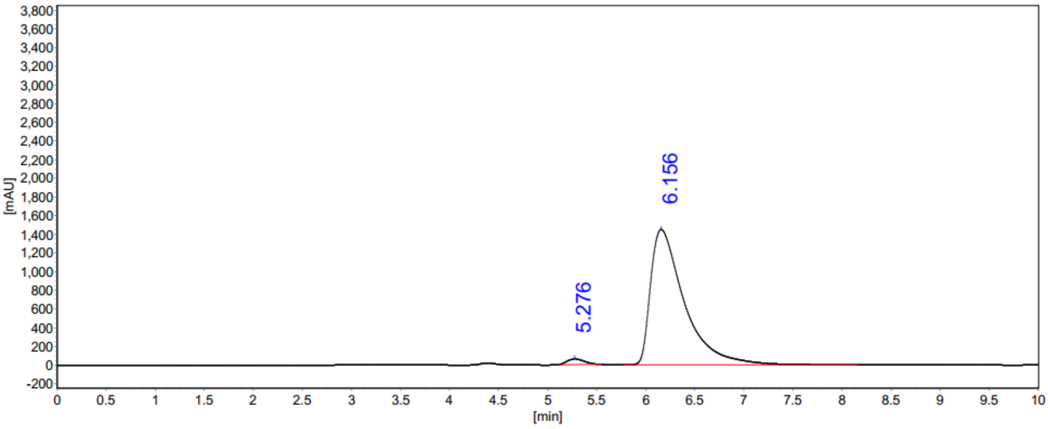

Analysis Results

No.	Compound	R.Time	Height	Area	Area%	Conc.	Type
1		5.276	59376.5	746602.5	2.1009	2.1009	+ BB
2		6.156	1449107.1	34790571.5	97.8991	97.8991	+ BB
	**Total:**		**1508483.6**	**35537174.1**	**100.0000**	**100.0000**	

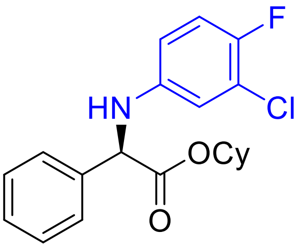

70

Comment:

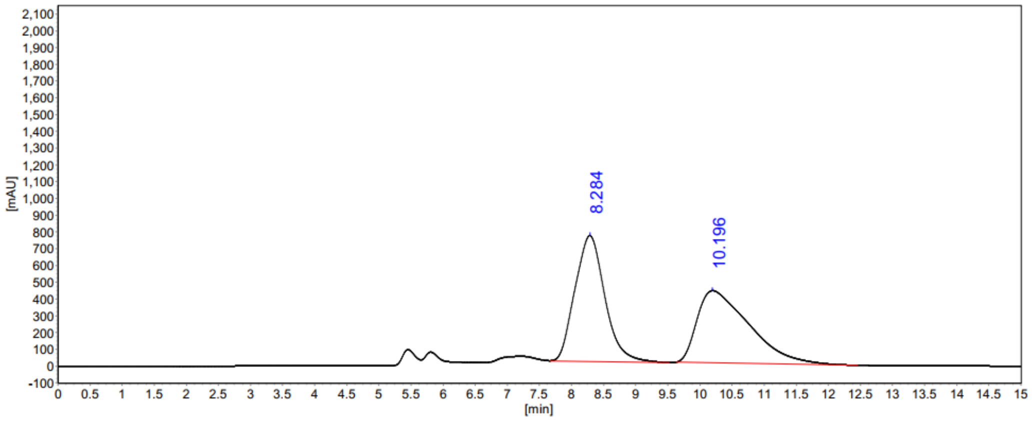

Analysis Results

No.	Compound	R.Time	Height	Area	Area%	Conc.	Type
1		8.284	750575.8	24297535.0	49.9978	49.9978	+ BB
2		10.196	430795.9	24299665.2	50.0022	50.0022	+ BB
	**Total:**		**1181371.7**	**48597200.3**	**100.0000**	**100.0000**	

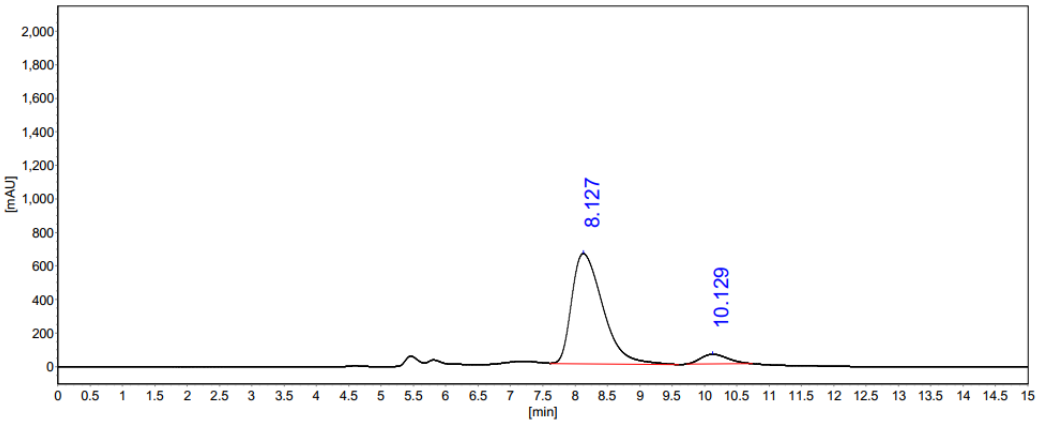

Analysis Results

No.	Compound	R.Time	Height	Area	Area%	Conc.	Type
1		8.127	656657.1	21847129.1	92.8623	92.8623	+ BB
2		10.129	57542.0	1679246.2	7.1377	7.1377	+ BB
	**Total:**		**714199.1**	**23526375.3**	**100.0000**	**100.0000**	

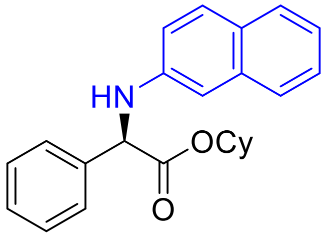

71

Comment:

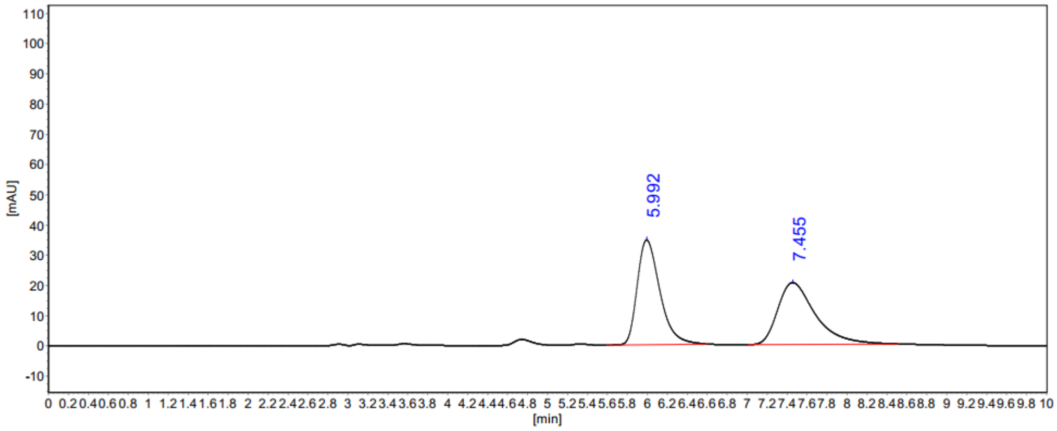

Analysis Results

No.	Compound	R.Time	Height	Area	Area%	Conc.	Type
1		5.992	34642.2	545603.7	50.4330	50.4330	+ BB
2		7.455	20459.9	536235.0	49.5670	49.5670	+ BB
	**Total:**		**55102.1**	**1081838.7**	**100.0000**	**100.0000**	

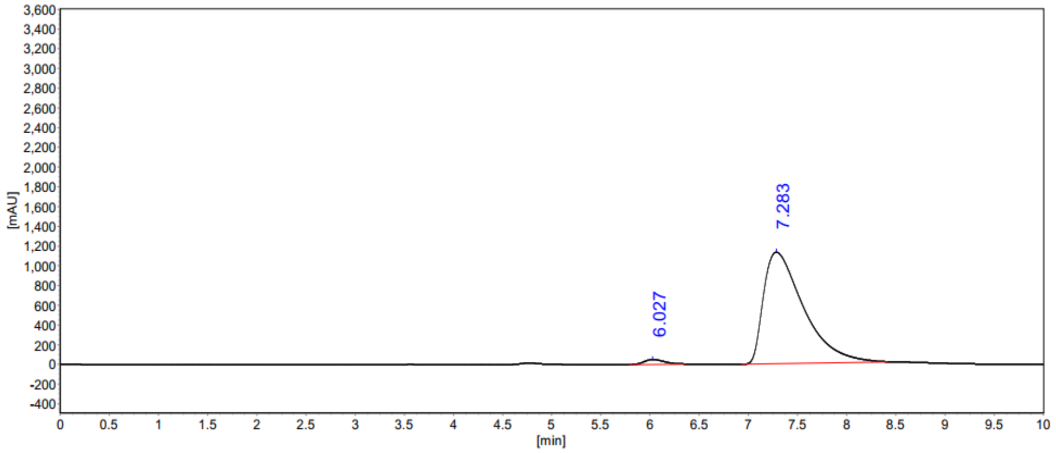

Analysis Results

No.	Compound	R.Time	Height	Area	Area%	Conc.	Type
1		6.027	48251.8	670696.8	2.0469	2.0469	+ BB
2		7.283	1131401.4	32095491.6	97.9531	97.9531	+ BB
	**Total:**		**1179653.2**	**32766188.4**	**100.0000**	**100.0000**	

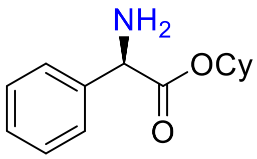

73

Comment:

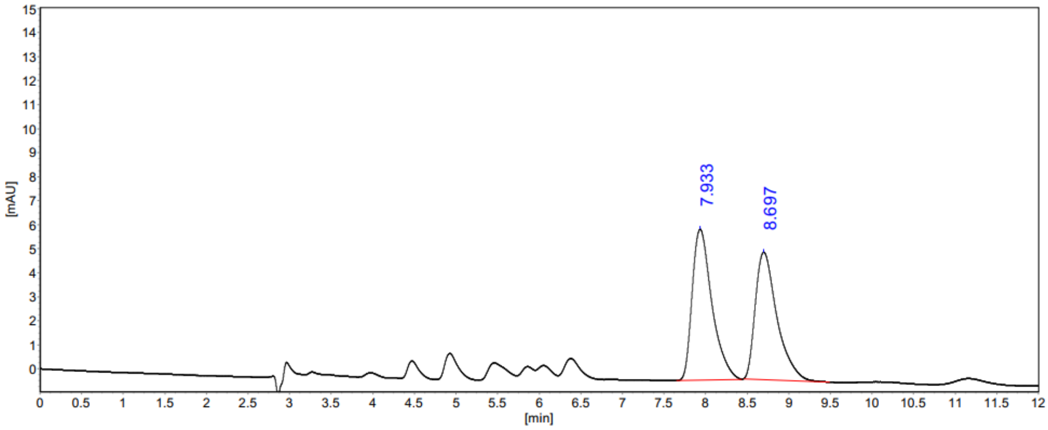

Analysis Results

No.	Compound	R.Time	Height	Area	Area%	Conc.	Type
1		7.933	6283.8	103714.6	51.8811	51.8811	+ BB
2		8.697	5301.8	96193.6	48.1189	48.1189	+ BB
	**Total:**		**11585.6**	**199908.2**	**100.0000**	**100.0000**	

Comment:

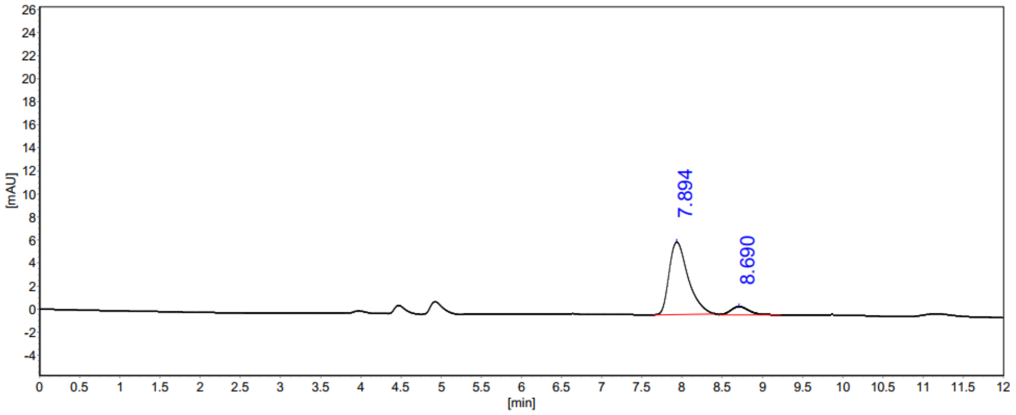

Analysis Results

No.	Compound	R.Time	Height	Area	Area%	Conc.	Type
1		7.894	423.9	7743.4	94.9628	94.9628	+ BB
2		8.690	46.5	410.7	5.0372	5.0372	+ BB
	**Total:**		**470.4**	**8154.2**	**100.0000**	**100.0000**	

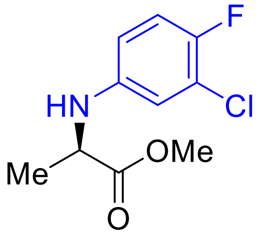

74

Comment:

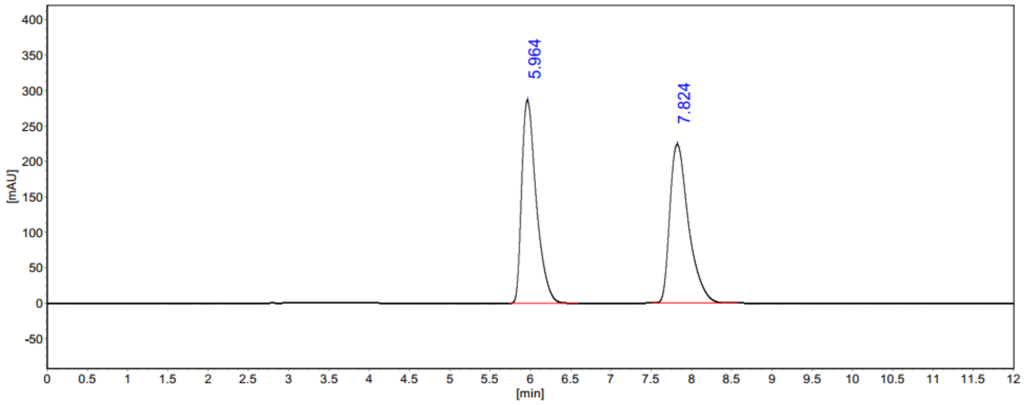

Analysis Results

No.	Compound	R.Time	Height	Area	Area%	Conc.	Type
1		5.964	286342.0	3603308.0	49.9879	49.9879	+ BB
2		7.824	223693.4	3605049.6	50.0121	50.0121	+ BB
	**Total:**		**510035.4**	**7208357.6**	**100.0000**	**100.0000**	

Comment:

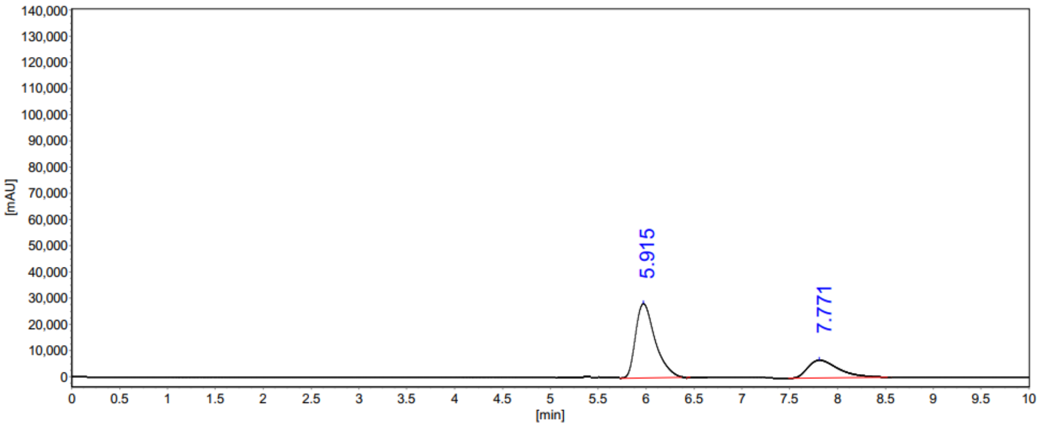

Analysis Results

No.	Compound	R.Time	Height	Area	Area%	Conc.	Type
1		5.915	2194237.8	31470874.1	82.0116	82.0116	+ BB
2		7.771	678506.4	6902799.9	17.9884	17.9884	+ BB
	**Total:**		**2872744.2**	**38373674.0**	**100.0000**	**100.0000**	

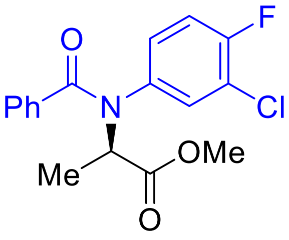

75

Comment:

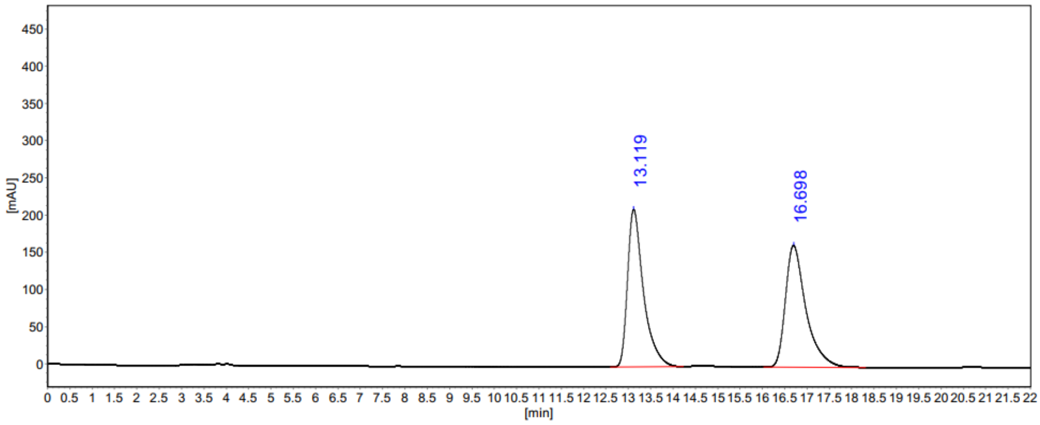

Analysis Results

No.	Compound	R.Time	Height	Area	Area%	Conc.	Type
1		13.119	211692.7	5257024.1	50.0664	50.0664	+ BB
2		16.698	163789.5	5243077.8	49.9336	49.9336	+ BB
	**Total:**		**375482.2**	**10500101.9**	**100.0000**	**100.0000**	

Comment:

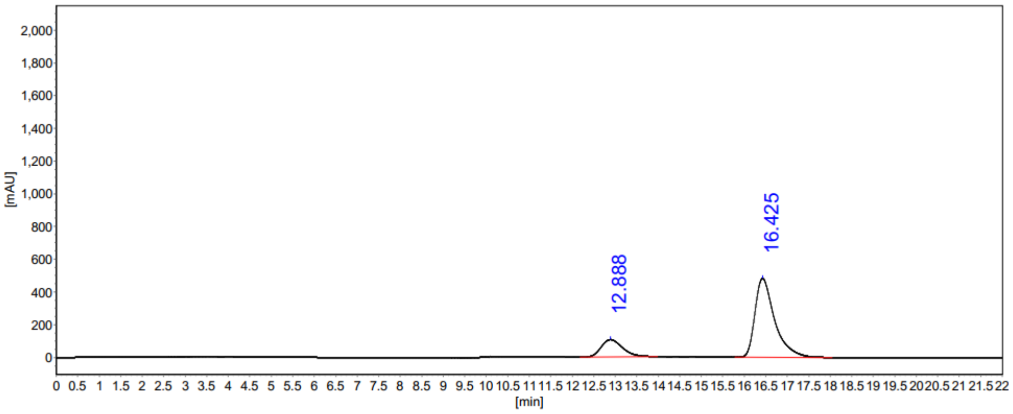

Analysis Results

No.	Compound	R.Time	Height	Area	Area%	Conc.	Type
1		12.888	229843.5	3512657.2	18.5274	18.5274	+ BB
2		16.425	483188.6	15446577.1	81.4726	81.4726	+ BB
	**Total:**		**713032.1**	**18959234.3**	**100.0000**	**100.0000**	
